# Siderophore conjugation with cleavable linkers boosts the potency of RNA polymerase inhibitors against multidrug-resistant *E. coli*†

**DOI:** 10.1039/d2sc06850h

**Published:** 2023-04-26

**Authors:** Carsten Peukert, Anna C. Vetter, Hazel L. S. Fuchs, Kirsten Harmrolfs, Bianka Karge, Marc Stadler, Mark Brönstrup

**Affiliations:** a Department of Chemical Biology, Helmholtz Centre for Infection Research Inhoffenstraße 7 38124 Braunschweig Germany Mark.Broenstrup@helmholtz-hzi.de; b Department of Microbial Drugs, Helmholtz Centre for Infection Research Inhoffenstraße 7 38124 Braunschweig Germany; c German Center for Infection Research (DZIF) Site Hannover-Braunschweig, Inhoffenstraße 7 38124 Braunschweig Germany; d Institute of Microbiology, Technische Universität Braunschweig Spielmannstraße 7 38106 Braunschweig Germany; e Institute for Organic Chemistry (IOC), Leibniz Universität Hannover Schneiderberg 1B 30167 Hannover Germany

## Abstract

The growing antibiotic resistance, foremost in Gram-negative bacteria, requires novel therapeutic approaches. We aimed to enhance the potency of well-established antibiotics targeting the RNA polymerase (RNAP) by utilizing the microbial iron transport machinery to improve drug translocation across their cell membrane. As covalent modifications resulted in moderate-low antibiotic activity, cleavable linkers were designed that permit a release of the antibiotic payload inside the bacteria and unperturbed target binding. A panel of ten cleavable siderophore–ciprofloxacin conjugates with systematic variation at the chelator and the linker moiety was used to identify the quinone trimethyl lock in conjugates 8 and 12 as the superior linker system, displaying minimal inhibitory concentrations (MICs) of ≤1 μM. Then, rifamycins, sorangicin A and corallopyronin A, representatives of three structurally and mechanistically different natural product RNAP inhibitor classes, were conjugated *via* the quinone linker to hexadentate hydroxamate and catecholate siderophores in 15–19 synthetic steps. MIC assays revealed an up to 32-fold increase in antibiotic activity against multidrug-resistant *E. coli* for conjugates such as 24 or 29 compared to free rifamycin. Experiments with knockout mutants in the transport system showed that translocation and antibiotic effects were conferred by several outer membrane receptors, whose coupling to the TonB protein was essential for activity. A functional release mechanism was demonstrated analytically by enzyme assays *in vitro*, and a combination of subcellular fractionation and quantitative mass spectrometry proved cellular uptake of the conjugate, release of the antibiotic, and its increased accumulation in the cytosol of bacteria. The study demonstrates how the potency of existing antibiotics against resistant Gram-negative pathogens can be boosted by adding functions for active transport and intracellular release.

## Abstract 2

RNA polymerase inhibitors against the bacterium *E. coli* become more potent when coupled to a siderophore enhancing cellular uptake, if the linker is cleaved inside the cell.

## Graphical abstract

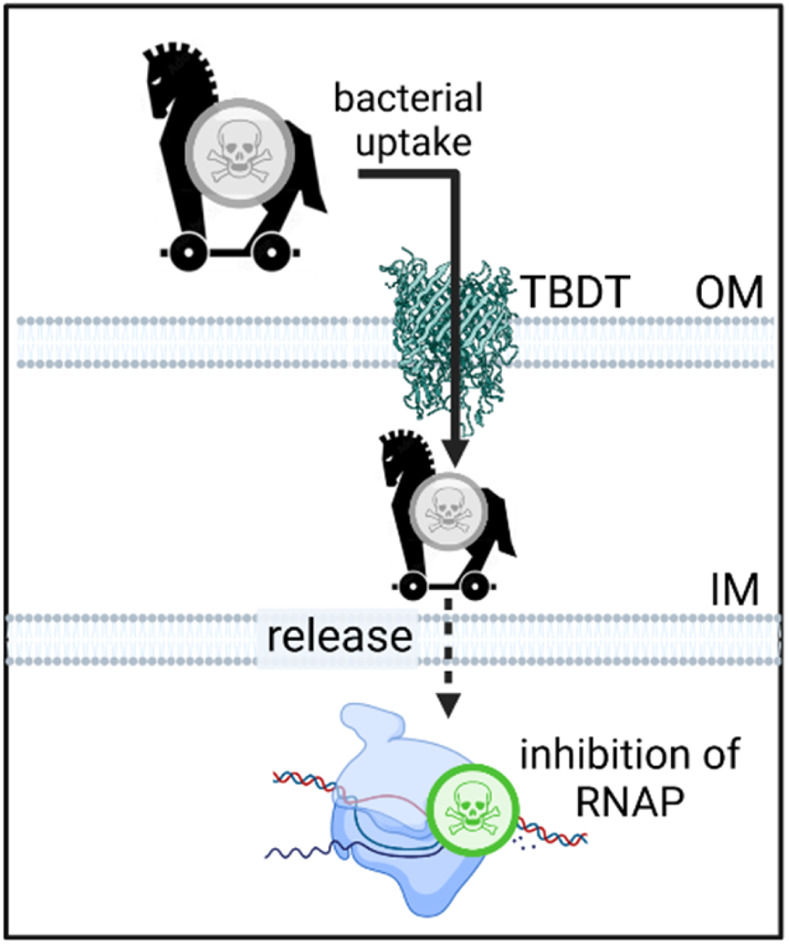

## Introduction

**Fig. 1:**
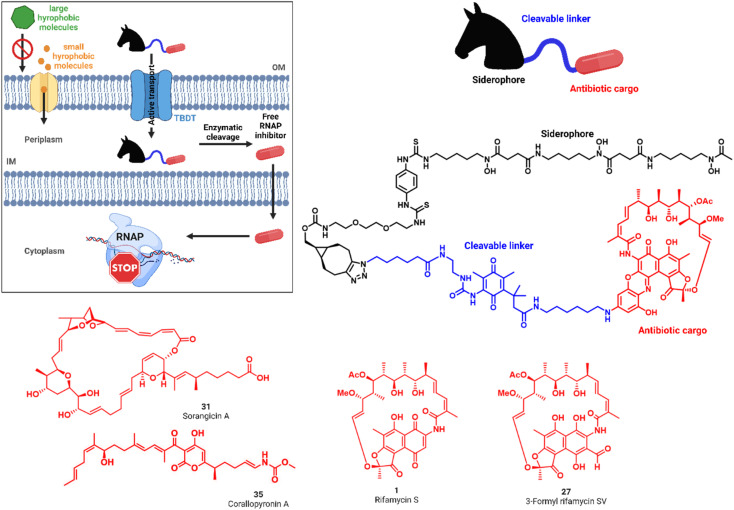
'Trojan Horse' strategy to increase the activity of antibiotics against Gram-negative pathogens. Siderophore ‘Trojan Horses’ (black) are attached through enzymatically cleavable linkers (blue) to Gram-positive only antibiotics (red) to convey antimicrobial activity. Internalization by bacterial TonB-dependent transporters (TBDTs) enables subsequent linker cleavage, payload release and antimicrobial action. Structures of the RNA polymerase inhibitors rifamycin S 1 and 3-formyl rifamycin SV 27, sorangicin A 31 and corallopyronin A 35 explored in this study.

Drug resistance in bacteria poses an increasing threat to public health that is exacerbated by the fact that only few new antimicrobials have reached approval and market launch in the past decades.^1–4^ Specifically Gram-negative microbes are a serious challenge, including four of the six ESKAPE pathogens (*Escherichia coli*, *Staphylococcus aureus*, *Klebsiella pneumoniae*, *Acinetobacter baumannii*, *Pseudomonas aeruginosa*, *Enterobacter faecium*).^5^ Their intrinsic resistance is conveyed by efflux pumps and a two-membrane cell envelope that results in low permeability of harmful molecules such as antibiotics.^6,7^ The outer membrane (OM) prevents the penetration of large, hydrophobic molecules. In contrast, the inner membrane (IM), together with a thin peptidoglycan layer, restricts the translocation of polar compounds (Fig. 1A).^8,9^ Uptake studies found that for some antibiotics the low accumulation into Gram-negative bacteria directly correlates with a low antimicrobial activity against the pathogen.^10^ The tightly controlled translocation across the Gram-negative cell wall is contrasted by the bacterial need for nutrients to survive in the environment or a eukaryotic host organism. Ferric iron (Fe^3+^) is an essential nutrient involved in central cellular processes such as DNA synthesis, ATP generation or protection against oxidative stress and thus plays a pivotal role in bacterial growth.^11^ To capture the metal from the environment, bacteria synthetize and secrete small molecule iron chelators, termed siderophores.^12^ TonB-dependent outer membrane transporters (TBDTs) internalize these ferric chelates into the bacterial periplasm. Numerous studies have exploited siderophores as carrier moieties in a ‘Trojan Horse’ strategy, in order to actively enrich antibiotics inside the bacterial pathogen.^13–17^ This principle has been validated clinically by the siderophore-containing cephalosporin antibiotic cefiderocol (Fetroja™) that recently obtained market authorization.^18,19^ While cefiderocol and the majority of conjugates use β-lactams acting in the periplasm, the transport to targets in the cytoplasm has been by and large – with few exceptions – unsuccessful so far.^16,17^ A path forward was demonstrated by Ji and Miller, who conjugated ciprofloxacin, targeting gyrase in the bacterial cytosol, *via* a cleavable trimethyl-lock linker to the natural trishydroxamate desferrioxamine (DFO) siderophore.^20^ We aimed to follow the concept of this pivotal study by combining three functionalities in one molecule: a siderophore vector, which is efficiently internalized by TBDTs, an enzymatically cleavable linker with widespread occurrence in both Gram-positive and Gram-negative bacteria, that serves to release the third component, a natural product-based RNA polymerase inhibitor (RNAP-I) (Fig. 1). We built on our previous synthetic and mechanistic work on siderophore mimics as bacterial targeting agents for diagnostic or therapeutic purposes.^21–23^ The triscatecholate DOTAM (1,4,7,10-tetraazacyclododecane–1,4,7,10-tetraacetic acid amide) core, harboring three catecholates, and DFO were chosen. Three different classes of RNAP inhibitors, represented by rifamycin S 1, sorangicin A 31 and corallopyronin 35, were employed as the antibiotic warheads (Fig. 1).

The RNA polymerase, the enzyme responsible for transcription of DNA into RNA, is the target of potent, clinically relevant ansamycin antibiotics such as rifampicin, derived semisynthetically from rifamycin S 1 or 3-formyl-rifamycin SV 27, which constitute a cornerstone in the treatment of tuberculosis.^24,25^ The polyketide sorangicin A (sorA, 31) was isolated from the myxobacterium *Sorangium cellulosum* and exhibited selective, potent activity against bacterial RNAPs.^26,27^ Despite targeting a similar binding site as rifampicin,^28^31 was found recently to inhibit wild-type and mutant RNAPs through different mechanisms.^29^ The third RNAP-I is corallopyronin A (corA, 35), isolated from *Corallococcus coralloides*, which bears the potential to overcome rifamycin resistance by binding to the switch region of the RNAP; the compound is currently in preclinical development for the treatment of filariasis.^30–32^ All natural products exhibit high activities against Gram-positive bacteria, but show 100–1000 fold increased minimal inhibitory concentrations (MICs) against Gram-negative species (Tables S1 and S2†).^33^ Although systematic investigations for the underlying cause of this difference are missing, we hypothesized that it is due to their difficulty to translocate across the Gram-negative cell wall.^34^ We aimed to improve the delivery of these RNAP-Is to their cytosolic target in Gram-negative bacteria by active internalization through bacterial transporters and subsequent enzymatic release, as reported below.

## Results and discussion

### Mono- and dicatechol-conjugated rifamycins show a moderate potency gain in Gram-negative bacteria

**Fig. 2:**
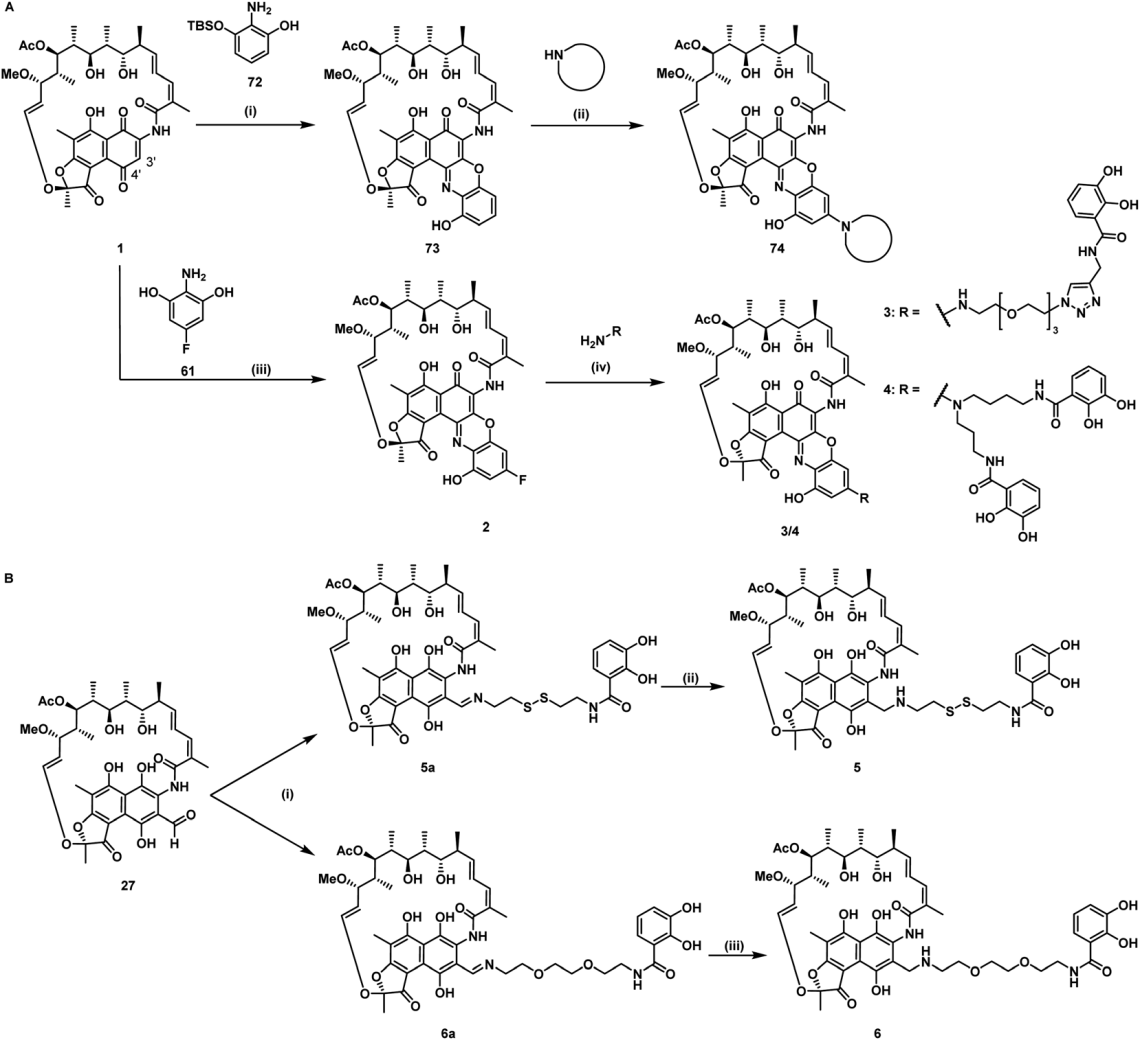
Synthesis of mono and dicatechol rifamycin derivatives 3–6. (A) Amine-functionalized rifamycin S derivatives: (i) toluene, 22 °C, 19 h, then MnO_2_, EtOH, 22 °C, 23 h, (ii) MnO_2_, 65 °C, low yield,^40^ (iii) 61, under argon, 1,4-benzoquinone or TEMPO, oxygen, iPrOAc, 23 °C, 48 h, yield for Ar/BQ/O_2_: 21% and Ar/TEMPO/O_2_: 50%, (iv) DIPEA, DMSO, THF, 25–60 °C, 30–55%.^42^ (B) Monocatechol derivatives of 3-formyl rifamycin SV 27: (i) 40 or 41, TEA, THF, 0–23 °C, 93–98%, 1 h, (ii/iii) NaBH(OAc)_3_, 1 h, 0–23 °C, 57–79%. For detailed information, please refer to the ESI (Fig. S1 and S2†).

For siderophore conjugation especially mild synthetic strategies needed to be developed, as the natural products are sensitive to a range of reaction conditions commonly used in organic chemistry. For example, the lactone units of the rifamycins were prone to hydrolysis, their quinone moiety was redox sensitive, and the double bond of 35 was prone to isomerization at C_19_ to C_20_ (Fig. 1C). Those structural moieties required avoidance of transition metal catalyzed reactions (*e.g.* click chemistry) or strongly acidic protection group chemistry (*e.g.* TFA, HCl). As the ansamycin bridge motif has been described to be important for rifamycin function, modifications generally exhibited a negative effect on the antimicrobial efficacy.^34^ Prior medicinal chemistry efforts at the 3′/4′ position retained or increased the molecule's antibiotic activity and even yielded potent, clinically used antibiotics like rifampicin, or rifabutin (Table S1†).^35–38^ As the binding site of rifamycin is located within a shallow concave pocket formed by the RNAP β subunit, linkers at least 6 atoms long were employed to reduce negative steric effects perturbing target binding.^39^ We initially aimed to introduce a conjugation handle of the naphthyl core of 1 based on existing protocols using mono TBS protected 2-amino-resorcinol 72 to give 73 (Fig. 2A). This could be reacted further to intermediate 74 by attaching an amine of choice using manganese dioxide.^40^ However, the workup was found to be difficult, and the reaction generally led to low yields in our hands. Therefore, we switched to a procedure using 4-fluoro-modified 2-amino-resorcinol 61, enabling a more facile access to amine-modified, conjugatable rifamycins.^41,42^ Condensation of 61 to 1 under protective atmosphere, conversion to the imine (not shown) and subsequent re-oxidation to the quinone with oxygen and benzoquinone (BQ) or TEMPO yielded the fluororesorcinol-modified rifamycin S 2. Upon nucleophilic aromatic substitution with primary amines, the mono- and dicatechol conjugated rifamycins 3 and 4, respectively, were obtained. 3-Formyl rifamycin 27 was reacted with the primary amines 40 or 41, bearing a monocatechol unit with a disulfide linker or a covalent PEG linker, respectively, to give the imines 5a and 6a (Fig. 2B). A subsequent reduction with NaBH(OAc)_3_ led to the amines 5 and 6.

Antibiotic testing of rifamycin 1 and its derivatives 2–6 in an MIC assay under iron-depleted conditions showed that 2, 3, 5a and 6a retained activity against multidrug-resistant (MDR) *S. aureus* compared to the unmodified drug (see ESI for details on medium preparation and Fig. S24–S27 and Table S3†). In MDR Gram-negative bacteria, some conjugates exhibited a higher antibiotic efficacy than free, unmodified 1 or the fluorinated intermediate 2. Notably, dicatechol 3 exhibited an MIC of 0.5 μM in MDR *A. baumannii*, corresponding to a 4–16-fold increase in activity compared to 1 or 2 (Fig. S27 and Table S3†). In a recent study, the rifamycin derivative rifabutin was shown to exhibit a nanomolar MIC in carbapenem-resistant *A. baumannii* following uptake *via* the bacterium's siderophore receptor FhuE under iron-depleted growth conditions.^43,43,44^ However, the increase of activity upon conjugation observed here suggests that any inherent ansamycin activity can be further enhanced by siderophores. Nevertheless, the introduction of bi- and tetradentate chelators failed to achieve broad-spectrum antimicrobial activity. These observations are in accordance with literature findings that generally report a poor activity for most covalently coupled conjugates with warheads aiming at cytosolic targets.^17,45^ The findings imply that the passage of those compounds over the inner membrane needs to be considered in order to achieve a significant antimicrobial effect. Given the orthogonal properties of the two membranes, we hypothesized that the insufficient passage of those compounds over the inner prevented a stronger antimicrobial effect. Since these compounds passage successfully over the Gram-positive membrane, which is structurally comparable to the inner membrane of Gram-negative bacteria, conjugates may substantially benefit from an intracellular release mechanism for the antibiotic cargo to facilitate independent translocation over the inner membrane.

A second factor for the limited efficacy of 3–6 might be their reduced competitiveness towards natural triscatecholate siderophores such as enterobactin, which form more stable 1 : 1 iron complexes.^46^ We therefore aimed to couple a cleavable linker to hexadentate triscatechol and trishydroxamate siderophores which, due to their higher coordination number, form more stable iron complexes.^22,47^

### Cleavable, hexadentate siderophore ciprofloxacin conjugates serve to select optimal linkers

**Fig. 3:**
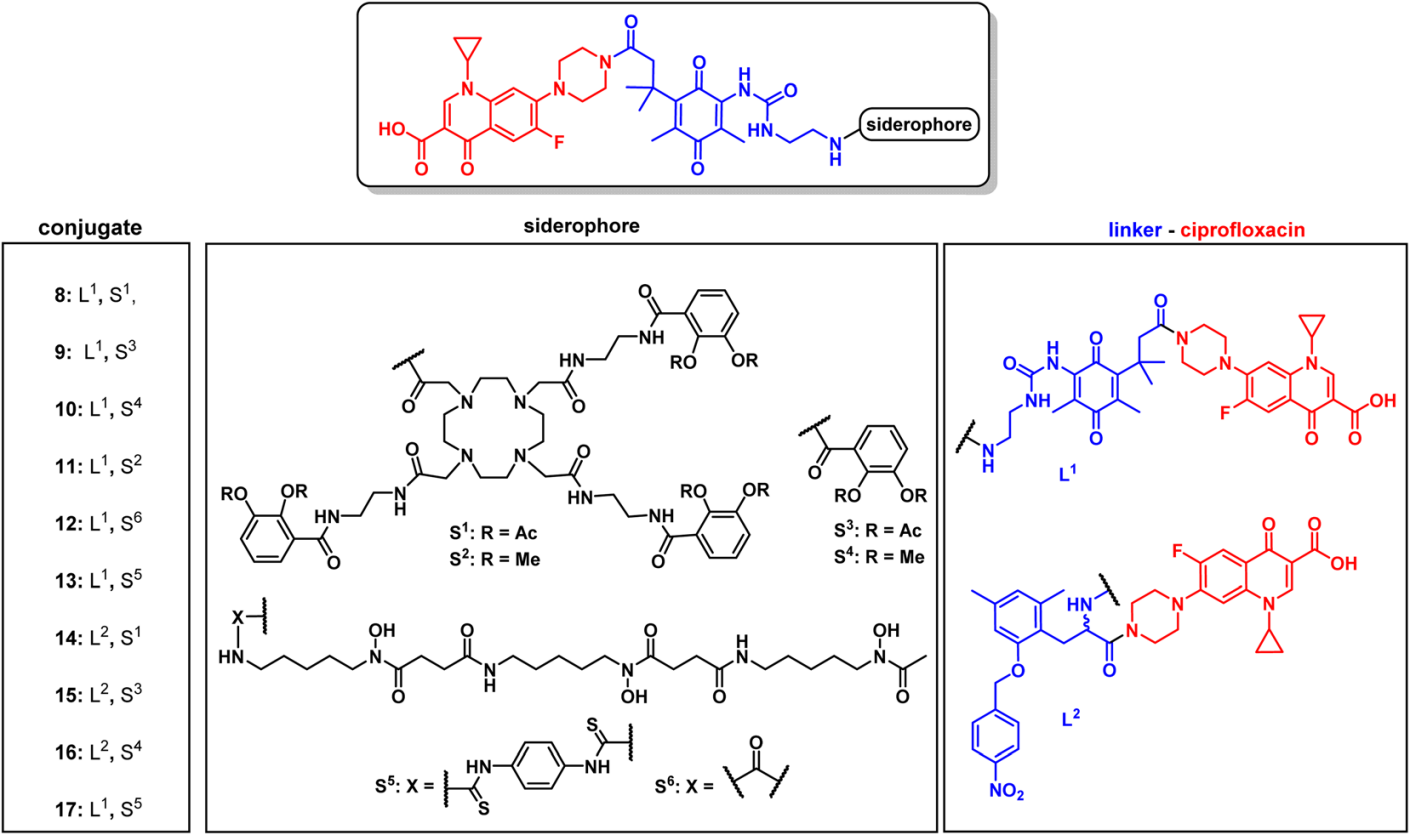
Structures of monocatechol, DOTAM-anchored triscatechol and DFO-TML ciprofloxacin conjugates 8–17. For information on the synthetic procedures and reagents consult the ESI, Fig. S3 to S5.†

For release within the pathogens, the trimethyl lock (TML) linker system was adapted, which was introduced by Ji and Miller to construct ciprofloxacin siderophore conjugates with a reduction triggered TML cleavable linker for intracellular drug release in bacteria.^20^ In order to optimize the chemistry and to identify beneficial siderophore-linker combinations, we used the antibiotic ciprofloxacin due to its availability, well-established chemistry and cytosolic mode of action, before coupling to the more complex RNAP-inhibiting natural products. For an efficient release, a quinone-based (L1 in Fig. 3) or a *para*-nitrobenzyl-based (L2 in Fig. 3) linker was coupled to ciprofloxacin and then attached to monocatechol or hexadentate siderophores. Starting from 2,6-dimethylbenzene-1,4-diol and methyl 3-methylbut-2-enoate, the amino benzoquinone 51 was prepared in 8% yield over six steps based on modified literature procedures (see ESI, Fig. S3 to S5†).^48^ Derivatization attempts with phosgene proved to be unsuccessful (side products, no product formation), but triphosgene in toluene at 80 °C was employed to introduce an *N*-Boc ethylendiamine linker and form the urea intermediate 52. Ester hydrolysis, amide coupling and Boc group cleavage yielded the key fragment 59a in 51% over three steps. This key intermediate was attached by mixed anhydride chemistry or thiourea formation to a monocatechol, a DOTAM-anchored triscatechol or a DFO siderophore to yield conjugates 8–13 (Fig. 3). Compound 12 is the reference cleavable conjugate reported by Ji and Miller.^20^

The second linker was synthesized starting from a commercial synthetic amino acid to yield the *para*-nitrobenzyl intermediate 54 in 83% yield over two steps (Fig. S5A†). Similarly, ester hydrolysis, EDCI-activated coupling of ciprofloxacin and Boc group cleavage led to the key intermediate 60 in 65% yield over three steps, which was conjugated to give the monocatechol, triscatechol and DFO siderophore conjugates 14–17 (Fig. 3B, S5B and C†). Catecholate siderophores with transient (acetylated), stable (methylated) or no protecting groups at the phenol moieties were used to examine conjugate activity. In accordance with earlier studies, acetyl-masked catecholates serve as prodrugs, by avoiding the inactivation of iron-chelating phenol functions by catechol-*O*-methyltransferases and are deacetylated at physiological pH over time.^22,26^ Siderophores with methylated phenols are unable to complex ferric iron and thus serve as a negative control.

The conjugates as well as the intermediates 52 and 59 were subjected to antimicrobial susceptibility testing under iron-depleted conditions in MDR *E. coli* to find the linker–siderophore combinations which retain the highest activity (Table 1 and Fig. S28†). Overall, the hexadentate iron binders 8, 12, 13 and 17 exhibited a higher retained activity than the monocatechol conjugates. The quinone-based TML L^1^ yielded lower MIC values than the *para*-nitrobenzyl TML analogue L^2^. The latter lacked two geminal methyl groups at the methylene unit that drive the lactonization and payload release, thus possibly accounting for the observed lower activity.

**Table 1:** MIC values^a^ for cleavable siderophore ciprofloxacin conjugates 8 to 17

Compound	MW	Siderophore	Linker	MIC in *E. coli*
8	1823	S^1^	L^1^	2
9	844	S^3^	L^1^	4
10	788	S^4^	L^1^	32
11	1655	S^2^	L^1^	>64
12	1151	S^6^	L^1^	≤1
13	1387	S^5^	L^1^	≤1
14	1830	S^1^	L^2^	32
15	877	S^3^	L^2^	>64
16	821	S^4^	L^2^	>64
17	1410	S^5^	L^2^	≤1
52	451	—	L^1^	>64
59	650	—	L^1^	2
Cefiderocol^b^	752	Catechol	Alkyl	0.01
Ciprofloxacin	331	—	—	0.24

a Values are given in [μM], MW = molecular weight [g mol^−1^].

b Payload is a cephalosporin.

The increased steric bulk of the triscatechol conjugates 8 and 14 also translated to a lower antibiotic activity than observed for the less strained DFO conjugates 12, 13 and 17 with their linear structure. In line with previous studies, the catechols were masked as acetylated prodrugs in order to avoid immediate deactivation of the iron chelating units by bacterial catechol-*O*-methyltransferases.^49^ An exchange of the acetyl groups of 9 (4 μM) with stable methyl groups as in 10 prohibited iron complexation and in turn active uptake, and it decreased activity eightfold. The molecular weight cut-off for unspecific uptake *via* bacterial porins ranges between 600–800 Da.^50,51^ In light of this, monocatechol 10, with methylated catechols unable to complex iron but a lower molecular weight of 788 Da, may still enter by passive diffusion, however with a lower efficiency than triscatecholate 9, which is reflected by their antimicrobial activity. The methylated triscatecholate conjugate 11, with a molecular weight of 1655 Da significantly above the cutoff for passive uptake, had no antimicrobial activity.^52^ This suggests a central role of the siderophore carrier for bacterial accumulation and antimicrobial efficacy. In summary, compounds with a quinone-based TML linker retained the highest activity, and thus the quinone based TML was selected for the conjugation of the RNAP-I payloads.

### Design and synthesis of covalent and cleavable RNAP-I siderophore conjugates

**Fig. 4:**
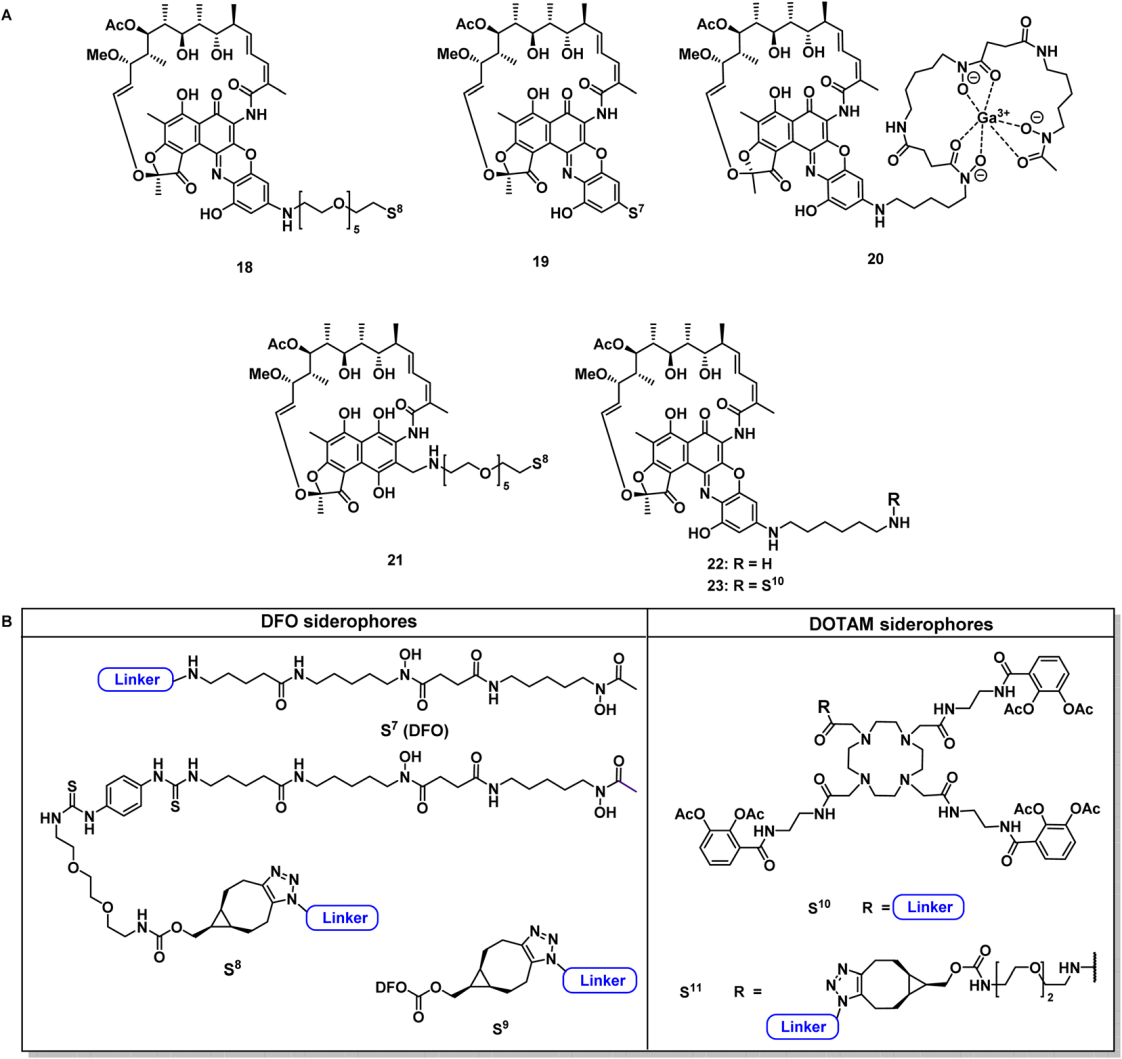
Structural overview of covalent conjugates 18–21 and 23 and control rifamycin derivative 22 (A); overview of employed siderophore vectors (B). For synthetic procedures and full structures see ESI, Fig. S3 to S18.†

**Fig. 5:**
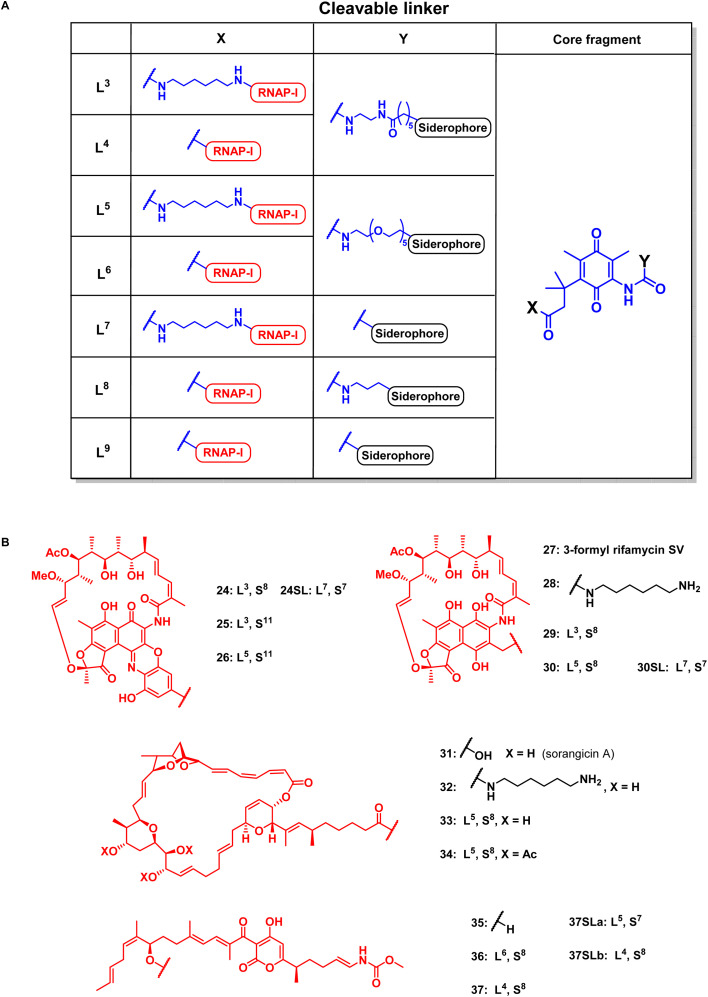
Structural overview of linker motifs (A) and cleavable conjugates 24–37 (B). Refer to Fig. 4 for siderophore S^*X*^ structures and the ESI† for synthetic procedures.

Next, reaction conditions were developed to synthesize covalent and cleavable RNAP-I siderophore conjugates. Because the first series of rifamycin conjugates (Fig. 2) had one or two bidentate iron binders, selected covalent conjugates were prepared as comparators, which were able to hexacoordinate iron. Rifamycins 1 and 27 were modified at the naphthyl core using the conditions established above. Conjugation *via* strain-promoted azide–alkyne cycloaddition (SPAAC) to the alkyne DFO 47 then furnished conjugates 18 and 21 in yields of 88% and 57%, respectively (Fig. 4). Direct aromatic nucleophilic substitution of 2 gave DFO rifamycin 19 (84% yield), which could be converted quantitatively to the gallium complex 20 under mild conditions. The 1,6-diaminohexane-modified rifamycin 22 could be attached to the triscatecholate siderophore 7 by mixed anhydride coupling and afforded conjugate 23 in 79% yield. For the synthesis of cleavable RNAP-I conjugates, an azido linker was incorporated at the TML instead of the *N*-Boc-ethylendiamine for the ciprofloxacin conjugates, as the natural products otherwise decomposed or isomerized under the strongly acidic reaction conditions otherwise required for protecting group cleavage. Ester hydrolysis followed by mixed anhydride activation at the TML allowed the attachment of the native or 1,6-diaminohexyl-modified payloads (Fig. 5 and S6–S16†). Finally, a SPAAC with the BCN-modified DFO 47 or the triscatecholate 45 yielded the TML-triggerable rifamycins 24, 29 and 30, or 25 and 26, respectively.

According to previous studies, replacing of the acid moiety in sorangicin A with amide derivatives mostly retained its antibiotic activity in *S. aureus* and *E. coli* (Table S2†).^53^ This encouraged us to conjugate sorangicin A by amide chemistry. Along those lines, SPAAC with the BCN-modified DFO siderophore 47 yielded the sorangicin A conjugates 33 and 34 (Fig. 5, S12 and S13†). Two corallopyronin conjugates, 36 and 37, were prepared by an esterification of the secondary alcohol of the natural product with cleavable TML-linked DFO moieties (Fig. 5, S14 and S15†).^47^ To assess the effect of a shorter distance between DFO chelator and TML, conjugates or linker fragments 73, 74, 24SL, 30SL and 37SLa/b were prepared. These compounds were obtained as follows: 22, 28 or 35 were reacted with 72 or with 58a in a mixed anhydride coupling with *iso*-butyl chloroformate or by amide coupling with EDCI/HOBt/DMAP. SPAAC of azide 74 with strained alkyne 47 then furnished conjugate 37SLb. These conjugates were also designed to reduce molecular weight and thus possibly compete better with the smaller, natural chelators of the bacterial pathogens.

### Covalent and cleavable RNAP-I conjugates show antimicrobial activity

All conjugates, reference natural products (1, 27, 31, 35) and modified payloads were subjected to antimicrobial efficiency testing under iron-depleted conditions against MDR pathogens from the ESKAPE panel and against two siderophore-deficient strains (Table 2, Fig. S29–S39 and Tables S4–S10†). While the control antibiotics exhibited activities in the expected ranges,^18,26,31^ neither the DOTAM-anchored siderophore 7, nor the bare TML linker 53 or the linker-payload intermediates without the siderophore carrier 65, 67, 69 and 70 were active (Tables S4–S10†). Interestingly, the covalent conjugate 19 and its gallium complex 20 retained activity against MDR *S. aureus.* Thus, the DFO conjugates could host a metal cation, *e.g.* for PET imaging purposes, as well as an antimicrobial payload, and still exert an antibiotic affect. The data are in line with recent studies on the *in vivo* imaging and treatment of microbes with DFO based probes,^54^ and they underline the potential of DFO conjugates to serve as theranostics in antimicrobial chemotherapy.^55,56^ However, an activity increase was not seen upon gallium(iii) incorporation, and solely the uncomplexed 19 showed a threefold lower MIC compared to free 1 in MDR *E. coli*. Possibly the direct attachment of DFO to the rifamycin and the steric bulk of the complex negatively affected target binding in the bacteria. This suggests that analogues with longer distances between the DFO unit and the antibiotic should be explored in subsequent optimization rounds.

**Table 2:** MIC values^a^ for 18–37 in MDR *E. coli* and *S. aureus*

Compound	*S. aureus*	*E. coli*
2	4	>32
18	32	13
19	1	8
20	1	>32
21	>32	4
22	4	>32
23	1	>32
24	32	1
24SL	>32	32
25	1	>32
26	2	>32
27, CHO-rifSV	≤0.5	16
28	>32	>32
29	>32	1
30	>32	2
30SL	>32	16
31, sorA	2	>32
32	>32	>32
33	32	16
34	22	>32
35*, corA	>32	>32
35, corA	2	>32
36	20	2
37	8	10
37SLa	>32	>32
37SLb	4	>32
Cefiderocol	>0.64	<1

a [μM] for test compounds and cefiderocol, [μg mL^−1^] for ciprofloxacin, amikacin, linezolid, DFO = desferrioxamine, rifS = rifamycin S, CHO-rifSV = 3-formyl rifamycin SV, sorA = sorangicin A, corA = corallopyronin A, GE = growth enhancing, 35* = corA re-isolated from preparation of 36/37.

**Fig. 6:**
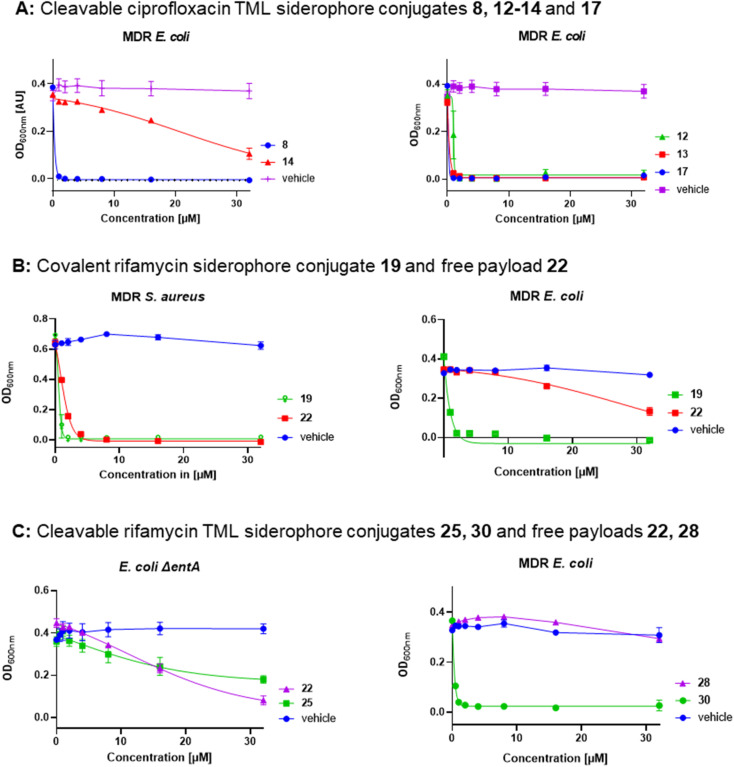
Dose–response curves of gyrase inhibitors and RNAP inhibitors in MDR and siderophore-deficient *E. coli* strains. (A) Conjugates 8, 14 (triscatecholate carrier) and 12, 13, 17 (DFO carrier), dose response of controls are deposited in Fig. S28,† (B) covalent DFO rifamycin S conjugate 19 and free rifamycin 22, (C) DOTAM (25) and DFO (30) TML rifamycin conjugates with free payloads 22 and 28, error bars ± SEM, *n* = 2–3, information on the strains and the protocol is deposited in the ESI.†

Covalent and cleavable conjugates with a more bulky triscatecholate carrier, namely 23, 25 and 26, were completely inactive in *E. coli* and *P. aeruginosa*. Their retained activity in *S. aureus* indicates an impaired uptake into Gram-negative bacteria and/or a slower, inefficient enzymatic payload release, as observed for the ciprofloxacin conjugates. The free natural products and their derivatives displayed better activities in *S. aureus* than the high molecular weight conjugates. Notably, the cleavable DFO–rifamycin conjugates 24, 29 and 30 exhibited MIC values around 1–2 μM in MDR *E. coli*, corresponding to a 12–32-fold improvement compared to 1 or 2 (Fig. S29†). In contrast, conjugates 21, 24, 29 and 30, based on the rifamycin 27, led to an increased growth of *A. baumannii* and *P. aeruginosa* in comparison to the solvent control (Fig. S32–S35†). This observation indicates that the conjugates shuttled iron into the bacteria, but exerted no antibiotic effect. With regard to the sorangicin conjugates, a basal activity could be detected for free sorA 31 in *S. aureus* and in siderophore-deficient mutants of *E. coli*, but not for the amide-modified 32. Conjugates 33 and 34 showed a weak retained activity in *S. aureus* (22–32 μM) and an MIC of 16 μM for 33 in MDR wildtype *E. coli* (Fig. S31 and S29†). The corallopyronin conjugates 36 and 37 exhibited potent to moderate activity against MDR *E. coli* and *P. aeruginosa* in the range of 2–32 μM. In line with other RNAP-I conjugates, the activity against *S. aureus* was weaker compared to free 35. Corallopyronins are known to be labile in solution due to an *E*/*Z* isomerization of the C_19_–C_20_ double bond, accelerated with increasing temperature or acidic conditions.^57^ We therefore expected an isomerization during the synthesis and isolation process, which not only applies to corallopyronin A (CorA) 35/corallopyronin A′ (CorA′) 35′ but also to their conjugates 36 and 37. The isomerization might be of importance here, as 35′ showed a decreased biological activity by a factor of 5–10.^33^ The ratio of the reisolated starting material was determined to be 75% 35 and 25% 35′ in MeCN-*d*_3_*via* NMR analysis (Fig. S19 and S20†). The isomeric ratio of conjugate 37 could not be clearly determined due to signal overlap in the respective region (Fig. S21†). Due to the isomerization problems, which could not be controlled under the needed synthetic conditions, we decided to not pursue corallopyronin conjugates further, in spite of their partly promising activity (Table 2). Overall, several RNAP-I conjugates from three structural classes, represented by 29, 30, 33 and 36, illustrate the potential of siderophore Trojan Horses to enhance the activity of complex natural product antibiotics with cytosolic targets by harnessing TBDTs. This is illustrated by dose–response curves for the ciprofloxacin conjugates 8, 12–14 and 17, for the rifamycin conjugates 19, 25 and 30 as well as for the unconjugated rifamycins 22 and 28 (Fig. 6).

Generally, a shorter distance between siderophore carrier and the cleavable TML moiety led to slightly decreased antimicrobial activities than for conjugates with a longer distance. This is especially evident for conjugates 24*vs.*24SL (MIC 4–8 fold ↑) 30*vs.*30SL (MIC 8–16 fold ↑) and 36/37*vs.*37SLa/b (MIC 2–32 fold ↑) in MDR *E. coli* and *P. aeruginosa* (Tables S4 and S7 *vs.* S9 and S11†). We speculate that this shorter distance might lead to steric clashes within the quinone oxidoreductase for more bulky rifamycin or corallopyronin payloads, compared to the smaller ciprofloxacin, and perturb the enzymatic release, thereby leading to a diminished antimicrobial activity.

### The siderophore transport machinery is essential for conjugate accumulation and antimicrobial activity

A subset of ciprofloxacin (8, 11, 12, 13), rifamycin (19, 24SL, 30SL) and corallopyronin A (37SLa) siderophore conjugates was further evaluated in MIC assays with *E. coli* mutants that were deficient in either enterobactin synthesis (*ΔentA*), or catecholate receptors (*ΔfepA*, *Δfiu*), hydroxamate siderophore receptors (*ΔfhuA*) or key proteins responsible for siderophore internalization or energy provision (*ΔfepB*, *ΔtonB*).^23^ The ciprofloxacin–TML–triscatecholate conjugate 11 with methylated catechols served as a negative control, because no siderophore uptake could occur. As expected, it did not show any antibiotic activity (Fig. S42, and Table S14†). In contrast, ciprofloxacin–TML–triscatecholate conjugate 8, with acetylated catechols, showed a 2 μM MIC in the *E. coli ΔentAΔfhuA*, which doubled (4 μM) upon knockout of either the periplasmic catecholate binding protein FepB or the catecholate TBDTs Fiu or FepA. This indicates a role of these proteins in conjugate internalization. The cleavable DFO ciprofloxacin conjugates 12 and 13 showed an unperturbed MIC of ≤1 μM in all tested catecholate TBDT KO mutants and in the Δ*fepB* mutant, while the *ΔentAΔfhuA* mutant displayed a slight increase of the MIC to 2 μM (Fig. S42E†). Also for the RNAP-I DFO conjugates 19, 24SL, 30SL or 37Sla, no effect was observed upon loss of *fepA* or any catecholate TBDT, but the loss of the FhuA TBDT (*ΔentAΔfhuA*) lead to a 2–4-fold increased MIC. This finding suggests a role of the FhuA receptor, aside the main ferrioxamine B transporter FhuE, in conjugate uptake (Fig. S41†).^58^ A loss of the inner membrane protein TonB, involved in energy transduction on the TBDTs by the proton-motive force however completely abolished antibiotic activity of all conjugates and increased the MIC of the reference siderophore antibiotic cefiderocol 64-fold to 64 μM (Table S13 and Fig. S42†). This underlines the relevance of the siderophore uptake machinery for conjugate accumulation. Importantly, the MIC of ciprofloxacin remained unaffected (≤1 μM) by any of the knockout mutants, as this compound is known to diffuse through membranes in its neutral form or enter through porins (Fig. S42†).^59^

### Bacterial quinone oxidoreductases enable *in vitro* linker cleavage and drug release

Next, we investigated whether bacterial enzymes could activate the linker and release the payload. For this purpose, two bacterial enzymes, a quinone oxidoreductase QOR2 from *E. coli* and a diaphorase from *C. kluyveri*, were incubated with the ciprofloxacin conjugate 12 and the rifamycin conjugate 24SL in phosphate buffer (pH 7.4) at 30 °C.^23^ The ciprofloxacin conjugate 12 was chosen because of its small payload size. Conjugate 24SL showed favourable chromatographic properties that facilitated its sensitive detection by analytical HPLC, in contrast to other conjugates. It also served to demonstrate that bigger, more complex natural products can be released from their respective conjugates by bacterial enzymes.^20^ Analytical RP-HPLC traces were recorded at discrete times and compared to a reference injection of free ciprofloxacin or free rifamycin payload 22. Both enzymes led to an almost complete cleavage of 12 as well as of 24SL within 18–24 hours and a concomitant release of ciprofloxacin or the rifamycin payload 22, respectively (Fig. 6A, S22 and S23†). This demonstrates that the enzymatic activation was not impaired by the functional moieties attached to the TML.

### LC-MS/MS uptake measurements of 24SL and 36 in *E. coli* show bacterial accumulation and intracellular payload release

**Fig. 7:**
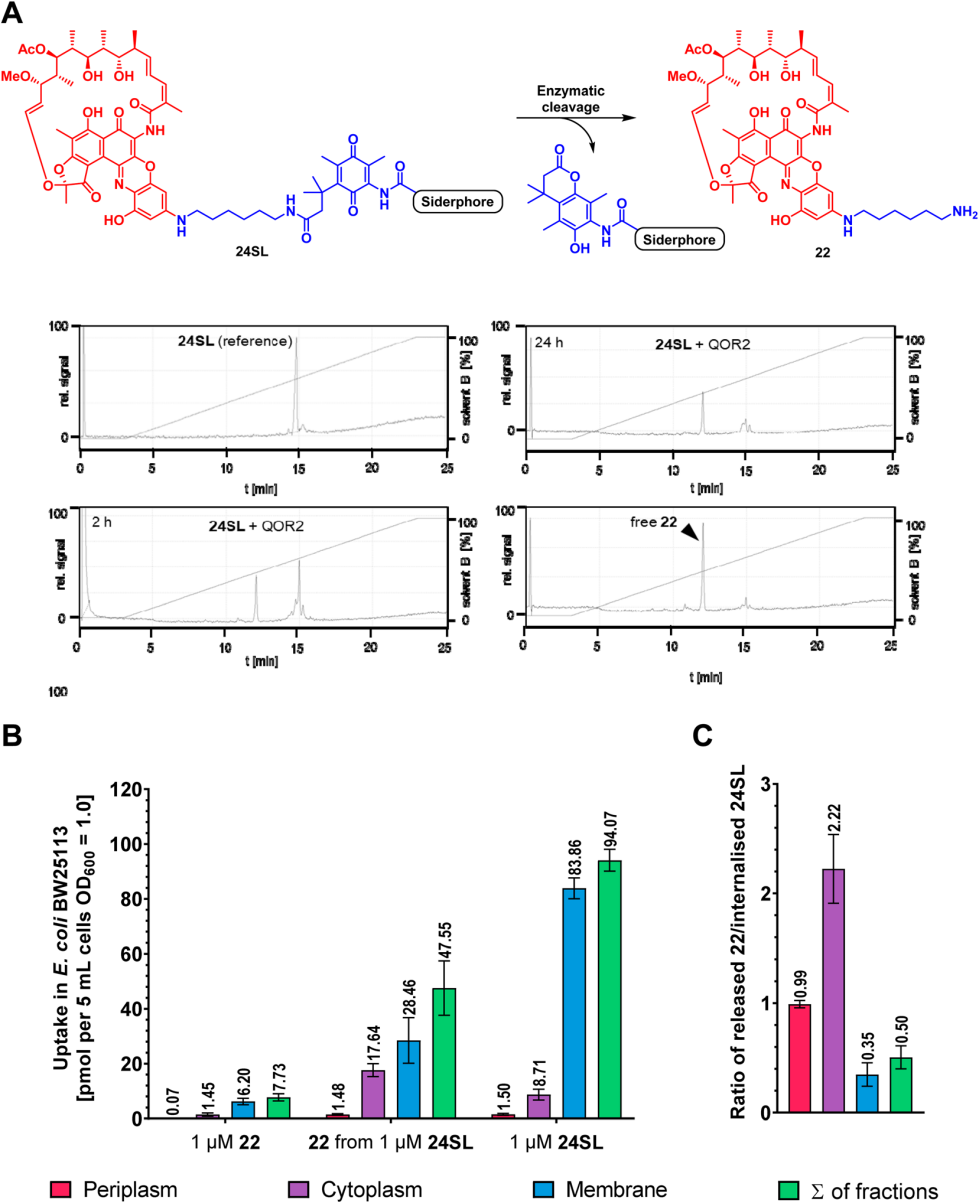
Antibiotic payload release in Gram-negative bacteria. (A) *In vitro* enzymatic activation of 24SL to give 22 by the *E. coli* enzyme QOR2. Relative UV signals (220 nm) of analytical HPLC runs after 2 h and 24 h of incubation and controls, normalized to the highest peak, are displayed (for details, see ESI†). (B) Accumulation of 1 μM 22 and 1 μM 24SL after incubation for 10 min in *E. coli* BW25113; left: quantification of 22 accumulated upon incubation with 1 μM 22; middle: quantification of 22 accumulated upon incubation with 1 μM 24SL; right: quantification of 24SL accumulated upon incubation with 1 μM 24SL. Error bars = ± SEM, two biological replicates with two technical replicates. (C) Ratio of the amounts of 22 released from 24SL and internalized 24SL detected in the fractions. Single replicate values were used to calculate the ratios, then all ratios were averaged across replicates in 7C.

To investigate whether the payload is also released in living bacteria, the uptake of 22, 24SL and the intracellular release of 22 from 24SL was studied in *E. coli* at a concentration of 1 μM (Fig. 7B). To this end, label-free detection and quantification of internalized compound was performed by LC-MS/MS analysis using compound-specific multiple reaction monitoring (MRM) as previously described,^60^ albeit with slight modifications: to ensure comparable assay conditions to the MIC experiments, the bacteria were first incubated one night in iron-depleted, cation-adjusted medium and then starved a second night in LMR, followed by growth up to OD_600_ = 0.5 in LMR (see ESI†). Cultures of approx. 3.9 × 10^9^ bacteria were incubated with compounds in LMR at the indicated assay concentrations and then subjected to a fractionation procedure to determine the amount of internalized compound in the periplasm, cytoplasm, and membrane fractions. Importantly, the amount of free 22 was lower starting from 22 compared to the amount of 22 released from 24SL, in good agreement with the MIC values (MIC of 22 = >64 μM, MIC of 24SL = 8–16 μM; Table S9†). The majority of 24SL was found in the membrane fraction. The ratio of free 22 to 24SL was higher than one (*i.e.* 2.22) in the cytoplasm fraction, whereas it was smaller in other fractions (0.99 in the periplasm and only 0.35 in the membranes; Fig. 7C). The experiments clearly show that 22 could indeed be released from 24SL inside the cell, and that siderophore conjugation enhanced the cytosolic concentration of the antibiotic 22.

Compound uptake was further determined for 35 and 36 in MDR *E. coli* DSM1116. Using the transitions of 35, it could be shown that negligible amounts of free CorA 35 accumulated in the bacteria. Upon incubation with 36, an approx. 300-fold increase in the internalized amount of 35 and its isomers formed after release was observed (Fig. S45†).

**Fig. 8:**
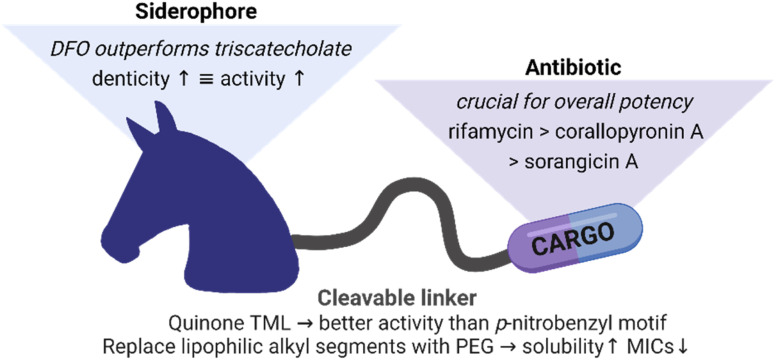
Summary of structure–activity relationships for siderophore conjugates.

Overall, we could extend applicability of Gram-positive only RNAP-Is to some Gram-negative bacteria by conjugation to siderophore carriers. The relationships between the conjugates structures and their activities are summarized in Fig. 8. Conjugation to ciprofloxacin generally led to a higher potency than to RNAP-I. The employment of a quinone-based TML as well as the conjugation to an elongated, less bulky DFO siderophore generally led to lower MIC values than the *p*-nitrobenzyl TML analogue or triscatecholate siderophore, respectively. Insertion of PEG linkers instead of more lipophilic alkyl linkers led to higher solubility and also better antimicrobial activity of the lipophilic RNAP-Is. Microbial activity testing in KO mutants revealed a moderate increase in MICs upon loss of single TBDTs. In contrast, an *E. coli ΔtonB* mutant, unable to internalize siderophores *via* TBDTs due to lack of the proton-motive force *via* the TonB protein, led to inactivity for all conjugates. This underlines the central role of the siderophore carrier moiety in conjugate uptake. The microbiological findings were supported by mechanistic studies showing enzymatic cleavage of the conjugate, intracellular conjugate accumulation and release of the payload at its cytosolic target.

## Conclusion

In this study, we successfully enabled antibiotic activity of complex natural RNAP inhibitors against Gram-positive bacteria, namely rifamycin S (V), sorangicin A and corallopyronin A, in Gram-negative bacteria by conjugation to siderophores. Several mono-, di- and triscatecholate as well as hydroxamate units were coupled covalently to rifamycin antibiotics. However, just a serendipitous improvement in antibiotic activity was observed for single compounds. This finding is in agreement with literature that reports a reduced activity for most covalent siderophore conjugates attached to payloads with cytoplasmic targets.^61,62^ A systematic exploration of two cleavable linker systems with the payload ciprofloxacin identified the quinone TML as the superior candidate in terms of antibiotic activity, which was subsequently used to couple a series of DOTAM-based catechol or hydroxamate carriers to RNAP-I. While the unconjugated linker fragments remained inactive, siderophore conjugation enabled antimicrobial potency in Gram-negative pathogens. However, further optimization is needed to achieve reliable broad-spectrum activity. RNAP-I conjugates reach low micromolar MICs, but not the exquisite, low nanomolar potency found for the free drugs against Gram-positive organisms such as *S. aureus*. This implies that further efforts are required to provide next generation conjugates combining improved outer membrane transport, efficient enzymatic release and potent antibiotic activity. In conclusion, the study provides a proof of principle how siderophore-mediated active import and enzymatic drug release can expand the antibiotic spectrum of natural RNAP inhibitors to Gram-negative bacteria.^22^

## Data availability

All the data supporting this article have been included in the main text and the ESI.†

## Author contributions

C. P. conceived the idea, designed and synthetized siderophores, conjugates and intermediates, performed characterization by NMR and MS, performed the enzymatic cleavage experiments, MIC assays, managed the project, wrote and corrected the manuscript. A. C. V. and H. L. S. F. designed and conducted the LC-MS/MS uptake experiments, wrote and corrected the manuscript. K. H. conducted 1D and 2D NMR measurements, wrote and corrected the manuscript. B. K. conducted MIC assays, M. S. provided corA and sorA and wrote and corrected the manuscript. M. B. conceptualized the study, secured the funding, wrote and corrected the manuscript.

## Conflicts of interest

There are no conflicts to declare.

## Supplementary Material

SC-014-D2SC06850H-s001
