# Supplementary material for: Siderophore conjugation with cleavable linkers boosts the potency of RNA polymerase inhibitors against multidrug-resistant E. coli
Source: Chem Sci. 2023 Apr 26;14(20):5490–502. doi: 10.1039/d2sc06850h (PMC10208051; doi:10.1039/d2sc06850h)
Supplement: SC-014-D2SC06850H-s001 [file SC-014-D2SC06850H-s001.pdf]

Electronic Supplementary Material (ESI) for Chemical Science.
This journal is © The Royal Society of Chemistry 2023

SUPPORTING INFORMATION

Siderophore conjugation with cleavable linkers boosts the potency of RNA polymerase inhibitors against multi-drug resistant *E. coli*

Carsten Peukert,[a,§] Anna C. Vetter,[a,§] Hazel L.S. Fuchs,[a, §] Kirsten Harmrolfs,[a] Bianka Karge,[a] Marc Stadler[b,c,d] and Mark Brönstrup[a, c, e]

Affiliations

a Department of Chemical Biology, Helmholtz Centre for Infection Research, Inhoffenstraße 7, 38124 Braunschweig

b Department of Microbial Drugs, Helmholtz Centre for Infection Research, Inhoffenstraße 7, 38124 Braunschweig

c German Center for Infection Research (DZIF), Site Hannover-Braunschweig, Inhoffenstraße 7, 38124 Braunschweig, Germany

d Institute of Microbiology, Technische Universität Braunschweig, Spielmannstraße 7, 38106 Braunschweig, Germany

e Institute for Organic Chemistry (IOC), Leibniz Universität Hannover, Schneiderberg 1B, 30167 Hannover, Germany

* corresponding author: Mark.Broenstrup@helmholtz-hzi.de

Table of Contents

Chemical methods 3
General chemical remarks 3
Chemistry figures and tables 5
Synthesis procedures 22
Siderophore synthesis 22
Cleavable linker synthesis 32
Gyrase inhibitor constructs 41
RNAP inhibitor (RNAP-I) constructs 55
Rifamycin S intermediates 55
Sorangicin A intermediates 66
Corallopyronin A intermediates 71
Mono and dicatechol rifamycin conjugates 75
DOTAM and DFO RNAP-I conjugates 80
Biological methods 100
Biology figures and tables 100
Enzymatic quinone TML activation 130
MIC assay in iron-depleted medium 131
Measurement of Uptake of siderophore conjugates and payloads into Gram-negative bacteria by LC-MS/MS 135
NMR spectra 141
References 225

Chemical methods

General chemical remarks

All reactions that required anhydrous conditions were performed under an argon atmosphere and with dry, commercial solvents. All reactions were carried out at room temperature (23-25 °C) unless stated otherwise. All general reagents, including salts and solvents, were purchased from Sigma-Aldrich, Acros Organics and employed without purification in the respective synthetic procedures. Chemicals and solvents were either p. a. grade or purified by standard techniques. For work up procedures and purifications, HPLC grade or p. a. grade were employed. Glassware was dried at 120 °C in an oven for minimum 24 h prior to being used for synthesis. Indicated yields are calculated based on substance purity (≥95%) analyzed by NMR spectroscopy and liquid chromatography mass spectrometry (LCMS).

Thin-layer chromatography (TLC)

Reaction progress was controlled by thin layer chromatography (TLC) or Liquid Chromatography-coupled Mass Spectrometry (LCMS). TLC silica gel plates were Merck® 60 F254 and compounds were visualized by irradiation with UV light.

Column chromatography

Preparative normal phase purifications were performed with silica gel Merck® 60 (particle size 0.040-0.063 mm), eluent given in parentheses.

Reverse-phase HPLC (RP-HPLC)

RP-HPLC was performed on a Dionex Ultimate system from Thermo Fisher Scientific® with the HPLC columns indicated below. The eluent is specified in parentheses for the respective synthetic procedure. Two columns (both C18, 250x4.6mm) were used.

- Luna C18, 5 µm, 100 Å, 00G-4252-PO-AX
- Gemini C18, 10 µm, 110 Å, 00G-4436-PO

Characterization of synthetic compounds

All final compounds were characterized by ^{1}H-, ^{13}C-NMR spectra and mass spectrometry and the spectra are added in the appendix.

NMR spectroscopy

NMR spectra were recorded on a 500 MHz Avance III (UltraShield) spectrometer equipped with a 5mm Nitrogen cooled Prodigy CryoProbe (BBO) or on a 700 MHz Avance III HD (Ascend) spectrometer equipped with a 5mm Helium cooled CryoProbe (TCI) by Bruker BioSpin GmbH, at 298 K. Chemical shift values δ of ^{1}H- and ^{13}C-NMR spectra are reported in ppm relative to the residual solvent signal, given as an internal standard and referenced to reported standard values. [1]

Mass spectrometry

High-resolution mass spectrometry (HRMS) was performed via a Dionex Ultimate 3000 HPLC system (Thermo Fisher Scientific, Dreieich, Germany) equipped with a DAD detector and a QTOF mass detector with electrospray ionization (ESI) (Bruker maxis HD, Bremen, Germany). Samples were directly injected via an Ultimate 3000RS autosampler (Thermo Fisher Scientific, Dreieich, Germany). The mass-to-charge ratio m/z is indicated.

Origin of payloads

Sorangicin A was re-purified from stock material available in the compound library of the HZI that was left from previous work at the GBF and had been produced following the paper by Irschik et al.[2] Corallopyronin A was likewise taken from the stock of the HZI compound library and had been produced as previously described by Schiefer et al.[3] Rifamycin S was purchased from TCI Germany GmbH (product #: R0200) and 3-formyl rifamycin SV was purchased from abcam (ab143401). Ciprofloxacin was purchased from SigmaAldrich (product #: 17850-5G-F).

Chemistry figures and tables

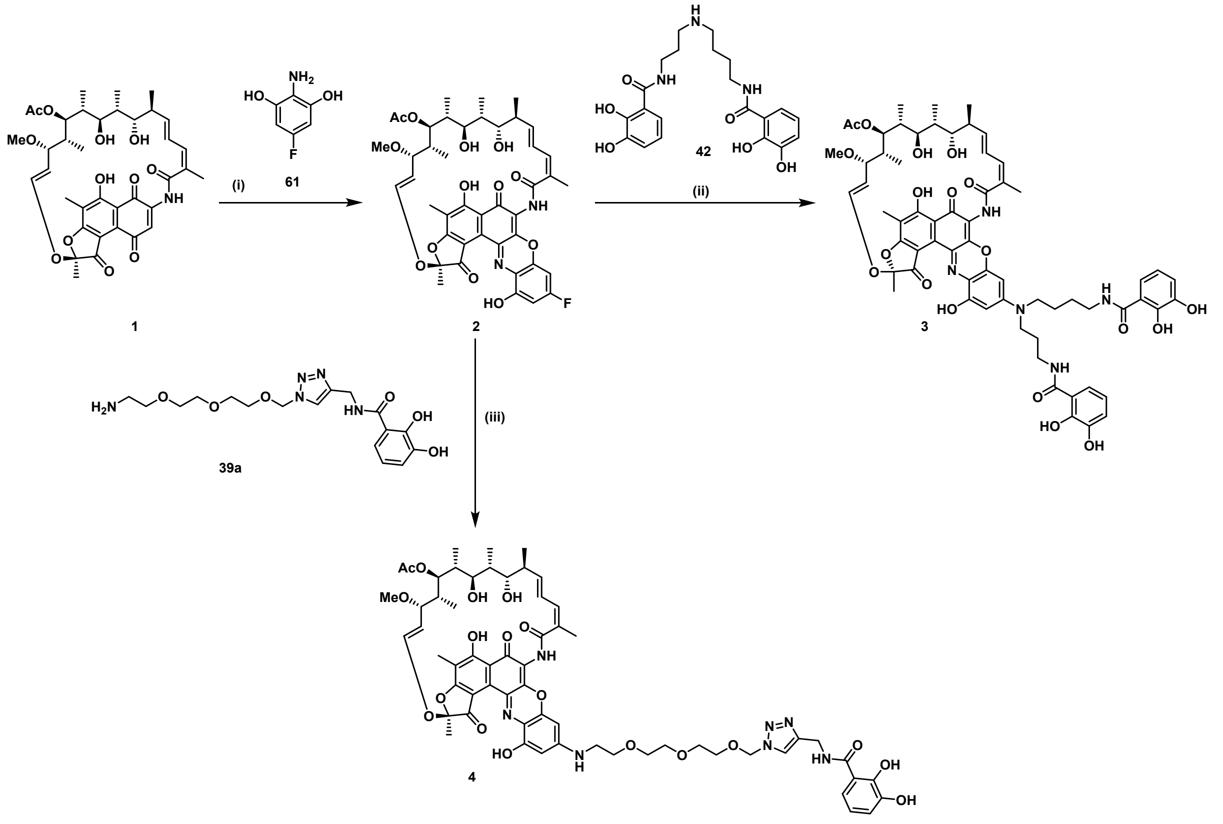

Figure S1. Synthesis of mono- and dicatechol rifamycin S derivatives **2-4**. (i) **61**, benzoquinone or TEMPO, oxygen gas, iPrOAc, 23 °C, 48 h, yield for benzoquinone/O_2: 21% and TEMPO/O_2: 50%, (ii) **42**, THF, DMSO, pyridine, DIPEA, 25-60 °C, 55%. (iii) **39a**, DIPEA, DMSO, THF, 25-45 °C, 30% over two steps.

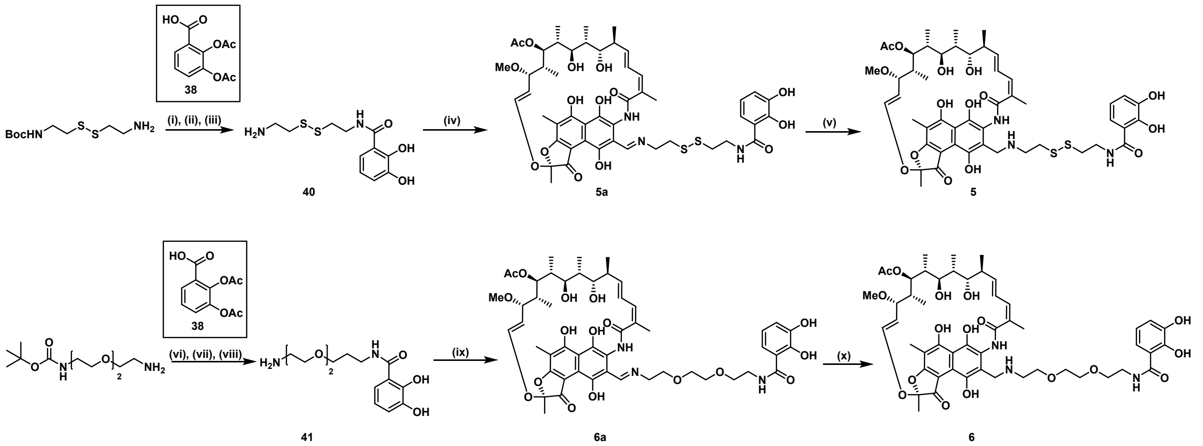

Figure S2. Synthesis of 3-formyl rifamycin SV monocatechol derivatives **5**-**6**. (i) **38**, *iso*-butylchloroformate, NMM, THF, 0-23 °C, (ii) TFA, AcOH, DCM, 0-23 °C, (iii) 20% DIPEA in anhydrous MeOH, 87% over three steps, (iv) 3-formyl rifamycin SV **27**, TEA, THF, 0-23 °C, 1 h, 90% crude, (v) $NaBH(OAc)_3$, 1h, 0-23 °C, 57% (vi) **38**, *iso*-butylchloroformate, NMM, THF, 0-23 °C, (vii) TFA, AcOH, DCM, 0-23 °C, (viii) 20% DIPEA in anhydrous MeOH, 93% over three steps, (ix) 3-formyl rifamycin SV **27**, TEA, THF, 0-23 °C, 1 h, 98% crude, (x) $NaBH(OAc)_3$, 1h, 0-23 °C, 79%.

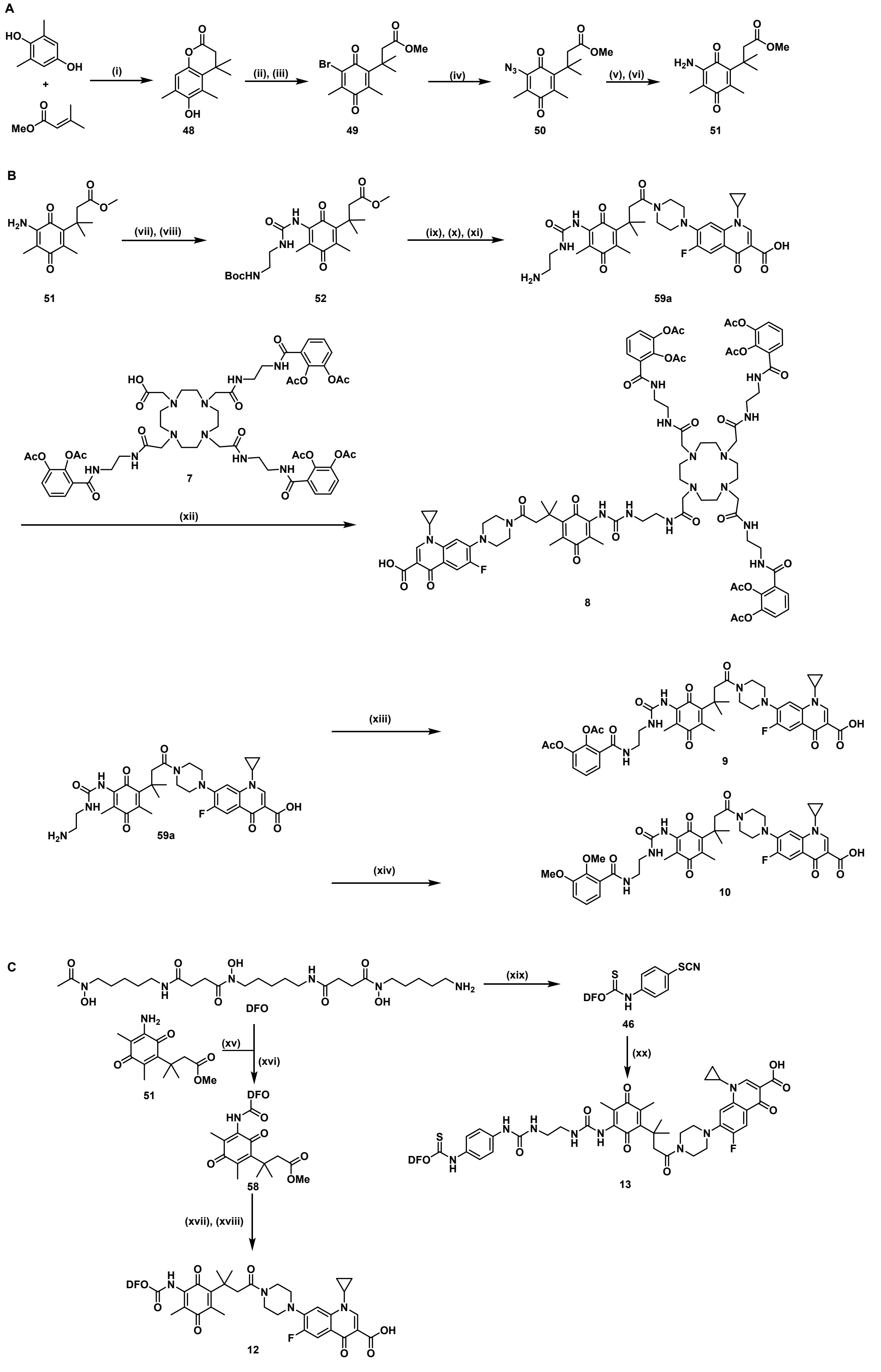
A
(i)
(ii), (iii)
(iv)
(v), (vi)
48
49
50
51
B
(vii), (viii)
(ix), (x), (xi)
51
52
59a
7
(xii)
8
(xiii)
(xiv)
9
10
59a
C
DFO
(xix)
46
(xv)
(xvi)
51
58
(xx)
13
(xvii), (xviii)
12

Figure S3. Synthesis of ciprofloxacin quinone trimethyl lock siderophore conjugates **8**-**13**. (A) Trimethyl lock synthesis: (i) $MeSO_3H$, 70 °C, 2 h, 91%, (ii) Br_2, AcOH, 23 °C, 56%, (iii) $SOCl_2$, MeOH, reflux, 50%, (iv) NaN_3, MeOH, H_2O, 25 °C, quant., (v) PPh_3, DCM, 23 °C, (vi) AcOH, THF, H_2O, 32% over two steps. (B) Catechol and DOTAM conjugates: (vii) triphosgene, anhydrous toluene, 23 - 80 °C, 4h, (viii) then *N*-Boc ethylene diamine, TEA, DCM, 0 - 23 °C, 59% over two steps, (ix) 1N NaOH, MeOH/H_2O, (x) ciprofloxacin, EDCI*HCl, HOBt, DIPEA, DCM, DMF, 0 - 23 °C, overnight, (xi) 25% TFA in anhydrous DCM, 0 - 23 °C, 2 h, 51% over three steps, (xii) *iso*-butylchloroformate, NMM, THF, 0-23 °C, 79%, (xiii) **38**, *iso*-butylchloroformate, NMM, THF, 0-23 °C, 83%, (xiv) 2,3-dimethoxybenzoic acid, *iso*-butylchloroformate, NMM, THF, 0-23 °C, 89%, (C) DFO conjugates: (xv) **51**, triphosgene, anhydrous toluene, 80 °C, overnight, then (xvi) DFO, DMF, TEA, 1 h at 0 °C, then 1 h at 25 °C, 18% over two steps, (xvii) 1N KOH in H_2O, MeOH, 25 °C, 3h, (xviii) ciprofloxacin, EDCI*HCl, HOBt, DIPEA, DCM, DMF, 0 - 23 °C, overnight, 35% over two steps, (xix) 1,4-dithiocyanatobenzene, 2-propanol/milliQ H_2O (10:1), chloroform, TEA, 0 - 24 °C, DCM wash, 77%, (xx) **46**, DMF, TEA, 24 °C, 73%.

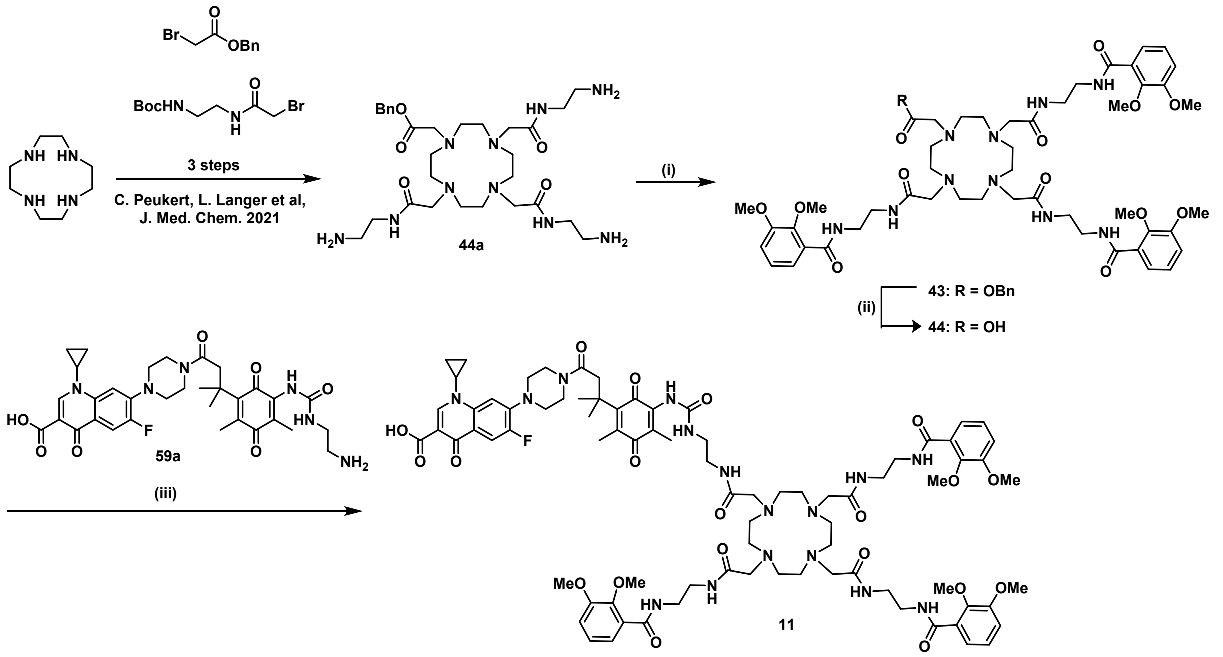

Figure S4. Synthesis of ciprofloxacin trimethyl lock DOTAM conjugate **11** with methylated catechols. Bromides synthetized as described in C. Peukert, L. Langer et al,[4] (i) 2,3-dimethoxybenzoic acid, HATU, DIPEA, DCM, DMF, 25 °C, overnight, 73% over three steps, (ii) H_2, Pd/C, MeOH, 25 °C, quant., (iii) *iso*-butylchloroformate, NMM, THF, 0-23 °C, 71%.

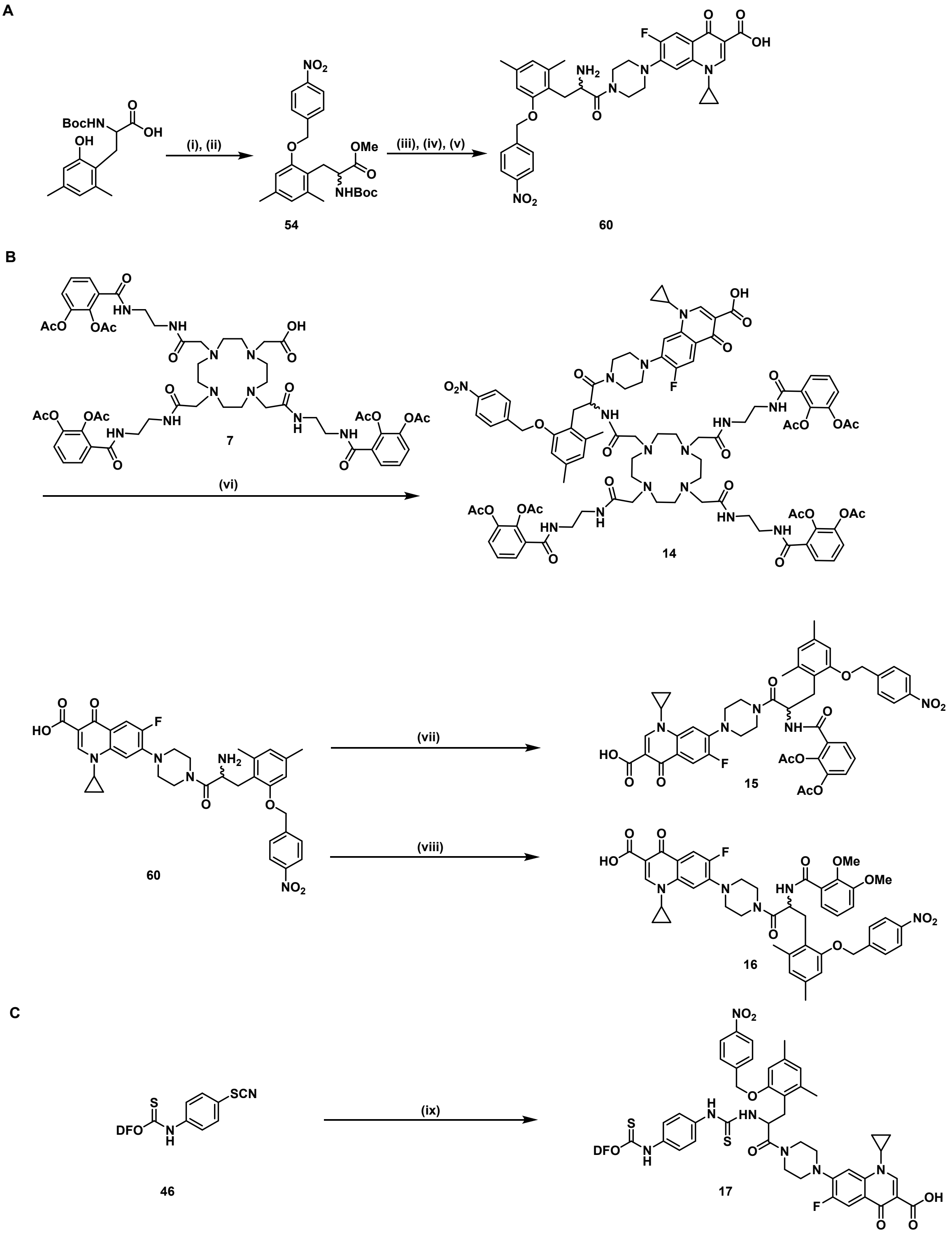

Figure S5. Synthesis of ciprofloxacin trimethyl lock analogue siderophore conjugates **14-16**. (A) Linker synthesis: (i) dimethoxypropane, MeOH, 20% HCl, 23 °C, 2 h, (ii) *para*-nitrobenzyl bromide, potassium carbonate, ACN, 23 °C, 83% over two steps, (iii) 1N NaOH in H_2O, MeOH, overnight, (iv) ciprofloxacin, EDCI*HCl, HOBt, DIPEA, DCM, DMF, 0 - 23 °C, (v) 25% TFA in anhydrous DCM, 0-22 °C, 2 h, 65% over three steps. (B) Catechol and DOTAM conjugates: (vi) *iso*-butylchloroformate, NMM, THF, 0 - 23 °C, 87%, (vii) **38**, *iso*-butylchloroformate, NMM, THF, 0-23 °C, 69%, (viii) 2,3-dimethoxybenzoic acid, *iso*-butylchloroformate, NMM, THF, 0-23 °C, 81%, (C) DFO conjugates: (ix) **60**, TEA, DMF, 73%.

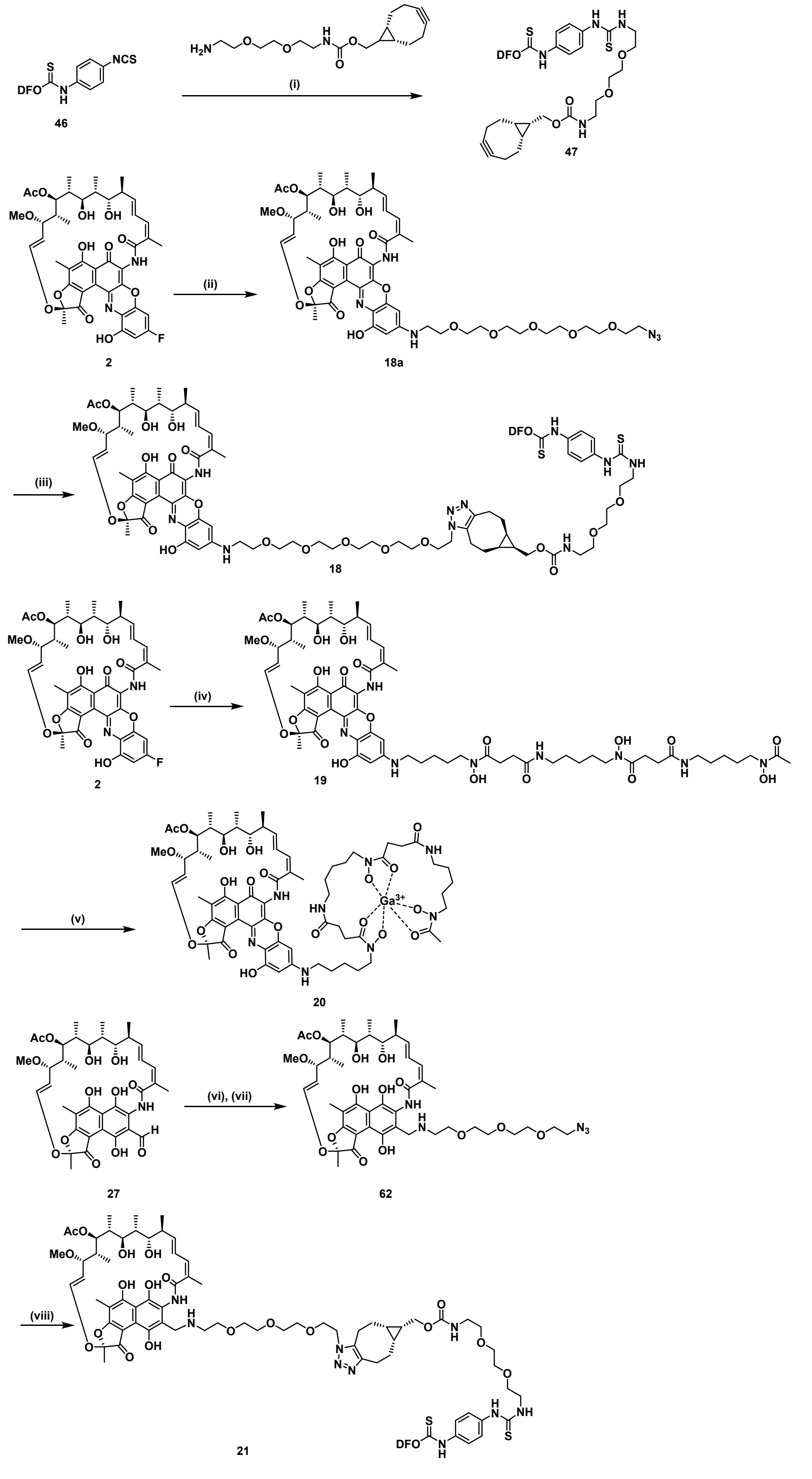

Figure S6. Synthesis of covalent DFO rifamycin conjugates **18**-**21**. (i) DMSO, TEA, 24 °C, 18 h, 51%, (ii) 17-azido-3,6,9,12,15-pentaoxaheptadecan-1-amine, TEA, THF, 24 °C, 48 h, (iii) **47**, ACN, H_2O (1:1), 24 °C, overnight, 88% over two steps, (iv) DFO, TEA, DMF, THF, 45 °C, overnight, 84%, (v) $GaCl_3$, NaOAc pH 4.5, overnight, 23 °C, quant., (vi) 2-(2-(2-(2-azidoethoxy)ethoxy)ethoxy)ethan-1-amine, TEA,

THF,1 h, 23 °C, (vii) $NaBH(OAc)_3$, THF, 0 - 23 °C, 98%, (vii) **47**, ACN, H_2O (1:1), 24 °C, overnight, 57%.

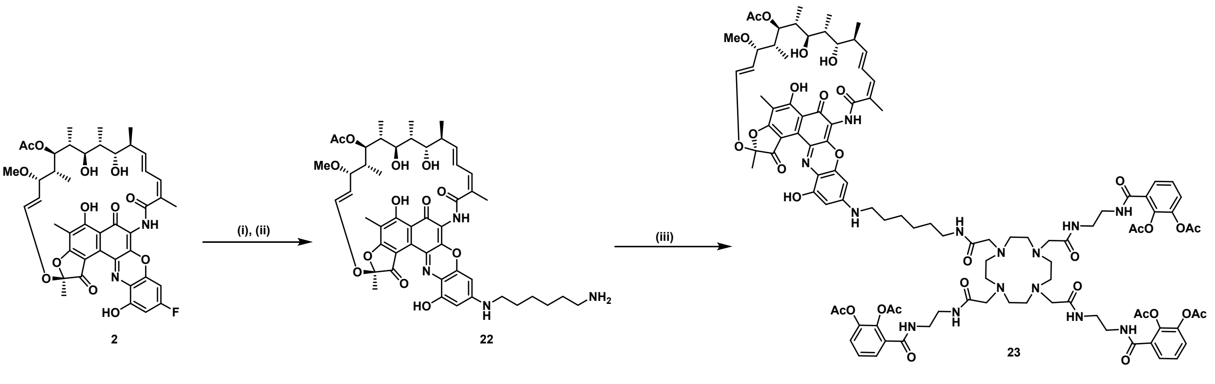

Figure S7. Synthesis of covalent rifamycin DOTAM conjugate **23**. (i) *N*-Fmoc 1,6-diaminohexane, TEA, THF, overnight, 23 °C, 92%, (ii) diethylamine, ACN, 23 °C, 2 h, 92%, (iii) **7**, *iso*-butylchloroformate, NMM, THF, 0-23 °C, 79%.

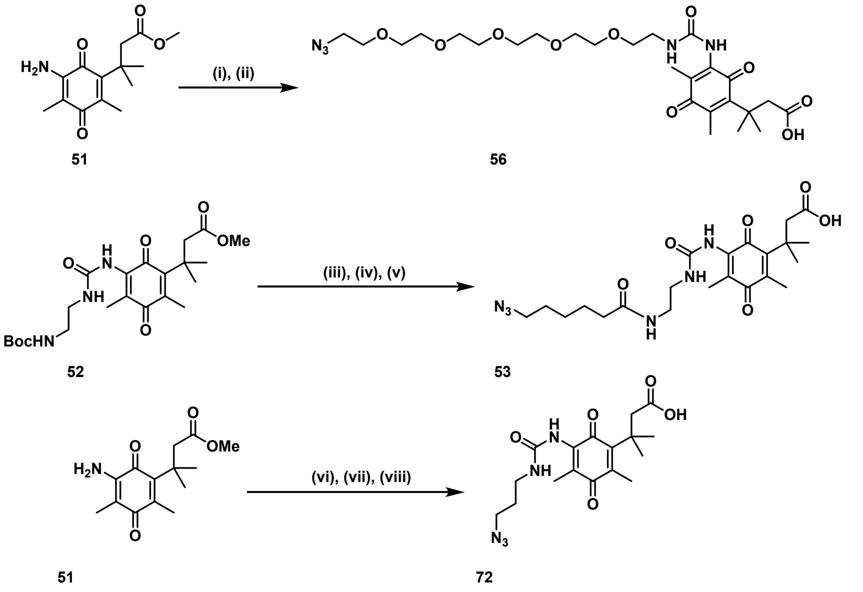

Figure S8. Synthesis of azido TML linkers **53, 56** and **72.** (i) triphosgene, toluene, 80 °C, overnight, then reduced pressure, 17-azido-3,6,9,12,15-pentaoxaheptadecan-1-amine, TEA, DCM, 0-24 °C, quant. crude, (ii) 1 M LiOH, MeOH, 23 °C, 2 h, 95%, (iii) 25% TFA, anhydrous DCM, 0-24 °C, 2 h, (iv) 6-azido hexanoic acid, HATU, DIPEA, 24 °C, 2 h, (v) 1 M LiOH in H_2O, MeOH, 3 h, 24 °C, 74% over three steps. (i) triphosgene, toluene, 80 °C, overnight, then reduced pressure, 3-azido-propan-amine, TEA, DCM, 0-24 °C, (ii) 1 M LiOH, MeOH, 23 °C, 2 h, 48% over two steps,

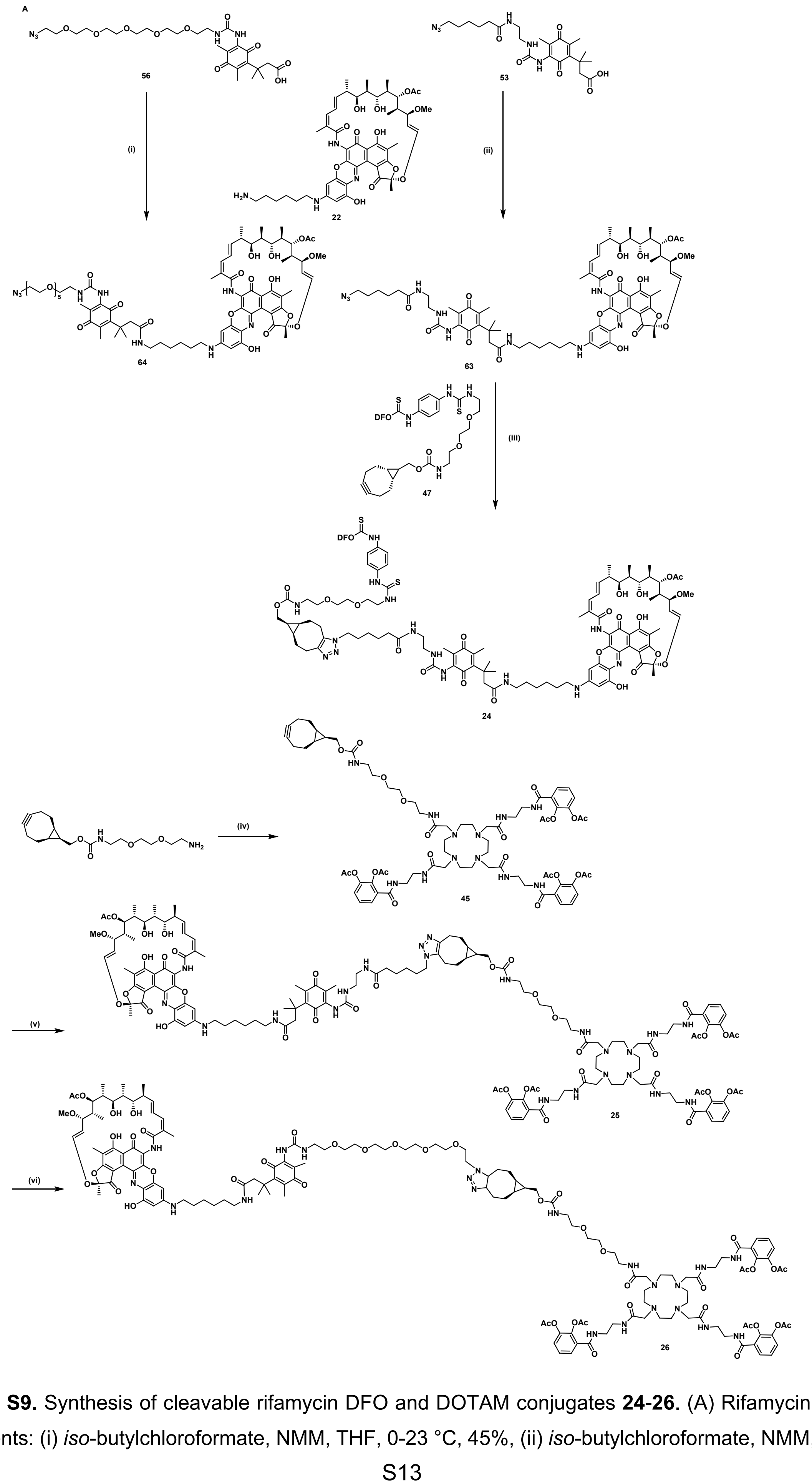

Figure S9. Synthesis of cleavable rifamycin DFO and DOTAM conjugates **24**-**26**. (A) Rifamycin linker fragments: (i) *iso*-butylchloroformate, NMM, THF, 0-23 °C, 45%, (ii) *iso*-butylchloroformate, NMM, THF,

0-23 °C, 39%, (B) DFO conjugate: (iii) MeOH, 24 °C, overnight, 81%, (C) DOTAM conjugates: (iv) **7**, *iso*-butylchloroformate, NMM, THF, 0-24 °C, 79% crude, (v) **63**, ACN, H_2O, 24 °C, overnight, 79%, (vi) **64**, ACN, H_2O, 24 °C, overnight, 82%.

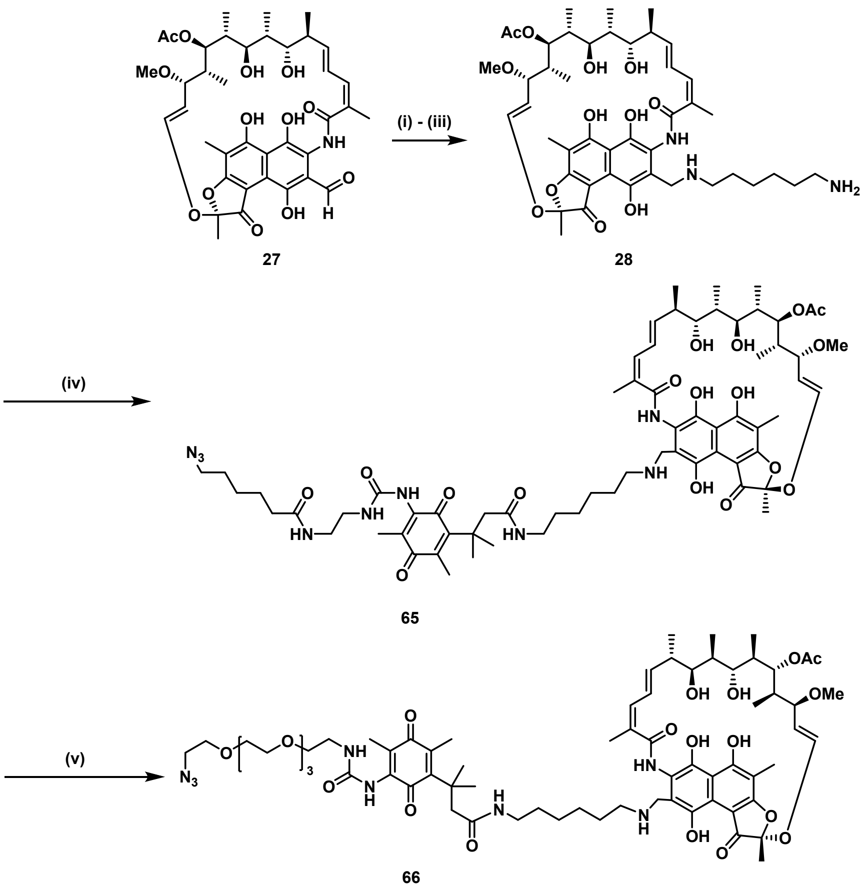

Figure S10. Synthesis of rifamycin trimethyl lock linker fragments **65** and **66**. (i) *N*-Fmoc 1,6-diaminohexane, TEA, THF, 1 h, (ii) $NaBH(OAc)_3$, THF, 0 - 23 °C, overnight, (iii) 20% diethylamine, ACN, 23 °C, 45 min, 67% over three steps, (iv) **53**, *iso*-butylchloroformate, NMM, THF, 0-23 °C, overnight, 73%, (v) **56**, *iso*-butylchloroformate, NMM, THF, 0-23 °C, overnight, 76% (38% yield f. EDCI based coupling, not shown).

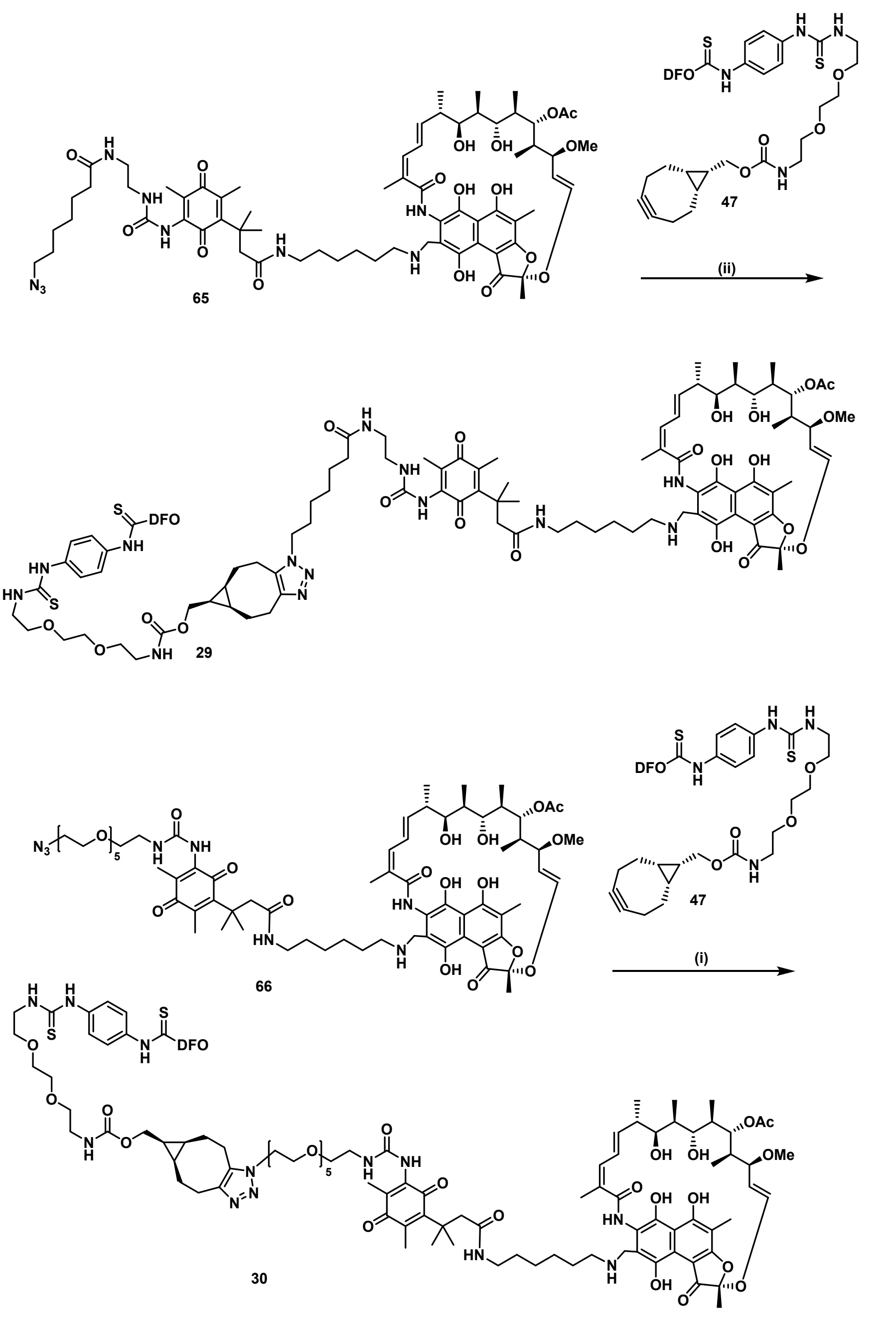

Figure S11. Synthesis of cleavable rifamycin trimethyl lock DFO conjugates **29** and **30**. (i) ACN, H_2O, (1:1), 24 °C, 30 h, 64%, (ii) ACN, H_2O, (1:1), 24 °C, 30 h, 88%.

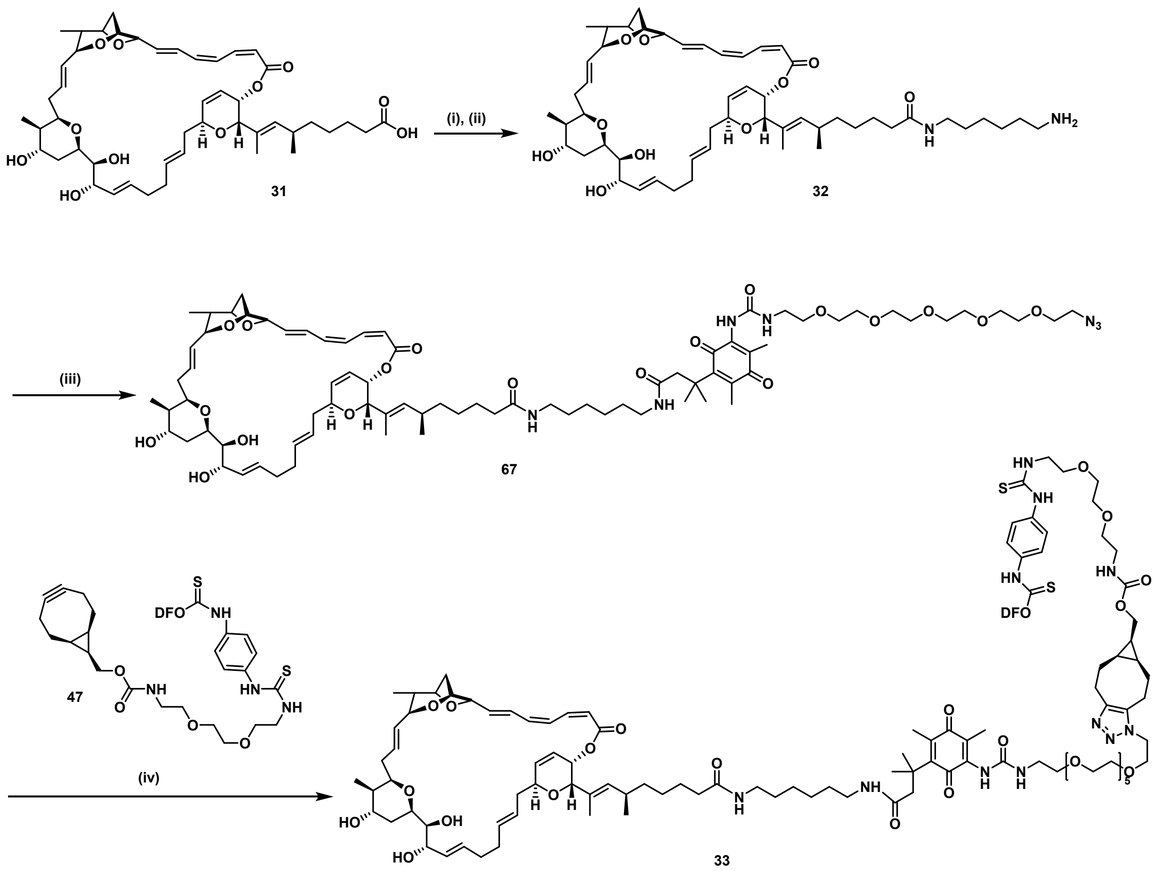

Figure S12. Synthesis of cleavable sorangicin trimethyl lock DFO conjugate **33**. (i) *N*-Fmoc 1,6-diaminohexane, DIPEA, DCM, DMF, 23 °C, 2 h, (ii) 20% diethylamine, ACN, 1 h, 56% over two steps, (iii) **56**, *iso*-butylchloroformate, NMM, THF, 0-23 °C, overnight, 84%, (iv) ACN, H_2O, (1:1), 24 °C, 30 h, 71%.

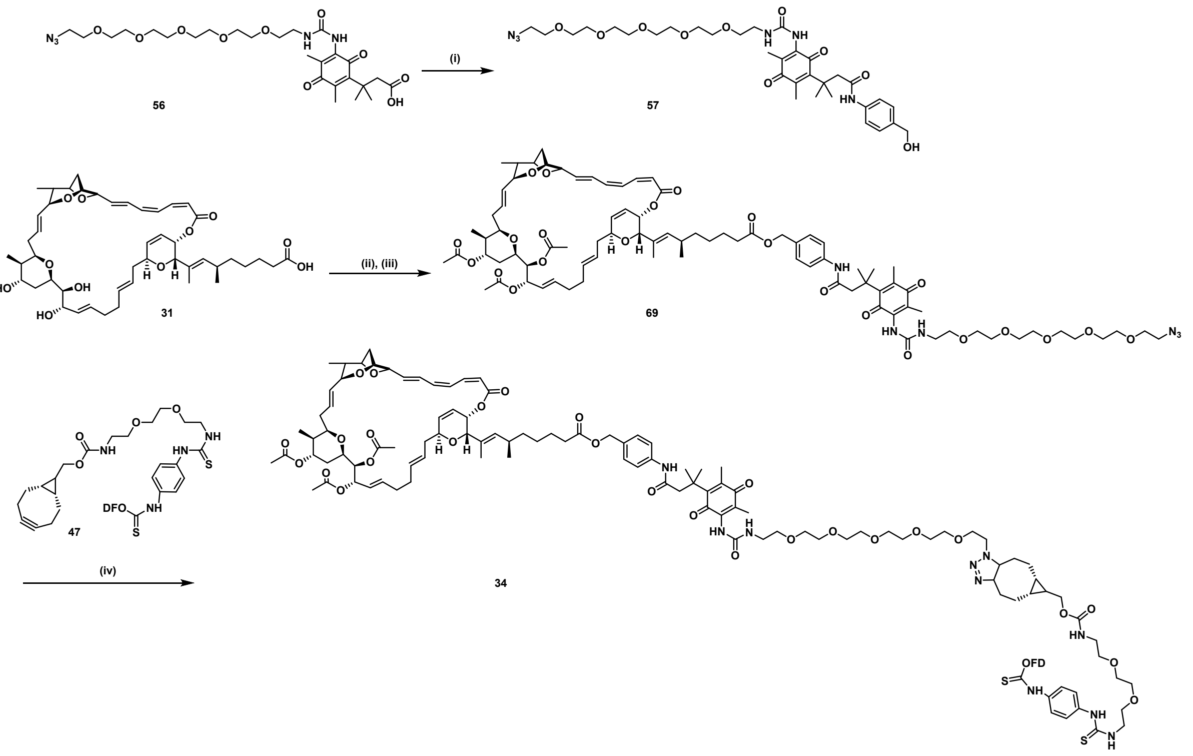

Figure S13. Synthesis of cleavable sorangicin trimethyl lock DFO conjugate **34**. (i) para-amino benzyl alcohol, *iso*-butylchloroformate, NMM, THF, 0-23 °C, overnight, 77%, (ii) acetic anhydride, THF, pyridine, 24 °C, quant. crude, (iii) *iso*-butylchloroformate, NMM, THF, 10 min 0 °C, 45 min 24 °C, then **57**, NMM, THF, 0 – 24 °C, overnight, 63% over two steps, (iv) ACN, H_2O (1:1), 23 °C, 36 h, 95%.

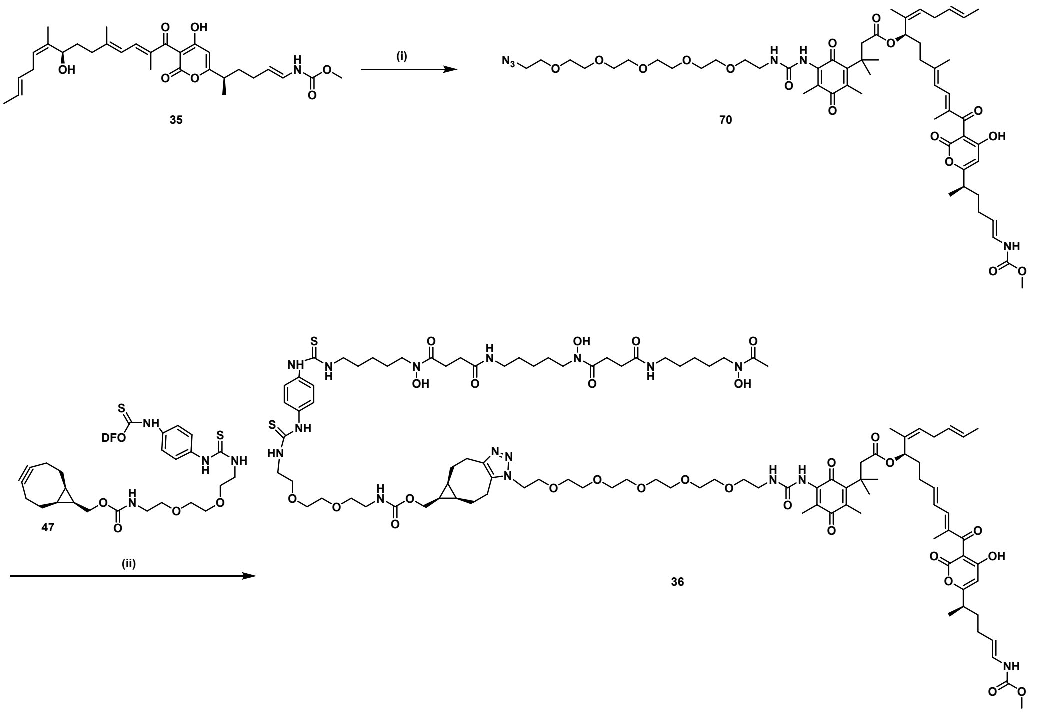

Figure S14. Synthesis of cleavable corallopyronin A conjugate **36**. (i) **56**, *iso*-butylchloroformate, NMM, THF, 0 - 24 °C, overnight, 71%, (ii) ACN, H_2O (1:1), 24 °C, 30 h, 79%.

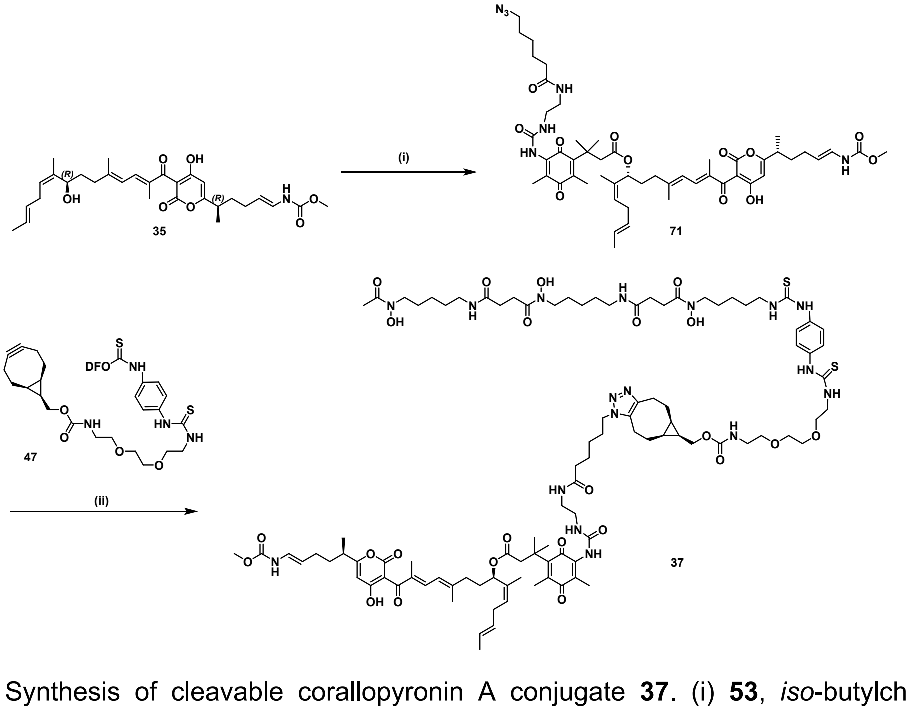

Figure S15. Synthesis of cleavable corallopyronin A conjugate **37**. (i) **53**, *iso*-butylchloroformate, pyridine, THF, 0 - 24 °C, 48 h, 75%, (ii) ACN, H_2O (1:1), 24 °C, 30 h, 49%.

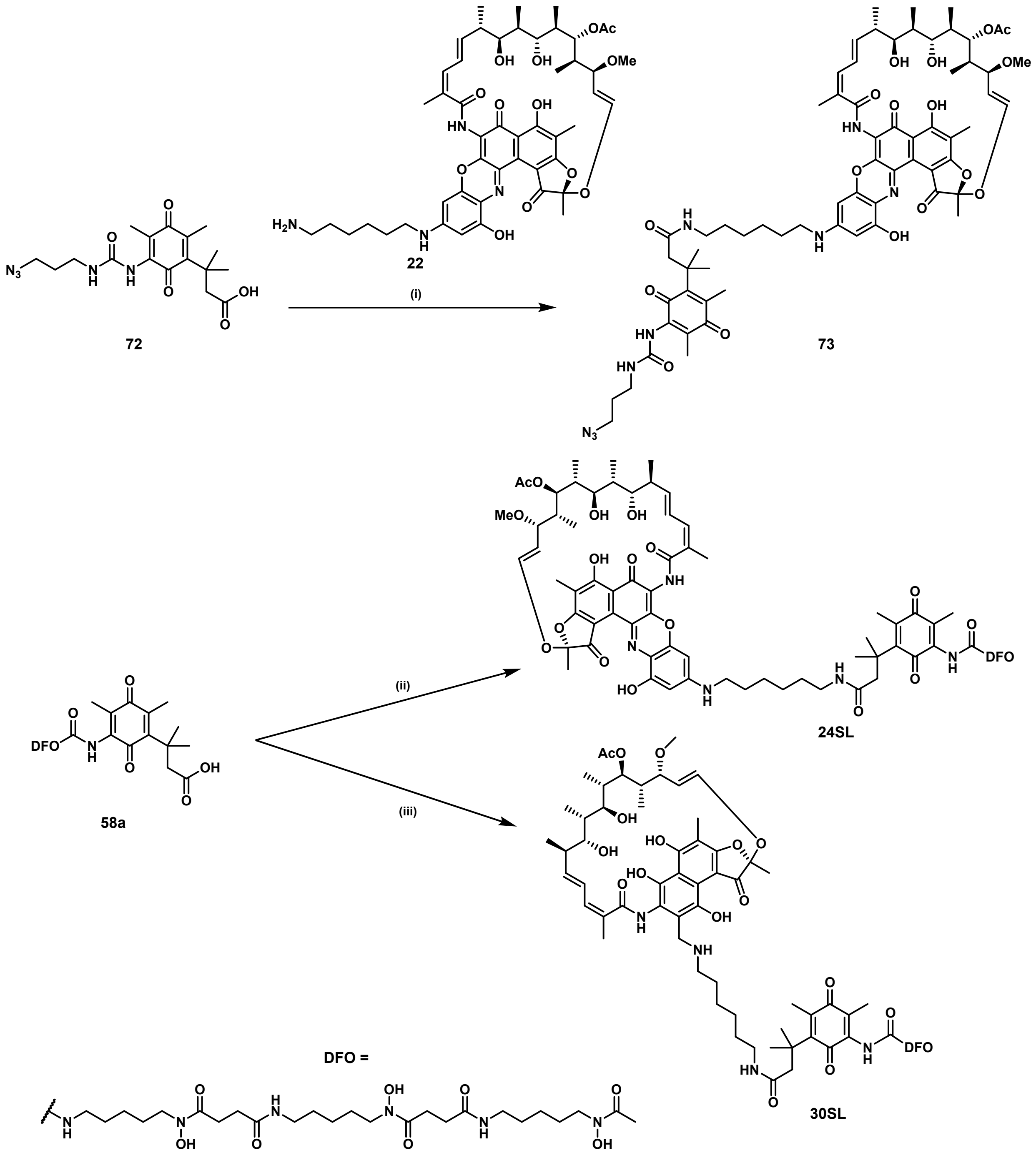

Figure S16. Synthesis of rifamycin S and 3CHO-rifamycin SV conjugates **73, 24SL** and **30SL** with a shorter linker between siderophore and cleavable TML. (i) iso-butyl chloroformate, NMM, DCM, 0-23 °C, 34%, (ii) EDCI, HOBt, DMAP, DMF, then **22**, DIPEA, DMF, 0-23 °C 74%, (iii) EDCI, HOBt, DMAP, then **28**, DIPEA, DMF, 0-23 °C, 47%.

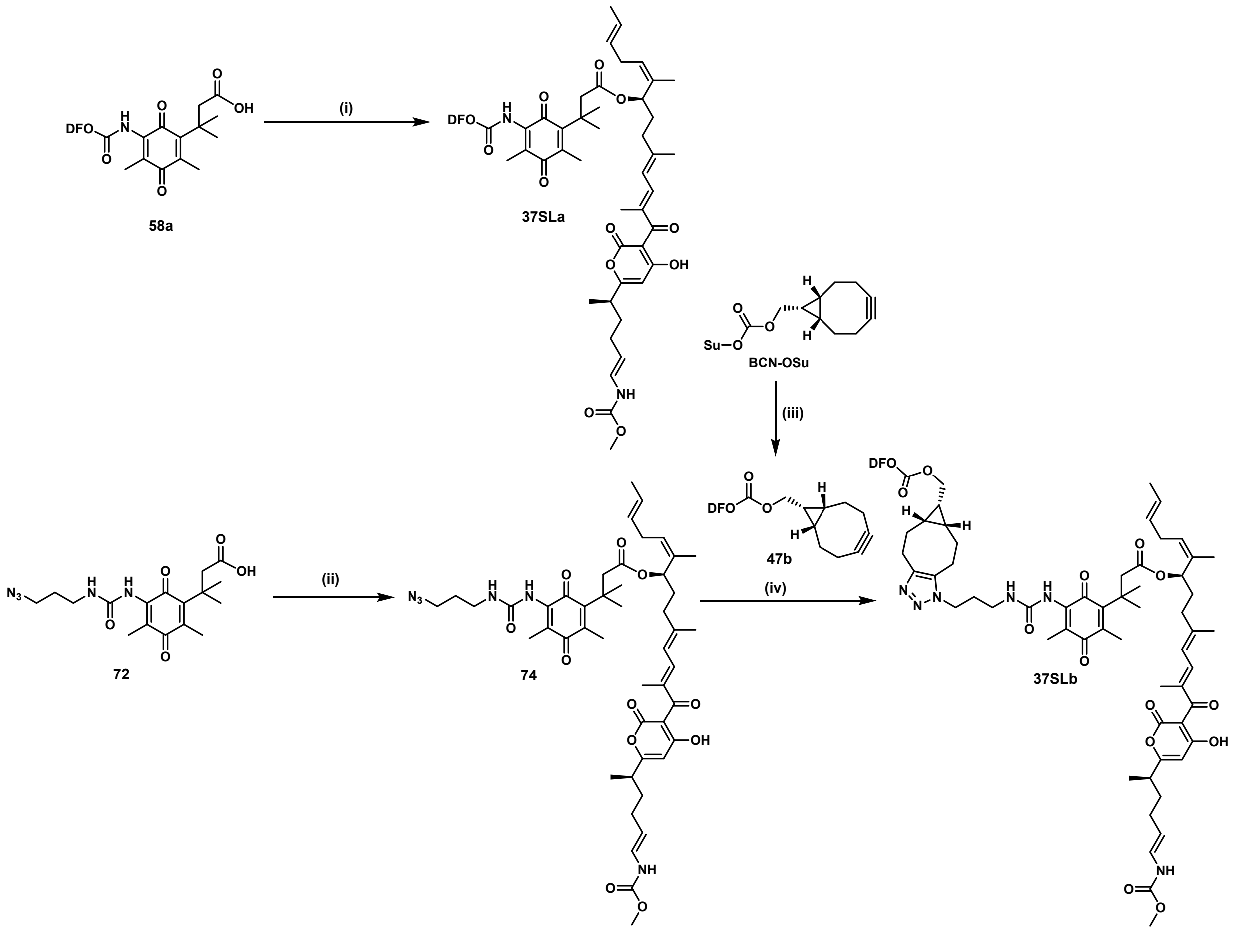

Figure S17. Synthesis of CorA conjugates **74**, **37SLa** and **37SLb** with a shorter linker between siderophore and cleavable linker. (i) EDCI, HOBt, DMAP, DMF, 0-23 °C, then **35**, DMF, 0 -23 °C, 28%, (ii) iso-butyl chloroformate, NMM, DCM, 0-23 °C, then **35**, DCM, 0-23 °C, 22%, (iii) DFO, DMF, TEA, 0-23 °C, 85%, (iv) **47b**, ACN/H_2O, 23 °C, ON, 20%.

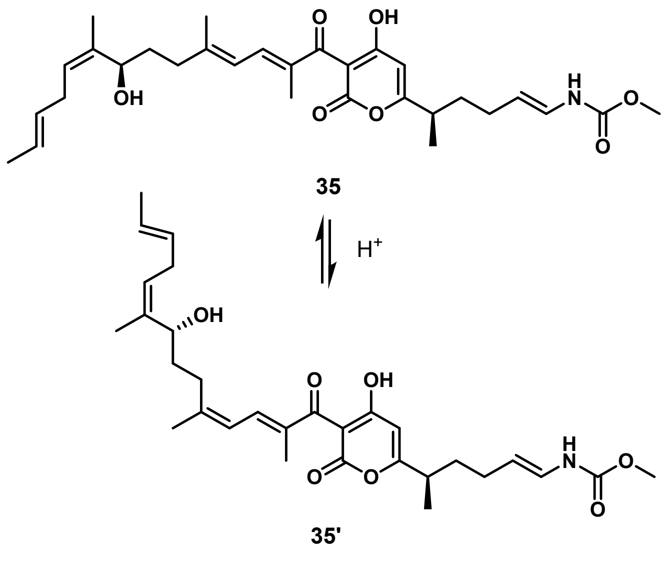

Figure S18. Isomerization of CorA **35** to **35‘** under non-neutral conditions.

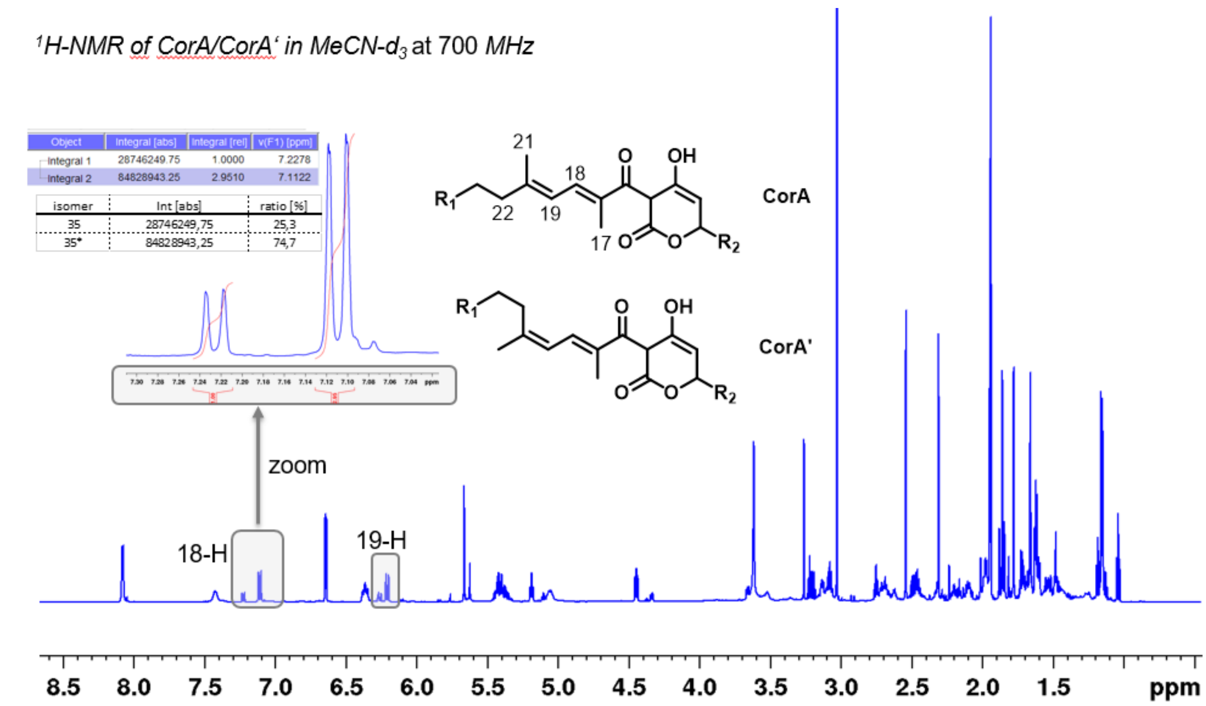

Figure S19. NMR analysis of **35** to **35'** ratio after re-isolation from synthesis towards **37** (figure S17).

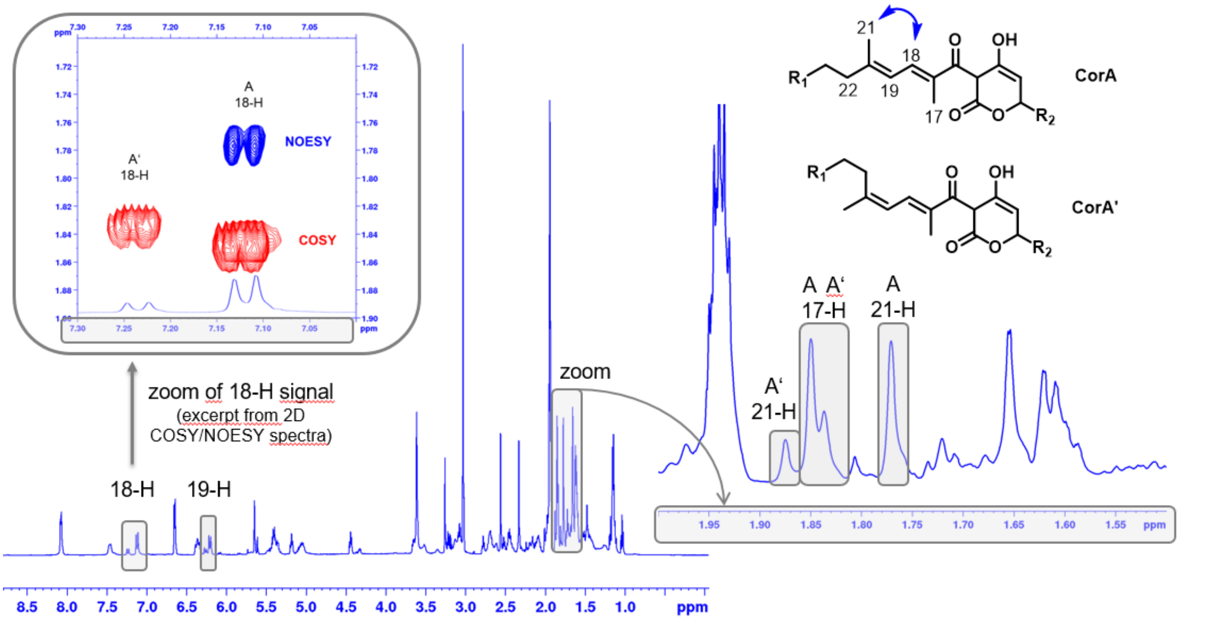

Figure S20. NMR analysis of NOESY and COSY spectra to corroborate *E/Z* configuration of reisolated isomers **35** and **35‘**. *E* configuration in **35** verified via NOESY signal between 18-H signal and 21-Me signal.

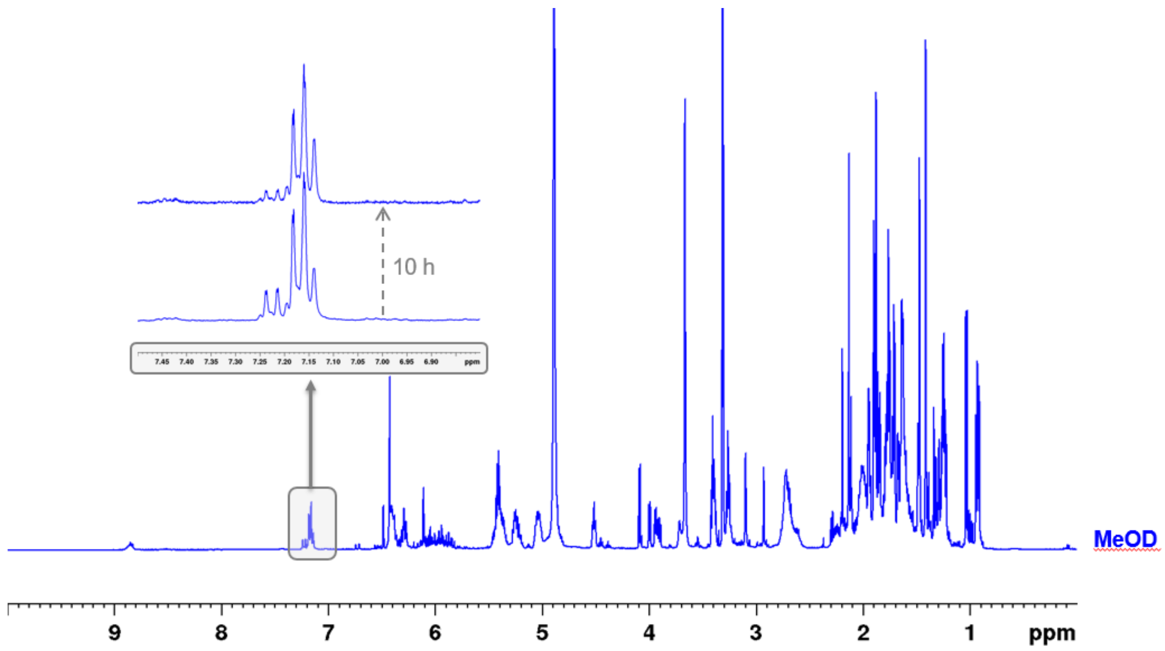

Figure S21. NMR analysis of isomeric ratio in **37**. Signal overlap in the region of C_{19}-C_{20} double bond signals impeded integration of specific signals, even though measurements were conducted in five different deuterated solvents (MeOH-d_4, MeCN-d_3, DMSO-d_6, THF-d_8, Pyridin-d_5). Isomeric ratio changes over time (^{1}H NMR spectrum of the same sample after ten hours) and with increasing temperature.

Synthesis procedures

Siderophore synthesis

Compound **38**

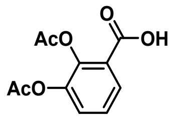

Chemical Formula: $C_{11}H_{10}O_6$
Exact Mass: 238,0477
Molecular Weight: 238,1950

Compound **38** was synthetized as published in C. Peukert, L. Langer et al.[4]

Compound **39**

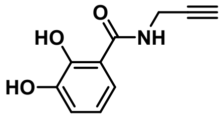

Chemical Formula: $C_{10}H_9NO_3$
Exact Mass: 191,0582
Molecular Weight: 191,1860

Acid **38** (100 mg, 0.42 mmol, 1.0 eq) and HATU (175.6, 0.46 mmol, 1.1 eq.) were dissolved in 200 µL of a 1:1 mixture of DCM/DMF. Then, DIPEA (298.7 µL, 1.68 mmol, 4.0 eq) was added, which was followed by the dropwise addition of propagylamine (23.1 mg, 0.42 mmol, 1.0 eq, diluted in 100 µL 1:1 DCM/DMF) over 10 minutes at 0 °C. Deacetylation was driven to completion by addition of MeOH (200 µL) and DIPEA (50 µL). The reaction mixture was concentrated *in vacuo* to yield **39** as a crude colorless oil (63.5 mg, 0.332 mmol, 79%) that became crystalline at 0 °C.

HRMS (ESI) calculated for ($[M+H]^+$): m/z = 192.0654; experimental = 192.0655

Compound **40**

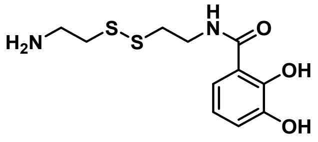

Chemical Formula: $C_{11}H_{16}N_2O_3S_2$
Exact Mass: 288,0602
Molecular Weight: 288,3800

Catechol **38** (94.38 mg, 0.396 mmol, 1.0 eq) was dissolved in anhydrous THF (15 mL). To the resultant solution, NMM (60 µL) was added under argon atmosphere at 0°C. Next, *iso*-butyl chloroformate (38.4 µl, 0.396 mmol, 1.0 eq) was added and upon addition, the reaction turned turbid instantly. The reaction mixture was stirred at 0 °C for 10 minutes and for 50 minutes at 23 °C. After this time, the mixture was cooled down to 0 °C again and *N*-Boc cystamine (100 mg, 0.396 mmol, 1.05 eq), suspended in THF (5 ml) was added basified with NMM (60 µl) dropwise over 5 minutes. The reaction was stirred for 10 minutes 0 °C and for 50 minutes at ambient temperature. Then AcOH (1 mL) was added and the reaction solvent was removed *in vacuo* and resultant residue dried under reduced pressure overnight. The next day, the residue was dissolved in DCM (10 mL) and cooled to 0 °C prior to the addition of a 1:1 mixture of TFA/AcOH (10 mL) was added at 0 °. The reaction mixture was stirred for two hours at 0 °C before the solvent was evaporated and the residue dried under reduced pressure. Then 20% DIPEA in MeOH (10 mL) was added and the reaction continued stirring for four hours at ambient temperature. The solvent was removed by rotary evaporation and the residue purified by RP-HPLC (10-85% ACN/H_2O, 0.1% HCOOH, 220 nm). The product containing fractions were lyophilized to yield amine **40** as beige oil (99.85 mg, 0.346 mmol, 87%).

^{1}H-NMR (500 MHz, ACN-d_3): δ = 7.87 (s, 1H), 7.16 (dd, J = 1.45, 8.1), 6.98 (dd, J = 1.74, 8.1 Hz, 1H), 6.75 (t, J = 8.1 Hz, 1H), 3.68 (q, J = 6.46, 12.82 Hz, 2H), 3.30 (t, J = 6.46 Hz, 2H), 3.03 (t, J = 6.65 Hz, 2H), 2.97 (t, J = 6.46 Hz, 2H), 2.30 (bs, 2H).

^{13}C-NMR (126 MHz, ACN-d_3): δ = 171.51, 161.50, 161.24, 150.50, 146.90, 119.48, 119.46, 39.58, 39.38, 37.92, 35.32.

HRMS (ESI) calculated for ($[M+H]^+$): m/z = 289.0675; experimental = 289.0683

Compound **41**

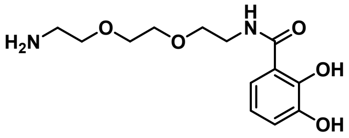

Chemical Formula: $C_{13}H_{20}N_2O_5$
Exact Mass: 284,1372
Molecular Weight: 284,3120

Catechol **38** (95,92 mg, 0.403 mmol, 1.0 eq) was dissolved in anhydrous THF (15 mL), NMM (60 µL) was added under argon atmosphere at 0 °C, followed by addition of *iso*-butyl chloroformate (38.4 µl, 0.403 mmol, 1.0 eq). The reaction turned turbid instantly and stirred at 0 °C for 10 minutes and for 50 minutes at 23 °C. After cooling to 0 °C, mono-boc-cystamin (100 mg, 0.403 mmol, 1.05 eq), suspended in THF (5 ml), was added as a suspension, together with NMM (60 µl), dropwise over 5 minutes. The reaction stirred for 10 minutes 0 °C and then for 50 minutes at ambient temperature. Then AcOH (1 mL) was added and the reaction was evaporated to dryness. The residue was dried overnight under reduced pressure, then dissolved in DCM (10 mL) and cooled to 0 °C where a mixture of TFA/AcOH (1:1, 10 mL) was added at 0 °C. The reaction stirred for two hours at 0 °C before the solvent was evaporated and the residue dried under reduced pressure. Then 20% DIPEA in MeOH (10 mL) was added and the reaction continued stirring for four hours at ambient temperature. The solvent was removed by rotary evaporation and the residue purified by RP-HPLC (C18, 10-85% ACN/H_2O, 0.1% HCOOH, 220 nm). The product containing fractions were lyophilized to yield amine **41** a beige oil (104,8 mg, 0.369 mmol, 93%).

^{1}H-NMR (500 MHz, ACN-d_3): δ = 7.92 (m, 1H), 7.19 (dd, J = 1.26, 8.00 Hz, 1H), 6.98 (dd, J = 1.71, 7.66 Hz, 1H), 6.72 (t, J = 7.89 Hz, 1H), 3.65 (m, 4H), 3.59 (s, 4H), 3.53 (q, J = 5.6, 11.20 Hz, 2H), 3.08 (t, J = 5.26 Hz, 2H), 2.60 (bs, 2H).

^{13}C-NMR (126 MHz, ACN-d_3): δ = 171.41, 150.41, 146.93, 119.52, 119.47, 118.29, 70.89, 70.78, 70.07, 67.24, 40.45, 40.10.

HRMS (ESI) calculated for ($[M+H]^+$): m/z = 285.1445; experimental = 285.1439

Compound **42**

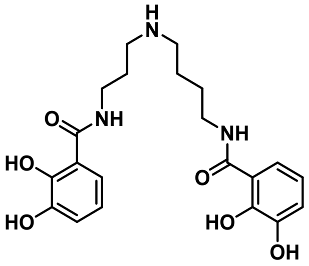

This compound was previously synthetized by Miller et al.[5] Catechol **38** (98.4 mg, 0.41 mmol, 1.2 eq) was dissolved in dry DCM (14 mL) and carbonyldiimidazole (66.98 mg, 0.41 mmol, 1.2 eq) was added in 3 portions at 0 °C. The mildly orange solution was stirred 1 hour at 0 °C under argon atmosphere and one more 1 hour at 25 °C. Once again the reaction temperature was decreased to 0 °C and spermidine (50 mg, 0.34 mmol, 1.0 eq) was added as solution in anhydrous DCM (1 mL). Upon addition formation of a precipitate was instantly oberved. The reaction mixture was stirred at 25 °C overnight. Following phase separation, the organic phase was washed with brine/water (1:1, 2x50 mL), dried over Na_2SO_4 and the solvent was removed *in vacuo*. The residue was dissolved in MeOH (8 mL) and DIPEA (2 mL) was added. The deacetylation reached full conversion after 4 hours and the solvent was removed by rotary evaporation, yielding a beige oil that was dried *in vacuo* to give crude dicatechol **42** (104.5 mg, 0.25 mmol, 73%) which was used without purification directly in the next step.

Compound **7**

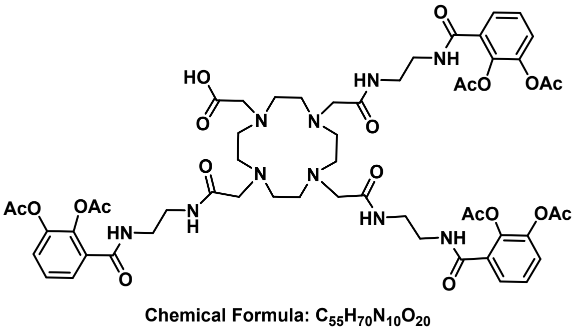

Compound **7** was synthetized as previously described by C. Peukert and L. N. B. Langer et al.[4]

^{1}**H-NMR** (700 MHz, DMSO-d6+AcOH-d4): δ = 8.60 (m, 1H), 8.10 (m, 2H), 7.53 (m, 3H), 7.34 (m, 8H), 7.22 (m, 1H), 3.53 (m, 2H), 3.40 (m, 4H), 3.25 (m, 6H), 3.13 (m, 10H), 3.02 (m, 2H), 2.96 (s, 2H), 2.87 (m, 4H), 2.78 (m, 2H), 2.59 (m, 8H), 2.27 (s, 9H), 2.21 (s, 9H).

^{13}**C-NMR** (176 MHz, DMSO-d6+AcOH-d4): δ = 172.03, 171.99, 170.61, 170.52, 170.25, 170.17, 168.57, 168.32, 168.26, 168.13, 167.85, 167.81, 167.67, 166.71, 165.65, 164.62, 164.58, 164.54, 164.49, 142.83, 142.58, 140.21, 140.18, 139.17, 138.55, 131.73, 130.71, 130.66, 130.61, 130.56, 126.72, 126.25, 126.17, 126.10, 125.42, 125.33, 124.89, 124.29, 57.71, 57.12, 53.11, 51.65, 50.29, 47.80, 39.52, 38.93, 38.81, 38.34, 38.16, 38.08, 34.23, 21.05, 20.69, 20.58, 20.47, 20.43, 20.38, 20.34, 20.25, 20.13, 19.99, 1.15.

HRMS (ESI) calculated for ($[M+H]^+$): m/z = 1191.4841; experimental = 1191.4846

Compound **43**

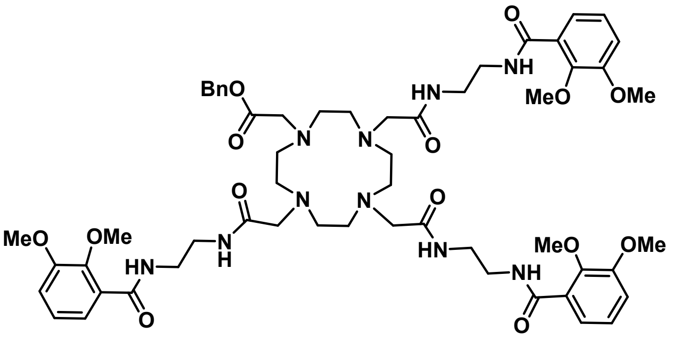

Chemical Formula: $C_{56}H_{76}N_{10}O_{14}$
Exact Mass: 1112,5542
Molecular Weight: 1113,2800

Cyclen (20 mg, 0.116 mmol, 1.0 eq) was dissolved in ACN (75 mL) and NaOAc (31.4 mg, 0.371 mmol, 3.2 eq) was added. Then *tert*-butyl (2-(2-bromoacetamido)ethyl)carbamate (67.3 mg, 0.371 mmol, 3.2 eq), synthetized according to Peukert and Langer et al,[6] was added, dissolved in ACN (25 mL) dropwise at 24 °C over 60 minutes. The reaction was stirred overnight at 24 °C and then filtered. The reaction was filtered and the flowthrough was concentrated. The resultant transparent oil was dissolved in ACN (75 mL) and K_2CO_3 (32.1 mg, 0.23 mmol, 2.0 eq) was added. Next, benzyl bromoacetate (31.9 mg, 0.232 mmol, 2.0 eq) was added as solution in ACN (25 mL) over the course of 10 minutes at 24 °C. The reaction mixture was stirred for two hours at ambient temperature and was then filtered. The filtrate was evaporated to dryness. The residue was taken up in DCM (100 mL) and washed with HCl (2x100 mL), $NaHCO_3$ (2x100 mL), water (2x200 mL) and dried over Na_2SO_4. The solvent was

removed in *vacuo* and the beige solid was dissolved in anhydrous DCM (25 mL). Then, the reaction was cooled to 0 °C and TFA (25 mL) was added. The reaction continued stirring for 2 hours, was then evaporated to dryness and dried under high vacuum overnight. The next day 2,3-dimethoxybenzoic acid (84.6 mg, 0.464 mmol, 4.0 eq) was dissolved under argon atmosphere in dry DCM:DMF (50:2 mL). Then HATU (264.9 mg, 0.697 mmol, 6.0 eq) was added, followed by DIPEA (250 µL). The slight yellow solution was stirred 5 minutes at 24 °C before the crude amine **43a** in anhydrous DCM:DMF (50:3 mL) with DIPEA (250 µL) was added dropwise. Upon addition, the color changed to yellow and the reaction stirred overnight at ambient temperature. Then the solvent was removed *in vacuo*. The residue was taken up in DCM (100 mL) and washed with HCl (2x100 mL), $NaHCO_3$ (2x100 mL), water (2x200 mL) and dried over Na_2SO_4. The residue was purified by RP-HPLC (10-70% ACN/H_2O, 0.1 % HCOOH, 220 nm) and product containing fractions were jointly lyophilized to yield ester **43** (94.46 mg, 0.085 mmol, 73% over three steps) as a white powder.

^{1}H-NMR (500 MHz, DMSO-d6): δ = 8.66 (m, 1H), 8.30 (m, 4H), 8.12 (m, 1H), 7.75 (m, 3H), 7.35 (m, 8H), 7.15 (m, 11H), 5.10 (s, 2H), 3.96 (m, 6H), 3.82 (s, 9H), 3.74 (s, 9H), 3.49 (m, 4H), 3.39 (m, 15H), 3.14 (m, 18H).

^{13}C-NMR (176 MHz, DMSO-d6): δ = 165.85, 165.69, 152.52, 152.49, 146.36, 146.31, 135.59, 129.56, 129.46, 128.43, 128.38, 128.31, 128.22, 128.13, 128.05, 123.97, 120.75, 120.66, 114.87, 114.78, 66.50, 65.85, 60.95, 60.91, 55.95, 55.93, 54.68, 52.33, 50.86, 48.19, 47.73, 38.54, 38.47, 38.05.

HRMS (ESI) calculated for ($[M+H]^+$): m/z = 1113.5615; experimental = 1113.5618

Compound **44**

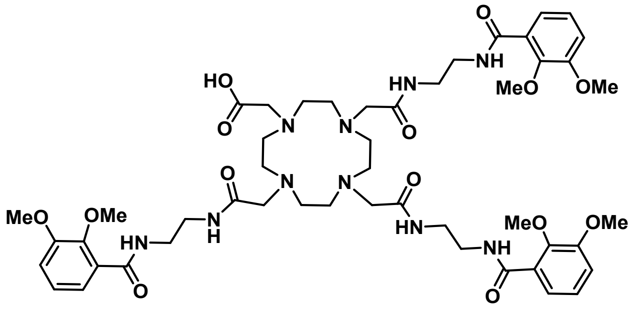

Chemical Formula: $C_{49}H_{70}N_{10}O_{14}$
Exact Mass: 1022,5073
Molecular Weight: 1023,1550

Under argon atmosphere, benzyl ester **43** (253 mg, 0.227 mmol, 1.0 eq) was dissolved in MeOH (30 mL) andPd/C (25 mg, 0.1eq) was added. The atmosphere was changed to hydrogen and the reaction was stirred at 25 °C overnight. After this, the catalyst was removed by filtration using a syringe filter and the reaction mixture was evaporated to dryness to yield crude pure acid **44** (232.4 mg, 0.227 mmol, quantitative) as a crude, white solid, which was used directly in the next step.

^{1}H-NMR (500 MHz, DMSO-d6): δ = 8.32 (m, 6H), 8.09 (m, 9H), 7.22 (s, 9H), 7.07 (s, 9H), 3.83 (m, 8H), 3.75-2.57 (m, 28H).

HRMS (ESI) calculated for ([M+H]$^+$): m/z = 1023.5146; experimental = 1023.5159

Compound **45**

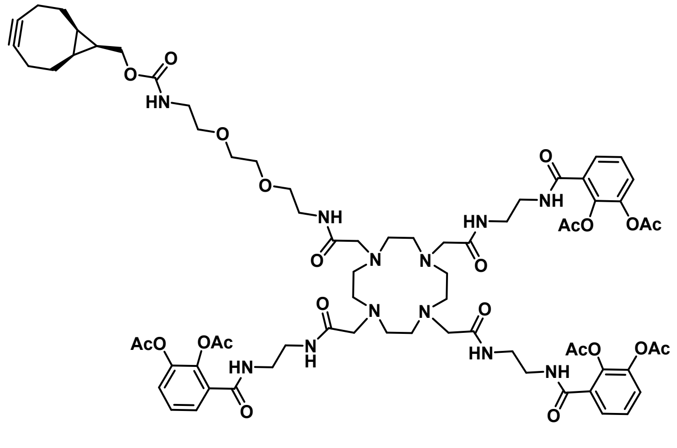

Chemical Formula: $C_{72}H_{96}N_{12}O_{23}$
Exact Mass: 1496,6711
Molecular Weight: 1497,6210

To a solution of acid **7** (81 mg, 0.068 mmol, 1.0 eq) in dry THF (1 mL), NMM (100 µL) was added under argon atmosphere and the resultant mixture was cooled to 0 °C. Then, *iso*-butylchloroformate (6.59 µL, 0.068 mmol, 1.0 eq) was added and the reaction mixture went

turbid instantly. Agitation was continued for 15 minutes at 0 °C and for another hour at 24 °C. Then, BCN amine (25.37 mg, 0.078 mmol, 1.15 eq) was added dropwise at 0 °C, dissolved in anhydrous THF (1 mL) and basified with NMM (100 µL) before addition. The reaction continued stirring at 0 °C for 15 minutes and then for 1 hour at 24 °C. Then the reaction was quenched with AcOH (200 µL) and the THF was removed *in vacuo* at 30 °C. The residual solution was diluted 50 times and lyophilized to dryness to yield **45** as a crude white powder (19.06 mg, 0.01 mmol, 79%).

1H NMR data?

HRMS (ESI) calculated for ($[M+H]^+$): m/z = 1497.6784; experimental = 1497.6761, calculated for ($[M+2H]^{2+}$): m/z = 749.3428; experimental = 749.3412.

Compound **46**

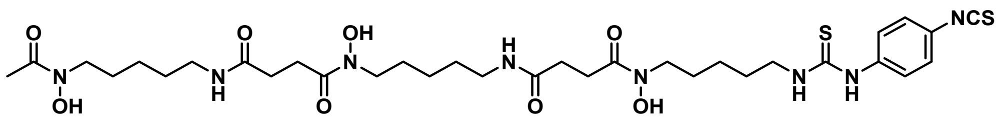

Chemical Formula: $C_{33}H_{52}N_8O_8S_2$
Exact Mass: 752,3350
Molecular Weight: 752,9470

Crude **46** was synthetized according to a patent by P. S. Donnely et al.[7] In particular, desferrioxamine (DFO, 1000 mg, 1.78 mol, 1.0 eq) was stirred in *i*PrOH/H_2O (64:6 mL), and a solution of 1,4-dithiocyanatobenzene (1542.11 mg, 8.02 mol, 4.5 eq) in $CHCl_3$ (20 mL) was added. Triethylamine (500 µL) was added, and the reaction mixture was stirred for 1.5 h at 25 °C. HCl (0.1 M, 100 mL) was added and the organic layer was separated. The solvent was evaporated to give a beige solid which was triturated with $CHCl_3$. The remaining solid was filtered off and dried to give modified DFO **46** as a white powder (1033.3 mg, 1.372 mol, 89%).

^{1}H-NMR (500 MHz, DMSO-d6): δ = 8.96 (m, 1H), 8.45 (m, 1H), 8.00 (m, 7H), 7.74 (m, 1H), 7.38 (s, 4H), 5.11 (s, 2H), 4.00 (m, 2H), 3.71 (m, 2H), 3.50 (m, 5H), 3.37 (m, 6H), 3.29 (m, 2H), 3.12 (m, 4H), 3.01 (m, 2H), 2.92 (m, 6H), 2.08 (s, 2H), 1.91 (s, 1H).

^{13}C-NMR (126 MHz, DMSO-d6): δ = 171.89, 171.36, 170.08, 139.91, 125.99, 122.01, 47.05, 46.75, 43.34, 38.40, 30.02, 28.78, 28.00, 27.72, 26.02, 23.59, 23.52, 20.43.

HRMS (ESI) calculated for ([M+H]$^+$): m/z = 753.3423; experimental = 753.3525.

Compound **47**

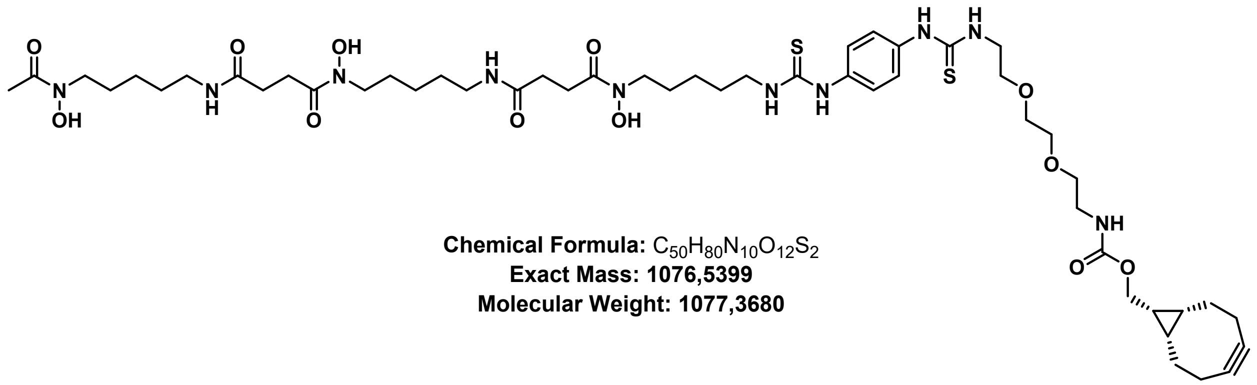

46 (209 mg, 0.278 mmol, 1.0 eq) was suspended in DMSO (5 mL) and the mixture was stirred until complete dissolution (5 min) was observed. Then, N-[(1R,8S,9s)-bicyclo[6.1.0]non-4-in-9-ylmethyloxycarbonyl]-1,8-diamino-3,6-dioxaoctan (Sigma aldrich, 100.86 mg, 0.311 mmol, 1.12 eq) was dissolved in DMSO (5 mL), added dropwise and following this, TEA (300 µL) was added. The slightly turbid solution cleared after 10 minutes and was stirred for 18 h at ambient temperature. The next morning, the reaction was filtered and purified by RP-HPLC (5-75% H_2O/ACN, 0.1% HCOOH, 220 nm). The product containing fractions were identified by LCMS and lyophilized to dryness to yield strained alkyne **47** as a white powder (153.1 mg, 0.142 mmol, 51%).

^{1}H-NMR (500 MHz, DMSO-d6): δ = 9.60 (m, 3H), 9.41 (m, 1H), 7.77 (m, 2H), 7.64 (m, 1H), 7.34 (s, 4H), 4.02 (d, J = 8.34 Hz, 2H), 3.63 (m, 2H), 3.53 (m, 6H), 3.45 (m, 8H), 3.40 (m, 2H), 3.33 (m, 1H), 3.11 (q, J = 5.64, 5.89, 2H), 3.00 (q, J = 7.11, 13.49 Hz, 4H), 2.58-2.54 (m, 4H), 2.26 (m, 4H), 2.20 (m, 1H), 2.15 (m, 2H), 1.96 (s, 3H), 1.52 (m, 10H), 1.38 (q, J = 7.11, 14.96 Hz, 4H), 1.24 (m, 6H), 0.85 (t, J = 9.12 Hz, 1H).

^{13}C-NMR (126 MHz, DMSO-d6): δ = 180.50, 180.28, 171.97, 171.31, 170.13, 156.46, 123.33, 99.00, 69.54, 69.17, 68.59, 61.38, 47.08, 46.78, 43.76, 43.58, 40.43, 39.52, 38.43, 29.90, 28.82, 28.59, 28.22, 27.57, 26.13, 26.03, 23.60, 23.50, 20.86, 20.36, 19.55, 17.66.

HRMS (ESI) calculated for ([M+H]$^+$): m/z = 1077.5472; experimental = 1077.5473

Compound **47b**

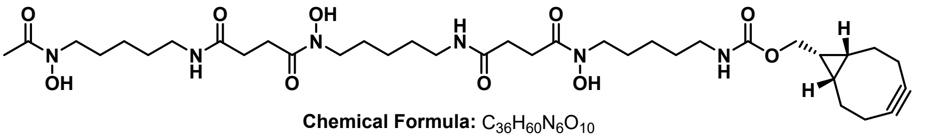

Chemical Formula: $C_{36}H_{60}N_6O_{10}$
Exact Mass: 736,4371
Molecular Weight: 736,9080

DFO mesylate (SigmaAldrich, # D9533-1G, 225.5 mg, 0.34 mol, 1.0 eq) was solubilized in anhydrous DMF (20 mL) by gentle heating with a heat gun. This solution was quickly cooled to 0 °C and DIPEA (100 µL) was added. Then BCN-OSu (SigmaAldrich, # 744867-100MG, 100 mg, 0.34 mol, 1.0 eq) was added dropwise in anhydrous DMF (5 mL) at 0 °C. Caution: Too long cooling to 0 °C lead to precipitation of the DFO mesylate and stalling of the reaction. The reaction continued stirring at 23 °C for 2 hours and the base was then removed by rotary evaporation at 30 °C. The residual solution was purified by RP-HPLC (2-50% ACN/H2O, 0.1% HCOOH, 220 nm, collect all), product containing fractions were lyophilized to yield 47b as a white powder (212.97 mg, 0.29 mol, 85%).

^{1}H-NMR (700 MHz, MeOH-d_4): δ = 4.14 (d, J = 8.16 Hz, 2H), 3.60 (m, 8H), 3.17 (t, J = 6.77 Hz, 4H), 3.09 (t, J = 2.06, 6.47 Hz, 2H), 2.76 (dt, J = 6.47, Hz, 4H), 2.45 (dt, J = 294, 7.21 Hz, 5H), 2.24 (m, 3H), 2.18 (m, 1H), 2.09 (s, 3H), 1.63 (m, 6H), 1.52 (m, 7H), 1.34 (m, 6H), 0.94 (t, J = 8.53 Hz, 1H).

^{13}C-NMR (176 MHz, MeOH-d_4): δ = 174.93, 174.49, 173.52, 159.34, 99.52, 63.56, 57.72, 57.60, 57.48, 57.35, 57.24, 41.57, 40.28, 31.48, 30.53, 30.17, 29.98, 28.92, 27.34, 24.91, 24.79, 21.94, 21.39, 20.23, 18.98, 17.50, 17.39, 17.28, 17.18, 17.06.

DEPT (176 MHz, MeOH-d_4): δ = 63.27, 41.29, 39.99, 31.21, 30.24, 29.89, 29.70, 29.67, 28.64, 27.06, 24.63, 24.60, 24.51, 21.65, 21.11, 19.94, 18.70.

HRMS (ESI) calculated for ([M+H]$^+$): m/z = 737.4444 experimental = 737.4446, calculated for ([M+Na]$^+$): m/z = 759.4263 experimental = 759.4266

Cleavable linker synthesis

Compound **48**

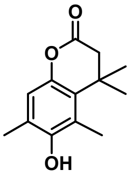

Chemical Formula: $C_{13}H_{16}O_3$
Exact Mass: 220,1099
Molecular Weight: 220,2680

Methanesulfonic acid (100 mL) was filled in a round bottom flask and heated to 70 °C. Then, 2,6-dimethylbenzene-1,4-diol (5.0 g, 36.21 mmol, 1.0 eq) and methyl 3-methylbut-2-enoate (4.7 g, 41.07 mmol, 1.1 eq) were added carefully, each in one portion, to yield a brown solution, which was stirred at 70 °C for 2 hours. After this time, the reaction was diluted with water (100 mL), extracted with EtOAc (3 x 100 mL) and the combined organic extracts were washed with water (1x75mL), sat. $NaHCO_3$ (2x 75 mL), brine (75 mL) and dried over Na_2SO_4. The solvent was removed *in vacuo* yielding lactone **48** as a crude, beige solid (7.2 g, 32.91 mmol, 91%), which was directly used in the next step without further purification.

^{1}H-NMR (500 MHz, $CDCl_3$): δ = 6.71 (s, 1H), 2.56 (s, 2H), 2.37 (s, 3H), 2.22 (s, 3H), 1.45 (s, 6H).

^{13}C-NMR (126 MHz, $CDCl_3$): δ = 168.67, 149.15, 144.77, 128.56, 122.50, 121.96, 116.78, 45.97, 35.35, 27.71, 15.87, 14.39.

Compound **49**

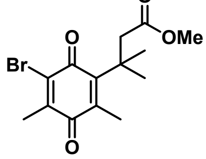

Chemical Formula: $C_{14}H_{17}BrO_4$
Exact Mass: 328,0310
Molecular Weight: 329,1900

Lactone **48** (7.2 g, 32.91 mmol, 1.0 eq) was dissolved in AcOH (120 mL)/H_2O (30mL) and bromine (1.5 g, 96.92 mmol, 4.0 eq) was added in AcOH (30 mL) using a dropping funnel over 15 minutes. The resultant brown-orange solution was stirred overnight, protected from light.

The solvent was evaporated by rotary evaporation in a fume hood (HBr/Br_2 fumes were quenched with sat. $Na_2S_2O_3$ in the collecting flask) and the residue was partitioned between water and DCM (each 200 mL). The aqueous phase was extracted with DCM (3x150 mL) and the combined extracts were extracted with sat. $NaHCO_3$ solution (8x250 mL). The $NaHCO_3$ extracts were acidified to pH < 3 with conc. HCl and extracted with DCM (3x 200 mL). The combined organic extracts were dried over Na_2SO_4 and the solvent was evaporated *in vacuo* yielding crude bromo acid **49a** (4254.4, 13.5 mmol, 56%) as a neon-yellow oil. The acid **49a** (4.3 g, 13.5 mmol, 1.0 eq) was dissolved directly in anhydrous MeOH (100 mL) and cooled to 0 °C. Then $SOCl_2$ (1.3 mL, 17.6 mmol, 1.3 eq) was added dropwise. The reaction was heated to reflux for two hours and then all volatiles were removed *in vacuo*. The residue was taken up in EtOAc and washed with sat. $NaHCO_3$ (2x 20 mL), brine (1 x20 mL) and dried over Na_2SO_4. The solvent was removed under reduced pressure to yield ester **49** (2.5 g, 7.5 mmol, 50%) as a crude brown-yellow oil.

^{1}H-NMR (500 MHz, $CDCl_3$): δ = 3.59 (s, 3H), 2.99 (s, 2H), 2.18 (s, 3H), 2.14 (s, 3H), 1.44 (s, 6H).

^{13}C-NMR (126 MHz, MeOH-d_4): δ = 186.32, 183.68, 174.63, 153.86, 142.19, 141.70, 141.19, 52.05, 48.41, 40.04, 29.28, 14.92, 13.65.

Compound **50**

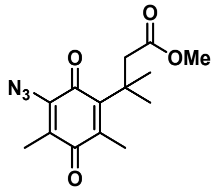

Chemical Formula: $C_{14}H_{17}N_3O_4$
Exact Mass: 291,1219
Molecular Weight: 291,3070

Bromide **49** (2.5 g, 7.5 mmol, 1.0 eq) was dissolved in MeOH (20 mL) and to this, sodium azide (1.5 g, 22.5 mmol, 3.0 eq) was added in one portion. Then, H_2O (20 mL) was added and the mixture stirred overnight at 25 °C. The organic portion was removed *in vacuo* and the residual phase was extracted with EtOAc (3 x 100 mL) and the combined extracts were washed with brine (1x 200 mL) and dried over Na_2SO_4. The solvent was removed *in vacuo* and yielded the azide **50** (2.2 g, 7.5 mmol, quant.) as an orange-brown oil.

^{1}H-NMR (500 MHz, $CDCl_3$): δ = 3.61 (s, 3H), 2.99 (s, 2H), 2.15 (s, 3H), 1.89 (s, 3H), 1.43 (s, 6H).

Compound **51**

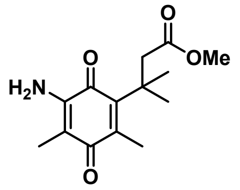

Chemical Formula: $C_{14}H_{19}NO_4$
Exact Mass: 265,1314
Molecular Weight: 265,3090

The azide **50** (2.2 g, 7.63 mmol, 1.0 eq) was dissolved in dry, degassed DCM (100 mL) and PPh_3 (3.6 g, 13.74 mmol, 1.8 eq) was added in three portions. The color of the reaction mixture became purple and it was stirred at 24 °C for 1 hour. Then, the solvent was evaporated by rotary evaporation at 40 °C and shortly dried under reduced pressure. The residue was dissolved in a mixture of AcOH/THF/H_2O (1:1:3, 600 mL) and heated to reflux (ca 100 °C) for 1 hour. The solvent was removed by rotary evaporation and EtOAc and H_2O (each 100 mL) were added. The organic phase was again washed with sat. $NaHCO_3$ (3 x 70 mL). The solution was concentrated and purified by silica gel chromatography (DCM/MeOH 4:1 to 2:1) yield amino TML **51** (642.4 mg, 2.42 mmol, 32%) as a blood-red oil. Purification by prepTLC (SiO_2, DCM, max. 5% MeOH) was used additionally to remove traces of triphenylphosphine oxide.

^{1}H-NMR (500 MHz, $CDCl_3$): δ = 7.80 (s, 1H), 3.10 (s, 2H), 2.91 (s, 3H), 2.76 (s, 3H), 2.13 (bs, 2H), 1.99 (s, 6H).

^{13}C-NMR (126 MHz, $CDCl_3$): δ = 168.75, 149.28, 144.99, 128.79, 122.58, 122.07, 116.98, 77.16, 46.16, 35.55, 27.90, 16.02, 14.55.

HRMS (ESI) calculated for ($[M+H]^+$): m/z = 266.1387; experimental = 266.1391.

Compound **52**

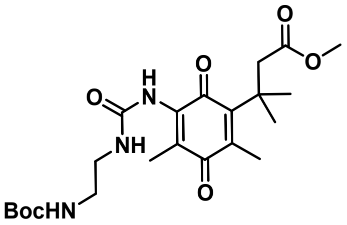

Amino TML **51** (200 mg, 0.754 mmol, 1.0 eq) was dissolved in anhydrous toluene (30 mL) and triphosgene (894.74 mg, 3.02 mmol, 4.0 eq) was added in one portion under argon atmosphere. The reaction was heated from 23 °C to 80 °C and stirred at that temperature overnight. A prominent color change from red to orange was observed after 2 hours. The next day, the solvent was removed by rotary evaporation at 40 °C and the residue was dried under reduced pressure for one hour. The carbamate was dissolved in anhydrous DCM (20 mL) and cooled to 0 °C under argon atmosphere. *N*-Boc ethylene-1,6-diamine (101 mg, 0.63 mmol, 6.0 eq) was added dropwise over 5 minutes, diluted in anhydrous DCM (20 mL), followed by addition of TEA (800 µL, 6.03 µmol, 8.0 eq) at the same temperature. The reaction continued stirring at 0 °C for 1 hours and for 1 hour at 23 °C and the color changed from orange to canary yellow. The reaction was diluted with DCM (100 mL) and washed with water (2x50 mL), 1 M HCl (2x 75 mL) and brine (2x 75 mL). The organic phase was dried over Na_2SO_4 and concentrated to dryness. The yellow oil was purified by multiple RP-HPLCs (10-60% ACN/H_2O, 0.1% HCOOH, 220 nm). Yellow fractions were verified to be pure product by LCMS, combined and lyophilized to yield **52** as a canary yellow oil (200.6 mg, 0.44 mmol, 59%).

^{1}H-NMR (500 MHz, DMSO-d6): δ = 8.11 (s, 1H), 6.74 (t, J = 5.36 Hz, 1H), 6.61 (t, J = 5.21 Hz, 1H), 3.49 (s, 3H), 3.01 (q, J = 6.92, 12.5 Hz, 2H), 2.89 (q, J = 6.92 Hz, 2H), 2.08 (s, 3H), 1.76 (s, 3H), 1.38 (s, 6H), 1.36 (s, 9H).

^{13}C-NMR (126 MHz, DMSO-d6): δ = 186.76, 186.56, 171.81, 155.59, 153.43, 149.93, 141.18, 139.20, 124.52, 77.31, 51.14, 46.33, 42.02, 37.97, 29.49, 29.46, 28.68, 28.28, 26.00, 13.95, 11.93.

HRMS (ESI) calculated for ([M+H]$^+$): m/z = 452.2392; experimental = 452.2379.

Compound **53**

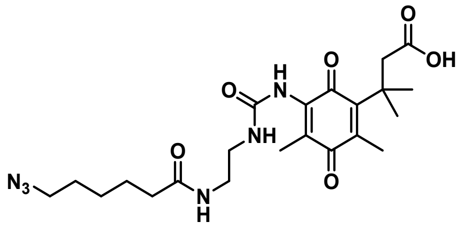

Chemical Formula: $C_{22}H_{32}N_6O_6$
Exact Mass: 476,2383
Molecular Weight: 476,5340

N-Boc protected amine **52** (100 mg, 0.221 mmol, 1.0 eq) was dissolved in anhydrous DCM (750 µL) and TFA (250 µL) was added at 0 °C. The reaction was stirred at 24 °C for 2 hours before the reaction was concentrated to dryness and dried overnight under reduced pressure. Then, 6-azido hexanoic acid (69.92 mg, 0.443 mmol, 2.0 eq) was dissolved in DCM:DMF (10:1, 18 + 2 mL) and HATU (336.86 mg, 0.886 mmol, 4.0 eq) was added. The suspension was stirred for 5 minutes, then the crude Boc-deprotected amine and DIPEA (50 µL) were added together in DCM:DMF (5 mL, 10:1). The reaction stirred for 2 hours at 24 °C and was then diluted with DCM (100 mL) and washed with LiCl (5% w/v, 1x 100 mL), 1 M HCl (2x100 mL) and brine (1x100 mL) before the organic phase was dried over Na_2SO_4. The solvent was removed by rotary evaporation and the residue was again dissolved in MeOH (9 mL) and 1 M LiOH (1 mL) was added. The reaction stirred for 3 hours at 24 °C and was then acidified by dropwise conc. HCl addition. The MeOH was removed by rotary evaporation and the residual aqueous phase (+ 10 mL water) was extracted with DCM (3x20 mL). The combined organic extracts were concentrated *in vacuo* and the residue was purified by RP-HPLC (10-60% ACN/H2O, 0.1% HCOOH, 220 nm). The product containing fractions were lyophilized to yield a yellow powder as **53** (78.3 mg, 0.164 mmol, 74% over three steps).

^{1}H-NMR (500 MHz, DMSO-d6): δ = 12.01 (s, 1H), 8.76 (dd, J = 1.34, 4.32 Hz, 1H), 8.54 (dd, J = 1.46, 8.5 Hz, 1H), 7.52 (q, J = 3.84, 8.16 Hz, 1H), 3.50 (s, 1H), 3.31 (t, J = 7.28 Hz, 4H), 3.10 (m, 1H), 2.69 (s, 3H), 2.21 (t, J = 7.44 Hz, 3H), 2.08 (m, 2H), 1.77 (s, 1H), 1.51 (m, 9H), 1.38 (s, 2H), 1.32 (m, 4H).

^{13}C-NMR (126 MHz, DMSO-d6): δ = 187.21, 186.92, 174.83, 174.58, 172.66, 172.23, 165.06, 162.77, 154.16, 151.62, 150.53, 141.63, 140.08, 139.56, 135.11, 129.35, 125.21, 121.21, 51.62, 50.98, 46.75, 46.23, 38.71, 38.43, 36.25, 35.73, 35.39, 33.97, 31.23, 29.14, 28.48, 28.46, 26.32, 26.27, 26.19, 25.20, 25.08, 24.49, 14.38, 12.31, 9.11.

HRMS (ESI) calculated for ($[M+H]^+$): m/z = 477.2456; experimental = 477.2439.

Compound **54**

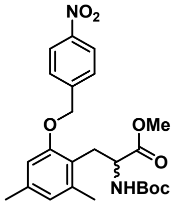

Chemical Formula: $C_{24}H_{30}N_2O_7$
Exact Mass: 458,2053
Molecular Weight: 458,5110

Methyl2-((*t*-butoxycarbonyl)amino)-3-(2,4-dimethyl-6-((4-nitrobenzyl)oxy)phenyl) propanate (chemspace ID: CSC010216371, 500 mg, 1.62 mmol, 1.0 eq) was dissolved in MeOH (10 mL) and dimethoxy propane (396 µL, 3.232 mmol, 2.0 eq) was added, followed by 20% HCl (10 µL). The reaction was stirred at 23 °C for 2 hours. before the solvent was removed *in vacuo*. The resultant residue was dried for two hours under reduced pressure before ACN (10 mL), *para*-nitro benzyl bromide (523.7 mg, 2.42 mmol, 1.5 eq) and K_2CO_3 (446.9 mg, 3.23 mmol, 2.0 eq) were added. The suspension stirred overnight at 23 °C. Then the reaction was filtered and the filter was washed with DCM (100 mL). The solvent was removed *in vacuo* and the residue was washed with ice-cold petrolether. The residue was purified by flash chromatography (C18, 10-100% ACN/H_2O, 220 nm) and product containing fractions were identified by LCMS. The combined fractions were lyophilized to dryness to yield ether **54** (614.71 mg, 1.34 mmol, 83% over two steps) as a yellow oil.

^{1}H-NMR (500 MHz, DMSO-d$_6$): δ = 8.24 (d, J = 8.8 Hz, 2H), 7.76 (d, J = 1.53 Hz, 1H), 7.24 (d, J = 7.84 Hz, 1H), 6.66 (d, 2H), 5.25 (s, 2H), 4.22 (q, J = 7.84, 15.49, 1H), 3.48 (s, 3H), 2.98 (m, 2H), 2.22 (s, 3H), 2.19 (s, 3H), 1.33 (s, 9).

^{13}C-NMR (126 MHz, DMSO-d$_6$): δ = 172.80, 156.15, 155.21, 146.86, 145.38, 137.62, 136.43, 127.73, 123.61, 123.46, 121.07, 110.30, 78.22, 68.17, 53.28, 51.57, 39.52, 28.26, 28.09, 27.65, 21.03, 19.19.

DEPT (126 MHz, DMSO-d$_6$): δ = 127.47, 123.35, 123.20, 110.04, 67.92, 53.03, 51.31, 39.52, 28.00, 27.83, 27.39, 20.77, 18.93.

HRMS (ESI) calculated for ([M+H]$^+$): m/z = 459.2126; experimental = 459.2132.

Compound **55**

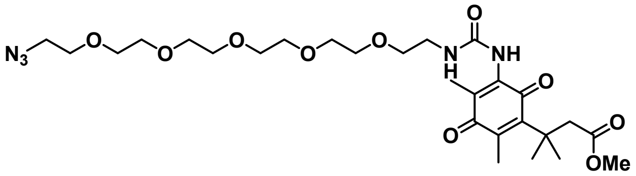

Chemical Formula: $C_{27}H_{43}N_5O_{10}$
Exact Mass: 597,3010
Molecular Weight: 597,6660

51 (50 mg, 0.188 mmol, 1.0 eq) was dissolved in anhydrous toluene (50 mL) and triphosgene (111.85 mg, 0.377 mmol, 2.0 eq) was added at 23 °C. The reaction was heated to 80 °C for 18 h and then the solvent was removed by rotary evaporation. The residue was dried under reduced pressure for 2 hours. Anhydrous DCM (15 mL) was added under argon atmosphere and the solution was cooled to 0 °C. Then N_3-PEG-NH_2 (230.94 mg, 0.754 mmol, 4.0 eq) dissolved in anhydrous DCM (10 mL) and TEA (500 µL) were added dropwise together at 0 °C. The reaction was equilibrated to 23 °C and stirred at this temperature for two hours. The reaction progress was monitored by LCMS and after complete conversion the solvent was removed by rotary evaporation and dissolved in ACN (4 mL), filtered and purified by RP-HPLC (C18, 5-75% ACN/H_2O, 0.1% HCOOH, 220 nm,). The product containing fractions were lyophilized to dryness to yield **55** (285.4 mg, 0.477 mmol, 92%) as an orange oil.

^{1}H-NMR (500 MHz, MeOH-d_4): δ = 3.66 (m, 18H), 3.57 (m, 5H), 3.37 (q, J = 5.08, 9.87 Hz, 4H), 2.94 (s, 2H), 2.17 (s, 3H), 1.88 (s, 3H), 1.46 (s, 6H).

^{13}C-NMR (126 MHz, MeOH-d_4): δ = 187.36, 186.38, 172.86, 149.99, 140.15, 139.91, 127.48, 70.23, 70.22, 70.17, 70.16, 70.12, 69.92, 69.74, 50.50, 50.37, 46.69, 38.31, 28.19, 13.23, 11.12.

Compound **56**

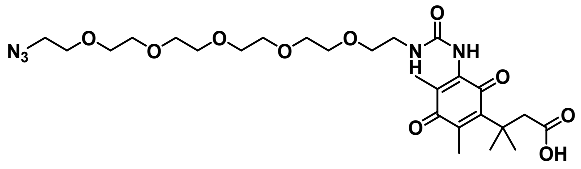

Chemical Formula: $C_{26}H_{41}N_5O_{10}$
Exact Mass: 583,2853
Molecular Weight: 583,6390

The ester **55** (29 mg, 0.048 mmol, 1.0) was dissolved in MeOH (900 µL) and 1M KOH (100 µL) was added. The reaction continued stirring at 24 °C. Upon completion (~1 h), the pH was adjusted to two (color change purple to yellow) by the dropwise addition of concentrated HCl on ice. The MeOH fraction was removed by rotary evaporation at 30 °C, then water (10 mL) and brine (10 mL) were added. The aqueous phase was extracted with DCM (3x20 mL) and the combined organic extracts were dried over Na_2SO_4. The solvent was removed *in vacuo* to yield acid **56** (28.04mg, 0.048 mmol, quant.) as a crude oil that was directly used in the next step.

HRMS (ESI) calculated for ($[M+H]^+$): m/z = 584.2926; experimental = 584.2937.

Compound **57**

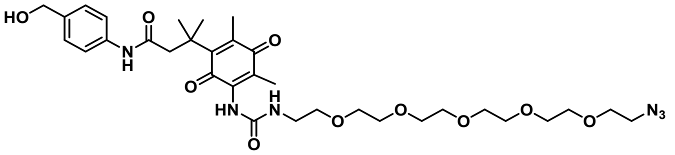

Chemical Formula: $C_{33}H_{48}N_6O_{10}$
Exact Mass: 688,3432
Molecular Weight: 688,7790

The acid **56** (30 mg, 0.051 mmol, 1.0 eq) was dissolved in anhydrous THF (2.5 mL), NMM (200 µL) was added under argon atmosphere and the reaction was cooled to 0 °C, before *iso*-butyl chloroformate (4.98 µL, 0.051 mmol, 1.0 eq) was added and the reaction mixture turned turbid instantly. The mixture was stirred for 15 minutes at 0 °C and subsequently one hour at 24 °C. Then, *para*-amino benzyl alcohol (7.6 mg, 0.062 mmol, 1.2 eq) was added at 0 °C, dissolved in anhydrous THF (1 mL) and basified with NMM (200 µL) before addition. The reaction continued stirring at 0 °C for 15 minutes and then for 1 hour at 24 °C. The reaction was diluted with DCM (100 mL) and washed with 1M HCl (3x100 mL), dried over Na_2SO_4 and evaporated to dryness to yield crude **57** as a yellow oil (27.4 mg, 0.04 mmol, 77%).

HRMS (ESI) calculated for ($[M+H]^+$): m/z = 689.3505; experimental = 689.3508.

Compound **72**

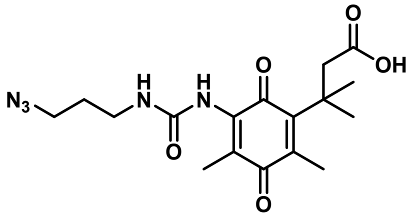

Chemical Formula: $C_{17}H_{23}N_5O_5$
Exact Mass: 377,1699
Molecular Weight: 377,4010

51 (400 mg, 1.5 mmol, 1.0 eq) was dissolved in dry toluene (50 mL) and solid triphosgene (490.2 mg, 1.7 mmol, 1.1 eq) was added at 24 °C. The reaction was heated under argon atmosphere to 80 °C for 18 hours and then evaporated to dryness. The residue was dried under vacuum, dissolved in anhydrous DCM (40 mL) and 3-azido-propane-1-amine (225.7 mg, 2.3 mmol, 1.5 eq) was added as solution in dry DCM (10 mL) together with DIPEA (785 µL, 3.0 eq) at 0 °C. The reaction mixture was stirred 1 hour at 0 °C and then 1 hour at 24 °C. More DCM (200 mL) was added and the organic phase was washed with 1N HCl (2x100 mL), brine (2x100 mL) and dried over anhydrous Na_2SO_4. The solvent was removed *in vacuo* and the residue was dissolved in MeOH (40 mL) and 1 N LiOH (5 mL) was added. The reaction was stirred for 2 hours at ambient temperature and was then acidified with HCl to pH 2. The MeOH was removed by rotary evaporation before NaCl and EA (200 mL), brine (200 mL) were added. The aqueous phase was extracted with EA (2x100 mL), the combined organic extracts were dried over Na_2SO_4 and concentrated to dryness to yield **72** (274.6 mg, 0.73 mmol, 48% over two steps) as a crude orange solid.

^{1}H-NMR (500 MHz, DMSO-d$_6$): δ = 12.08 (s, 1H), 8.17 (s, 1H), 6.78 (t, J = 6.06 Hz, 1H), 3.37 (t, J = 7.06 Hz, 2H), 3.34 (s, 1H), 3.10 (q, J = 6.72, 12.68 Hz, 2H), 2.80 (s, 2H), 2.09 (s, 3H), 1.76 (s, 3H), 1.66 (m, 2H), 1.37 (s, 6H).

DEPT (126 MHz, DMSO-d$_6$): δ = 48.15, 46.65, 28.65, 28.41, 13.82, 12.02.

^{13}C-NMR (126 MHz, DMSO-d$_6$): δ = 186.94, 186.51, 173.21, 153.53, 150.19, 140.73, 139.10, 124.87, 48.39, 46.89, 37.80, 36.79, 28.90, 28.66, 28.38, 14.06, 13.93, 12.27, 11.87.

HRMS (ESI) calculated for ([M+H]$^+$): m/z = 378,1771; experimental = 378.1772.

Gyrase inhibitor constructs

Compound **58**

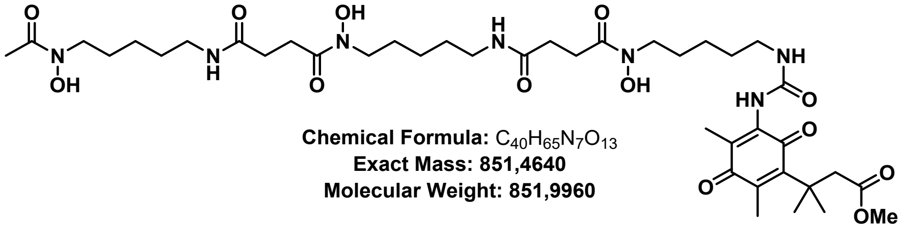

This compound was synthetized according to a modified procedure by Miller et al.[5] Amino TML **51** (160.5 mg, 0.61 mmol, 1.0 eq) was dissolved in dry toluene (12.5 mL) and triphosgene (538.5 mg, 1.82 mmol, 3.0 eq) was added at 25 °C in one portion under argon atmosphere. Then the reaction mixture was heated to reflux overnight, cooled to 25 °C and filtered. The solvent was removed *in vacuo*, the residue was dried under reduced pressure for 30 minutes and then dissolved in anhydrous DMF (1 mL). DFO (339.2 mg, 0.61 mmol, 1 eq) was added in DMF (4.5 mL, solubilize w. heat gun) together with TEA (335 µL, 4.84 mmol, 4.0 eq) dropwise at 0 °C over five minutes. The reaction mixture was stirred at 0 °C for one hour and at 25 °C for two hours. The base and solvent were removed under reduced pressure and the residue was dissolved in MeOH/ACN/H_2O and purified by RP-HPLC (10-50% ACN, 0.1% HCOOH, 220 nm) to yield **58** as a yellow solid (87.4 mg, 0.102 mmol, 18%).

^{1}H-NMR (500 MHz, DMSO-d$_6$): δ = 9.62 (m, 2H), 8.12 (s, 1H), 7.77 (t, J = 5.09 Hz, 2H), 6.62 (t, J = 5.46 Hz, 1H), 3.49 (s, 3H), 3.45 (m, 8H), 3.00 (m, 6H), 2.85 (s, 2H), 2.57 (t, J = 6.31 Hz, 3H),2.27 (m, 5H), 2.11 (s, 1H), 2.08 (s, 3H)), 1.96 (s, 1H), 1.77 (s, 3H), 1.54 (s, 2H), 1.49 (m, 6H), 1.38 (m, 10H), 1.21 (m, 6H).

^{13}C-NMR (126 MHz, DMSO-d$_6$): δ = 186.76, 186.55, 171.98, 171.81, 171.48, 171.34, 170.16, 154.57, 153.44, 149.94, 149.86, 141.18, 139.19, 135.23, 127.78, 126.07, 124.53, 117.66, 106.25, 51.14, 50.89, 47.10, 46.80, 46.44, 46.33, 38.43, 38.05, 37.97, 30.24, 29.92, 29.20, 28.81, 28.68, 27.57, 26.03, 23.49, 20.34, 15.24, 13.94, 10.99.

Compound **12**

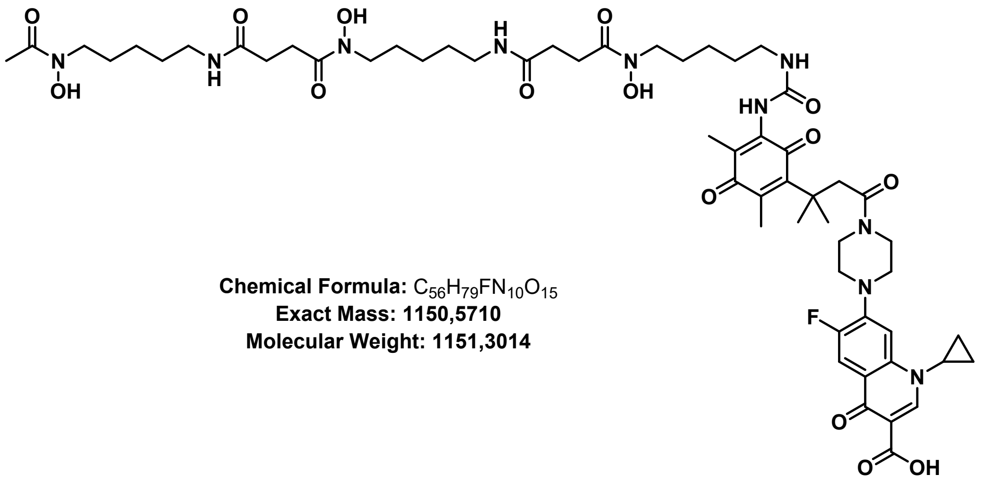

This compound was previously synthetized by Miller et al.[5] Ester **58** (11.34 mg, 0.013 mmol, 1.0 eq) was dissolved in MeOH (1 mL). A 5 N KOH solution was added (100 µL) at 25 °C. The color changed from yellow to a dark red and the reaction stirred 3 h at ambient temperature. After completion, the pH was adjusted to 2 with 3 M HCl and the solution was loaded on a milliQ water equilibrated C18-SiO_2 pad. The salts were removed with milliQ water (5 mL) while the compound retained and was then eluted with 70% ACN/H_2O. The yellow solution was frozen in liquid N_2 and lyophilized to yield crude acid **58a**. Acid **58a** was dissolved in DMF (5 mL), cooled to 0 °C before EDCI*HCl (14.5 mg, 0.076 mmol, 5.7 eq) and HOBt (10.25 mg, 0.076 mmol, 5.7 eq) were added. The yellowish solution was stirred at 0 °C for 5 minutes, before ciprofloxacin (6.16 mg, 0.016 mmol, 1.2 eq) and TEA (7.87 µL, 0.106 mmol, 8.0 eq), followed by DMAP (0.33 mg, 0.003 mmol, 0.2 eq), were added. The mixture was warmed to 25 °C and stirred for 16 h. The next morning, the solvent was removed under reduced pressure and the residue was taken up in 40% ACN in milliQ H_2O, filtered and injected into the HPLC (10-70% ACN/H_2O, 0.1% HCOOH, 220 nm). The product containing fractions were identified by LCMS and lyophilized to yield **12** as a yellow powder overnight (5.4 mg, 0.005 mmol, 35%).

^{1}H-NMR (700 MHz, DMSO-d_6): δ = 15.20 (s, 1H), 9.60 (m, 2H), 8.67 (s, 1H), 8.00 (s, 1H), 7.95 (d, J = 14.55 Hz, 1H), 7.78 (m, 2H), 7.54 (d, J = 10.91 Hz, 1H), 6.62 (t, J = 5.61 Hz, 1H), 3.80 (m, 1H), 3.70 (m, 2H), 3.60 (m, 2H), 3.45 (m, 4H), 3.39 (m, 2H), 3.36 (m, 2H), 3.29 (m, 3H), 2.99 (m, 8H), 2.57 (q, J = 8.13, 15.58 Hz, 4H), 2.25 (s, 4H), 2.07 (s, 3H), 1.96 (s, 3H), 1.76 (s, 3H), 1.49 (m, 4H), 1.41 (s, 6H), 1.35 (m, 8H), 1.19 (m, 5H), 1.14 (m, 1H)

^{13}**C-NMR** (176 MHz, DMSO-d_6): δ = 186.94, 186.62, 176.36, 171.93, 171.27, 169.74, 165.91, 153.37, 152.21, 148.09, 141.09, 139.15, 136.65, 106.75, 50.49, 47.05, 46.75, 45.05, 44.73, 40.56, 40.00, 38.39, 38.09, 35.86, 33.48, 29.86, 29.19, 28.79, 28.70, 27.97, 27.53, 26.00, 25.69, 24.00, 23.47, 23.33, 20.32, 13.85, 11.98, 7.57.

DEPT (176 MHz, DMSO-d_6): δ = 147.85, 110.88, 110.75, 106.13, 50.25, 49.22, 48.78, 46.80, 46.51, 44.81, 44.49, 40.32, 39.52, 39.13, 38.16, 35.63, 33.24, 29.62, 28.95, 28.55, 28.46, 27.73, 27.29, 25.76, 25.45, 23.76, 23.23, 23.09, 20.08, 13.61, 11.74, 7.33, 0.15.

^{19}**F-NMR** (471 MHz, DMSO-d_6): δ = -121.63.

HRMS (ESI) calculated for ($[M+H]^+$): m/z = 1151.5783; experimental = 1151.5791.

Compound **59**

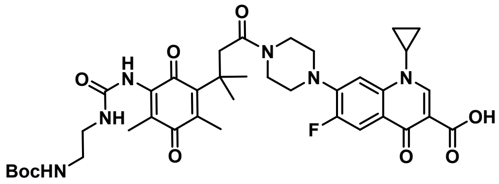

Chemical Formula: $C_{38}H_{47}FN_6O_9$
Exact Mass: 750,3389
Molecular Weight: 750,8254

This compound was synthetized with modified conditions from Miller et al.[5] Ester **52** (20 mg, 0.044 mmol, 1.0 eq) was dissolved in MeOH (1 mL) and 1M NaOH (1 mL) was added at 23 °C. The reaction mixture was stirred for two hours, then acidified with 1M HCl to pH 2 and quickly extracted with DCM (2 x 25 mL). The combined organic phases were dried over Na_2SO_4 and concentrated to dryness to yield acid **52a** as a crude, yellow oil. This oil was dried under reduced pressure for 1 hour, before DCM / DMF (10 and 1 mL) were added. This was followed by HOBt (13.8 mg, 0.089 mmol, 2.0 eq) and EDCI*HCl (12.1 mg, 0.089 mmol, 2.0 eq) addition at 0°C under argon atmosphere. The reaction mixture was stirred five minutes at 0 °C before ciprofloxacin (22 mg, 0.066 mmol, 1.5 eq in 1 mL DMF) and DIPEA (30 µL) were added at 0 °C. The ice bath was removed and the reaction continued stirring overnight at 23 °C. The solvent was removed by rotary evaporation and the residue was dissolved in DCM (100 mL) and was washed with 1M HCl/brine (1:1, 3x50 mL), brine (3x50 mL). After drying over Na_2SO_4 the solvent was removed *in vacuo* and the residue was purified by RP-HPLC (C18, 10-70% ACN/H_2O, 0.1 % HCOOH, 220 nm). Product containing fractions were identified by LCMS and

lyophilized to yield intermediate **59** (19.6 mg, 0.026 mmol, 59% over two steps) as a yellow oil.

¹H-NMR (700 MHz, DMSO-d6): δ = 15.20 (s, 1H), 8.67 (s, 1H), 8.09 (s, 1H), 7.93 (d, J = 14.6 Hz, 2H), 7.55 (d, J = 8.73 Hz, 2H), 6.76 (t, J = 5.5 Hz, 1H), 6.67 (t, J = 5.5 Hz, 1H), 4.08 (q, J = 5.22, 10.7 Hz, 1H), 3.81 (m, 1H), 3.70, 3.61 (m, 2H), 3.36 (m, 2H), 3.29 (m, 6H), 3.16 (d, J = 4.95 Hz, 4H), 3.03 (q, J = 6.02, 12.23 Hz, 2H), 2.99 (s, 2H), 2.91 (q, J = 6.02, 11.96 Hz, 2H), 2.07 (s, 3H), 1.91 (s, 1H), 1.77 (s, 3H), 1.41 (s, 6H), 1.34 (s, 9H), 1.31 (m, 2H), 1.19 (m, 2H).

¹³C-NMR (176 MHz, DMSO-d6): δ = 186.98, 186.54, 176.37, 169.70, 165.90, 153.60, 152.23, 148.09, 144.83, 140.97, 139.15, 136.69, 106.76, 106.40, 77.62, 48.57, 44.99, 44.74, 38.13, 35.86, 35.76, 28.69, 28.17, 21.03, 13.85, 11.96, 7.57.

HRMS (ESI) calculated for ([M+H]⁺): m/z = 751,3461; experimental =751.3454.

Compound **8**

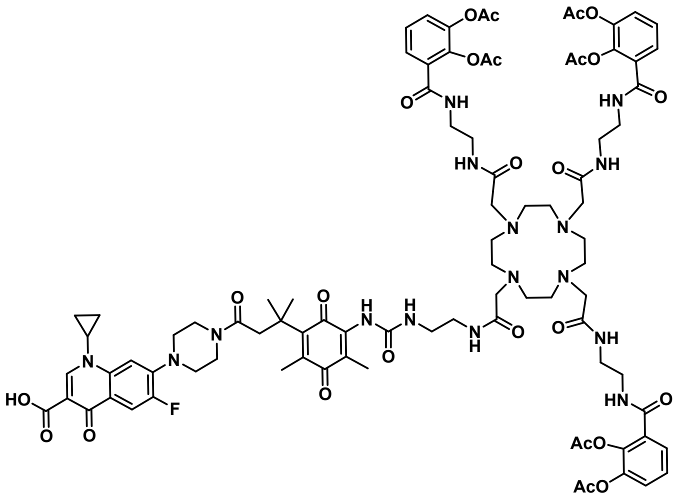

Chemical Formula: $C_{88}H_{107}FN_{16}O_{26}$
Exact Mass: 1822,7526
Molecular Weight: 1823,9084

N-Boc amine **59** (10 mg, 0.013 mmol, 1.0 eq) was dissolved in anhydrous DCM (0.75 mL) and TFA (0.25 mL) was added at 0 °C. The yellow solution stirred for 2 hours at 24 °C, and was then concentrated to dryness to yield **59a**. The orange residue was dried under reduced pressure for 2 hours. Meanwhile acid **7** (23.8 mg, 0.02 mmol, 1.5 eq) was dissolved in anhydrous THF (500 µL), and NMM (30 µL) was added under argon atmosphere. At 0 °C *iso*-butyl chloroformate (1.4 µL, 0.020 mmol, 1.5 eq) was added and the reaction went turbid instantly. The mixture was stirred ten minutes at 0 °C and 1 h at 24 °C. Then amine **59a** was added dropwise at 0 °C, in anhydrous THF (500 µL) and together with NMM (30 µL). The reaction continued stirring at 0 °C for 5 minutes and one hour at 24 °C. Then AcOH (200 µL) was added, the solvent was removed *in vacuo* at 30 °C and the residue was purified by RP-

HPLC (C18, 10-70% ACN/H_2O, 1% AcOH, 220 nm). Product containing fractions were combined and lyophilized to yield conjugate **8** as a slightly yellow powder (19.06 mg, 0.01 mmol, 79%).

^{1}H-NMR (500 MHz, DMSO-d_6+1% AcOH-d4): δ = 8.67 (s, 3H), 8.26 (d, J = 1.69 Hz, 2H), 8.12 (d, J = 13.17 Hz, 2H), 7.83 (m, 2H), 7.55 (m, 3H), 7.58 (m, 1H), 6.69 (m, 1H), 3.81 (m, 5H), 3.70 (m, 7H), 3.61 (m, 6H), 3.48 (m, 2H), 3.41 (m, 2H), 3.35 (m, 6H), 3.27 (m, 6H), 3.18 (m, 2H), 3.04 (m, 15H), 2.73 (m, 6H), 2.07 (s, 9H), 1.79 (s, 2H), 1.41 (s, 9H), 1.35 (s, 9H), 1.41 (s, 6H), 1.32 (m, 4H), 1.25 (m, 2H), 1.18 (m, 4H), 1.10 (m, 2H).

^{13}C-NMR (176 MHz, DMSO-d_6+1% AcOH-d4): δ = 187.02, 186.53, 176.37, 169.75, 169.32, 169.23, 165.84, 153.62, 153.49, 152.20, 148.15, 144.89, 140.92, 140.76, 139.15, 138.49, 136.76, 124.76, 118.82, 116.68, 111.14, 111.01, 106.71, 106.44, 49.87, 49.53, 49.02, 44.99, 44.78, 40.57, 38.80, 38.68, 38.15, 35.87, 34.28, 28.70, 22.58, 22.53, 21.00, 13.89, 12.08, 12.05, 7.59.

HRMS (ESI) calculated for ($[M+2H]^{2+}$): m/z = 953.4101; experimental =953.4115.

Compound **9**

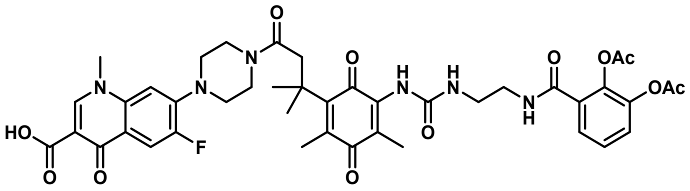

Chemical Formula: $C_{42}H_{45}FN_6O_{12}$
Exact Mass: 844,3079
Molecular Weight: 844,8504

2,3-diacethoxy benzoic acid **38** (2.75 mg, 0.012 mmol, 1.5 eq) was dissolved in anhydrous THF (500 µL) and anhydrous NMM (10 µL) was added under argon atmosphere at 0 °C. At this temperature *iso*-butyl chloroformate (1.9 µL, 0.012 mmol, 1.5 eq) was added and the mixture was stirred at 0 ° for five minutes and at 24 °C for 30 minutes. Then, the amine **59a** (preparation see above, 5.0 mg, 0.008 mmol, 1.0 eq) was added in THF (500 µL) with NMM (10 µL) dropwise at 0 °C. The reaction stirred for 30 minutes at 24 °C and then AcOH (200 µL) was added. The reaction was concentrated and the residue taken up in ACN, filtered and purified by RP-HPLC (5-80% ACN/H2O, 1% AcOH, 220 nm). Product containing fractions were lyophilized to yield amide **9** (5.565 mg, 0.006 mmol, 83%) as a yellow powder.

^{1}H-NMR (500 MHz, DMSO-d6, 1% AcOH-d4): δ = 8.67 (m, 1H), 7.92 (m, 1H), 7.54 (m, 1H), 7.44 (m, 1H), 7.34 (m, 1H), 3.81 (m, 2H), 3.69 (m, 3H), 3.61 (m, 3H), 3.34 (m, 6H), 3.27 (m, 3H), 3.18 (m, 3H), 3.05 (t, J = 5.76 Hz, 1H), 2.99 (m, 3H), 2.27 (s, 3H), 2.20 (s, 1H), 2.07 (s, 3H), 1.91 (s, 1H), 1.77 (s, 3H), 1.41 (s, 6H), 1.37 (m, 1H), 1.30 (m, 3H), 1.23 (m, 1H), 1.17 (m, 3H), 1.09 (m, 1H), 0.92 (d, J = 7.16 Hz, 2H), 0.84 (d, J = 6.80 Hz, 3H).

^{13}C-NMR (176 MHz, DMSO-d6+1% AcOH-d4): δ = 171.94, 168.25, 168.18, 164.98, 164.92, 164.43, 151.74, 143.04, 142.74, 142.04, 139.96, 129.67, 128.62, 128.46, 128.16, 126.65, 126.28, 126.18, 126.04, 125.41, 74.56, 74.53, 74.49, 69.71, 69.55, 69.52, 69.49, 68.65, 38.79, 38.08, 35.84, 28.70, 27.27, 27.14, 18.47, 18.37, 13.85, 7.57.

HRMS (ESI) calculated for ($[M+2H]^{2+}$): m/z = 845.3152; experimental = 845.3158.

Compound **10**

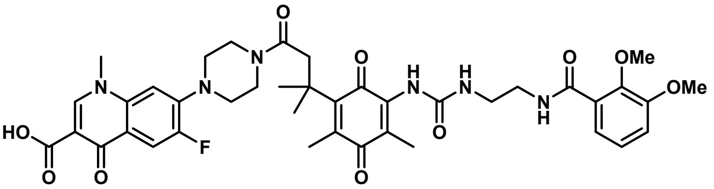

Chemical Formula: $C_{40}H_{45}FN_6O_{10}$
Exact Mass: 788,3181
Molecular Weight: 788,8304

2,3-dimethoxy benzoic acid (2.52 mg, 0.014 mmol, 1.5 eq) was dissolved in anhydrous THF (500 µL) and anhydrous NMM (10 µL) was added under argon atmosphere at 0 °C. At this temperature *iso*-butyl chloroformate (1.7 µL, 0.014 mmol, 1.5 eq) was added and the reaction continued stirring at 0 ° for five minutes. The reaction stirred for 30 minutes at 24 °C. Then the amine **59a** (6.0 mg, 0.009 mmol, 1.0 eq) was added in THF (500 µL) with NMM (10 µL) dropwise at 0 °C. The reaction stirred for 30 minutes at 24 °C and was then concentrated *in vacuo*. The residue was taken up in ACN, filtered and purified by RP-HPLC (5-80% ACN/H2O, 1% AcOH, 220 nm). Product containing fractions were lyophilized to yield amide **10** (6.703 mg, 0.008 mmol, 89%) as a yellow powder.

^{1}H-NMR (500 MHz, DMSO-d_6): δ = 15.20 (s, 1H), 8.64 (m, 1H), 8.17 (m, 2H), 7.91 (d, J = 15.78 Hz, 1H), 7.53 (m, 1H), 7.11 (m, 4H), 6.73 (m, 1H), 3.80 (m, 3H), 3.72 (m, 2H), 3.69 (m, 1H), 3.60 (m, 1H), 3.41 (m, 1H), 3.35 (m, 2H), 3.32 (s, 6H), 3.28 (m, 3H), 3.20 (m, 2H), 2.98 (bs, 1H), 2.07 (s, 2H), 1.76 (s, 2H), 1.41 (s, 6H), 1.30 (q, J = 6.58, 13.67 Hz, 2H), 1.23 (m, 1H), 1.17 (m, 2H).

^{13}C-NMR (126 MHz, DMSO-d_6): δ = 186.96, 186.53, 169.71, 165.91, 165.43, 153.72, 152.45, 152.36, 148.01, 146.35, 141.01, 139.11, 136.58, 129.25, 123.88, 120.71, 114.77, 110.91, 106.74, 106.34, 60.88, 60.85, 55.89, 45.02, 44.74, 40.54, 38.14, 35.83, 28.68, 13.84, 11.90, 7.56.

HRMS (ESI) calculated for ($[M+H]^+$): m/z = 789.3254; experimental = 789.3256.

Compound **11**

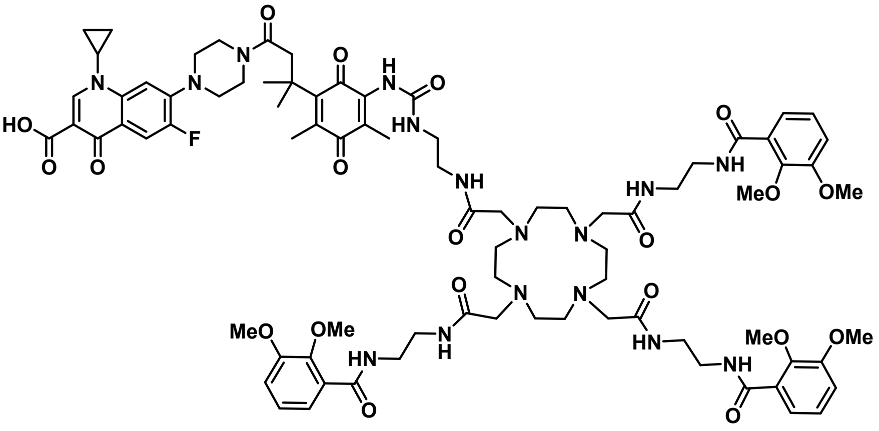

Chemical Formula: $C_{82}H_{107}FN_{16}O_{20}$
Exact Mass: 1654,7832
Molecular Weight: 1655,8484

44 was dissolved in DCM/DMF (5/0.25 mL) and HATU (14.87 mg, 0.039 mmol, 2.0 eq) was added in one portion. After 5 minutes, the amine **59a** (preparation see above, 15.26 mg, 0.023 mmol, 1.2 eq) was added in DCM/DMF (5/0.25 µL) together with DIPEA (100 µL) and the yellow solution stirred overnight at ambient temperature. The solvent was removed by rotary evaporation and the residue was purified by RP-HPLC (C18, 5-85% ACN/H_2O, 0.1% HCOOH, 220 nm) and product containing fractions were lyophilized to yield **11** (21.6 mg, 0.014 mmol, 71%) as a yellow solid.

^{1}H-NMR (700 MHz, DMSO-d6): δ = 15.20 (s, 1H), 8.66 (m, 2H), 8.21 (t, J = 5.73 Hz, 1H), 8.12 (m, 1H), 7.91 (d, J = 13.27 Hz, 2H), 7.52 (d, J = 7.21 Hz, 2H), 7.12 (m, 4H), 7.07 (m, 2H), 6.74 (m, 1H), 3.80 (m, 9H), 3.72 (s, 5H), 3.69 (m, 4H), 3.60 (m, 4H), 3.35 (m, 20H), 3.27 (m, 8H), 3.20 (q, J = 5.57, 11.79 Hz, 4H), 2.98 (bs, 4H), 2.07 (s, 6H), 1.91 (s, 2H), 1.76 (s, 6H), 1.41 (s, 12H), 1.30 (q, J = 7.53, 15.23 Hz, 4H), 1.17 (m, 4H).

^{13}C-NMR (176 MHz, DMSO-d6): δ = 186.97, 186.54, 176.35, 169.72, 165.91, 165.44, 153.73, 153.59, 152.46, 152.36, 152.18, 148.03, 146.36, 144.82, 141.02, 139.12, 136.60, 129.26, 124.74, 123.89, 120.72, 114.78, 111.09, 110.96, 106.75, 106.37, 60.85, 55.90, 49.50, 48.99, 45.02, 44.75, 40.54, 40.02, 39.52, 38.14, 35.83, 28.69, 21.03, 13.85, 11.90, 7.56.

HRMS (ESI) calculated for ($[M+2H]^{2+}$): m/z = 823.8988; experimental = 823.9001.

Compound **13**

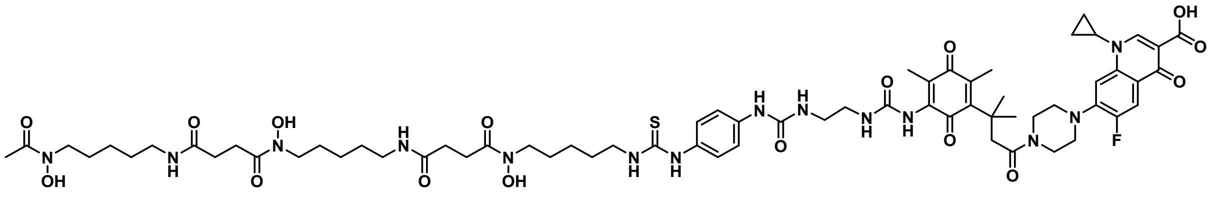

Chemical Formula: $C_{66}H_{91}FN_{14}O_{16}S$
Exact Mass: 1386,6442
Molecular Weight: 1387,5944

Boc-protected amine **59** (14.4 mg, 0.019 mmol, 1.2 eq) was dissolved in DCM (0.75 mL) and TFA (0.25 mL) was added at 0 °C. The reaction mixture was stirred for two hours at 24 °C and upon full conversion as per LCMS the solution was concentrated to dryness to yield **59a**. After drying for several hours under reduced pressure, the residue was dissolved in anhydrous DMF (500 µL) and isothiocyanate **46** (12.0 mg, 0.016 mmol, 1.0 eq) was added. Then TEA (50 µL) was added and the mixture was stirred at 24 °C overnight. The base was removed by rotary evaporation, the solution was filtered and purified by RP-HPLC (C18, 10-80% ACN/H_2O, 0.1% HCOOH, 220 nm). The product containing fractions were identified by LCMS and lyophilized to yield pure title compound **13** as a beige solid (19.56 mg, 0.014 mmol, 73% over two steps).

^{1}H-NMR (700 MHz, DMSO-d6): δ = 9.62 (m, 3H), 7.90 (m, 1H), 7.77 (m, 2H), 7.54 (d, J = 9.52 Hz, 2H), 7.37 (d, J = 9.18 Hz, 2H), 3.47 (m, 8H), 3.00 (q, J = 6.85, 13.52 H, 2.57 (q, J = 7.68, 13.02 Hz, 4H), 2.27 (q, J = 6.34, 12.52 Hz, 4H), 1.96 (s, 3H), 1.52 (m, 4H), 1.49 (m, 4H), 1.38 (m, 4H), 1.26 (m, 2H), 1.21 (m, 4H).

^{13}C-NMR (176 MHz, DMSO-d6): δ = 187.01, 186.51, 176.35, 169.73, 169.30, 169.22, 165.82, 153.60, 153.48, 152.18, 148.13, 144.87, 140.90, 140.74, 139.13, 138.47, 136.74, 124.74, 118.80, 116.67, 111.12, 110.99, 106.69, 106.42, 49.86, 49.51, 49.01, 44.97, 44.76, 40.55, 38.78, 38.66, 38.14, 35.85, 34.26, 28.68, 22.56, 22.51, 20.98, 13.87, 12.07, 12.03, 7.57.

DEPT (176 MHz, DMSO-d6): δ = 187.49, 187.00, 176.83, 172.44, 170.21, 169.78, 169.70, 166.30, 165.56, 154.08, 154.02, 153.96, 152.64, 148.61, 145.29, 141.36, 141.22, 139.61, 137.22, 125.13, 119.30, 111.59, 111.40, 107.19, 106.93, 106.89, 49.99, 49.50, 45.45, 45.25, 41.04, 38.62, 36.33, 34.75, 29.16, 23.04, 22.99, 21.47, 14.35, 12.54, 12.51, 8.05.

HRMS (ESI) calculated for ([M+H]$^+$): m/z = 1404.6780; experimental = 1404.6788.

Compound **60**

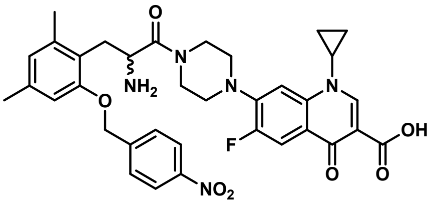

Chemical Formula: $C_{35}H_{36}FN_5O_7$
Exact Mass: 657,2599
Molecular Weight: 657,6994

Ester **54** (65 mg, 0.142 mmol, 1.0 eq) was dissolved in MeOH (1 mL) and 1M NaOH (1 mL) was added at 23 °C. The reaction mixture was stirred for 2 hours at that temperature prior to being acidified with 1M HCl to pH 2 and then quickly extracted with DCM (2x25 mL). The combined organic phases were dried over Na_2SO_4 and concentrated to dryness to yield acid **54a** as a yellow, crude oil. This oil was dried for one hour under reduced pressure. Then DCM/DMF (10 and 1 mL) were added, followed by HOBt (38.60 mg, 0.284 mmol, 2.0 eq) and EDCI*HCl (44.01 mg, 0.284 mmol, 2.0 eq) at 0°C under argon atmosphere. The reaction mixture was stirred for five minutes at 0 °C before ciprofloxacin (22 mg, 0.066 mmol, 1.5 eq in 1 mL DMF) and DIPEA (30 µL) were added. The ice bath was removed and the reaction agitation continued overnight at 23 °C. The solvent was removed by rotary evaporation and the residue was dissolved in DCM (100 mL) and was washed with brine (3x 50 mL). After drying over Na_2SO_4 the solvent was removed *in vacuo*, the residue was purified by RP-HPLC (C18, 10-70% ACN/H_2O, 220 nm)and product containing fractions were combined to yield pure **60a** (69.9 mg, 0.092, 65%). Then TFA/DCM (25%, 0.25:0.75 mL) was added at 0 °C and the reaction continued stirring at 23 °C for two hours. Then the solvent was removed by rotary evaporation and the residue was purified by RP-HPLC. Product containing fractions were

identified by LCMS and lyophilized to yield amine **60** (60.7 mg, 0.092 mmol, 65% over three steps) as a beige solid.

60a

^{1}H-NMR (700 MHz, DMSO-d$_6$): δ = 15.17 (s, 1H), 8.68 (s, 1H), 8.12 (s, 1H), 7.94 (d, J = 11.78 Hz, 1H), 7.61 (d, J= 6.34 Hz, 1H), 3.83 (m, 1H), 3.61 (m, 4H), 3.35 (t, J = 5.25 Hz, 2H), 3.30 (t, J = 5.25 Hz, 1H), 1.32 (m, 2H)), 1.19 (m, 2H).

^{13}C-NMR (176 MHz, DMSO-d$_6$): δ = 176.38, 165.87, 161.01, 153.71, 152.30, 148.12, 144.96, 144.90, 139.10, 119.05, 119.00, 111.09, 110.96, 107.04, 106.78, 50.20, 50.18, 49.09, 49.07, 44.39, 35.89, 7.57.

HRMS (ESI) calculated for ([M+H]$^+$): m/z = 658.2672; experimental = 658.2673.

Compound **14**

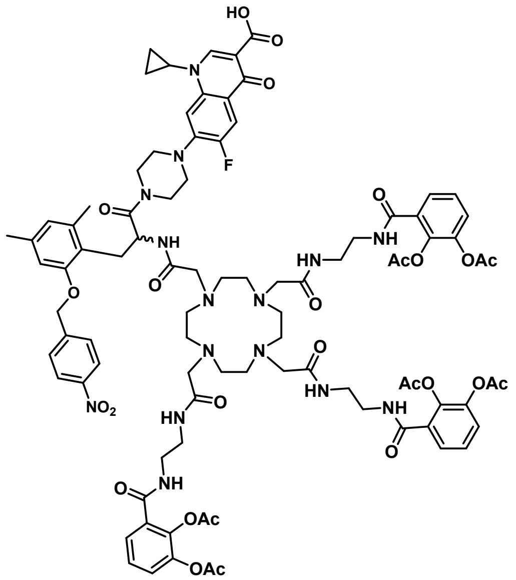

Chemical Formula: $C_{90}H_{104}FN_{15}O_{26}$
Exact Mass: 1829,7261
Molecular Weight: 1830,8994

Acid **7** (10 mg, 0.008 mmol, 1.0 eq) was dissolved in anhydrous THF (1.0 mL), NMM (30 µL) and *iso*-butyl chloroformate (0.81 µL, 0.008, 1.0 eq) was added at 0 °C. The solution stirred 0 °C for ten minutes and 50 minutes at 24 °C. The amine **60** (6.62 mg, 0.01 mmol, 1.2 eq) was dissolved in anhydrous THF (1.0 mL), NMM (30 µL) was added under argon atmosphere at 0 °C. The reaction stirred ten minutes at 0 °C and then 1 h at 24 °C. The reaction continued stirring at 0 °C for five minutes and then for one hour at 24 °C. Then AcOH (200 µL) was added, the solvent was removed *in vacuo* at 30 °C and the residue was purified by RP-HPLC (C18,

10-70% ACN/H_2O, 1% AcOH, 220 nm). Product containing fractions were combined and lyophilized to yield the conjugate **14** as a slightly yellow powder (13.4 mg, 0.007 mmol, 87%).

^{1}H-NMR (700 MHz, DMSO-d6+AcOH-d4): δ = 8.67 (s, 3H), 8.10 (d, J = 15.45 Hz, 2H), 7.92 (d, J = 13.18 Hz, 3H), 7.83 (m, 2H), 7.55 (m, 3H), 7.55 (m, 1H), 6.69 (m, 1H), 3.81 (m, 5H), 3.70 (m, 6H), 3.61 (m, 6H), 3.48 (m, 2H), 3.41 (m, 2H), 3.35 (m, 6H), 3.27 (m, 6H), 3.18 (m, 2H), 3.04 (m, 12H), 2.99 (bs, 6H), 2.07 (s, 9H), 1.91 (s, 2H), 1.77 (s, 9H), 1.75 (s, 9H), 1.41 (s, 6H), 1.32 (m, 4H), 1.25 (m, 2H), 1.18 (m, 4H), 1.10 (m, 2H).

^{13}C-NMR (176 MHz, DMSO-d6+AcOH-d4): δ = 187.02, 186.53, 176.37, 169.75, 169.32, 169.23, 165.84, 153.62, 153.49, 152.20, 148.15, 144.89, 140.92, 140.76, 139.15, 138.49, 136.76, 124.76, 118.82, 116.68, 111.14, 111.01, 106.71, 106.44, 49.87, 49.53, 49.02, 44.99, 44.78, 40.57, 39.52, 38.80, 38.68, 38.15, 35.87, 34.28, 28.70, 22.58, 22.53, 21.00, 13.89, 12.08, 12.05, 7.59.

DEPT (176 MHz, DMSO-d6): δ = 187.03, 186.53, 176.37, 171.97, 169.75, 169.32, 169.23, 165.83, 165.09, 153.62, 153.55, 153.49, 152.18, 148.15, 144.83, 140.90, 140.76, 139.15, 136.75, 124.67, 118.84, 111.12, 110.94, 106.73, 106.47, 106.42, 49.53, 49.04, 44.99, 44.78, 40.57, 38.15, 35.87, 34.28, 28.70, 22.58, 22.53, 21.00, 13.89, 12.08, 12.05, 7.58.

HRMS (ESI) calculated for ($[M+2H]^{2+}$): m/z = 915.8703; experimental = 915.8707.

Compound **15**

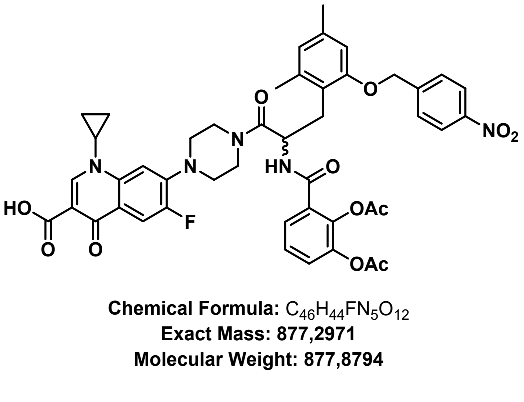

Chemical Formula: $C_{46}H_{44}FN_5O_{12}$
Exact Mass: 877,2971
Molecular Weight: 877,8794

Acid **38** (4.2 mg, 0.018 mmol, 2.0 eq) was dissolved in anhydrous THF (500 µL) and anhydrous NMM (30 µL) was added under argon atmosphere at 0 °C. At this temperature *iso*-butyl chloroformate (3 µL, 0.018 mmol, 2.0 eq) was added and the reaction continued stirring at 0 ° for five minutes and for 30 minutes at 24 °C. Then amine **60** (5.8 mg, 0.009 mmol, 1.0 eq) was

added in THF (500 µL) with NMM (30 µL) dropwise at 0 °C. The reaction stirred for 30 minutes at 24 °C was then concentrated to dryness. The residue was taken up in ACN and purified by RP-HPLC (10-80% ACN/H_2O, 1% AcOH, 220 nm) and product containing fractions were lyophilized to yield amide **15** (4.9 mg, 0.006 mmol, 69%) as a beige powder.

^{1}H-NMR (500 MHz, ACN-d_3): δ = 15.11 (s, 1H), 8.71 (s, 1H), 8.24 (d, J = 8.69 Hz, 2H), 7.94 (d, J = 13.11 Hz, 1H), 7.81 (d, J = 8-69 Hz, 2H), 7.56 (q, J = 2.69, 6.32 Hz, 1H), 7.51 (d, J = 7.90 Hz, 1H), 7.42 (d, J = 7.27 Hz, 1H), 7.35 (m, 2H), 6.69 (d, J = 21.49 Hz, 2H), 5.43 (q, J = 8.21, 15.48 Hz, 1H), 5.22 (d, J = 2.69 Hz, 2H), 3.66 (m, 3H), 3.49 (m, 1H), 3.18 (m, 6H), 2.57 (m, 1H), 2.31 (s, 3H), 2.27 (d, 6H), 2.20 (s, 3H), 1.39 (s, 2H), 1.33 (m, 2H), 1.27 (m, 1H), 1.13 (m, 1).

^{19}F-NMR (471 MHz, ACN-d_3): δ = -123.29.

^{13}C-NMR (176 MHz, ACN-d_3): δ = 178.29, 171.32, 169.57, 169.42, 167.52, 164.23, 158.06, 155.27, 149.32, 148.64, 146.24, 146.14, 144.29, 141.32, 140.57, 139.85, 138.75, 130.84, 129.31, 127.95, 127.73, 127.25, 126.46, 125.06, 124.63, 121.58, 112.52, 112.39, 111.38, 107.42, 69.88, 50.46, 50.22, 49.26, 46.02, 42.50, 36.74, 35.10, 31.11, 30.70, 21.51, 20.89, 20.82, 20.00, 8.73, 8.69.

HRMS (ESI) calculated for ($[M+H]^+$): m/z = 878.3044; experimental = 878.3049.

Compound **16**

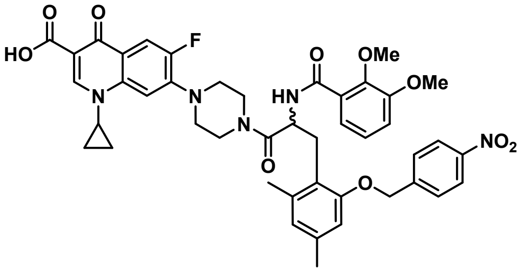

Chemical Formula: $C_{44}H_{44}FN_5O_{10}$
Exact Mass: 821,3072
Molecular Weight: 821,8594

2,3-dimethoxy benzoic acid (3.3 mg, 0.018 mmol, 2.0 eq) was dissolved in anhydrous THF (500 µL) and anhydrous NMM (30 µL) was added under argon atmosphere at 0 °C. At this

temperature *iso*-butyl chloroformate (1.8 µL, 0.018 mmol, 2.0 eq) was added and the reaction was stirred at 0 ° for five minutes and for 30 minutes at 24 °C. Then amine **60** (6.0 mg, 0.009 mmol, 1.0 eq) was added in THF (500 µL) together with NMM (30 µL) dropwise at 0 °C. The reaction mixture was stirred for 30 minutes at 24 °C and was then concentrated to dryness. The residue was taken up in ACN and purified by RP-HPLC (10-80% ACN/H_2O, 0.1% HCOOH, 220 nm) and product containing fractions were lyophilized to yield amide **16** (6 mg, 0.007 mmol, 81%) as a white powder.

^{1}H-NMR (700 MHz, ACN-d_3): δ = 15.11 (s, 1H), 8.72 (s, 1H), 8.24 (m, 2H), 7.93 (m, 1H), 7.81 (m,4H), 7.46 (d, J = 8.63 Hz, 1H), 7.41 (m, 3H), 6.69 (m, 2H), 5.88 (d, J = 8.63 Hz, 1H), 5.26 (m, 5H), 5.00 (m, 1H), 3.78 (s, 1H), 3.71 (m, 4H), 3.63 (m, 2H), 3.24 (m, 3H), 3.05 (m, 5H), 2.35 (s, 3H), 2.29 (s, 3H), 2.19 (s, 5H), 1.35 (m, 2H), 1.34 (m, 2H), 1.27 (m, 2H), 1.14 (m, 1H), 1.13 (m, 1H), 0.88 (m, 6H).

^{13}C-NMR (176 MHz, MeOH-d_4): δ = 185.83, 179.59, 175.37, 173.27, 166.09, 161.99, 156.46, 154.67, 148.59, 147.62, 137.85, 137.18, 136.51, 133.61, 132.99, 132.90, 130.73, 125.13, 119.83, 77.90, 70.68, 65.50, 49.00, 45.34, 39.25, 30.50, 28.91, 17.11, 17.06.

HRMS (ESI) calculated for ($[M+H]^+$): m/z = 822.3145; experimental = 822.3122.

Compound **17**

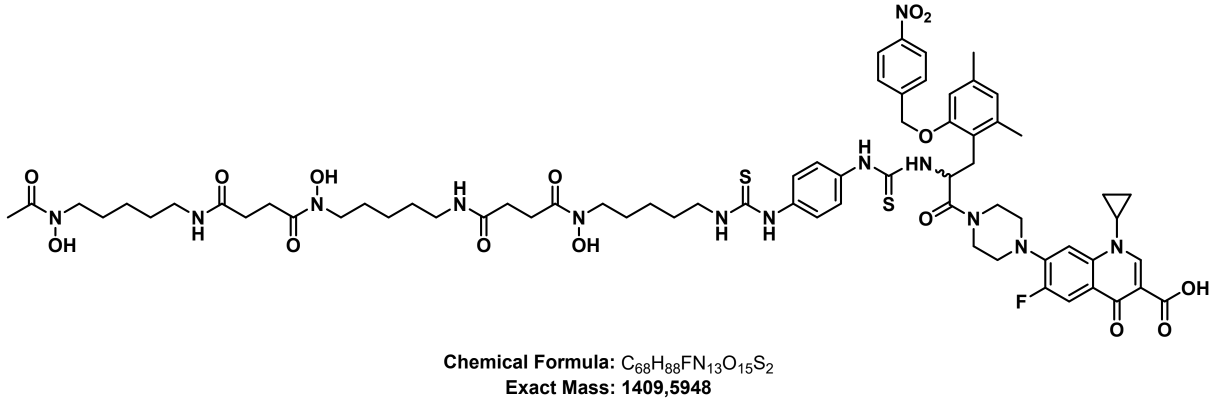

Chemical Formula: $C_{68}H_{88}FN_{13}O_{15}S_2$
Exact Mass: 1409,5948
Molecular Weight: 1410,6464

Amine **60** (20.96 mg, 0.032 mmol, 1.2 eq) was dissolved in DMF (0.5 mL) and TEA (50 µL) was added. The isothiocyanate **46** (20.0 mg, 0.027 mmol, 1.0 eq) was added and the reaction stirred at 24 °C. The base was removed by rotary evaporation, the residual solution was filtered

and purified by RP-HPLC (C18, 10-90% ACN/H_2O, 0.1% HCOOH, 220 nm). The product containing fractions were identified by LCMS and lyophilized to yield pure title compound **17** as a beige solid (27.39 mg, 0.019 mmol, 73%).

^{1}H-NMR (700 MHz, DMSO-d6) δ 9.62 (m, 3H), 9.39 (m, 1H), 7.77 (m, 2H), 7.65 (m, 1H), 7.36 (m, 3H), 7.21 (m, 1H), 3.74 (m, 1H), 3.48 (m, 2H), 3.45 (t, J = 7.51 Hz, 6H), 2.99 (q, J = 7.05, 12.71 Hz, 4H), 2.57 (m, 4H), 2.26 (q, J = 6.59, 12.87 Hz, 4H), 1.96 (s, 3H), 1.54 (m, 4H), 1.49 (m, 4H), 1.38 (m, 4H), 1.26 (m, 2H), 1.21 (m, 5H), 1.01 (m, 6H).

^{13}C-NMR (176 MHz, DMSO-d6): δ = 186.47, 182.02, 181.84, 179.49, 176.37, 171.94, 171.27, 170.09, 168.77, 166.94, 165.92, 163.17, 162.07, 142.75, 142.73, 142.28, 131.95, 126.67, 123.91, 106.76, 82.16, 78.86, 47.11, 47.05, 46.75, 45.69, 43.72, 38.40, 38.38, 29.86, 28.79, 28.66, 28.19, 27.96, 27.54, 26.86, 26.10, 26.00, 23.57, 23.47, 21.64, 21.04, 20.33, 19.76, 12.70, 12.60, 11.03, 10.93, 7.59.

HRMS (ESI) calculated for ($[M+H]^+$): m/z = 1410.6021; experimental = 1410.6022

RNAP inhibitor (RNAP-I) constructs
Rifamycin S intermediates

Compound **61**

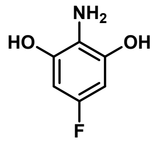

Chemical Formula: $C_6H_6FNO_2$
Exact Mass: 143,0383
Molecular Weight: 143,1174

The title compound **61** was synthetized according to a patent by Bachmann et al.[8] 1,3,5-trifluoro-2-nitrobenzene (from TCI, 5000 mg, 28.24 mmol, 1.0 eq) was dissolved in iPrOAc (150 mL). Then benzyl alcohol (3.23 ml, 31.06 mmol, 1.1 eq) was mixed with KOtBu (4752.51 mg, 42.35 mmol, 1.5 eq) for 5 minutes in dry iPrOAc (20 mL) and then added dropwise over 30 minutes via a dropping funnel. The reaction continued stirring at 0 °C for 2 hours. The solvent was removed *in vacuo* and the residue was washed with cold petrolether (bp 60-80°C, 5x 200 mL) over a glass frit to yield crude **61a** which was dried for one hour. The residue was dissolved in anhydrous MeOH/toluene under argon atmosphere, Pd/C (50 mg, 0.2 eq) was added and the atmosphere was changed to hydrogen. The solution stirred overnight at 35 °C with three balloons. Removal of the solvent by rotary evaporation yielded resorcinol crude **61** as a beige solid, which was washed with ice-cold diethyl ether, petrolether and DCM (each 100 ml) before being dried under reduced pressure (1.9 g, 13.42 mmol, 48% over 2 steps).

61a:

^{1}H-NMR (500 MHz, $CDCl_3$): δ = 7.37 (m, 10H), 6.39-6.37 (d, J = 10.42 Hz, 2H), 5.12 (s, 2H).

61:

^{1}H-NMR (500 MHz, $CDCl_3$): δ = 6.08-6.06 (d, J = 9.73 Hz), 3.46 (bs, 2H).

^{13}C-NMR (126 MHz, $CDCl_3$): δ = 158.08, 156.21, 147.05, 146.95, 117.33, 94.76, 94.56, 49.74, 49.57, 49.40, 49.23, 49.06, 48.89, 48.72.

HRMS (ESI) calculated for ($[M+H]^+$): m/z = 144.0456; experimental = 144.0459.

Compound **2** with benzoquinone (BQ)/oxygen as oxidant

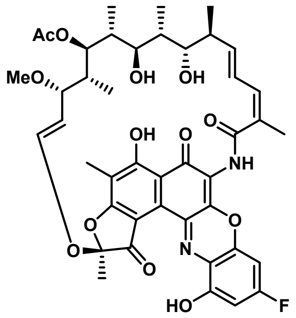

Chemical Formula: $C_{43}H_{47}FN_2O_{13}$
Exact Mass: 818,3062
Molecular Weight: 818,8484

The title compound **2** was synthetized according to a patent by Bachmann et al.[8] **61** (1.0 g, 1.44 mmol, 4.0 eq – 1 eq per cycle, 4 cycles total) and rifamycin S **1** (from TCI, 822.81 mg, 5.75 mmol, 4 eq) were weight in a glass reactor and dissolved in 25 mL iPrOAc (25 mL) under argon atmosphere. The brown-reddish solution continued stirring at ambient temperature for 2 hours and developed a blood red color. Then, the solution was cooled to 0 °C and the oxidant benzoquinone (621.45 mg, 5.75 mmol, 4 eq - 1 eq per cycle, 4 cycles in total) was added in iPrOAc (2 mL) over 10 minutes. The mixture was stirred at 25 °C for 1 hour. The addition procedure was repeated until all fluoride **61** and benzoquinone had been added and the reaction continued stirring at 25 °C for 48 h. The reaction was washed with 10% sodium ascorbate (w/v, 200 mL), and water (2 x 200 mL). The organic phase was dried over Na_2SO_4 and the solvent was removed by rotary evaporation. The residue was purified by SGC (PE - PE/EA 20%, UV-Vis, 24 g silica column) and product containing fractions were identified by TLC (DCM)/LCMS and combined. Then the solvent was removed by rotary evaporation to yield fluoro rifamycin **2** (252.0 mg, 0.31 mmol, 21%) as a blood red solid.

Compound **2** with TEMPO/oxygen as oxidant

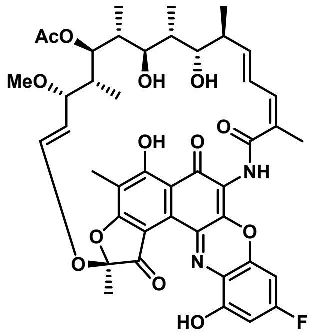

Chemical Formula: $C_{43}H_{47}FN_2O_{13}$
Exact Mass: 818,3062
Molecular Weight: 818,8484

The title compound **2** was synthetized according to a patent by Bachmann et al.[8] **61** (1.0 g, 1.44 mmol, 4.0 eq – 1 eq per cycle, 4 cycles total) and rifamycin S **1** (TCI, 822.81 mg, 5.75 mmol, 4 eq) were weight in a glass reactor and dissolved in 25 mL iPrOAc (25 mL) under argon atmosphere. The brown-reddish solution continued stirring at ambient temperature for 2 hours and developed a blood red color. Then the solution was cooled to 0 °C and the oxidant TEMPO (1122.87 mg, 7.19 mmol, 5 eq - – 1.25 eq per cycle, 4 cycles in total) was added in iPrOAc (2 mL) over 10 minutes while the atmosphere was changed to oxygen. Then the reaction continued stirring at 25 °C for 1 hour. Then the flask was charged with argon and the addition procedure was repeated till all fluoride **61** and TEMPO had been added. The reaction continued stirring overnight at 25 °C for 48 h. The reaction was washed with 10% sodium ascorbate (w/v, 200 mL), and water (2x200 mL). The organic phase was dried over Na_2SO_4 and the solvent was removed by rotary evaporation. The residue was purified by SGC (PE - PE/EA 20%, UV-Vis, 24 g silica column) and product containing fractions were identified by TLC (DCM)/LCMS and combined. Then the solvent was removed by rotary evaporation to yield pure **2** (589.5 mg, 0.72 mmol, 50%) as a blood red solid.

^{1}H-NMR (700 MHz, CD_2Cl_2): δ = 14.35 (s, 1H), 10.23 (s, 1H), 7.47 (bs, 1H), 6.76 (dd, J = 3.22, 10.40Hz, 1H), 6.66 (dd, J = 2.23, 9.29 Hz, 1H), 6.11 (m, 1H), 5.98 (m, 1H), 5.04 (m, 1H), 4.99 (q, J = 8.30, 12.14 Hz, 1H), 3.07 (s, 4H), 2.99 (m, 1H), 2.32 (s, 3H), 2.10 (s, 4H), 2.02 (s, 3H), 1.78 (s, 3H), 1.69 (q, J = 8.13, 13.97 Hz, 1H), 1.55 (bs, 6H), 1.41 (m, 2H), 1.36 (m, 1H), 1.30 (m, 1H), 0.91 (bs, 3H), 0.76 (bs, 3H), 0.53 (bs, 3H).

^{13}C-NMR (176 MHz, CD_2Cl_2): δ = 194.38, 184.63, 174.60, 172.52, 169.12, 168.58, 167.12, 158.14, 158.05, 145.25, 143.43, 142.26, 142.10, 140.93, 131.74, 126.63, 120.47, 113.75, 107.89, 100.13, 99.98, 95.46, 95.29, 73.96, 41.63, 37.30, 32.97, 22.61, 21.27, 21.00, 18.81, 17.69, 11.08, 8.09.

HRMS (ESI) calculated for ($[M+H]^+$): m/z = 819.3135; experimental = 819.3135.

Compound **22**

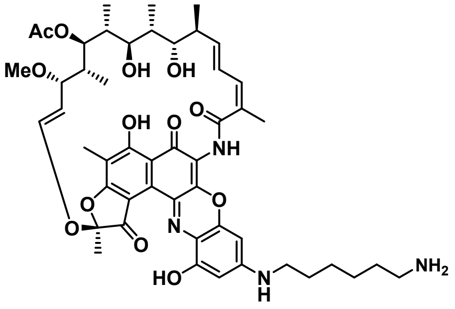

Chemical Formula: $C_{49}H_{62}N_4O_{13}$
Exact Mass: 914,4313
Molecular Weight: 915,0500

Fluoride **2** (50 mg, 0.061 mmol, 1.0 eq) was dissolved in anhydrous THF (1 mL) under argon atmosphere. Then *N*-Fmoc 1,6-diaminohexane (22.7 mg, 0.067 mmol, 1.1 eq) and TEA (100 µL) were added. The reaction continued stirring overnight and a color change from red to blue, as well as a partial cleavage of the Fmoc group was observed. The reaction was concentrated to dryness and then ACN (800 µL) and diethylamine (200 µL) were added. The reaction continued stirring for one hour at ambient temperature before the solvent was removed and the residue was washed with diethyl ether (3x50 mL, ice-cold, 4000 g, 5 min, 0 °C) and dried overnight under reduced pressure to yield **22** as crude, blue solid (51,42 mg, 0.056 mmol, 92%).

^{1}H-NMR (700 MHz, MeOH-d_4): δ = 6.84 (m, 1H), 6.40 (m, 2H), 6.23 (m, 3H), 5.02 (m, 2H), 3.69 (d, J = 8.61 Hz, 1H), 3.59 (m, 3H), 3.19 (m, 3H), 3.11 (m, 1H), 2.99 (m, 5H), 2.29 (m, 4H), 2.10 (m, 4H), 1.97 (m, 4H), 1.78 (m, 4H), 1.61 (m,1H), 1.29 (m, 3H), 0.93 (m, 10H), 0.78 (m, 2H), 0.69 (m, 1H), 0.04 (d, J = 7.13 Hz, 3H), -0.30 (d, J = 7.87 Hz, 3H).

HRMS (ESI) calculated for ($[M+H]^+$): m/z =915.4386; experimental = 915.4392.

Compound **62**

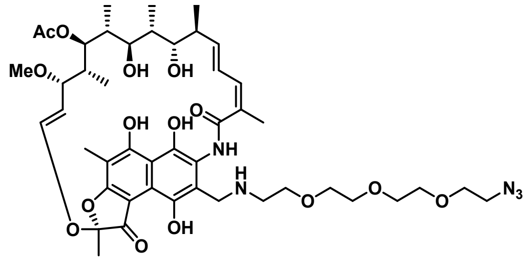

Chemical Formula: $C_{46}H_{65}N_5O_{15}$
Exact Mass: 927,4477
Molecular Weight: 928,0460

3-formyl rifamycin SV **27** (abcam: ab143401, 50.0 mg, 0.069 mmol, 1.0 eq) was dissolved in anhydrous THF (15 mL) under argon atmosphere. Then the N_3-PEG-NH_2 (16.5 mg, 0.076 mmol, 1.1 eq) in THF (5 mL) and TEA (50 µL) was added. The solvent was removed *in vacuo* and the residue was taken up in DCM (100 mL). The organic phase was washed with 1 M HCl (2x100 mL), water (1x100 mL), brine (1x50 mL) and dried over Na_2SO_4. The solvent was removed by rotary evaporation and dried under reduced pressure. The residue was dissolved in anhydrous THF (20 mL) under argon atmosphere, cooled to 0 °C before $NaBH(OAc)_3$ (21.9 mg, 0.103 mmol, 2.0 eq) was added. The reaction continued stirring overnight at 23 °C, was then concentrated to dryness and taken up in DCM (100 mL). The organic phase was washed water (1x100 mL), brine (1x50 mL) and dried over Na_2SO_4. The solvent was removed by rotary evaporation and dried under reduced pressure to yield the crude amine **62** (62.4 mg, 0.067 mmol, 98%).

62

^{1}H-NMR (700 MHz, CD_2Cl_2): δ = 8.75 (s, 1H), 6.61 (q, J = 11.16 Hz, 1H), 6.33 (d, J = 10.80 Hz, 1H), 6.15 (d, J = 12.57 Hz, 1H), 6.02 (dd, J = 5.16, 15.96 Hz, 1H), 5.13 (dd, J = 6.13, 12.09 Hz, 1H), 4.98 (d, J = 10.64 Hz, 1H), 3.86 (m, 1H), 3.75 (m, 4H), 3.63 (m, 8H), 3.61 (m, 4H), 3.59 (m, 4H), 3.50 (m, 1H), 3.41 (t, J = 5.07 Hz, 2H), 3.35 (t, J = 4.98 Hz, 2H), 3.05 (s, 3H), 3.02 (m, 2H), 2.33 (m, 1H), 2.14 (s, 3H), 2.11 (s, 3H), 2.03 (s, 3H), 2.01 (m, 1H), 1.77 (s, 3H), 1.74 (m, 1H), 1.56 (m, 1H), 1.49 (m, 1H), 1.00 (d, J = 7.12 Hz, 3H), 0.84 (d, J = 7.12 Hz, 3H), 0.62 (d, J = 6.94 Hz, 3H), -0.14 (d, J = 7.03 Hz, 3H).

27 (3-formyl rifamycin S)

^{1}H-NMR (700 MHz, CD_2Cl_2): δ = 13.11 (s, 1H), 12.64 (s, 1H), 12.26 (s, 1H), 10.61 (s, 1H), 6.48 (m, 2H), 6.23 (d, J = 12.80 Hz, 1H), 6.03 (m, 1H), 5.11 (q, J = 6.77, 12.80 Hz, 1H), 4.91 (d, J = 10.59 Hz, 1H), 3.75 (d, J = 9.85 Hz, 1H), 3.51 (d, J = 6.91 Hz, 1H), 3.02 (m, 4H), 2.37 (m, 1H), 2.25 (s, 3H), 2.08 (s, 3H), 2.05 (m, 4H), 1.81 (s, 3H), 1.77 (m, 1H), 1.51 (m, 1H), 1.35 (m,

1H), 1.00 (d, J = 7.21 Hz, 3H), 0.88 (d, J = 6.77 Hz, 3H), 0.66 (d, J = 6.77 Hz, 3H), -0.33 (d, J = 7.21 Hz, 3H).

Compound **28**

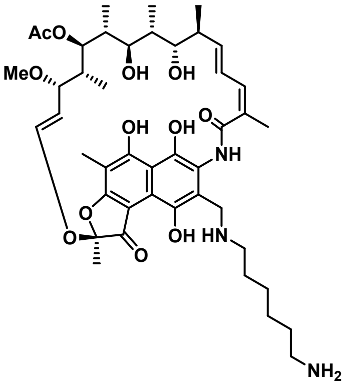

Chemical Formula: $C_{44}H_{63}N_3O_{12}$
Exact Mass: 825,4412
Molecular Weight: 825,9970

3-formyl rifamycin SV **27** (abcam: ab143401, 100 mg, 0.138 mmol, 1.0 eq) was dissolved in anhydrous THF (15 mL) under argon atmosphere. Then the *N*-Fmoc-1,6-diaminohexane (93.3 mg, 0.276 mmol, 2.0 eq) in THF (5 mL) and TEA (50 µL) was added. The solvent was removed *in vacuo* and the residue was taken up in DCM (100 mL). The organic phase was washed with 1 M HCl (2x100 mL), water (1x100 mL), brine (1x50 mL) and dried over Na_2SO_4. The solvent was removed by rotary evaporation and dried under reduced pressure. The residue was dissolved in anhydrous THF (20 mL) under argon atmosphere, cooled to 0 °C before $NaBH(OAc)_3$ (58.4 mg, 0.276 mmol, 2.0 eq) was added. The reaction continued stirring overnight at 23 °C, was then concentrated to dryness and taken up in DCM (100 mL). The organic phase was washed water (1x100 mL), brine (1x50 mL) and dried over Na_2SO_4. The solvent was removed by rotary evaporation and dried under reduced pressure to yield **28a**. The residue was dissolved in ACN (16 mL) and diethylamine (4 mL) were added. The solution continued stirring at 23 °C for 45 minutes and was then evaporated by rotary evaporation. The residue was washed with ice-cold petrol ether and diethyl-ether (-20 °C), further drying under reduced pressure gave crude title compound **28** (76.1 mg, 0.092 mmol, 67% over three steps) as a red solid.

HRMS (ESI) calculated for ($[M+H]^+$): m/z = 826.4485; experimental = 826.4433.

Compound **63**

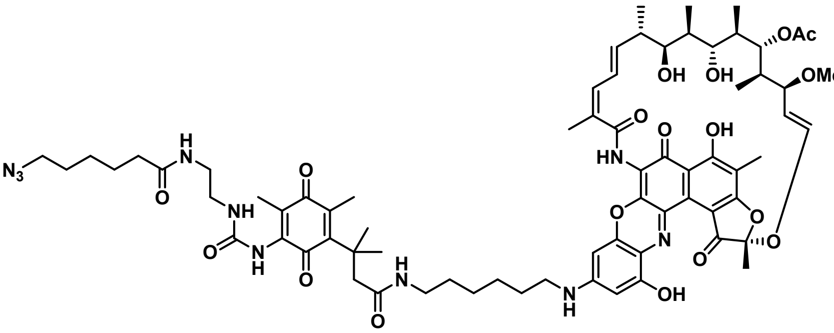

Chemical Formula: $C_{71}H_{92}N_{10}O_{18}$
Exact Mass: 1372,6591
Molecular Weight: 1373,5690

Acid **53** (6 mg, 0.013 mmol, 1.0 eq) was dissolved under argon atmosphere in anhydrous THF (1 mL) and NMM (20 µL) was added. The reaction was cooled to 0 °C and *iso*-butyl chloroformate (1.22 µL, 0.013 mmol, 1.0 eq) was added. The reaction mixture was stirred for one hour at 0 °C and during this time, the color of the solution changed from yellow to orange. Following 15 min agitation at 23 °C, amine **22** (11.52 mg, 0.013 mmol, 1.0 eq) diluted in THF (1 mL) and basified with NMM (20 µL) was added at 0 °C. The ice-bath was left to thaw overnight. The solvent was removed by rotary evaporation, the residue diluted with DCM (100 mL) and washed with 1M HCl (3x50 mL), brine (1x50 mL) and dried over Na_2SO_4. The solvent was removed *in vacuo* and residue was dried under reduced pressure to yield crude **63** as a blue solid (6.8 mg, 0.005 mmol, 39%).

Compound **64**

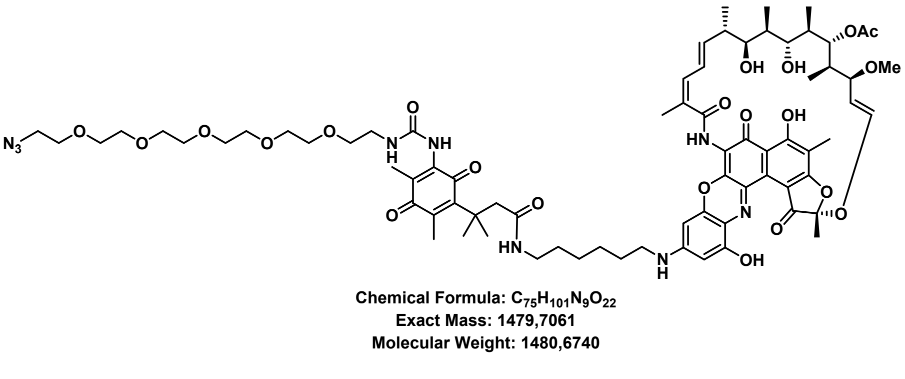

Chemical Formula: $C_{75}H_{101}N_9O_{22}$
Exact Mass: 1479,7061
Molecular Weight: 1480,6740

Acid **56** (13 mg, 0.029 mmol, 1.0 eq) was dissolved under argon atmosphere in anhydrous THF (1 mL) and NMM (20 µL) was added. The reaction was cooled to 0 °C and iso-butyl chloroformate (2.79 µL, 0.029 mmol, 1.0 eq) was added. The reaction continued to stir for one hour at 0 °C while the color of the solution changed from yellow to orange. Then the reaction stirred for 15 min at 23 °C, before the amine **22** (26.3 mg, 0.029 mmol, 1.0 eq) diluted in THF (1 mL) and basified with NMM (20 µL) was added at 0 °C. The ice-bath was left to thaw

overnight. The solvent was removed by rotary evaporation, the residue diluted with DCM (100 mL) and washed with 1M HCl (3x50 mL), brine (1x50 mL) and dried over Na_2SO_4. The solvent was removed *in vacuo* and residue was dried under reduced pressure to yield crude **64** as a blue solid (19.29 mg, 0.013 mmol, 45%).

Compound **65**

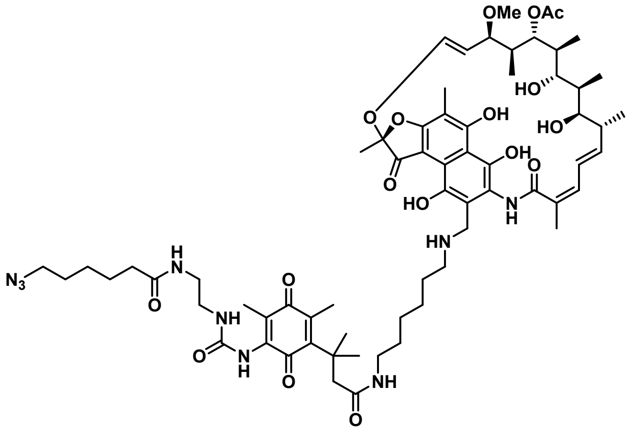

Chemical Formula: $C_{66}H_{93}N_9O_{17}$
Exact Mass: 1283,6689
Molecular Weight: 1284,5160

<u>EDCI/HOBt procedure</u>

Acid **53** (10 mg, 0.021 mmol, 1.0 eq) was dissolved in DCM/DMF (10:1 - 15 mL) and EDCI*HCl (6.52 mg, 0.042 mmol, 2.0 eq), HOBt (5.67 mg, 0.042 mmol, 2.0 eq) and DMAP (1.28 mg, 0.01 mmol, 0.5 eq) were added at 0 °C. The mixture stirred 20 minutes at that temperature and equilibrated to room temperature over 20 minutes. Then the amine **28** (20.81 mg, 0.013 mmol, 1.2 eq) and TEA (30 µL) were added, dissolved in DCM/DMF (5 mL) at 0 °C, and the ice bath was removed and the reaction continued stirring at ambient temperature for 18 hours. The solvent was removed by rotary evaporation and the residue was purified by RP-HPLC (15%-100% ACN/H_2O, 220 nm, collect all). The product containing fractions were combined and lyophilized to dryness yielding **65** as an orange solid (12.3 mg, 0.01 mmol, 38%).

<u>Mixed anhydride procedure</u>

Compound **65** could also be afforded by mixed anhydride coupling, with the same reaction conditions to the procedure of compound **66** below, with nearly the double yield (23.6 mg, 0.02, 73%).

^{1}H-NMR (700 MHz, DMSO-d6): δ = 12.77 (s, 1H), 9.30 (bs, 1H), 7.72 (m, 1H), 6.69 (d, 1H), 6.62 (m, 1H), 6.33 (d, J = 10.91 Hz, 1H), 6.24 (d, J = 11.31 Hz, 1H), 6.08 (q, 1Hz), 5.09 (d, J = 14.66 Hz, 1H), 5.04 (d, J = 3.91 Hz, 1H), 4.90 (q, J = 8.51, 12.89 Hz, 1H), 3.99 (d, J = 8.98

Hz, 1H), 3.68 (m, 2H), 3.29 (m, 5H), 3.24 (d, J =8.58 Hz, 1H), 3.07 (m, 1H), 2.96 (m, 6H), 2.88 (s, 3H), 2.82 (t, J = 9.81 Hz, 2.29 (m, 1H), 2.03 (m, 6H), 1.97 (m, 7H), 1.91 (s, 3H), 1.77 (s, 1H), 1.69 (m, 2H), 1.64 (s, 3H), 1.50 (m, 8H), 1.34 (m, 5H), 1.27 (m, 8H), 1.22 (m, 6H), 0.99 (m, 2H), 0.92 (d, J = 7.10 Hz, 3H), 0.86 (d, J = 6.95 Hz, 3H), 0.50 (d, J = 6.45 Hz, 3H), -0.33 (d, J 6.45 Hz, 3H).

DEPT (176 MHz, DMSO-d6) δ = 142.82, 138.88, 131.31, 128.80, 126.32, 125.66, 117.35, 114.38, 76.05, 75.51, 72.92, 72.63, 55.38, 50.29, 50.26, 39.52, 37.97, 37.84, 37.59, 34.98, 32.40, 31.19, 28.64, 27.75, 25.70, 25.55, 25.31, 24.58, 24.48, 21.81, 20.40, 19.60, 17.95, 10.92, 8.71, 8.53, 7.09.

HRMS (ESI) calculated for ($[M+2H]^{2+}$): m/z =642.8417 experimental = 642.8423.

Compound **66**

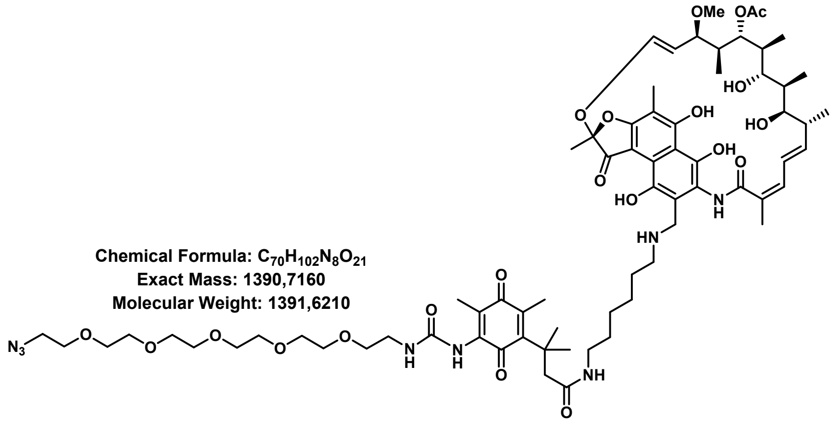

Acid **56** (8 mg, 0.014 mmol, 1.0 eq) was dissolved under argon atmosphere in anhydrous THF (500 µL) and NMM (15 µL) was added. The reaction was cooled to 0 °C and *iso*-butyl chloroformate (1.87 mg, 0.015 mmol, 1.0 eq) was added. The reaction continued to stir for one hour at 0 °C while the color of the solution changed from yellow to orange. Then the reaction stirred for 15 min at 23 °C, before amine **28** (22.6 mg, 0.027 mmol, 2.0 eq), diluted in THF (500 µL) and basified with NMM (15 µL), was added at 0 °C. The ice-bath was left to thaw overnight. The solvent was removed by rotary evaporation and purified by RP-HPLC (10-100% ACN/H_2O, 0.1% HCOOH, 220 nm). The product containing fractions were lyophilized to yield the title compound **66** (13.9 mg, 0.01 mmol, 73%) as a yellow-orange solid.

^{1}H-NMR (700 MHz, CD_2Cl_2): δ = 8.08 (bs, 1H), 6.54 (q, J = 9.77, 15.52 Hz, 1H), 6.34 (d, J = 10.92 Hz, 1H), 6.23 (dd, J = 7.33, 15.66 Hz, 1H), 6.14 (d, J = 12.79 Hz, 1H), 5.06 (q, J = 6.32,

12.79 Hz, 1H), 4.93 (d, J = 10.06 Hz, 1H), 4.38 (d, J = 13.07 Hz, 1H), 3.62 (m, 18H), 3.44 (m, 2H), 3.38 (m, 4H), 3.28 (bs, 1H), 3.08 (m, 9H), 2.62 (m, 2H), 2.38 (m, 1H), 2.15 (m, 4H), 2.07 (s, 3H), 2.02 (s, 6H), 1.88 (m, 3H), 1.76 (m, 1H), 1.68 (m, 6H), 1.47 (d, J = 11.64 Hz, 8H), 1.42 (s, 6H), 1.29 (m, 10H), 1.19 (m, 2H), 1.01 (d, J = 7.04 Hz, 3H), 0.88 (d, J = 6.03 Hz, 3H), 0.60 (d, J = 7.61 Hz, 3H), -0.19 (d, J = 6.18 Hz, 3H).

^{13}C-NMR (176 MHz, CD_2Cl_2): δ = 205.59, 204.73, 204.66, 202.52, 194.46, 193.79, 187.92, 187.72, 187.17, 173.44, 172.69, 172.30, 172.27, 143.42, 138.49, 127.14, 125.42, 125.15, 109.21, 78.58, 77.36, 74.60, 71.07, 70.96, 70.88, 70.79, 70.74, 70.70, 70.60, 70.54, 70.39, 70.38, 57.24, 54.00, 51.80, 51.33, 51.28, 49.75, 47.64, 39.77, 39.16, 38.81, 38.22, 38.00, 34.00, 32.50, 30.59, 30.26, 29.93, 29.60, 29.51, 21.95, 21.19, 20.52, 18.27, 18.21, 14.83, 14.44, 11.22, 9.57, 9.14, 7.37

HRMS (ESI) calculated for ($[M+2H]^{2+}$): m/z = 696.3653; experimental = 696.3651.

Compound **73**

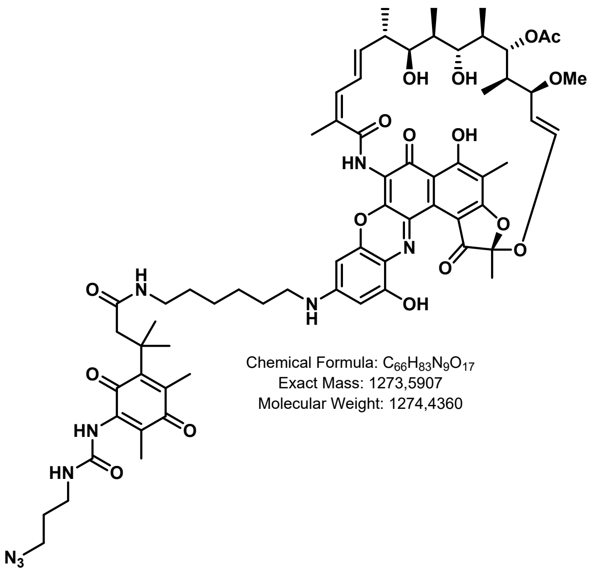

Acid **72** (5.0 mg, 0.013 mmol, 1.0 eq) was dissolved in anhydrous DMF (2.5 mL) and NMM (80 µL) and iso-butylchloroformate (1.71 µL, 0.031 mmol, 1.0 eq) were added in one portion at 0 °C under argon atmosphere. The reaction stirred 45 minutes at 0 °C before **22** (18.2 mg, 0.013 mmol, 1.0 eq) was added dropwise in anhydrous DMF (2.5 mL) at 0 °C and the ice bath was left to thaw. Afterwards the reaction stirred 60 minutes more and then the solvent was removed by rotary evaporation at 30 °C water bath temperature. The residue was taken up in DCM (20 mL), washed with 1 M HCl, sat. $NaHCO_3$ and brine (each 2x10 mL) and dried over Na_2SO_4.

The solvent was evaporated to dryness to yield **73** as a crude blue powder (5.9 mg, 0.004 mmol, 34%).

^{1}H-NMR (700 MHz, MeOH-d_4): δ = 6.92 (s, 1H), 6.86 (m, 1H), 6.38 (m, 1H), 6.29 (m, 2H), 6.19 (m, 2H), 5.00 (m, 2H), 3.37 (m, 4H), 3.21 (m, 6H), 3.09 (m, 4H), 2.98 (m, 5H), 2.36 (m, 1H), 2.26 (m, 5H), 2.22 (s, 3H), 2.14 (s, 3H), 2.12 (m, 2H), 2.09 (m, 2H), 1.97 (m, 4H), 1.85 (m, 6H), 1.78 (m, 4H), 1.73 (m, 6H), 1.64 (m, 9H), 1.46 (m, 9H), 1.40 s, 9H), 1.32 (s, 6H), 1.23 (m, 6H), 1.01 (m, 6H), 0.92 (m, 12H), 0.78 (m, 2H), 0.07 (m, 3H), -0.37 (m, 3H).

^{13}C-NMR (176 MHz, MeOH-d_4): δ = 163.87, 145.37, 140.77, 134.16, 127.39, 126.26, 121.06, 78.10, 76.36, 75.18, 74.93, 72.01, 56.94, 50.21, 50.14, 49.42, 49.37, 46.11, 44.57, 41.90, 41.69, 40.56, 40.09, 39.53, 38.97, 38.63, 34.29, 31.03, 30.47, 30.44, 29.89, 29.73, 29.51, 29.27, 27.74, 27.59, 22.93, 22.35, 21.46, 20.85, 20.48, 19.50, 19.17, 15.27, 14.94, 14.73, 10.99, 10.00, 7.52.

HRMS (ESI) calculated for ($[M+H]^+$: m/z = 1274.5979; experimental = 1274.5972

Sorangicin A intermediates

Compound **32**

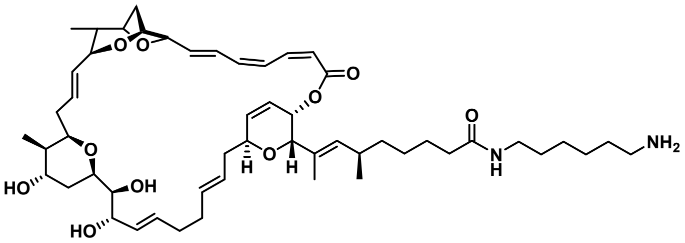

Chemical Formula: $C_{53}H_{80}N_2O_{10}$
Exact Mass: 904,5813
Molecular Weight: 905,2270

Sorangicin A **31** (50 mg, 0.062 mmol, 1.0 eq) was dissolved in anhydrous DCM/DMF (6 mL 1:10) and HATU (47.12 mg, 0.124 mmol, 2.0 eq) was added. The suspension was stirred at 23 °C for 10 minutes, before the *N*-Fmoc 1,6-diaminohexane (41.84 mg, 0.124 mmol 2.0 eq) and DIPEA (50 µL) were added. The suspension cleared visibly and the reaction continued stirring 2 hours at 23 °C. Upon completion, the solvent was removed by rotary evaporation. The residue was dissolved in ACN (8 mL) and diethylamine (2 mL) was added. The solution was stirred at 23 °C for 1 hour and then the solvent was removed *in vacuo*. The residue was taken up in ACN (4 mL), purified by RP-HPLC (5-85% ACN/H_2O, 220 nm, collect all) and product containing fractions were lyophilized yielding **32** as a beige solid (38.5 mg, 0.044 mmol, 56%).

^{1}H-NMR (700 MHz, DMSO-d_6) δ = 7.68 (t, J = 5.12 Hz, 1H), 7.58 (bs, 3H), 7.11 (t, J = 11.88 Hz, 1H), 7.03 (t, J = 11.40 Hz, 1H), 6.92 (t, J = 13.42 Hz, 1H), 6.47 (t, J =11.40 Hz, 1H), 6.22 (dd, J = 3.67, 15.07 Hz, 1H), 6.14 (dd, J = 3.09, 9.57 Hz, 1H), 5.96 (m, 1H), 5.62 (d, J = 10.91 Hz, 1H), 5.53 (m, 3H), 5.45 (q, J = 4.73, 8.79 Hz, 2H), 5.30 (m, 5H), 5.22 (d, J = 8.31 Hz, 2H), 4.56 (m, 1H), 4.49 (m, 1H), 4.44 (m, 2H), 4.38 (m, 1H), 4.34 (m, 1H), 4.18 (m, 2H), 3.94 (t, J = 5.12 Hz, 1H), 3.73 (t, J = 7.24 Hz, 1H), 3.68 (m, 1H), 3.63 (t, J = 8.31 Hz, 1H), 3.53 (m, 1H), 3.29 (m, 1H), 3.01 (m, 2H), 2.76 (m, 2H), 2.28 (m, 3H), 2.09 (m, 5H), 2.00 (m, 4H), 1.80 (d, J = 10.82 Hz, 1H), 1.54 (s, 3H), 1.50 (m, 4H), 1.43 (m, 3H), 1.36 (m, 3H), 1.27 (m, 4H), 1.13 (m, 2H), 0.78 (d, J = 6.18, 3H), 0.76 (d, J = 7.24 Hz, 3H), 0.71 (d, J = 6.18 Hz, 3H).

^{13}**C-NMR** (176 MHz, DMSO-d$_6$): δ = 172.11, 165.20, 137.63, 136.82, 135.85, 135.00, 132.22, 132.08, 131.87, 130.54, 130.32, 130.20, 129.55, 126.68, 125.39, 124.58, 122.36, 118.38, 80.01, 78.85, 78.44, 76.27, 75.13, 72.97, 72.72, 72.46, 72.14, 71.70, 68.44, 64.73, 40.12, 38.81, 38.50, 38.22, 36.82, 36.76, 35.66, 35.58, 33.69, 32.57, 32.11, 31.09, 28.99, 26.94, 26.58, 25.86, 25.44, 25.37, 20.79, 14.77, 13.48, 10.26.

HRMS (ESI) calculated for ([M+H]$^+$): m/z = 905.5886; experimental =905.5855.

Compound **67**

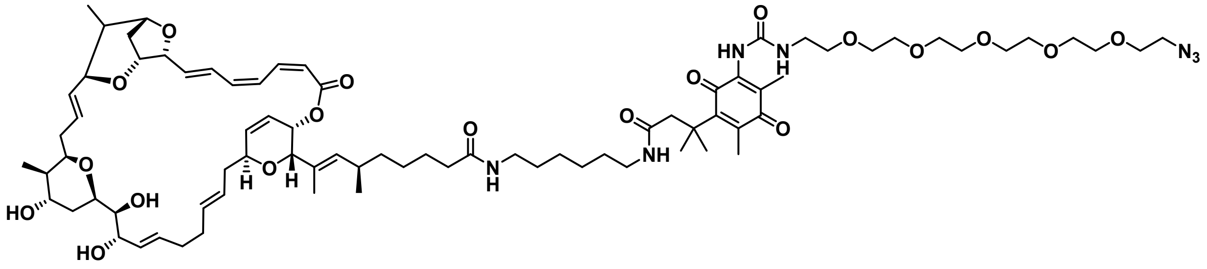

Chemical Formula: $C_{79}H_{119}N_7O_{19}$
Exact Mass: 1469,8561
Molecular Weight: 1470,8510

Acid **56** (10 mg, 0.017 mmol, 1.0 eq) was dissolved in dry THF (500 µL) under argon atmosphere, NMM (30 µL) was added and the flask was cooled to 0 °C. Then *iso*-butylchloroformate (1.66 µL, 0.017 mmol, 1.0 eq) was added at 0 °C and the reaction was stirred at that temperature for one hour. The color changed from yellow to orange and the amine **32** (23.3 mg, 0.026 mmol, 1.5 eq) was added in THF (500 µL) with NMM (30 µL) at 0 °C. The reaction continued stirring overnight, while the thawing ice bath equilibrated the reaction steadily to ambient temperature. The next morning the solvent was removed and the reaction was purified by RP-HPLC (10-100% ACN/H_2O, 0.1% HCOOH, 220 nm). Product containing fractions were identified and lyophilized to yield **67** (21.1 mg, 0.14 mmol, 84%) as a slight yellow powder.

^{1}H-NMR (700 MHz, CD_2Cl_2): δ = 7.13 (t, J = 11.57 Hz, 1H), 7.05 (t, J = 12.82 Hz, 1 H), 6.97 (t, J = 13.45 Hz, 1H), 6.63 (m, 1H), 6.43 (t, J = 10.94 Hz, 1H), 6.24 (m, 1H), 6.06 (m, 3H), 5.73 (m, 1H), 5.57 (m, 2H), 5.53 (m 2H), 5.45 (m, 1H), 5.35 (m, 1H), 4.51 (s, 1H), 4.39 (m, 1H), 4.35 (m, 1H), 4.24 (m, 2H), 4.10 (s, 1H), 3.88 (m, 2H), 3.82 (m, 2H), 3.78 (t, J = 10.01 Hz, 1H), 3.70 (m, 1H), 3.63 (m, 22H), 3.55 (t, J = , 5.63 Hz, 2H), 3.37 (t, J = 5.32 Hz, 4H), 3.10 (m, 4H), 3.04 (m, 1H), 2.60 (dd, J = 12.51, 14.10 Hz, 2H), 2.36 (m, 2H), 2.25 (m, 1H), 2.18 (m, 2H), 2.13 (s, 3H), 2.10 (m, 6H), 1.96 (m, 1H), 1.89 (s, 3H), 1.79 (m, 1H), 1.74 (m, 1H), 1.67 (m, 2H), 1.58 (m, 5H), 1.48 (s, 6H), 1.39 (m, 2H), 1.32 (m, 4H), 1.26 (s, 2H), 1.19 (m, 7H), 0.94 (d, J = 6.74 Hz, 2H), 0.90 (t, J = 6.74, 2H), 0.85 (d, J = 7.41 Hz, 3H), 0.81 (d, J = 6.41 Hz, 3H), 0.77 (d, J = 6.30 Hz, 3H).

^{13}C-NMR (176 MHz, CD_2Cl_2): δ = 187.09, 174.46, 166.47, 137.85, 137.13, 136.23, 134.96, 133.90, 132.91, 132.79, 132.22, 131.68, 129.42, 129.17, 127.73, 127.12, 126.08, 123.50, 119.33, 81.00, 80.15, 80.06, 76.65, 76.61, 74.91, 74.54, 74.39, 74.35, 73.83, 73.12, 71.10, 71.07, 71.04, 70.97, 70.92, 70.79, 70.67, 70.59, 70.43, 64.77, 54.00, 51.34, 49.81, 41.35, 40.65, 39.75, 39.53, 39.14, 38.28, 37.52, 37.11, 36.33, 34.66, 33.56, 33.09, 32.56, 31.46, 30.32, 30.28, 29.84, 29.80, 28.39, 27.98, 26.64, 26.56, 26.45, 21.79, 19.36, 19.21, 15.32, 14.79, 14.35, 11.72, 10.89.

HRMS (ESI) calculated for ($[M+2H]^{2+}$): m/z =735.9353; experimental = 735.9352.

Compound **68**

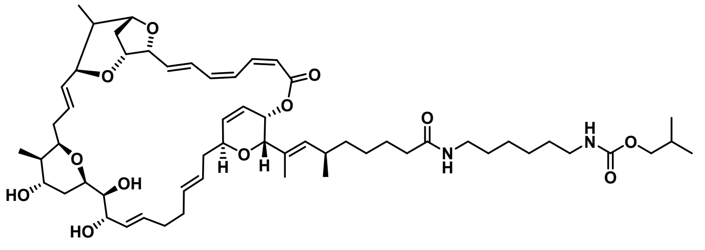

Chemical Formula: $C_{58}H_{88}N_2O_{12}$
Exact Mass: 1004,6337
Molecular Weight: 1005,3440

The compound **68** (4.05 mg, 0.004 mmol, 24%) was obtained as a side product during the synthesis of **32**.

^{1}**H-NMR** (700 MHz, CD_2Cl_2): δ = 7.13 (t, J = 11.98 Hz, 1H), 7.05 (t, J = 12.11 Hz, 1H), 6.97 (m, 1H), 6.43 (t, J = 10.94 Hz, 1H), 6.22 (dd, J = 4.17, 15.37 Hz, 1H), 6.05 (m, 2H), 5.74 (m, 2H), 5.58 (dt, J = 1.56, 11.59 Hz, 1H), 5.53 (m, 3H), 5.45 (m, 1H), 5.34 (m, 2H), 5.29 (dt, J = 2.21, 9.77, 1H), 4.51 (m, 1H), 4.39 (m, 1H), 4.35 (q, J = 2.21 Hz, 1H), 4.25 (d, J = 5.99 Hz, 1H), 4.19 (q, J = 5.21, 10.61 Hz, 1H), 4.11 (s, 1H), 3.89 (m, 1H), 3.84 (m, 1H), 3.78 (m, 2H), 3.70 (m, 1H), 3.59 (m, 1H), 3.48 (q, J = 5.99, 11.72 Hz, 1H), 3.17 (q, J = 6.77, 12.54 Hz, 2H), 3.12 (q, J = 6.38, 13.15 Hz, 2H), 2.68 (m, 2H), 2.33 (m, 2H), 2.25 (m, 1H), 2.11 (m, 14H), 1.98 (m, 1H), 1.88 (m, 2H), 1.79 (m, 1H), 1.69 (m, 1H), 1.59 (s, 1H), 1.56 (m, 9H), 1.47 (q, J = 7.16, 13.54 Hz, 4H), 1.32 (m, 8H), 1.18 (m, 2H), 0.91 (d, J = 6.77 Hz, 6H), 0.85 (d, J = 6.77 Hz, 3H), 0.81 (d, J = 6.38 Hz, 3H), 0.77. (d, J = 6.38 Hz, 3H).

^{13}**C-NMR** (176 MHz, CD_2Cl_2): δ = 206.95, 174.20, 166.45, 157.37, 137.85, 137.12, 136.20, 134.89, 133.86, 133.04, 132.77, 132.18, 131.78, 129.54, 129.23, 127.76, 127.05, 126.04, 123.48, 119.31, 81.03, 80.14, 80.02, 76.62, 76.46, 74.92, 74.63, 74.39, 73.90, 73.21, 71.30, 70.56, 64.92, 41.36, 41.11, 39.67, 39.54, 38.15, 37.60, 37.16, 36.33, 34.70, 33.46, 33.04, 32.45, 31.30, 31.16, 30.40, 29.89, 28.65, 27.87, 26.68, 26.54, 26.38, 21.67, 19.36, 15.31, 14.70, 10.87.

HRMS (ESI) calculated for ($[M+H]^+$): m/z = 1005.6410; experimental = 1005.6415.

Compound **69**

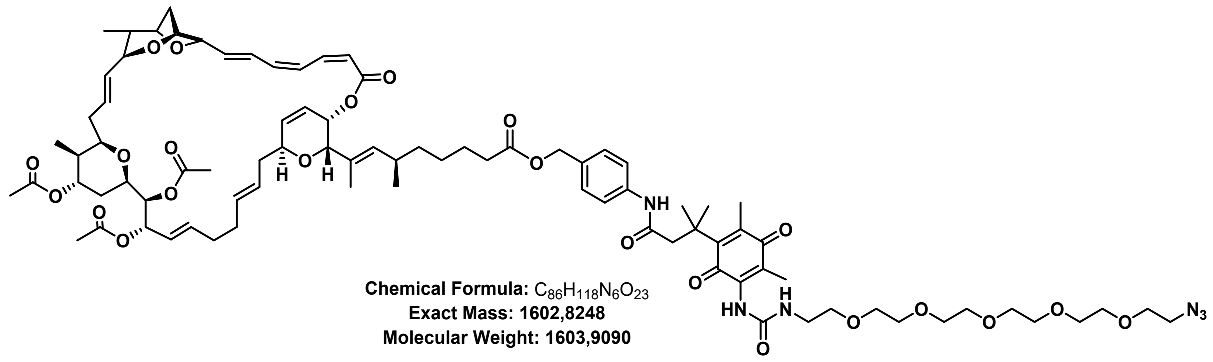

Sorangicin A **31** (20 mg, 0.025 mmol, 1.0 eq) was dissolved in anhydrous THF (5 mL) and pyridine (5 mL) and acetic anhydride (25.30 mg, 0.248 mmol, 10.0 eq) were added at 0 °C. Then the reaction was stirred for 4 hours at 24 °C. The solvent was removed *in vacuo* and the residue dried under reduced pressure for two hours, before anhydrous THF (2.5 mL) and NMM (100 µL) were added under argon atmosphere. The reaction was cooled to 0 °C and then *iso*-butyl chloroformate (2.4 µL, 0.025 mmol, 1.0 eq) was added and the slightly yellow solution

went turbid instantly. The reaction was stirred 10 minutes at 0 °C and 45 minutes at 24 °C, before benzyl alcohol **57** (20.48 mg, 0.03 mmol, 1.2 eq) was added in anhydrous THF (2.5 mL) together with anhydrous NMM (100 µL) at 0 °C. The reaction continued stirring for two hours at 24 °C and was then concentrated to dryness. The residue was purified by RP-HPLC (5-98% ACN/H_2O, 0.1% AcOH, 220 nm, collect all). The product containing fractions were lyophilized to yield **59** (25.2 mg, 0.016 mmol, 63% over two steps) as a beige powder.

^{1}H-NMR (600 MHz, DMSO-d_6): δ = 11.92 (s, 1H), 7.08 (m, 2H), 6.92 (m, 1H), 6.45 (t, J = 10.49 Hz, 1H), 6.21 (dd, J = 3.86, 15.28 Hz, 1H), 6.13 (dd, J = 3.15, 10.00 Hz, 1H), 5.95 (m, 1H), 5.62 (m, 2H), 5.43 (m, 3H), 5.34 (m, 1H), 5.24 (m, 3H), 4.82 (d, J = 5.51 Hz, 1H), 4.76 (dd, J = 4.33, 7.25 Hz, 1H), 4.61 (d, J = 2.52 Hz, 1H), 4.49 (m, 1H), 4.36 (m, 2H), 4.19 (m, 2H), 4.07 (m, 1H), 3.75 (m, 1H), 3.65 (m, 3H), 2.30 (m, 3H), 2.10 (m, 10H), 1.98 (s, 3H), 1.94 (m, 3H), 1.78 (d, J = 10.48 Hz, 1H), 1.54 (s, 3H), 1.45 (m, 3H), 1.34 (m, 6H), 1.17 (m, 3H), 1.08 (m, 1H), 0.80 (d, J = 6.62 Hz, 3H), 0.74 (d, J = 7.25 Hz, 3H), 0.70 (d, J = 6.77 Hz, 3H).

^{13}C-NMR (151 MHz, DMSO-d_6): δ = 174.52, 169.76, 165.15, 137.66, 136.81, 135.87, 134.96, 132.13, 132.09, 132.07, 131.97, 129.85, 129.70, 129.02, 126.84, 125.25, 124.57, 122.31, 118.34, 79.91, 78.84, 78.43, 77.25, 75.10, 72.61, 72.55, 72.12, 70.92, 70.05, 67.93, 64.79, 39.52, 38.50, 36.80, 36.55, 35.49, 33.80, 33.63, 32.40, 31.94, 31.16, 29.24, 26.52, 24.58, 20.80, 20.77, 14.72, 13.41, 10.14.

DEPT (151 MHz, DMSO-d6): δ = 137.40, 136.56, 135.62, 134.70, 131.87, 131.83, 131.81, 131.71, 129.59, 128.76, 126.73, 126.59, 124.99, 124.31, 122.05, 118.08, 79.65, 78.59, 78.29, 78.17, 77.00, 74.84, 72.35, 72.29, 71.87, 71.76, 70.67, 69.79, 67.96, 67.68, 64.54, 64.44, 38.25, 36.55, 36.29, 35.24, 33.54, 33.37, 32.14, 31.68, 30.91, 28.98, 26.26, 26.17, 24.33, 24.22, 20.91, 20.54, 20.52, 14.46, 13.28, 13.15, 9.88.

HRMS (ESI) calculated for ($[M+2H]^{2+}$): m/z =802.4197; experimental = 802.4199.

Corallopyronin A intermediates

Compound **70**

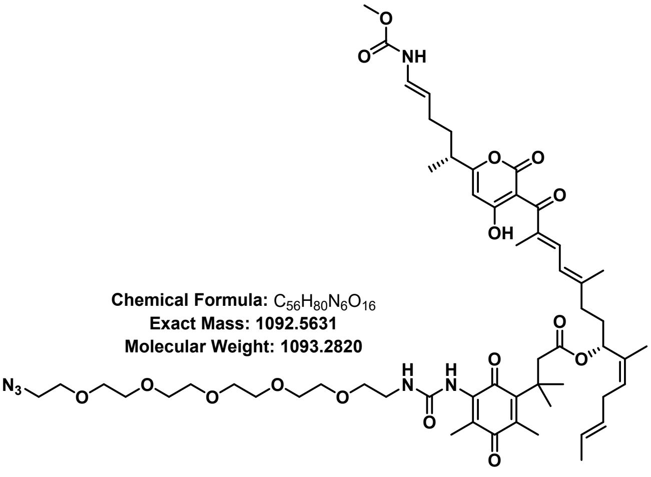

Acid **56** (10 mg, 0.017 mmol, 1.0 eq) was dissolved in dry THF (500 µL) under argon atmosphere, NMM (15 µL) was added and the flask was cooled to 0 °C. Then *iso*-butylchloroformate (1.66 µL, 0.017 mmol, 1.0 eq) was added at 0 °C and the reaction continued stirring at that temperature for one hour. The color changed from yellow to orange and corallopyronin A **35** (18.08 mg, 0.034 mmol, 2.0 eq) was added in THF (500 µL) at 0 °C. The reaction continued stirring overnight, while the thawing ice bath equilibrated the reaction steadily to ambient temperature. The next morning the solvent was removed and the reaction was purified by RP-HPLC (10-100% ACN/H_2O, 0.1% HCOOH, 220 nm). Product containing fractions were identified and lyophilized to yield **70** (13.35 mg, 0.12 mmol, 71%) as a slight beige powder. The residual, unreacted corallopyronin A **35** was re-isolated and tested for antibiotic activity, as the natural product's double bonds are prone to isomerize under basic conditions. Isomerization at C19/C20 reported and observed (e.g. by NMR), intermediate eluted as isomeric mixture as reported for free CorA **35** in Figure S18-S21.

^{1}H-NMR (700 MHz, CD_2Cl_2): δ = 7.46 (s, 1H), 7.14 (dd, J = 1.27, 11.67 Hz, 1H), 6.91 (m, 1H), 6.71 (d, J = 10.40 Hz, 1H), 6.43 (m, 3H), 6.28 (dd, J = 1.27, 11.45 Hz, 1H), 6.20 (m, 1H), 5.96 (m, 1H), 5.42 (m, 4H), 5.29 (m, 2H), 4.98 (m, 2H), 4.53 (m, 1H), 3.89 (d, J = 6.62 Hz, 1H), 3.67 (m, 3H), 3.63 (m, 8H), 3.61 (s, 4H), 3.56 (t, J = 4.73 Hz, 2H), 3.37 (m, 4H), 3.02 (m, 1H), 2.75 (m, 2H), 2.70 (m, 4H), 2.61 (m, 2H), 2.13 (s, 3H), 1.97 (s, 2H), 1.95 (s, 2H), 1.88 (s, 3H), 1.77 (s, 4H), 1.71 (s, 6H), 1.64 (m, 4H), 1.64 (m, 2H), 1.39 (d, J = 2.00 Hz, 6H, 1.23 (m, 6H), 0.94 (d, J = 6.62 Hz, 3H).

^{13}C-NMR (176 MHz, CD_2Cl_2): δ = 202.39, 191.89, 187.58, 187.02, 181.42, 176.46, 170.65, 168.91, 161.82, 160.54, 152.98, 152.60, 148.58, 140.92, 140.80, 140.12, 137.76, 137.62, 136.11, 134.32, 132.85, 132.70, 130.43, 130.39, 130.23, 130.19, 127.41, 127.20, 126.49, 126.35, 125.85, 125.81, 124.92, 123.32, 121.57, 120.91, 114.70, 109.99, 109.63, 101.51, 100.24, 99.60, 74.34, 71.09, 71.03, 70.94, 70.86, 70.76, 70.71, 70.43, 69.71, 69.62, 54.00, 52.82, 51.33, 47.70, 38.77, 38.64, 38.44, 38.38, 37.73, 37.51, 34.90, 33.68, 33.61, 31.02, 29.71, 29.37, 28.39, 27.74, 24.20, 19.21, 18.31, 18.21, 18.19, 18.16, 18.04, 17.97, 17.71, 17.66, 14.73, 13.79, 12.29, 11.14.

HRMS (ESI) calculated for ($[M+H+Na]^{2+}$): m/z = 558.2798 ; experimental = 558.2801.

Compound **71**

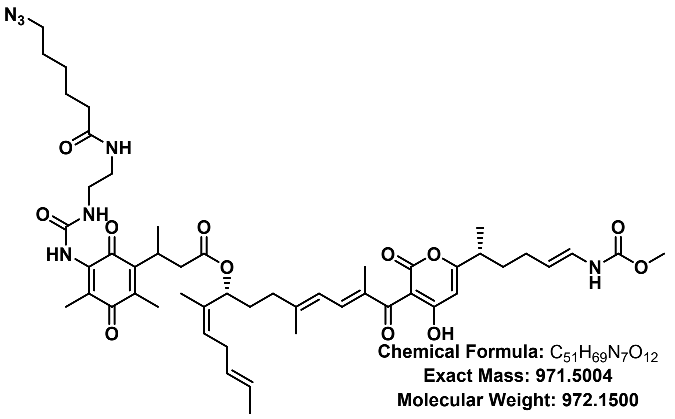

Acid **53** (10 mg, 0.021 mmol, 1.0 eq) was dissolved in dry THF (500 µL) under argon atmosphere, pyridine (5 µL) was added and the flask was cooled to 0 °C. Then *iso*-butylchloroformate (2.03 µL, 0.021 mmol, 1.0 eq) was added at 0 °C and the reaction continued stirring at that temperature for one hour. The color changed from yellow to orange and corallopyronin A **35** (22.14 mg, 0.042 mmol, 2.0 eq) was added in THF (500 µL) at 0 °C. The reaction continued stirring for 48 hours, while the thawing ice bath equilibrated the reaction steadily to ambient temperature. The next morning the solvent was removed and the reaction was purified by RP-HPLC (10-100% ACN/H_2O, 0.1% HCOOH, 220 nm). Product containing fractions were lyophilized to yield **71** (15.358 mg, 0.16 mmol, 75%) as a slight beige powder. The residual, unreacted corallopyronin A **35** was re-isolated and tested for antibiotic activity, as the double bonds are prone to isomerize under basic conditions. Isomerization at C19/C20 reported and observed (e.g. by NMR), intermediate eluted as isomeric mixture as reported for free CorA **35** in Figure S18-S21.

^{1}H-NMR (700 MHz, CD_2Cl_2): δ = 7.15 (dd, J = 1.89, 11.57 Hz, 1H), 6.45(m, 2H), 6.33 (m, 1H), 6.28 (m, 1H), 6.15 (s,1H), 5.96 (m, 1H), 5.87 (m, 1H), 5.40 (m, 5H), 5.28 (t, J = 6.73 Hz, 1H), 5.20 (t, J = 7.16 Hz, 1), 4.97 (m, 2H), 4.73 (m, 1H), 4.52 (t, J = 6.52 Hz, 1), 4.03 (m, 1H), 3.96 (d, J = 6.10 Hz, 2H), 3.67 (m, 6H), 3.26 (m, 1H), 2.75 (m, 2H), 2.68 (m, 4H), 2.59 (m, 1H), 2.42 (t, J = 7.79 Hz, 1H), 2.26 (m, 1H), 2.15 (m, 1H), 1.99 (m, 7H), 1.94 (s, 3H), 1.89 (s, 3H), 1.82 (s, 3H, 1.70 (s, 6H), 1.65 (s, 3H), 1.64 (m, 8H), 1.35 (m, 2H), 1.31 (s, 2H), 1.24 (m, 9H), 0.96 (m, 6H), 0.91 (d, J = 6.73 Hz, 4H).

^{13}C-NMR (176 MHz, CD_2Cl_2): δ = 191.36, 190.95, 171.32, 170.03, 168.31, 162.90, 162.77, 161.72, 160.00, 156.64, 154.55, 152.57, 152.42, 151.29, 140.97, 140.13, 140.03, 137.64, 136.74, 135.64, 134.48, 132.69, 131.77, 130.40, 130.32, 130.16, 126.48, 126.46, 126.37, 126.02, 125.85, 125.82, 125.74, 125.64, 124.99, 122.75, 122.64, 121.59, 121.52, 114.54, 114.23, 109.68, 101.23, 100.55, 100.24, 99.60, 98.99, 98.47, 97.87, 97.65, 83.59, 76.81, 76.72, 76.39, 76.10, 76.01, 69.72, 69.68, 69.64, 54.00, 52.80, 51.79, 51.73, 39.07, 38.77, 38.67, 38.57, 38.53, 38.45, 38.32, 38.22, 37.77, 37.51, 35.14, 34.87, 34.69, 34.34, 33.66, 31.13, 31.07, 31.01, 31.00, 30.96, 30.26, 29.88, 28.98, 28.35, 28.20, 27.75, 27.55, 27.17, 26.47, 24.81, 24.56, 19.10, 19.04, 18.95, 18.35, 18.30, 18.20, 18.17, 18.04, 17.94, 17.92, 17.69, 14.28, 12.32, 12.19, 11.16.

HRMS (ESI) calculated for ($[M+H+Na]^{2+}$): m/z = 994.4896; experimental = 994.4899.

Compound **74**

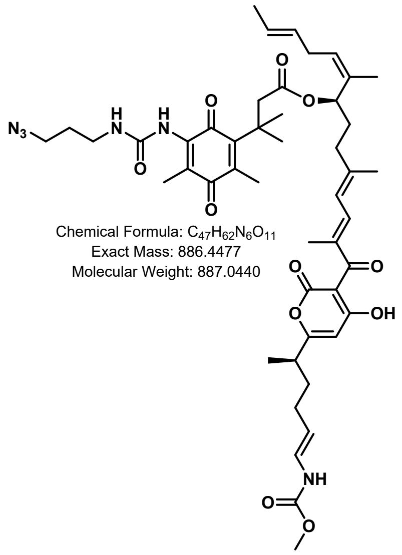

Acid **72** (11.7 mg, 0.031 mmol, 1.0 eq) was dissolved in anhydrous DCM (2.5 mL) and NMM (20 µL) and iso-butylchloroformate (4 µL, 0.031 mmol, 1.0 eq) were added in one portion at 0 °C under argon atmosphere. The reaction mixture was stirred 45 minutes at 0 °C before CorA **35** (18.0 mg, 0.031 mmol, 1.0 eq) was added dropwise in anhydrous DCM (2.5 mL) at 0 °C and the ice bath was left to thaw. Afterwards, the reaction stirred 60 minutes more and then the solvent was removed by rotary evaporation at 30 °C water bath temperature. The residue was taken up in DCM (10 mL) and washed with water and brine (2x10 mL each), dried over Na_2SO_4 and evaporated by rotary evaporation to yield **74** as a crude, yellow powder (6.1 mg, 0.007 mmol, 22%). Isomerization at C19/C20 reported and observed (e.g. by NMR), intermediate eluted as isomeric mixture as reported for free CorA **35** in Figure S18-S21.

^{1}H-NMR (500 MHz, MeOH-d_4): δ = 7.18 (m, 2H), 6.41 (m, 4H), 6.29 (t, J = 11.86 Hz, 2H), 5.95 (m, 3H), 5.41 (m, 6H), 5.23 (m, 3H), 5.03 (m, 3H), 4.51 (m, 1H), 4.08 (d, J = 6.31 Hz, 1H), 3.92 (m, 2H), 3.66 (s, 6H), 3.40 (m, 3H), 3.26 (m, 3H), 3.10 (m, 2H), 2.71 (m, 7H), 2.19 (m, 2H), 2.13 (m, 6H), 2.00 (m, 6H), 1.94 (m, 5H), 1.87 (m, 12H), 1.78 (m, 8H), 1.71 (m, 5H), 1.67 (m, 3H), 1.63 (m, 10H), 1.47 (m, 5H), 1.41 (s, 6H), 1.32 (m, 3H), 1.25 (m, 6H), 1.04 (d, J = 6.94 Hz, 2H), 0.92 (m, 4H).

^{13}C-NMR (126 MHz, MeOH-d_4): δ = 188.79, 187.69, 187.59, 172.23, 171.97, 170.06, 165.47, 156.93, 142.11, 140.96, 138.97, 138.80, 138.66, 138.18, 138.03, 137.37, 137.17, 137.01, 135.34, 131.08, 131.04, 131.01, 130.96, 127.36, 127.21, 126.96, 126.67, 126.60, 126.51, 126.38, 126.28, 126.24, 126.18, 126.10, 125.64, 125.44, 125.24, 124.94, 123.24, 123.04, 122.38, 110.75, 110.71, 110.63, 103.02, 102.49, 100.82, 100.42, 85.10, 84.87, 78.06, 77.93,

77.75, 77.61, 75.71, 74.83, 69.67, 69.64, 69.59, 52.82, 50.35, 50.22, 50.19, 47.99, 43.23, 40.42, 39.72, 39.62, 39.43, 39.31, 38.98, 38.84, 38.67, 38.41, 38.13, 36.07, 35.92, 35.83, 34.83, 34.34, 31.84, 31.70, 31.66, 31.64, 31.63, 31.35, 31.27, 31.23, 30.53, 30.47, 30.32, 30.15, 30.00, 29.89, 29.79, 29.66, 29.50, 29.22, 29.11, 28.69, 28.65, 27.66, 27.39, 25.14, 19.51, 19.30, 19.23, 18.52, 18.44, 18.37, 18.29, 18.23, 17.87, 17.56, 14.90, 14.79, 14.74, 14.68, 14.52, 14.38, 12.96, 12.84, 12.72, 12.68, 12.30, 12.10, 12.05, 11.87, 10.96.

Mono and dicatechol rifamycin conjugates

Compound **4**

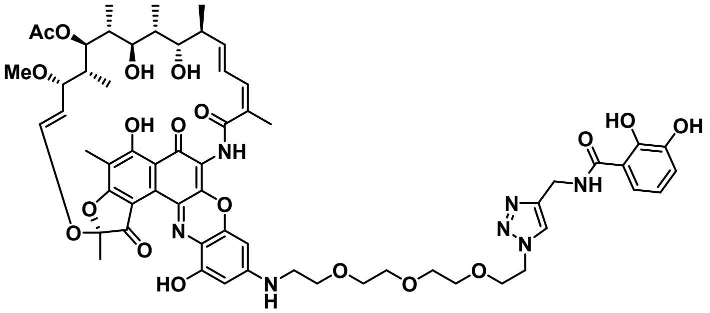

Chemical Formula: $C_{61}H_{73}N_7O_{19}$
Exact Mass: 1207,4961
Molecular Weight: 1208,2850

Alkyne **39** (11.9 mg, 0.062 mmol, 3.0 eq) and N_3-PEG$_3$-NH$_2$ (15.9 mg, 0.73 mmol, 3.5 eq) were dissolved in DMSO (200 µL). Sodium ascorbate (4.11 mg, 0.021 mmol, 1.0 eq), $CuSO_4$ (3.31 mg, 0.021 mmol, 1.0 eq) and TBTA (2.20 mg, 0.004, 0.2 eq) were premixed in 1x PBS (pH 7.4, 200 µL) and added to the reaction mixture. The reaction mixture was stirred 1 hour at 25 °C. Upon complete consumption of the alkyne (LCMS), the mixture was diluted with water (10 mL) and freeze-dried overnight. The residue was dissolved in dry ethyl acetate/MeOH and filtered through a syringe filter and concentrated *in vacuo*. The brown solid was taken up in anhydrous DMSO (200 µL) and added dropwise to fluoro rifamycin **2** (17.0 mg, 0.021 mmol, 1.0 eq) in anhydrous THF (5 mL) under argon atmosphere at 0 °C. The reaction stirred at 0 °C for 5 minutes and continued stirring at 25 °C for an hours. The addition of anhydrous DIPEA (30 µL) and warming to maximum 45 °C drove the reaction to completion. The solvent was removed by rotary evaporation and the residual liquid was purified by RP-HPLC (60-90% ACN/H_2O, 1% AcOH, 220 nm,). The product containing fractions were identified by LCMS and lyophilized overnight to yield pure title compound **4** as a blue solid (7.42 mg, 0.006 mmol, 30% over two steps).

^{1}H-NMR (700 MHz, Tol-d8, AcOH-d4, ACN-d$_3$): δ = 13.24 (s, 1H), 9.52 (s, 1H), 9.22-9.09 (m, 3H), 7.91 (m, 1H), 7.75 (d, J = 11.65 Hz; 1H), 7.58 (d, J = 8.59 Hz, 1H), 7.17 (m, 1H), 6.88 (m, 1H), 6.67 (m, 1H), 6.33 (m, 1H), 6.27 (d, J = 12.06 Hz, 2H), 6.03 (m, 1H), 5.74 (m, 2H), 5.38

(q, J = 7.56, 13.08 Hz, 2H), 5.21 (t, J = 9.81 Hz, 1H), 5.08 (m, 2H), 4.70 (m, 2H), 4.61 (m, 1H), 4.37 (m, 1H), 4.30 (m, 2H), 3.86 (m, 1H), 3.61 (m, 3H), 3.46 (m, 6H), 3.38 (m, 5H), 3.08 (m, 4H), 2.95 (m, 5H), 2.91 (m, 1H), 2.29 (s, 5H), 2.22 (m, 5H), 1.94 m, 4H), 1.78 (m, 12H), 1.61 (m, 5H), 1.26 (m, 4H), 1.06 (d, J = 7.15 Hz, 4H), 0.99 (d, J = 7.56 Hz, 3H), 0.93 (m, 2H), 0.73 (m, 3H), 0.62 (m, 5H).

^{13}C-NMR (176 MHz, Toluene-d_8, AcOH-d_4, ACN-d_3) δ = 185.23, 181.78, 172.37, 170.72, 170.20, 166.62, 147.22, 144.14, 142.00, 137.32, 137.26, 137.04, 137.00, 132.80, 131.07, 130.56, 128.28, 128.26, 128.23, 127.37, 127.36, 125.10, 124.49, 123.88, 119.19, 118.04, 116.67, 114.91, 107.74, 76.72, 73.03, 72.75, 70.40, 70.29, 70.25, 69.26, 55.85, 49.74, 37.33, 35.13, 32.95, 21.85, 20.72, 16.81, 11.30, 9.40, 7.43.

HRMS (ESI) calculated for ([M+H]$^+$): m/z = 1208.5034; experimental = 1208.5025.

Compound **5a** and **5**

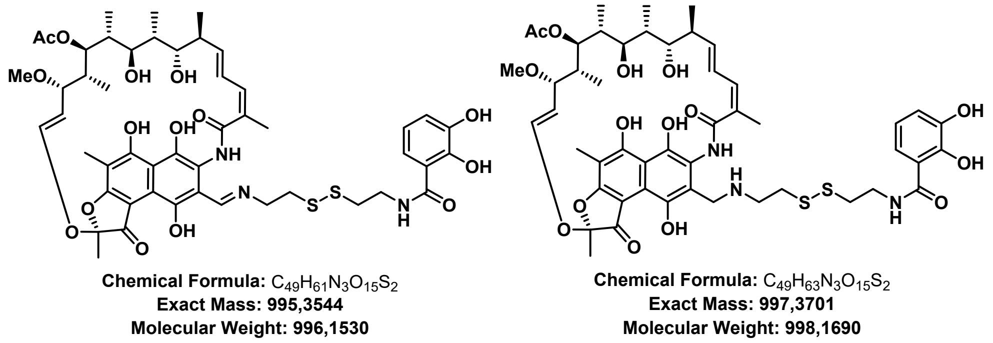

3-formyl rifamycin **27** (50 mg, 0.069 mmol, 1.0) was dissolved in anhydrous THF (20 mL) under argon atmosphere and amine **40** (19.9 mg, 0.069 mmol 1.0 eq) was added as a solution in THF (5 mL) with TEA (30 µL) at 0 °C. The ice bath was removed and the color of the solution changed from red to purple over the course of 1 hour at ambient temperature. The reaction mixture was diluted with DCM (100 mL) and washed with 1 M HCl (1x 75 mL), water/brine (1:1, 2x100 mL) and dried over Na_2SO_4. The solvent was removed in vacuo to yield pure imine **5a** (61.39 mg, 0.062 mmol, 90%) as a red-purple solid. A small fraction of the imine **5a** was directly tested for biological activity in MIC assays and stored under argon in dry DMSO at - 20 °C in the dark. The rest was dissolved in anhydrous THF and $NaBH(OAc)_3$ (21.91 mg, 0.103 mol, 1.5 eq) was added in one portion at 0 °C under argon. The reaction stirred 30 minutes at 30 °C and 30 minutes at ambient temperature. Then the reaction was diluted with DCM (120 mL) and washed with 1 M HCl, water and brine (each 2x100 mL). The organic phase was dried over Na_2SO_4 and the solvent was removed *in vacuo* to yield crude amine **5** as a red solid (38.99 mg, 0.039 mmol, 57% over 2 steps).

5a:

^{1}H-NMR (700 MHz, CD_3CN): δ = 12.67 (s, 1H), 12.23 (s, 1H), 10.57 (s, 1H), 6.54 (dd, J = 11.59 Hz, 1H), 6.47 (m, 1H), 6.32 (d, J = 12.35 Hz, 1H), 6.05 (dd, J = 5.04, 15.37 Hz, 1H), 5.10 (m, 1H), 5.01 (d, J = 10.08 Hz, 1H), 3.74 (d, J = 9.58 Hz, 1H), 3.64 (m, 6H), 3.43 (d, J = 8.07 Hz, 1H), 3.10 (m 1H), 3.02 (m, 2H), 2.97 (s, 3H), 2.33 (m, 1H), 2.22 (s, 3H), 2.06 (s, 3H), 2.00 (s, 3H), 1.80 (m, 9H), 1.39 (s, 3H), 1.26 (m, 1H), 1.15 (m, 1H), 0.95 (d, J = 7.31 Hz, 3H), 0.85 (d, J = 7.31 Hz, 3H), 0.61 (d, J = 6.55 Hz, 3H), -0.39 (d, J = 6.55 Hz, 3H).

5

^{1}H-NMR (700 MHz, DMSO-d_6): δ = 12.79 (m, 1H), 9.37 (m, 1H), 8.61 (m, 2H), 8.47 (m, 1H), 7.81 (m, 1H), 7.70 (m, 1H), 7.28 (m, 2H), 6.25 (m, 1H), 6.17 (m, 1H), 6.07 (m, 1H), 5.91 (dd, J = 6.52, 16.76 Hz, 1H), 5.08 (m, 1H), 5.00 (m, 2H), 4.90 (m, 1H), 3.28 (m, 2H), 3.15 (m, 1H), 3.09 (m, 1H), 2.89 (m, 4H), 2.73 (s, 1H), 2.02 (m, 3H), 1.97 (m, 5H), 1.91 (m, 3H), 1.89 (m, 2H), 1.64 (s, 1H), 1.35 (s, 1H), 1.23 (bs, 3H), 0.93 (m, 6H), 0.85 (d, J = 7.45 Hz, 3H), 0.81 (d, J = 7.08 Hz, 3H), 0.27 (m, 1H), -0.03 (m, 1H), -0.34 (d, J = 6.89 Hz, 1H).

HRMS (ESI) calculated for ($[M+H]^+$): m/z = 998.3774; experimental =998.3782.

Compound **6a** and **6**

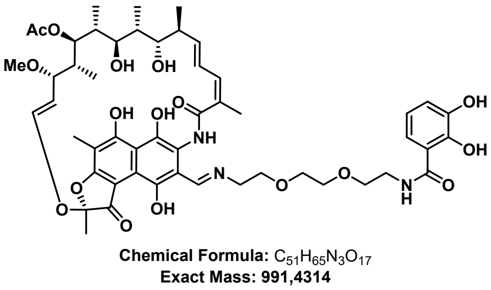

Chemical Formula: $C_{51}H_{65}N_3O_{17}$
Exact Mass: 991,4314
Molecular Weight: 992,0850

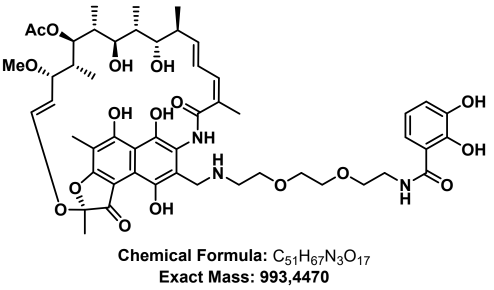

Chemical Formula: $C_{51}H_{67}N_3O_{17}$
Exact Mass: 993,4470
Molecular Weight: 994,1010

3-formyl rifamycin **27** (50 mg, 0.069 mmol, 1.0) was dissolved in anhydrous THF (20 mL) under argon atmosphere and the amine **41** (19.59 mg, 0.069 mmol 1.0 eq) was added as a solution in THF (5 mL) with TEA (30 µL) at 0 °C. The ice bath was removed and the color of the solution changed from red to purple over the course of 1 hour at ambient temperature. The reaction was diluted with DCM (100 mL) and washed with 1 M HCl (1x 75 mL), water/brine (1:1, 2x100 mL) and dried over Na_2SO_4. The solvent was removed *in vacuo* to yield pure imine **6a** (61.39 mg, 0.062 mmol, 90%) as a red-purple solid. A small fraction of the imine **6a** was directly tested

for biological activity in MIC assays and stored under argon in dry DMSO at - 20 °C in the dark. The rest was dissolved in anhydrous THF and $NaBH(OAc)_3$ (21.91 mg, 0.103 mol, 1.5 eq) was added in one portion at 0 °C under argon. The reaction stirred 30 minutes at 30 °C and 30 minutes at ambient temperature. Then the reaction was diluted with DCM (120 mL) and washed with 1 M HCl, water and brine (each 2x100 mL). The organic phase was dried over Na_2SO_4 and the solvent was removed *in vacuo* to yield crude title compound **6** as a red solid (54.2 mg, 0.055 mmol, 79% over 2 steps).

^{1}H-NMR (700 MHz, ACN-d_3): δ = 15.11 (s, 1H), 8.72 (s, 2H), 8.25 (m, 2H), 7.94 (m, 1H), 7.81 (m, 2H), 7.47 (d, J = 6.82 Hz, 1H), 7.41 (m, 2H), 6.71 (s, 1H), 6.66 (s, 1H), 5.89 (d, J = 9.97 Hz, 1H), 5.32 (s, 1H), 5.26 (m, 2H), 5.23 (s, 1H), 5.02 (d, J = 7.61 Hz, 1H), 3.78 (s, 1H), 3.72 (m, 2H), 3.63 (m, 2H), 3.24 (m, 2H), 3.05 (m, 4H), 2.98 (m, 1H), 2.51 (m, 1H), 2.34 (m, 5H), 2.32 (m, 2H), 2.29 (m, 4H), 2.19 (m, 2H), 1.79 (m, 1H), 1.34 (d, J = 7.61 Hz, 3), 1.27 (d, J = 5.72 Hz, 3H), 1.13 (d, J = 7.18 Hz, 3H), 0.87 (m, 6H).

^{13}C-NMR (176 MHz, ACN-d_3): δ = 189.30, 178.29, 171.84, 167.53, 158.01, 149.32, 148.69, 146.19, 142.56, 140.56, 139.80, 139.55, 138.59, 135.70, 129.64, 129.43, 129.25, 129.12, 128.01, 125.70, 125.05, 124.68, 121.81, 120.81, 120.40, 112.50, 112.37, 111.32, 108.58, 107.41, 71.58, 69.88, 67.35, 50.52, 50.31, 50.22, 45.92, 42.37, 36.73, 31.41, 28.97, 21.48, 21.30, 21.23, 20.08, 19.26, 8.73, 8.69, 1.88.

HRMS (ESI) calculated for ($[M+H]^+$): m/z = 994.4543; experimental = 994.4556.

Compound **3**

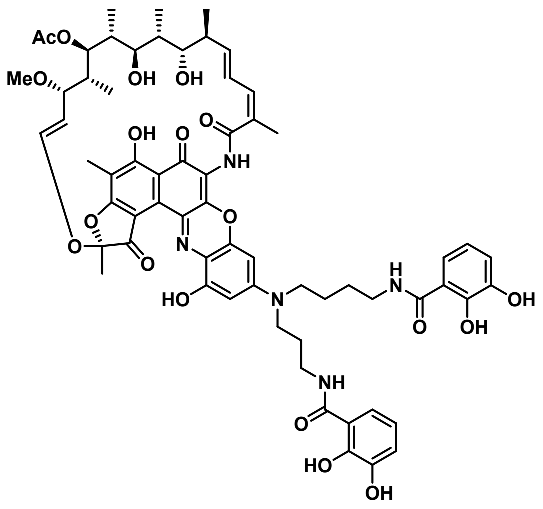

Chemical Formula: $C_{64}H_{73}N_5O_{19}$
Exact Mass: 1215,4900
Molecular Weight: 1216,3040

2 (30 mg, 0.037 mmol, 1.0 eq) was dissolved in dry THF (1 mL) under argon atmosphere and crude dicatechol **42** (30.6 mg, 0.073 mmol, 2.0 eq) was added in THF/DMSO (1:1, 1mL) dropwise at 0 °C over 10 minutes. Then DIPEA (100 µL) and pyridine (100 µL) were added and the reaction was warmed to 45 °C and the color changed from red to blue. The reaction continued stirring overnight and the solvent was removed by rotary evaporation. The residue was dried, filtered over a syringe filter and purified by RP-HPLC (60-100% H_2O/ACN, 0.1% AcOH, 220nm). Product containing fractions were lyophilized overnight to yield dicatechol rifamycin **3** (24.6mg, 0.02 mmol, 55%) as a blue solid.

^{1}H-NMR (700 MHz, CD_2Cl_2): δ = 9.46 (t, J = 2.89 Hz, 1H), 7.45 (d, J = 7.56 Hz, 2H), 7.40 (t, J = 7.65 Hz, 2H), 7.35 (d, J = 7.65 Hz, 1H), 7.01 (dd, J = 7.96, 26.62 Hz, 1H), 6.94 (t, J = 7.65 Hz, 1H), 6.76 (m, 1H), 6.70 (s, 1H), 6.59 (s, 1H), 6.29 (d, 1H), 5.04 (s, 2H), 5.00 m, 1H), 4.10 d, J = 7.21 Hz, 1H), 3.57 (m, 9H), 3.07 (m, 2H), 2.91 (d, J = 2.54 Hz, 2H), 2.51 (s, 3H), 2.30 (m, 5H), 2.23 (s, 3H), 2.10 (s, 2H), 2.01 (bs, 3H), 1.97 (s, 3H), 1.75 (m, 4H), 1.60 (m, 3H), 1.56 (s, 8H), 0.89 (m, 9H), 0.83 (m, 6H).

DEPT (176 MHz, DMSO-d_6): δ = 137.65, 136.81, 135.86, 134.95, 132.08, 131.96, 129.01, 128.44, 126.84, 124.56, 122.30, 118.33, 78.84, 78.42, 77.24, 75.09, 72.60, 72.54, 72.11, 70.91, 70.04, 67.92, 64.78, 38.49, 36.54, 35.48, 33.80, 31.93, 31.15, 26.51, 24.58, 20.79, 20.26, 14.71, 13.40, 10.26, 10.13.

HRMS (ESI) calculated for ($[M+H]^+$): m/z = 1216.4973; experimental = 1216.4941.

DOTAM and DFO RNAP-I conjugates

Compound **18**

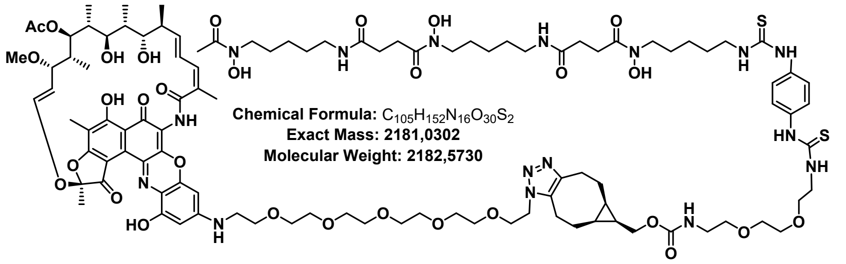

Red fluoro rifamycin **2** (7.5 mg, 0.009 mmol, 1.0 eq) and N_3-PEG-NH_2 (5.5 mg, 0.018 mmol, 2.0 eq) were mixed in anhydrous THF (5 mL) and DIPEA (20 µL) was added. The reaction stirred for four hours at ambient temperature. Then the solvent was removed by rotary evaporation and the residue was dissolved in DCM (20 mL). The organic phase was washed with 1 M HCl (2x10 mL) and the organic phase was dried over Na_2SO_4. The solvent was removed *in vacuo* and the residue was dried under reduced pressure to yield crude azide **18a** as a blue solid (quant.). Then azide **18a** (9.6 mg, 0.009 mmol, 1.0 eq) and strained alkyne **47** (14.97 mg, 0.014 mmol, 1.6 eq) were weight in 1.5 mL tubes and then dissolved in degassed mixture of ACN:H_2O (1:1, 300 µL each). The compounds were added together under argon atmosphere and continued stirring for 30 hours at 24 °C. The orange solution was filtered and purified by RP-HPLC (15-98-100% ACN/H_2O, 0.1% HCOOH, 220 nm, collect all.). Product containing fractions were lyophilized to yield **18** (15.55 mg, 0.007 mmol, 82%) as a beige solid. The compound eluted as a diastereomeric mixture in one peak from the HPLC, just one isomer is depicted here.

^{1}H-NMR (600 MHz, DMSO-d$_6$): δ = 9.62 (m, 4H), 9.16 (m, 1H), 8.41 (m, 1H), 7.78 (m, 4H), 7.65 (m, 2H), 7.34 (m, 4H), 6.47 (m, 2H), 6.37 (m, 2H), 6.21 (m, 1H), 4.63 (m, 1H), 4.57 (m, 1H), 4.48 (m, 1H), 4.37 (m, 1H), 4.09 (m, 4H), 4.01 (m, 2H), 3.63 (t, J = 5.45 Hz, 6H), 3.58 (m, 10H), 3.54 (m, 20H), 3.51 (m, 20H), 3.38 (t, J = 5.45 Hz, 10H), 3.17 (s, 12H), 3.00 (q, J = 5.45, 12.76 Hz, 4H), 2.58 (m, 4H), 2.27 (m, 4H), 2.14 (m, 5H), 2.04 (m, 12H), 2.00 (m, 4H), 1.97 (s, 3H), 1.55 (m, 6H), 1.38 (m, 4H), 1.24 (m, 6H), 0.92 (m, 16H), 0.79 (m, 8H), 0.74 (m, 8H).

^{13}C-NMR (151 MHz, DMSO-d$_6$): δ = 202.07, 195.55, 174.20, 171.77, 170.38, 105.54, 104.53, 86.50, 77.85, 73.59, 70.29, 70.27, 70.24, 70.14, 70.00, 69.70, 69.15, 50.44, 49.06, 47.61, 46.32, 43.55, 38.88, 35.36, 31.76, 31.50, 30.36, 29.48, 29.29, 29.17, 28.04, 26.49, 24.07, 23.96, 23.34, 22.56, 21.18, 20.82, 16.96, 14.43, 12.85, 7.79, 6.84.

DEPT (151 MHz, DMSO-d_6): δ = 86.51, 77.86, 73.60, 70.29, 70.28, 70.25, 70.22, 70.14, 70.12, 69.95, 69.70, 69.69, 69.15, 50.44, 50.41, 49.07, 47.62, 46.33, 43.55, 38.90, 35.36, 31.50, 30.36, 29.48, 29.29, 28.04, 26.49, 23.97, 23.34, 21.18, 20.82, 12.85, 7.79, 6.84.

HRMS (ESI) calculated for ($[M+3H]^{3+}$): m/z = 1091.5223; experimental = 1091.5236.

Compound **19**

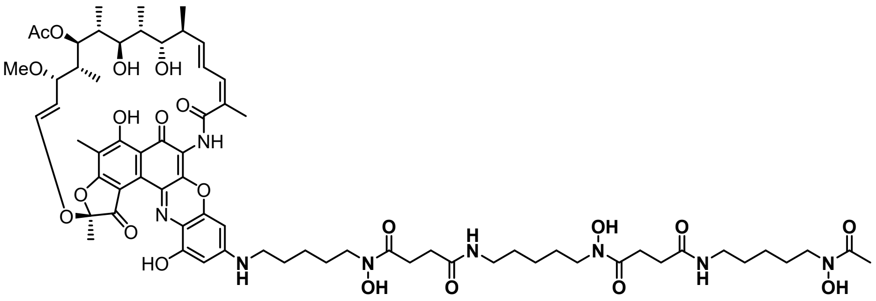

Chemical Formula: $C_{68}H_{94}N_8O_{21}$
Exact Mass: 1358,6534
Molecular Weight: 1359,5350

2 (50.0 mg, 0.061 mmol, 1.0) was dissolved in anhydrous THF (35 mL) and TEA (0.5 mL) was added under argon atmosphere. DFO (68.47 mg, 0.122 mmol, 2 eq) was added in DMF (20 mL) and dissolved with gentle heating with a heat gun. The clear solution was added quickly to the solution of **2**. The solution was heated to 45 °C overnight and the color changed from blood red to intense blue. Then the solvent was removed by rotary evaporation. The residue was taken up in MeOH/ACN (5 mL) and purified by RP-HPLC (C18, 220 nm, 15-85% ACN/H_2O, collect all). Product containing fractions were lyophilized to dryness to yield **19** (69.5, 0.051 mmol, 84%) as a dark blue solid.

^{1}H-NMR (600 MHz, DMSO-d_6): δ = 16.61 (s, 1H), 16.41 (s, 1H), 9.63 (m, 4H), 9.14 (m, 1H), 8.61 (m, 1H), 8.34 (m, 1H), 7.77, 6.80 (s, 4H), 6.80 (m, 1H), 6.39 (m, 4H), 6.12 (m, 1H), 6.02 (m, 1H)), 5.78 (m, 2H), 5.18 (m, 2H), 4.97 (m, 1H), 4.85 (m, 2H), 4.01 (bs, 1H), 3.59 (m, 1H), 3.47 (m, 10H), 3.19 (m, 3H), 3.02 (m, 10H), 2.86 (bs, 2H), 2.59 (m, 5H), 2.28 (m, 6H), 2.21 (m, 2H), 2.12 (m, 2H), 1.97 (m, 12H), 1.75 (m, 2H), 1.66 (m, 2H), 1.59 (m, 6H), 1.50 (m, 9H), 1.38 (m, 6H), 1.22 (m, 6H), 0.85 (m, 8H), 0.67 (m, 3H), -0.08 (m, 2H), -0.42 (m, 2H).

^{13}C-NMR (151 MHz, DMSO-d_6): δ = 192.27, 181.12, 180.78, 173.78, 172.00, 171.96, 171.28, 170.47, 170.12, 169.86, 169.25, 168.88, 158.02, 143.82, 143.54, 141.57, 131.72, 130.98, 129.16, 128.11, 125.84, 120.25, 118.81, 113.27, 112.18, 111.05, 109.71, 108.21, 107.35, 106.15, 104.45, 92.67, 77.94, 77.08, 75.67, 75.58, 72.56, 72.18, 55.78, 54.04, 48.58, 47.06,

46.98, 46.77, 43.00, 41.87, 41.44, 39.52, 38.42, 35.49, 32.23, 30.91, 29.84, 28.81, 27.99, 27.55, 26.02, 23.80, 23.77, 23.49, 22.60, 22.30, 20.94, 20.53, 20.34, 19.92, 18.28, 16.35, 12.13, 10.62, 8.98, 7.52, 7.40.

DEPT (151 MHz, DMSO-d_6): δ = 185.17, 185.01, 183.56, 183.56, 182.96, 182.88, 182.82, 178.08, 176.68, 175.41, 144.52, 144.45, 144.38, 139.59, 139.59, 139.59, 138.01, 138.01, 136.74, 135.98, 135.36, 135.35, 135.35, 134.82, 133.21, 130.56, 129.94, 129.94, 129.88, 129.87, 129.87, 129.80, 126.44, 126.39, 126.22, 125.52, 100.00, 76.18, 73.77, 73.69, 73.61, 70.33, 70.20, 70.20, 70.13, 70.06, 70.06, 69.45, 69.43, 69.43, 65.25, 65.18, 64.65, 64.64, 64.64, 63.99, 63.25, 61.98, 61.98, 60.41, 55.55, 55.55, 21.91, 17.13, 16.43.

HRMS (ESI) calculated for ($[M+2H]^{2+}$): m/z = 680.3340; experimental = 680.3349.

Compound **20**

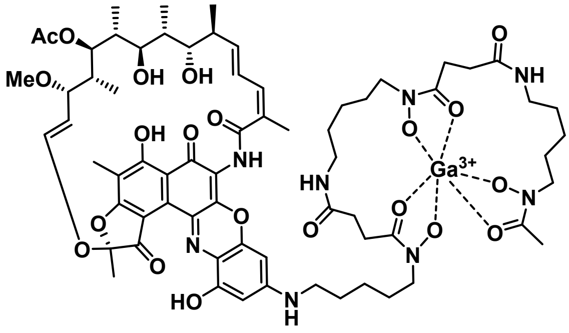

Chemical Formula: $C_{68}H_{91}GaN_8O_{21}^{3+}$
Exact Mass: 1424,5538
Molecular Weight: 1426,2324

Complex **20** was synthetized according to previously established conditions in Peukert and Langer et al.[6] **19** (5 mg, 0.004mmol, 1.0 eq) was dissolved in ddH_2O/ACN mixture (1:1, 200 µL) and $GaCl_3$ (0.71 mg, 0.004 mmol, 1.0 eq) was added, dissolved in NaOAc buffer (200 µL, pH 4.5). The reaction stirred overnight at room temperature, was then acidified with AcOH (200 µL), immediately diluted with ddH_2O and lyophilized to yield a blue solid as **20** (5.23 mg, 0.004 mmol, quant.).

^{1}H-NMR (700 MHz, MeOH-d_4): δ = 6.87 (m, 1H), 6.40 (m, 2H), 6.28 (d, J = 11.91 Hz, 1H), 6.22 (m, 2H), 5.01 (m, 2H), 4.58 (s, 5H), 3.90 (m, 2H), 3.71 (d, J = 8.00 Hz, 1H), 3.55 (m, 5H), 3.12 (m, 4H), 2.98 (m, 5H), 2.81 (m, 4H), 2.46 (m, 1H), 2.36 (m, 3H), 2.27 (bs, 3H), 2.16 (bs, 5H), 2.11 (s, 3H), 1.98 (bs, 6H), 1.78 (bs, 5H), 1.72 (m, 7H), 1.62 (m, 6H), 1.50 (m, 11H), 1.32 (m, 10), 0.93 (m,12H), 0.78 (m, 4H), 0.69 (m, 2H), 0.05 (m, 3H), -0.32 (m, 3H).

HRMS (ESI) calculated for ([M+H]+): m/z =1425.5610; experimental =1425.5630, calculated for ([M+Na]+): m/z =1447.5430; experimental = 1447.5463, calculated for ([M+2H]2+): m/z = 713.2842; experimental = 713.2859.

Compound **21**

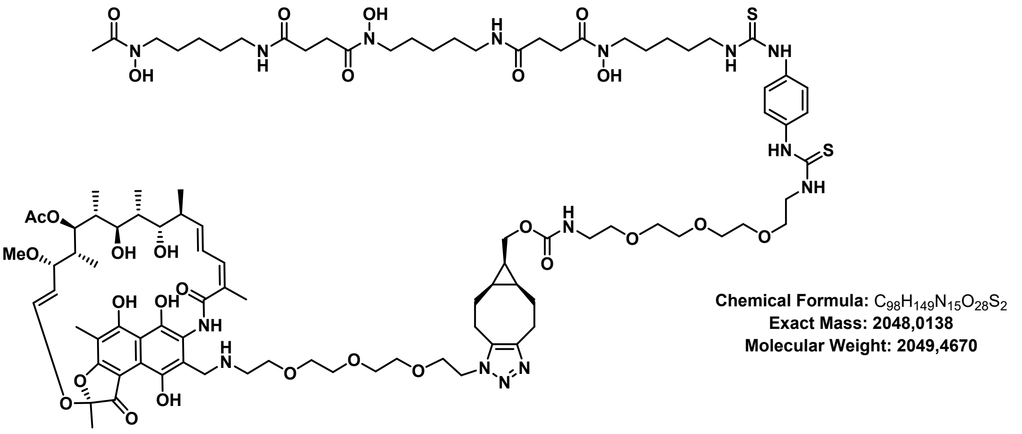

Strained alkyne **47** (20 mg, 0.019 mmol, 1.0 eq) was dissolved in DMSO (1 mL) and the azide **62** (20.67 mg, 0.022 mmol, 1.2 eq) was added dissolved in MeOH (10 mL). The reaction continued stirring for 18 hours at 23 °C. Upon complete consumption of the strained alkyne, DCM was added (30 mL), brine (20 mL) and the organic phases were separated. The organic phase was washed trice with a brine/ddH2O mixture (1:1), dried over Na_2SO_4 and the solvent was removed by rotary evaporation. The residue was purified by RP-HPLC (15-95% ACN/H_2O 0.1% HCOOH, 220 nm, collect all). The product containing fractions were lyophilized to dryness to yield **21** as a yellow solid (21.3 mg, 0.011 mmol, 57%). The compound eluted as a diastereomeric mixture in one peak from the HPLC, just one isomer is depicted here.

^{1}H-NMR (700 MHz, DMSO-d_6): δ = 9.62 (m, 4H), 9.39 (m, 1H), 7.77 (m, 2H), 7.63 (m, 1H), 7.33 (q, J = 8.66, 13.66 Hz, 4H), 7.23 (m 1H), 7.08 (m, 1H), 5.85 (m, 1H), 5.16 (m, 1H), 4.36 (m, 1H), 4.03 (m, 2H), 3.73 (m, 2H), 3.62 (m, 2H), 3.54 (m, 6H), 3.46 (m, 10H), 3.40 (m, 2H), 3.30 (m, 2H), 3.12 (m, 2H), 2.99 (q, J = 6-67, 12.66 Hz, 4H), 2.57 (q, J = 9.66, 14.66 Hz, 4H), 2.27 (q, J = 9.66, 15.33 Hz, 4H), 2.06 (m, 2H), 1.96 (s, 3H), 1.86 (m, 1H), 1.74 (m, 2H), 1.65 (m, 1H), 1.53 (m, 6H), 1.49 (m, 6H), 1.37 (m, 5H), 1.21 (m, 10H), 0.91 (m, 4H), 0.85 (m, 2H), 0.71 (m, 2H).

^{13}C-NMR (176 MHz, DMSO-d$_6$): δ = 201.25, 197.62, 194.57, 193.73, 193.51, 182.45, 180.51, 176.36, 173.86, 171.30, 170.72, 170.55, 170.13, 156.44, 155.22, 155.06, 144.93, 143.14, 133.71, 132.21, 130.46, 126.43, 123.34, 115.52, 75.93, 73.47, 70.77, 69.75, 69.53, 69.16, 68.58, 67.02, 66.67, 66.60, 66.20, 65.76, 61.43, 47.15, 46.78, 43.56, 40.34, 40.02, 39.52, 38.41, 36.57, 33.96, 29.89, 29.19, 28.81, 28.21, 27.56, 26.02, 25.35, 23.49, 22.22, 21.27, 20.86, 20.35, 19.17, 17.31, 16.44, 10.92, 10.17, 9.83, -15.18.

HRMS (ESI) calculated for ($[M+3H]^{3+}$): m/z = 683.6785; experimental = 683.6794.

Compound **23**

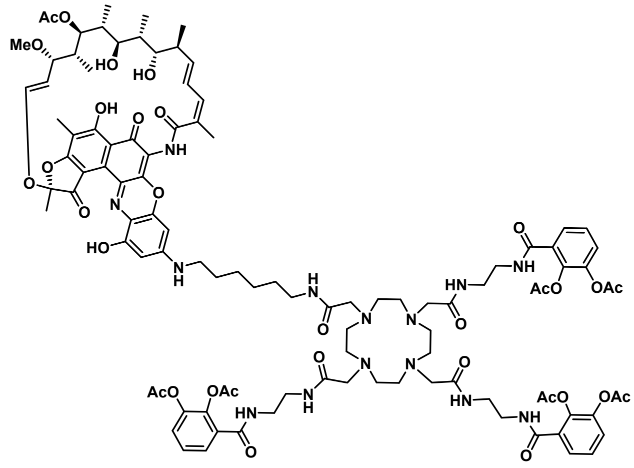

Chemical Formula: $C_{104}H_{130}N_{14}O_{32}$
Exact Mass: 2086,8976
Molecular Weight: 2088,2500

Acid **7** (10 mg, 0.008 mmol, 1.0 eq) was dissolved in anhydrous THF (1 mL), DMF (100 µL), NMM (100 µL) was added under argon atmosphere and the reaction was cooled to 0 °C, before iso-butyl chloroformate (0.81 µL, 0.008 mmol, 1.0 eq) was added and the reaction went turbid instantly. The reaction stirred 5 minutes at 0 °C and then 30 minutes at 24 °C. Then amine **22** (8.45 mg, 0.009 mmol, 1.1 eq) was added dropwise at 0 °C, dissolved in anhydrous THF (1 mL) and basified with NMM (100 µL) before addition. The reaction continued stirring at 0 °C for 5 minutes and then for 30 minutes at 24 °C. Then the reaction was quenched with AcOH (200 µL) and the THF was removed *in vacuo* at 30 °C. The residual solution was diluted and purified by RP-HPLC to yield **23** as a blue solid (13.91 mg, 0.007 mmol, 79%).

^{1}H-NMR (700 MHz, MeOH-d_4): δ = 7.47 (m, 3H), 7.32 (m, 6H), 7.14 (m, 1H), 6.86 (m, 1H), 6.38 (d, J = 10.46 Hz, 1H), 6.32 (m, 1H), 6.26 (d, J = 12.79 Hz, 1H), 6.19 (m,, 2H), 3.69 (m, 1H), 3.47 (m, 10H), 3.40 (m, 10H), 3.18 (m, 2H), 3.08 (m, 1H), 2.98 (m, 10H), 2.86 (m, 8H), 2.28 (s, 9H), 2.25 (s, 9H), 2.10 (m, 2H), 1.99 (s, 2H), 1.96 (m, 5H), 1.93 (m, 1H), 1.78 (m, 3H), 1.61 (m, 4H), 1.50 (m, 4H), 1.29 (m, 10H), 1.08 (m, 2H), 0.98 (m, 1H), 0.94 (m, 3H), 0.90 (m, 6H), 0.82 (m, 2H), 0.78 (m, 2H), 0.03 (d, J = 5.81 Hz, 3H), -0.34 (d, J = 5.18 Hz, 3H).

HRMS (ESI) calculated for ($[M+3H]^{3+}$): m/z = 1044.4561; experimental =1044.4570, calculated for ($[M+4H]^{4+}$): m/z = 696.9756; experimental =696.9752.

Compound **24**

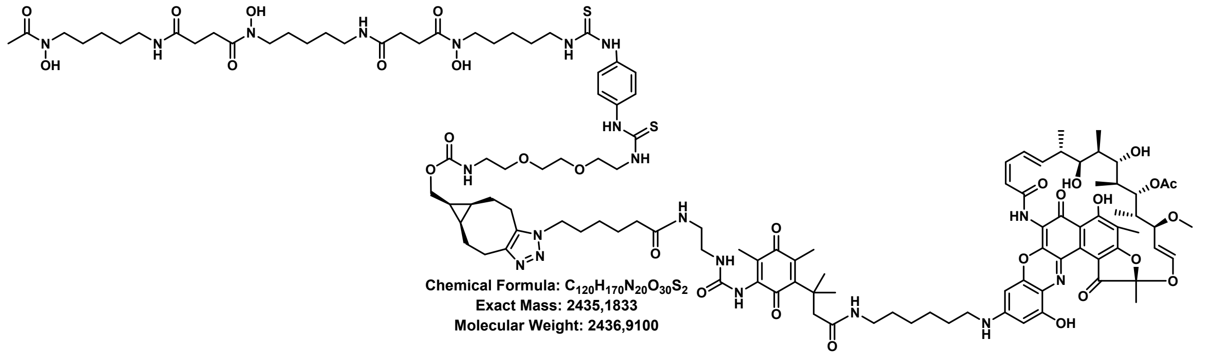

The strained alkyne **47** (10 mg, 0.009 mmol, 1.0 eq) was dissolved in anhydrous MeOH (7 mL) and the azide **63** (14.67 mg, 0.011 mmol, 1.0 eq) was added in MeOH (8 mL) under argon atmosphere. The blue solution continued stirring overnight and the reaction progress was monitored by LCMS. The solvent was removed by rotary evaporation and was purified by RP-HPLC (15%-100% ACN/H_2O 220 nm, collect all). The product containing fractions were lyophilized to dryness to yield **24** (18.24 mg, 0.007 mmol, 81%) as a blue solid. The compound was obtained as a mixture of 1,4 / 1,5 isomer that eluted as one peak from the HPLC. The compound eluted as a diastereomeric mixture in one peak from the HPLC, just one isomer is depicted here.

^{1}H NMR (700 MHz, DMSO-d_6): δ = 9.63 (m, 8H), 9.47 (m, 2H), 7.83 (m, 1H), 7.77 (m, 6H), 7.71 (m, 2H), 7.66 (m, 2H), 7.34 (m, 12H), 7.15 (m, 5H), 4.99 (dd, J = 2.61 Hz, 7.45 Hz, 1H), 4.94 (dd, J = 3.35, 11.36 Hz, 1H), 4.19 (m, 1H), 4.05 (m, 6H), 3.63 (m, 6H), 3.54 (m, 20H), 3.46 (m, 24H), 3.40 (t, J = 6.52 Hz, 10H), 3.11 (q, J = 4.84, 11.73 Hz, 8H), 3.00 (q, J = 6.85, 12.85 Hz, 12H), 2.58 (q, J = 7.08, 12.85 Hz, 12H), 2.27 (q, J = 5.96, 11.36 Hz, 12H), 2.13 (s, 4H), 1.99 (s, 3H), 1.96 (m, 14H), 1.85 (m, 8H), 1.53 (m, 12H), 1.49 (m, 12H), 1.38 (m, 12H), 1.25 (m, 6H), 1.22 (m, 10H), 1.12 (m, 4H), 0.87 (m, 4H), 0.75 (m, 2H).

^{13}C-NMR (176 MHz, DMSO-d$_6$): δ = 212.68, 180.50, 171.96, 171.30, 170.58, 170.12, 156.40, 123.32, 77.65, 76.30, 69.53, 69.15, 68.58, 61.12, 61.02, 47.07, 46.77, 43.73, 43.57, 43.45, 41.33, 40.06, 40.02, 38.42, 32.67, 32.06, 29.89, 28.81, 28.20, 27.99, 27.57, 26.11, 26.02, 23.58, 23.49, 20.63, 20.34, 20.11, 19.19, 18.88, 18.82, 18.72, 18.15, 17.90, 17.83, 17.13.

DEPT (176 MHz, DMSO-d$_6$): δ = 123.06, 77.39, 76.05, 69.27, 68.89, 68.32, 61.19, 60.86, 60.76, 46.87, 46.81, 46.52, 43.49, 43.31, 43.19, 41.07, 38.16, 32.42, 31.81, 29.64, 28.55, 27.95, 27.73, 27.31, 25.85, 25.76, 23.33, 23.23, 20.37, 20.09, 19.85, 18.62, 18.56, 17.89, 17.65, 17.57, 17.36, 16.87, 16.48.

HRMS (ESI) calculated for ([M+2H]$^{2+}$): m/z = 1181.1117; experimental = 1181.1126.

Compound **25**

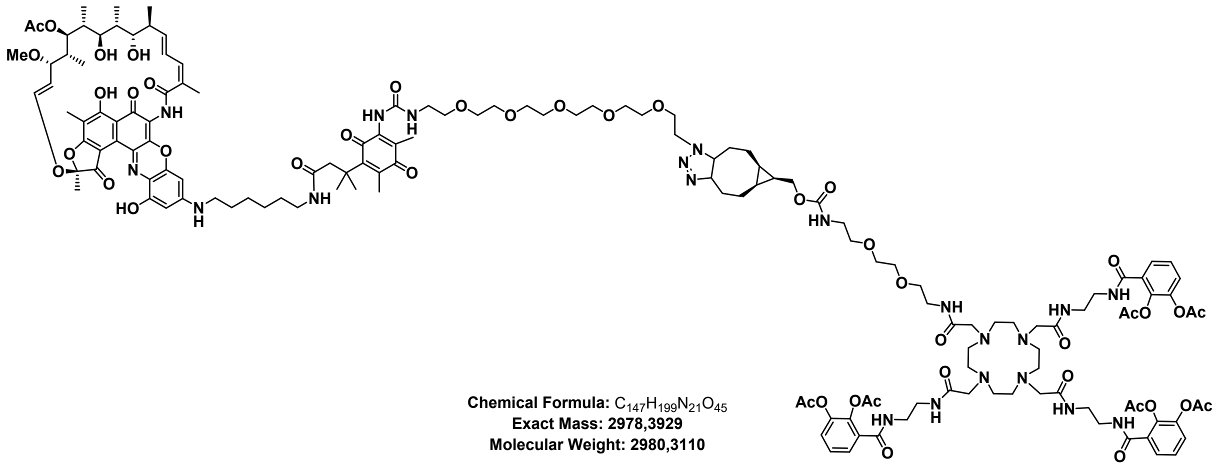

Alkyne **45** (2 mg, 0.001 mmol, 1.0 eq) and azide **64** (1.98 mg, 0.001 mmol, 1.0 eq) were weight in 1.5 mL tubes and then dissolved in degassed mixture of ACN:H_2O (1:1,500 µL each). The compounds were added together, AcOH (10 µL) was added, and the reaction continued stirring for 30 hours at 24 °C under argon atmosphere. The blue solution was filtered and purified by RP-HPLC (15-98-100% ACN/H_2O, 0.1% HCOOH, 220 nm, collect all.). Product containing fractions were lyophilized to yield **25** (2.995 mg, 0.001 mmol, 82%) as a beige solid. The compound eluted as a diastereomeric mixture in one peak from the HPLC, just one isomer is depicted here.

^{1}H-NMR (700 MHz, MeOH-d_4): δ = 8.83 (m, 3H), 8.68 (m, 6H), 8.23 (m, 1H), 7.73 (m, 2H), 7.63 (d, J = 12.94 Hz, 1H), 7.56 (m, 1H), 5.59 (t, J = 6.15 Hz, 2H), 5.46 (m, 2H), 5.06 (d, J = 9.97 Hz, 1H), 4.88 (m, 20H), 4.72 (m, 7H), 4.59 (m, 7H), 4.44 (m, 2H), 4.36 (d, J = 11.03 Hz, 4H), 4.26 (m, 9H), 4.15 (m, 1H), 4.07 (m, 2H), 3.63 (s, 9H), 3.60 (s, 9H), 3.52 (t, J = 7.43 Hz, 4H), 3.47 (bs, 3H), 3.43 (bs, 3H), 3.38 (d, J = 9.12 Hz, 1H), 3.34 (m, 2H), 3.20 (bs, 2H), 3.12 (m, 5H), 2.96 (m,9H), 2.79 (m, 10H), 2.64 (m, 30H), 2.36 (m, 2H), 2.30 (d, J = 7.43 Hz, 3H), 2.26 (m, 6H), 2.14 (m, 2H), 1.41 (d, J = 4.88 Hz, 3H), 1.03 (d, J = 6.79 Hz, 3H).

HRMS (ESI) calculated for ([M+2H]$^{2+}$): m/z =994.4443; experimental = 994.4454.

Compound **24SL**

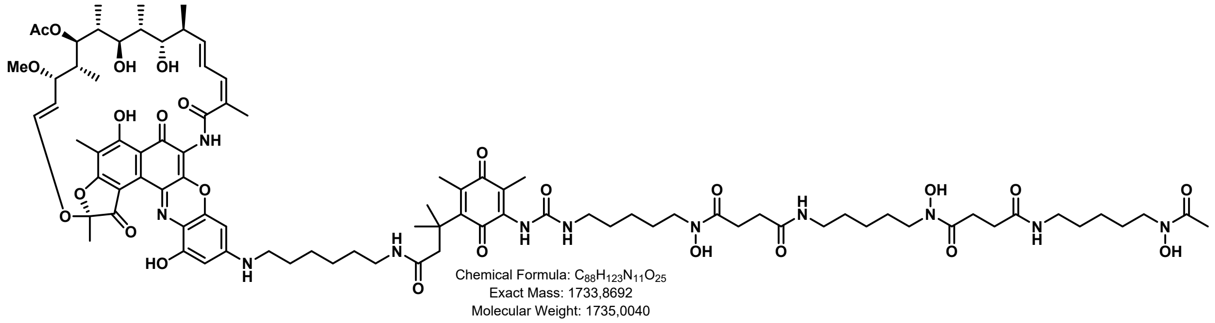

Acid **58a** (5.0 mg, 5 mmol, 1.0 eq) was dissolved in anhydrous DMF (1 mL) and HOBt (1.34 mg, 10.0 mmol, 2.0 eq), EDCI (1.43 mg, 10. mmol, 2 .0 eq) and DMAP (1.21 mg, 10.0 mmol, 2.0 eq) were added in one portion at 0 °C under argon atmosphere. The reaction stirred 45 minutes at 0 °C before **22** (6.81 mg, 7.0 mmol, 1.5 eq) was added, together with DIPEA (8.4 µL, 0.05 mmol, 10.0 eq) in anhydrous DMF (1 mL) at 0 °C and the ice bath was left to thaw overnight. The solvent was then blown off with a firm stream of nitrogen and the residue was purified by RP-HPLC (5-85-100% ACN/H_2O, 0.1% HCOOH, C18, 220 nm, collect all). The product containing fractions were identified by LCMS and lyophilized to yield **24SL** as a blue powder (6.35 mg, 4.0 mmol, 74%).

^{1}H-NMR (500 MHz, MeOH-d_4): δ = 8.03 (s, 1H), 7.76 (dd, J = 7.31, 26.98 Hz, 1H)), 7.34 (dt, J = 5.80, 1H), 6.84 (q, J = 10.09, 15.98 Hz, 1H), 6.39 (d, J = 10.09 Hz, 1H), 6.25 (m, 4H), 5.00 (m, 2H), 4.64 (s, 1H), 3.68 (d, J = 10.34 Hz, 1H), 3.59 (m, 5H), 3.35 (s, 2H), 3.16 (m, 6H), 3.09 (m, 2H), 2.96 (s, 3H), 2.76 (m, 4H), 2.45 (m, 3H), 2.35 (m, 1H), 2.27 (s, 3H), 2.21 (m, 1H), 2.14 (s, 2H), 2.12 (bs, 3H), 2.09 (s, 3H), 1.97 (bs, 3H), 1.85 (bs, 3H), 1.79 (bs, 3H), 1.63 (m, 6H), 1.51 (m, 6H), 1.46 (s, 6H), 1.33 (m, 10H), 0.95 (d, J = 6.70 Hz, 3H), 0.90 (m, 4H), 0.77 (m, 2H), 0.67 (m, 1H), 0.02 (d, J = 6.61 Hz, 3H), -0.34 (d, J = 6.33 Hz, 3 H).

DEPT (176 MHz, MeOH-d_4): δ = 163.46, 144.97, 140.37, 133.72, 133.67, 129.59, 127.92, 127.23, 121.76, 120.39, 119.99, 107.92, 77.68, 77.56, 75.46, 74.51, 56.53, 49.57, 44.24, 44.18, 41.49, 40.72, 40.08, 40.01, 39.67, 39.11, 38.55, 38.26, 33.89, 31.19, 30.28, 30.06, 29.70, 29.53, 29.44, 29.32, 28.63, 27.30, 27.19, 27.06, 24.61, 24.51, 22.51, 22.28, 20.45, 20.08, 20.04, 19.96, 14.32, 12.14, 10.59, 10.55, 9.57, 8.38, 7.10.

HRMS (ESI) calculated for ($[M+2H]^{2+}$): m/z = 867.9418; experimental = 867.9423.

Compound **26**

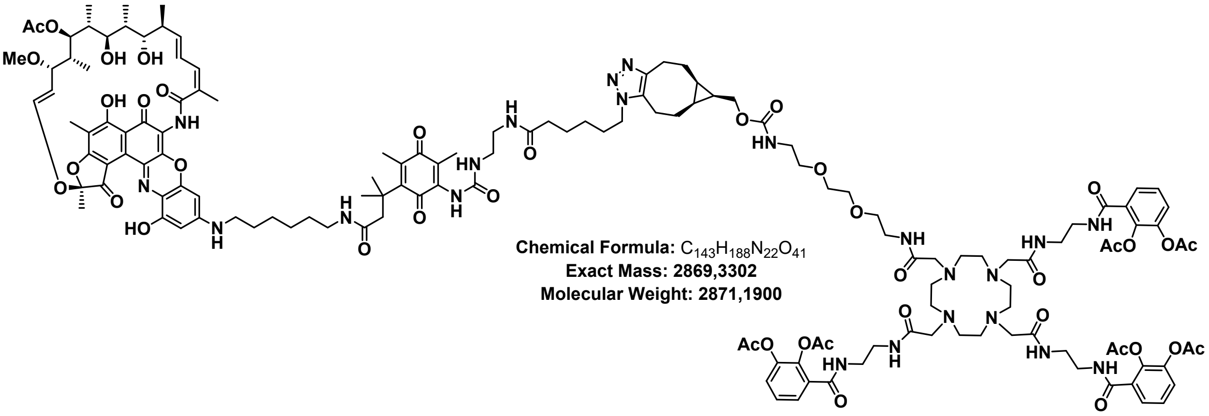

Alkyne **45** (5 mg, 0.003 mmol, 1.0 eq) and azide **63** (4.59 mg, 0.003 mmol, 1.0 eq) were weight in 1.5 mL tubes and then dissolved in degassed mixture of ACN:H_2O (1:1,500 µL each). The compounds were added together, AcOH (10 µL) was added, and the reaction continued stirring for 30 hours at 24 °C under argon atmosphere. The blue solution was filtered and purified by RP-HPLC (15-98-100% ACN/H_2O, 0.1% HCOOH, 220 nm, collect all.). Product containing fractions were lyophilized to yield **26** (7.49 mg, 0.003 mmol, 78%) as a beige solid. The compound eluted as a diastereomeric mixture in one peak from the HPLC, just one isomer is depicted here.

^{1}H-NMR (700 MHz, MeOH-d_4): δ = 7.48 (m, 3H), 7.34 (m, 6H), 6.88 (m, 1H), 6.38 (m, 1H), 6.28 (m, 1H), 6.21 (m, 1H), 4.26 (m, 1H), 4.11 (m, 1H), 3.71 (d, J = 10.40 Hz, 1H), 3.54 (m, 27H), 3.39 (m, 14H), 3.15 (m, 2H), 2.94 (m, 13H), 2.79 (m, 1H), 2.29 (s, 9H), 2.26 (s, 9H), 2.15 (t, J = 7.24 Hz, 3H), 2.09 (bs, 3H), 1.97 (m, 16H), 1.79 (m, 4H), 1.67 (m, 2H), 1.59 (m, 6H), 1.49 (m, 2H), 1.43 (m, 3H), 1.36 (m, 3H), 1.29 (bs, 10H), 1.01 (m, 2H), 0.95 (m, 3H), 0.91 (m, 5H), 0.05 (d, J = 5.20 Hz, 3H), -0.31 (d, J = 4.52 Hz, 3H).

HRMS (ESI) calculated for ($[M+2H]^{2+}$): m/z = 1435.6724; experimental = 1435.6733, calculated for ($[M+3H]^{3+}$): m/z =957.4501; experimental = 957.4501.

Compound **29**

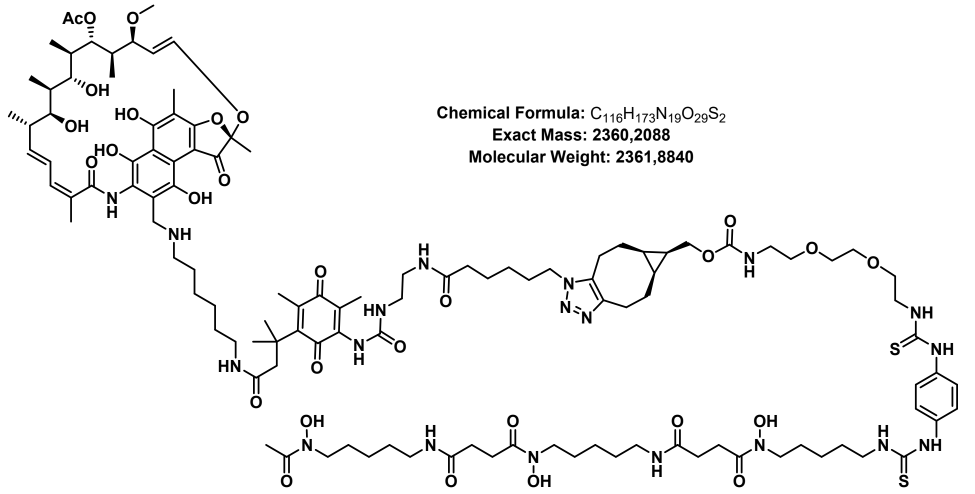

Azide **65** (12.3, 0.0096 mmol, 1.0 eq) and strained alkyne **47** (16.5 mg, 0.0153 mmol, 1.6 eq) were weight in 1.5 mL tubes and then dissolved in degassed mixture of ACN:H_2O (1:1, 300 µL each). The compounds were added together under argon atmosphere and continued stirring for 30 hours at 24 °C. The orange solution was filtered and purified by RP-HPLC (15-98-100% ACN/H_2O, 0.1% HCOOH, 220 nm, collect all.). Product containing fractions were lyophilized to yield **29** (19.85 mg, 0.008 mmol, 88%) as a beige solid. The compound eluted as a diastereomeric mixture in one peak from the HPLC, just one isomer is depicted here.

^{1}H-NMR (600 MHz, DMSO-d_6): δ = 9.64 (m, 5H), 9.44 (bs, 1H), 8.85 (d, J = 11.28 Hz, 1H), 8.30 (s, 1H), 7.78 (m, 4H), 7.68 (m, 4H), 7.35 (s, 4H), 7.20 (d, J = 5.77 Hz, 2H), 7.12 (m, 2H), 6.32 (d, J = 10.47 Hz, 1H), 6.25 (d, J = 12.75 Hz, 1H), 6.07 (dd, J = 8.19, 15.57 Hz, 1H), 5.07 (m, 2H), 4.90 (q, J = 7.79, 12.48 Hz, 1H), 4.19 (t, J = 6.58 Hz, 2H), 4.04 (m, 5H), 3.69 (m, 2H), 3.64 (m, 7H), 3.55 (m, 15H), 3.46 (m, 13H), 3.41 (m, 7H), 3.12 (q, J = 4.97, 10.87 Hz, 4H), 3.01 (m, 10H), 2.89 (s, 3H), 2.58 (m, 11H), 2.28 (m, 10H), 1.99 (s, 3H), 1.97 (s, 9H), 1.91 (s, 3H), 1.69 (m, 4H), 1.64 (s, 3H), 1.54 (m, 10H), 1.50 (m, 9H), 1.38 (m, 10H), 1.24 (m, 20H), 1.15 (m, 4H), 0.91 (m, 6H), 0.86 (m, 4H), 0.52 (d, J = 6.58 Hz, 3H), -0.33 (d, J = 6.31 Hz, 3H).

^{13}C-NMR (151 MHz, DMSO-d_6): δ = 180.98, 180.76, 172.43, 172.23, 172.11, 171.77, 170.60, 169.84, 156.93, 149.46, 143.81, 143.54, 133.17, 126.93, 123.82, 109.19, 99.02, 76.82, 76.27, 73.67, 70.25, 70.00, 69.63, 69.06, 61.92, 56.07, 47.60, 47.55, 47.48, 47.25, 44.22, 44.04, 40.74, 35.66, 33.11, 30.37, 29.79, 29.48, 29.29, 29.01, 28.68, 28.04, 27.36, 26.59, 26.50,

26.28, 26.09, 25.84, 25.24, 24.07, 23.97, 22.60, 22.36, 21.72, 21.12, 20.82, 20.44, 20.26, 20.01, 19.63, 19.35, 19.05, 18.58, 17.77, 14.31, 14.22, 13.11, 12.81, 11.65, 9.44, 9.29, 7.84, 6.36, -12.68.

DEPT (151 MHz, DMSO-d_6): δ = 76.82, 73.67, 70.01, 69.63, 69.06, 61.92, 56.07, 47.60, 47.55, 47.48, 47.25, 44.21, 44.04, 38.90, 38.88, 38.73, 38.52, 38.29, 35.65, 30.37, 29.79, 29.43, 29.29, 28.68, 28.04, 27.36, 26.50, 26.09, 25.84, 25.24, 25.13, 24.06, 23.96, 22.60, 22.35, 21.71, 21.12, 20.86, 20.82, 20.26, 19.62, 19.05, 17.77, 14.31, 11.64, 9.44, 9.29, 7.83.

HRMS (ESI) calculated for ($[M+2H]^{2+}$): m/z = 1181.1117; experimental = 1181.1126.

Compound **30**

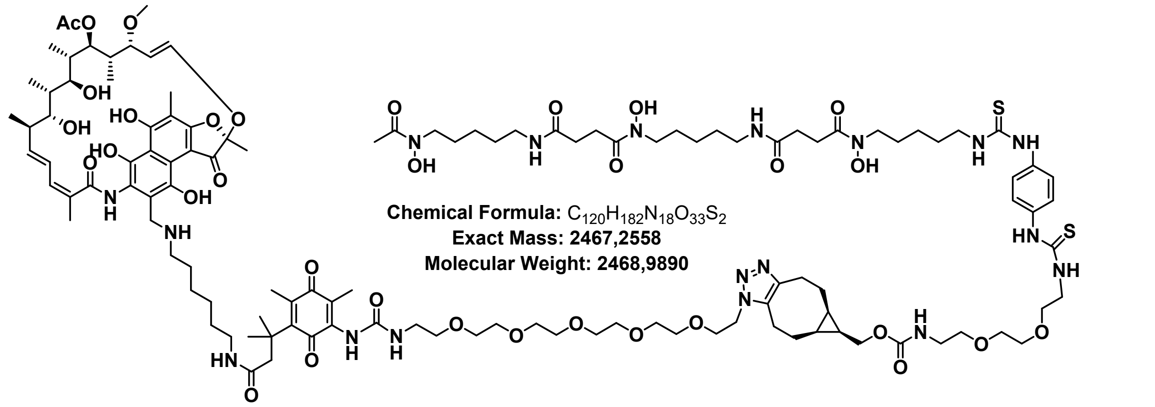

Azide **64** (12.3, 0.0096 mmol, 1.0 eq) and strained alkyne **47** (16.5 mg, 0.0153 mmol, 1.6 eq) were weight in 1.5 mL tubes and then dissolved in degassed mixture of ACN:H2O (1:1, 300 µL each). The compounds were added together under argon atmosphere and continued stirring for 30 hours at 24 °C. The orange solution was filtered and purified by RP-HPLC (15-98-100% ACN/H_2O, 0.1% HCOOH, 220 nm, collect all.). Product containing fractions were lyophilized to yield **30** (19.85 mg, 0.008 mmol, 88%) as a beige solid. The compound eluted as a diastereomeric mixture in one peak from the HPLC, just one isomer is depicted here.

^{1}H NMR (600 MHz, DMSO-d_6): δ = 9.62 (m, 4H), 7.78 (m, 2H), 7.66 (m, 1H), 7.35 (q, J = 9.65, 12.06 Hz, 4H), 7.11 (m, 1H), 4.39 (t, J = 5.13 Hz, 1H), 4.05 (m, 2H), 3.73 (t, J = 6.03 Hz, 1H), 3.64 (m, 2H), 3.49 (m, 30H), 3.30 (s, 4H), 3.16 (m,. 4H), 3.00 (q, J = 6.94, 13.87 Hz, 5H), 2.93 (m, 2H), 2.73 (m, 2H), 2.59 (m, 4H), 2.28 (m, 4H), 2.07 (n, 2H), 1.97 (m, 4H), 1.53 (m, 11H), 1.38 (m, 7H), 1.24 (m, 10H), 1.11 (m, 2H), 0.89 (m, 5H).

^{13}**C-NMR** (151 MHz, DMSO-d$_6$): δ = 187.71, 180.98, 172.44, 171.77, 170.60, 156.92, 143.64, 134.25, 125.76, 123.84, 70.27, 70.23, 70.14, 70.08, 70.01, 69.83, 69.63, 69.06, 61.93, 47.64, 47.25, 44.24, 44.04, 42.06, 40.53, 40.41, 40.28, 40.14, 40.00, 39.86, 39.72, 39.58, 38.90, 38.88, 38.41, 30.37, 29.48, 29.29, 28.82, 28.69, 28.04, 26.60, 26.50, 25.83, 24.07, 23.97, 22.70, 22.56, 22.44, 21.76, 20.82, 19.66, 19.09, 17.78, 14.75, 14.47, 14.37, 13.48, 13.18, 1.62.

DEPT (151 MHz, DMSO-d$_6$): δ = 123.84, 70.27, 70.23, 70.14, 70.08, 70.01, 69.83, 69.63, 69.06, 61.93, 47.64, 47.55, 47.25, 45.63, 44.23, 44.04, 42.05, 38.90, 30.36, 29.28, 28.81, 28.68, 28.03, 26.59, 26.50, 25.83, 24.07, 23.96, 22.70, 22.44, 21.76, 20.82, 19.66, 19.09, 17.77, 14.47, 13.48, 13.18, 1.62.

HRMS (ESI) calculated for ([M+2H]$^{2+}$): m/z = 1234.6352; experimental = 1234.6359.

Compound **30SL**

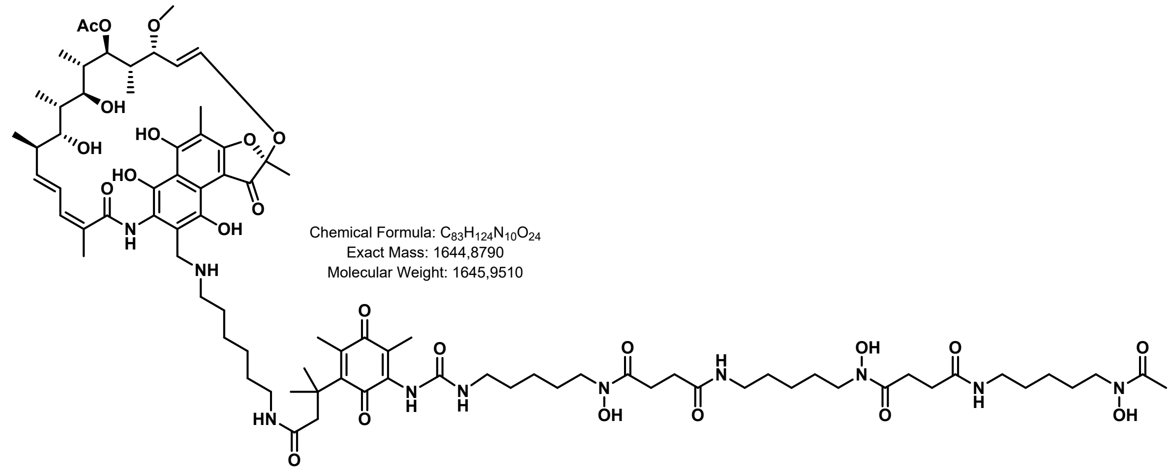

Acid **58a** (10.0 mg, 0.01 mmol, 1.0 eq) was dissolved in anhydrous DMF (1 mL) and HOBt (2.7 mg, 0.02 mmol, 2.0 eq), EDCI (3.8 mg, 0.015 mmol, 1.5 eq) and DMAP (2.42 mg, 0.02 mmol, 2.0 eq) were added in one portion at 0 °C under argon atmosphere. The reaction stirred 45 minutes at 0 °C before amine **28** (12.3 mg, 0.0154 mmol, 1.5 eq) was added, together with DIPEA (8.4 µL, 0.05 mmol, 10 eq) in anhydrous DMF (1 mL) at 0 °C and the ice bath was left to thaw overnight. The solvent was then blown off with a firm stream of nitrogen and the residue was purified by RP-HPLC (5-75-100% ACN/H_2O, 0.1% HCOOH, C18, 220 nm, collect all). The product containing fractions were identified by LCMS and lyophilized to yield **30SL** as a blue powder (7.6 mg, 0.005 mmol, 47%).

^{1}H-NMR (700 MHz, MeOH-d_4): δ = 6.18 (m, 2H), 5.15 (m, 1H), 5.07 (m, 1H), 4.58 (s, 1H), 3.80 (m, 1H), 3.59 (t, J = 7.01 Hz, 6H), 3.16 (m, 6H)), 3.06 (m, 3H), 3.01 (s, 2H), 2.76 (m, 5H), 2.45 (m, 4H), 2.15 (m, 3H), 2.09 (s, 3H), 2.03 (m, 6H), 1.88 (m, 3H), 1.71 (m, 4H), 1.63 (m, 6H), 1.52 (m, 7H), 1.45 (m, 6H), 1.32 (d, J = 6.61 Hz, 1.01 (d, J = 8.42 Hz, 3H), 0.92 (m, 3H), 0.67 (m, 2H), -0.22 (d, J = 6.81 Hz, 1H).

^{13}C-NMR (176 MHz, MeOH-d_4): δ = 144.74, 143.65, 141.28, 133.37, 127.29, 119.70, 117.99, 78.67, 78.17, 78.10, 76.09, 75.48, 75.36, 57.46, 56.90, 53.73, 45.13, 41.93, 41.15, 40.44, 40.13, 39.56, 39.29, 38.92, 38.84, 34.63, 31.61, 30.71, 30.25, 30.13, 29.92, 29.87, 29.06, 28.72, 27.58, 27.49, 27.15, 25.06, 25.04, 24.94, 22.44, 21.89, 20.95, 20.47, 20.39, 18.75, 14.74, 12.48, 11.28, 9.90, 9.78, 9.59, 7.53, 7.38.

Compound **33**

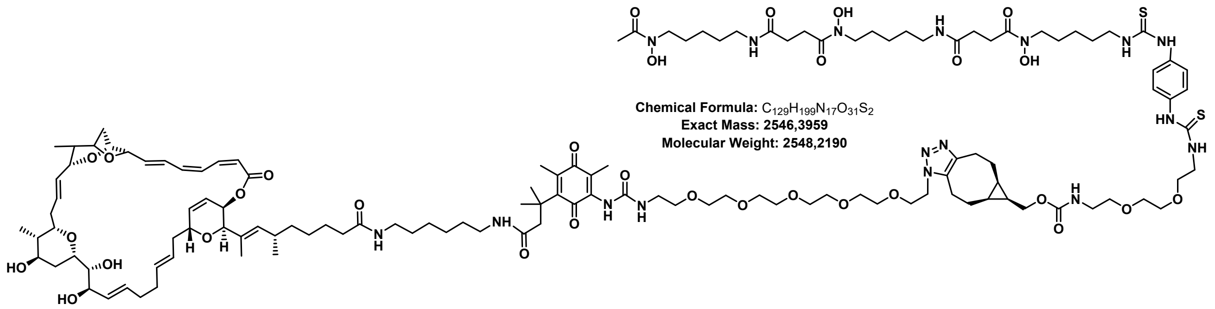

Azide **67** (18.4 mg, 0.008 mmol, 1.0 eq) and strained alkyne **47** (26.7 mg, 0.011 mmol, 2.0 eq) were weighed in 1.5 mL tubes and then dissolved in degassed mixture of ACN/H_2O (1:1, 300 µL each). The compounds were added together under argon atmosphere and continued stirring for 30 hours at 24 °C. The orange solution was filtered and purified by RP-HPLC (15-98-100% ACN/H_2O, 0.1% HCOOH, 220 nm, collect all.). Product containing fractions were lyophilized to yield **33** (21.58 mg, 0.009 mmol, 71%) as a yellow solid. The compound eluted as a diastereomeric mixture in one peak from the HPLC, just one isomer is depicted here.

^{1}H-NMR (600 MHz, DMSO-d_6): δ = 9.60 (m, 3H), 9.39 (bs, 1H), 8.11 (s, 1H), 7.85 (t, J = 4.51 Hz, 1H), 7.78 (t, J = 4.51 Hz, 1H), 7.65 (t, J = 5.29 Hz, 2H), 7.35 (d, J = 4.51 Hz, 4H), 7.11 (t, J = 10.70 Hz, 2H), 7.04 (t, J = 11.99 Hz, 1H), 6.92 (m, 1H), 6.81 (t, J = 5.93 Hz, 1H), 6.47 (10.83 Hz, 1H), 6.22 (dd, J = 4.51, 15.34 Hz, 1H), 6.14 (dd, J = 2.97, 10.44 Hz, 1H), 5.96 (m, 1H), 5.64 (d, J = 11.86 Hz, 1H), 5.51 (d, J = 6.32 Hz, 1H), 5.45 (m, 1H), 5.30 (m, 1H), 5.23 (m, 2H), 4.56 (d, J = 2.71, 1H), 4.50 (m, 1H), 4.43 (t, J = 5.54 Hz, 1H), 4.39 (t, J = 5.29 Hz, 2H), 4.18 (m, 2H), 4.05 (m, 2H), 3.95 (m, 1H), 3.73 (t, J = 5.29 Hz, 2H), 3.64 (m, 2H), 3.53 (m, 17H), 3.46 (m, 15H), 3.42 (q, J = 5.54, 10.44 Hz, 5H), 3.19 (q, J = 5.93, 11.22), 3.12 (q, J = 5.29,

11.99 Hz, 2H), 2.98 (m, 11H), 2.73 (m, 2H), 2.64 (m, 2H), 2.59 (q, J = 6.70, 12.77 Hz, 2H), 2.27 (m, 6H), 2.10 (m, 7H), 2.05 (s, 4H), 1.99 (m, 9H), 1.80 (d, J = 11.60 Hz, 1H), 1.77 (s, 3H), 1.54 (m, 12H), 1.38 (m, 5H), 1.35 (m, 12H), 1.22 (m, 14H), 1.12 (m, 5H), 0.92 (m, 2H), 0.78 (d, J = 7.09 Hz, 3H), 0.76 (d, J = 7.09 Hz, 3H), 0.71 (d, J = 6.70 Hz, 3H).

^{13}C-NMR (151 MHz, DMSO-d$_6$): δ = 187.49, 186.60, 180.98, 180.77, 172.52, 172.44, 171.77, 171.21, 170.59, 165.66, 156.92, 154.20, 151.82, 143.64, 141.28, 138.09, 138.05, 137.28, 136.31, 135.45, 134.24, 132.70, 132.59, 132.34, 131.01, 130.80, 130.68, 130.01, 127.13, 125.85, 125.72, 125.05, 123.81, 122.84, 118.85, 80.48, 79.32, 78.91, 76.76, 75.60, 73.44, 73.18, 72.92, 72.63, 72.19, 70.27, 70.23, 70.15, 70.08, 70.01, 69.83, 69.64, 69.06, 68.91, 65.22, 61.93, 48.05, 47.64, 47.55, 47.25, 44.24, 44.04, 40.61, 38.99, 38.90, 38.88, 38.79, 38.74, 37.28, 37.23, 36.15, 36.04, 34.18, 33.04, 32.59, 31.56, 30.36, 30.19, 29.60, 29.49, 29.29, 29.05, 28.69, 28.04, 27.15, 27.03, 26.59, 26.50, 25.84, 24.07, 23.97, 22.71, 22.44, 21.76, 21.59, 21.24, 20.82, 19.66, 19.09, 17.78, 15.40, 15.23, 14.37, 14.26, 13.93, 12.78, 10.74, 10.68, 6.36.

DEPT (151 MHz, DMSO-d$_6$): δ = 138.09, 137.28, 136.31, 135.45, 132.70, 132.59, 132.34, 131.01, 130.80, 130.68, 127.13, 125.85, 125.05, 123.79, 122.84, 118.85, 80.48, 79.32, 78.91, 76.76, 75.60, 73.43, 73.18, 72.92, 72.63, 72.19, 70.27, 70.23, 70.14, 70.08, 70.01, 69.83, 69.63, 69.06, 68.91, 65.22, 61.93, 48.04, 47.64, 47.55, 47.25, 44.23, 44.04, 38.99, 38.90, 38.88, 38.79, 38.73, 37.28, 37.22, 36.15, 36.04, 34.18, 33.04, 32.59, 31.56, 30.36, 30.19, 29.60, 29.49, 29.29, 29.05, 28.68, 28.03, 27.03, 26.59, 26.50, 25.83, 24.07, 23.96, 22.71, 22.44, 21.76, 21.24, 20.82, 19.66, 19.09, 17.77, 15.23, 14.37, 13.93, 12.78, 10.73.

HRMS (ESI) calculated for ($[M+3H]^{3+}$): m/z = 849.8059; experimental = 849.8066.

Compound **34**

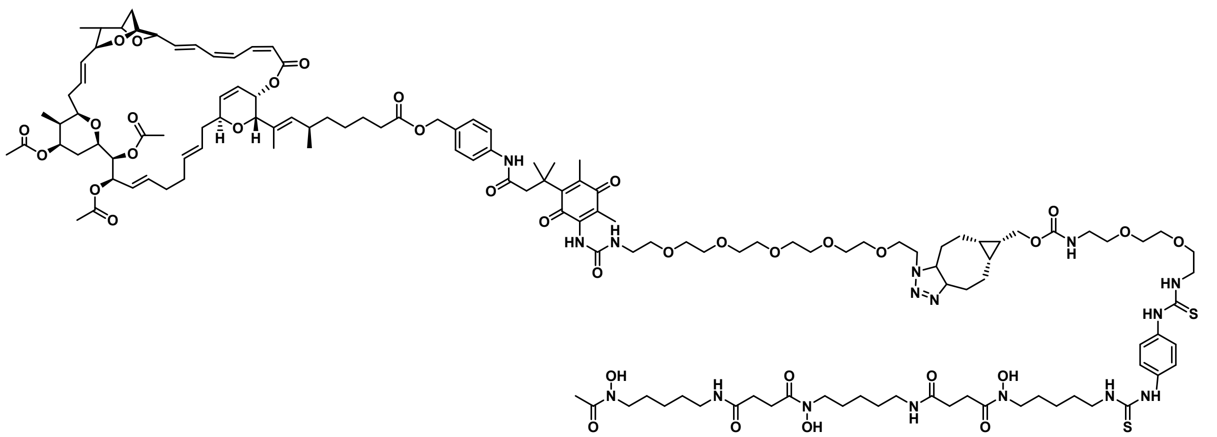

Chemical Formula: $C_{136}H_{200}N_{16}O_{35}S_2$
Exact Mass: 2681,3803
Molecular Weight: 2683,2930

Azide **69** (14.3 mg, 0.005 mmol, 1.0 eq) and strained alkyne **47** (12.58 mg, 0.010 mmol, 2.0 eq) were weight in 1.5 mL tubes and then dissolved in degassed mixture of ACN:H_2O (1:1, 300 µL each). The compounds were added together under argon atmosphere and continued stirring for 30 hours at 24 °C. The orange solution was filtered and purified by RP-HPLC (15-98-100% ACN/H_2O, 0.1% HCOOH, 220 nm, collect all.). Product containing fractions were lyophilized to yield **34** (12.69 mg, 0.005 mmol, 95%) as a beige solid. The compound eluted as a diastereomeric mixture in one peak from the HPLC, just one isomer is depicted here.

^{1}H-NMR (600 MHz, DMSO-d_6): δ = 7.12 (t, J = 11.06, 1H), 7.03 (m, 1H), 6.92 (m, 1H), 6.48 (t, J = 11.06 Hz, 1H), 6.22 (m, 1H), 6.14 (dd, J = 3.43, 9.34 Hz, 1H), 5.97 (m, 1H), 5.63 (m, 2H), 5.52 (m, 1H), 5.44 (m, 3H), 5.33 (m, 2H), 5.25 (m, 3H), 5.20 (d, J = 9.92 Hz, 1H), 4.50 (m, 1H), 4.35 (m, 3H), 4.19 (m, 3H), 3.67 (m, 9H), 3.17 (s, 3H), 2.55 (s, 3H), 2.30 (m, 4H), 2.13 (m, 15H), 1.96 (10H), 1.80 (m, 2H), 1.55 (m, 8H), 1.41 (m, 10H), 1.27 (m, 4H), 1.17 (m, 5H), 1.07 (m, 2H), 0.78 (d, J = 6.67 Hz, 3H), 0.75 (d, J = 7.06 Hz, 3H), 0.71 (d, J = 7.25 Hz, 3H).

^{13}C-NMR (151 MHz, DMSO-d_6): δ = 175.06, 175.00, 170.25, 169.79, 165.69, 165.64, 138.17, 137.23, 136.33, 135.59, 135.44, 135.25, 132.58, 132.45, 132.41, 132.36, 130.21, 130.07, 129.50, 127.47, 127.33, 125.99, 125.16, 125.05, 124.84, 122.94, 122.79, 118.94, 118.82, 80.46, 80.39, 79.41, 79.33, 79.03, 78.91, 77.73, 75.83, 75.76, 75.58, 73.87, 73.37, 73.27, 73.00, 72.60, 72.49, 71.41, 70.53, 68.69, 68.41, 66.82, 65.28, 65.18, 49.06, 40.91, 38.92, 37.36, 37.29, 37.03, 36.82, 35.97, 34.29, 34.04, 33.11, 32.88, 32.45, 31.64, 31.46, 30.98, 27.00, 26.91, 25.07, 24.96, 21.65, 21.42, 21.28, 21.26, 15.23, 15.20, 14.43, 14.02, 13.89, 10.69, 10.62.

DEPT (151 MHz, DMSO-d_6): δ = 137.43, 136.56, 136.50, 135.60, 134.85, 134.71, 134.52, 131.81, 131.71, 131.68, 131.63, 129.59, 129.47, 128.76, 126.73, 126.59, 125.25, 124.99, 124.43, 124.31, 124.10, 122.20, 122.05, 118.23, 118.20, 118.08, 79.74, 79.71, 78.69, 78.60, 78.30, 77.01, 75.10, 75.04, 73.14, 72.65, 72.54, 72.36, 72.30, 72.27, 71.88, 71.77, 71.74, 70.68, 69.80, 67.97, 67.68, 64.55, 64.46, 48.33, 40.17, 38.19, 36.63, 36.55, 36.29, 36.08, 35.23, 33.56, 33.37, 33.31, 32.38, 32.15, 31.71, 30.91, 30.72, 30.25, 28.99, 26.27, 26.18, 24.33, 24.23, 20.92, 20.69, 20.54, 20.52, 14.49, 14.46, 13.29, 13.16, 9.96, 9.88.

HRMS (ESI) calculated for ($[M+3H]^{3+}$): m/z = 894.8007; experimental = 894.8029.

Compound **36**

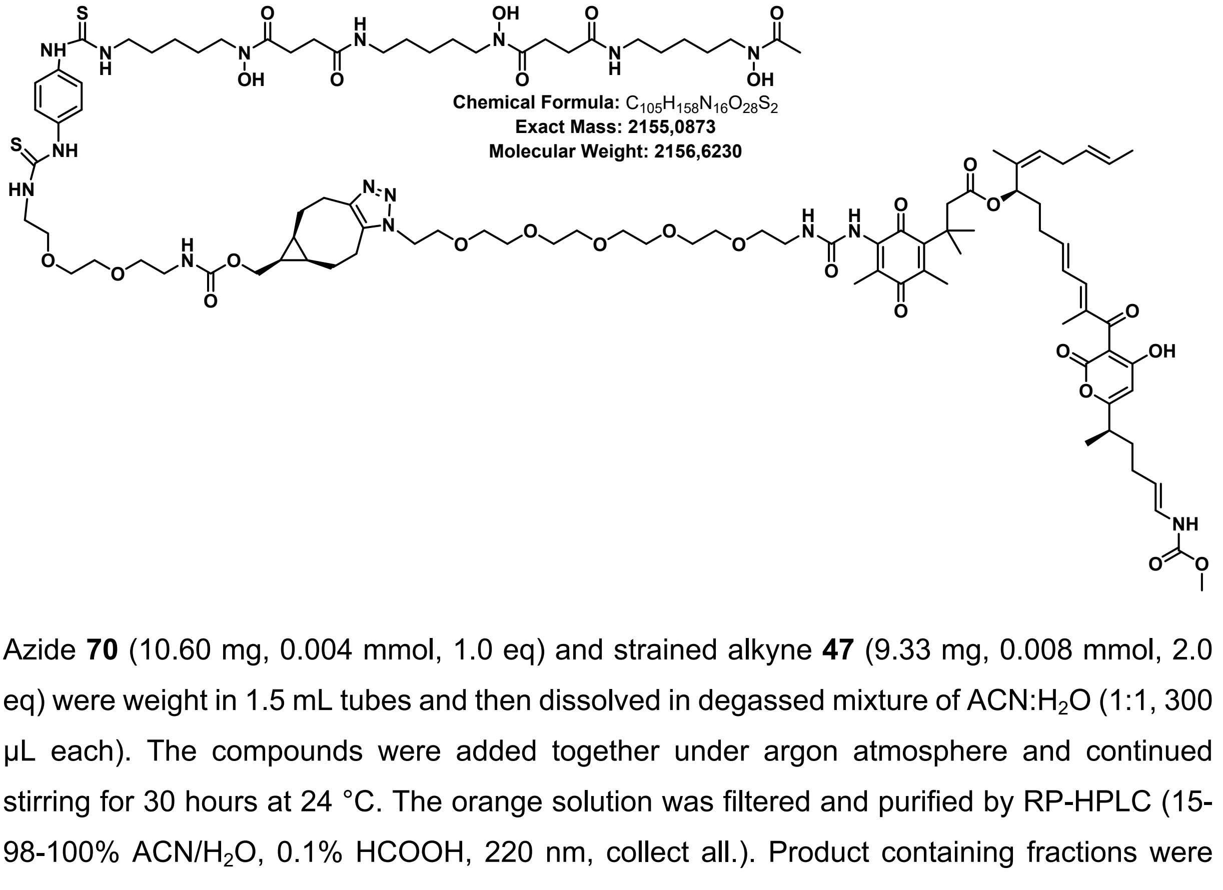

Azide **70** (10.60 mg, 0.004 mmol, 1.0 eq) and strained alkyne **47** (9.33 mg, 0.008 mmol, 2.0 eq) were weight in 1.5 mL tubes and then dissolved in degassed mixture of ACN:H_2O (1:1, 300 µL each). The compounds were added together under argon atmosphere and continued stirring for 30 hours at 24 °C. The orange solution was filtered and purified by RP-HPLC (15-98-100% ACN/H_2O, 0.1% HCOOH, 220 nm, collect all.). Product containing fractions were lyophilized to yield **36** (6.54 mg, 0.003 mmol, 79%) as a beige solid. The compound eluted as a diastereomeric mixture in one peak from the HPLC, just one isomer is depicted here. Isomerization at C19/C20 reported and observed (e.g. by NMR), intermediate eluted as isomeric mixture as reported for free CorA **35** in Figure S18-S21.

^{1}H-NMR (600 MHz, DMSO-d_6): δ = 9.62 (m, 5H), 9.39 (m, 1H), 9.21 (d, J = 10.47 Hz, 1H), 7.78 (m, 2H), 7.65 (m, 2H), 7.34 (d, J = 4.79 Hz, 4H), 7.11 (t, J = 5.08 Hz, 1H), 6.73 (m, 1H), 6.34 (m, 1H), 5.35 (m, 2H), 5.12 (t, J = 7.48 Hz, 1H), 5.02 (m, 1H), 4.39 (t, J = 5.08 Hz, 2H), 4.04 (m, 3H), 3.73 (t, J = 5.68 Hz, 2H), 3.64 (m, 3H), 3.56 (m, 21H), 3.46 (m, 25H), 3.22 (m, 3H), 3.13 (q, J = 6.13, 11.22 Hz, 3H), 3.01 (q, J = 6.58, 13.01 Hz, 6H), 2.71 (m, 5H), 2.59 (q, J = 7.33, 12.41 Hz, 4H), 2.27 (q, J = 6.43, 10.77 Hz, 4H), 2.05 (s, 3H), 1.97 (s, 3H), 1.76 (s, 3H), 1.60 (m, 10H), 1.53 (m, 20H), 1.38 (m, 9H), 1.23 (m, 8H), 1.11 (m, 5H), 0.92 (m, 2H).

^{13}C-NMR (151 MHz, DMSO-d_6): δ = 180.51, 180.28, 171.96, 171.29, 170.12, 168.70, 156.44, 154.17, 143.16, 133.77, 129.94, 129.88, 129.78, 124.86, 124.65, 124.54, 124.31, 123.34, 108.89, 82.69, 75.71, 69.79, 69.75, 69.67, 69.60, 69.53, 69.36, 69.16, 68.58, 67.18, 61.45, 51.63, 47.17, 47.08, 46.77, 43.76, 43.56, 38.42, 38.40, 38.13, 36.80, 36.62, 36.45, 34.21, 30.02, 29.96, 29.89, 28.81, 28.64, 28.57, 28.21, 27.99, 27.56, 26.89, 26.72, 26.64, 26.12, 26.02, 25.35, 24.14, 23.60, 23.49, 23.11, 22.23, 21.97, 21.28, 20.34, 19.18, 18.78, 18.61, 17.90, 17.69, 17.64, 17.30, 13.75, 13.68, 12.28, 5.88.

DEPT (151 MHz, DMSO-d_6): δ = 130.42, 130.35, 130.25, 125.34, 125.21, 125.13, 125.02, 123.82, 109.20, 76.19, 70.27, 70.23, 70.14, 70.08, 70.01, 69.83, 69.63, 69.06, 67.65, 61.93, 52.11, 47.64, 47.55, 47.25, 44.24, 44.04, 40.53, 40.39, 40.25, 40.15, 40.11, 40.01, 39.97, 39.87, 39.84, 39.72, 39.70, 39.27, 38.90, 38.88, 38.60, 37.10, 34.68, 33.79, 30.36, 29.29, 29.12, 28.69, 28.03, 27.36, 27.12, 26.60, 26.50, 25.83, 24.61, 24.07, 23.96, 22.70, 22.44, 21.76, 20.82, 19.66, 19.09, 18.38, 18.17, 18.12, 17.77, 14.23, 14.15.

HRMS (ESI) calculated for ($[M+2H]^{2+}$): m/z = 1085.5587; experimental = 1085599.

Compound **37**

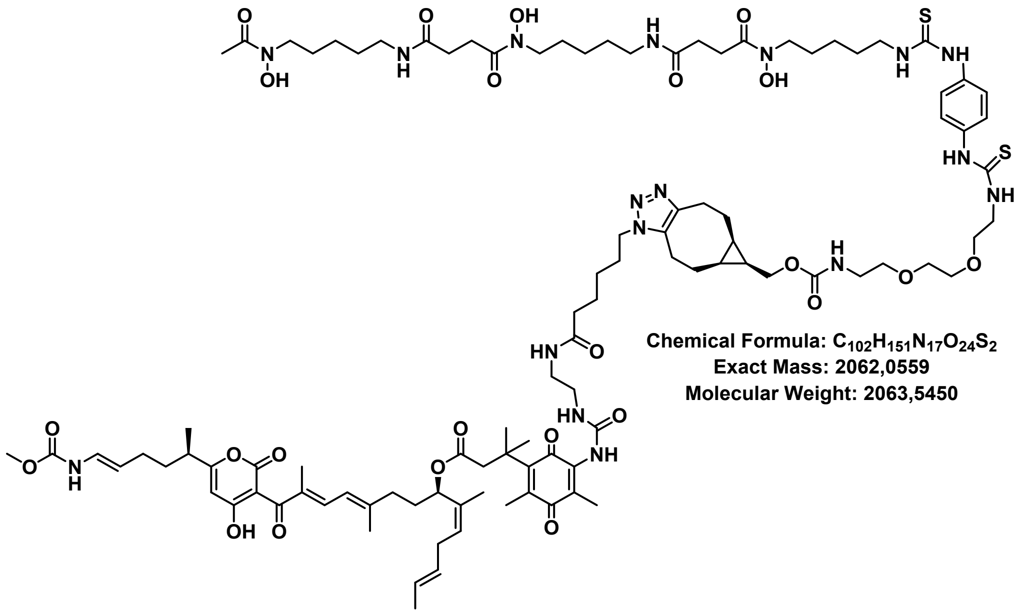

Strained alkyne **47** (10 mg, 9 µmol, 1.0 eq) and azide **71** (9 mg, 9 µmol, 1.0 eq) were added together and dissolved under Argon atmosphere in a 1:1 mixture of ACN and milliQ water (10 mL in total). The reaction was stirred overnight at 23 °C and the reaction progress was checked by LCMS. The solution obtained a less intense blue color upon full conversion, possibly due to quenching effects of the quinone moiety. The solvent was removed by rotary evaporation and the residue was dissolved in a mixture of ACN:MeOH:DMSO (5 mL) and purified by RP-HPLC (15-85% then 100%, 220 nm, collect all). The product containing fractions be found by LCMS and the product was lyophilized to dryness to yield **37** (5.45 mg, 0.005 mmol, 49%) as a slightly beige solid. The compound eluted as a diastereomeric mixture in one peak from the HPLC, just one isomer is depicted here. Isomerization at C19/C20 reported and observed (e.g. by NMR), intermediate eluted as isomeric mixture as reported for free CorA **35** in Figure S18-S21.

^{1}**H-NMR** (700 MHz, DMSO-d$_6$): δ = 9.59 (m, 4H), 9.35 (m, 1H), 7.84 (m, 1H), 7.77 (m, 2H), 7.67 (m, 2H), 7.34 (q, J = 7.94, 12.15 Hz, 4H), 7.11 (m, 1H), 4.18 (t, J = 6.78 Hz, 2H), 4.04 (m, 2H), 3.63 (m, 3H), 3.54 (m, 8H), 3.45-3.40 (m, 12H), 3.29 (s, 2H), 3.12 (q, J = 6.09, 11.45 Hz, 2H), 3.06 (bs, 2H), 2.99 (q, J = 6.07, 12.15 Hz, 6H), 2.93 (m, 5H), 2.88 (s, 1H), 2.74 (m, 1H), 2.67 (m, 1H), 2.58 (q, J = 8.18, 14.25 Hz, 4H), 2.27 (q, J = 7.24, 12.85 Hz, 4H), 2.01 (m, 7H), 1.96 (s, 4H), 1.76 (s, 1H), 1.68 (m, 3H), 1.64 (bs, 1H), 1.52 (m, 15H), 1.38 (m, 5H), 1.33 (m, 3H), 1.22 (m, 10H), 0.88 (m, 7H).

^{13}C-NMR (176 MHz, DMSO-d$_6$): δ = 180.49, 171.94, 171.28, 170.10, 156.43, 143.32, 132.67, 123.29, 69.51, 69.14, 68.57, 61.42, 55.60, 47.05, 46.97, 46.75, 43.55, 40.00, 38.40, 38.38, 38.24, 35.19, 29.87, 29.30, 28.79, 28.52, 28.19, 27.54, 26.10, 26.00, 25.60, 25.35, 24.74, 24.64, 23.58, 23.47, 22.11, 22.03, 21.85, 21.20, 20.63, 20.33, 19.13, 18.55, 18.17, 17.27, 13.89, 8.94, 8.75, 7.32.

Compound **37SLa**

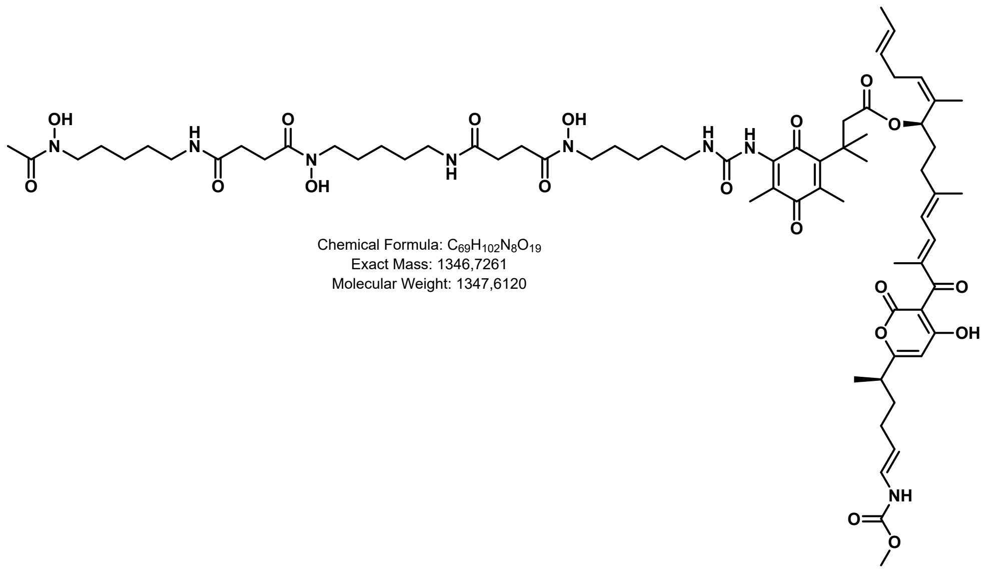

Acid **58a** (17.0 mg, 0.02 mmol, 1.0 eq) was dissolved in anhydrous DMF (1 mL) and HOBt (2.7 mg, 0.041 mg, 2.0 eq), EDCI (3.9 mg, 0.041 mmol, 2.0 eq) and DMAP (2.48 mg, 0.041 mmol, 2.0 eq) were added in one portion at 0 °C under argon atmosphere. The reaction stirred 45 minutes at 0 °C before CorA **35** (10.69 mg, 0.030 mmol, 1.5 eq) was added in anhydrous DMF (1 mL) at 0 °C and the ice bath was left to thaw overnight. The solvent was then blown off with a firm stream of nitrogen and the residue was purified by RP-HPLC (5-50-100% ACN/H_2O, no acid, C18, 220 nm, collect all). The product containing fractions were identified by LCMS and lyophilized to yield **37SLa** as a yellow powder (7.6 mg, 0.006 mmol, 28%). Isomerization at C19/C20 reported and observed (e.g. by NMR), intermediate eluted as isomeric mixture as reported for free CorA **35** in Figure S18-S21.

^{1}H-NMR (700 MHz, MeOH-d$_4$): δ = 6.13 (m, 2H), 5.42 (m, 2H), 5.18 (m, 1H), 4.50 (m, 2H), 3.86 (m, 2H), 3.60 (m, 9H), 3.16 (m, 5H), 2.92 (m, 4H), 2.84 (m, 1H), 2.76 (m, 2H), 2.45 (m, 2H), 2.18 (m, 4H), 2.09 (s, 3H), 1.98 (m, 5H), 1.87 (m, 3H), 1.77 (m, 3H), 1.71 (s, 3H), 1.64 (m, 5H), 1.53 (m, 9H), 1.33 (m, 7H), 1.24 (m, 2H), 1.15 (m, 3H), 0.90 (m, 3H).

^{13}C-NMR (176 MHz, MeOH-d$_4$): δ = 165.04, 152.34, 138.67, 131.11, 129.81, 126.67, 126.64, 126.42, 126.24, 125.46, 125.43, 125.39, 118.83, 118.81, 112.56, 112.54, 70.06, 69.61, 57.62, 57.17, 57.11, 56.96, 56.92, 56.89, 56.64, 54.42, 52.05, 51.83, 49.99, 49.67, 47.36, 46.85, 46.74, 44.23, 44.02, 43.58, 41.93, 41.78, 40.43, 40.28, 37.60, 37.44, 37.10, 31.80, 31.61, 30.89, 30.08, 29.06, 29.01, 28.96, 28.39, 27.97, 27.47, 27.41, 26.44, 25.01, 24.92, 20.37, 18.25, 18.21, 17.90, 17.87, 17.42, 17.28, 17.20, 15.83, 15.34, 10.12, 10.10.

Compound **37SLb**

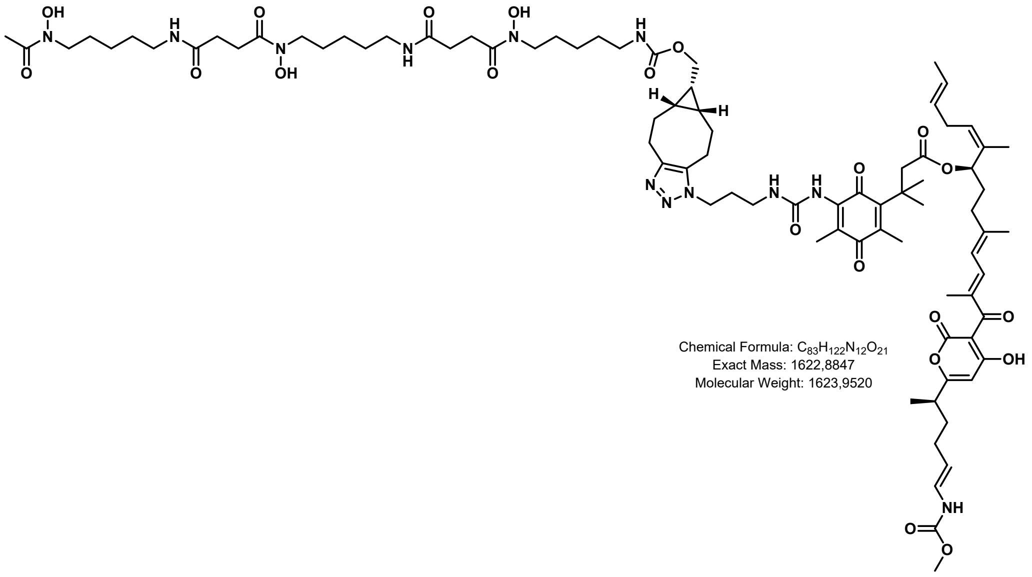

Strained alkyne **47** (10 mg, 9 µmol, 1.0 eq) and azide **74** (8.1 mg, 9 µmol, 1.0 eq) were added together and dissolved under Argon atmosphere in a 1:1 mixture of ACN and milliQ water (10 mL in total). The reaction was stirred overnight at 23 °C and the reaction progress was checked by LCMS. The solvent was removed by rotary evaporation and the residue was dissolved in a mixture of ACN:MeOH:DMSO (5 mL) and purified by RP-HPLC (20-80% then 100%, 220 nm, collect all). The product containing fractions were identified by LCMS and the product was lyophilized to dryness to yield **37SLb** (3.2 mg, 0.002 mmol, 20%) as a slightly yellow solid. The compound eluted as a diastereomeric mixture in one peak from the HPLC, just one isomer is depicted here. Isomerization at C19/C20 reported and observed (e.g. by NMR), intermediate eluted as isomeric mixture as reported for free CorA **35** in Figure S18-S21.

^{1}H-NMR (700 MHz, DMSO-d$_6$): δ = 9.61 (m, 2H), 9.21 (m, 1H), 7.77 (t, J = 5.81 Hz, 2H), 7.08 (t, J = 5.44 Hz, 1H), 6.32 (m, 1H), 5.34 (m, 2H), 5.11 (m, 1H), 5.00 (m, 1H), 4.26 (t, J = 6.00 Hz, 2H), 4.04 (m, 2H), 3.45 (s, 3H), 3.29 (m, 6H), 3.05 (s, 2H), 2.99 (m, 5H), 2.94 (t, J = 6.75 Hz, 3H), 2.57 (t, J = 7.69 Hz, 4H), 2.26 (m, 2H), 2.04 (m, 3H), 1.96 (s, 3H), 1.87 (m, 2H), 1.76

(s, 2H), 1.59 (bs, 6H), 1.49 (bs, 6H), 1.38 (bs, 12H), 1.23 (bs, 16H), 1.10 (m, 4H), 0.91 (m, 3H).

Biological methods

Biology figures and tables

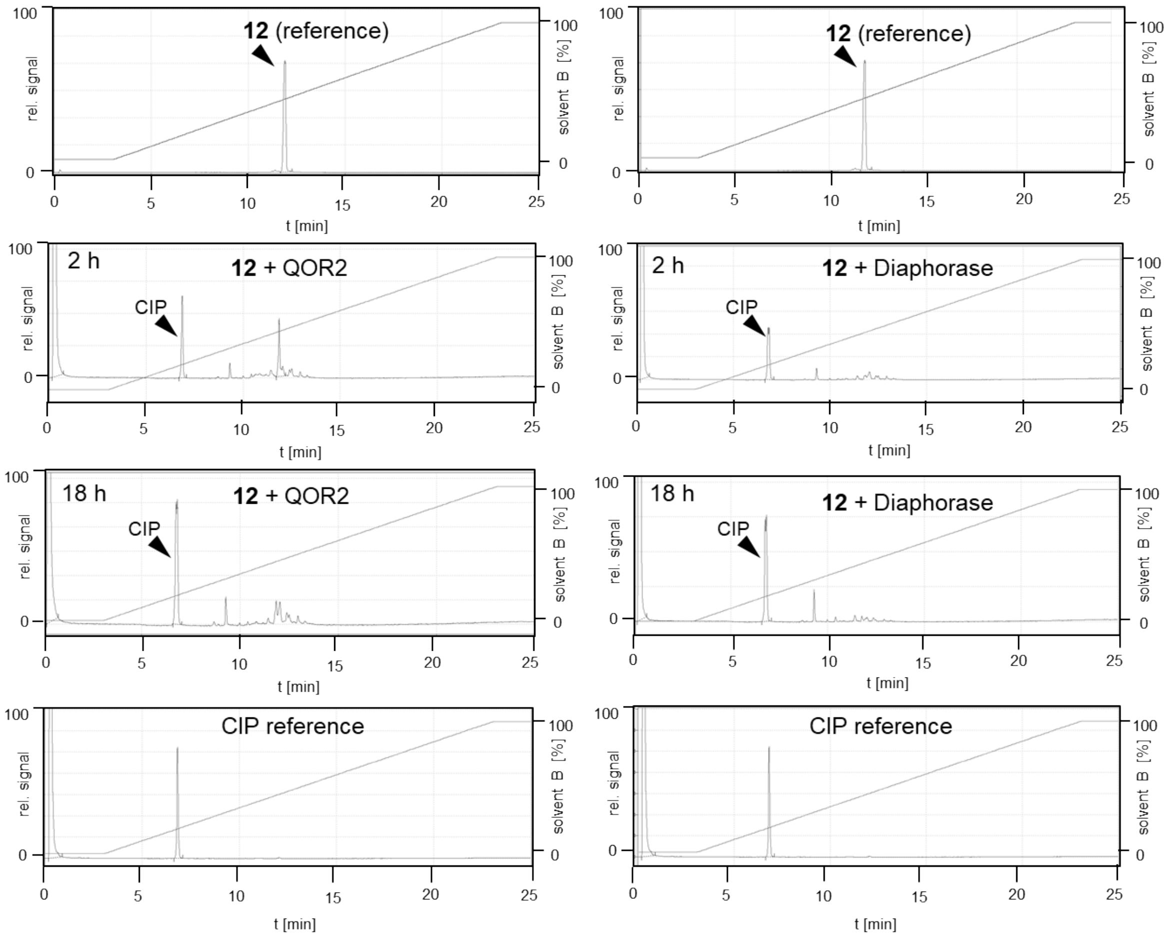

Figure S22. Qualitative information on the enzymatic cleavage of DFO TML ciprofloxacin conjugate **12** with QOR2 (left) and Diaphorase (right), relative UV (220 nm) signal intensity, normalized to the highest peak. Analytical HPLC runs from compound **12** at t = 0, 2 h and 18 h for QOR2 or diaphorase addition. Ciprofloxacin reference injection at the bottom, enzymatic reactions were quenched by 1:1 dilution of reaction with HPLC-grade MeOH.

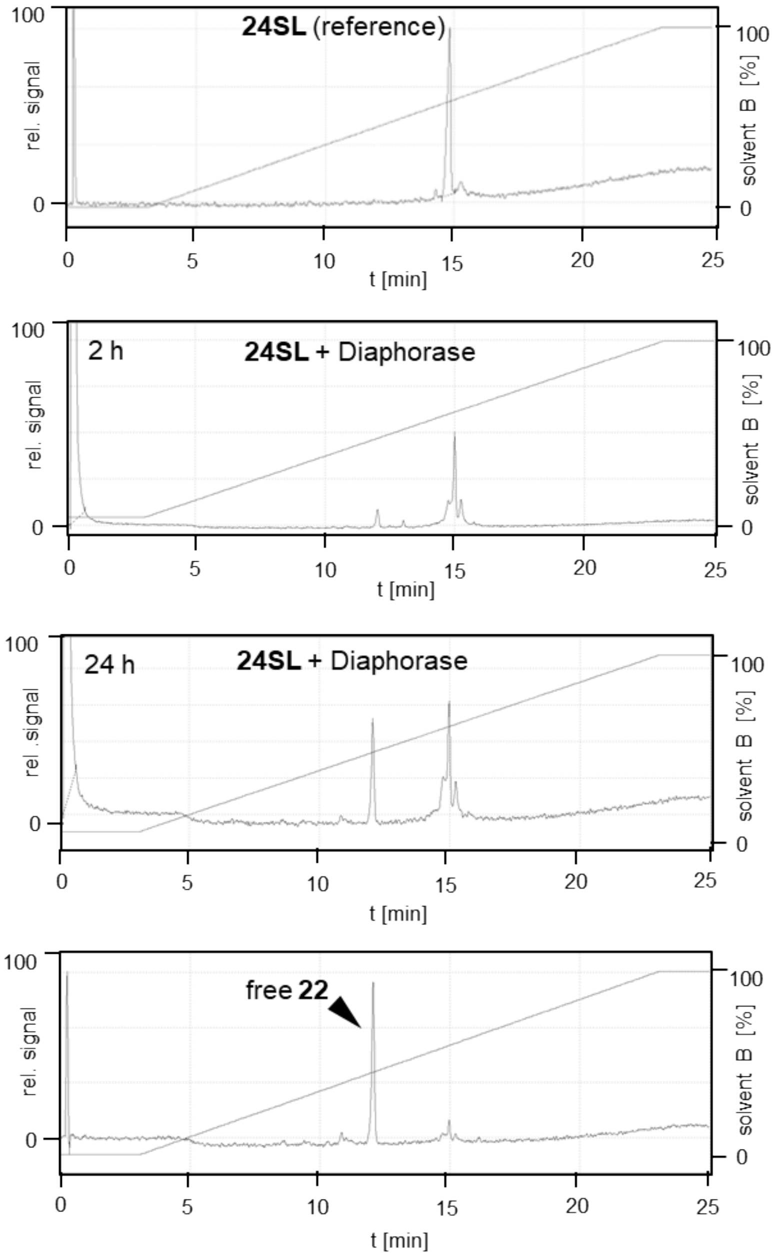

Figure S23. Qualitative information on the enzymatic cleavage of DFO TML rifamycin S conjugate **24SL** with Diaphorase, relative UV (220 nm) signal intensity, normalized to the highest peak. Analytical HPLC runs from compound **24SL** at t = 0, 2 h and 24 h for diaphorase addition. Reference injection of free payload **22** at the bottom, enzymatic reactions were quenched by 1:1 dilution of reaction with HPLC-grade MeOH.

Table S1. MIC values of published rifamycin derivatives, with a general structure of the rifamycin macrolide core (red = ansa-bridge, blue = aromatic core) above of the table.[9, 10, 11, 12]

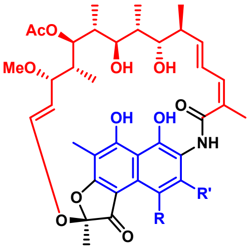

Name	R	R'	MIC *S aureus* (µg/mL)	MIC *E. coli* (µg/mL)
**rifamycin SV**	OH	H	0.032	8-32
**rifamycin B**	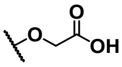	H	≤0.2-0.08	≥16-32
**rifampicin**	OH	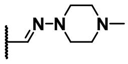	≤0.01-0.1	4-16
**rifabutin**	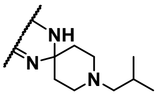		≤0.05	≥25
**rifalazil**	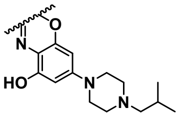		0.002-0.005	≥8-16

Table S2. MIC values of published sorangicin A amide derivatives, adapted from Jansen et al., with a general structure of sorangicin A above the table.[13]

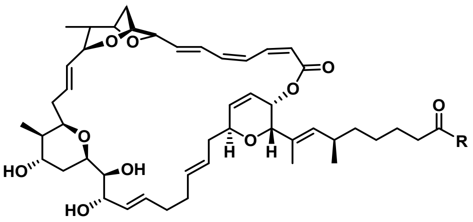

Name	R[1]	MIC *S. aureus* (µg/mL)	MIC *E. coli* (µg/mL)
**Sorangicin A**	OH	0.016-0.031	6-12
**Amide**	NH_2	0.125	-
***N*-methylamide**	NHMe	0.062	-
***N*-dimethylamide**	$N(Me)_2$	0.016	25
***N*-isopropylamide**	$N(iPr)_2$	0.060	50
***N*-hexylamide**	$NH(CH_2)_5CH_3$	1	1000
***N*-benzylamide**	NHBn	0.065-0.125	-
***N*-methoxyamide**	NHOMe	0.008-0.016	25
***N*-methoxy-*N*-methylamide**	NMe(OMe)	0.000125	>200

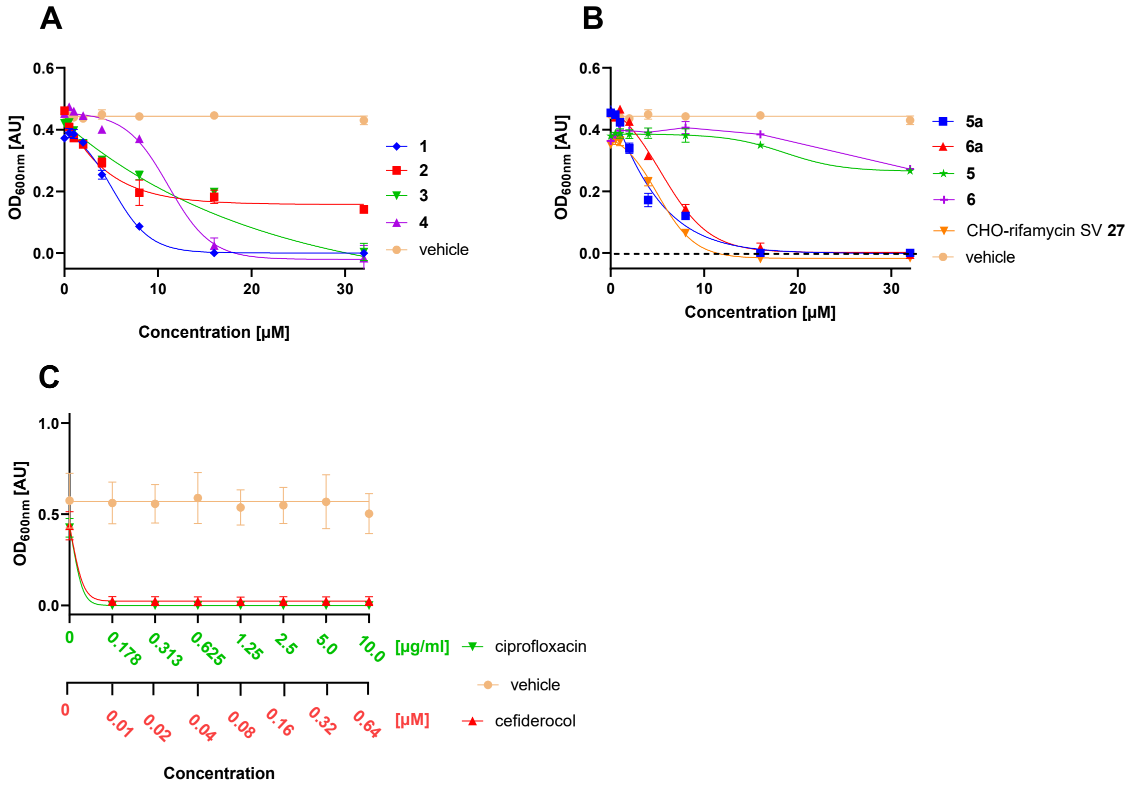

Figure S24. Antimicrobial activity of rifamycin conjugates **1** to **6** in MDR *E. coli* in iron-depleted, cation-adjusted medium (IDCAM), over 18 hours at 37 °C, error bars correspond to ± standard error of mean (SEM), n=2.

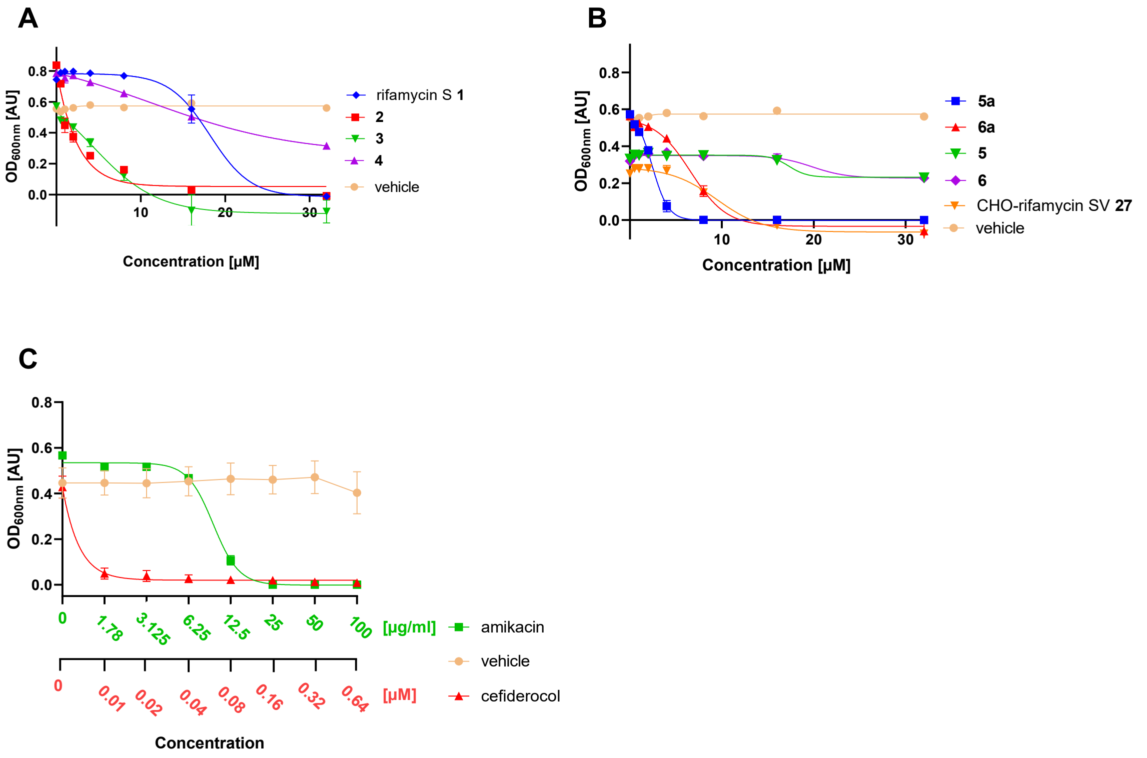

Figure S25. Antimicrobial activity of rifamycin conjugates **1** to **6** in MDR *P. aeruginosa* in iron-depleted, cation-adjusted medium (IDCAM), over 18 hours at 37 °C, error bars correspond to ± standard error of mean (SEM), n = 2.

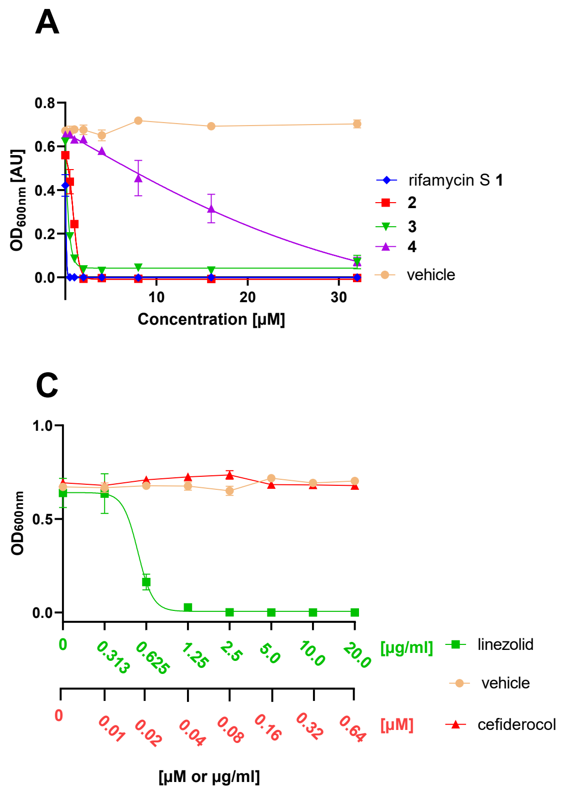

Figure S26. Antimicrobial activity of rifamycin conjugates **1** to **6** in MDR *S. aureus* in iron-depleted, cation-adjusted medium (IDCAM), over 18 hours at 37 °C, error bars correspond to ± standard error of mean (SEM), n = 2.

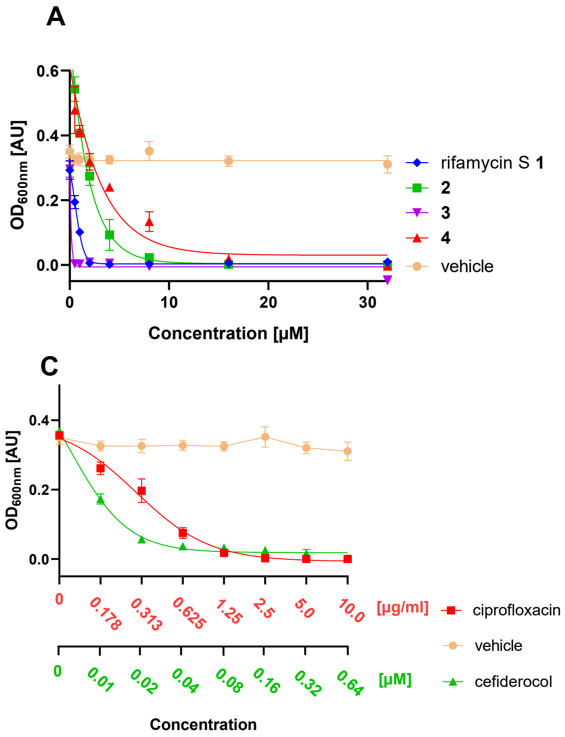

Figure S27. Antimicrobial activity of rifamycin conjugates **1** to **6** in MDR *A. baumannii* (DSM30007*)* in iron-depleted, cation-adjusted medium (IDCAM), over 18 hours at 37 °C, error bars correspond to ± standard error of mean (SEM), n = 2.

Table S3. MIC values[a] for **1** to **6** in four bacterial strains.

Compound	*E. coli*	*P. aeruginosa*	*S. aureus*	*A. baumannii*
rifamycin S **1**	>32	32	≤0.5	2
**2**	16	16	2	8
**3**	16	32	2	0.5
**4**	20	>32	32	16
formyl rif. SV **27**	8	16	≤0.5	-
**5a**	16	8	4	16
**5**	>32	>32	>32	>32
**6a**	16	16	4	16
**6**	>32	>32	>32	>32
cefiderocol	0.01	0.01	>0.64	0.04
ciprofloxacin	0.5	-	-	3.78
amikacin	-	25	-	-
linezolid	-	-	1.25	-

a values are given in [µM], controls in [µg/mL], except cefiderocol [µM].

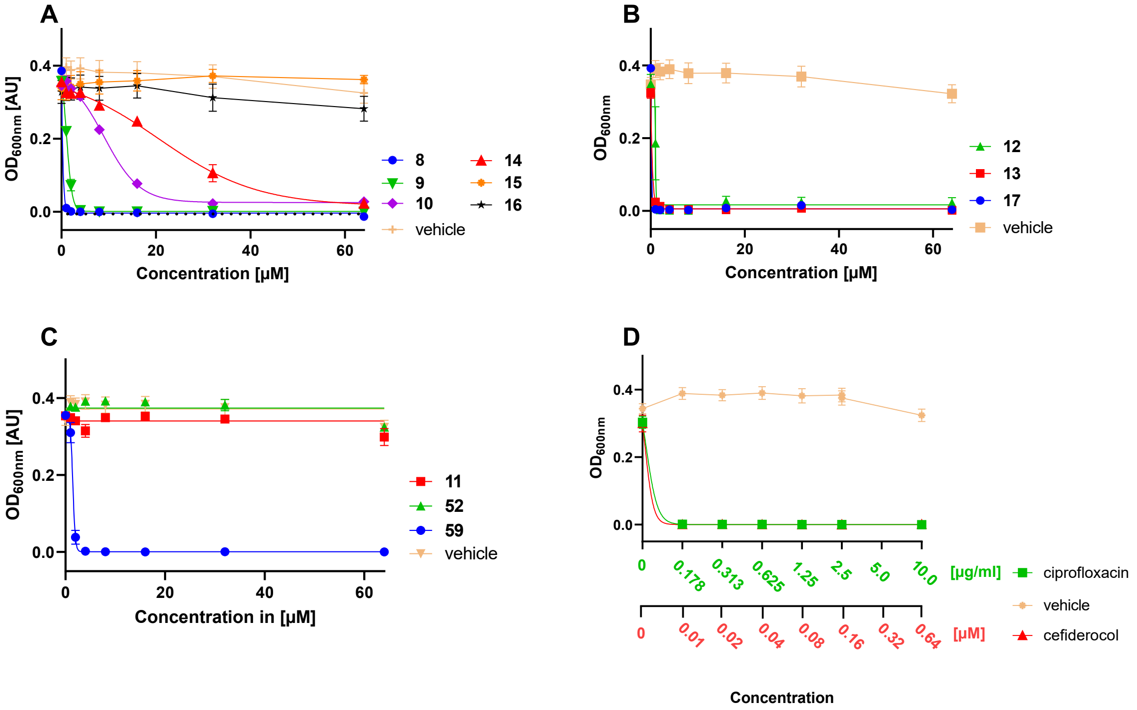

Figure S28. Antimicrobial activity of cleavable ciprofloxacin conjugates **8** to **17** in MDR *E. coli.* (A) ciprofloxacin TML / *para*-nitro TA catechol conjugates **8-10**, **13-15**, (B) ciprofloxacin TML / *para*-nitro TA DFO conjugates, (C) ciprofloxacin TML DOTAM with methylated catechols **17**, TML linker **52** and ciprofloxacin TML intermediate **59**, (D) antibiotic controls. Experiments were conducted in iron-depleted, cation-adjusted medium (IDCAM) over 18 hours at 37 °C. Error bars correspond to ± standard error of mean (SEM), n = 2-4.

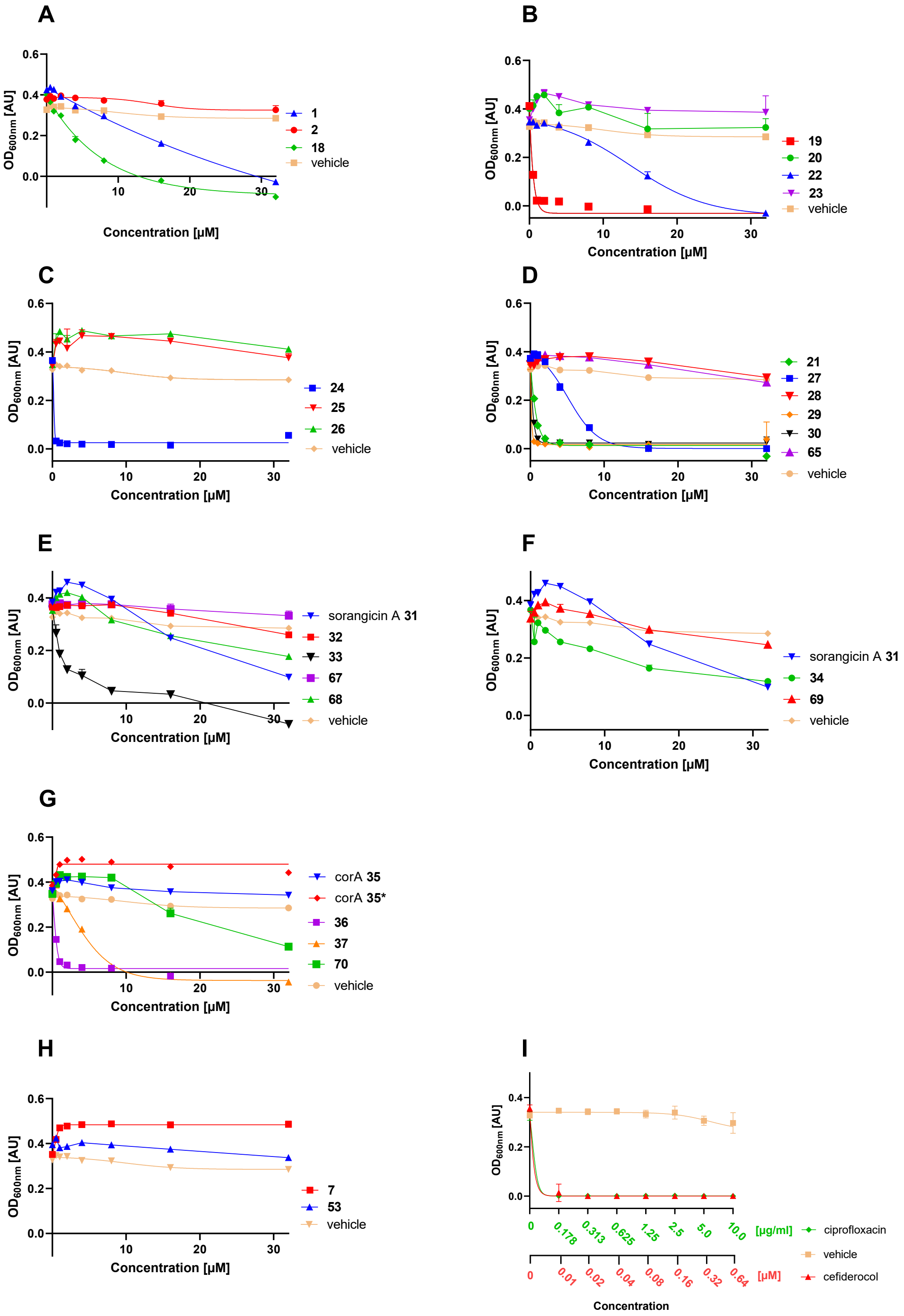

Figure S29 Antimicrobial activity of cleavable conjugates and control substances in MDR *E. coli.* (A-C) fluoro rifamycin compounds, (D) formyl rifamycin compounds, (E-F) sorangicin A compounds and (G) corallopyronin A compounds, (H) siderophore **7** and TML linker **53**, (I) control antibiotics cefiderocol and ciprofloxacin, in iron-depleted, cation-adjusted medium (IDCAM) over 18 hours at 37 °C, error bars correspond to ± standard error of mean (SEM), n = 2, corallop. A = corallopyronin A without exposure to basic conditions, corA* = reisolated from basic reaction conditions.

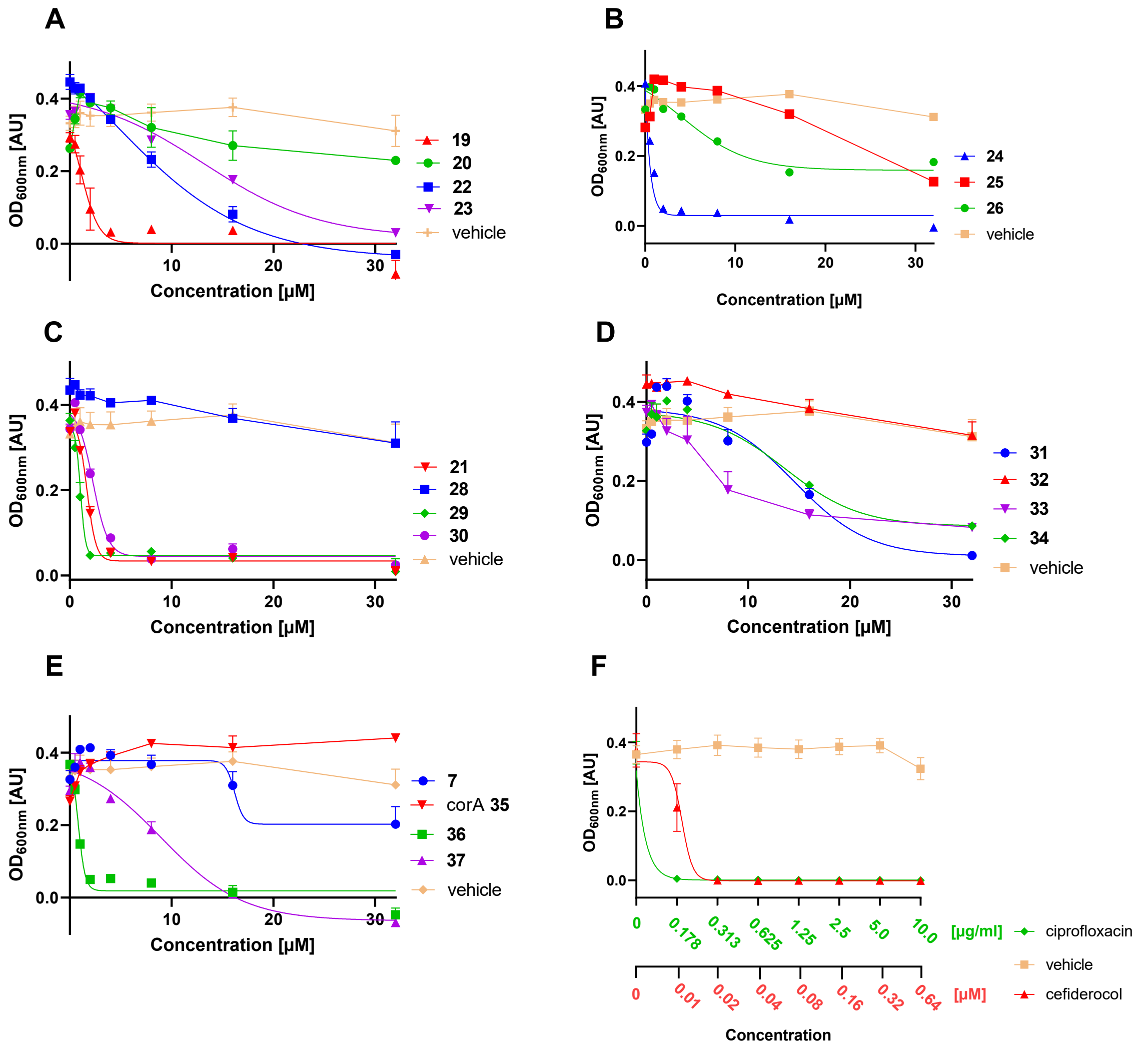

Figure S30. Antimicrobial activity of cleavable conjugates and control substances in siderophore-deficient *E. coli ΔentA* (A-B) fluoro rifamycin compounds, (C) formyl rifamycin compounds, (D) sorangicin A compounds and (E) corallopyronin A compounds and controls, (F) control antibiotics cefiderocol and ciprofloxacin, in iron-depleted, cation-adjusted medium (IDCAM) over 18 hours at 37 °C, error bars correspond to ± standard error of mean (SEM), n = 2 corA = corallopyronin A without exposure to basic conditions.

Table S4. MIC values[a] for **18** to **37** and controls in MDR *E. coli* and *E. coli ΔentA*.

Compound	Chelator	Effector	*E. coli*	*E. coli ΔentA*
**1**	-	RifS	>32	-
**2**	-	RifS	>32	>32
**7**	DOTAM	-	>32	>32
**18**	DFO	RifS	16	-
**19**	DFO	RifS	8	4
**20**	DFO-Ga	RifS	>32[GE]	>32
**21**	DFO	CHO-RifSV	4	4
**22**	-	RifS	>32	32
**23**	DOTAM	RifS	>32[GE]	32
**24**	DFO	RifS	1	4
**25**	DOTAM	RifS	>32	>32
**26**	DOTAM	RifS	>32	>32
**27**	-	CHO-RifSV	16	-
**28**	-	CHO-RifSV	>32	>32
**29**	DFO	CHO-RifSV	1	2
**30**	DFO	CHO-RifSV	2	8
**31**	-	SorA	>32	32
**32**	-	SorA	>32	>32
**33**	DFO	SorA	16	>32
**34**	DFO	SorA	>32	>32
**35**	-	CorA	>32[GE]	-
**35***	-	CorA	>32	>32
**36**	DFO	CorA	2	2
**37**	DFO	CorA	16	32
**53**	-	-	>32	-
**65**	-	CHO-RifSV	>32	-
**67**	-	SorA	>32	-
**68**	-	SorA	>32	-
**69**	-	SorA	>32	-
**70**	-	CorA	>32	-
cefiderocol			0.01	0.02
ciprofloxacin			0.538	0.538
amikacin			-	-
linezolid			-	-

a [µM] for test compounds and cefiderocol, [µg/mL] for ciprofloxacin, amikacin, linezolid, DFO = desferrioxamine, RifSV=rifamycin S, CHO RifSV=3-formyl rifamycin SV, SorA=sorangicin A, CorA=corallopyronin A, * = MIC f. CorA with base exposition, GE = growth enhancing.

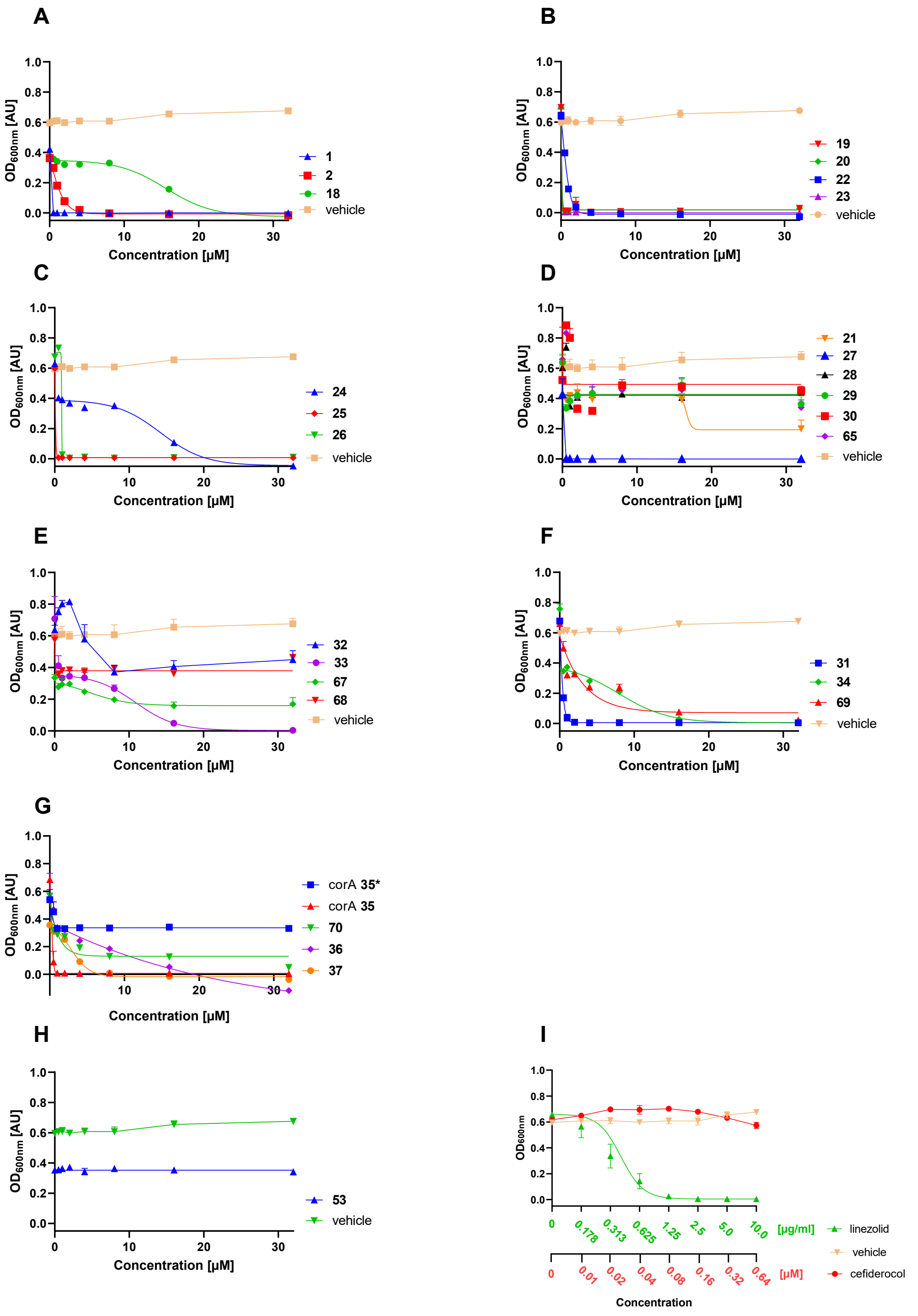

Figure S31. Antimicrobial activity of cleavable conjugates and control substances in MDR *S. aureus* (A-C) fluoro rifamycin compounds, (D) formyl rifamycin compounds, (E-F) sorangicin A compounds and (G) corallopyronin A compounds, (H) control linker **53**, (I) control antibiotics cefiderocol and ciprofloxacin, in iron-depleted, cation-adjusted medium (IDCAM) over 18 hours at 37 °C, error bars correspond to ± standard error of mean (SEM), n = 2, corallop. corA = corallopyronin A without exposure to basic conditions, corA* = corallopyronin A exposure to basic conditions.

Table S5. MIC values[a] for **18** to **37** and controls in MDR *S. aureus*.

Compound	Chelator	Effector	*S. aureus*
**1**	-	RifS	≤0.5
**2**	-	RifS	4
**7**	DOTAM	-	-
**18**	DFO	RifS	32
**19**	DFO	RifS	1
**20**	DFO-Ga	RifS	1
**21**	DFO	CHO-RifSV	>32
**22**	-	RifS	4
**23**	DOTAM	RifS	1
**24**	DFO	RifS	32
**25**	DOTAM	RifS	1
**26**	DOTAM	RifS	2
**27**	-	CHO-RifSV	≤0.5
**28**	-	CHO-RifSV	>32
**29**	DFO	CHO-RifSV	>32
**30**	DFO	CHO-RifSV	>32
**31**	-	SorA	2
**32**	-	SorA	>32[GE]
**33**	DFO	SorA	32
**34**	DFO	SorA	16
**35***	-	CorA	>32
**35**	-	CorA	2
**36**	DFO	CorA	32
**37**	DFO	CorA	8
**53**	-	-	>32
**65**	-	CHO-RifSV	>32
**67**	-	SorA	>32
**68**	-	SorA	>32
**69**	-	SorA	≥32
**70**	-	CorA	>32
cefiderocol			>0.64
ciprofloxacin			-
amikacin			-
linezolid			1.25

a [µM] for test compounds and cefiderocol, [µg/mL] for ciprofloxacin, amikacin, linezolid, DFO = desferrioxamine, RifS=rifamycin S, CHO RifSV=3-formyl rifamycin SV, SorA=sorangicin A, CorA=corallopyronin A, * = MIC f. CorA with base exposition, GE = growth enhancing.

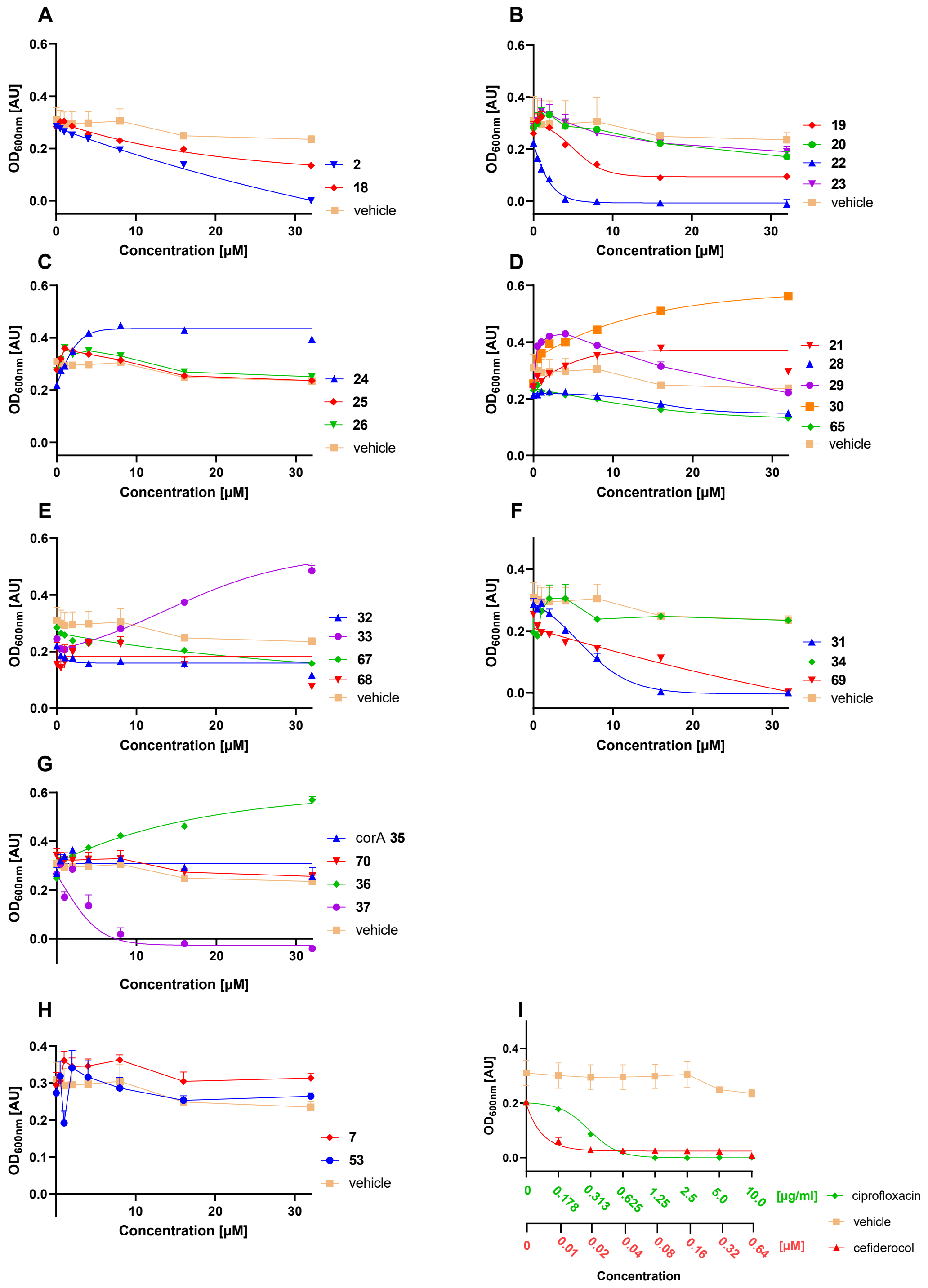

Figure S32. Antimicrobial activity of cleavable conjugates and control substances in MDR *A. baumannii* (DSM30007). (A-C) fluoro rifamycin compounds, (D) formyl rifamycin compounds, (E-F) sorangicin A compounds and (G) corallopyronin A compounds, (H) control linker **53** and siderophore **7**, (I) control antibiotics cefiderocol and ciprofloxacin, in iron-depleted, cation-adjusted medium (IDCAM) over 18 hours at 37 °C, error bars correspond to ± standard error of mean (SEM), n = 2, corallop. A = corallopyronin A without exposure to basic conditions.

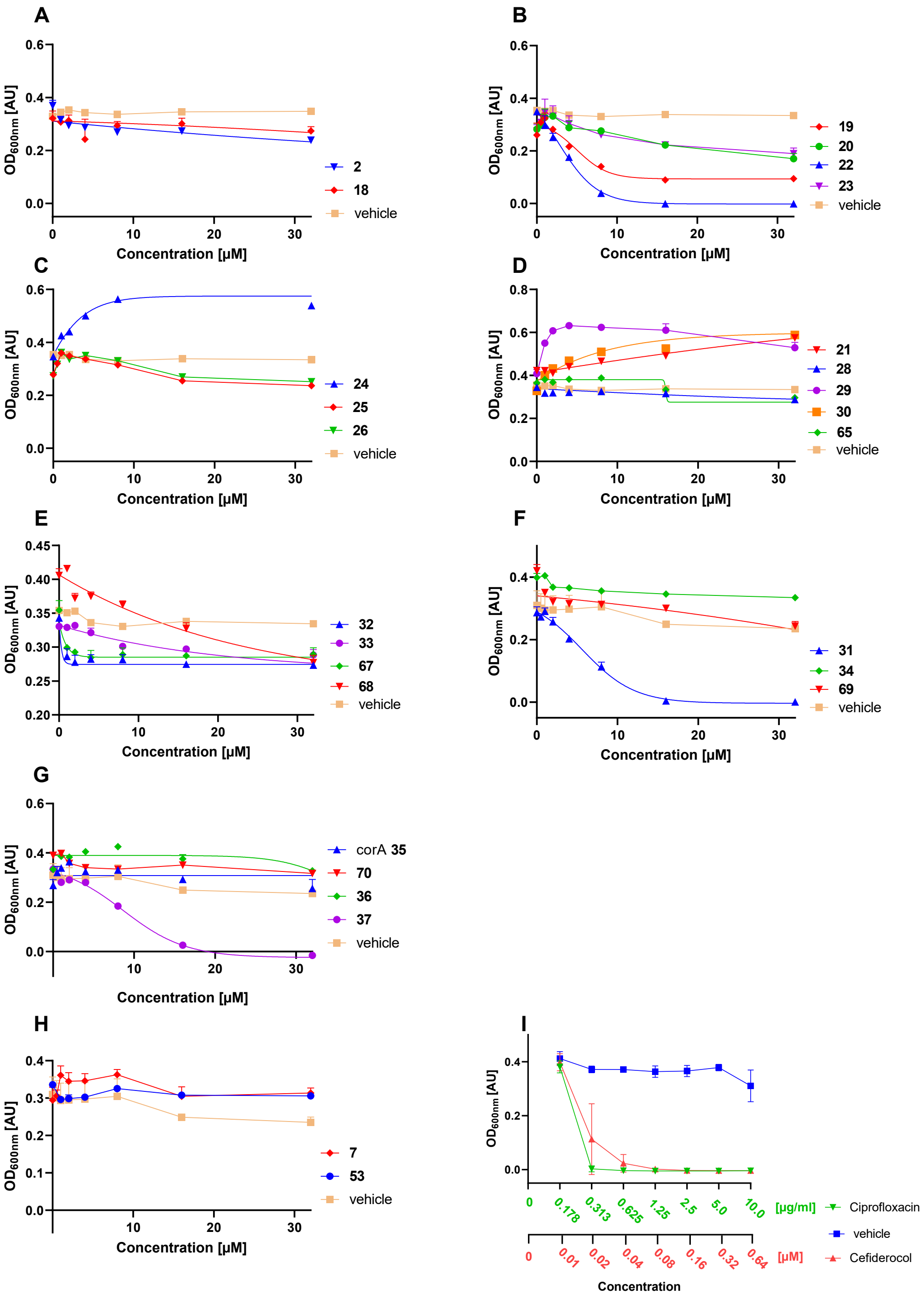

Figure S33. Antimicrobial activity of cleavable conjugates and control substances in MDR *A. baumannii* (DSM30008). (A-C) fluoro rifamycin compounds, (D) formyl rifamycin compounds, (E-F) sorangicin A compounds and (G) corallopyronin A compounds, (H) control linker **53** and siderophore **7**, (I) control antibiotics cefiderocol and ciprofloxacin, in iron-depleted, cation-adjusted medium (IDCAM) over 18 hours at 37 °C, error bars correspond to ± standard error of mean (SEM), n = 2, corallop. A = corallopyronin A without exposure to basic conditions, corallop. A* = reisolated from basic reaction conditions.

Table S6. MIC values[a] for **18** to **37** and controls in MDR *A. baumannii* DSM3007 and DSM30008.

**Compound**	**Chelator**	**Effector**	*DSM30007*	*DSM30008*
**2**	-	RifS	32	>32
**7**	DOTAM	-	>32	>32
**18**	DFO	RifS	>32	>32
**19**	DFO	RifS	>32	>32
**20**	DFO-Ga	RifS	>32	>32
**21**	DFO	CHO-RifSV	>32 GE	>32
**22**	-	RifS	8	32
**23**	DOTAM	RifS	>32	>32
**24**	DFO	RifS	>32GE	>32GE
**25**	DOTAM	RifS	>32	>32
**26**	DOTAM	RifS	>32	>32
**28**	-	CHO-RifSV	>32GE	>32
**29**	DFO	CHO-RifSV	>32	>32GE
**30**	DFO	CHO-RifSV	>32GE	>32GE
**31**	-	SorA	16	32
**32**	-	SorA	>32	>32
**33**	DFO	SorA	>32GE	>32GE
**34**	DFO	SorA	>32	>32
**35***	-	CorA	>32	>32
**35**	-	CorA	>32	>32
**36**	DFO	CorA	>32GE	>32GE
**37**	DFO	CorA	8	32
**53**	-	-	>32	>32
**65**	-	CHO-RifSV	>32	>32
**67**	-	SorA	>32	>32
**68**	-	SorA	>32	>32
**69**	-	SorA	≥32	>32
**70**	-	CorA	>32	>32
cefiderocol			0.02	0.64
ciprofloxacin			1.25	5.0
amikacin			-	-
linezolid			-	-

a [µM] for test compounds and cefiderocol, [µg/mL] for ciprofloxacin, amikacin, linezolid, DFO = desferrioxamine, RifS=rifamycin S, CHO RifSV=3-formyl rifamycin SV, SorA=sorangicin A, CorA=corallopyronin A, * = MIC f. CorA with base exposition, GE = growth enhancing.

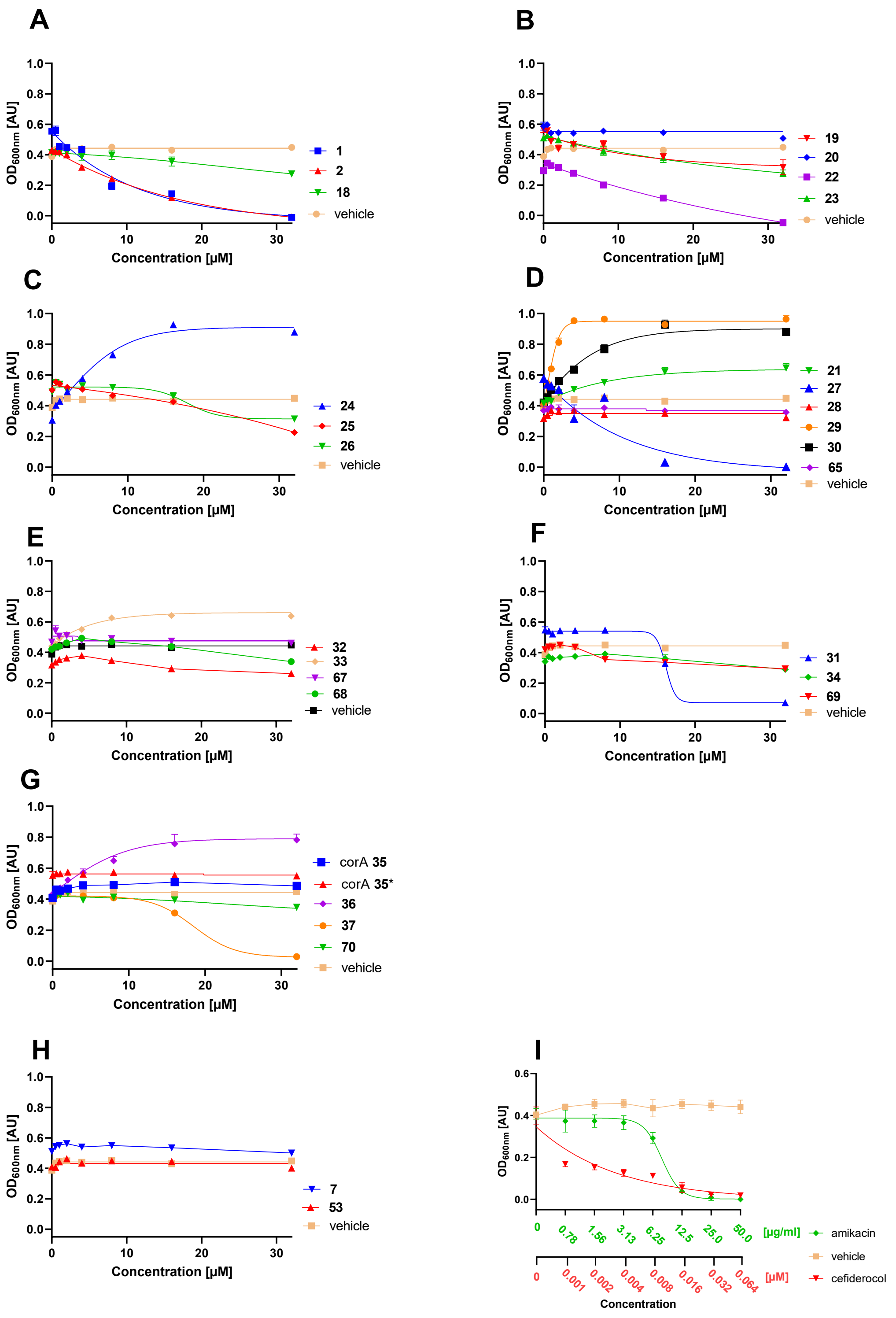

Figure S34. Antimicrobial activity of cleavable conjugates and control substances in MDR *P. aeruginosa.* (A-C) fluoro rifamycin compounds, (D) formyl rifamycin compounds, (E-F) sorangicin A compounds and (G) corallopyronin A compounds, (H) control linker **53** and siderophore **7**, (I) control antibiotics cefiderocol and ciprofloxacin, in iron-depleted, cation-adjusted medium (IDCAM) over 18 hours at 37 °C, error bars correspond to ± standard error of mean (SEM), n = 2, corallop. A* = corallopyronin A with exposure to basic conditions.

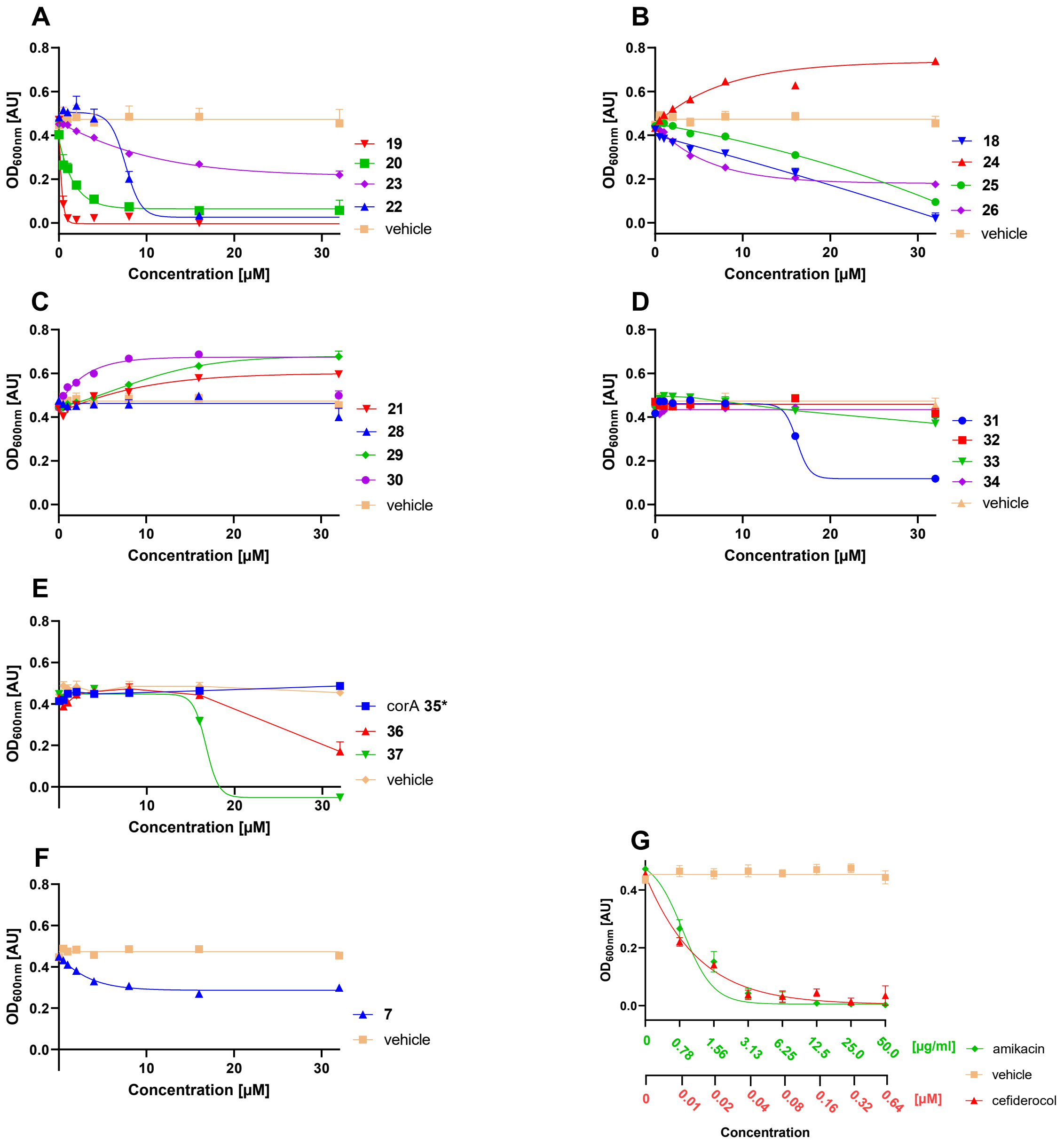

Figure S35. Antimicrobial activity of cleavable conjugates and control substances in siderophore-deficient *P. aeruginosa Δpvdpch* (A-B) fluoro rifamycin compounds, (C) formyl rifamycin compounds, (D) sorangicin A compounds and (E) corallopyronin A compounds, (F) DOTAM **7** control, (G) control antibiotics cefiderocol and ciprofloxacin, in iron-depleted, cation-adjusted medium (IDCAM) over 18 hours at 37 °C, error bars correspond to ± standard error of mean (SEM), n = 2 corallop. A = fresh natural product, corallop. A* = reisolated from reaction.

Table S7. MIC values[a] for **18** to **37** and controls in MDR *P. aeruginosa and P. aeruginosa Δpvdpch*.

Compound	Chelator	Effector	*P. aeruginosa*	*PAO1 Δpvdpch*
**1**	-	RifSV	32	-
**2**	-	RifSV	32	-
**7**	DOTAM	-	>32	>32
**18**	DFO	RifSV	>32	≥32
**19**	DFO	RifSV	>32	4
**20**	DFO-Ga	RifSV	>32	8
**21**	DFO	CHO-RifSV	>32[GE]	>32[GE]
**22**	-	RifSV	32	16
**23**	DOTAM	RifSV	>32	>32
**24**	DFO	RifSV	>32[GE]	>32[GE]
**25**	DOTAM	RifSV	>32	>32
**26**	DOTAM	RifSV	>32	>32
**27**	-	CHO-RifSV	32	-
**28**	-	CHO-RifSV	>32	>32
**29**	DFO	CHO-RifSV	>32[GE]	>32[GE]
**30**	DFO	CHO-RifSV	>32[GE]	>32[GE]
**31**	-	SorA	≥32	>32
**32**	-	SorA	>32	>32
**33**	DFO	SorA	>32[GE]	>32
**34**	DFO	SorA	>32	>32
**35***	-	CorA	>32	-
**35**	-	CorA	>32	>32
**36**	DFO	CorA	>32[GE]	>32
**37**	DFO	CorA	≥32	32
**53**	-	-	>32	-
**65**	-	CHO-RifSV	>32	-
**67**	-	SorA	>32	-
**68**	-	SorA	>32	-
**69**	-	SorA	>32	-
**70**	-	CorA	>32	-
cefiderocol			0.064	0.08
ciprofloxacin			-	
amikacin			25	12.5
linezolid			-	

a [µM] for test compounds and cefiderocol, [µg/mL] for ciprofloxacin, amikacin, linezolid, DFO = desferrioxamine, RifS=rifamycin SV, CHO RifSV=3-formyl rifamycin SV, SorA=sorangicin A, CorA=corallopyronin A, GE = growth enhancing.

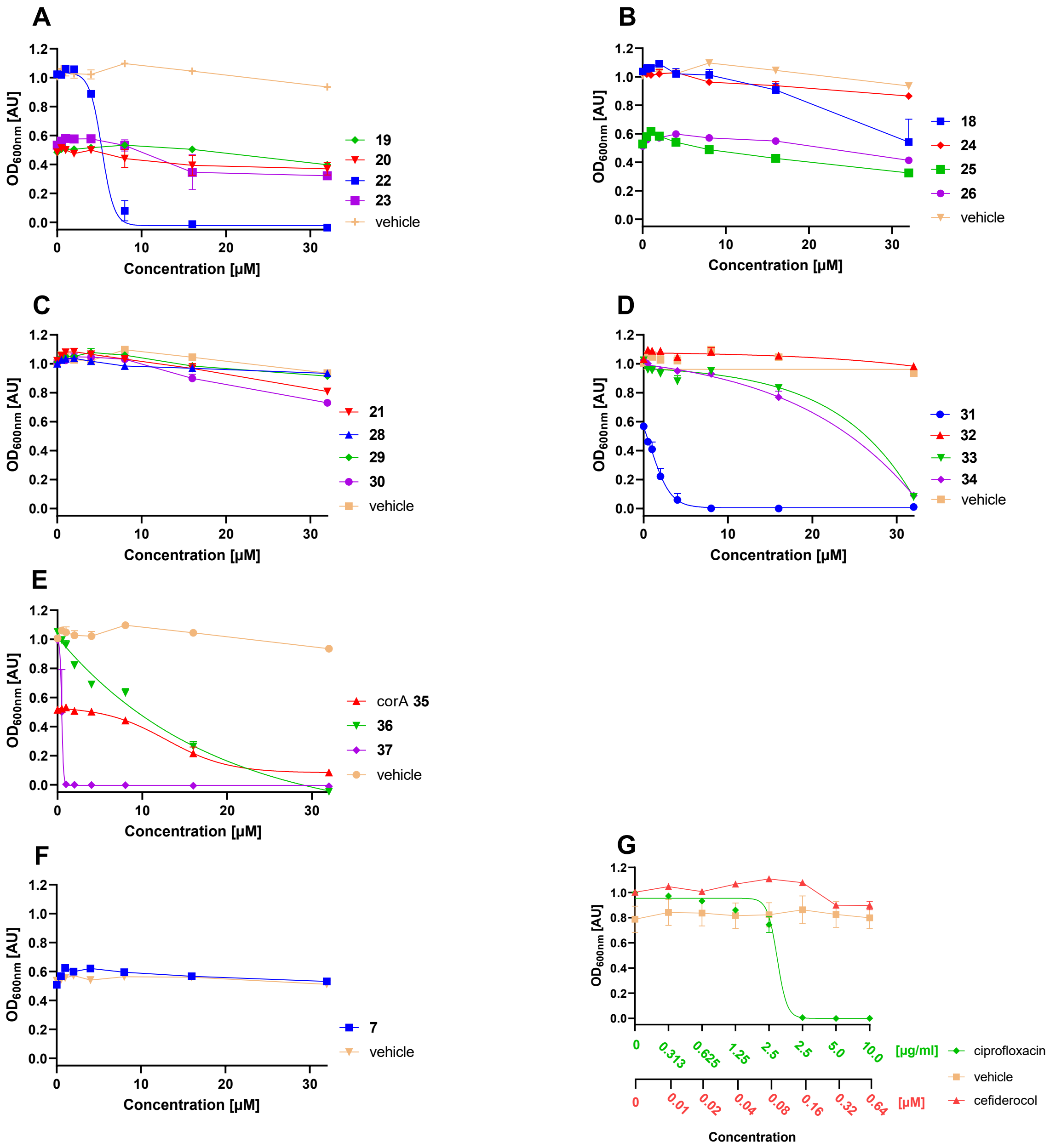

Figure S36. Antimicrobial activity of cleavable conjugates and control substances in MDR *E. faecium* (A-B) fluoro rifamycin compounds, (C) formyl rifamycin compounds, (D) sorangicin A compounds and (E) corallopyronin A compounds, (F) DOTAM **7** control, (G) control antibiotics cefiderocol and ciprofloxacin, in iron-depleted, cation-adjusted medium (IDCAM) over 18 hours at 37 °C, error bars correspond to ± standard error of mean (SEM), n = 2, corA = corallopyronin A without exposure to basic conditions.

Table S8. MIC values[a] for **18** to **37** and controls in MDR *E. faecium*.

Compound	Chelator	Effector	*E. faecium*
**7**	DOTAM	-	>32
**18**	DFO	RifSV	>32
**19**	DFO	RifSV	>32
**20**	DFO-Ga	RifSV	>32
**21**	DFO	CHO-RifSV	>32
**22**	-	RifSV	16
**23**	DOTAM	RifSV	>32
**24**	DFO	RifSV	>32
**25**	DOTAM	RifSV	>32
**26**	DOTAM	RifSV	>32
**28**	-	CHO-RifSV	>32
**29**	DFO	CHO-RifSV	>32
**30**	DFO	CHO-RifSV	>32
**31**	-	SorA	8
**32**	-	SorA	>32
**33**	DFO	SorA	32
**34**	DFO	SorA	32
**35**	-	CorA	32
**36**	DFO	CorA	32*
**37**	DFO	CorA	0.5
**53**	-	-	-
**65**	-	CHO-RifSV	-
**67**	-	SorA	-
**68**	-	SorA	-
**69**	-	SorA	-
**70**	-	CorA	-
cefiderocol			>0.64
ciprofloxacin			2.5
amikacin			-
linezolid			-

a [µM] for test compounds and cefiderocol, [µg/mL] for ciprofloxacin, amikacin, linezolid, DFO = desferrioxamine, DOTAM = enterobactin mimic, RifS=rifamycin SV, CHO-RifSV=3-formyl rifamycin SV, SorA=sorangicin A, CorA=corallopyronin A, * = MIC f. CorA with base exposition GE = growth enhancing

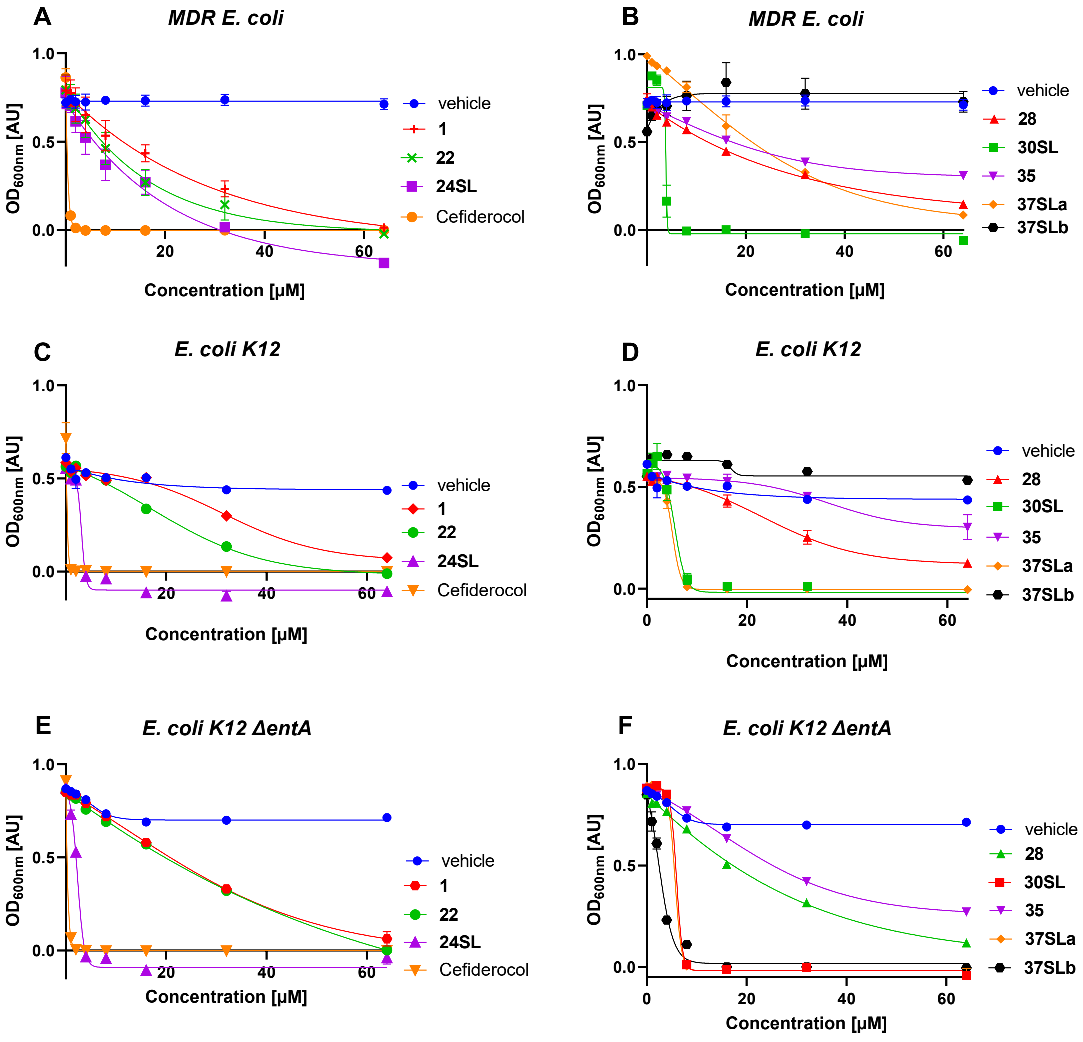

Figure S37. Antimicrobial activity of cleavable conjugates with shorter linkers and control substances in *E. coli* strains (A-B: *E. coli* MDR DSM1116,C-D: *E. coli* K12, E-F:*E coli ΔentA*), control antibiotics cefiderocol and rifamycin, in iron-depleted, cation-adjusted medium (IDCAM) over 18 hours at 37 °C, error bars correspond to ± standard error of mean (SEM), n = 2.

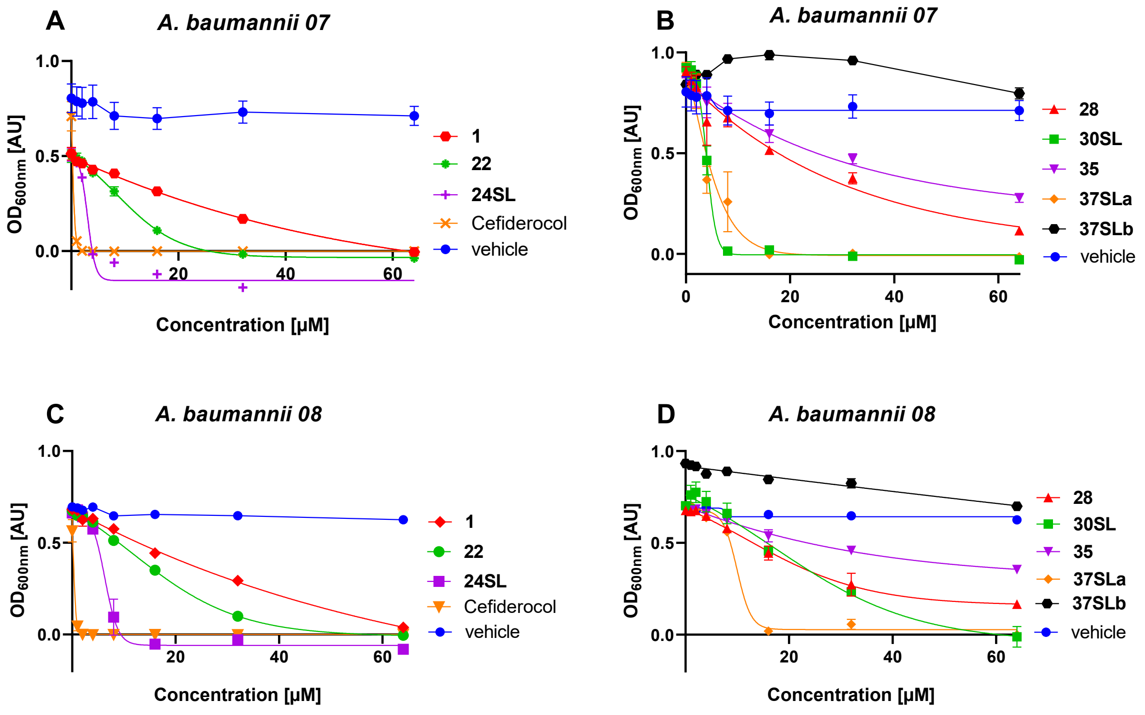

Figure S38. Antimicrobial activity of cleavable conjugates with shorter linkers and control substances in MDR A. baumannii strains (DSM30007 and DSM30008), control antibiotics cefiderocol and rifamycin, in iron-depleted, cation-adjusted medium (IDCAM) over 18 hours at 37 °C, error bars correspond to ± standard error of mean (SEM), n = 2.

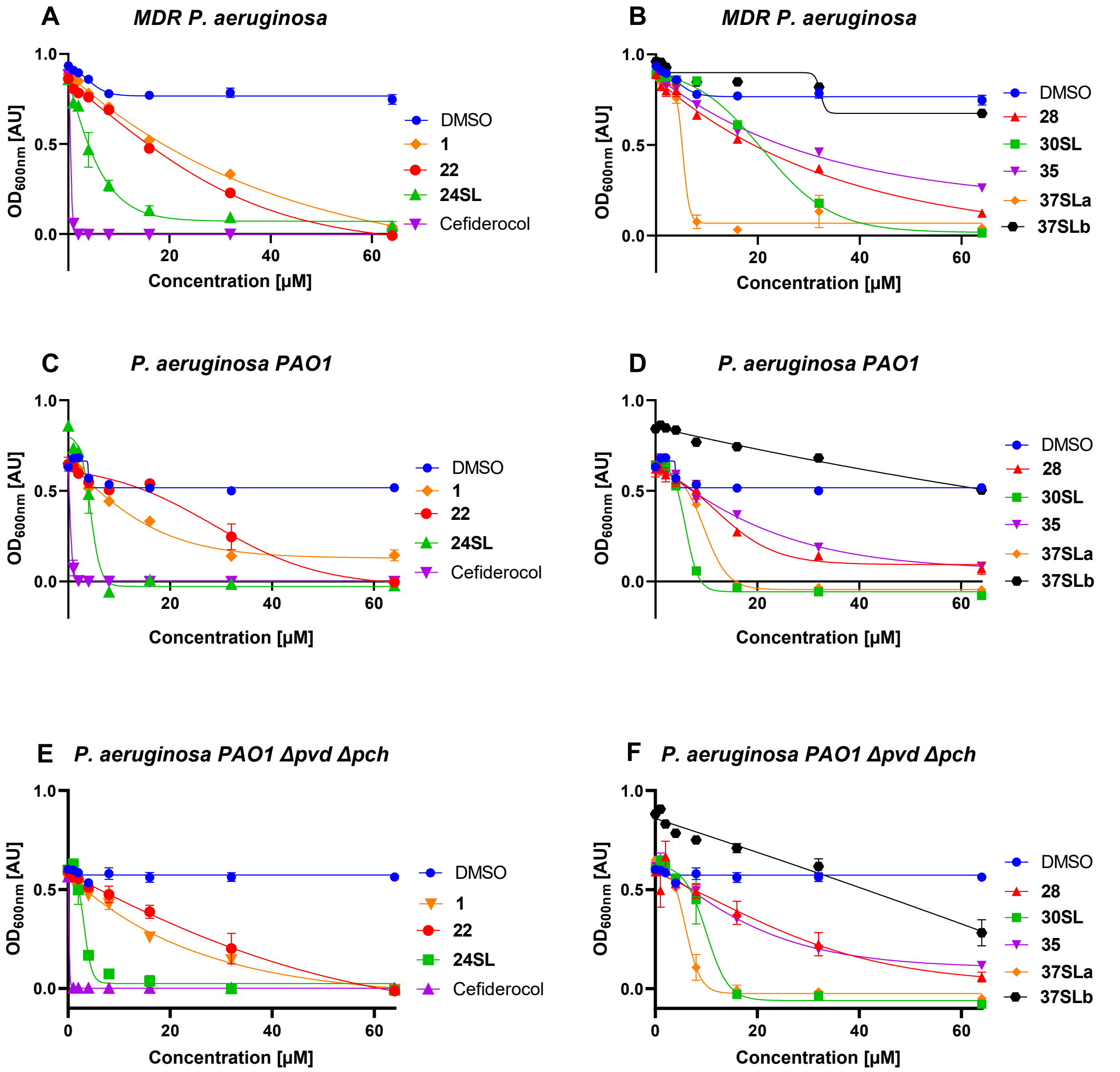

Figure S39. Antimicrobial activity of cleavable conjugates with shorter linkers and control substances in *P. aeruginosa* strains, control antibiotics cefiderocol and rifamycin, in iron-depleted, cation-adjusted medium (IDCAM) over 18 hours at 37 °C, error bars correspond to ± standard error of mean (SEM), n = 2.

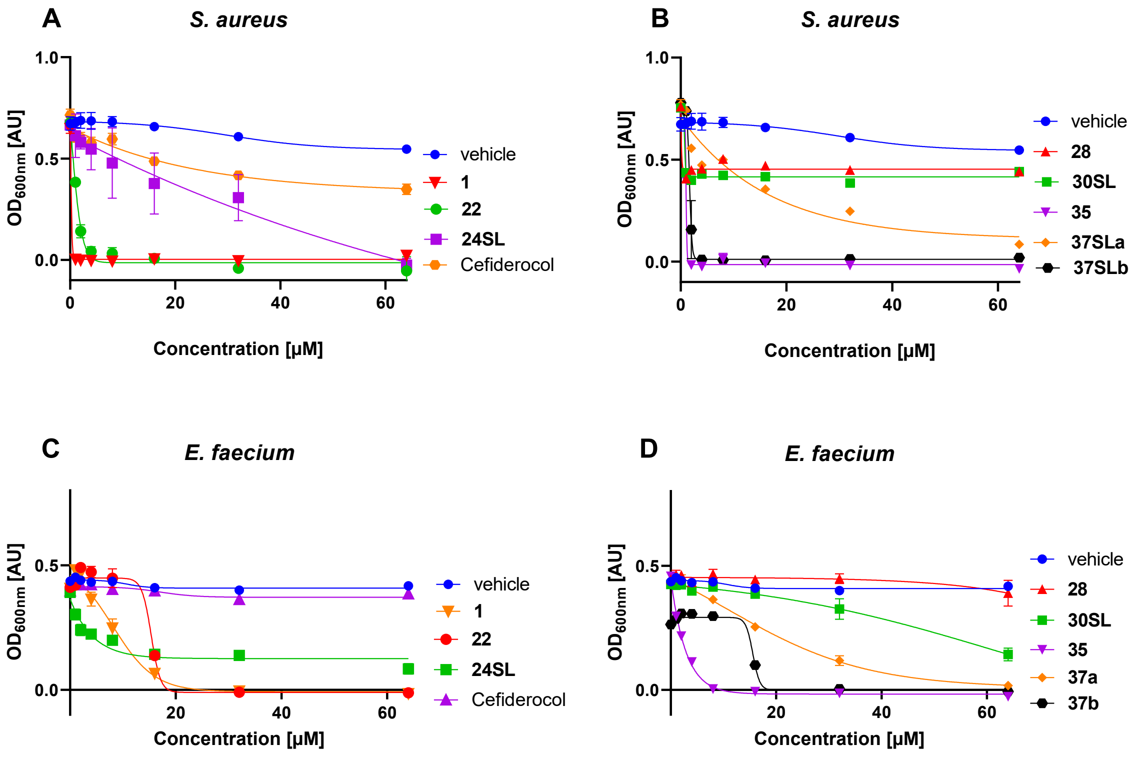

Figure S40. Antimicrobial activity of cleavable conjugates with a shorter linker and control substances in *S. aureus* and *E. faecium*, control antibiotics cefiderocol and rifamycin, in iron-depleted, cation-adjusted medium (IDCAM) over 18 hours at 37 °C, error bars correspond to ± standard error of mean (SEM), n = 2.

Table S9. MIC values[a] for **1, 22, 24SL, 28, 30SL, 37SLa/b** and controls in *E. coli* strains

Compound	***Chelator***	***Effector***	***E. coli MDR***	***E. coli K12***	***E. coli ΔentA***
**1**	-	RifS	64	≥64	64
**22**	-	RifS	64	64	64
**24SL**	DFO	RifS	64	8-16	4-8
**28**	-	CHO-RifSV	>64	>64	>64
**30SL**	DFO	CHO-RifSV	8	16	16
**35**	-	CorA	>64	>64	>64
**37SLa**	DFO	CorA	>64	16	16
**37SLb**	DFO	CorA	>64	>64	16
cefiderocol			*2*	*1*	*2*

a [µM] for test compounds and cefiderocol, DOTAM = enterobactin mimic, RifS=rifamycin S, CHO-RifSV=3-formyl rifamycin SV, CorA=corallopyronin A, GE = growth enhancing

Table S10. MIC values[a] for for **1, 22, 24SL, 28, 30SL, 37SLa/b** and controls in MDR *A. baumannii* strains

**Compound**	**Chelator**	**Effector**	***A. baumannii DSM30007***	***A. baumannii DSM30008***
**1**	-	RifS	64	64
**22**	-	RifS	32-64	64
**24SL**	DFO	RifS	32-64	16
**28**	-	CHO-RifSV	>64	>64
**30SL**	DFO	CHO-RifSV	8	64
**35**	-	CorA	>64	>64
**37SLa**	DFO	CorA	16	16
**37SLb**	DFO	CorA	>64[GE]	>64[GE]
cefiderocol			*2*	*2*

a [µM] for test compounds and cefiderocol, DOTAM = enterobactin mimic, RifS=rifamycin S, CHO-RifSV=3-formyl rifamycin SV, CorA=corallopyronin A, GE = growth enhancing

Table S11. MIC values[a] for **1, 22, 24SL, 28, 30SL, 37SLa/b** and controls in *P. aeruginosa* strains

Compound	Chelator	Effector	MDR P. aeruginosa	P. aeruginosa wildtype PAO1	P. aeruginosa Δpvd Δpch
**1**	-	RifS	≥64	≥64	64
**22**	-	RifS	64	64	64
**24SL**	DFO	RifS	32-64	8	16
**28**	-	CHO-RifSV	≥64	≥64	≥64
**30SL**	DFO	CHO-RifSV	64	16	16
**35**	-	CorA	>64	≥64	≥64
**37SLa**	DFO	CorA	16	16	16
**37SLb**	DFO	CorA	>64	>64	>64
cefiderocol			*≤1*	*2*	*2*

a [µM] for test compounds and cefiderocol, DOTAM = enterobactin mimic, RifS=rifamycin S, CHO-RifSV=3-formyl rifamycin SV, CorA=corallopyronin A, GE = growth enhancing

Table S12. MIC values[a] for for **1, 22, 24SL, 28, 30SL, 37SLa/b** and controls in MDR *S. aureus* and *E. faecium* strains

Compound	Chelator	Effector	*S. aureus*	*E. faecium*
**1**	-	RifS	<1	32
**22**	-	RifS	4	32
**24SL**	DFO	RifS	64	≥64
**28**	-	CHO-RifSV	>64	≥64
**30SL**	DFO	CHO-RifSV	>64	≥64
**35**	-	CorA	2	16
**37SLa**	DFO	CorA	≥64	16
**37SLb**	DFO	CorA	4	64
cefiderocol			*>64*	*>64*

a [µM] for test compounds and cefiderocol, DOTAM = enterobactin mimic, RifS=rifamycin S, CHO-RifSV=3-formyl rifamycin SV, CorA=corallopyronin A, GE = growth enhancing

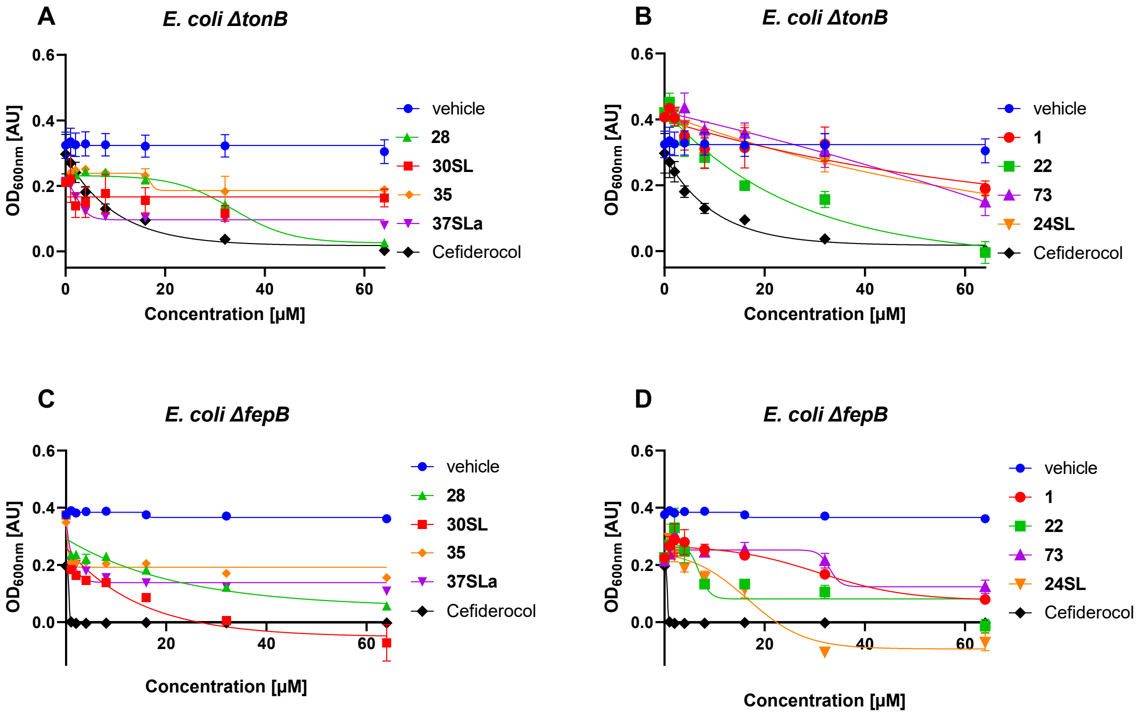

Figure S41 Antimicrobial activity of cleavable conjugates and control substances in *E. coli ΔfepB* (A-B) and *ΔtonB* mutants (C)-(D): (A/C) formyl rifamycin compounds, (B/D) fluoro rifamycin compounds, control antibiotics cefiderocol, in iron-depleted, cation-adjusted medium (IDCAM) over 18 hours at 37 °C, error bars correspond to ± standard error of mean (SEM). CorA = corallopyronin A without base exposition.

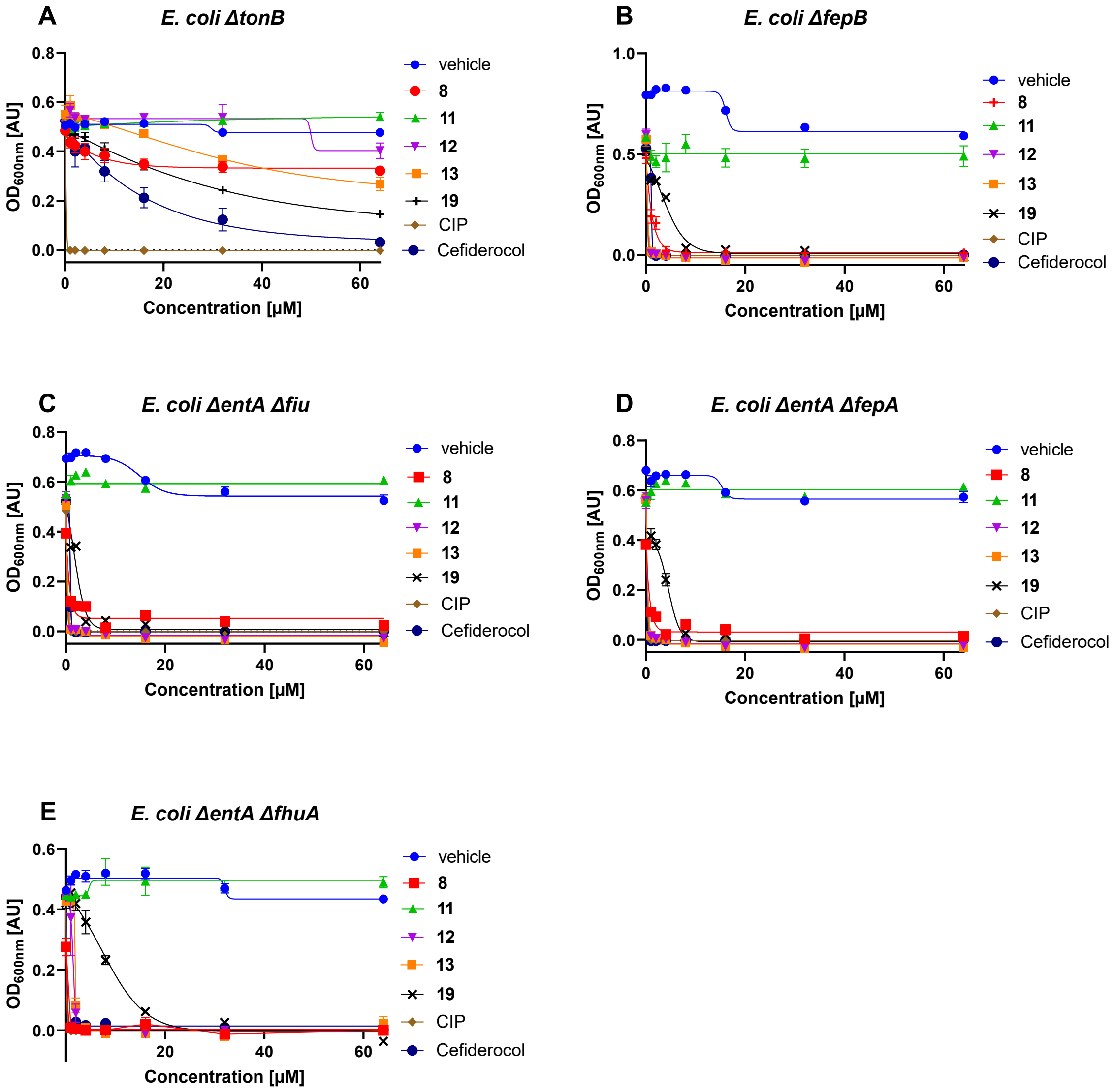

Figure S42. Antimicrobial activity of cleavable conjugates and control substances in *E. coli ΔtonB mutants* (A), *E. coli ΔfepB* (B), *E. coli ΔentAΔfiu* (C), *E. coli ΔentAΔfepA* (D), *E. coli ΔentAΔfhuA* (E) and: (A/C) formyl rifamycin compounds, (B/D) fluoro rifamycin compounds, control antibiotics cefiderocol, in iron-depleted, cation-adjusted medium (IDCAM) over 18 hours at 37 °C, error bars correspond to ± standard error of mean (SEM).

Table S13. MIC values[a] for selected conjugates and controls in *E. coli* mutants *ΔtonB* and *ΔfepB*.

Compound	Chelator	Effector	*E. coli ΔtonB*	*E. coli ΔfepB*
**1**	-	RifS	≥64	>64
**8**	DOTAM-Ac	CIP	>64	4
**11**	DOTAM-Me	CIP	>64	>64
**12**	DFO	CIP	>64	1
**13**	DFO	CIP	>64	1
**19**	DFO	RifS	>64	8
**22**	-	RifS	64	≥64
**24SL**	DFO	RifS	>64	32
**28**	-	CHO-RifSV	>64	≥64
**30SL**	DFO	CHO-RifSV	64	64
**35**	-	CorA	>64	>64
**37SLa**	DFO	Cor	>64	>64
cefiderocol			64	≤1
ciprofloxacin			≤1	≤1

a [μM] for test compounds and cefiderocol, DOTAM = enterobactin mimic, RifS=rifamycin S, CHO-RifSV=3-formyl rifamycin SV, CorA=corallopyronin A, GE = growth enhancing

Table S14. MIC values[a] for **8**, **11**, **12**, **13**, **19** in *E. coli* TBDT mutants *Δfiu, ΔfepA* and *ΔfhuA.*

Compound	Chelator	Effector	*E. coli ΔentA Δfiu*	*E. coli ΔentA ΔfepA*	*E. coli ΔentA ΔfhuA*
**8**	DOTAM-Ac	CIP	8	4	<1
**11**	DOTAM-Me	CIP	>64	>64	>64
**12**	DFO	CIP	≤1	2	4
**13**	DFO	CIP	≤1	2	2
**19**	DFO	RifS	4	8	32
cefiderocol			≤1	1	≤1
ciprofloxacin			≤1	≤1	≤1

a [μM] for test compounds and cefiderocol, DOTAM = enterobactin mimic, RifS=rifamycin S, CHO-RifSV=3-formyl rifamycin SV, CorA=corallopyronin A, GE = growth enhancing

Enzymatic quinone TML activation

This procedure was developed based on a publication by Pardeshi et al and our previous work in Peukert et al.[14, 15]

The NADH stock of NADH (500 mM in MQ water) was diluted 1:100 in 50 mM K_3PO_4 buffer (pH 7.0). The conjugates or control compounds in DMSO were diluted 5 mM NADH in phosphate buffer (final concentration = 150-300 µM) and the enzyme (2.5 µg/mL diaphorase or 1.25 µg/mL QOR2 final conc.) in buffer was added. The mixtures were incubated at 30 °C and 600 rpm. A compound preparation without enzyme was used as t_0 and separated with the sample analytical HPLC program. Samples for analytical HPLC (C18 gemini, 3 µm, NX-C18 110 A, 50 x 2 mm, ACN/H_2O, 0.1% TFA, DAD detector, 10% 3 min, 10-100% ACN 20 min, 100% ACN3 min) were quenched with an excess MeOH 1% AcOH. 20 µL / sample were injected and the chromatograms were compared to the reference measurements of the intact conjugates or free payloads at for their overlaid absorption at 220, 254 and 280 nm.

MIC assay in iron-depleted medium

The minimal inhibitory concentration was determined in iron-depleted, cation-adjusted medium (IDCAM), as previously described by L. Pinkert, Y. Lai et al and starved for ferric iron* before usage in the MIC assay as previously described by Peukert et al.[16, 17]

*Iron starvation: Inoculation of bacteria from glycerol stock in MHB-CHELEX medium overnight at 37 °C and 180 rpm. Then dilution (1:100) from overnight inoculum in 1xLMR medium without iron (see Peukert et al for medium composition[6]) and starvation for 24 h at 37 °C 180 rpm. Then dilution (1:10) of overnight culture in 1xLMR medium without iron and growth for 2-3 hours to reach exponential growth phase. Bacteria are then washed with 1xPBS (pH 7.4) and harvested by centrifugation (4500 rcf, 4 °C, 5 min). The pellet was resuspended in MHB-CHELEX and the OD600nm was adjusted to 0.01, before the dilution was employed in the MIC assay in a 1:1 dilution with the previously distributed compound dilution.

Due to their linker and/or their payload nearly all conjugates exhibited a high background signal at 600 nm. Thus the background signal (OD_{600nm} (0h)), after the addition of the bacteria to the compound dilutions, was subtracted from the OD_{600nm} (18h) measurements. This was necessary to allow the minimal inhibitory concentration (MIC) determination in the presence of these strongly colored compounds. However, due to medium evaporation and alteration of the compound and their absorption over 18 h, this caused negative values at high compound concentrations after the subtraction. In that case we used a tangent to the dose response curve to determine the MIC. In accordance with literature we define the MIC as the lowest concentration of an antibiotic that will inhibit the visible growth of a microorganism after overnight incubation.[18] For most compounds maximum 2% DMSO concentration were sufficient to solubilize them (variable DMSO concentration, highest 2% for highest compound concentration). For **73**, **24SL**, **30SL, 37Sla/b** 10% DMSO had to be used for compound dissolution for the highest compound concentration during the assay. The same concentration was always also applied for the DMSO control.

The MDR strains used in the MIC assays are shown in the table below. For the siderophore deficient strains (*E. coli ΔentA* and *P. aeruginosa ΔpvdΔpch*) see K. Ferreira et al and Peukert et al.[4, 19] For further information on the *E. coli* TBDT, TonB and PBP knockout mutants please see our previous publication by L. Pinkert, Y-H. Lai et al[20]:

Table S14. Bacterial strains used in the MIC assay.

Strain	DSMZ-#	Antibiotic Resistance	Medium
Escherichia coli[21]	DSM1116	Penicillin G, Oxacillin, Vancomycin, Lincomycin, Bacitracin, Clindamycin, Linezolid, Nystatin, Quinupristin, Teicoplanin, Piperacillin	MHB
Staphylococcus aureus[22]	DSM11822	Colistin, Kanamycin, Aztreonam, Oxacillin, Clindamycin, Nystatin, Lincomycin, Erythromycin, Norfloxacin, Pipedemic acid	TSY
Klebsiella pneumoniae[23]	DSM11678	-	MHB
Acinetobacter baumannii[24]	DSM30007	Penicillin G, Oxacillin, Ampicillin, Cefalotin, Cefazolin, Chloramphenicol, Vancomycin, Lincomycin, Pipedemic acid, Bacitracin, Clindamycin, Linezolid, Nystatin, Quinupristin, Teicoplanin,	MHB
Acinetobacter baumannii[25]	DSM30008	Penicillin G, Oxacillin, Ampicillin, Cefalotin, Cefazolin, Vancomycin, Lincomycin, Linezolid, Nystatin, Quinupristin, Teicoplanin	MHB
Pseudomonas aeruginosa[26]	DSM24068	Penicillin G, Oxacillin, Ampicillin, Mezlocillin, Cefalotin, Cefazolin, Cefotaxime, Chloramphenicol, Vancomycin, Erythromycin, Lincomycin, Ofloxacin, Norfloxacin, Pipedemic acid, Nitrofurantoin, Bacitracin, Kanamycin, Neomycin, Ceftriaxone, Clindamycin, Fosfomycin, Moxifloxacin, Linezolid, Nystatin, Quinupristin, Teicoplanin, Piperacillin	MHB
Enterococcus faecium[27]	DSM20477	Colistin, Polymyxin B, Pipedemic acid, Nystatin, Aztreonam	TSY

Table S15. Knockout mutants in this study

Strain	Source/Description	Antibiotic Resistance	Medium
E. coli K12 wildtype BW25113 parent	K. Ferreira et al[19]	Kanamycin	MHB
E. coli ΔentA	K. Ferreira et al[19]	Kanamycin	MHB
E. coli ΔentA ΔfepB	L. Pinkert, Y. H. Lai et al[20]	Chloramphenicol	MHB
E. coli ΔtonB	L. Pinkert, Y. H. Lai et al[20]	Kanamycin	MHB
E. coli ΔentA Δfiu	L. Pinkert, Y. H. Lai et al[20]	Chloramphenicol	MHB
E. coli ΔentA ΔfepA	L. Pinkert, Y. H. Lai et al[20]	Chloramphenicol	MHB
E. coli ΔentA ΔfhuA	L. Pinkert, Y. H. Lai et al[20]	Chloramphenicol	MHB
P. aeruginosa PAO1	DSM 22644[28]	-	MHB
P. aeruginosa PAO1 ΔpvdD ΔpchE-F	Gasser et al[28]	-	MHB

Measurement of Uptake of siderophore conjugates and payloads into Gram-negative bacteria by LC-MS/MS

Bacterial strain, cultivation and iron starvation

E. coli K12-BW25113 (wild-type) was obtained from the Coli Genetic Stock Center (CGSC, # 7636). The multidrug resistant strain (MDR) DSM1116 was used for the uptake of corallopyronin and its conjugate. A bacterial cultivation and iron-depletion workflow was followed to have comparable conditions to the MIC assays (see Figure S43). All cultures were incubated at 37 °C with shaking at 150 rpm. Pre-cultures were prepared in iron-depleted, cation-adjusted medium (MHB-CHELEX). These cultures were used to prepare overnight cultures in LMR with a start OD_{600} = 0.01, which were again cultivated overnight. Then the assay culture was prepared in fresh LMR (start OD_{600} = 0.1), and incubated until an OD_{600} = 0.5 was reached. The required volume of bacterial suspension (V1) was removed and the cells were pelleted by centrifugation at 4500 *g*, 4 °C and washed twice with an equal volume of LMR. In the meantime, 10 µL compound stock (1 mM in DMSO) was added to 15 mL falcon tubes. The washed cells were concentrated to OD_{600} = 1.0 by re-suspending in half of the initial volume (½V1) MHB-CHELEX and 10 mL of the resultant suspension added to each of the 15 mL falcon tubes and incubated for 10 min at 37°C, 150 rpm.

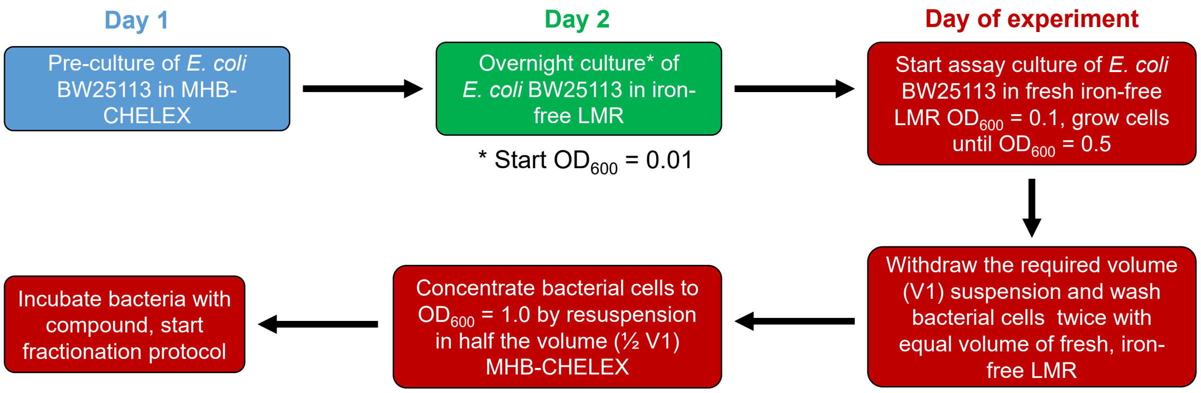

Figure S43. Workflow for the cultivation and iron starvation of bacteria foregoing the fractionation assay.

Generation of cell fractions and whole cell samples

The fractionation procedure was performed as previously described.[29] Post compound treatment, the cells were immediately collected by centrifugation at 4500 *g*, 5 min at 4°C. The supernatant was removed and the pelleted cells were re-suspended in 2 mL TBS (50 mM Tris-HCl pH 7.0, 135 mM NaCl, 2.5 mM KCl) and split in equal portions in two 2 mL Eppendorf tubes. Next, the tubes were centrifuged for 5 min at 4500 *g*, 4°C. The supernatant was removed and washed with 300 µl wash buffer (25 mM Tris HCl pH 7.4). After centrifugation as before, the cell pellet was re-suspended in 100 µl of the respective wash buffer and then 100 µl of a sucrose-EDTA solution (40% w/w in 25 mM Tris HCl pH 7.4 with 2 mM EDTA) was added, the

solutions were briefly mixed by inversion and treated for 5 min at room temperature. After treatment, the samples were centrifuged for 5 min at 3500 *g*, 4°C. After centrifugation, 200 µl of a mixture of 50% (v/v) of the respective wash buffer and the sucrose-EDTA solution was added to the pellets without disturbing them and they were settled by centrifugation at 3500 g, 4°C for 1 min, followed by removal of the supernatant. Next, the two tubes were used for different parts of the assay - one tube was used to prepare the whole cell sample, the second tube for the cell fractionation sample. For the whole cell sample, the pellet was re-suspended in 190 µl 10 mM Tris-HCl pH 7.4, and the sample was sonicated using a Bandelin Sonopuls sonifier with a MS2.5 tip twice for 1 kJ, cooling the sample on ice in between. For the fractionation sample, 200 µl 0.5 mM $MgSO_4$ solution was carefully added to the pellet, and then the sample was incubated on ice horizontally, with the pellet upwards, and the solution touching the pellet for 10 min. After the incubation on ice, the sample was centrifuged again for 10 min at 3500 *g*, 4°C. Subsequently, the supernatant was collected – this is the periplasmic fraction (PP). Next, the pellet was washed with 200 µl $MgSO_4$ solution without disturbing it and centrifuged for 1 min at 3500 *g*, 4°C. After removal of the supernatant, the pellet was treated in the 10 mM Tris-HCl buffer pH 7.4 and sonified as the whole-cell sample. For both samples, 10 µl DNA-mix (60 µg/ml in 1 M $MgCl_2$) was added and incubated at 37°C, 800 rpm for 15 min to digest the DNA in the samples. The whole-cell sample (WC) was stored until analysis, the fractionation sample was further divided into a cytoplasmic and membrane-containing fraction by centrifugation at 30.000 *g*, 4°C for 45 min. After centrifugation, the supernatant was collected, which is the cytoplasmic fraction (CP). The pelleted membranes were washed by addition of 200 µl 10 mM Tris-HCl pH 7.4 without re-suspending the pellet and centrifuged for 1 min at 16.000 *g*. The final supernatant was removed and the pellet re-suspended in 200 µl of 0.5 mM $MgSO_4$, this is the membranes fraction (M). Samples mock-treated with DMSO instead of compound were used to generate matrix for compound standard curves. All samples were stored at -20°C until preparation for LC-MS/MS analysis.

LC-MS/MS analysis

Standards were prepared in precipitation solution (320 µl 37.5% MeCN: 37.5% MeOH: 25% H_2O supplemented with 0.5% formic acid (final)) in appropriate concentrations to prepare comparable linear standard curves. These standards were supplemented with 80 µl mock-treated matrix in a 96-well deep well plate (Brand, BR701354). 80 µl of each sample was combined with 320 µl precipitation solution. The plate was sealed immediately and the contents mixed briefly at room temperature prior to centrifugation at 2250 *g* for 60 min. at 4°C. After centrifugation, 320 µl were transferred into a MTP with a V-shaped well bottom (Greiner Bio-One, 651201) and dried in a CentriVap fitted with a -80°C cold trap (Labconco, Kansas, MO, USA). The concentrated samples were re-suspended in 50 µl ACN:MeOH:H_2O (3:3:4) containing either 0.1% formic acid and 10 ng/ml caffeine (internal standard, positive mode) or

100 ng/mL glipizide (internal standard, negative mode). The samples were analyzed using a 1290 Infinity II LC System (Agilent Technologies, Santa Clara, CA, USA) with an AB SCIEX QTrap 6500 triple quadrupole mass spectrometer (AB SCIEX Germany GmbH, Darmstadt, Germany) in positive mode. 5 µl of each sample was injected on a Gemini® 3 µm NX-C18 110 Å 50 x 2 mm column (Phenomenex, Torrance, CA, USA) and separated with a gradient of H_2O and ACN, both supplemented with 0.1% formic acid. In brief, the LC profile consisted of an initial 1 min step at 95% H_2O, 5% ACN, followed by a gradient from 5% to 95% ACN over 4 min with a final step for 1 min at 95% ACN using a constant flow rate of 0.8 mL/min while the temperature of the column was stabilized to 25 °C. Compound **22** and caffeine were detected as $[M+H]^+$, while a fragment of m/z = 925.423 Da, assumed to be formed via McLafferty rearrangement (Figure S44), served to detect **24SL.** Glipizide and CorA **35** were detected in negative mode as the respective $[M-H]^-$ ions. Transitions are listed below in Table S15.

Table S15: MRMs used for MS/MS detection of compounds. * = fragment detected (see below); IS = internal standard.

ID	Mode	Q1 mass [Da]	Q3 mass [Da]	Time [ms]	DP [V]	EP [V]	CE [V]	CXP [V]
**Caffeine (IS)**	**Positive**							
Quantifier		195.116	138.1	50	66	10	27	10
Qualifier			110.1	50	66	10	31	6
**24SL***								
Quantifier		925.318	533.1	50	1	6	10	47
Qualifier / alternate quantifier			865.3	50	1	6	10	35
Alternate qualifier			390.9	50	1	6	10	95
**22**								
Quantifier		915.339	883.3	50	1	71	10	27
Qualifier		915.339	491.0	50	1	71	10	65
**Glipizide (IS)**	**Negative**							
Quantifier		443.936	319.1	50	-66	-10	-26	-21
Qualifier			170.1	50	-66	-10	-40	-7
**CorA**								
Quantifier		526.311	301.2	50	-85	-10	-32	-17
Qualifier			283.1	50	-85	-10	-34	-13
Alternate qualifier			163.2	50	-85	-10	-46	-11
Alternate qualifier			123.2	50	-85	-10	-38	-5

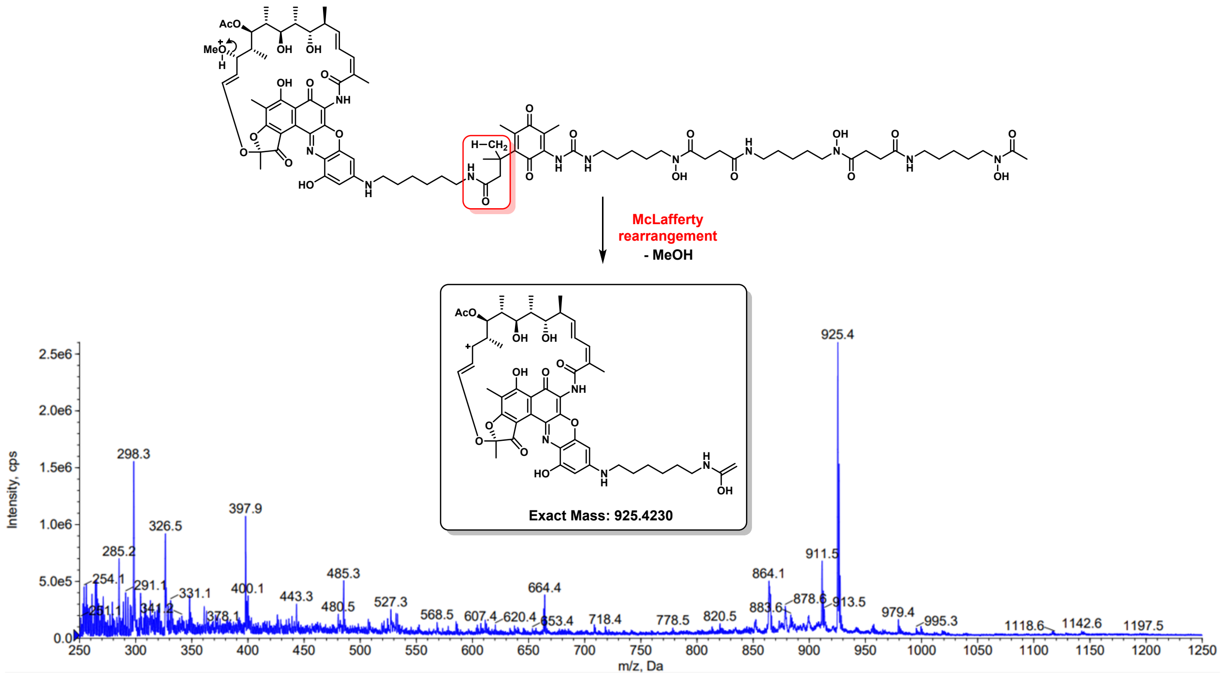

Figure S44. In the case of **24SL**, it is hypothesized that loss of MeOH and McLafferty rearrangement lead to the fragment at 925.4 Da, which was used for detection and quantification of **24SL**.

Uptake and release of corallopyronin A 35

Compound uptake was determined **35** and **36** in MDR *E. coli* DSM1116 at a concentration of 1 µM for both antibiotics. As highlighted earlier, **35** is prone to isomerisation, readily producing CorA' (**35')** and ultimately CorC (**35*)** after incubation in slightly acidic aqueous media (Figure S45B).[30, 31] Under the acidic workup conditions, both isomers were detected as additional peaks during the analysis of the samples, with the transitions used to detect **35** corresponding to the [M-H]- ions. Using specific analytical standards, the peaks were identified as CorA' (**35'**) and CorC **35***, respectively (data not shown). During accumulation experiments with **35**, a 50:50 mixture of **35** and **35*** was found in the cells, whereas equivalent experiments with conjugate **36** led to almost exclusively detection of **35***. We conclude that cyclisation must have occurred intracellularly post enzymatic cleavage or during workup. Mechanistically, we interpret that the enhanced formation of **35*** results from the release of the alcoholate after linker cleavage from **36**, followed by Michael addition to form the tetrahydrofurane. Thus the enhanced formation of **35*** is seen as indirect evidence for intracellular payload release. In previous studies, all three corallopyronin isomers had comparable bioactivites. In light of this, the sum of the three peak areas was used to provide the most reliable quantification of corallopyronin. Accordingly, it was found that solely negligible amounts of the natural product accumulated in the bacteria, while upon incubation with **36** an approx. 300-fold increase in the amount of corallopyronin internalised was observed in the whole-cell samples (see Figure S45).

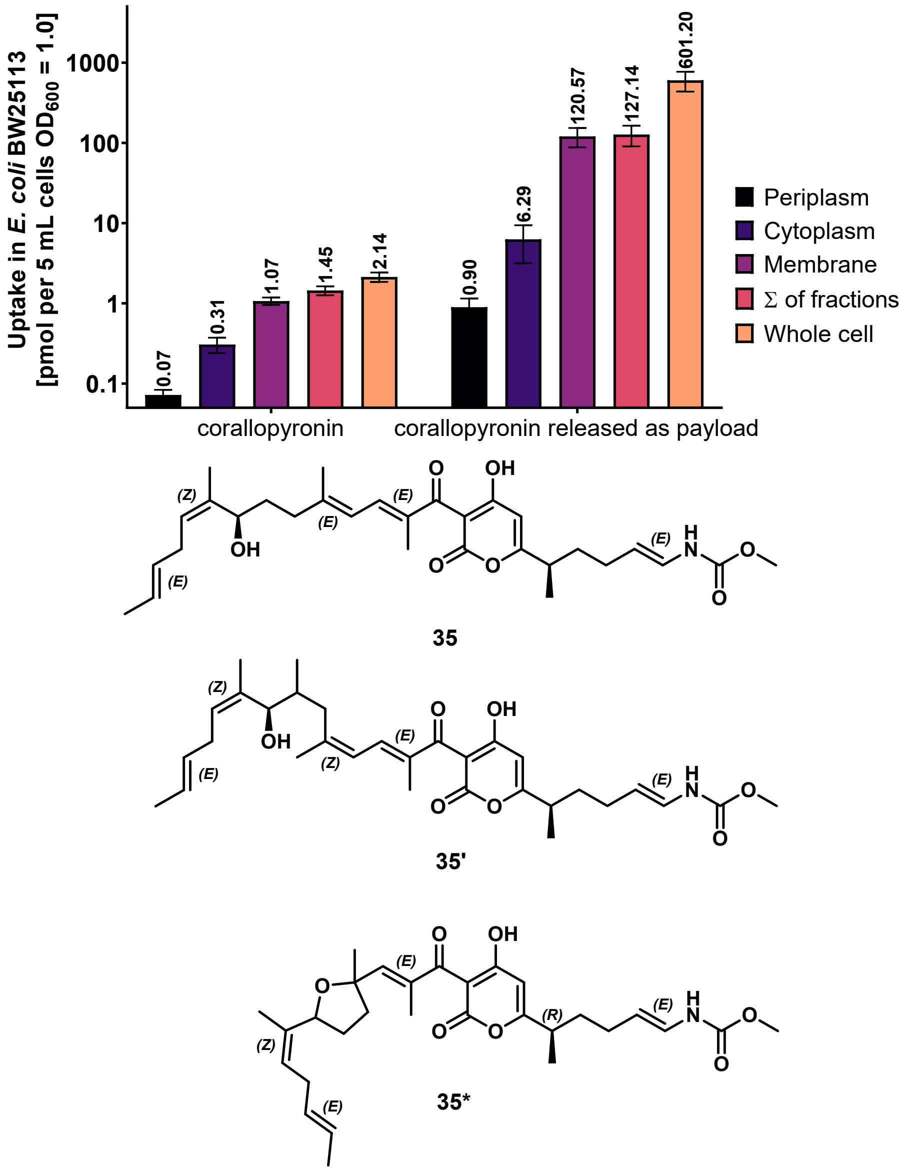

Figure S45. A) Uptake or CorA (**35**) and payload released from **36** into MDR resistant *E. coli* DSM1116. 1 µM compound was incubated with 5 ml cells OD600= 1.0 as described above. The uptake in the different compartments – periplasm (black), cytoplasm (violet), membrane (purple) – and whole cells (amber) is depicted as bar graphs. The sum of the amounts in the fractions periplasm, cytoplasm and membrane is shown in pink. The accumulation was monitored using the MRMs for **35**. Note the log scale of the graph. B) Structures of CorA (35), CorA' (35') and CorC (35*).[30]

NMR spectra

Compound **40**

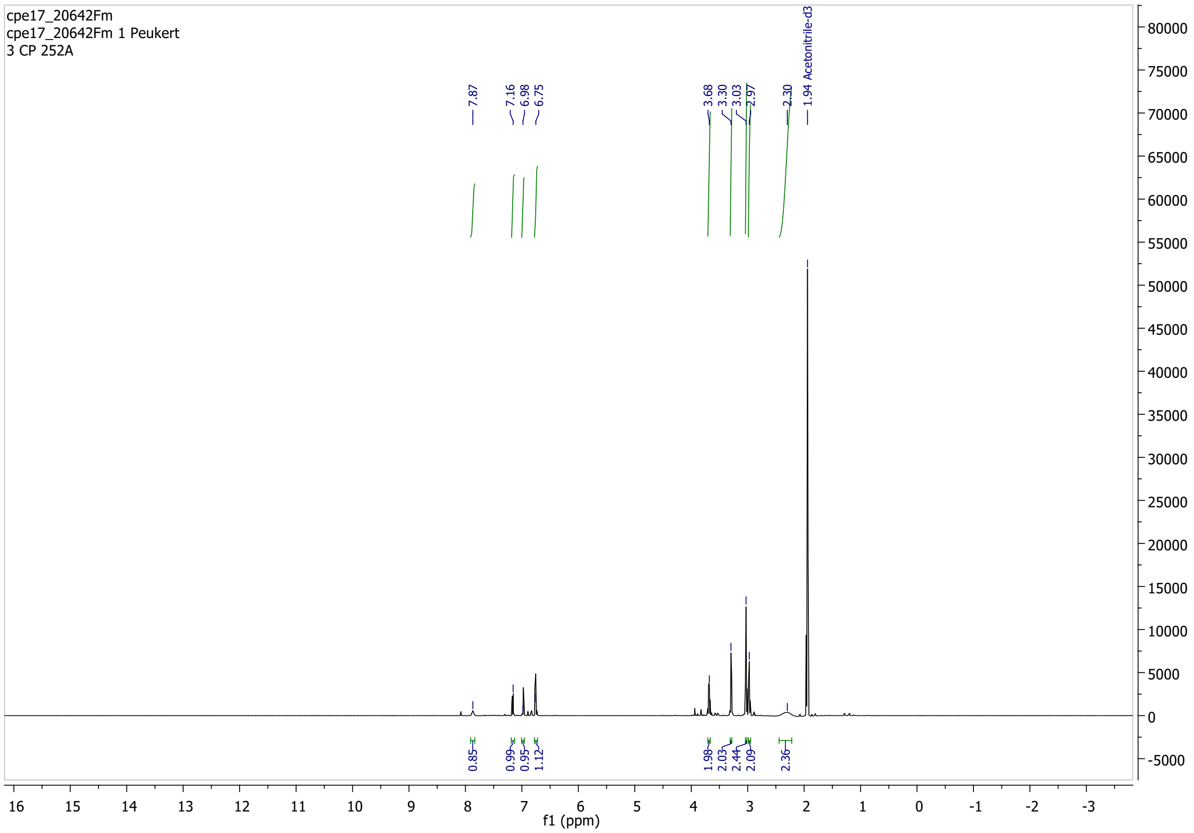

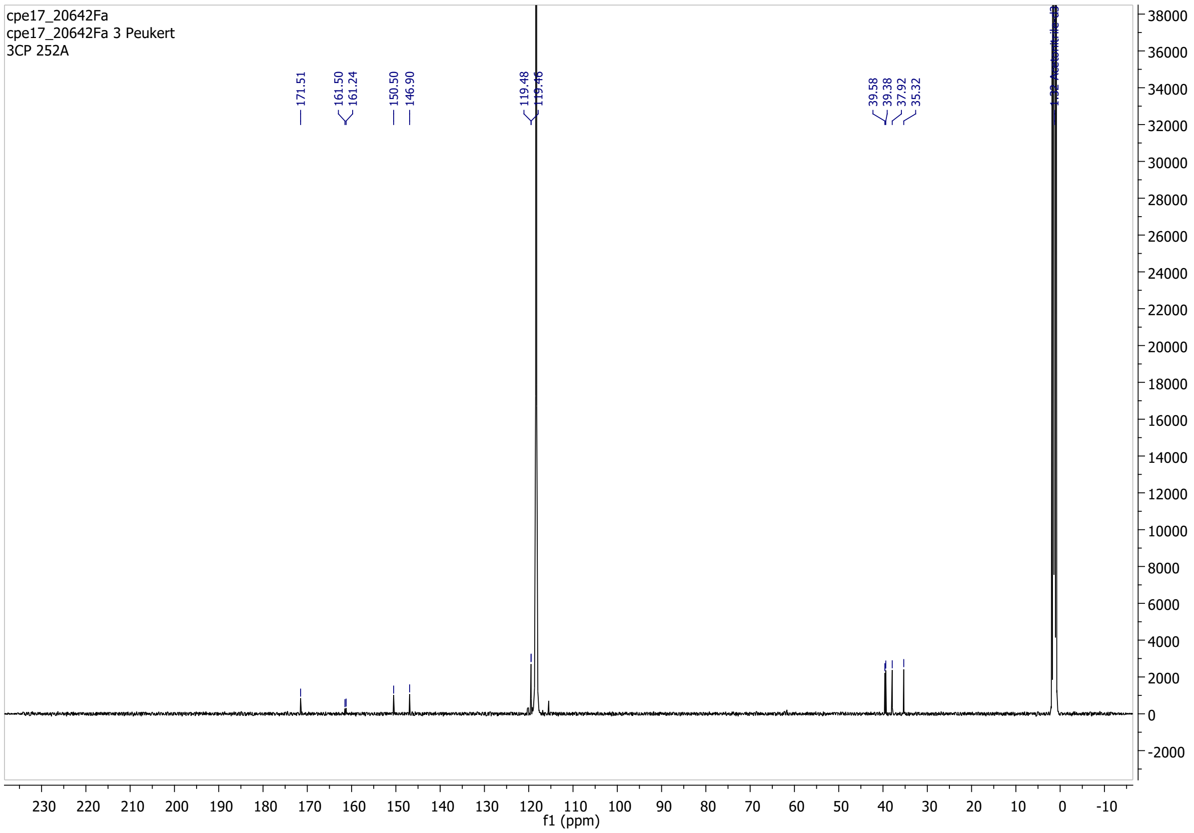

Compound **41**

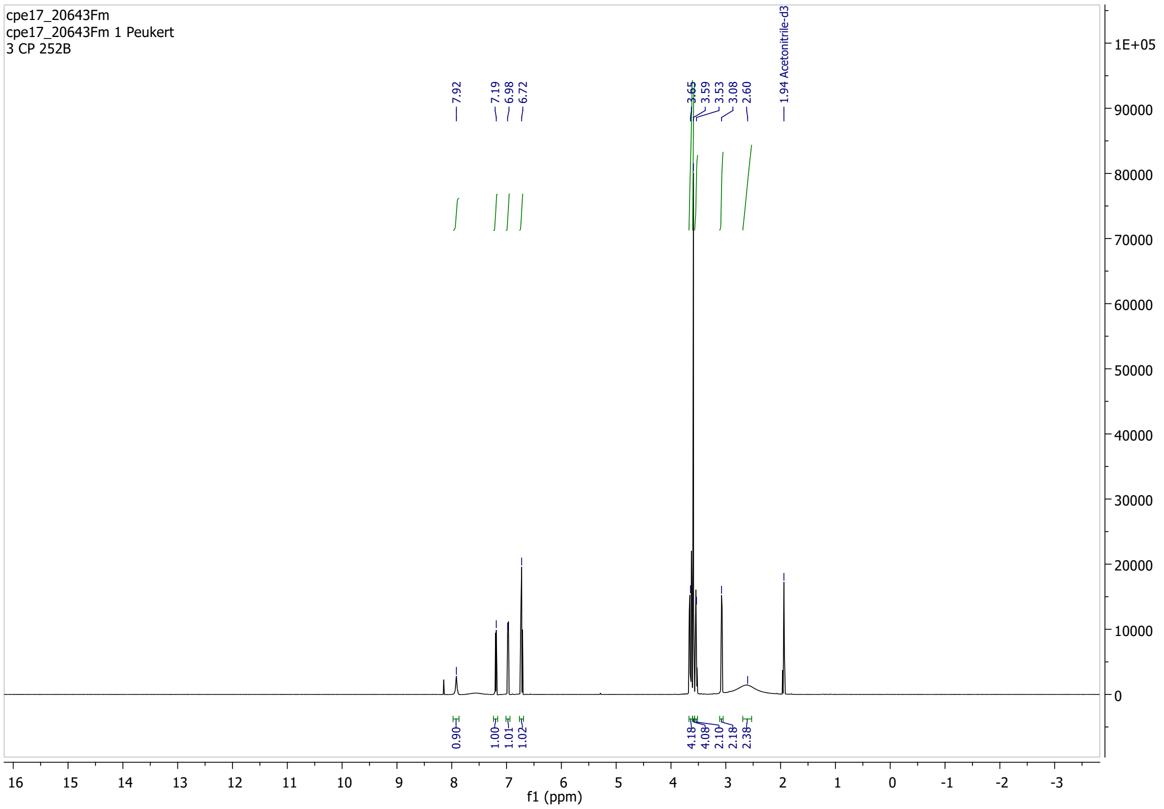
cpe17_20643Fm
cpe17_20643Fm 1 Peukert
3 CP 252B
7.92
7.19
6.98
6.72
3.65
3.59
3.53
3.08
2.60
1.94 Acetonitrile-d3
0.90
1.00
1.01
1.02
4.18
4.08
2.10
2.18
2.38
f1 (ppm)

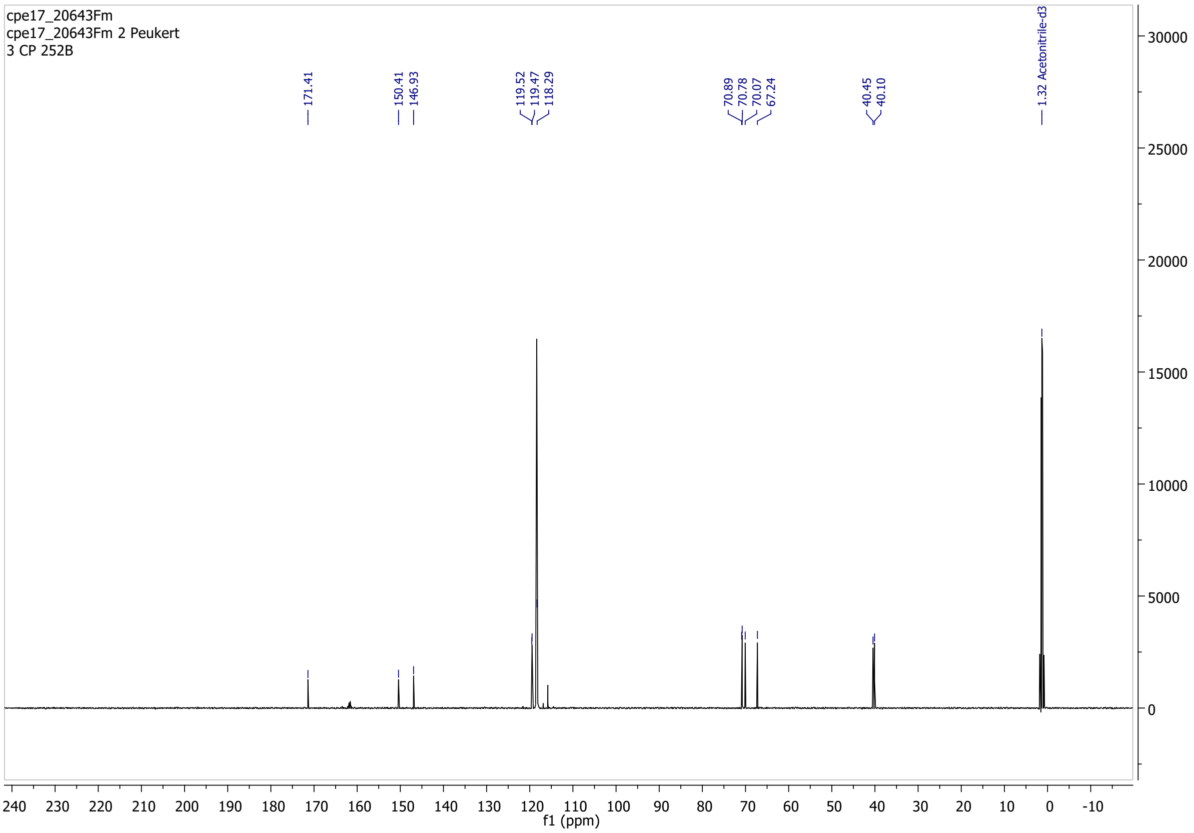
cpe17_20643Fm
cpe17_20643Fm 2 Peukert
3 CP 252B
171.41
150.41
146.93
119.52
119.47
118.29
70.89
70.78
70.07
67.24
40.45
40.10
1.32 Acetonitrile-d3
f1 (ppm)

Compound **7**

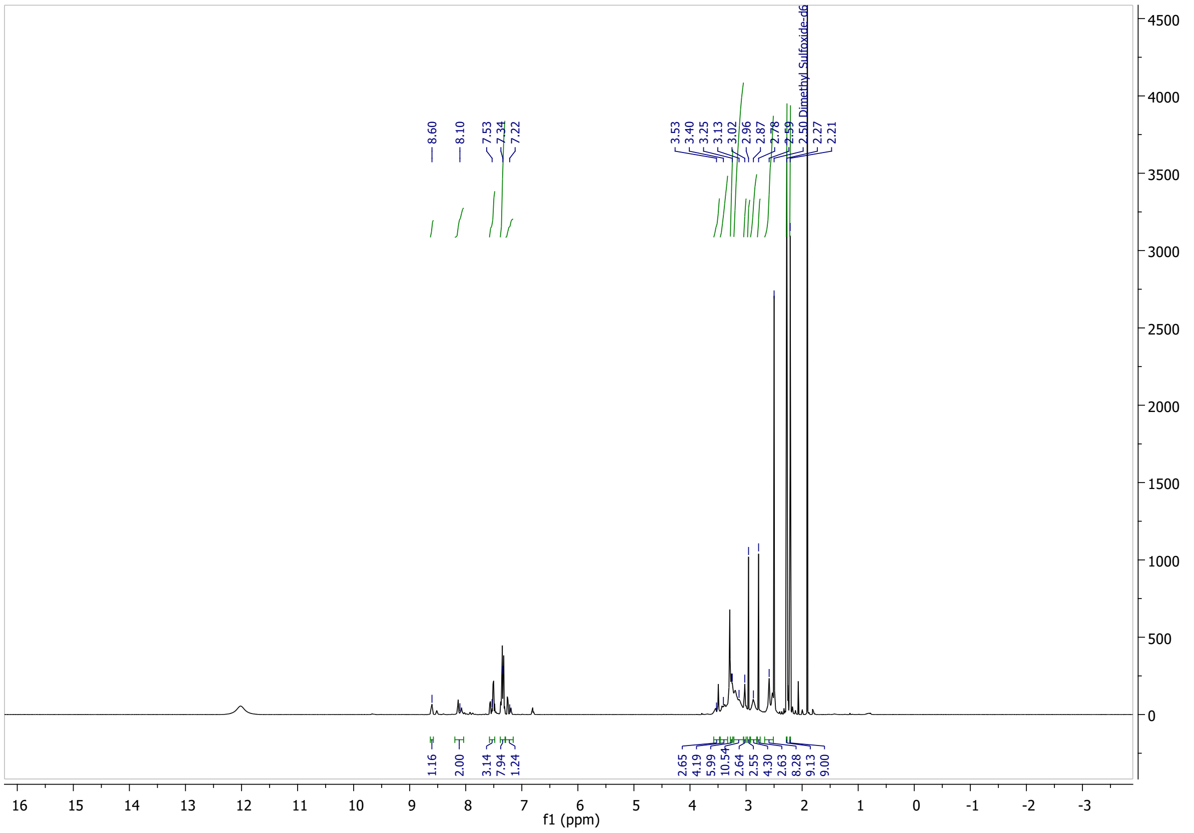

8.60
8.10
7.53
7.34
7.22
3.53
3.40
3.25
3.13
3.02
2.96
2.87
2.78
2.59
2.50 Dimethyl Sulfoxide-d6
2.27
2.21
1.16
2.00
3.14
7.94
1.24
2.65
4.19
5.99
10.54
2.64
2.55
4.30
2.63
8.28
9.13
9.00
f1 (ppm)

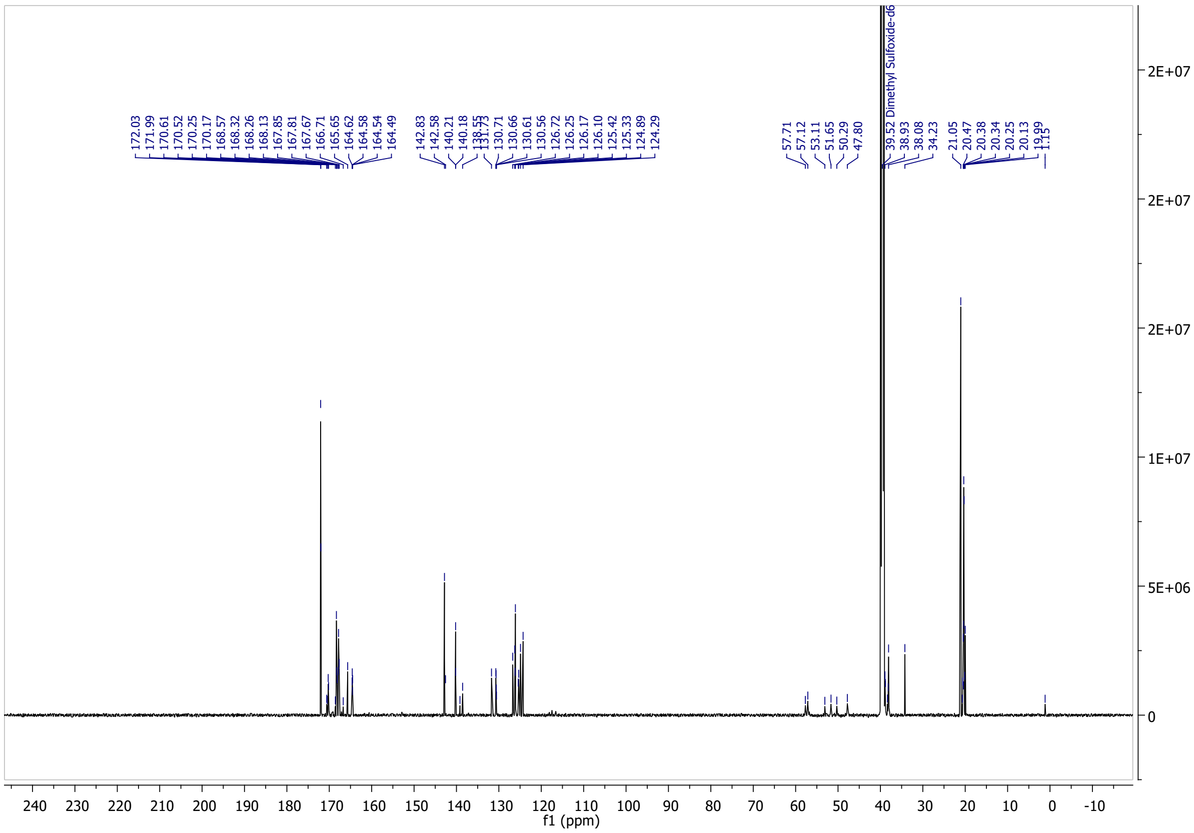

172.03
171.99
170.61
170.52
170.25
170.17
168.57
168.32
168.26
168.13
167.85
167.81
167.67
166.71
165.65
164.62
164.58
164.54
164.49
142.83
142.58
140.21
140.18
138.55
131.73
130.71
130.66
130.61
130.56
126.72
126.25
126.17
126.10
125.42
125.33
124.89
124.29
57.71
57.12
53.11
51.65
50.29
47.80
39.52 Dimethyl Sulfoxide-d6
38.93
38.08
34.23
21.05
20.47
20.38
20.34
20.25
20.13
19.99
1.15
f1 (ppm)

Compound **43**

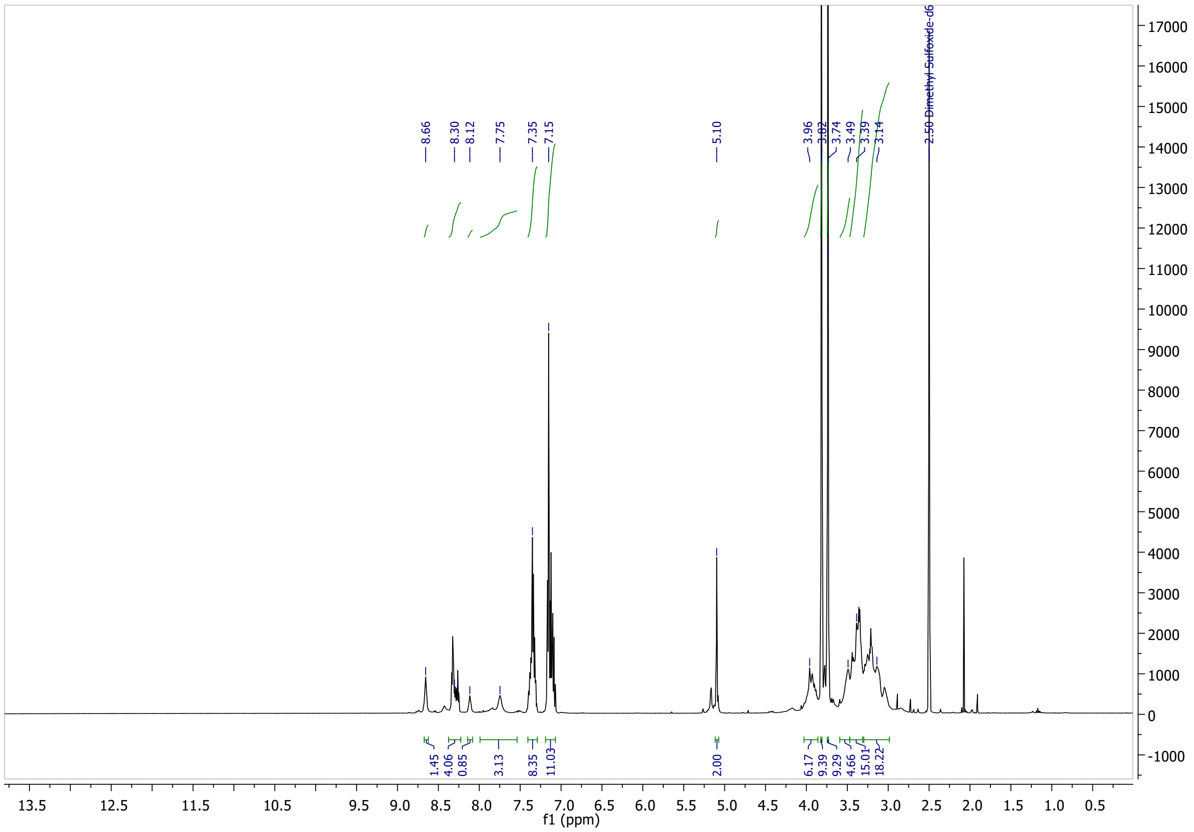

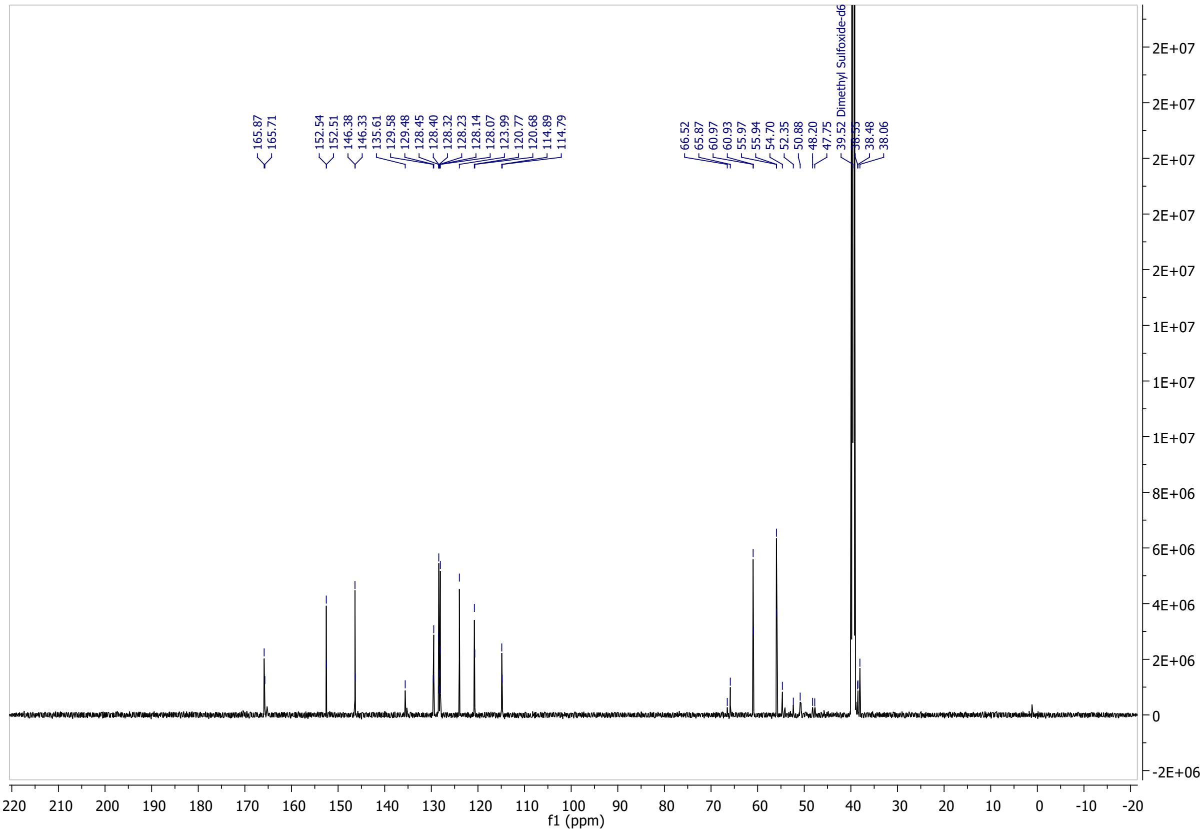

Compound **44**

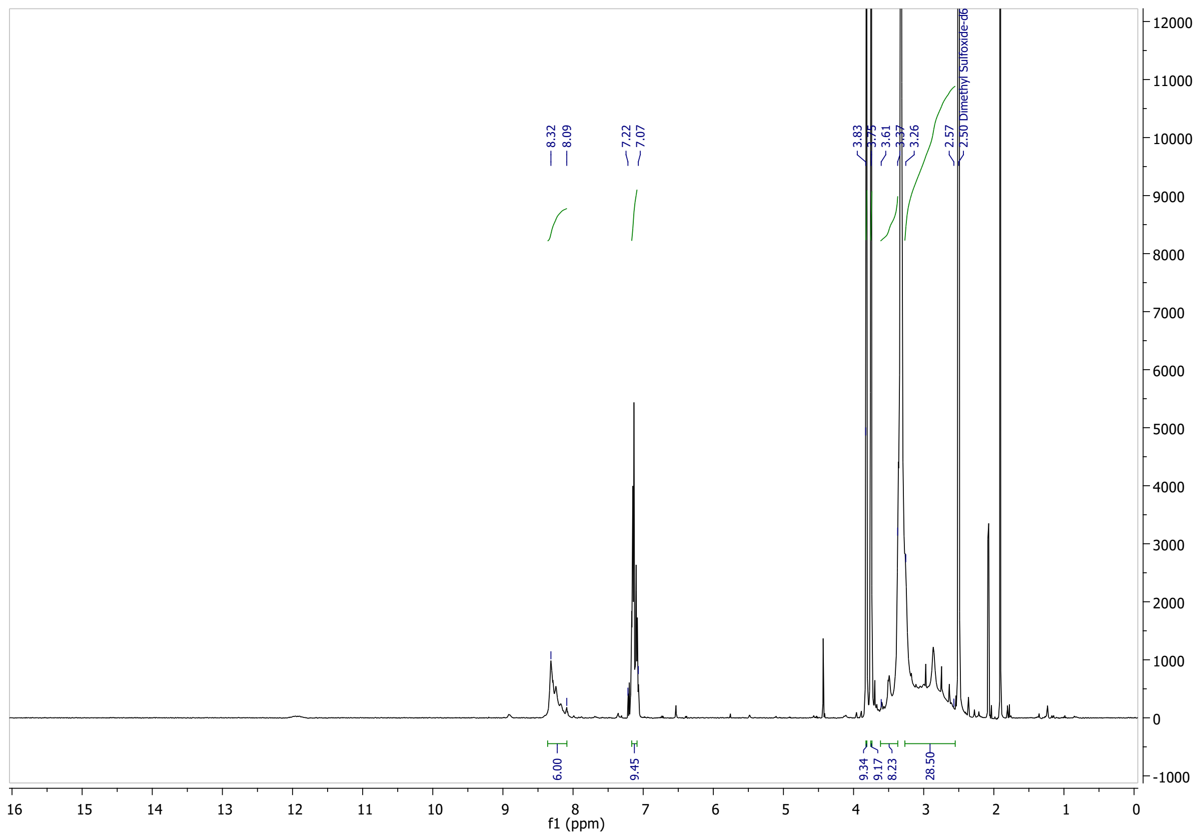
8.32
8.09
7.22
7.07
3.83
3.75
3.61
3.37
3.26
2.57
2.50 Dimethyl Sulfoxide-d6
6.00
9.45
9.34
9.17
8.23
28.50
f1 (ppm)

Compound **46**

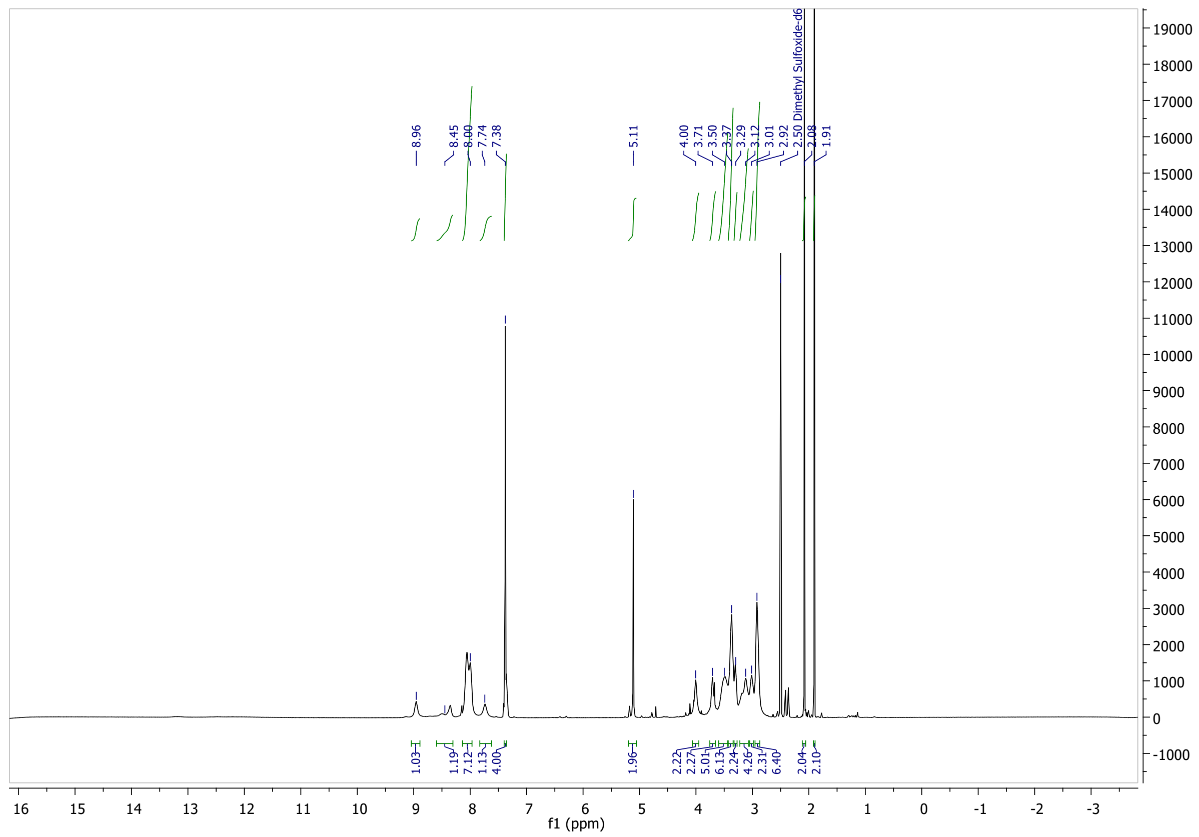
8.96
8.45
8.00
7.74
7.38
5.11
4.00
3.71
3.50
3.37
3.29
3.12
3.01
2.92
2.50 Dimethyl Sulfoxide-d6
2.08
1.91
1.03
1.19
7.12
1.13
4.00
1.96
2.22
2.27
5.01
6.13
2.24
4.26
2.31
6.40
2.04
2.10
f1 (ppm)

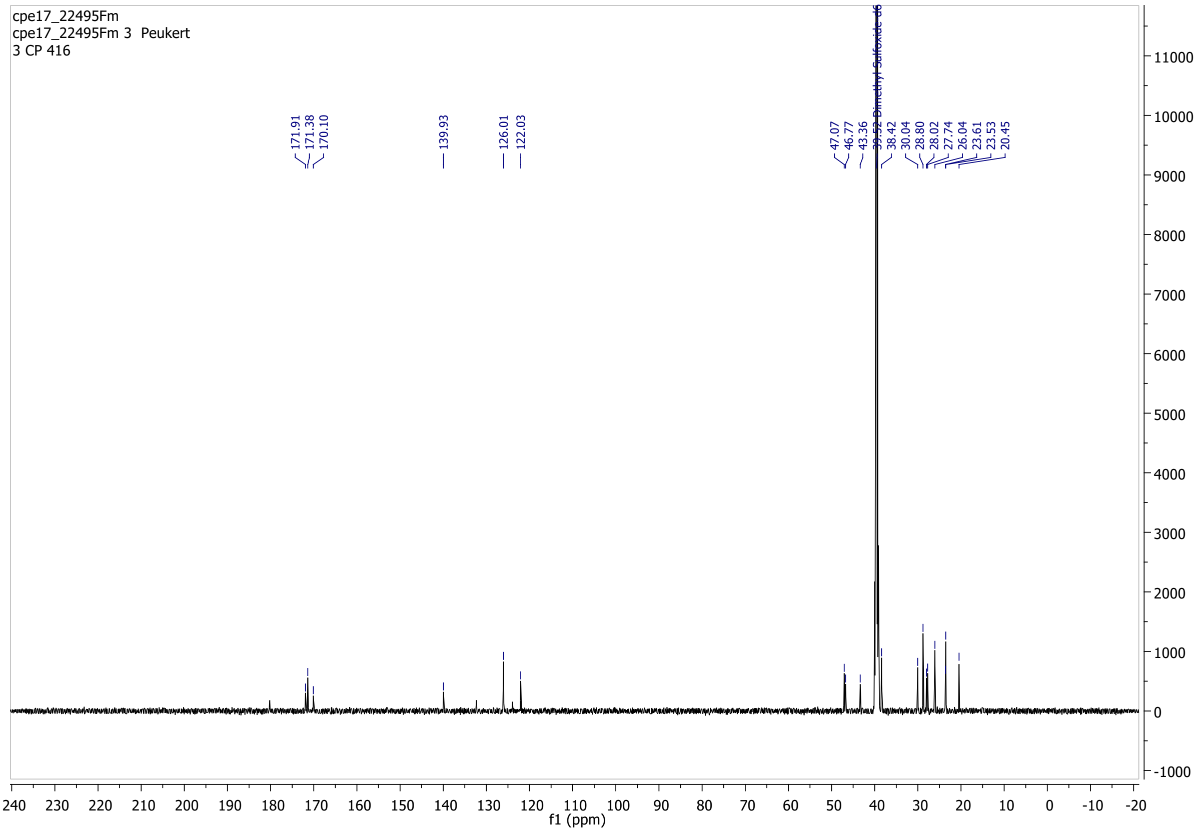
cpe17_22495Fm
cpe17_22495Fm 3 Peukert
3 CP 416
171.91
171.38
170.10
139.93
126.01
122.03
47.07
46.77
43.36
39.52 Dimethyl Sulfoxide-d6
38.42
30.04
28.80
28.02
27.74
26.04
23.61
23.53
20.45
f1 (ppm)

Compound **47**

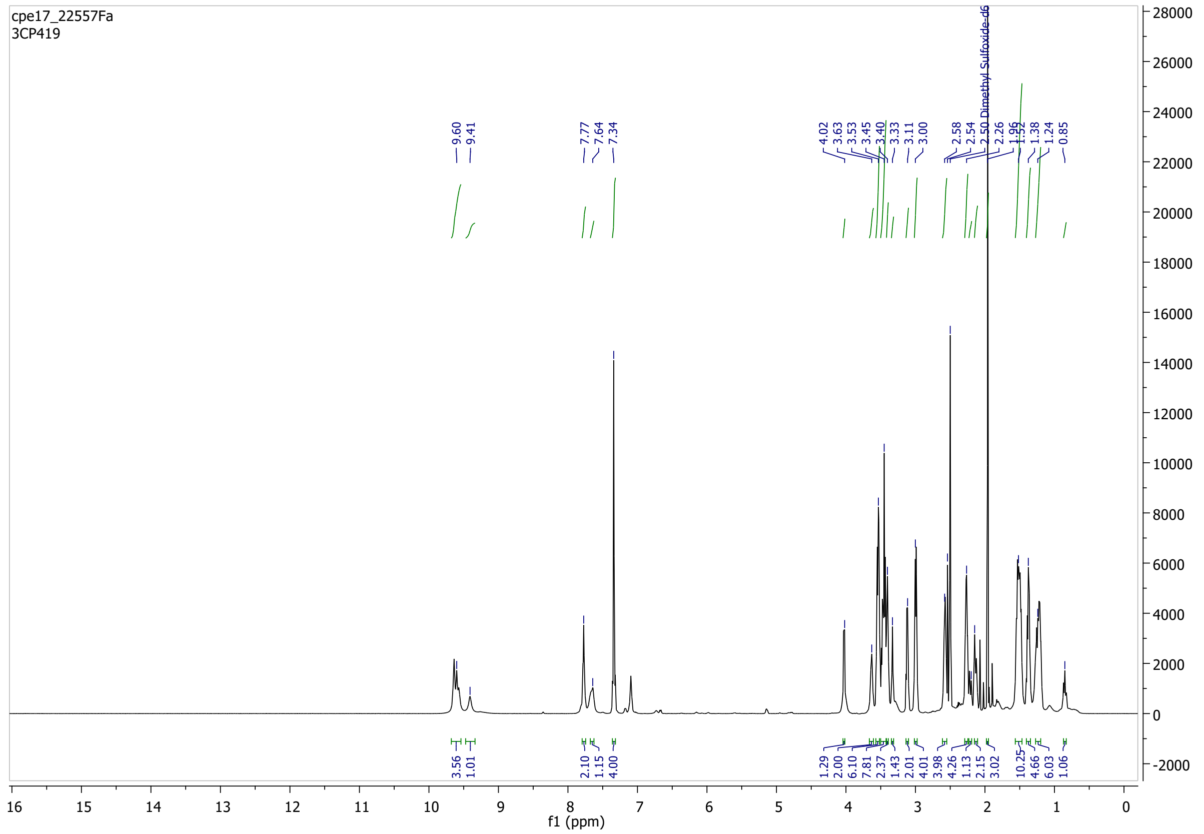
cpe17_22557Fa
3CP419
9.60
9.41
7.77
7.64
7.34
4.02
3.63
3.53
3.45
3.40
3.33
3.11
3.00
2.58
2.54
2.50 Dimethyl Sulfoxide-d6
2.26
1.96
1.52
1.38
1.24
0.85
3.56
1.01
2.10
1.15
4.00
1.29
2.00
6.10
7.81
2.37
1.43
2.01
4.01
3.98
4.26
1.13
2.15
3.02
10.25
4.66
6.03
1.06
f1 (ppm)

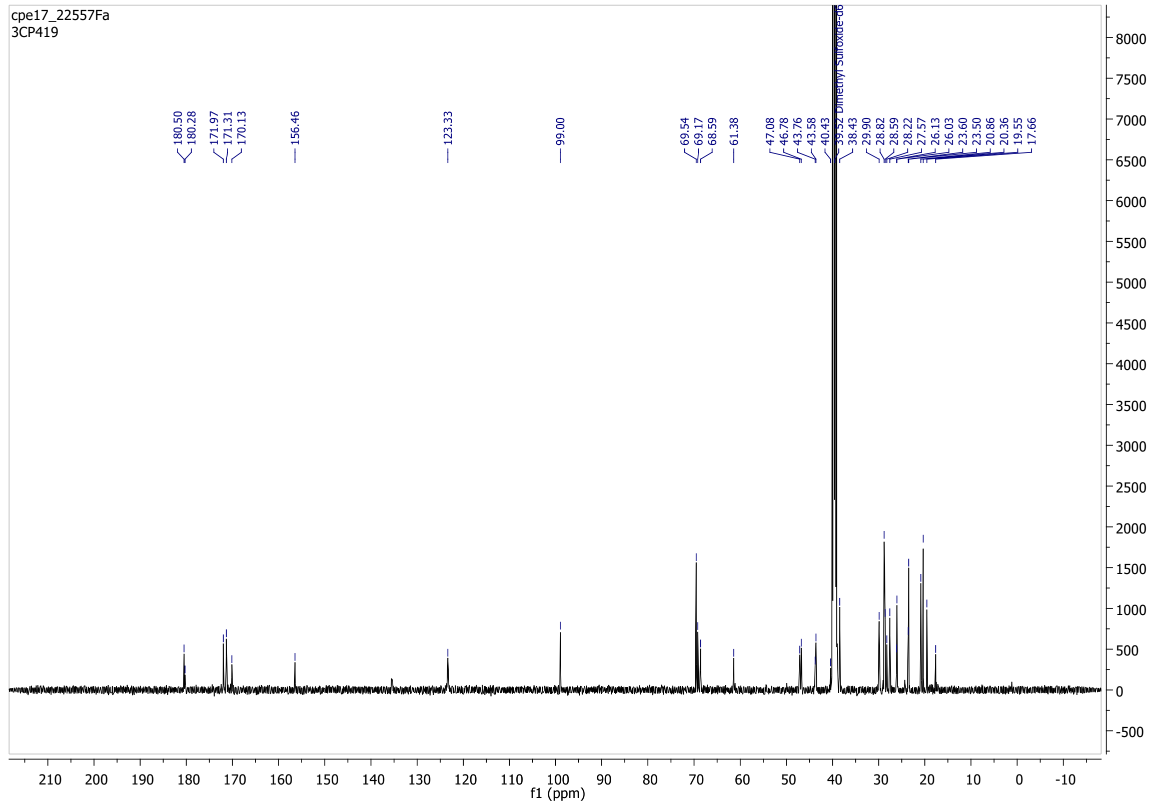
cpe17_22557Fa
3CP419
180.50
180.28
171.97
171.31
170.13
156.46
123.33
99.00
69.54
69.17
68.59
61.38
47.08
46.78
43.76
43.58
40.43
39.52 Dimethyl Sulfoxide-d6
38.43
29.90
28.82
28.59
28.22
27.57
26.13
26.03
23.60
23.50
20.86
20.36
19.55
17.66
f1 (ppm)

Compound **47b**

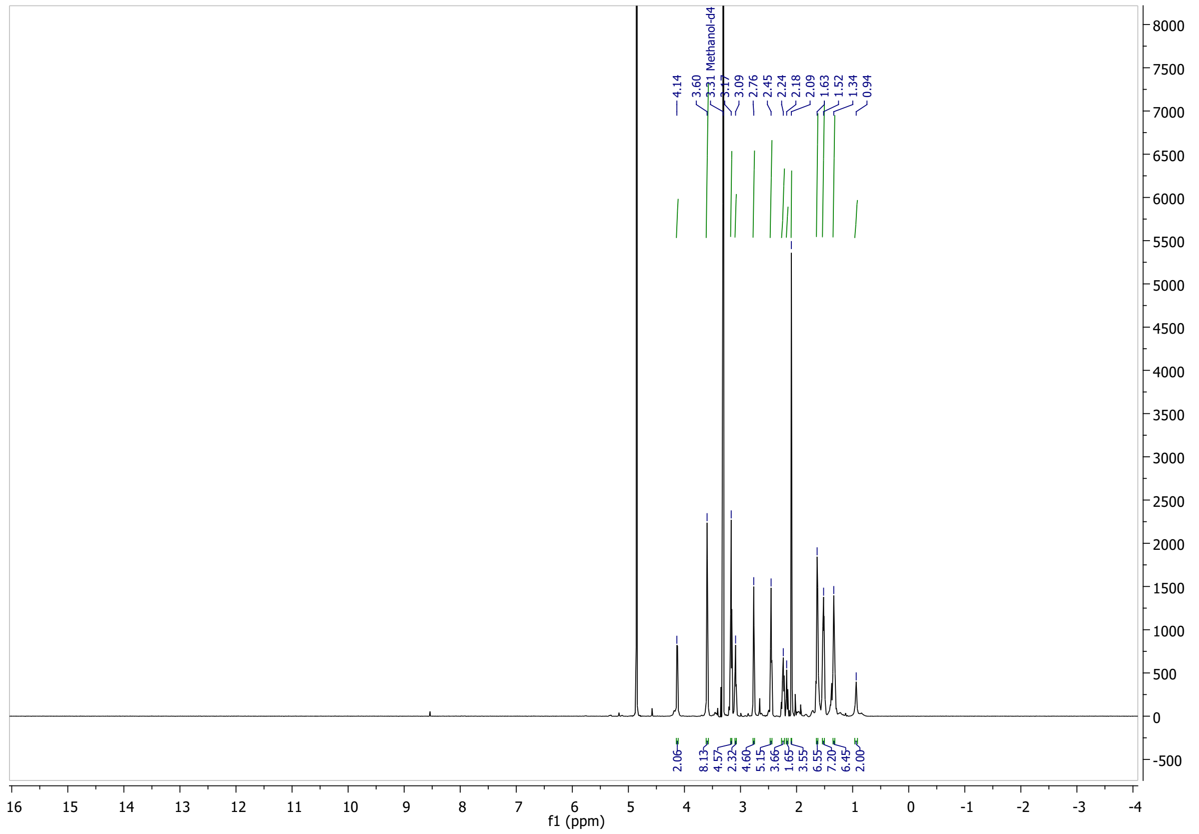

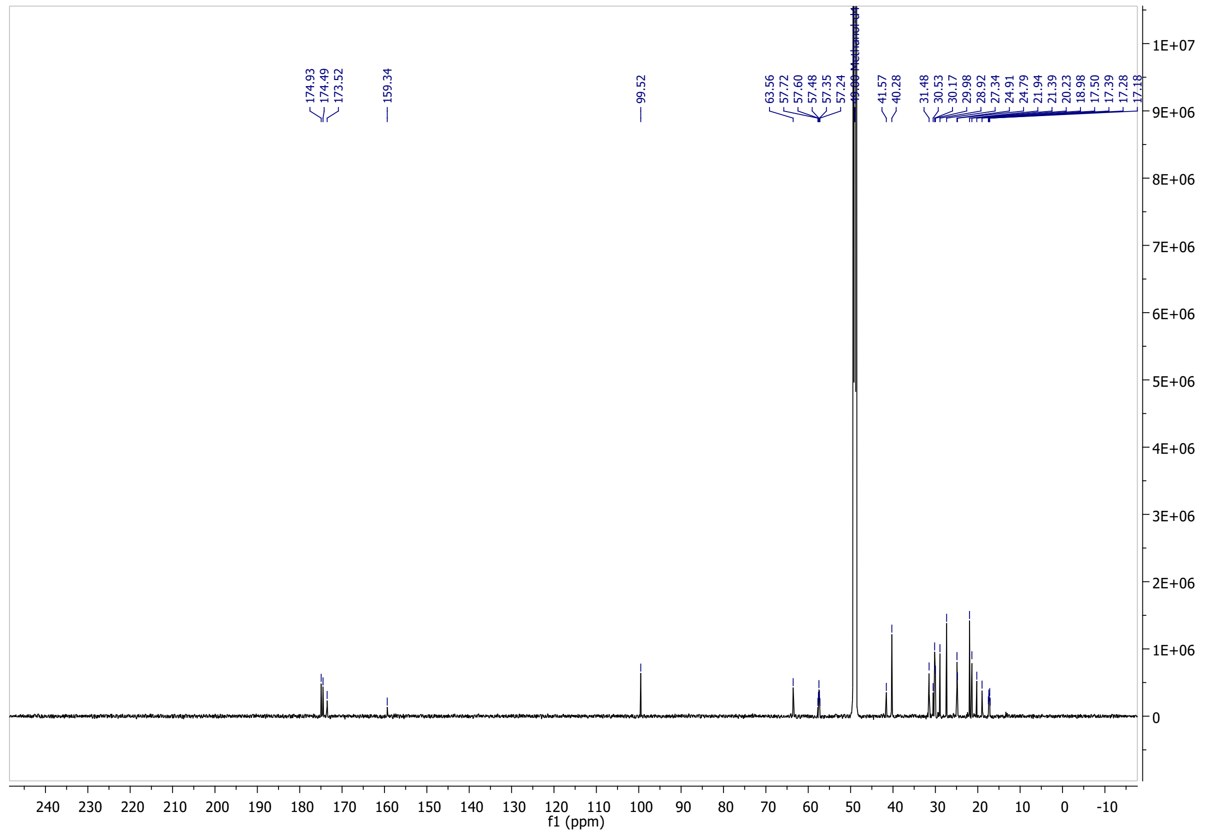

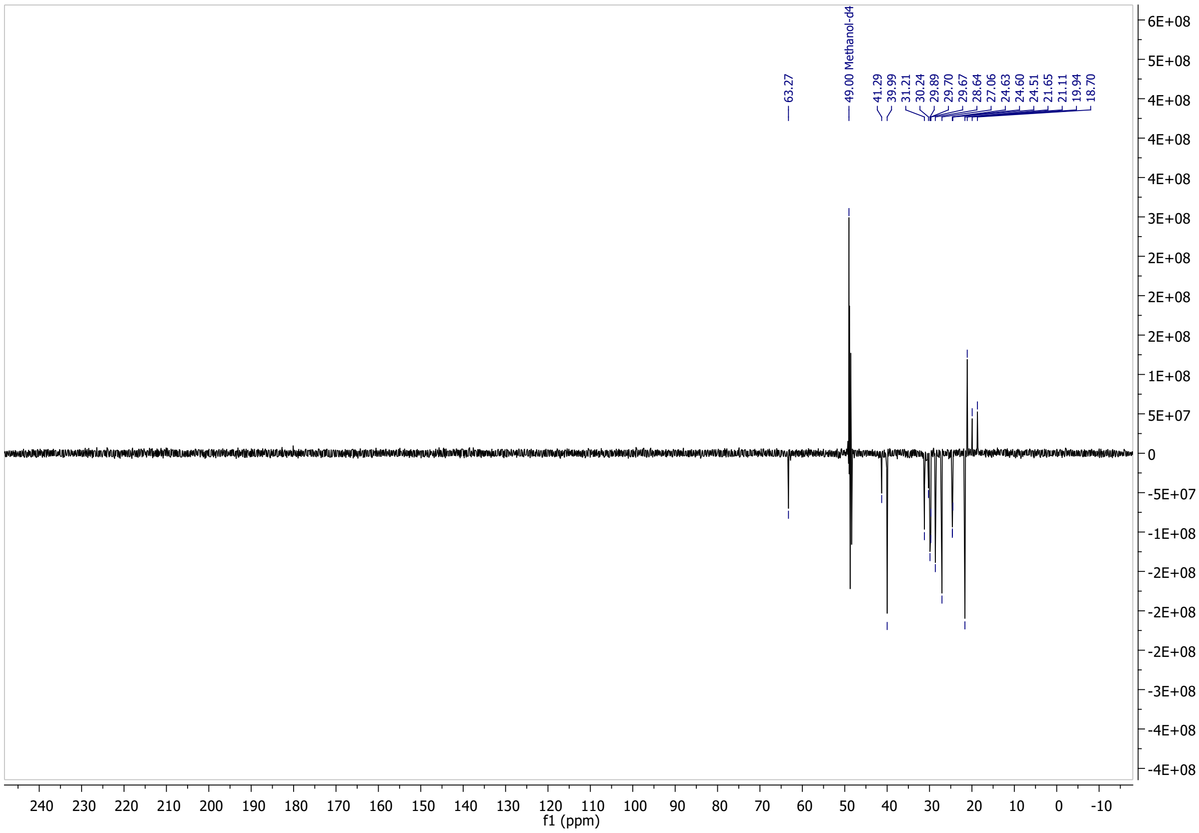

63.27
49.00 Methanol-d4
41.29
39.99
31.21
30.24
29.89
29.70
29.67
28.64
27.06
24.63
24.60
24.51
21.65
21.11
19.94
18.70
f1 (ppm)

Compound **48**

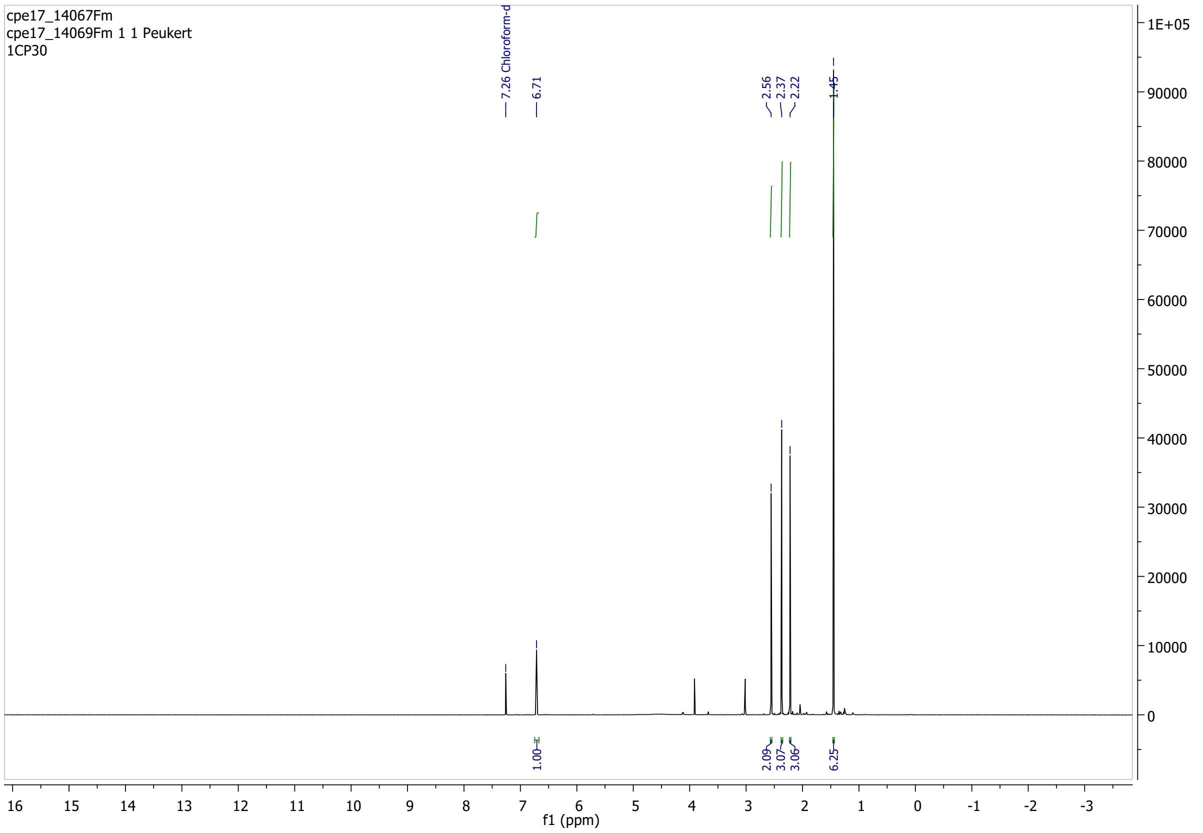

cpe17_14067Fm
cpe17_14069Fm 1 1 Peukert
1CP30
7.26 Chloroform-d
6.71
2.56
2.37
2.22
1.45
1.00
2.09
3.07
3.06
6.25
f1 (ppm)

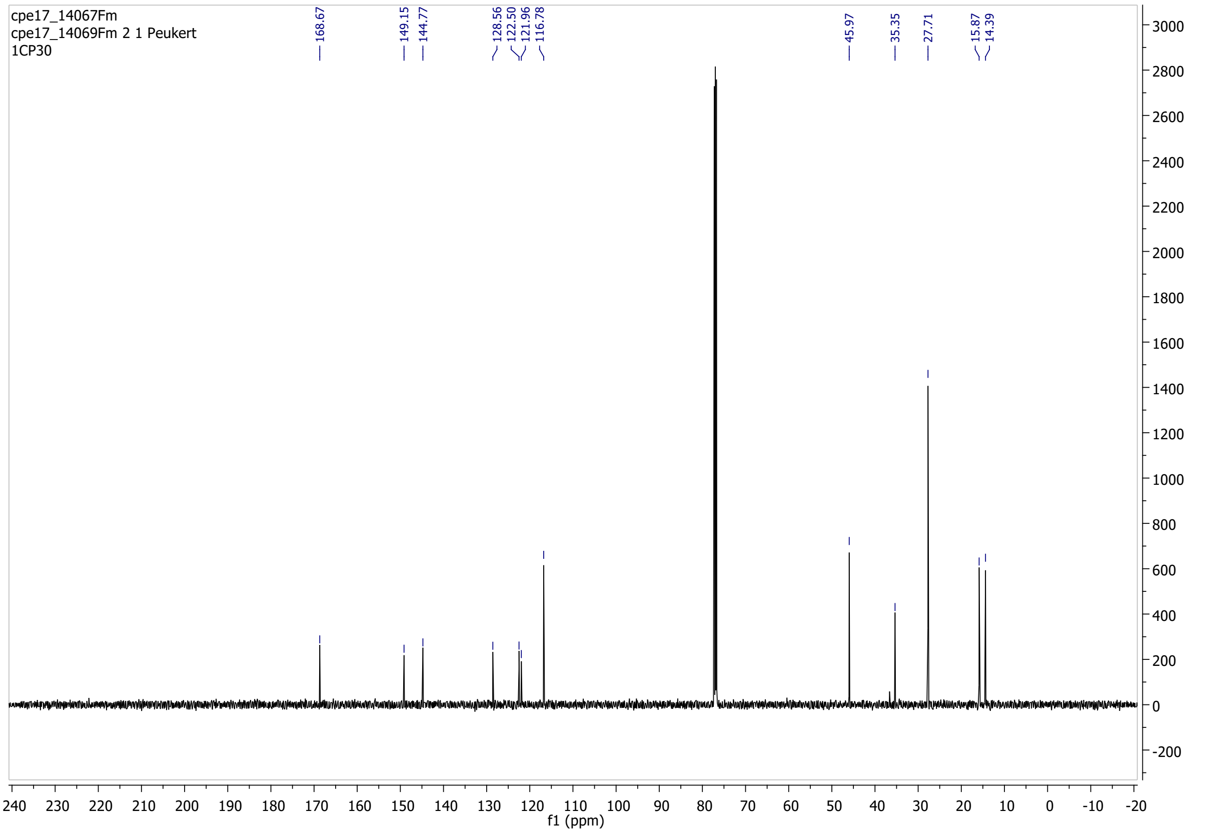

cpe17_14067Fm
cpe17_14069Fm 2 1 Peukert
1CP30
168.67
149.15
144.77
128.56
122.50
121.96
116.78
45.97
35.35
27.71
15.87
14.39
f1 (ppm)

Compound **49**

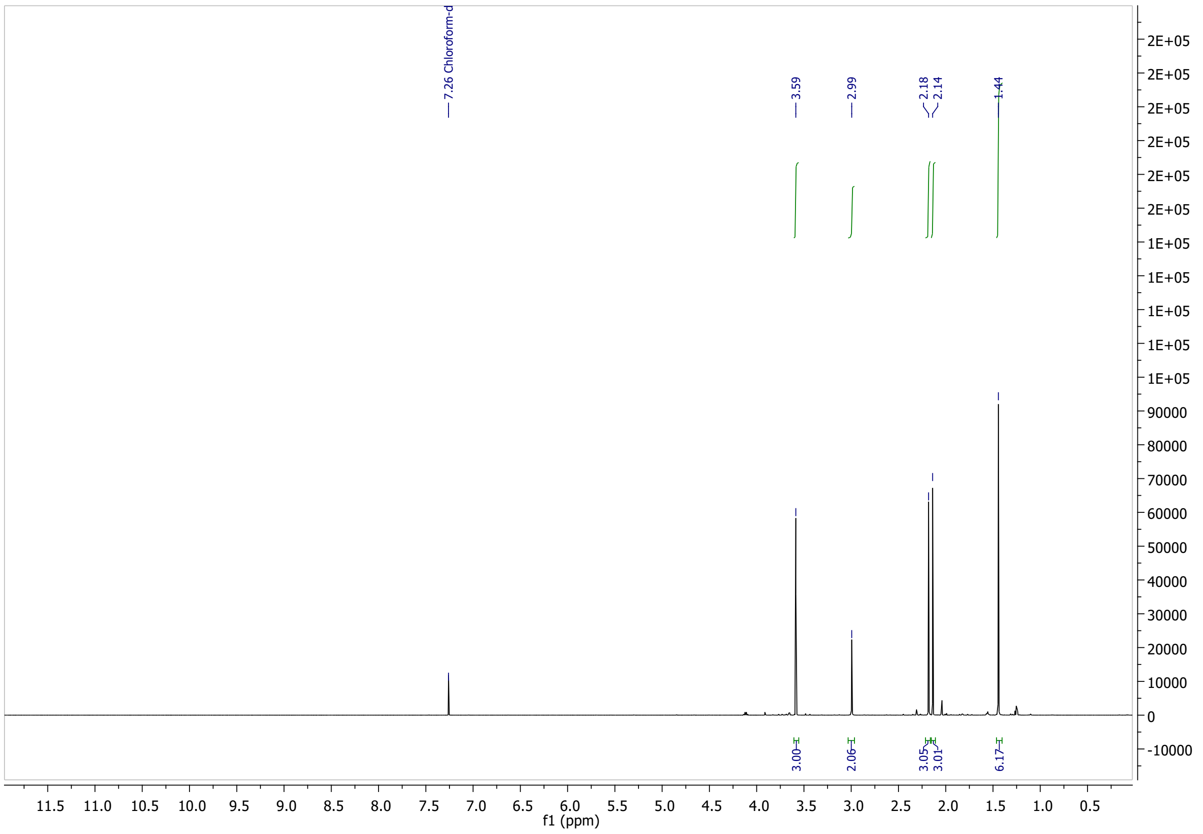

7.26 Chloroform-d
3.59
2.99
2.18
2.14
1.44
3.00
2.06
3.05
3.01
6.17
f1 (ppm)

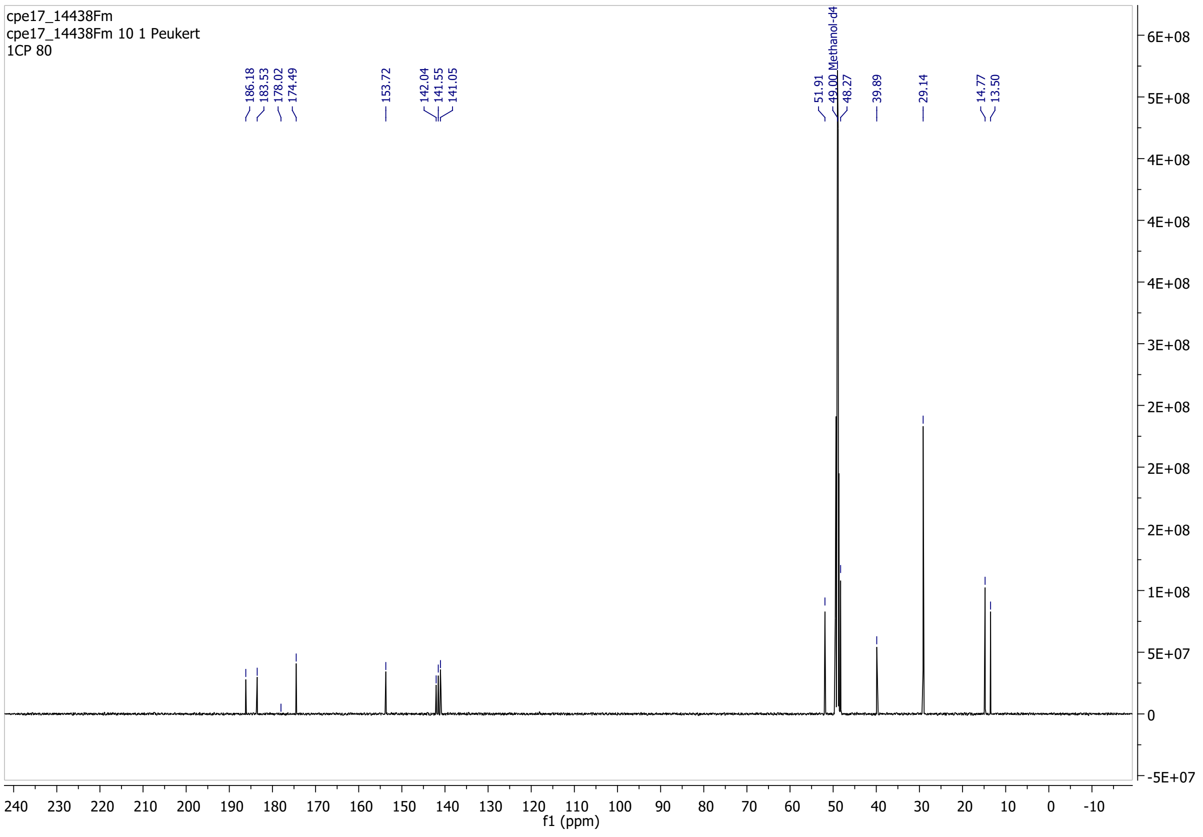

cpe17_14438Fm
cpe17_14438Fm 10 1 Peukert
1CP 80
186.18
183.53
178.02
174.49
153.72
142.04
141.55
141.05
51.91
49.00 Methanol-d4
48.27
39.89
29.14
14.77
13.50
f1 (ppm)

Compound **50**

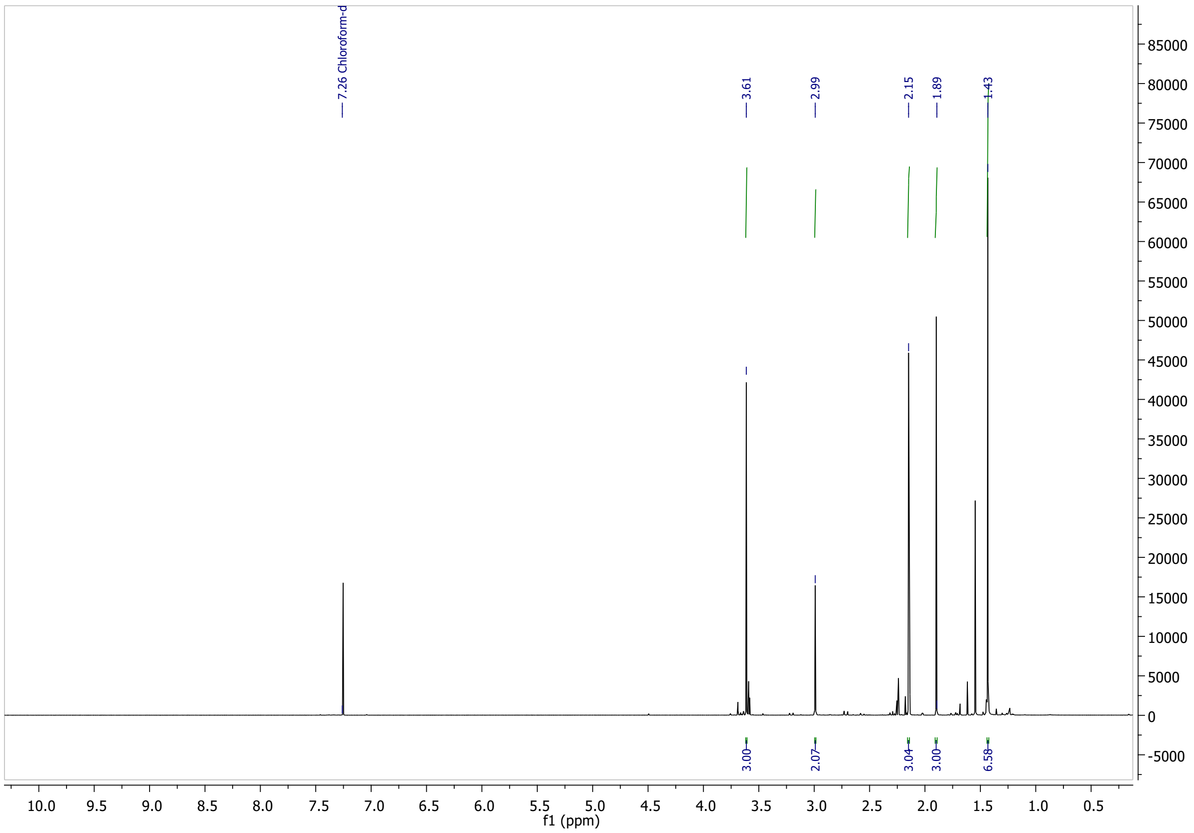

7.26 Chloroform-d
3.61
2.99
2.15
1.89
1.43
3.00
2.07
3.04
3.00
6.58
85000
80000
75000
70000
65000
60000
55000
50000
45000
40000
35000
30000
25000
20000
15000
10000
5000
0
-5000
10.0 9.5 9.0 8.5 8.0 7.5 7.0 6.5 6.0 5.5 5.0 4.5 4.0 3.5 3.0 2.5 2.0 1.5 1.0 0.5
f1 (ppm)

Compound **51**

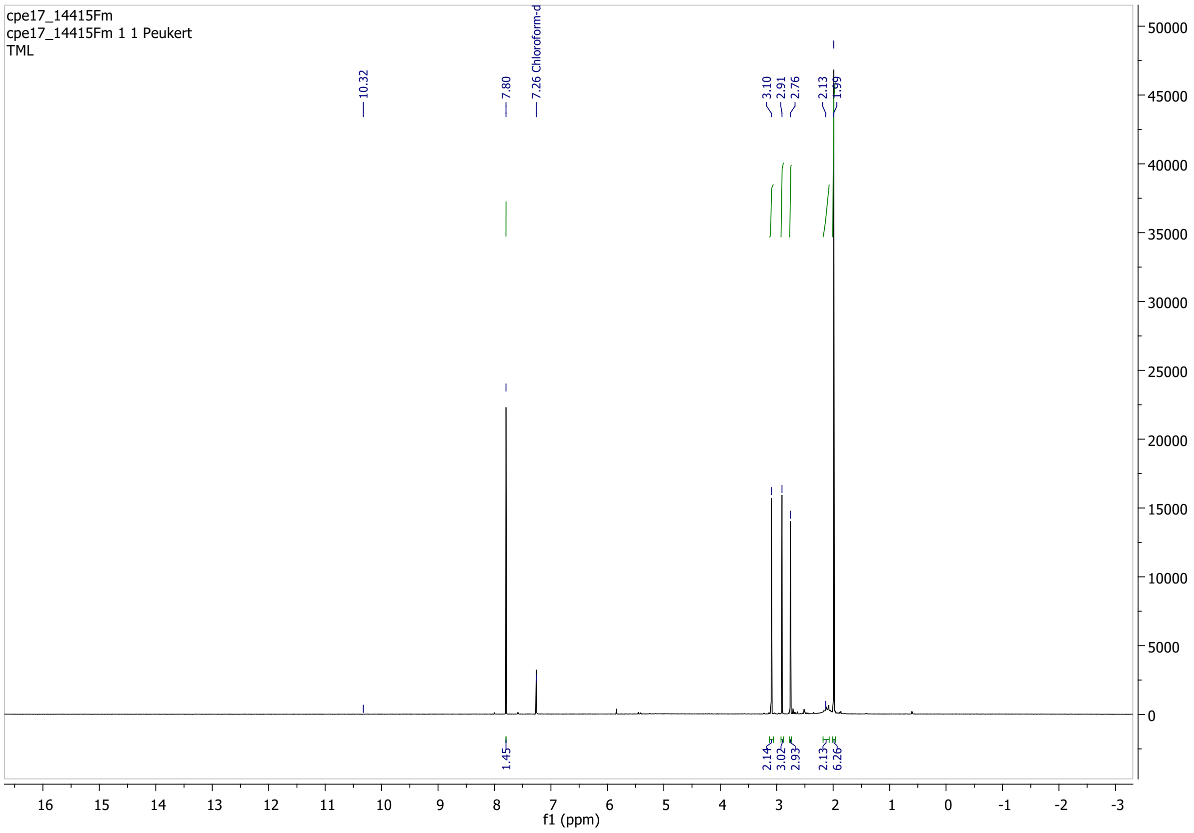
cpe17_14415Fm
cpe17_14415Fm 1 1 Peukert
TML
10.32
7.80
7.26 Chloroform-d
3.10
2.91
2.76
2.13
1.99
1.45
2.14
3.02
2.93
2.13
6.26
f1 (ppm)

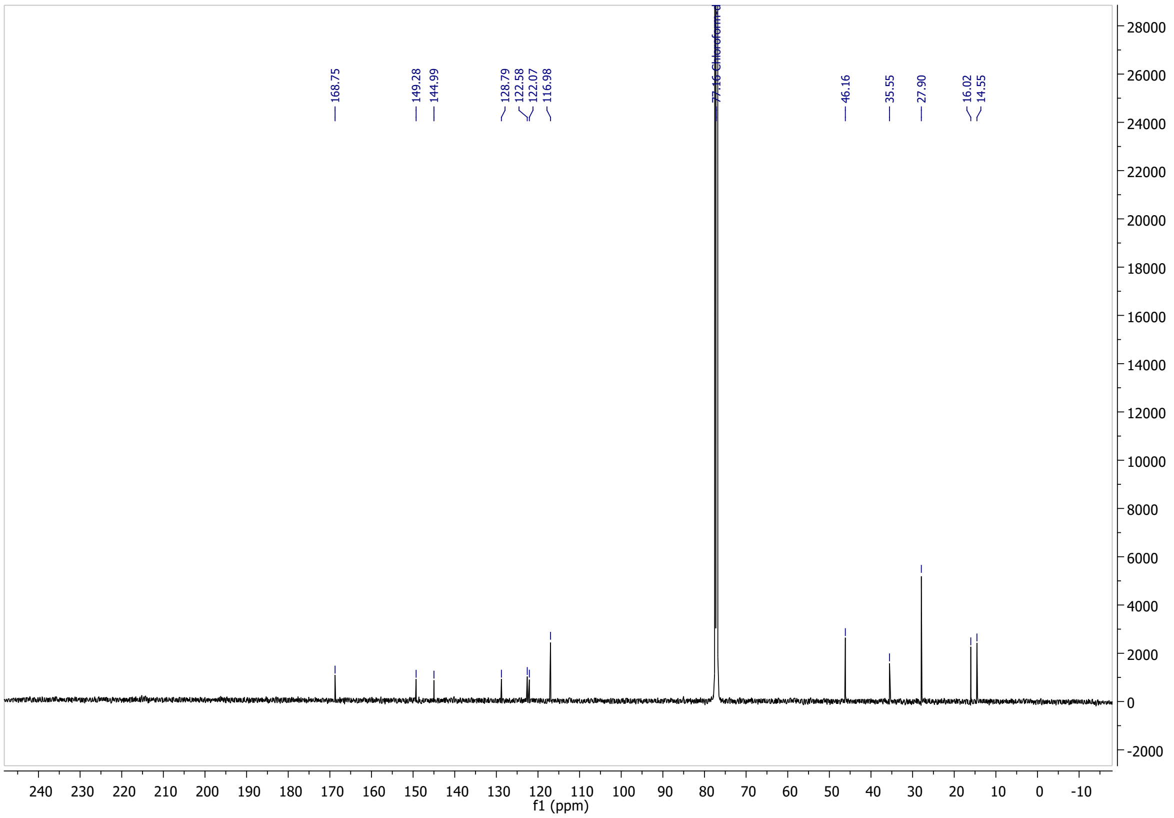
168.75
149.28
144.99
128.79
122.58
122.07
116.98
77.16 Chloroform-d
46.16
35.55
27.90
16.02
14.55
f1 (ppm)

Compound **52**

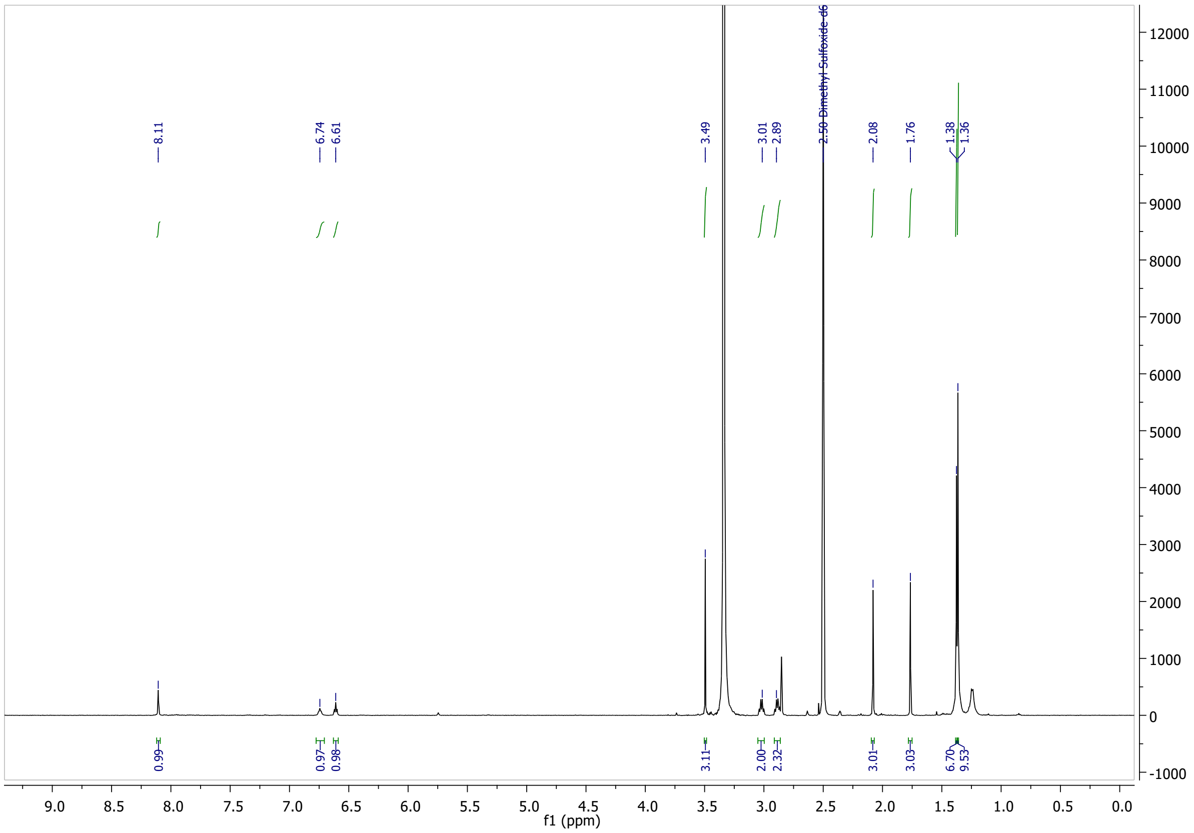
8.11
6.74
6.61
3.49
3.01
2.89
2.50 Dimethyl Sulfoxide-d6
2.08
1.76
1.38
1.36
0.99
0.97
0.98
3.11
2.00
2.32
3.01
3.03
6.70
9.53
f1 (ppm)

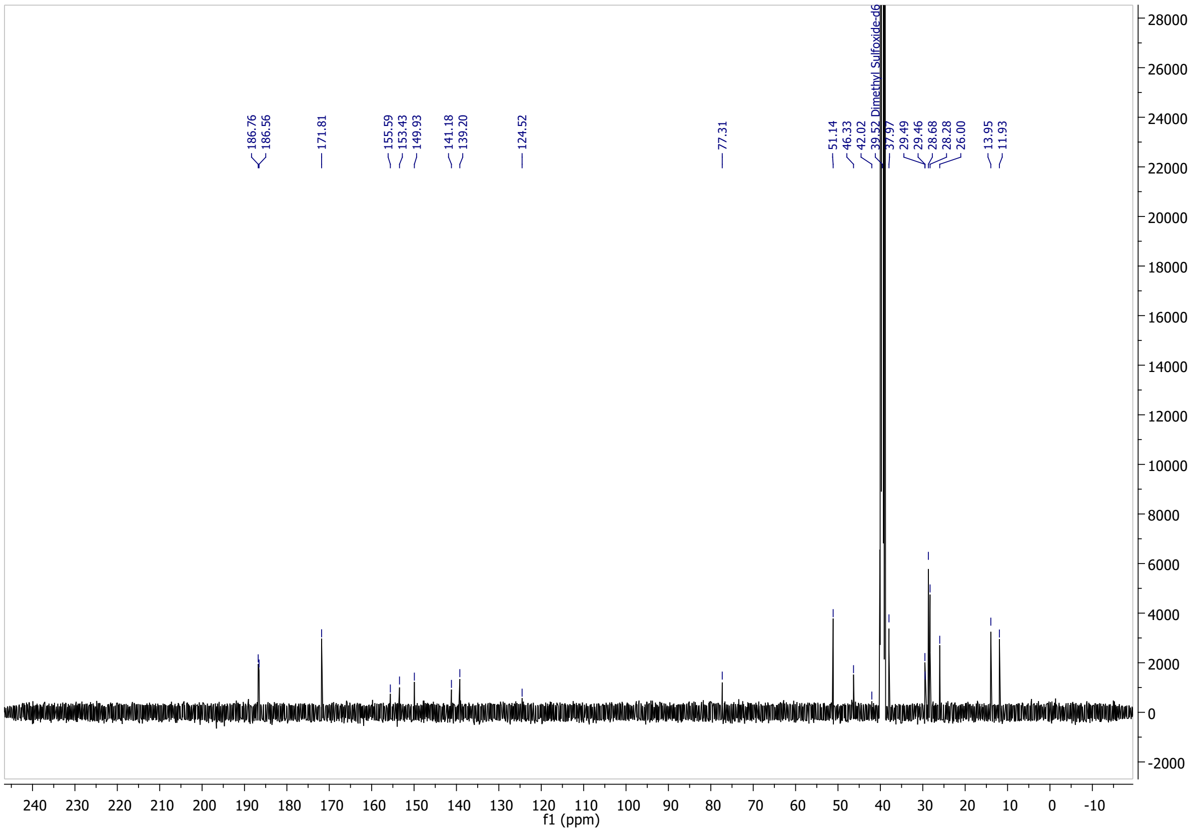
186.76
186.56
171.81
155.59
153.43
149.93
141.18
139.20
124.52
77.31
51.14
46.33
42.02
39.52 Dimethyl Sulfoxide-d6
37.97
29.49
29.46
28.68
28.28
26.00
13.95
11.93
f1 (ppm)

Compound **53**

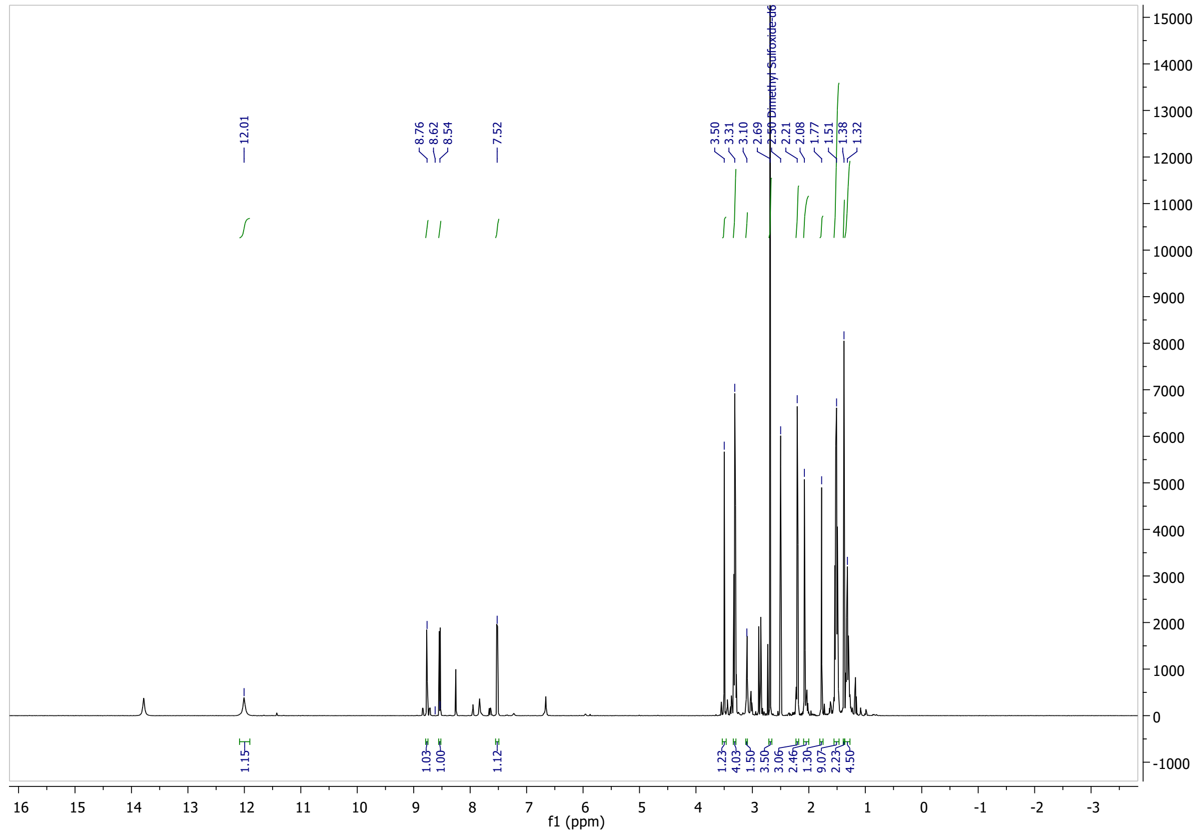

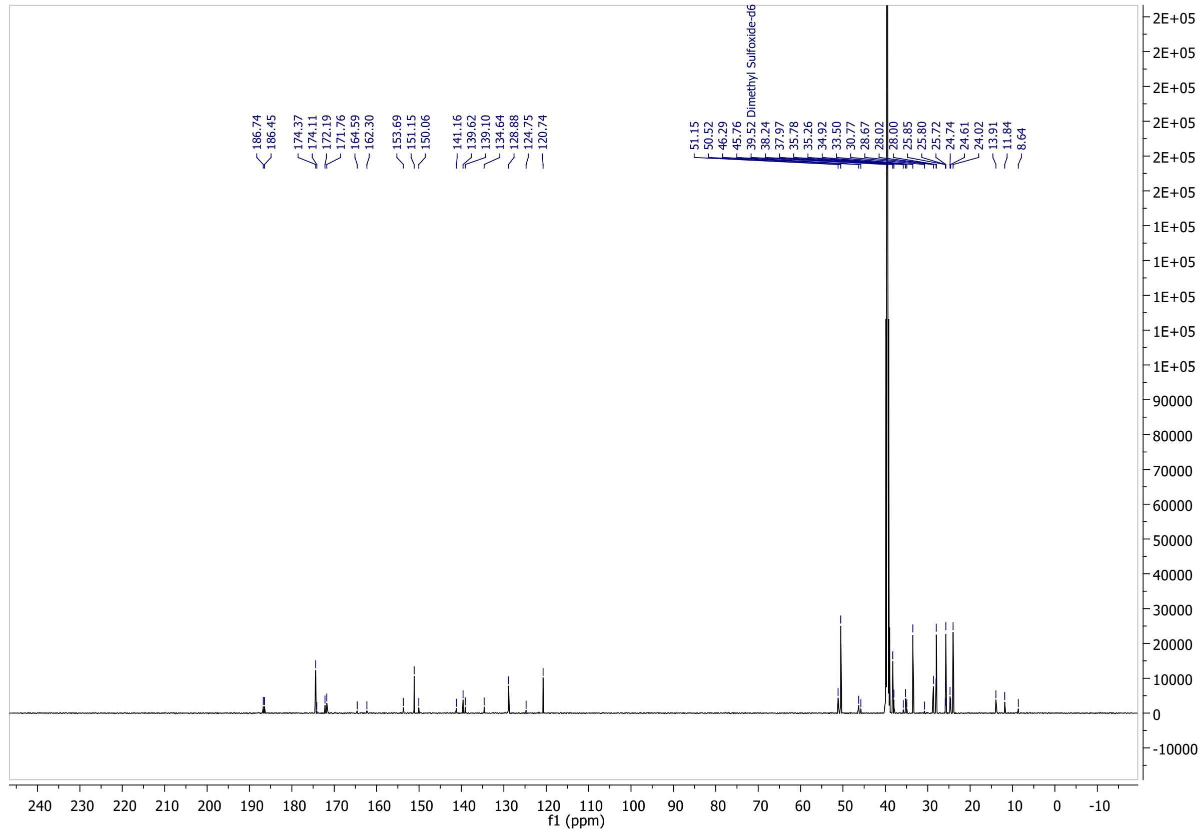

Compound **54**

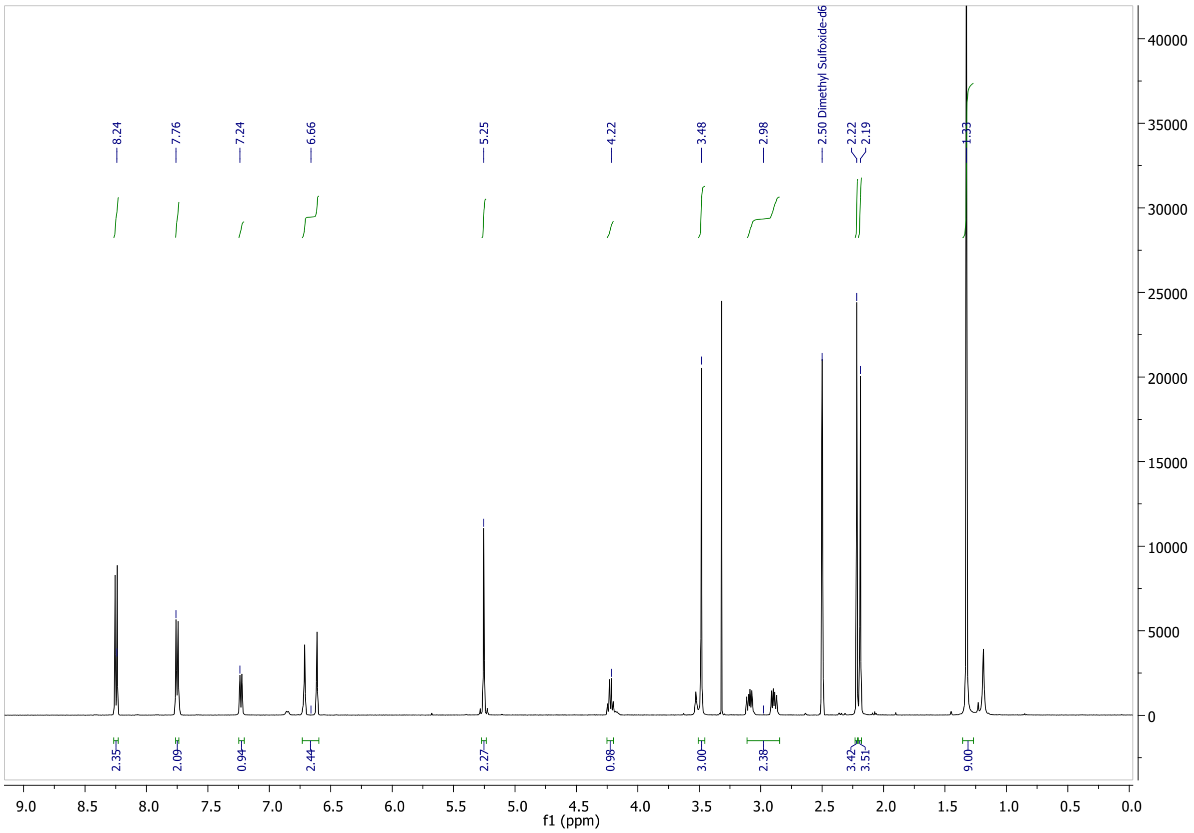

8.24
7.76
7.24
6.66
5.25
4.22
3.48
2.98
2.50 Dimethyl Sulfoxide-d6
2.22
2.19
1.33
2.35
2.09
0.94
2.44
2.27
0.98
3.00
2.38
3.42
3.51
9.00
40000
35000
30000
25000
20000
15000
10000
5000
0
9.0 8.5 8.0 7.5 7.0 6.5 6.0 5.5 5.0 4.5 4.0 3.5 3.0 2.5 2.0 1.5 1.0 0.5 0.0
f1 (ppm)

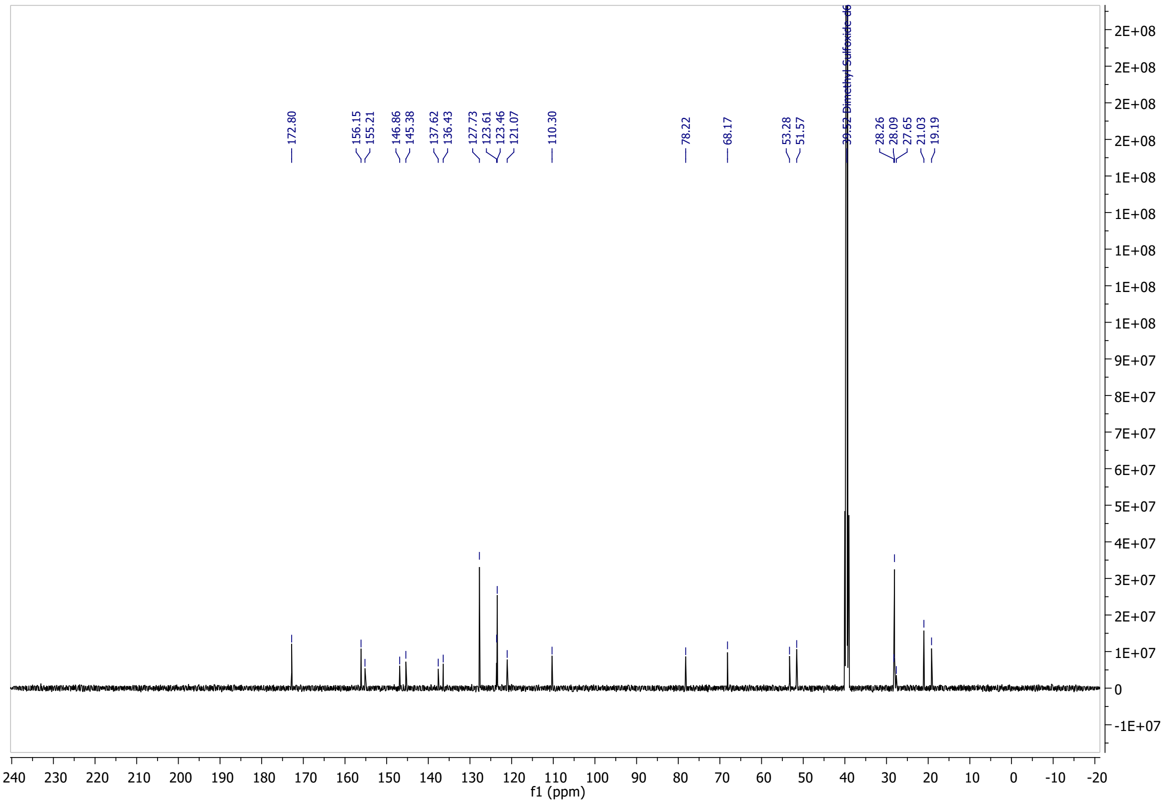

172.80
156.15
155.21
146.86
145.38
137.62
136.43
127.73
123.61
123.46
121.07
110.30
78.22
68.17
53.28
51.57
39.52 Dimethyl Sulfoxide-d6
28.26
28.09
27.65
21.03
19.19
2E+08
2E+08
2E+08
2E+08
1E+08
1E+08
1E+08
1E+08
1E+08
9E+07
8E+07
7E+07
6E+07
5E+07
4E+07
3E+07
2E+07
1E+07
0
-1E+07
240 230 220 210 200 190 180 170 160 150 140 130 120 110 100 90 80 70 60 50 40 30 20 10 0 -10 -20
f1 (ppm)

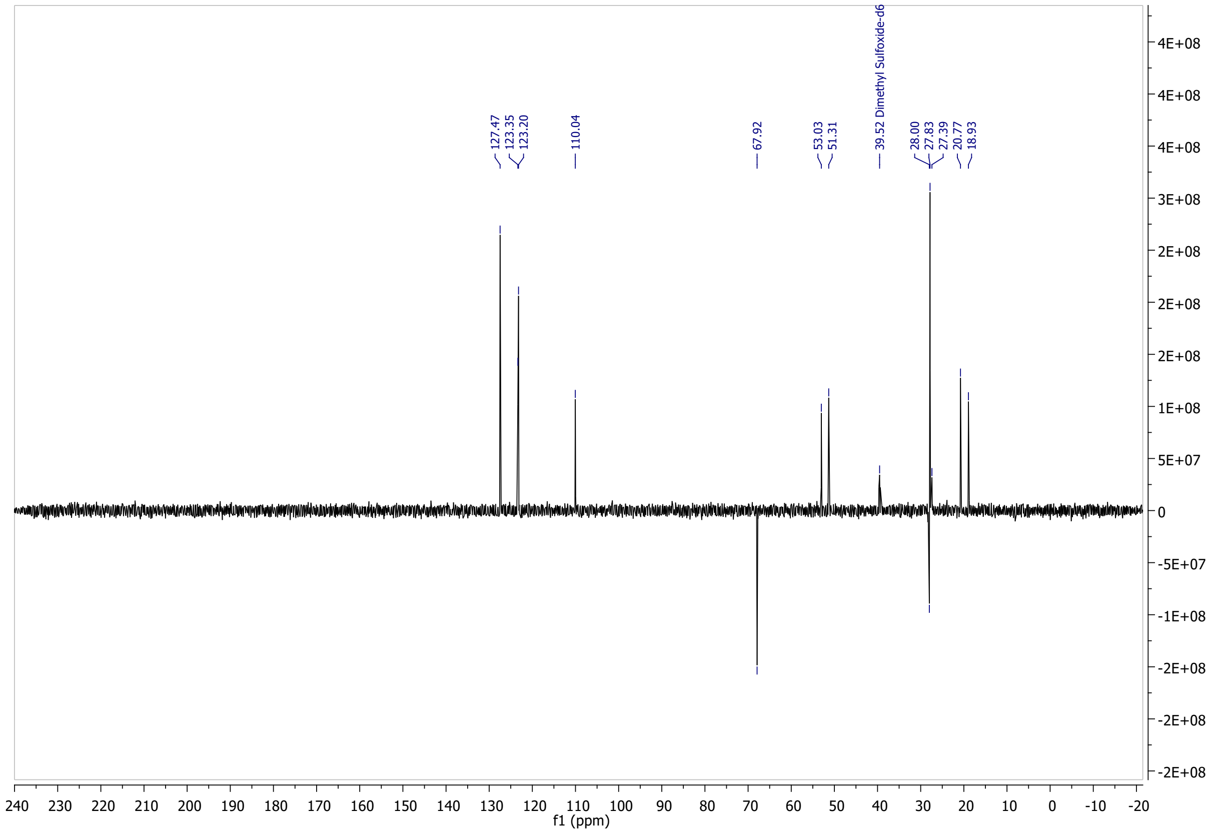

127.47
123.35
123.20
110.04
67.92
53.03
51.31
39.52 Dimethyl Sulfoxide-d6
28.00
27.83
27.39
20.77
18.93
f1 (ppm)

Compound **55**

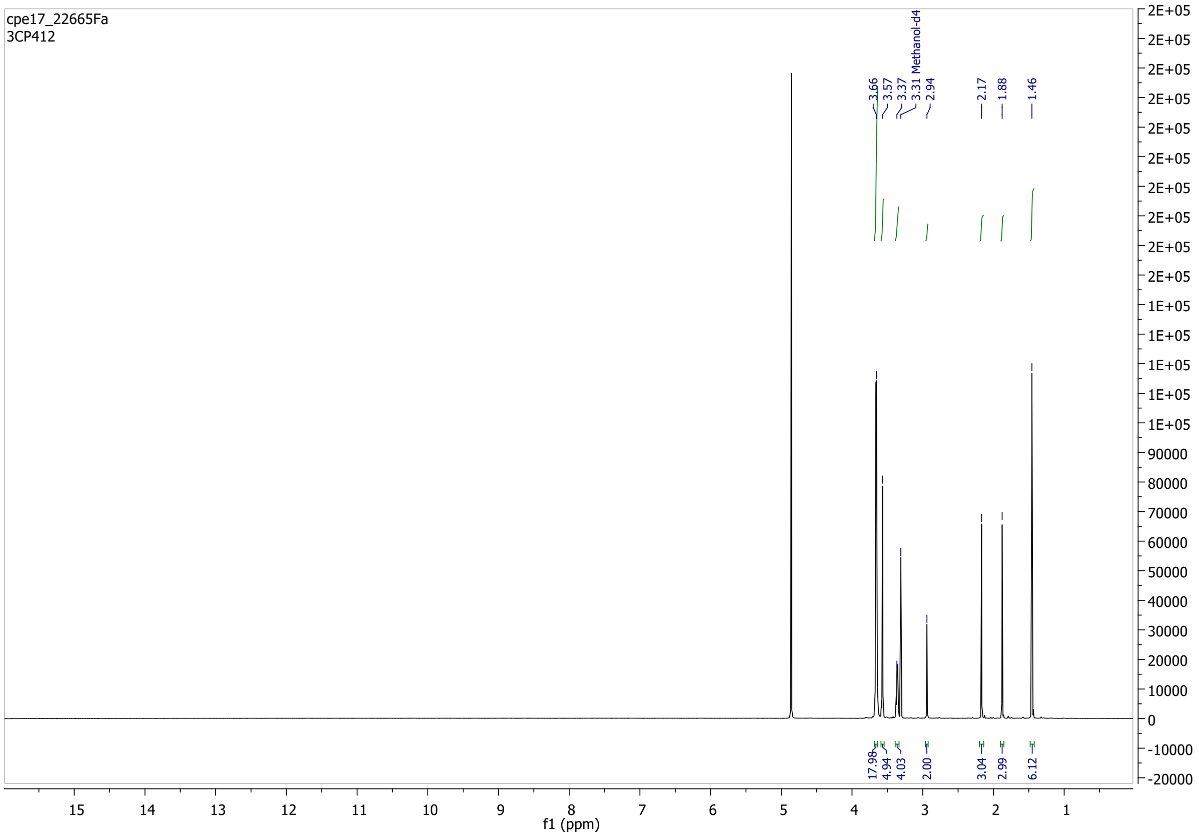
cpe17_22665Fa
3CP412
3.66
3.57
3.37
3.31 Methanol-d4
2.94
2.17
1.88
1.46
17.98
4.94
4.03
2.00
3.04
2.99
6.12
f1 (ppm)

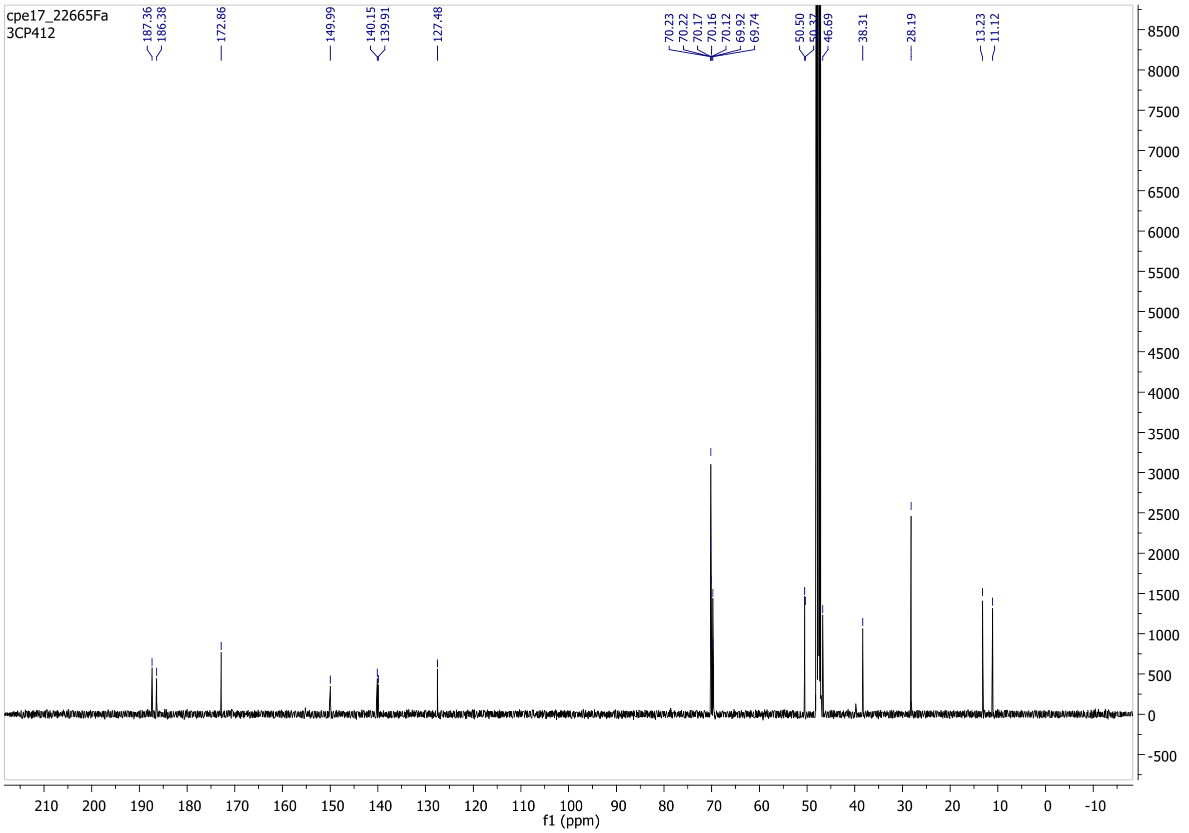
cpe17_22665Fa
3CP412
187.36
186.38
172.86
149.99
140.15
139.91
127.48
70.23
70.22
70.17
70.16
70.12
69.92
69.74
50.50
50.37
46.69
38.31
28.19
13.23
11.12
f1 (ppm)

Compound **58**

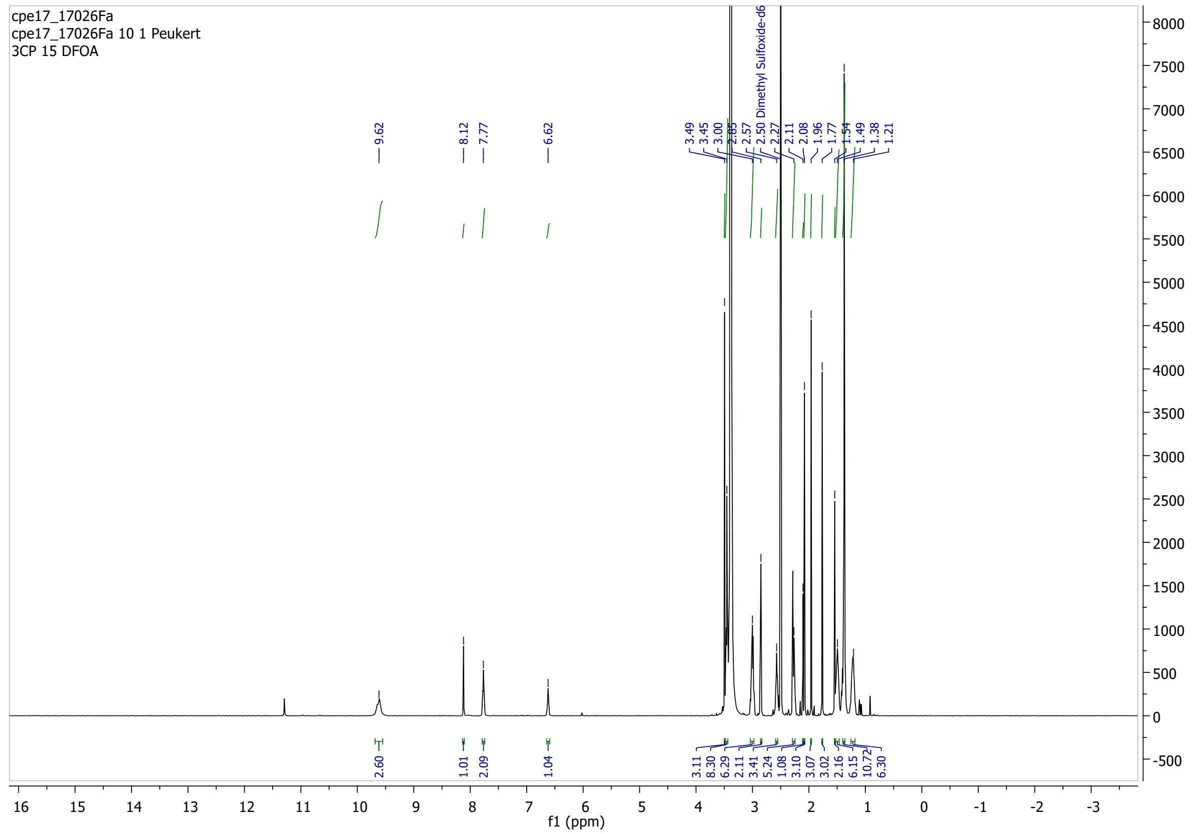

cpe17_17026Fa
cpe17_17026Fa 10 1 Peukert
3CP 15 DFOA
9.62
8.12
7.77
6.62
3.49
3.45
3.00
2.85
2.57
2.50 Dimethyl Sulfoxide-d6
2.27
2.11
2.08
1.96
1.77
1.54
1.49
1.38
1.21
2.60
1.01
2.09
1.04
3.11
8.30
6.29
2.11
3.41
5.24
1.08
3.10
3.07
3.02
2.16
6.15
10.72
6.30
f1 (ppm)

Compound **11**

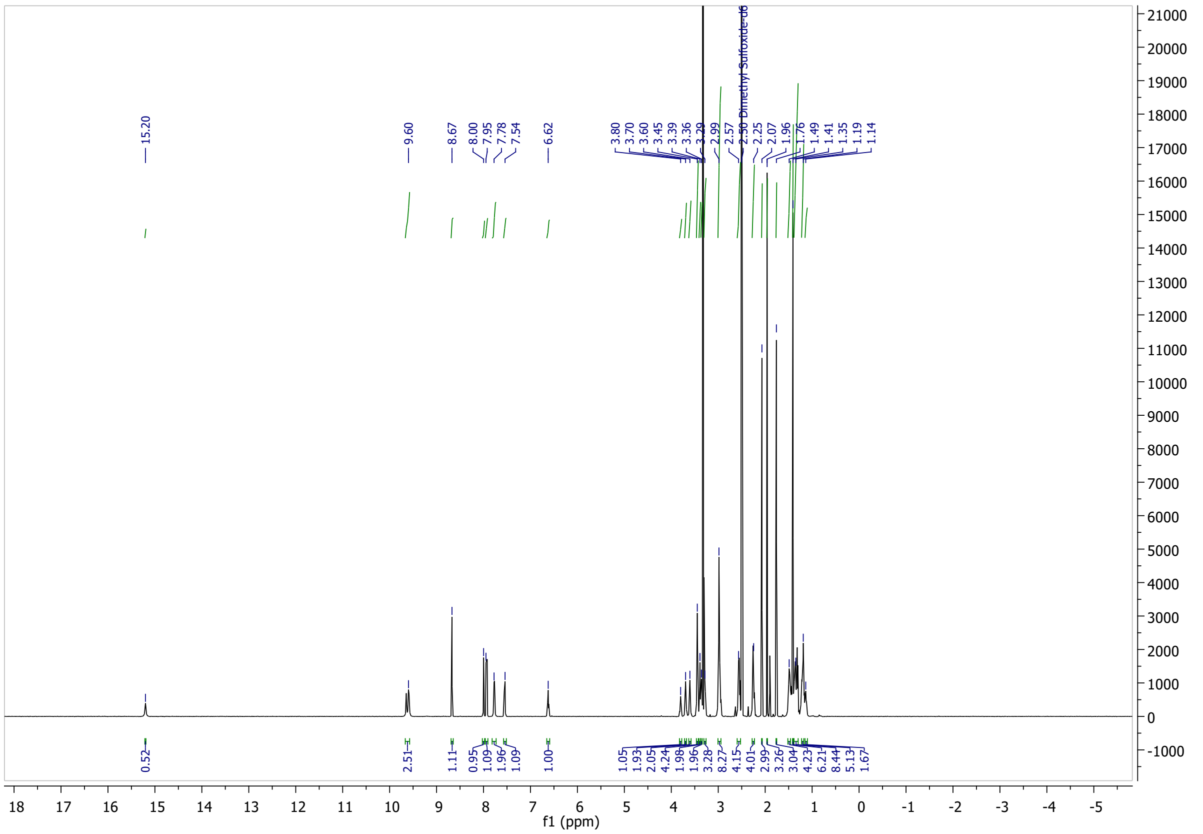

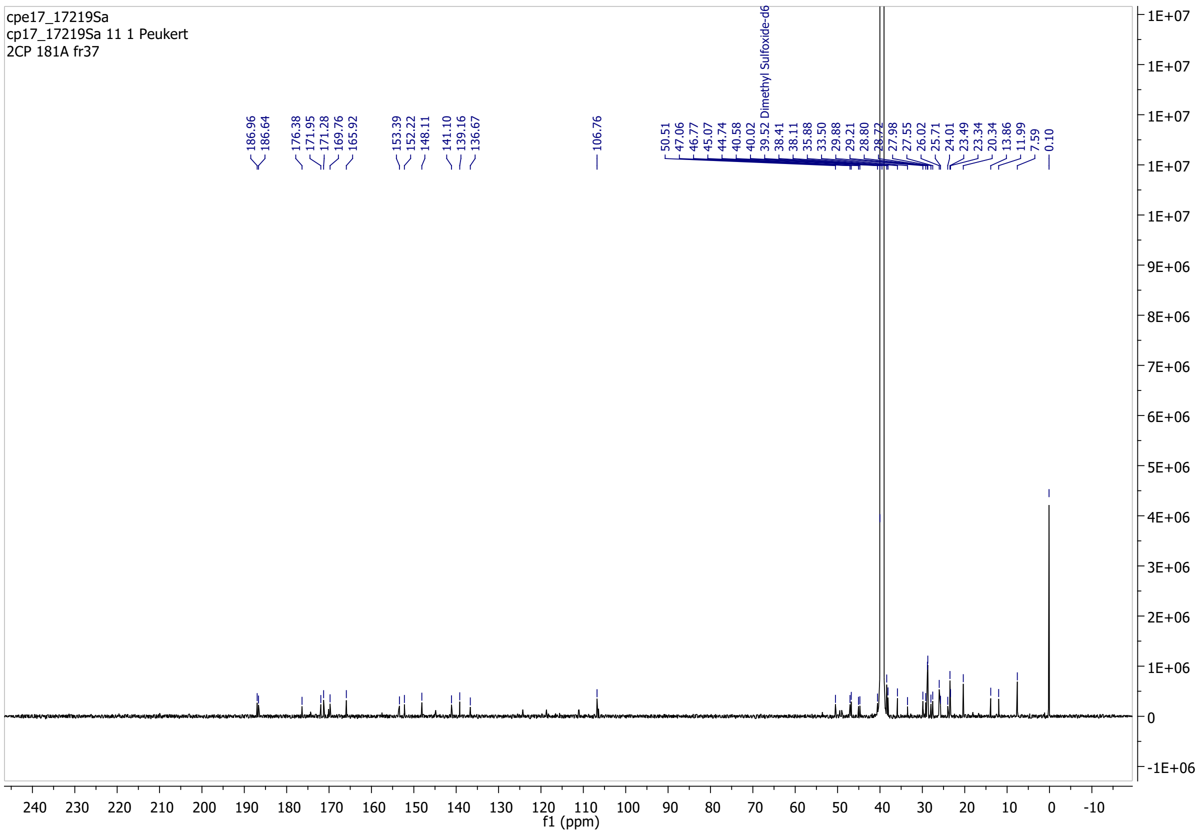

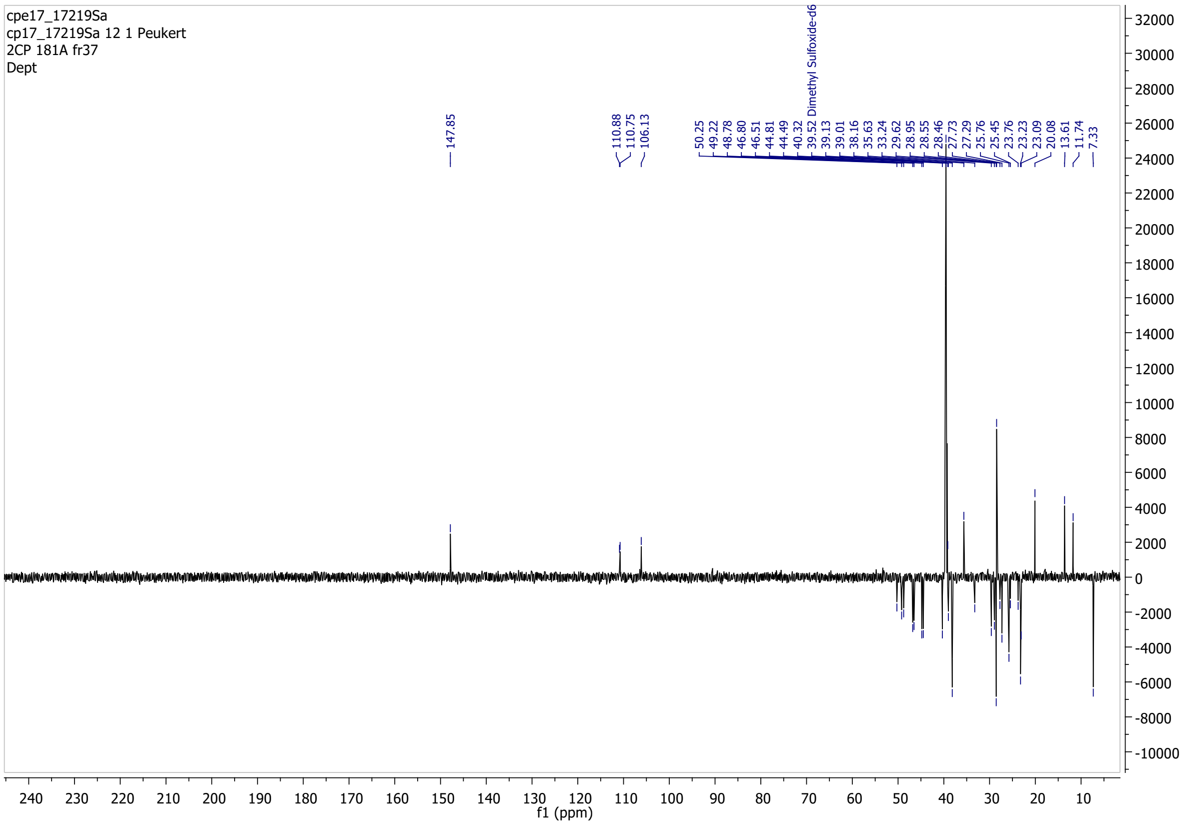
cpe17_17219Sa
cp17_17219Sa 12 1 Peukert
2CP 181A fr37
Dept
147.85
110.88
110.75
106.13
50.25
49.22
48.78
46.80
46.51
44.81
44.49
40.32
39.52 Dimethyl Sulfoxide-d6
39.13
39.01
38.16
35.63
33.24
29.62
28.95
28.55
28.46
27.73
27.29
25.76
25.45
23.76
23.23
23.09
20.08
13.61
11.74
7.33
f1 (ppm)

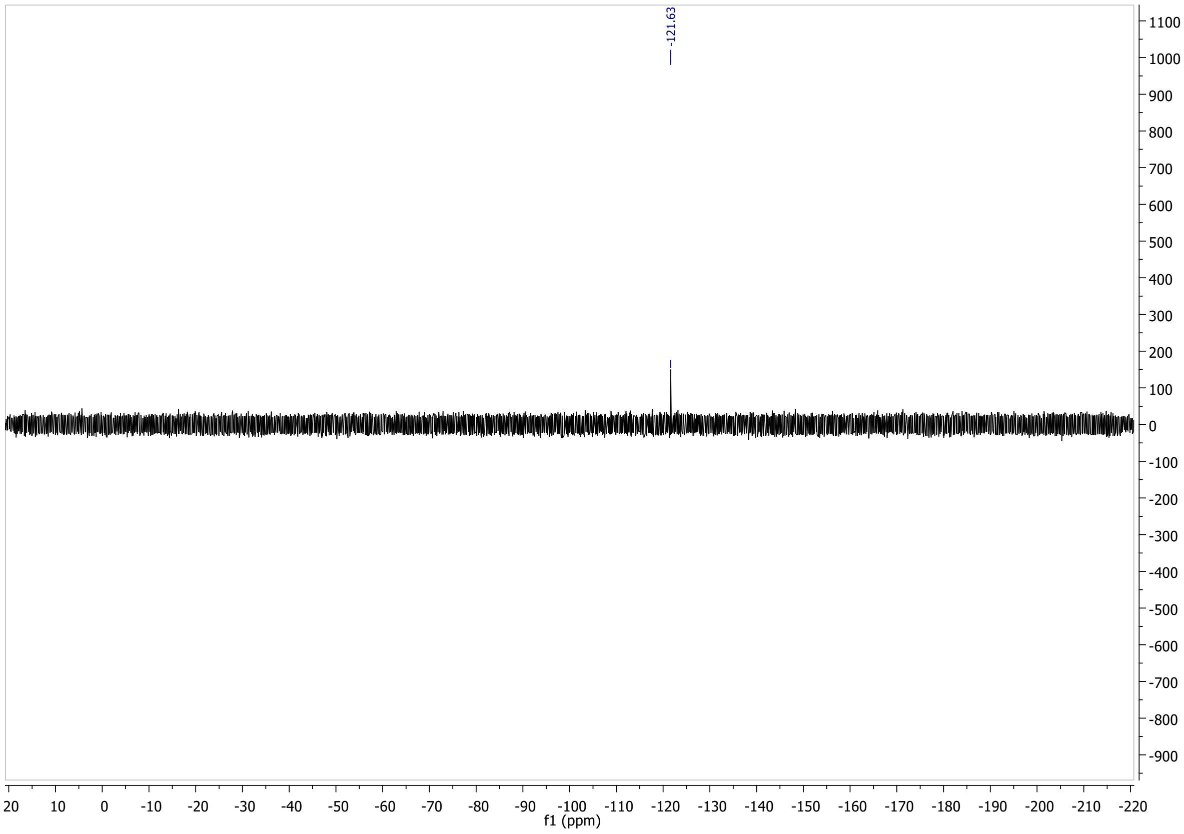
-121.63
f1 (ppm)

Compound **59**

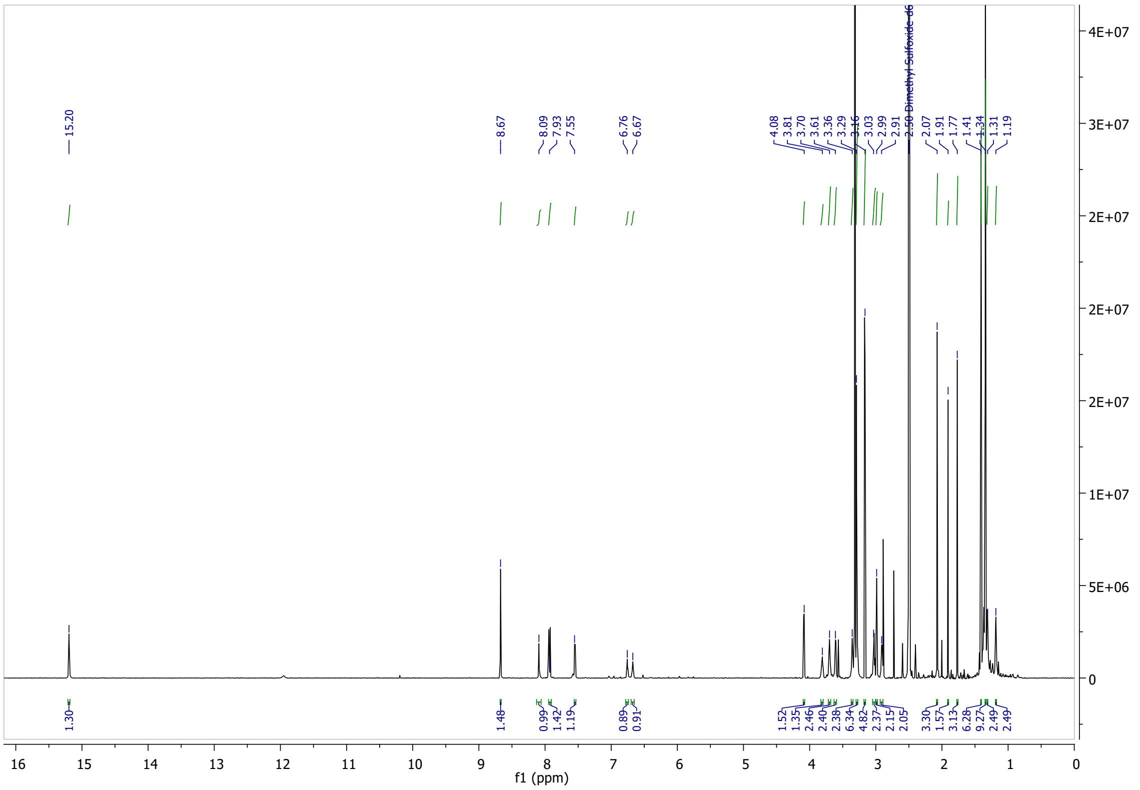
15.20
8.67
8.09
7.93
7.55
6.76
6.67
4.08
3.81
3.70
3.61
3.36
3.29
3.16
3.03
2.99
2.91
2.50 Dimethyl Sulfoxide-d6
2.07
1.91
1.77
1.41
1.34
1.31
1.19
1.30
1.48
0.99
1.42
1.19
0.89
0.91
1.52
1.35
2.46
2.40
2.38
6.34
4.82
2.37
2.15
2.05
3.30
1.57
3.13
6.28
9.27
2.49
2.49
f1 (ppm)

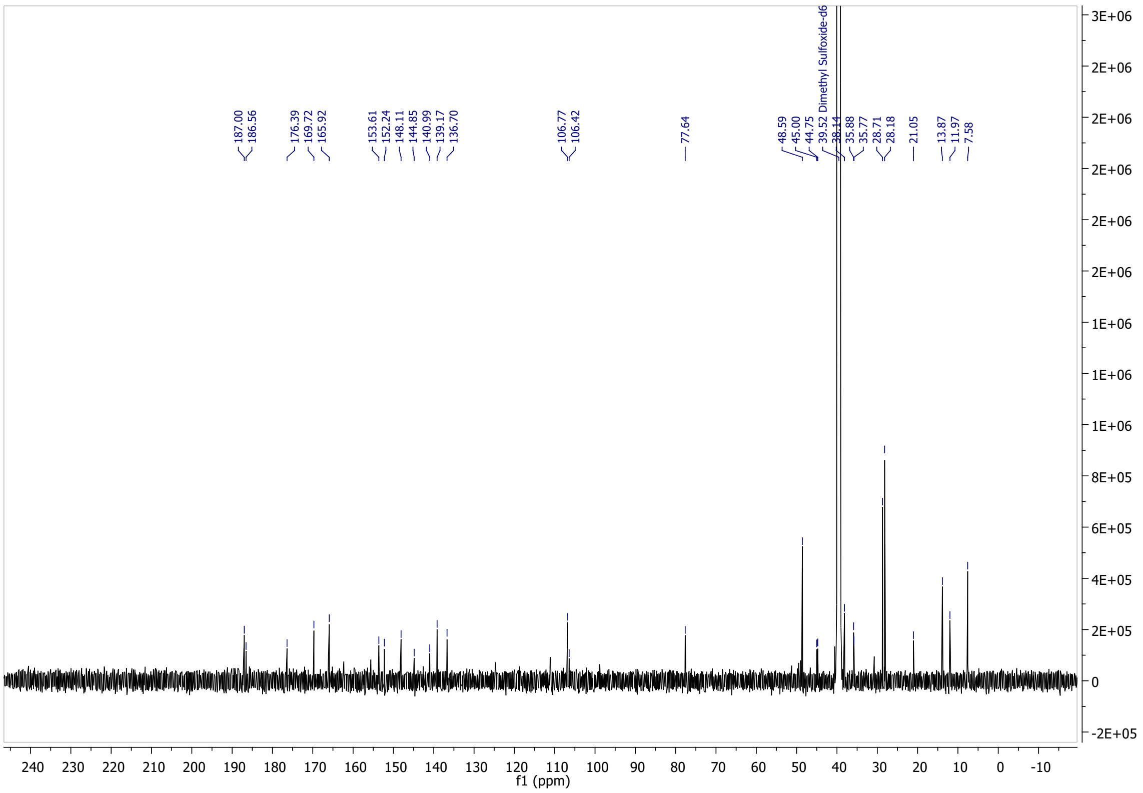
187.00
186.56
176.39
169.72
165.92
153.61
152.24
148.11
144.85
140.99
139.17
136.70
106.77
106.42
77.64
48.59
45.00
44.75
39.52 Dimethyl Sulfoxide-d6
38.14
35.88
35.77
28.71
28.18
21.05
13.87
11.97
7.58
f1 (ppm)

Compound **8**

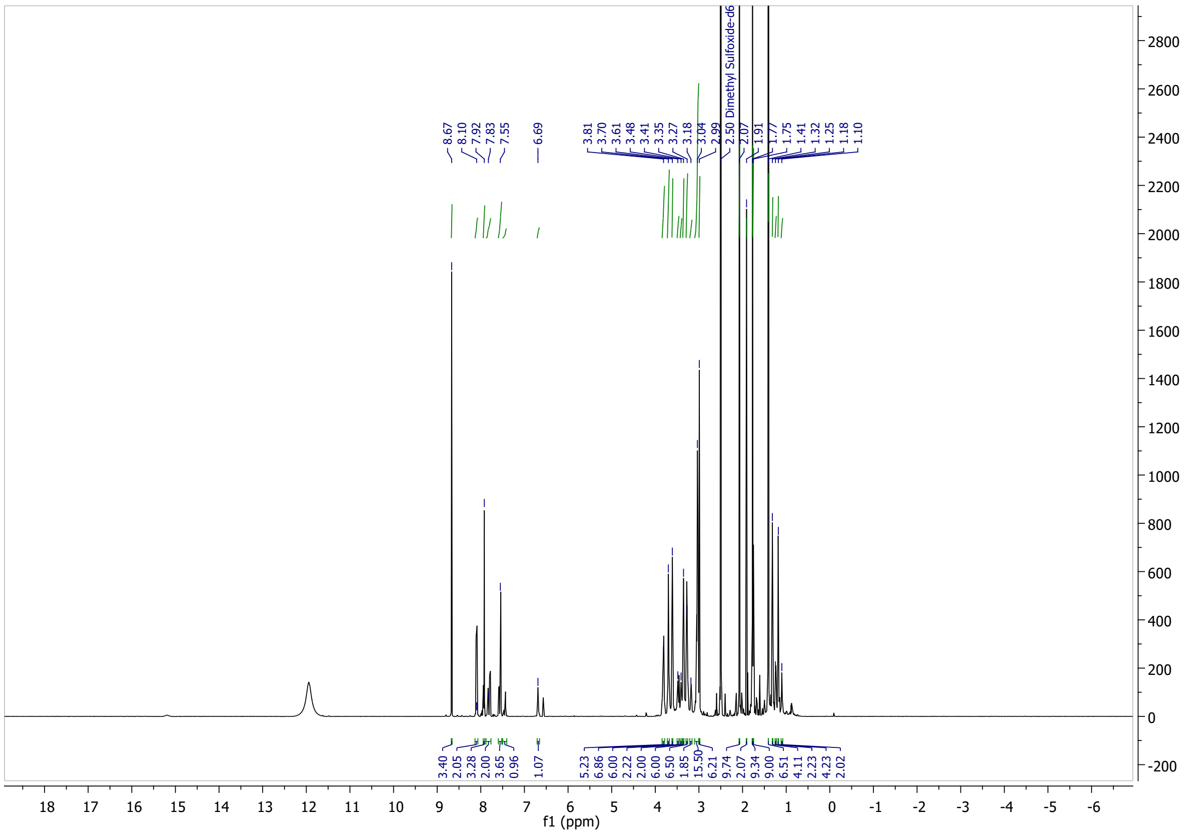
8.67
8.10
7.92
7.83
7.55
6.69
3.81
3.70
3.61
3.48
3.41
3.35
3.27
3.18
3.04
2.99
2.50 Dimethyl Sulfoxide-d6
2.07
1.91
1.77
1.75
1.41
1.32
1.25
1.18
1.10
3.40
2.05
3.28
2.00
3.65
0.96
1.07
5.23
6.86
6.00
2.22
2.00
6.00
6.50
1.85
15.50
6.21
9.74
2.07
9.34
9.00
6.51
4.11
2.23
4.23
2.02
f1 (ppm)

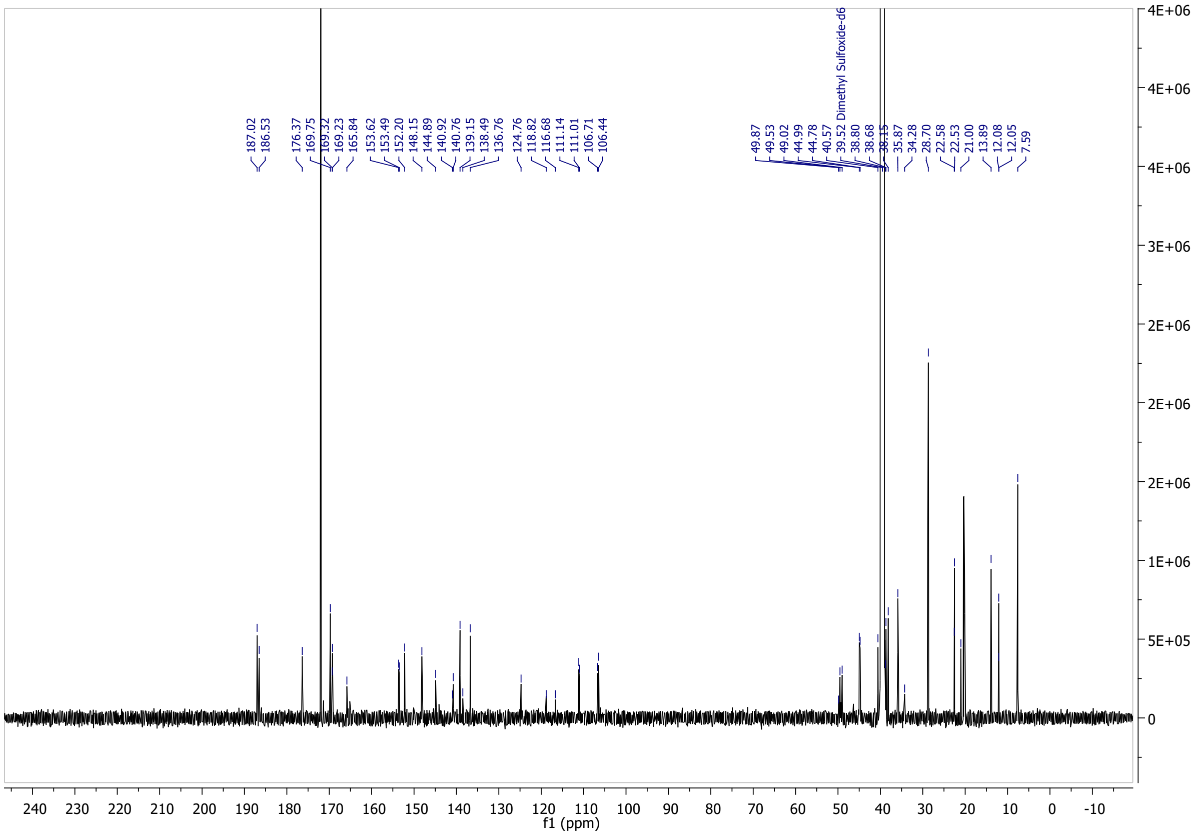
187.02
186.53
176.37
169.75
169.32
169.23
165.84
153.62
153.49
152.20
148.15
144.89
140.92
140.76
139.15
138.49
136.76
124.76
118.82
116.68
111.14
111.01
106.71
106.44
49.87
49.53
49.02
44.99
44.78
40.57
39.52 Dimethyl Sulfoxide-d6
38.80
38.68
38.15
35.87
34.28
28.70
22.58
22.53
21.00
13.89
12.08
12.05
7.59
f1 (ppm)

Compound **17**

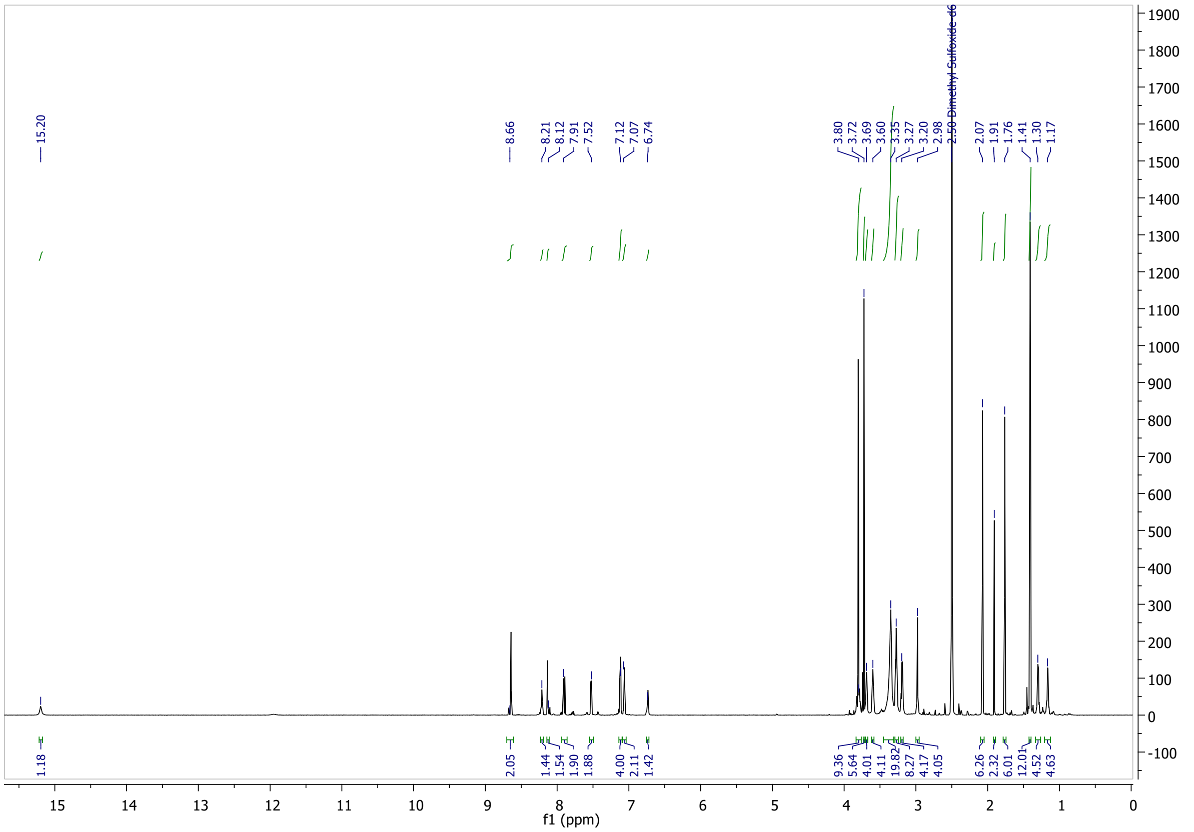
15.20
8.66
8.21
8.12
7.91
7.52
7.12
7.07
6.74
3.80
3.72
3.69
3.60
3.35
3.27
3.20
2.98
2.50 Dimethyl Sulfoxide-d6
2.07
1.91
1.76
1.41
1.30
1.17
1.18
2.05
1.44
1.54
1.90
1.88
4.00
2.11
1.42
9.36
5.64
4.01
4.11
19.82
8.27
4.17
4.05
6.26
2.32
6.01
12.01
4.52
4.63
f1 (ppm)

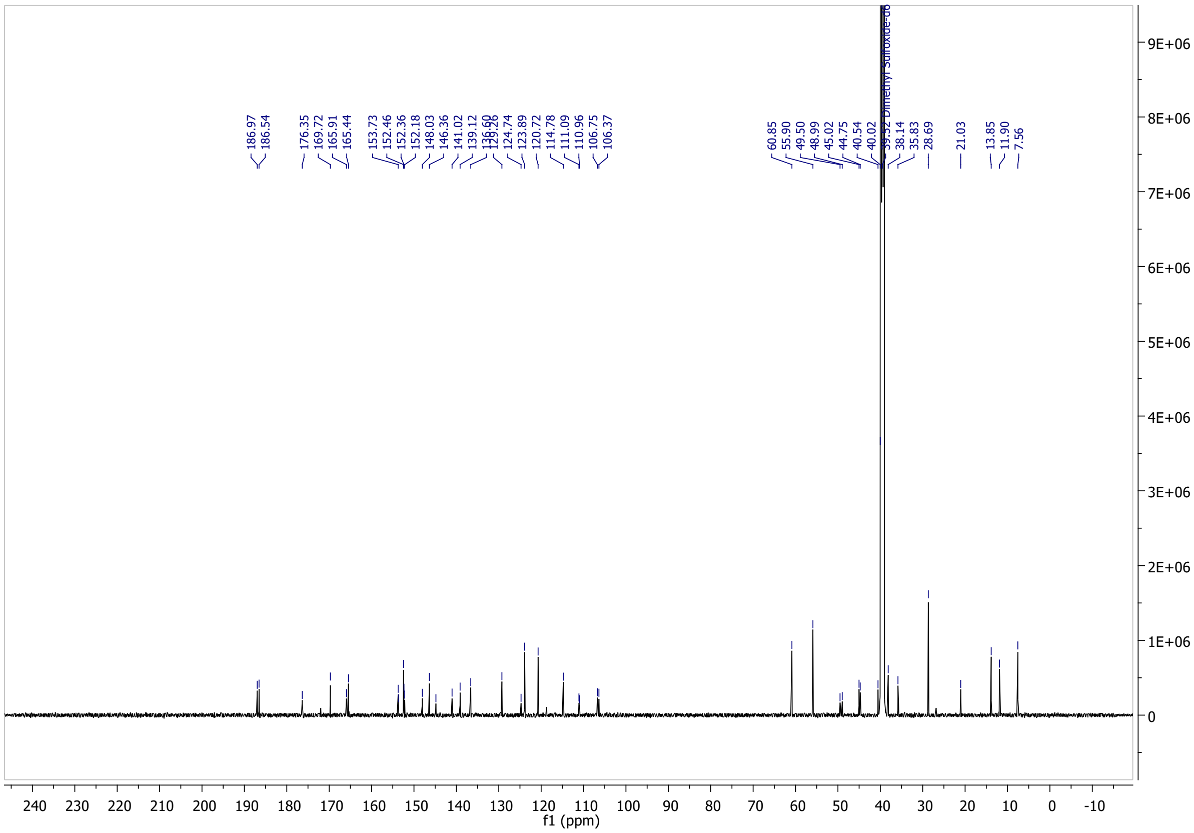
186.97
186.54
176.35
169.72
165.91
165.44
153.73
152.46
152.36
152.18
148.03
146.36
141.02
139.12
136.60
129.28
124.74
123.89
120.72
114.78
111.09
110.96
106.75
106.37
60.85
55.90
49.50
48.99
45.02
44.75
40.54
40.02
39.52 Dimethyl Sulfoxide-d6
38.14
35.83
28.69
21.03
13.85
11.90
7.56
f1 (ppm)

Compound **9**

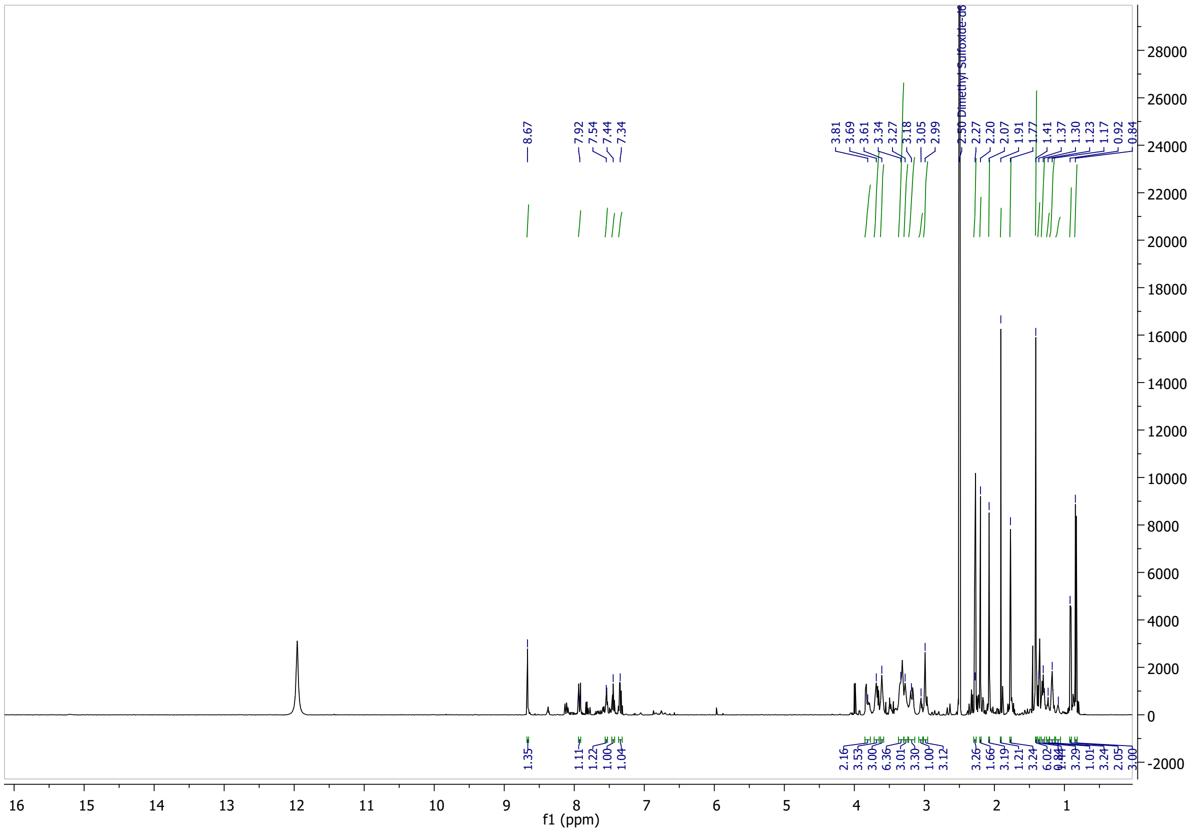

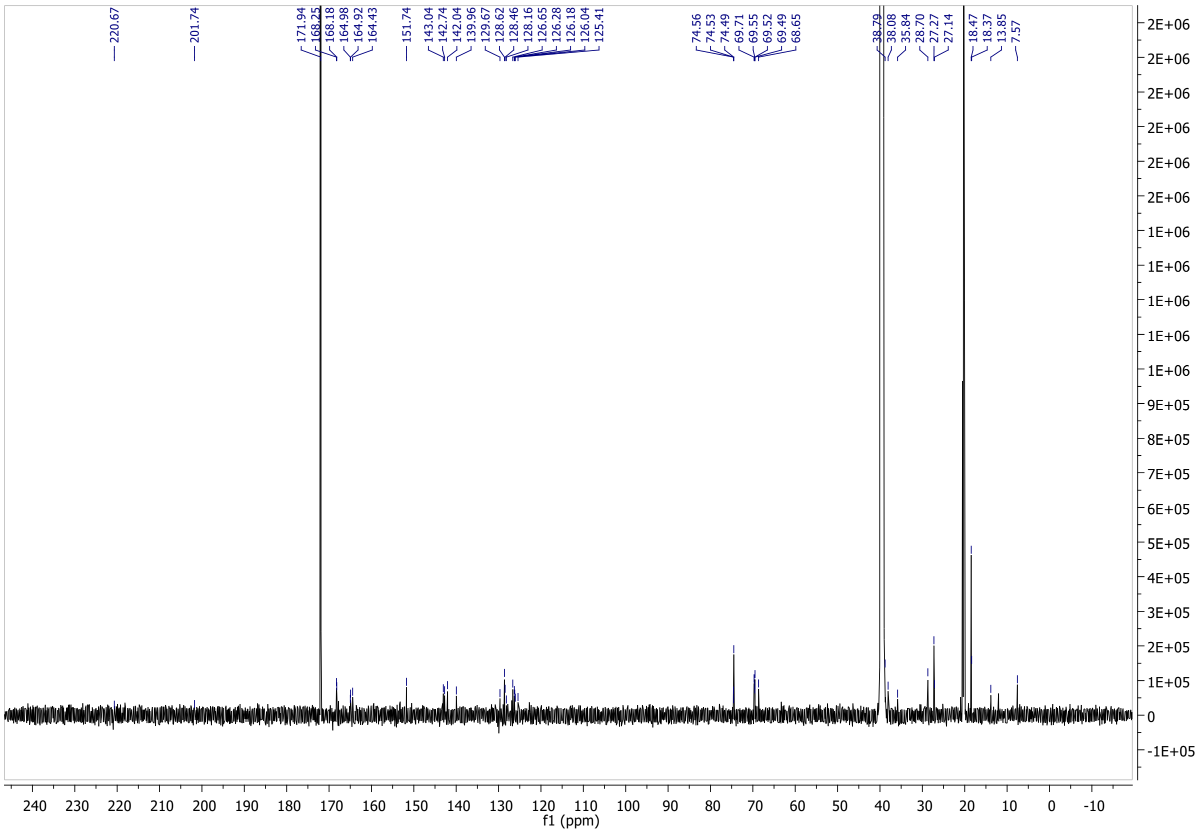

Compound **10**

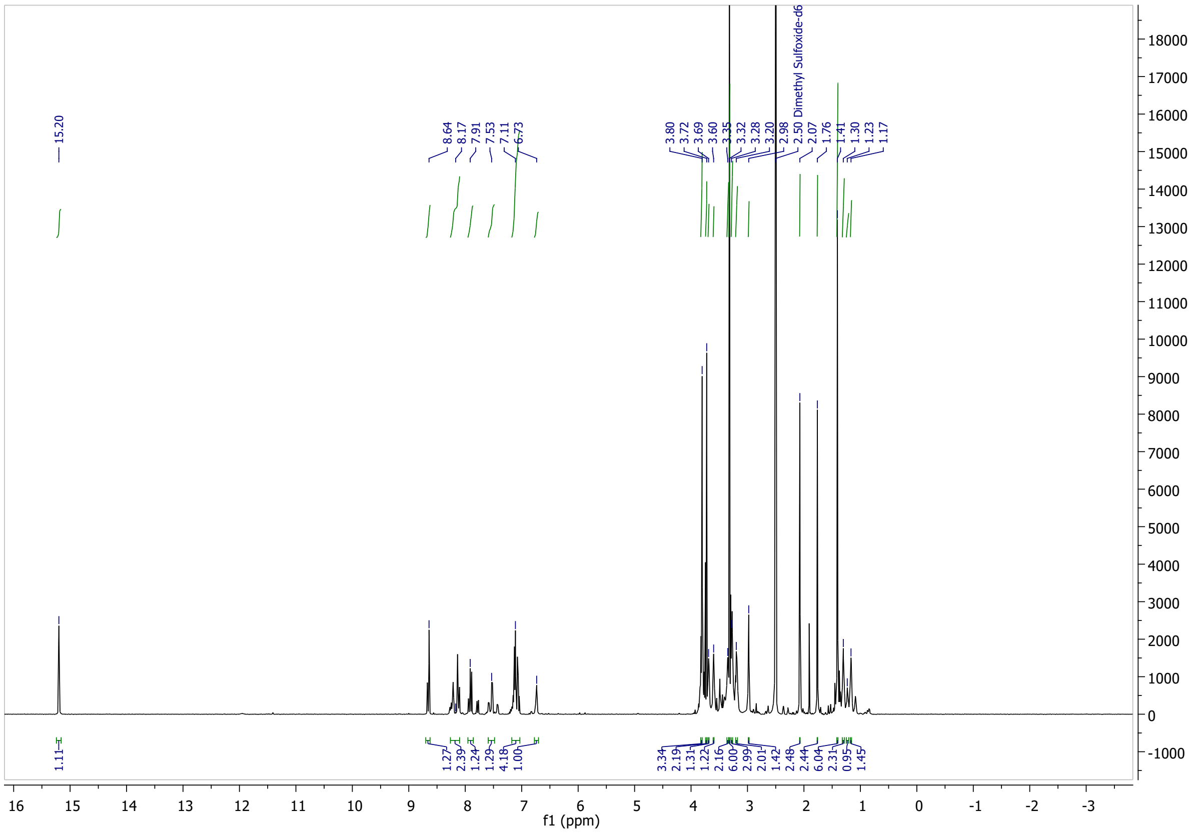
15.20
8.64
8.17
7.91
7.53
7.11
6.73
3.80
3.72
3.69
3.60
3.35
3.32
3.28
3.20
2.98
2.50 Dimethyl Sulfoxide-d6
2.07
1.76
1.41
1.30
1.23
1.17
1.11
1.27
2.39
1.24
1.29
4.18
1.00
3.34
2.19
1.31
1.22
2.16
6.00
2.99
2.01
1.42
2.48
2.44
6.04
2.31
0.95
1.45
f1 (ppm)

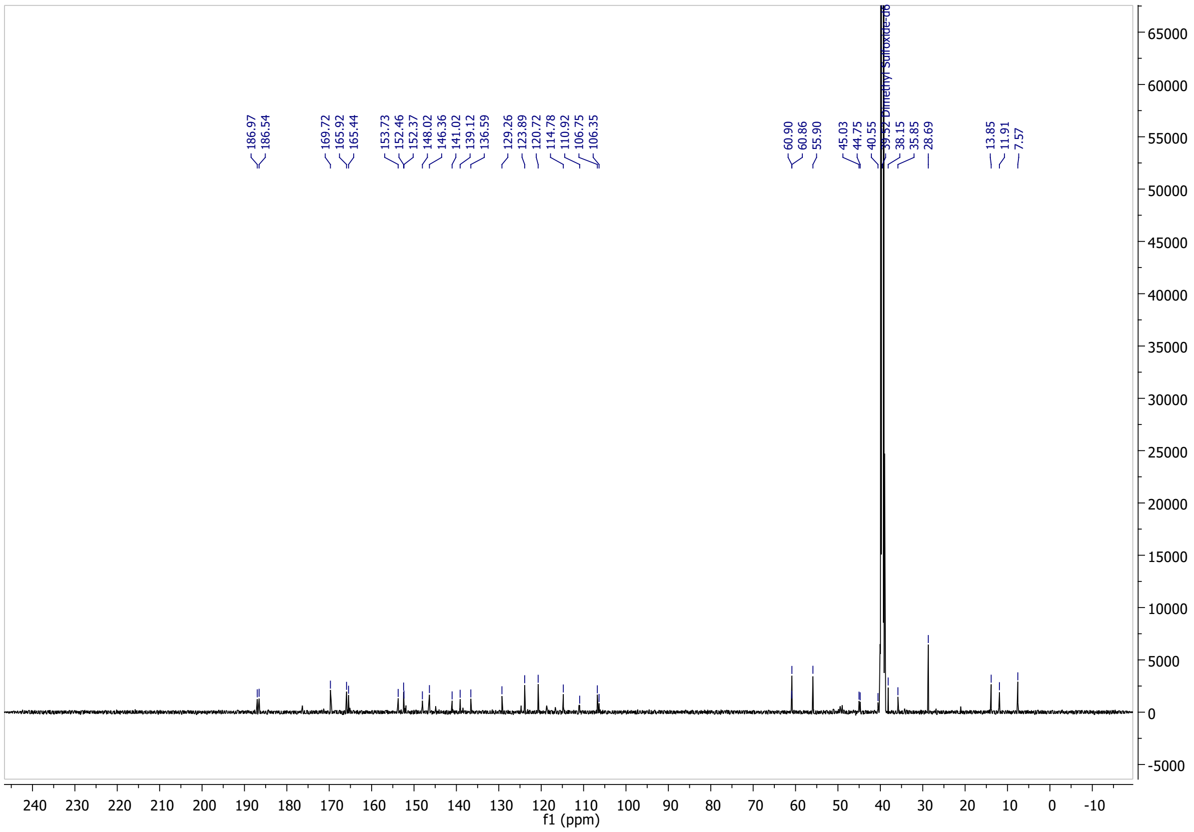
186.97
186.54
169.72
165.92
165.44
153.73
152.46
152.37
148.02
146.36
141.02
139.12
136.59
129.26
123.89
120.72
114.78
110.92
106.75
106.35
60.90
60.86
55.90
45.03
44.75
40.55
39.52 Dimethyl Sulfoxide-d6
38.15
35.85
28.69
13.85
11.91
7.57
f1 (ppm)

Compound **12**

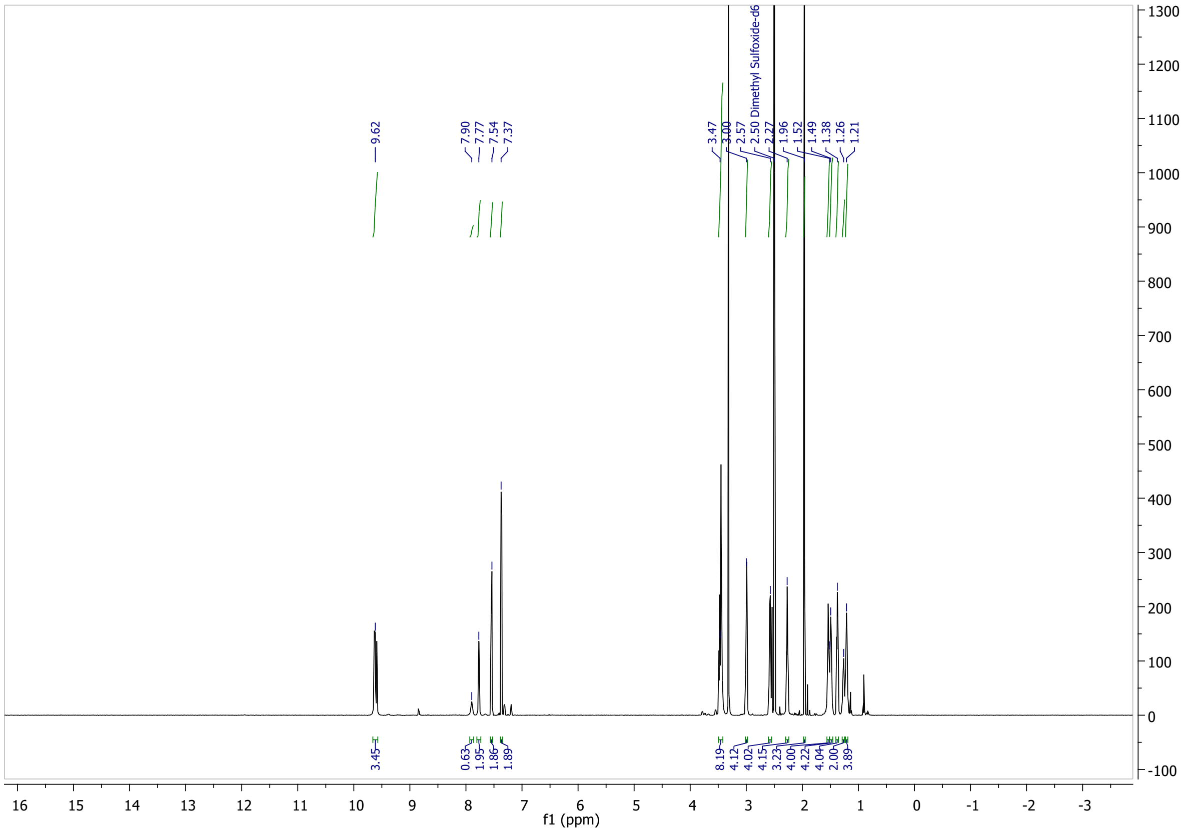

2.50 Dimethyl Sulfoxide-d6
f1 (ppm)

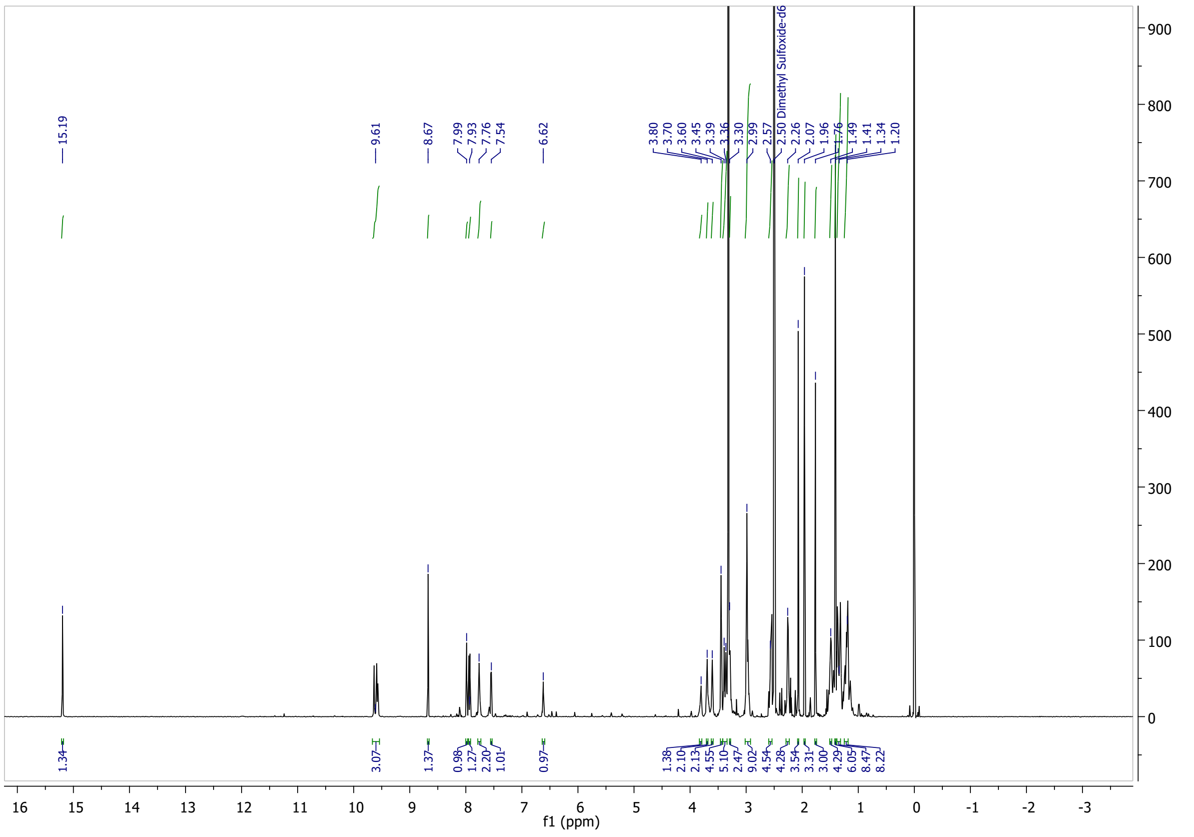

2.50 Dimethyl Sulfoxide-d6
f1 (ppm)

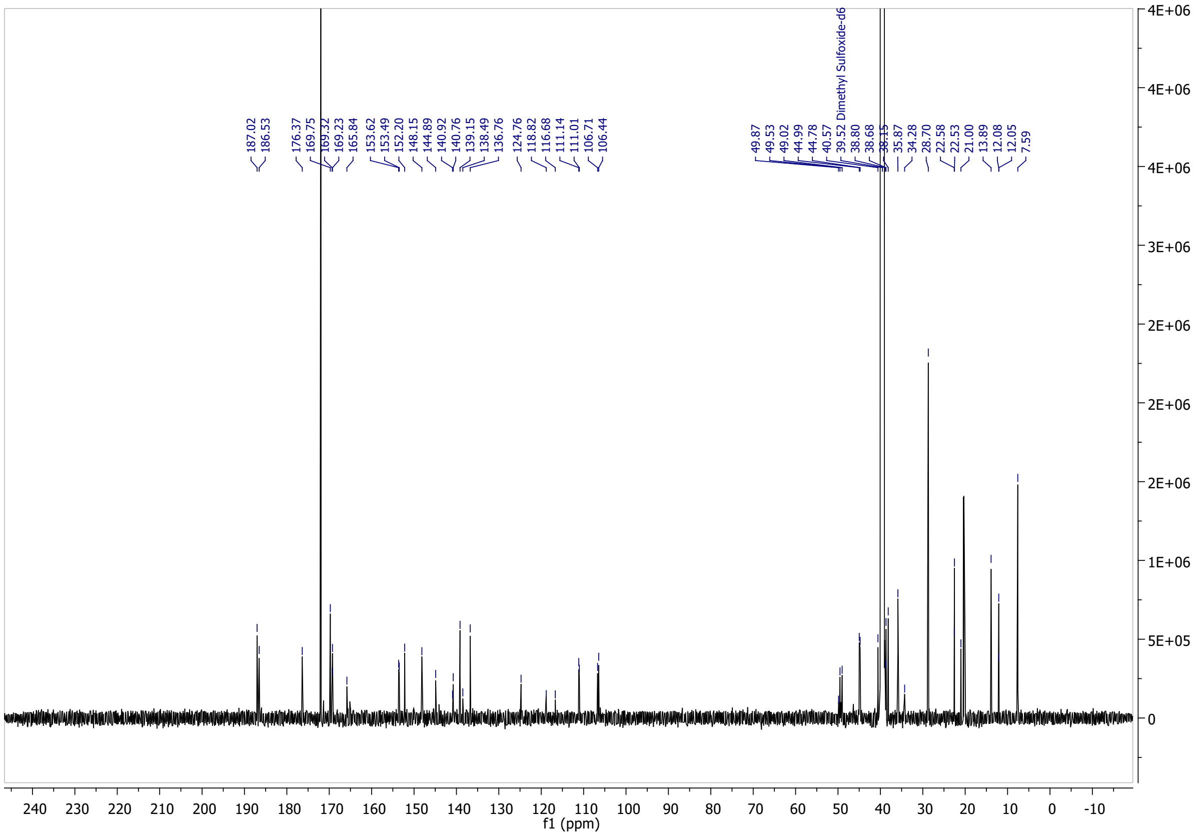

187.02
186.53
176.37
169.75
169.32
169.23
165.84
153.62
153.49
152.20
148.15
144.89
140.92
140.76
139.15
138.49
136.76
124.76
118.82
116.68
111.14
111.01
106.71
106.44
49.87
49.53
49.02
44.99
44.78
40.57
39.52 Dimethyl Sulfoxide-d6
38.80
38.68
38.15
35.87
34.28
28.70
22.58
22.53
21.00
13.89
12.08
12.05
7.59
f1 (ppm)

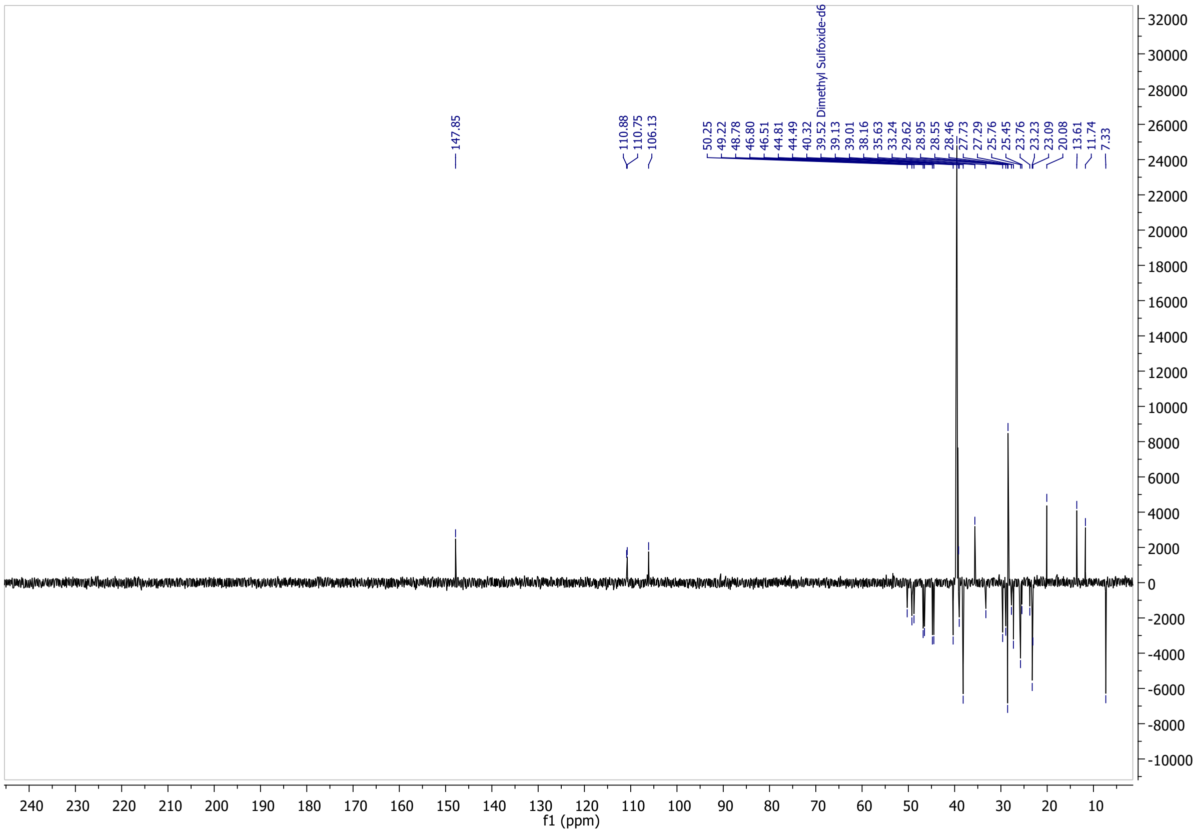
147.85
110.88
110.75
106.13
50.25
49.22
48.78
46.80
46.51
44.81
44.49
40.32
39.52 Dimethyl Sulfoxide-d6
39.13
39.01
38.16
35.63
33.24
29.62
28.95
28.55
28.46
27.73
27.29
25.76
25.45
23.76
23.23
23.09
20.08
13.61
11.74
7.33
f1 (ppm)

Compound **60**

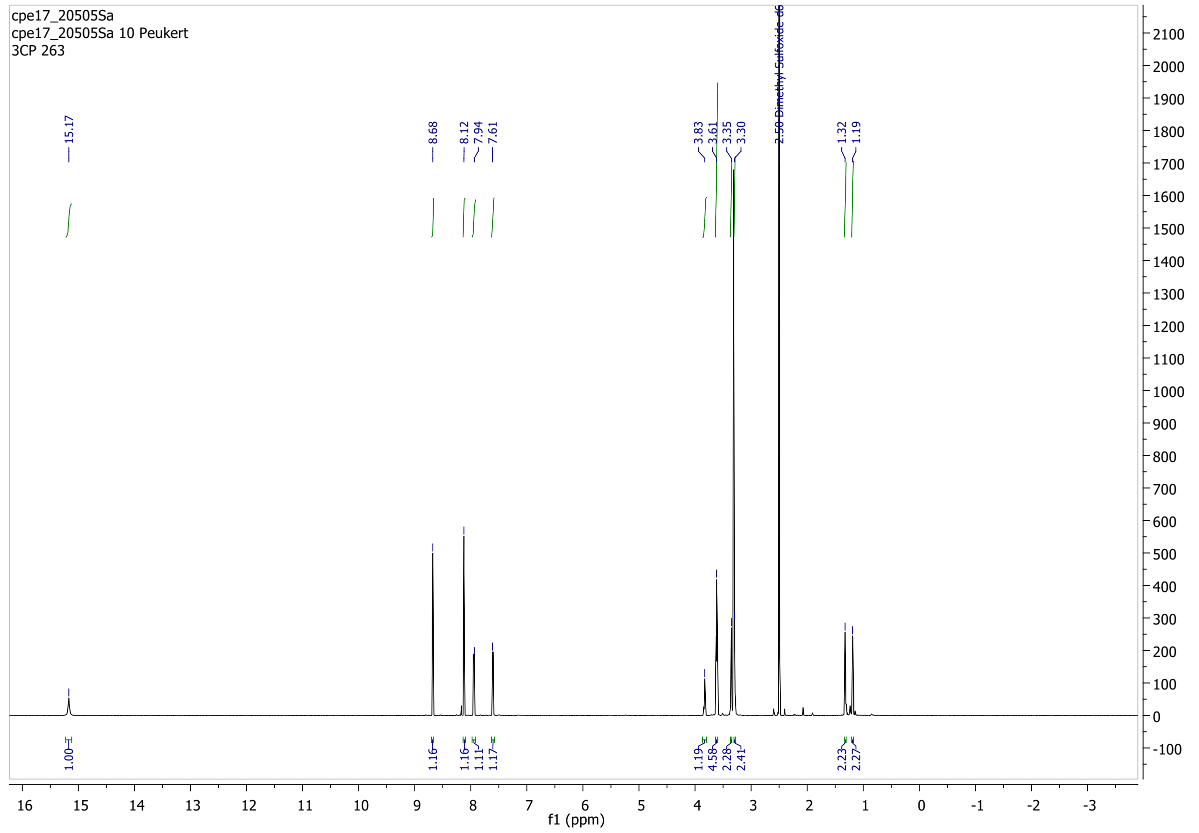
cpe17_20505Sa
cpe17_20505Sa 10 Peukert
3CP 263
15.17
8.68
8.12
7.94
7.61
3.83
3.61
3.35
3.30
2.50 Dimethyl Sulfoxide-d6
1.32
1.19
1.00
1.16
1.16
1.11
1.17
1.19
4.58
2.28
2.41
2.23
2.27
f1 (ppm)

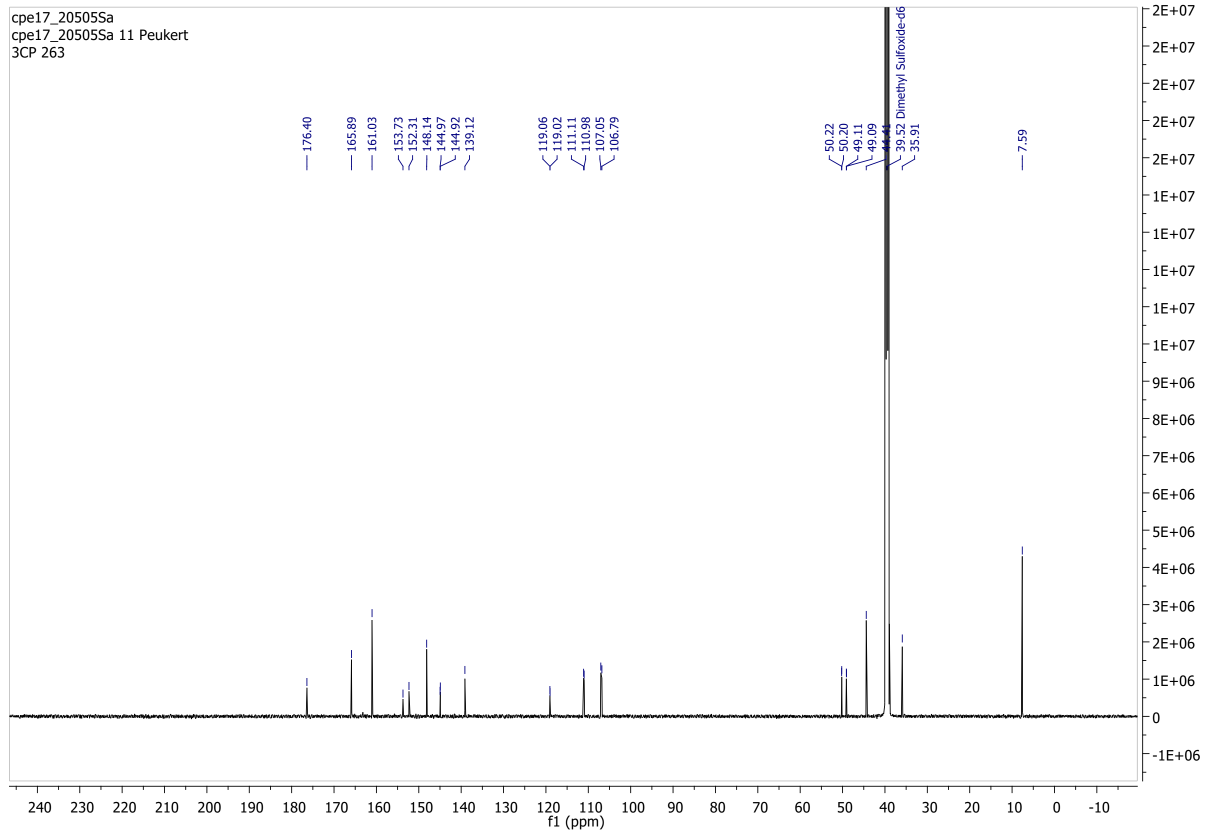
cpe17_20505Sa
cpe17_20505Sa 11 Peukert
3CP 263
176.40
165.89
161.03
153.73
152.31
148.14
144.97
144.92
139.12
119.06
119.02
111.11
110.98
107.05
106.79
50.22
50.20
49.11
49.09
44.41
39.52 Dimethyl Sulfoxide-d6
35.91
7.59
f1 (ppm)

Compound **13**

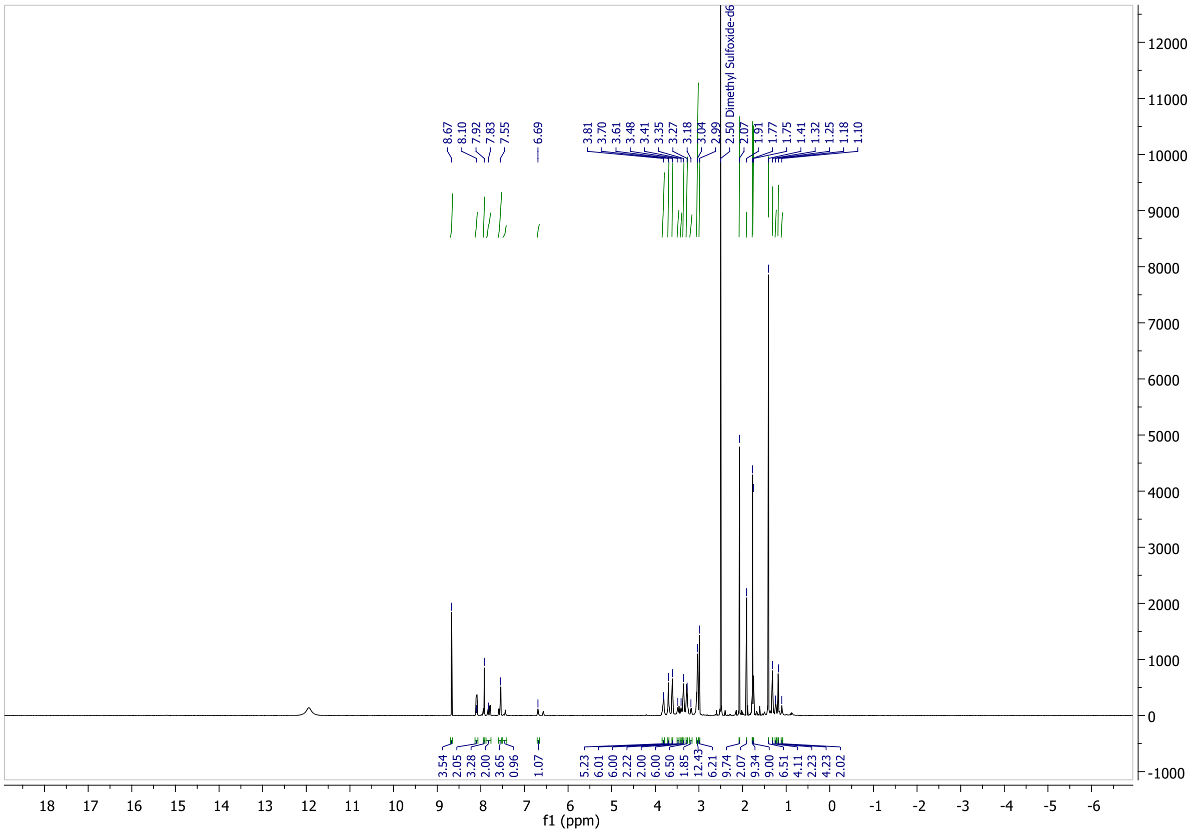
8.67
8.10
7.92
7.83
7.55
6.69
3.81
3.70
3.61
3.48
3.41
3.35
3.27
3.18
3.04
2.99
2.50 Dimethyl Sulfoxide-d6
2.07
1.91
1.77
1.75
1.41
1.32
1.25
1.18
1.10
3.54
2.05
3.28
2.00
3.65
0.96
1.07
5.23
6.01
6.00
2.22
2.00
6.00
6.50
1.85
12.43
6.21
9.74
2.07
9.34
9.00
6.51
4.11
2.23
4.23
2.02
f1 (ppm)

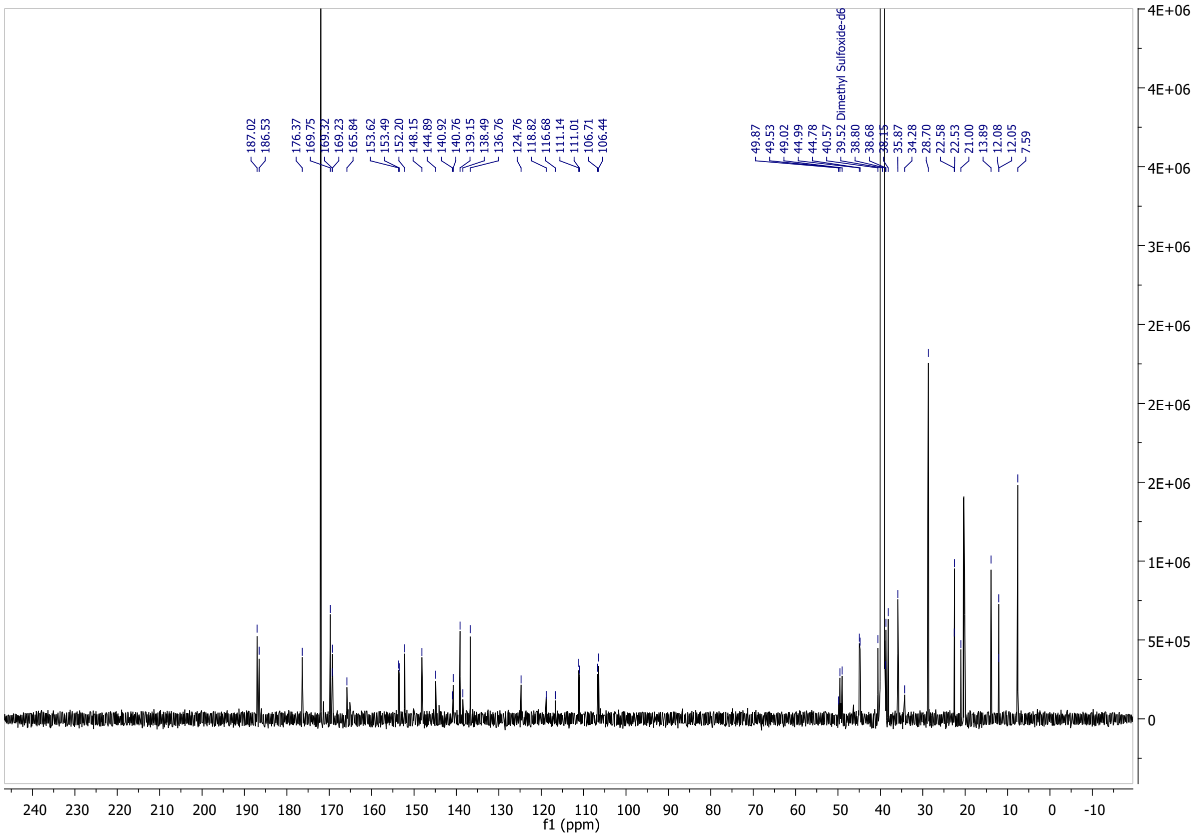
187.02
186.53
176.37
169.75
169.32
169.23
165.84
153.62
153.49
152.20
148.15
144.89
140.92
140.76
139.15
138.49
136.76
124.76
118.82
116.68
111.14
111.01
106.71
106.44
49.87
49.53
49.02
44.99
44.78
40.57
39.52 Dimethyl Sulfoxide-d6
38.80
38.68
38.15
35.87
34.28
28.70
22.58
22.53
21.00
13.89
12.08
12.05
7.59
f1 (ppm)

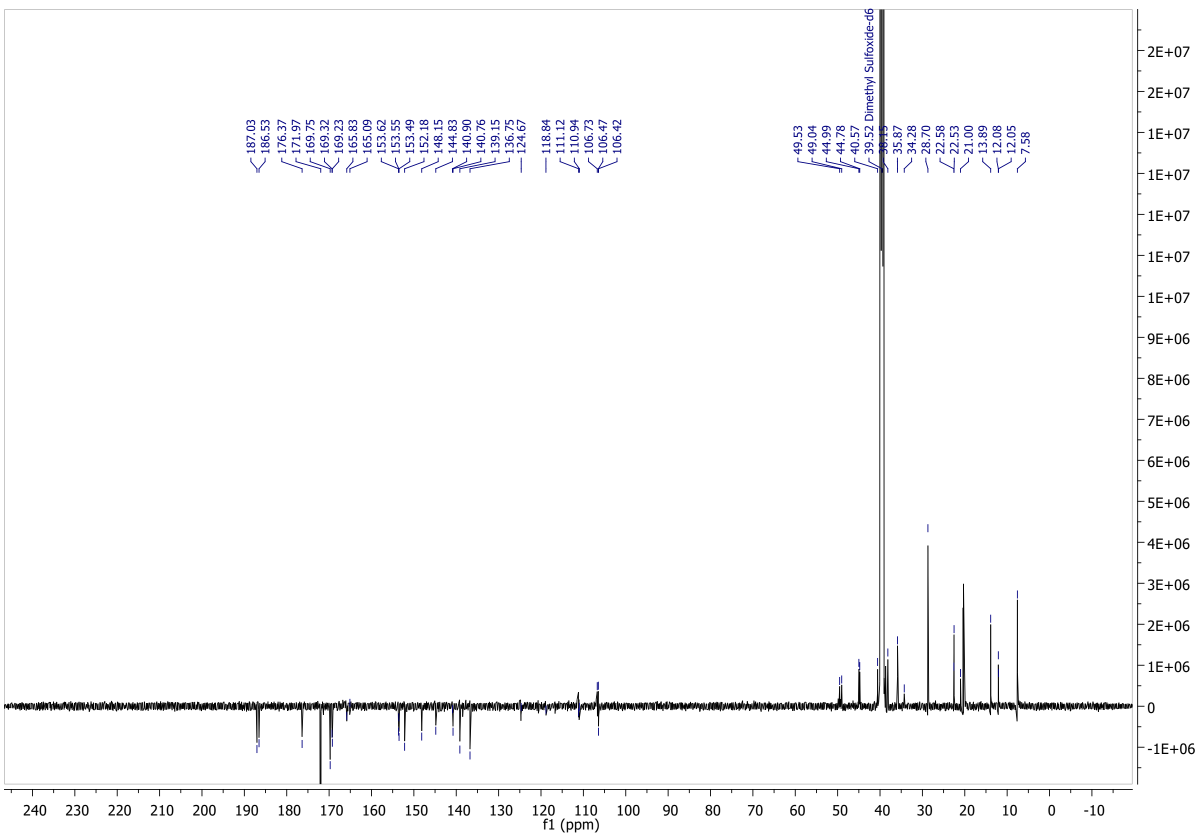

187.03
186.53
176.37
171.97
169.75
169.32
169.23
165.83
165.09
153.62
153.55
153.49
152.18
148.15
144.83
140.90
140.76
139.15
136.75
124.67
118.84
111.12
110.94
106.73
106.47
106.42
49.53
49.04
44.99
44.78
40.57
39.52 Dimethyl Sulfoxide-d6
38.15
35.87
34.28
28.70
22.58
22.53
21.00
13.89
12.08
12.05
7.58
f1 (ppm)

Compound **14**

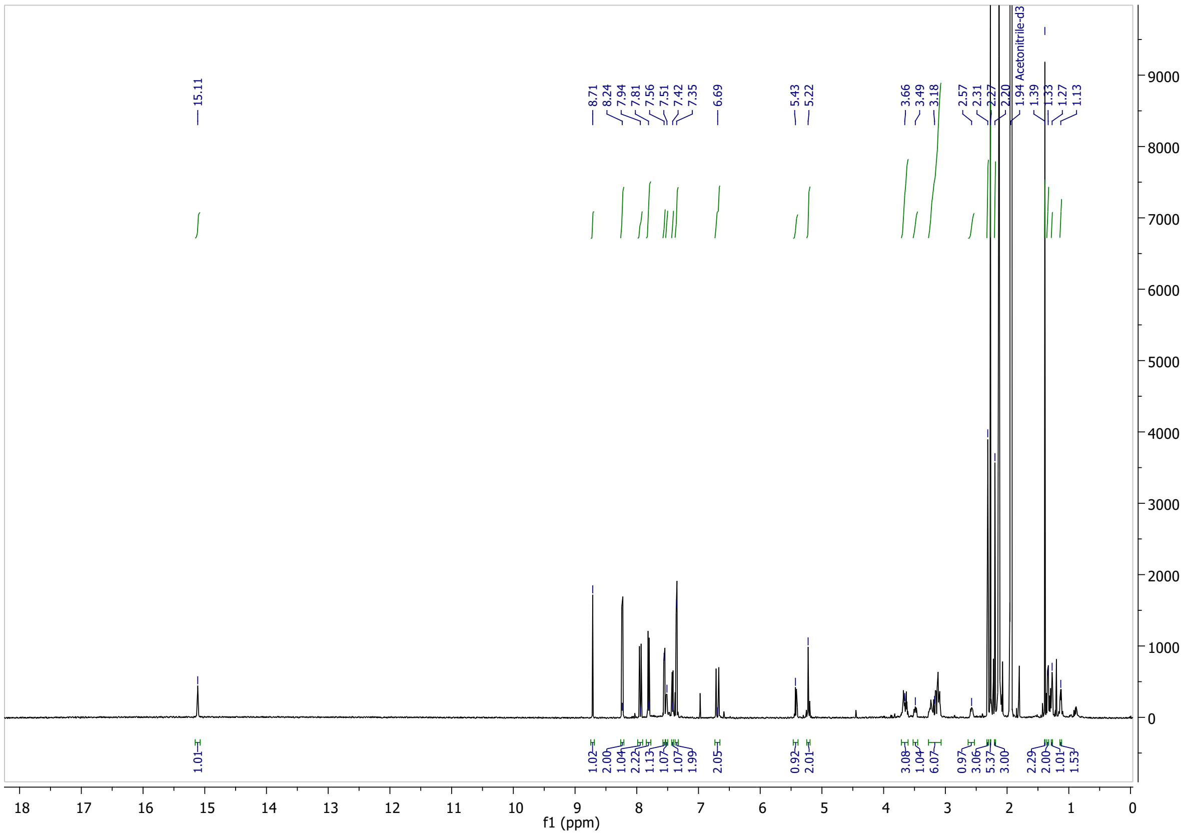
15.11
8.71
8.24
7.94
7.81
7.56
7.51
7.42
7.35
6.69
5.43
5.22
3.66
3.49
3.18
2.57
2.31
2.27
2.20
1.94 Acetonitrile-d3
1.39
1.33
1.27
1.13
f1 (ppm)

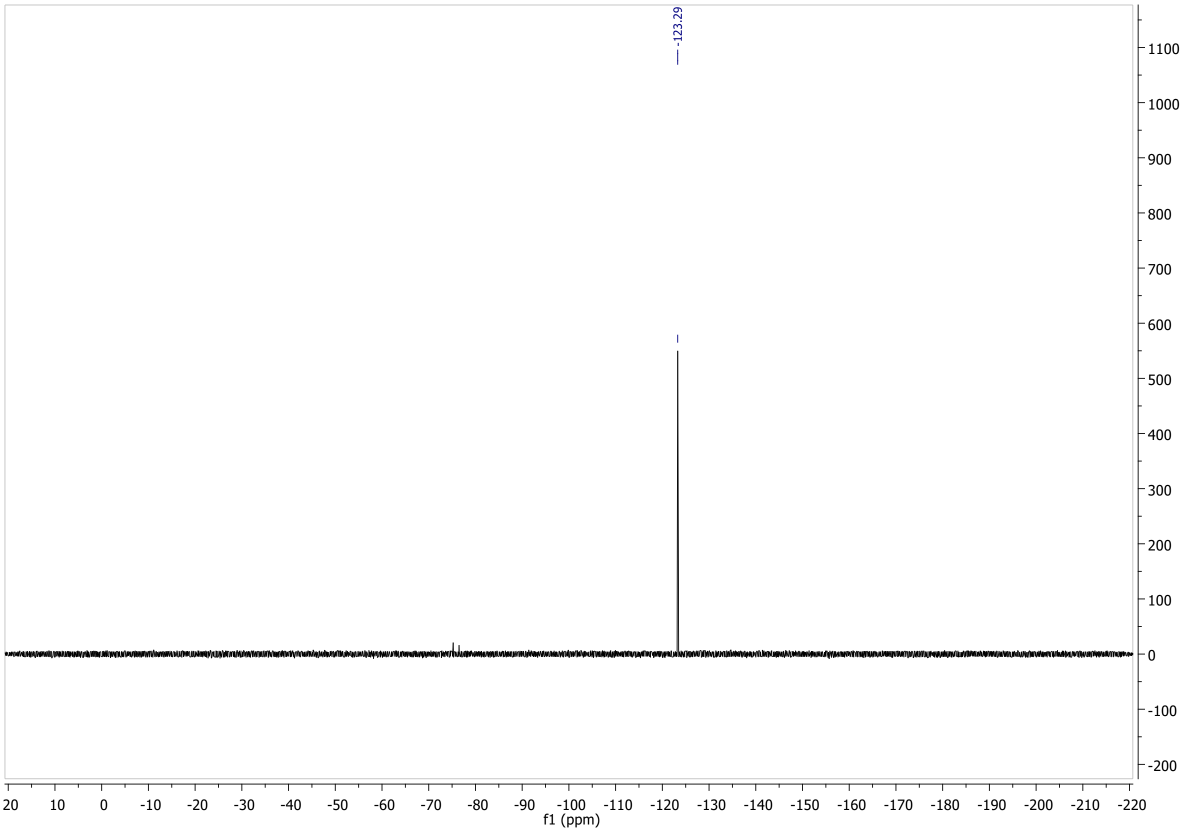
-123.29
f1 (ppm)

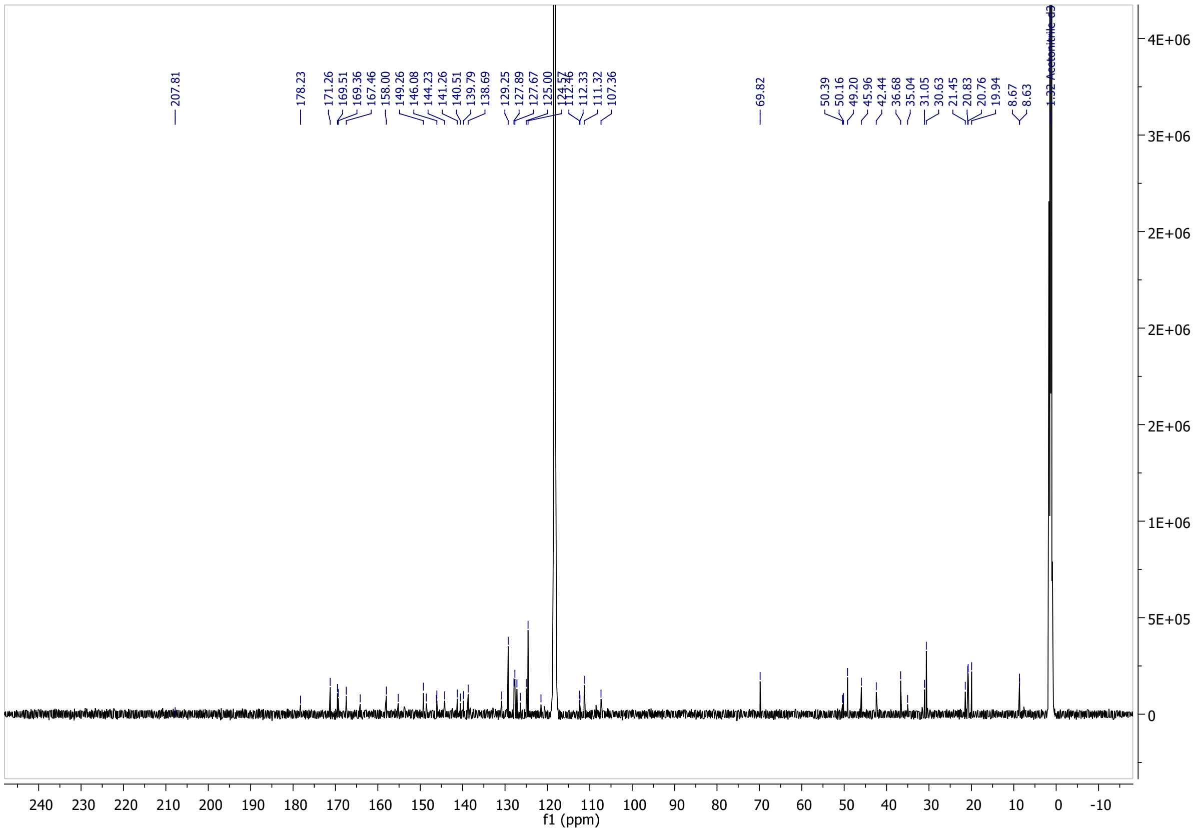
207.81
178.23
171.26
169.51
169.36
167.46
158.00
149.26
146.08
144.23
141.26
140.51
139.79
138.69
129.25
127.89
127.67
125.00
124.57
112.46
112.33
111.32
107.36
69.82
50.39
50.16
49.20
45.96
42.44
36.68
35.04
31.05
30.63
21.45
20.83
20.76
19.94
8.67
8.63
1.32 Acetonitrile-d3
4E+06
3E+06
2E+06
2E+06
2E+06
1E+06
5E+05
0
240
230
220
210
200
190
180
170
160
150
140
130
120
110
100
90
80
70
60
50
40
30
20
10
0
-10
f1 (ppm)

Compound **15**

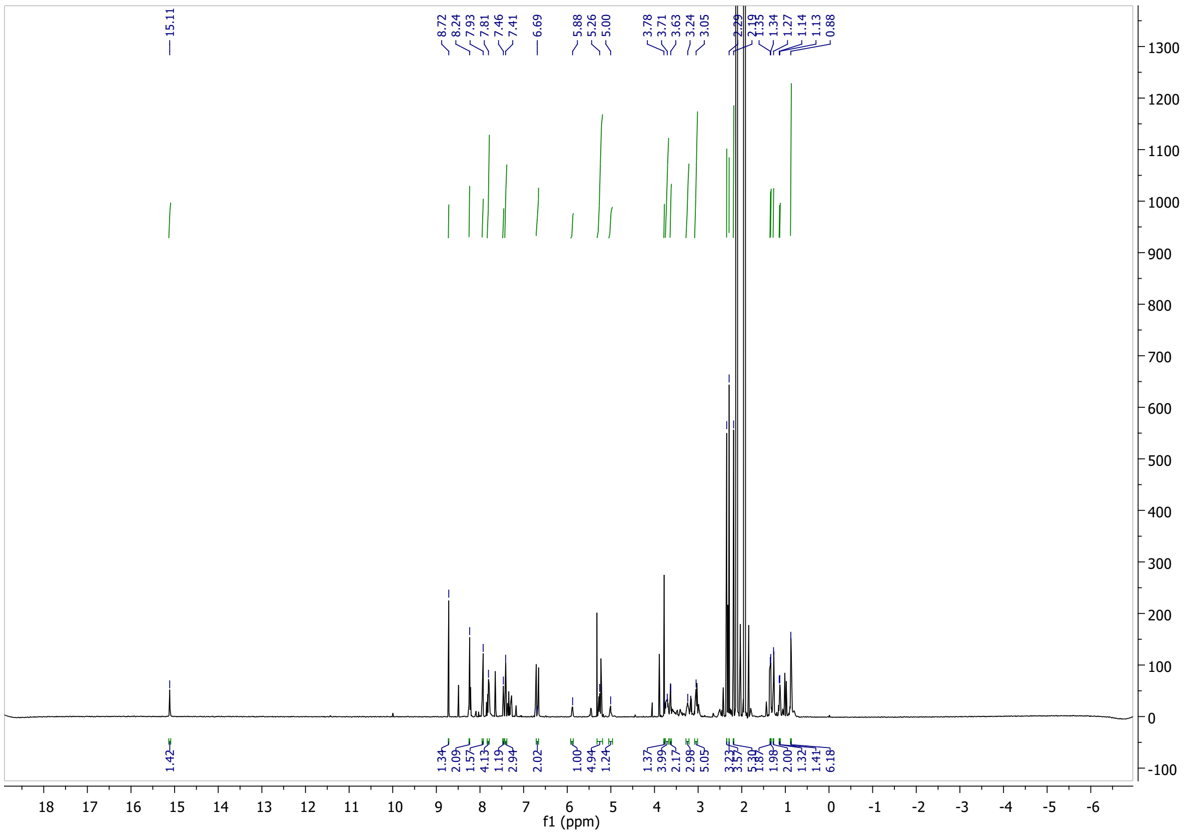
15.11
8.72
8.24
7.93
7.81
7.46
7.41
6.69
5.88
5.26
5.00
3.78
3.71
3.63
3.24
3.05
2.29
2.19
1.35
1.34
1.27
1.14
1.13
0.88
f1 (ppm)

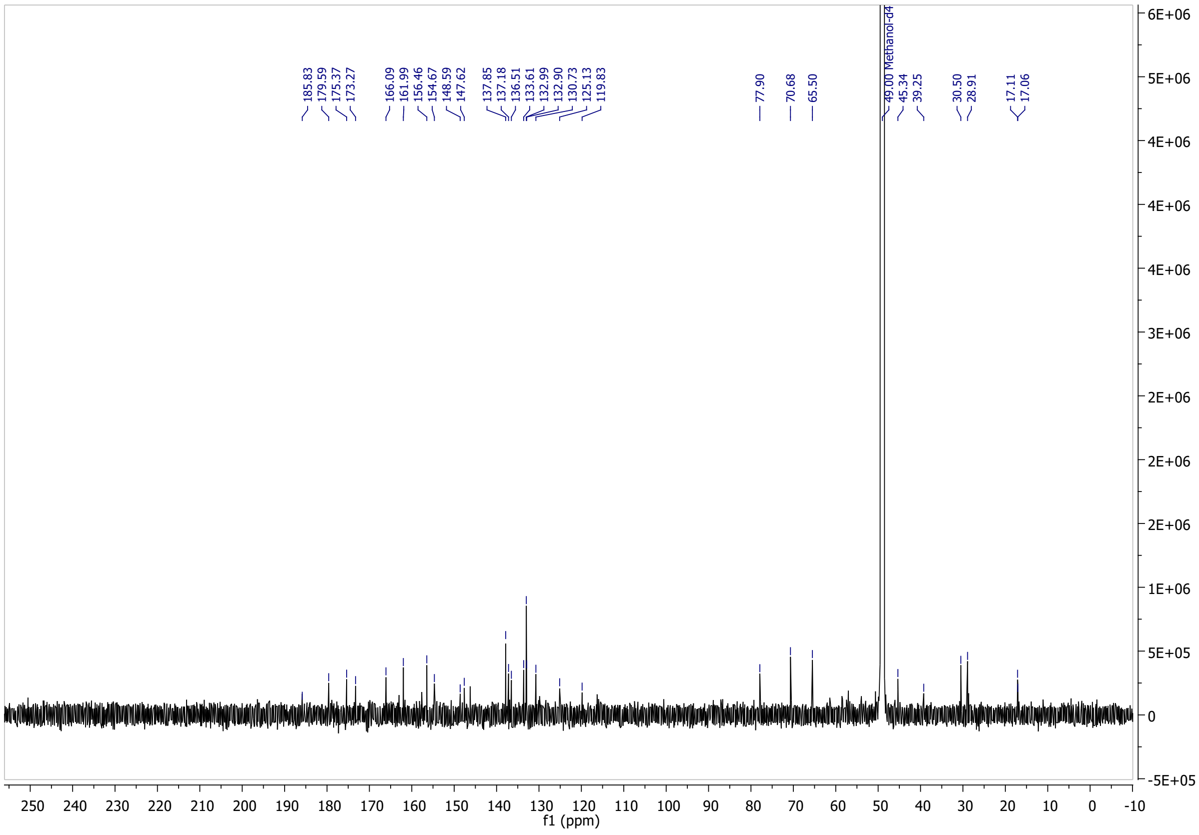
185.83
179.59
175.37
173.27
166.09
161.99
156.46
154.67
148.59
147.62
137.85
137.18
136.51
133.61
132.99
132.90
130.73
125.13
119.83
77.90
70.68
65.50
49.00 Methanol-d4
45.34
39.25
30.50
28.91
17.11
17.06
f1 (ppm)

Compound **16**

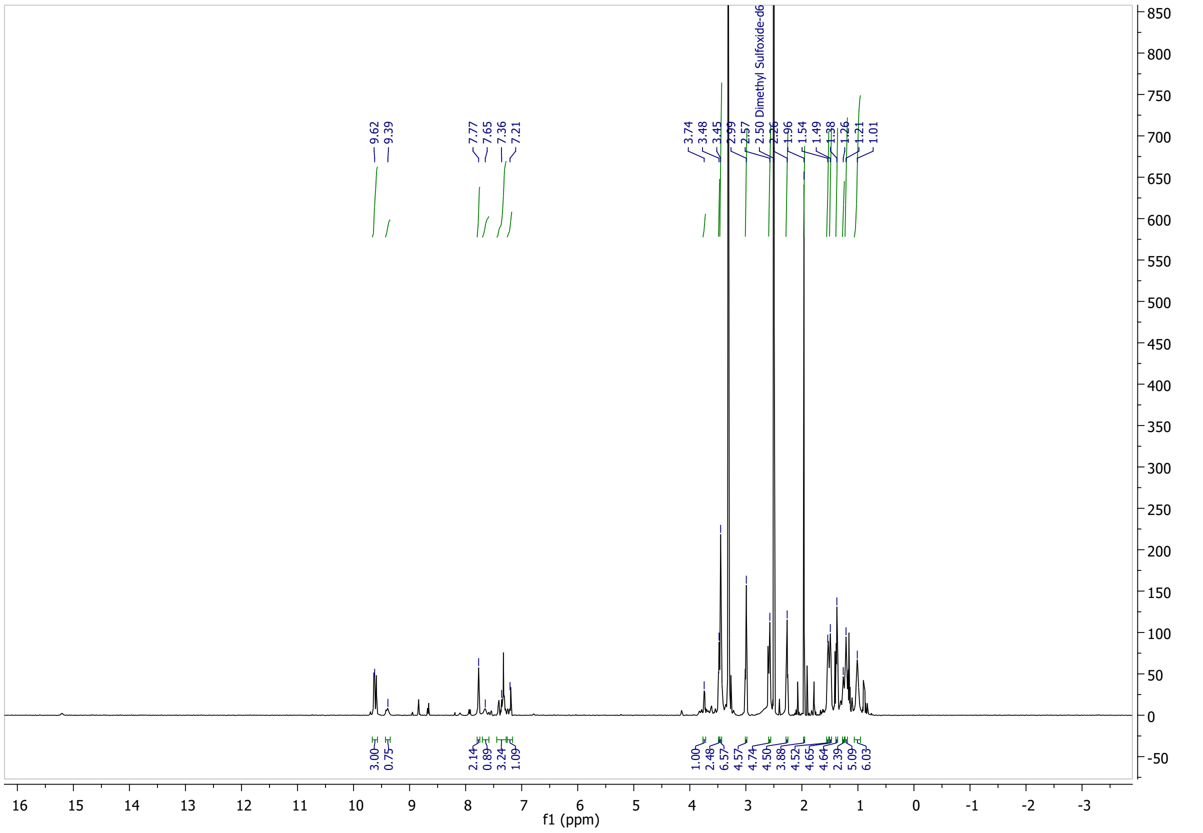
9.62
9.39
7.77
7.65
7.36
7.21
3.74
3.48
3.45
2.99
2.57
2.50 Dimethyl Sulfoxide-d6
2.26
1.96
1.54
1.49
1.38
1.26
1.21
1.01
3.00
0.75
2.14
0.89
3.24
1.09
1.00
2.48
6.57
4.57
4.74
4.50
3.88
4.52
4.65
4.64
2.39
5.09
6.03
f1 (ppm)

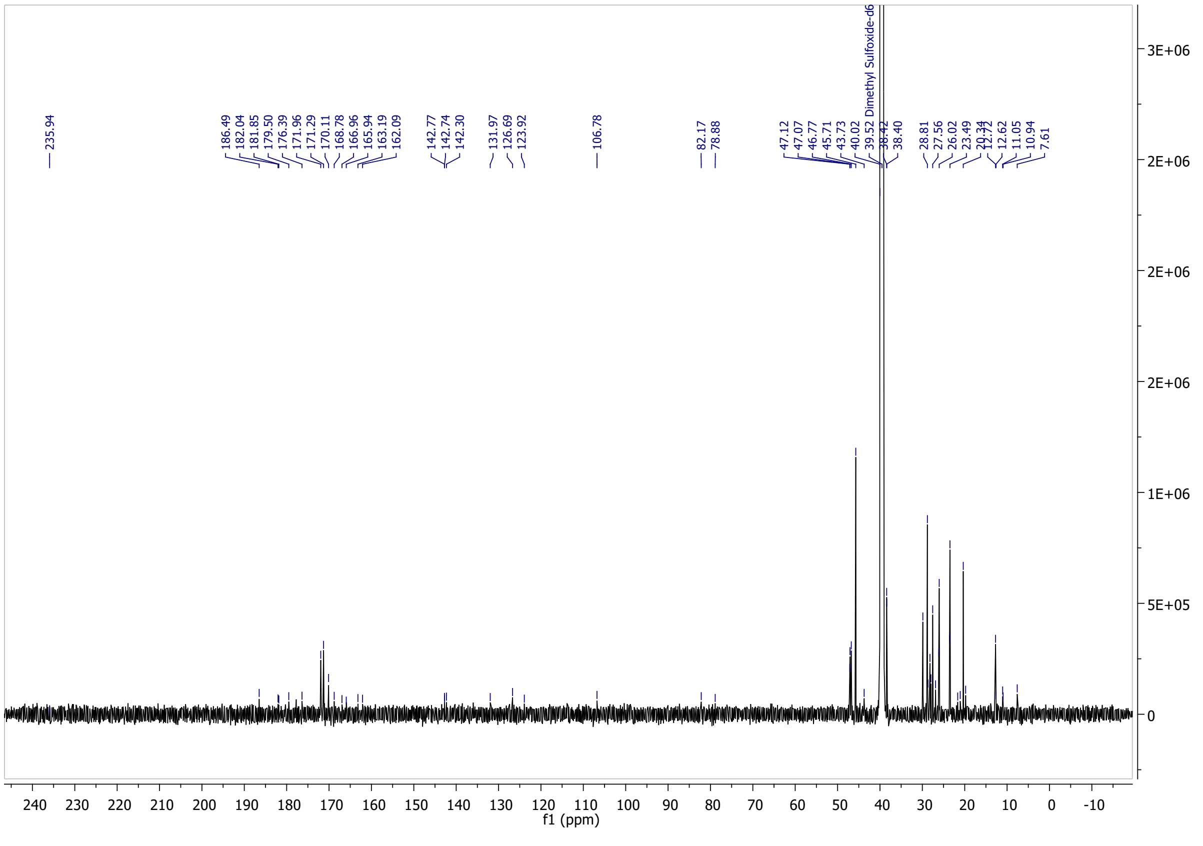
235.94
186.49
182.04
181.85
179.50
176.39
171.96
171.29
170.11
168.78
166.96
165.94
163.19
162.09
142.77
142.74
142.30
131.97
126.69
123.92
106.78
82.17
78.88
47.12
47.07
46.77
45.71
43.73
40.02
39.52 Dimethyl Sulfoxide-d6
38.42
38.40
28.81
27.56
26.02
23.49
20.34
12.72
12.62
11.05
10.94
7.61
f1 (ppm)

Compound **61a**

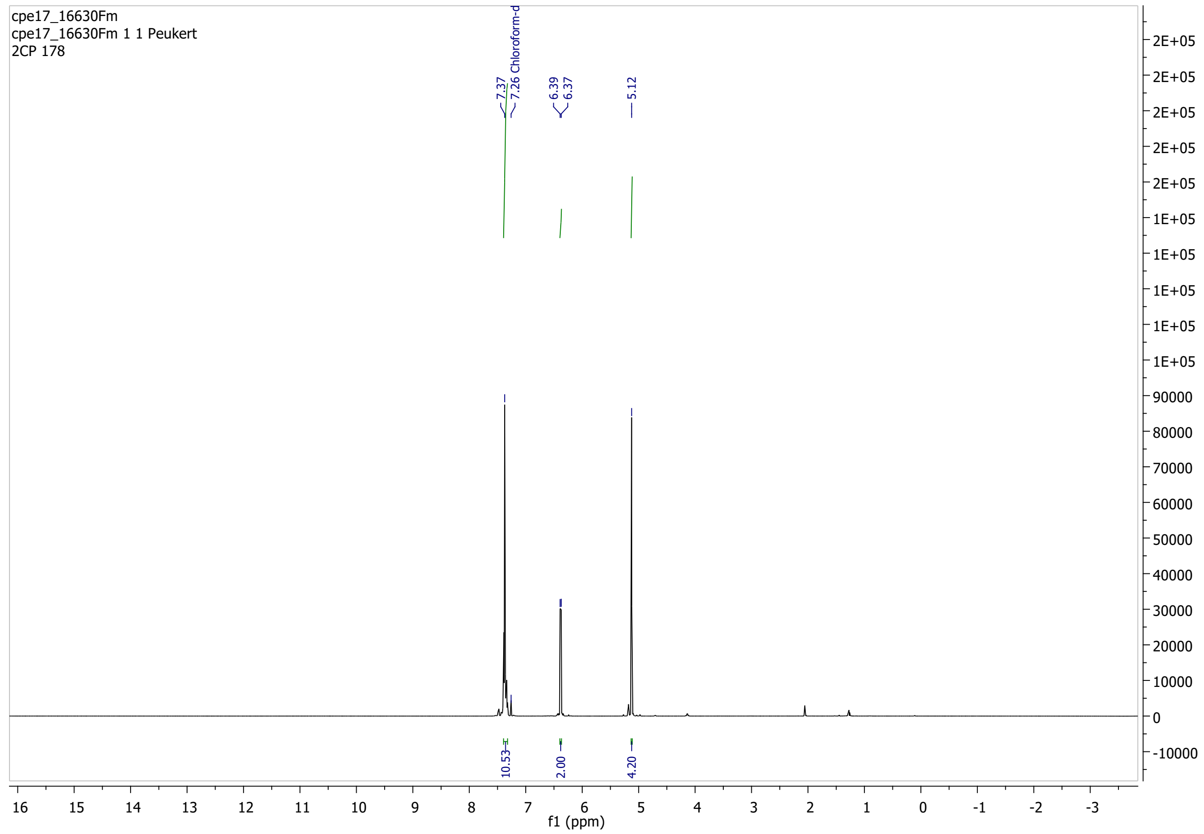

Compound **61a**

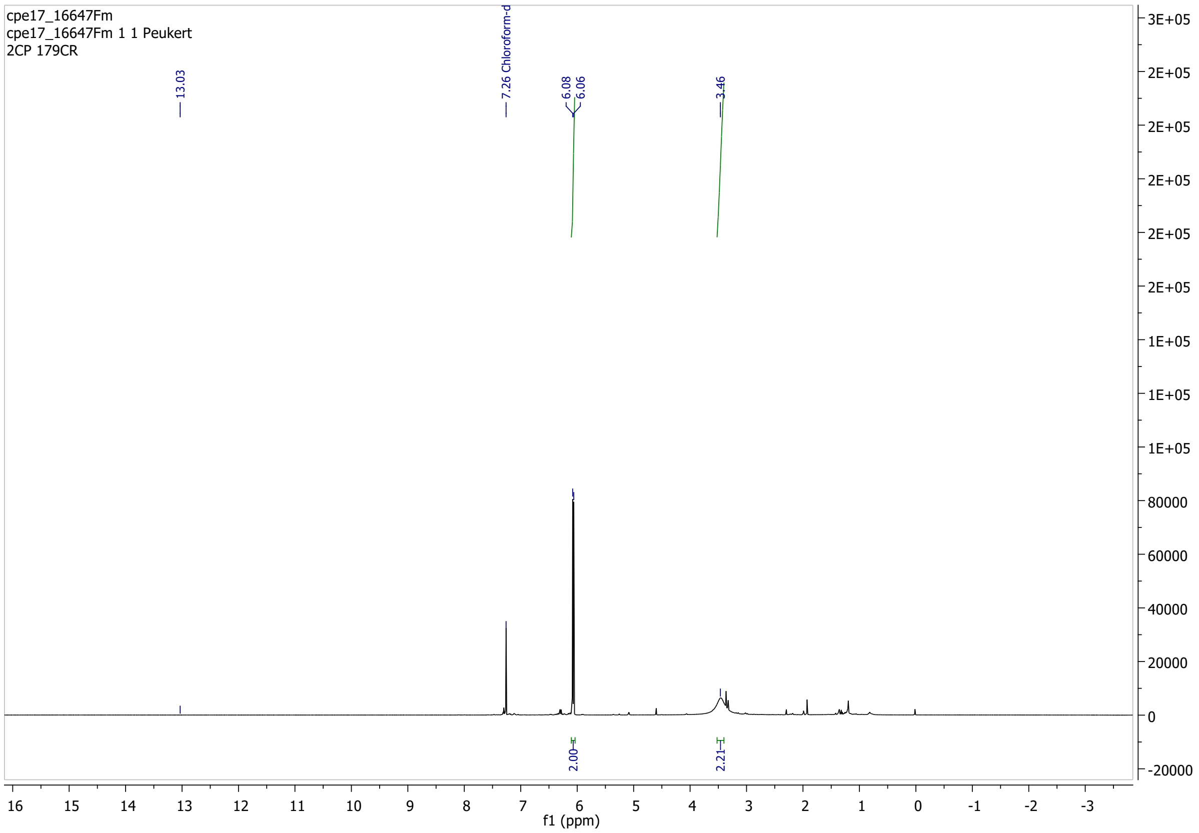

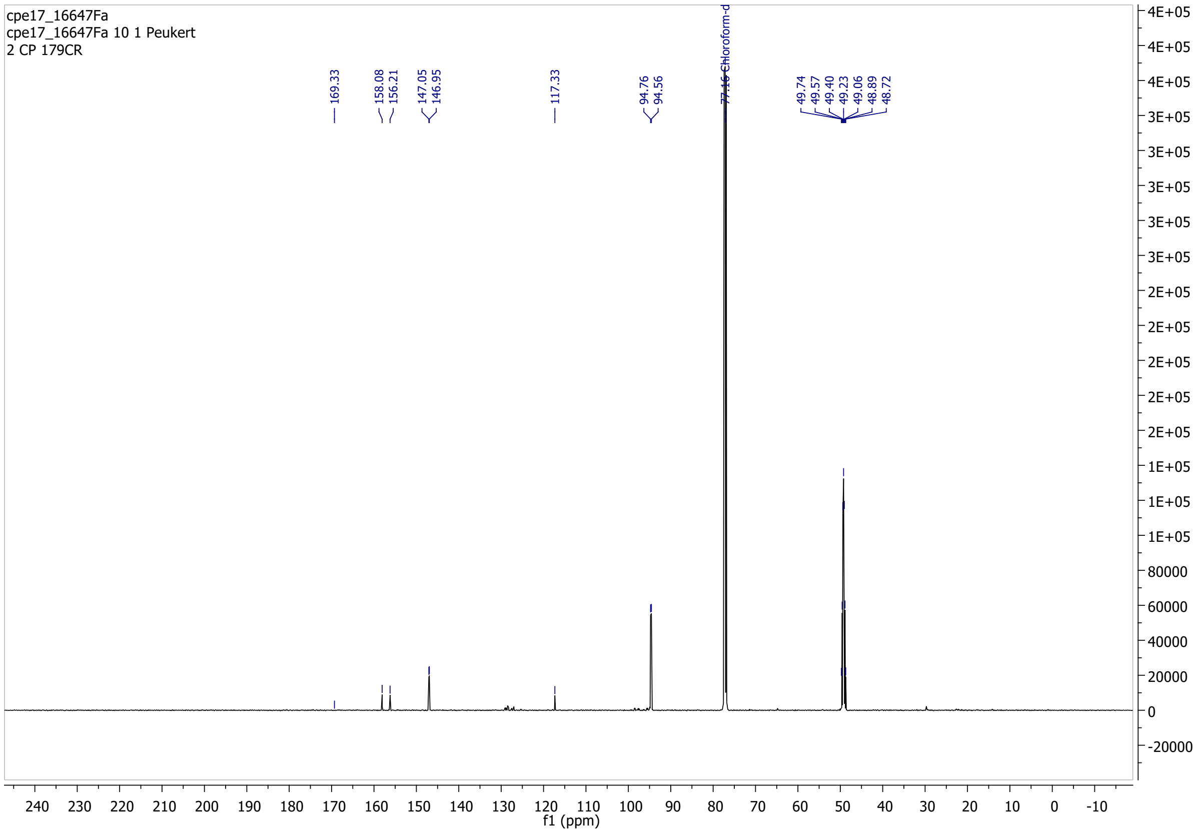

Compound **2**

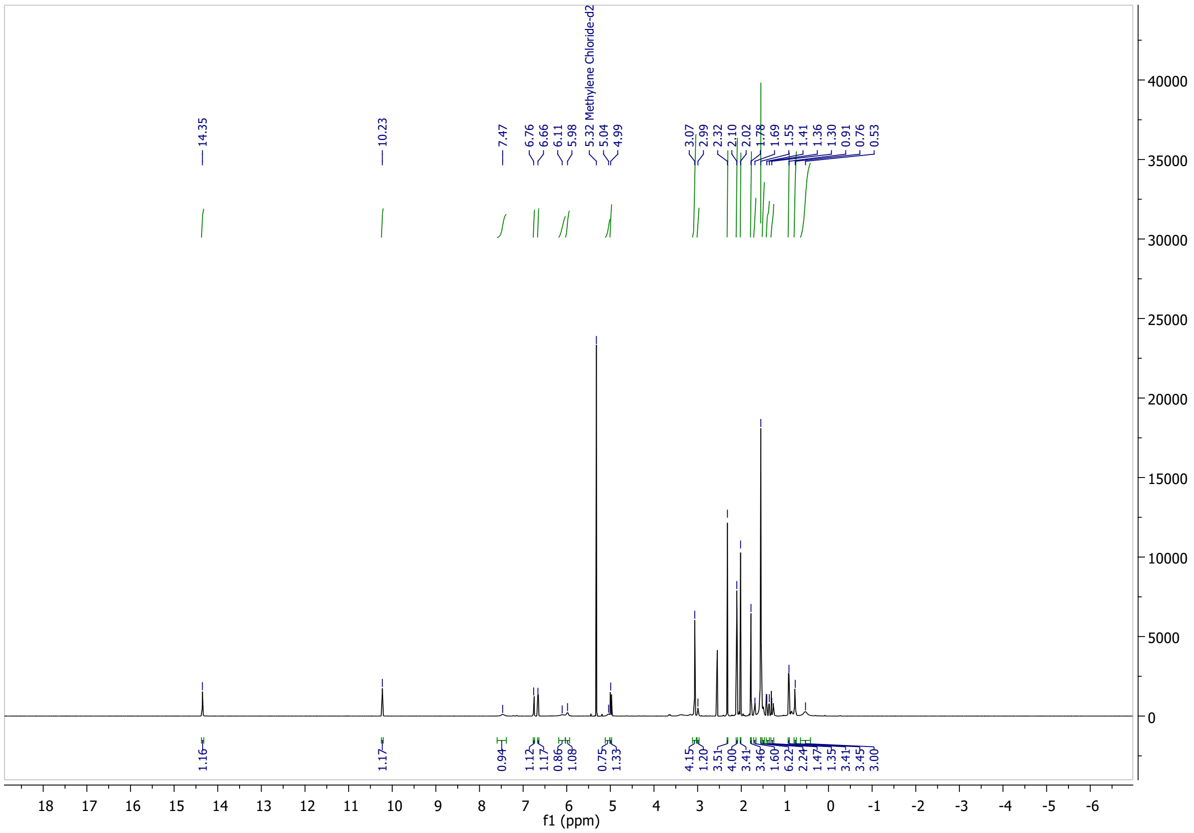
14.35
10.23
7.47
6.76
6.66
6.11
5.98
5.32 Methylene Chloride-d2
5.04
4.99
3.07
2.99
2.32
2.10
2.02
1.78
1.69
1.55
1.41
1.36
1.30
0.91
0.76
0.53
1.16
1.17
0.94
1.12
1.17
0.86
1.08
0.75
1.33
4.15
1.20
3.51
4.00
3.41
3.46
1.60
6.22
2.24
1.47
1.35
3.41
3.45
3.00
f1 (ppm)

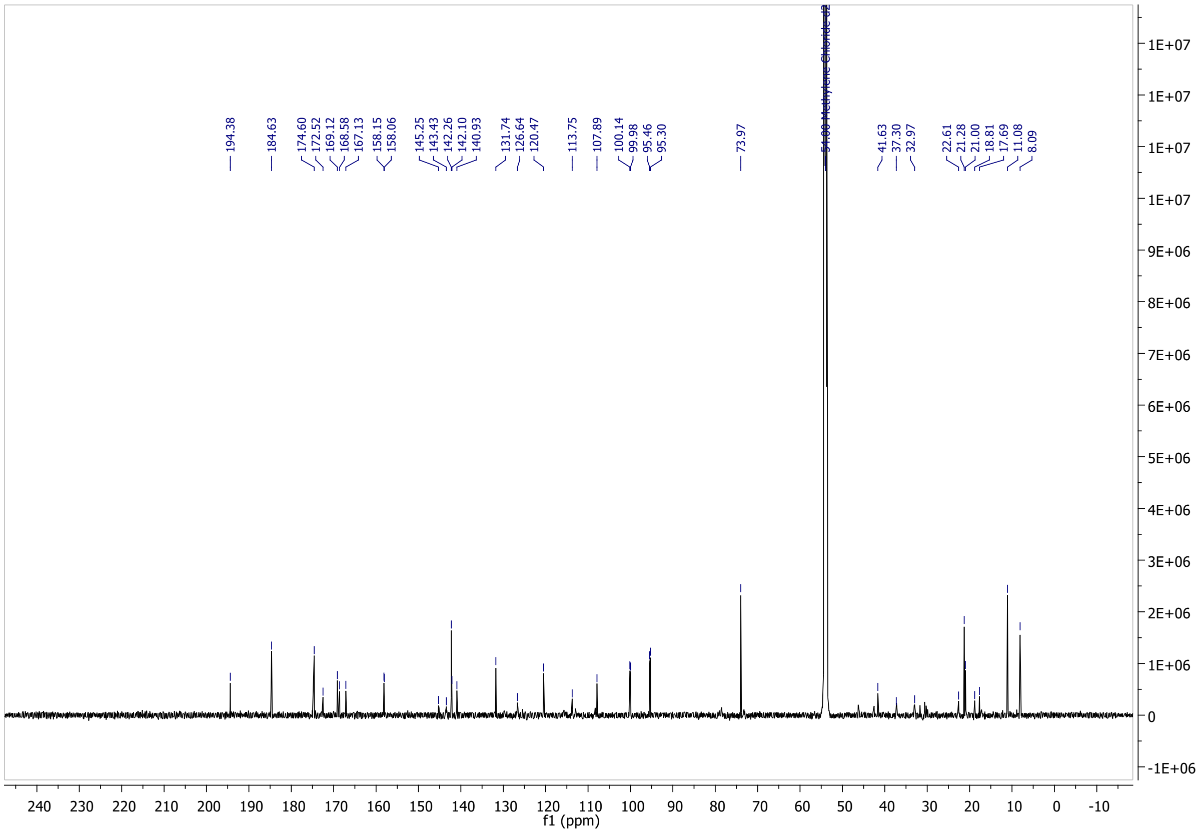
194.38
184.63
174.60
172.52
169.12
168.58
167.13
158.15
158.06
145.25
143.43
142.26
142.10
140.93
131.74
126.64
120.47
113.75
107.89
100.14
99.98
95.46
95.30
73.97
54.00 Methylene Chloride-d2
41.63
37.30
32.97
22.61
21.28
21.00
18.81
17.69
11.08
8.09
f1 (ppm)

Compound **22**

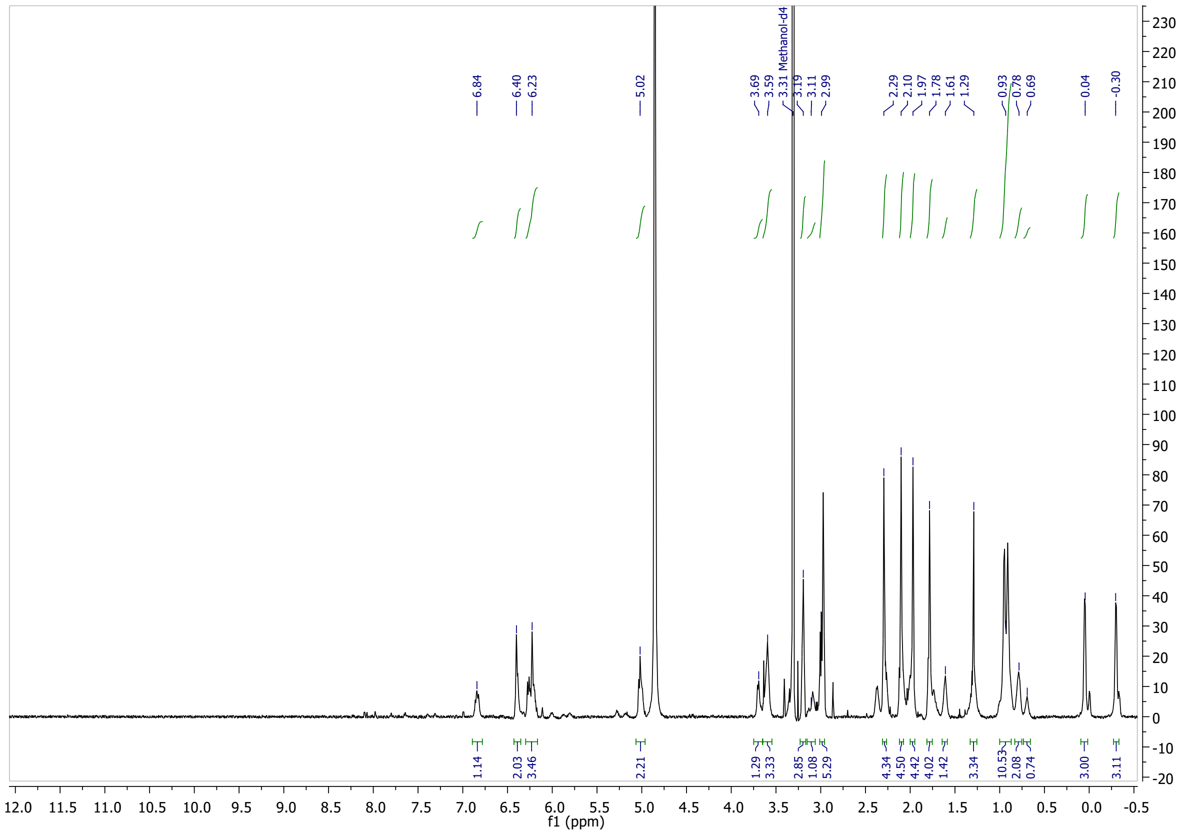
6.84
6.40
6.23
5.02
3.69
3.59
3.31 Methanol-d4
3.19
3.11
2.99
2.29
2.10
1.97
1.78
1.61
1.29
0.93
0.78
0.69
0.04
-0.30
1.14
2.03
3.46
2.21
1.29
3.33
2.85
1.08
5.29
4.34
4.50
4.42
4.02
1.42
3.34
10.53
2.08
0.74
3.00
3.11
f1 (ppm)

Compound **27**

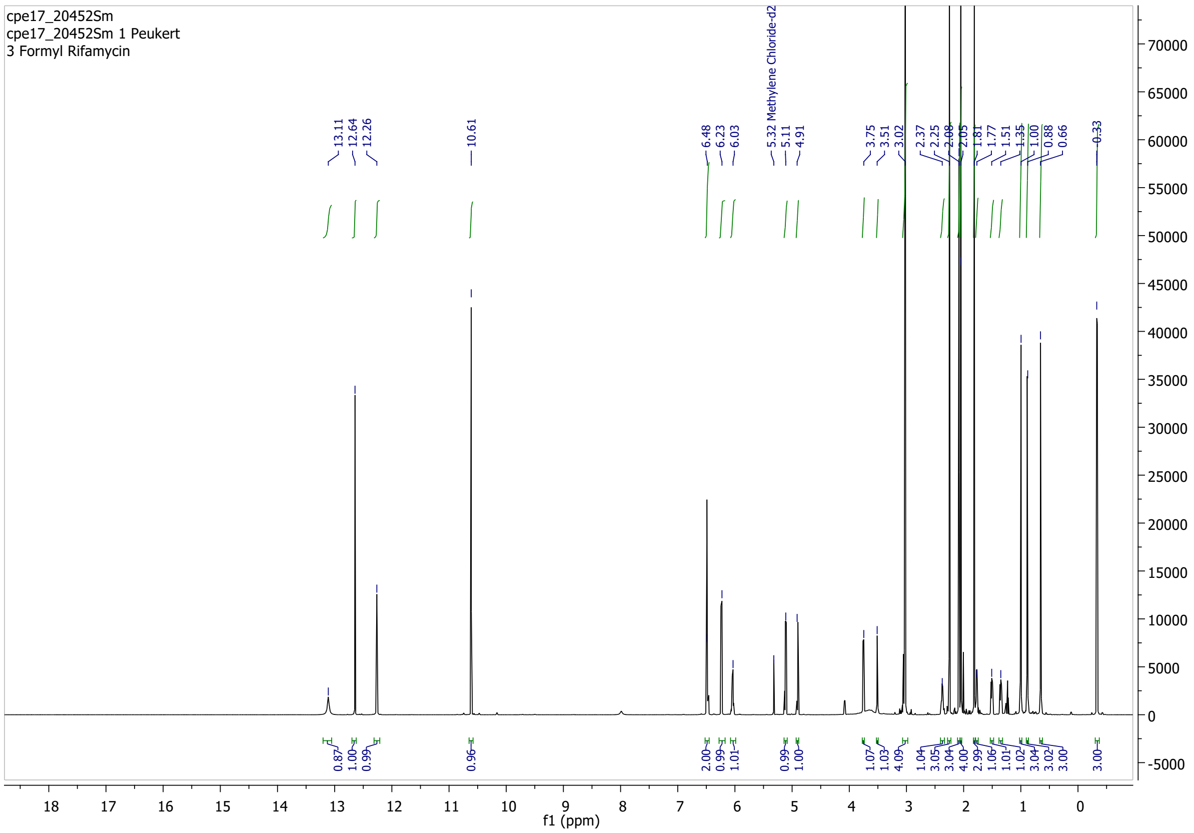

Compound 62

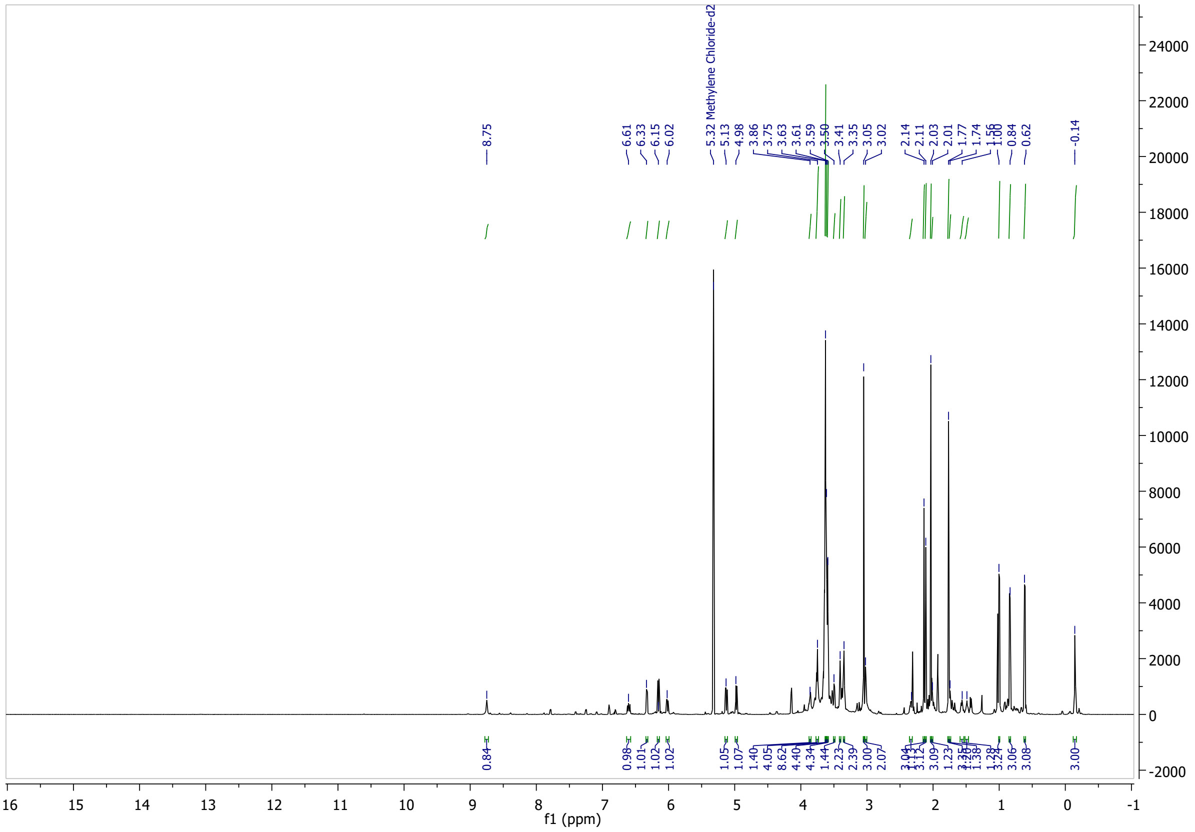

Compound **28**

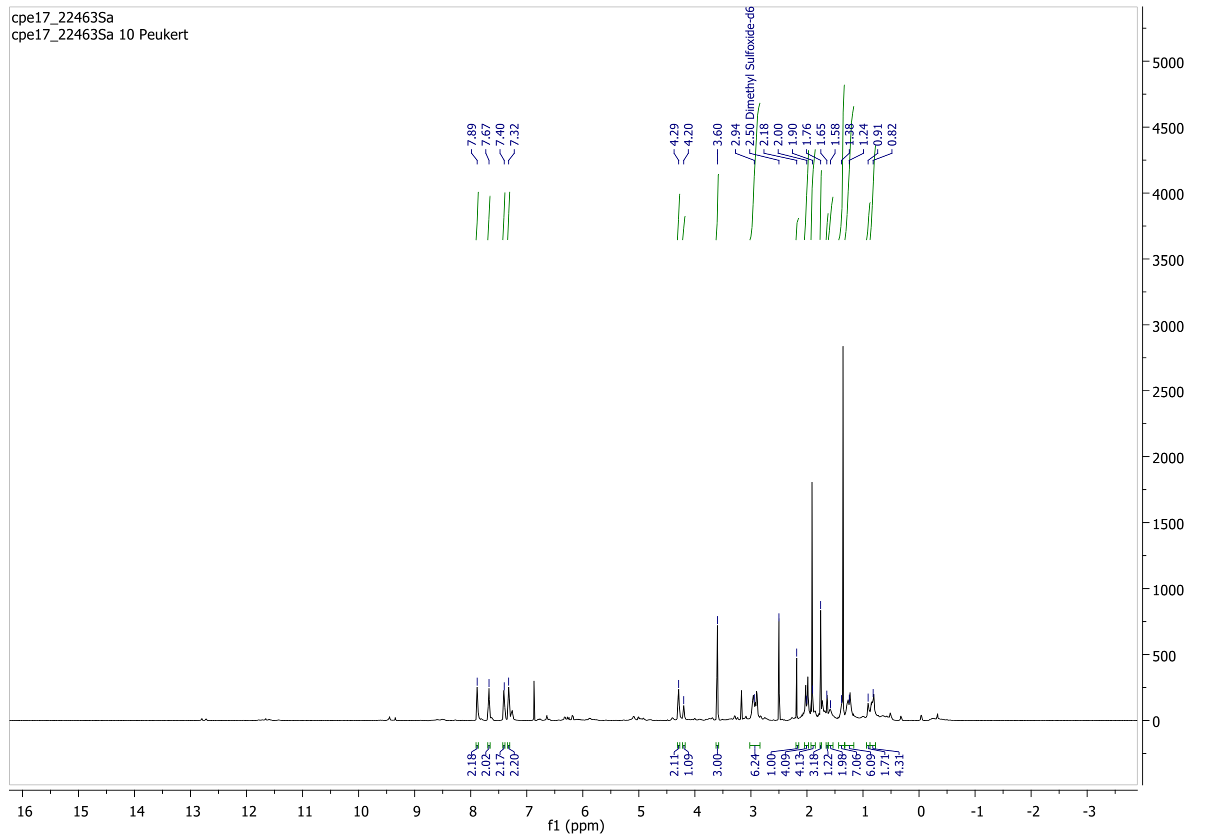

cpe17_22463Sa
cpe17_22463Sa 10 Peukert
7.89
7.67
7.40
7.32
4.29
4.20
3.60
2.94
2.50 Dimethyl Sulfoxide-d6
2.18
2.00
1.90
1.76
1.65
1.58
1.38
1.24
0.91
0.82
2.18
2.02
2.17
2.20
2.11
1.09
3.00
6.24
1.00
4.09
4.13
3.18
1.22
1.98
7.06
6.09
1.71
4.31
f1 (ppm)

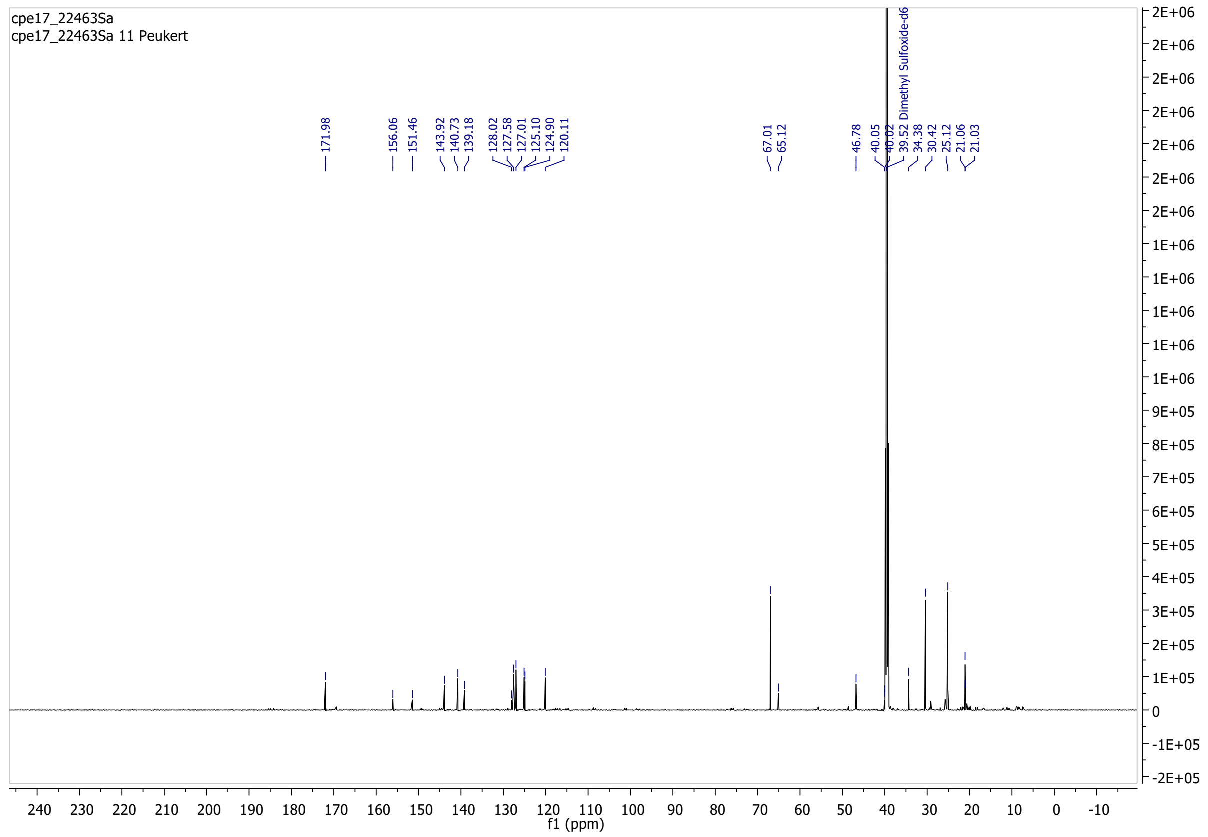

cpe17_22463Sa
cpe17_22463Sa 11 Peukert
171.98
156.06
151.46
143.92
140.73
139.18
128.02
127.58
127.01
125.10
124.90
120.11
67.01
65.12
46.78
40.05
40.02
39.52 Dimethyl Sulfoxide-d6
34.38
30.42
25.12
21.06
21.03
f1 (ppm)

Compound **65**

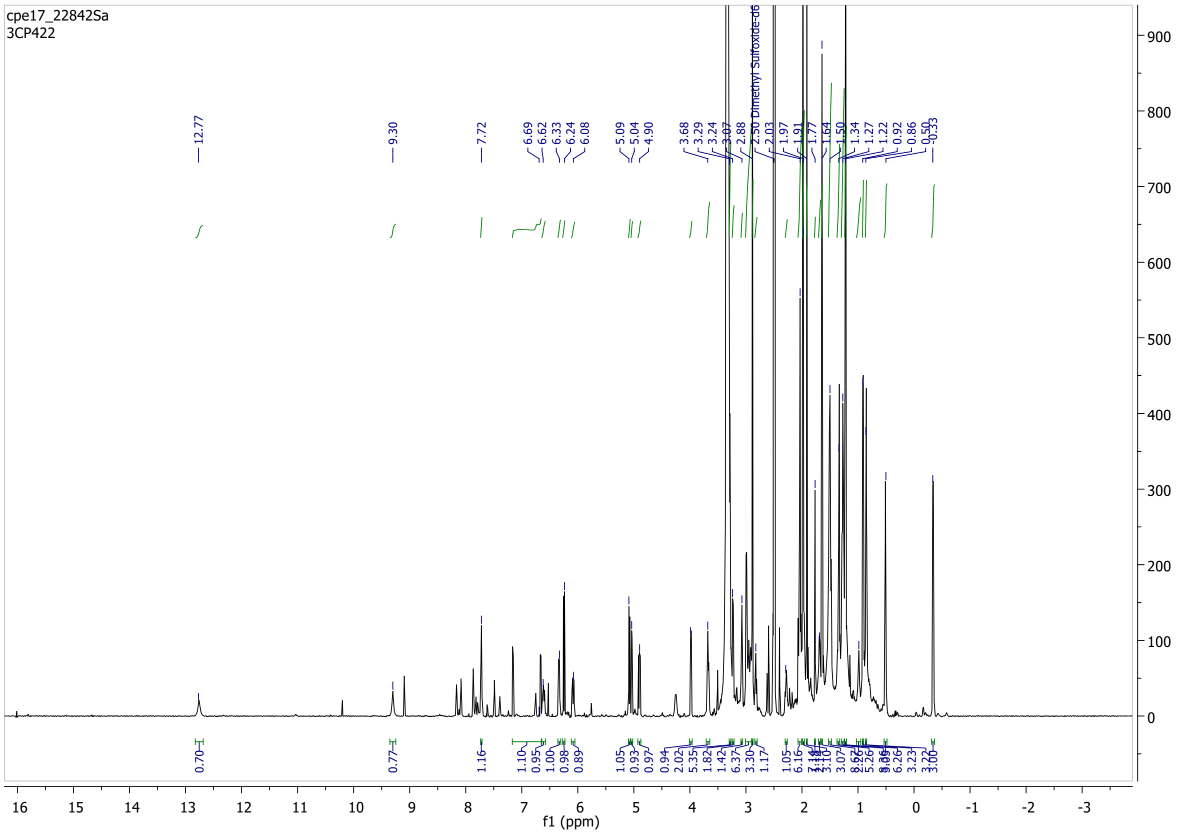

cpe17_22842Sa
3CP422
12.77
9.30
7.72
6.69
6.62
6.33
6.24
6.08
5.09
5.04
4.90
3.68
3.29
3.24
3.07
2.88
2.50 Dimethyl Sulfoxide-d6
2.03
1.97
1.91
1.77
1.64
1.50
1.34
1.27
1.22
0.92
0.86
0.50
-0.33
f1 (ppm)

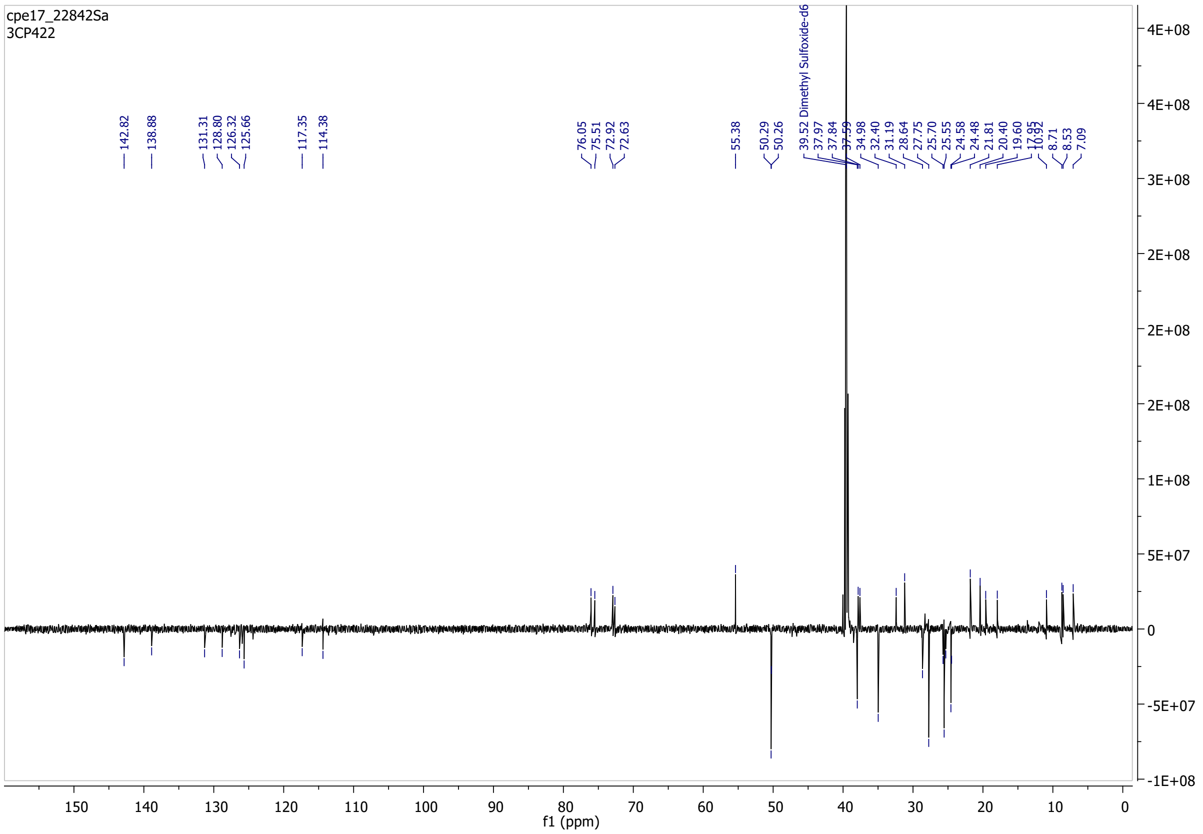

cpe17_22842Sa
3CP422
142.82
138.88
131.31
128.80
126.32
125.66
117.35
114.38
76.05
75.51
72.92
72.63
55.38
50.29
50.26
39.52 Dimethyl Sulfoxide-d6
37.97
37.84
34.98
32.40
31.19
28.64
27.75
25.70
25.55
24.58
24.48
21.81
20.40
19.60
17.95
10.92
8.71
8.53
7.09
f1 (ppm)

Compound **64**

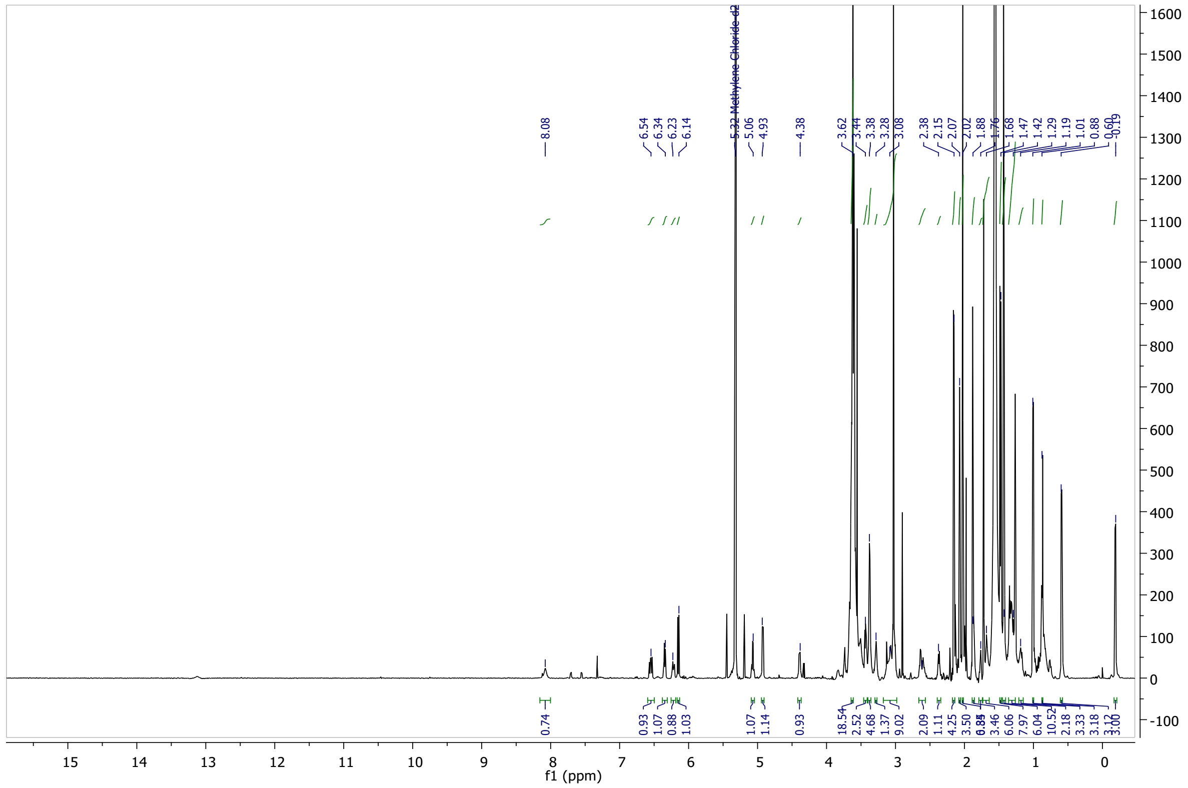

f1 (ppm)
Methylene Chloride-d2

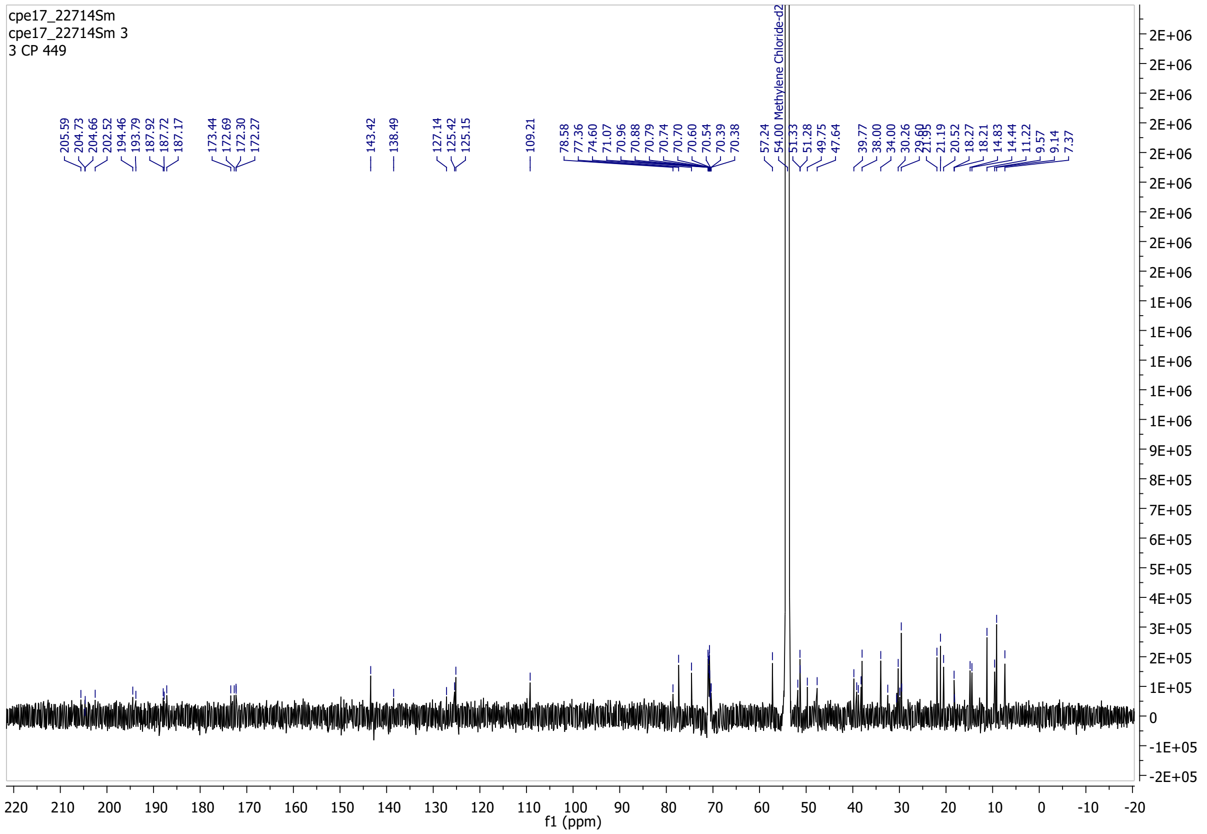

cpe17_22714Sm
cpe17_22714Sm 3
3 CP 449
Methylene Chloride-d2
f1 (ppm)

Compound **32**

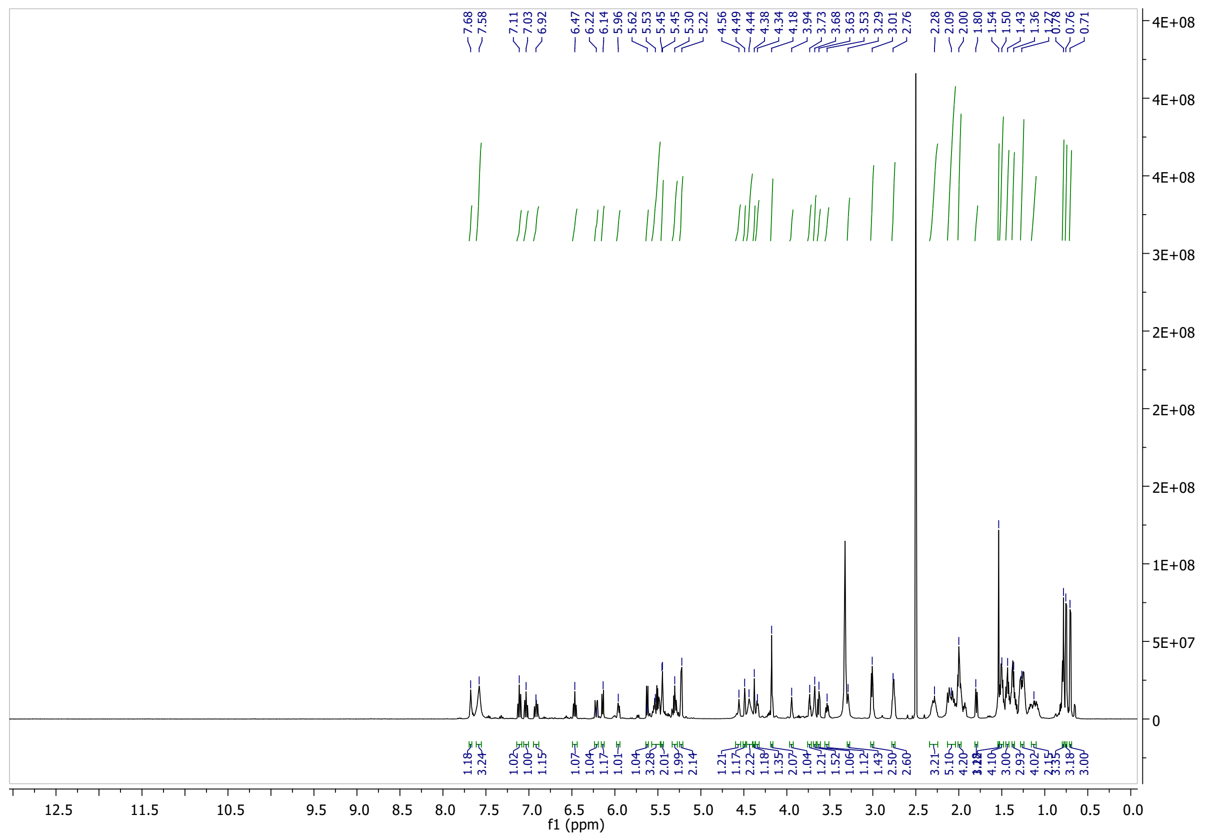

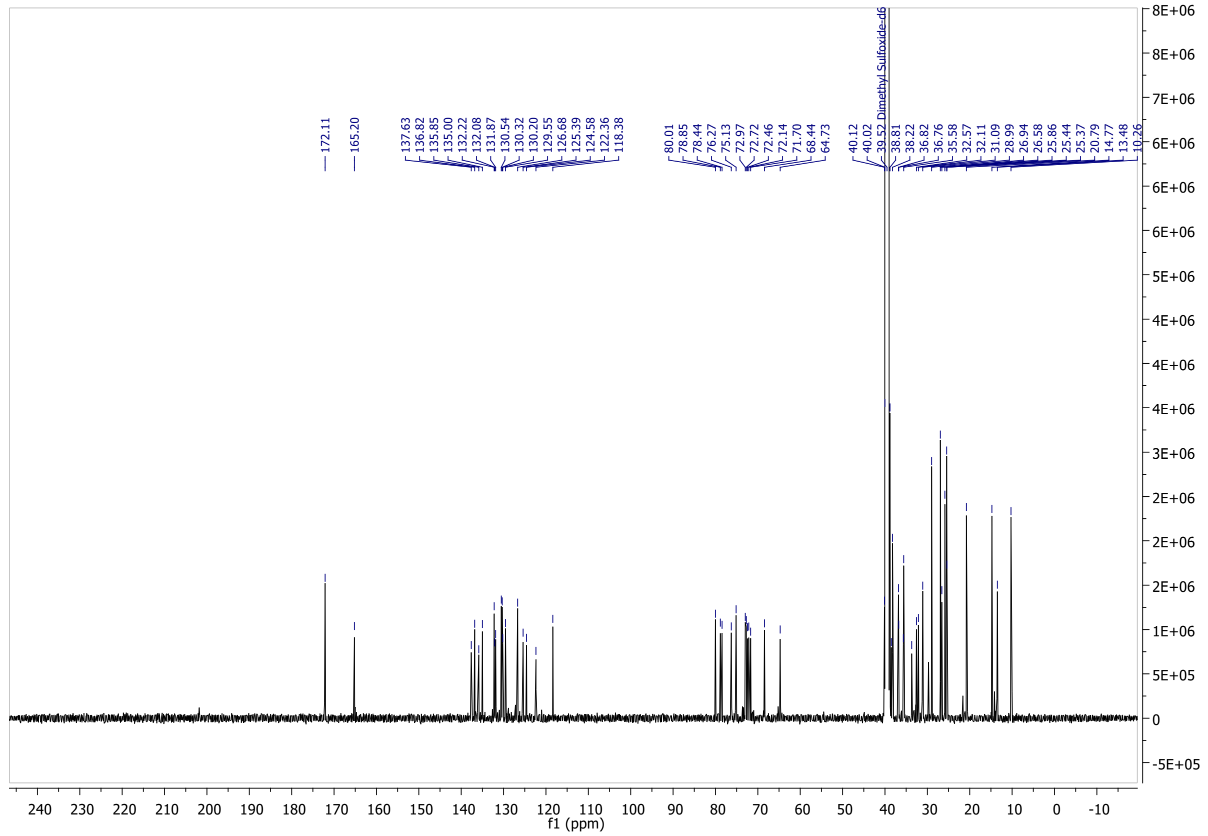

Compound **67**

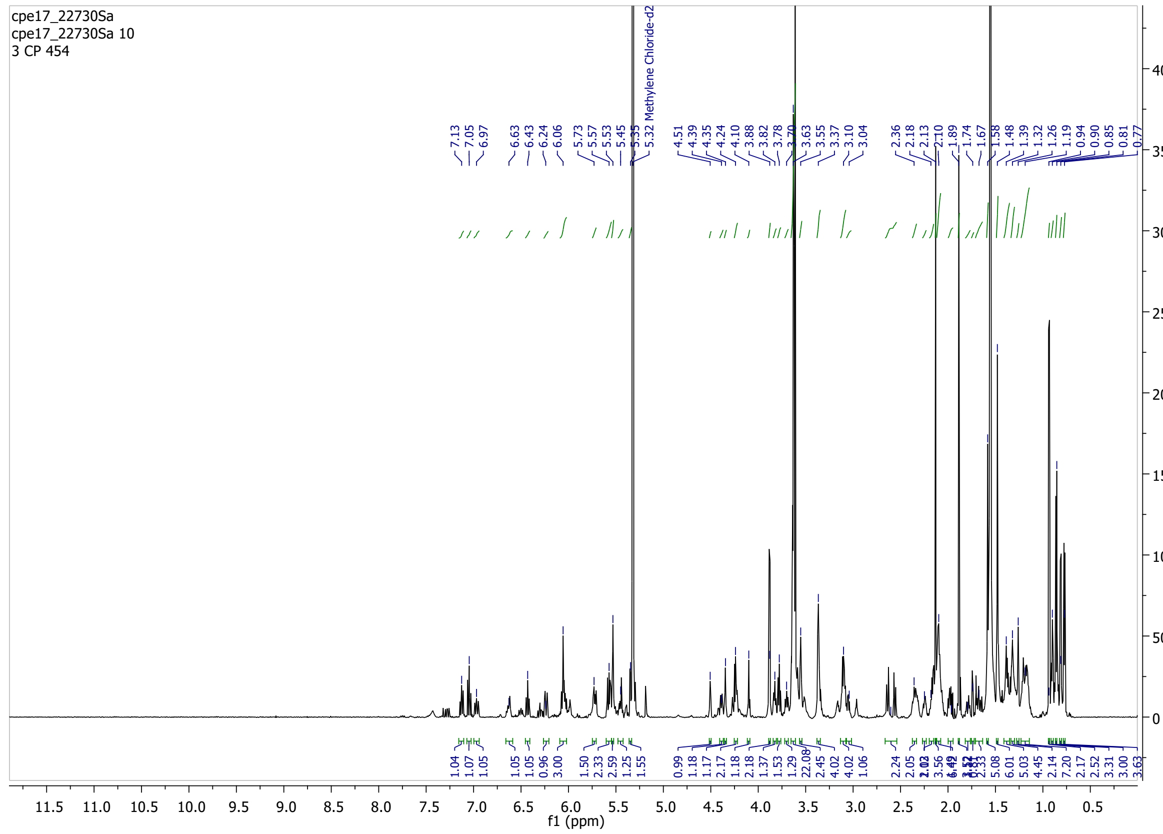

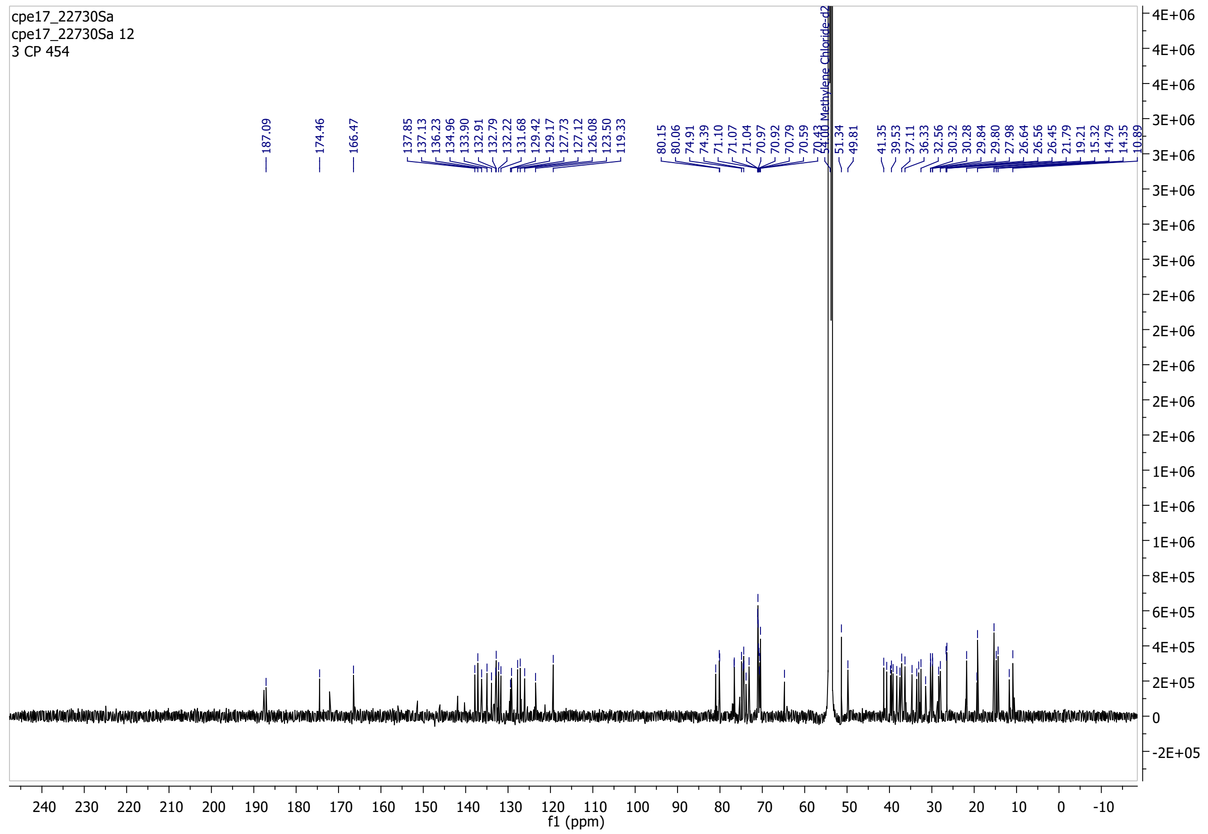

Compound **68**

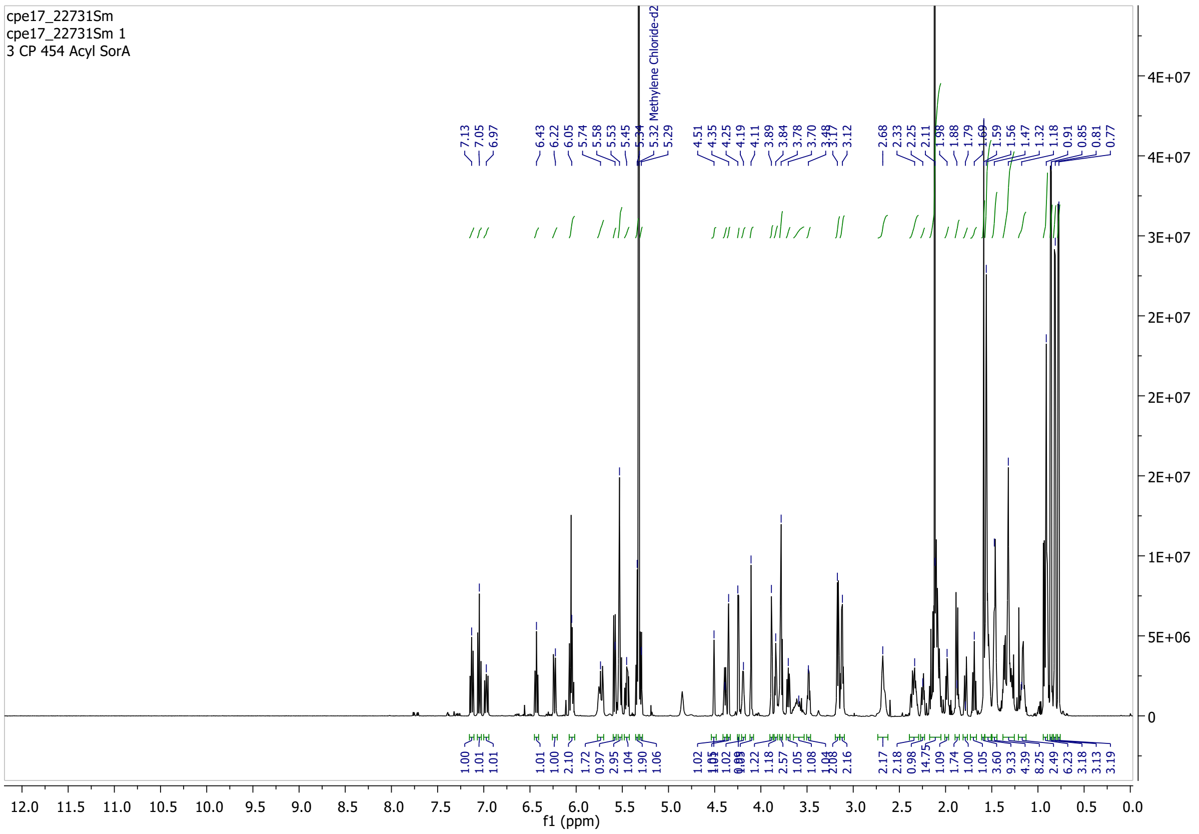

cpe17_22731Sm
cpe17_22731Sm 1
3 CP 454 Acyl SorA
Methylene Chloride-d2
f1 (ppm)

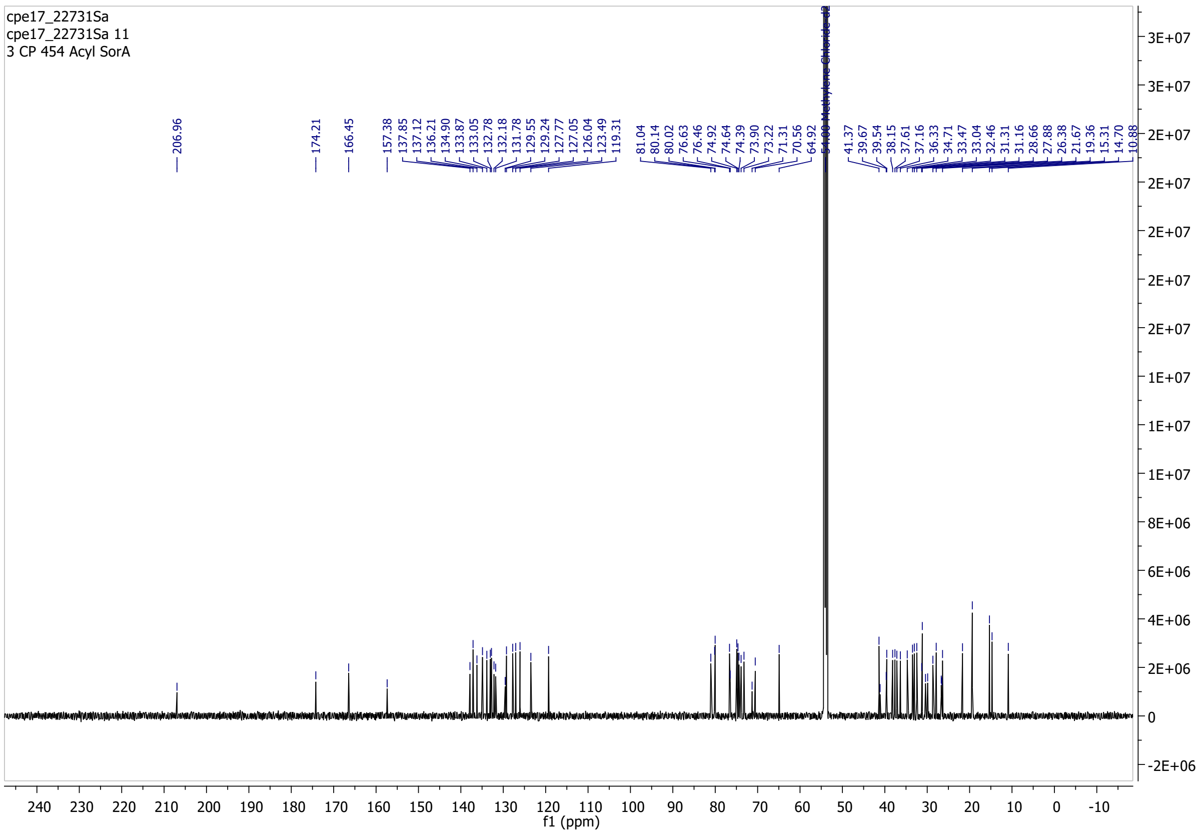

cpe17_22731Sa
cpe17_22731Sa 11
3 CP 454 Acyl SorA
Methylene Chloride-d2
f1 (ppm)

Compound **69**

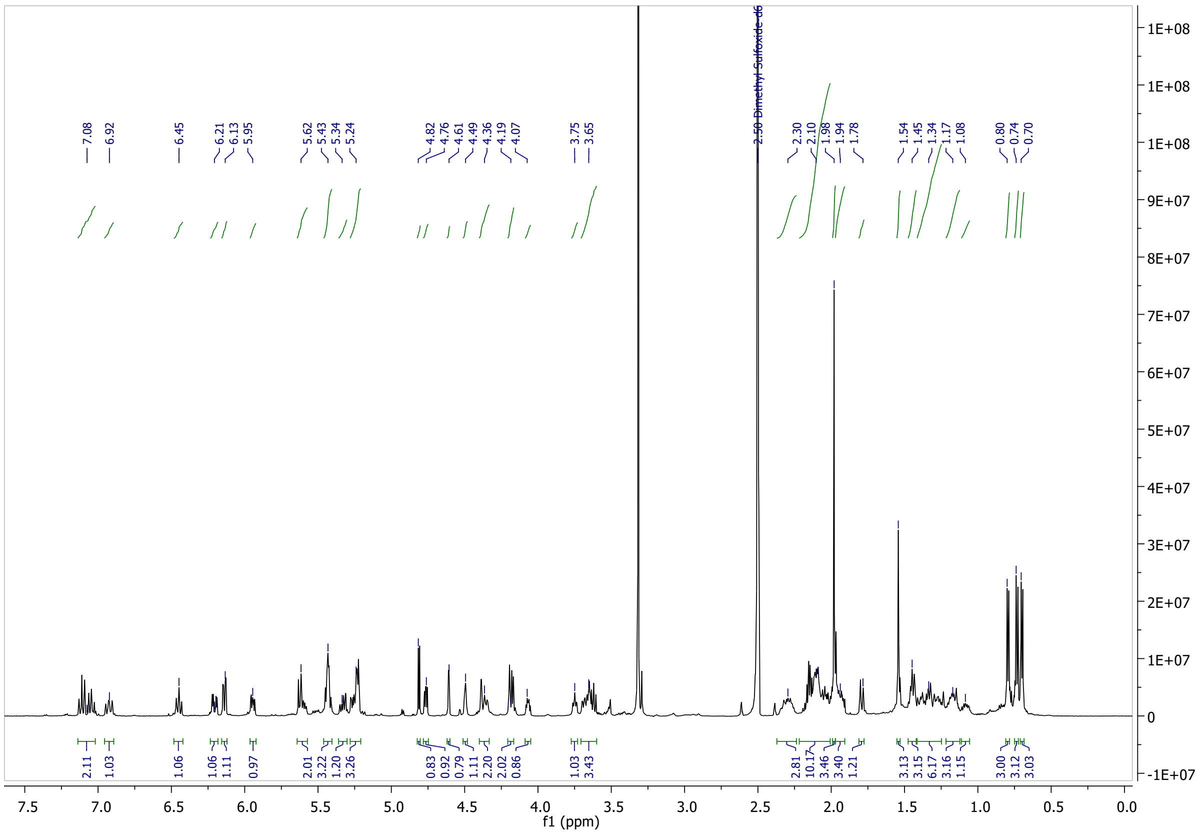

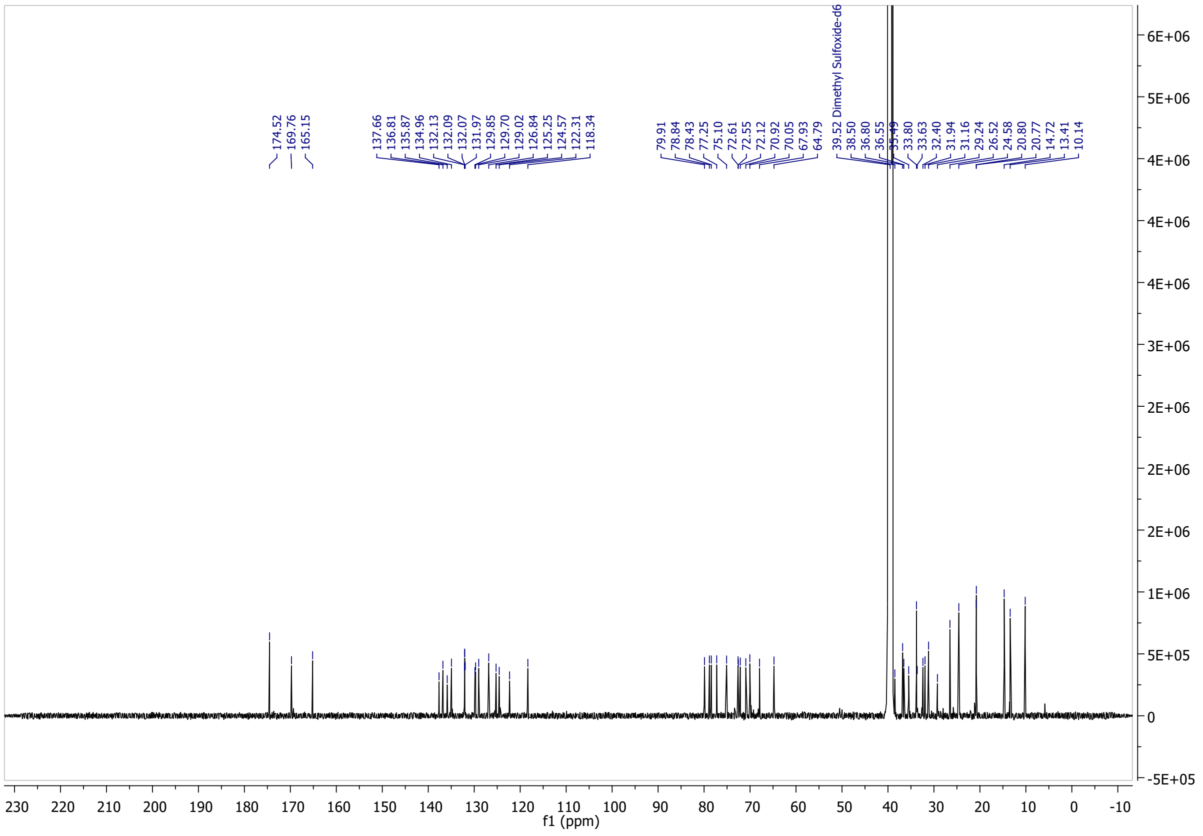

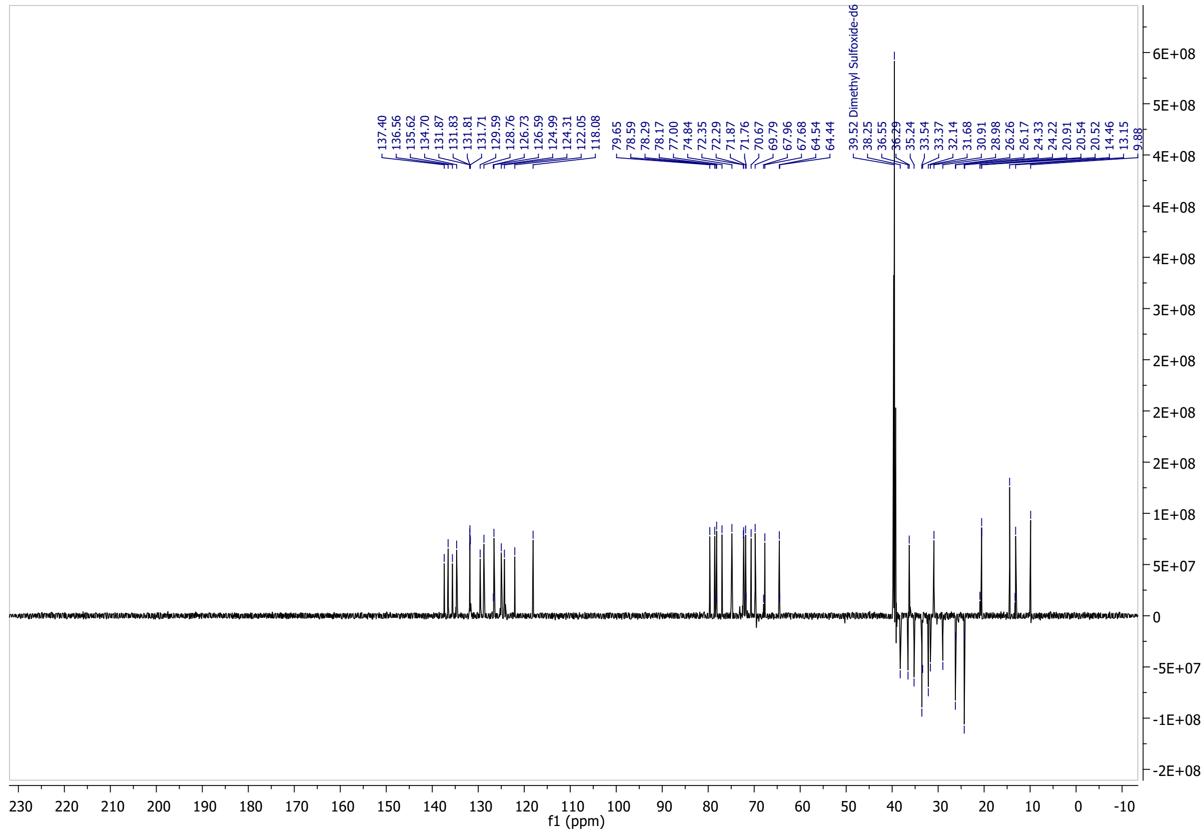

137.40
136.56
135.62
134.70
131.87
131.83
131.81
131.71
129.59
128.76
126.73
126.59
124.99
124.31
122.05
118.08
79.65
78.59
78.29
78.17
77.00
74.84
72.35
72.29
71.87
71.76
70.67
69.79
67.96
67.68
64.54
64.44
39.52 Dimethyl Sulfoxide-d6
38.25
36.55
36.29
35.24
33.54
33.37
32.14
31.68
30.91
28.98
26.26
26.17
24.33
24.22
20.91
20.54
20.52
14.46
13.15
9.88
6E+08
5E+08
4E+08
3E+08
2E+08
1E+08
5E+07
0
-5E+07
-1E+08
-2E+08
230 220 210 200 190 180 170 160 150 140 130 120 110 100 90 80 70 60 50 40 30 20 10 0 -10
f1 (ppm)

Compound **70**

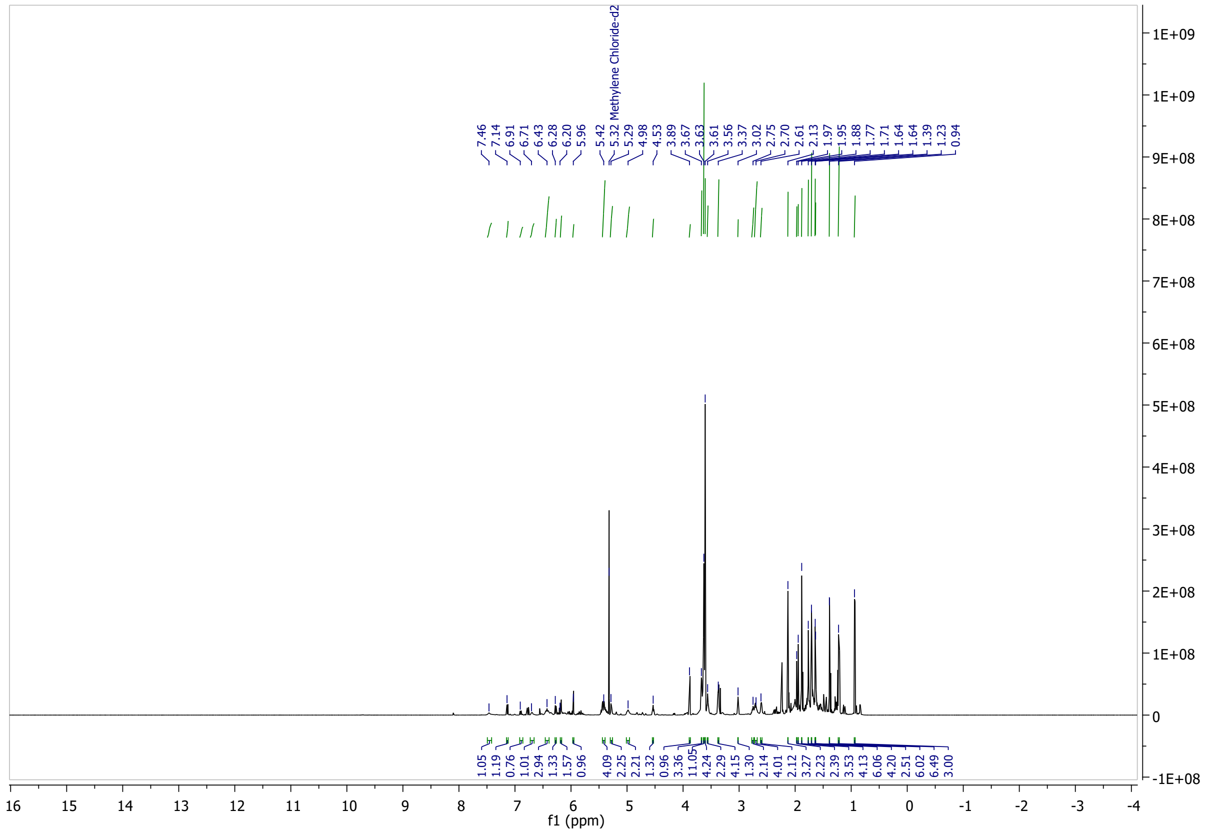

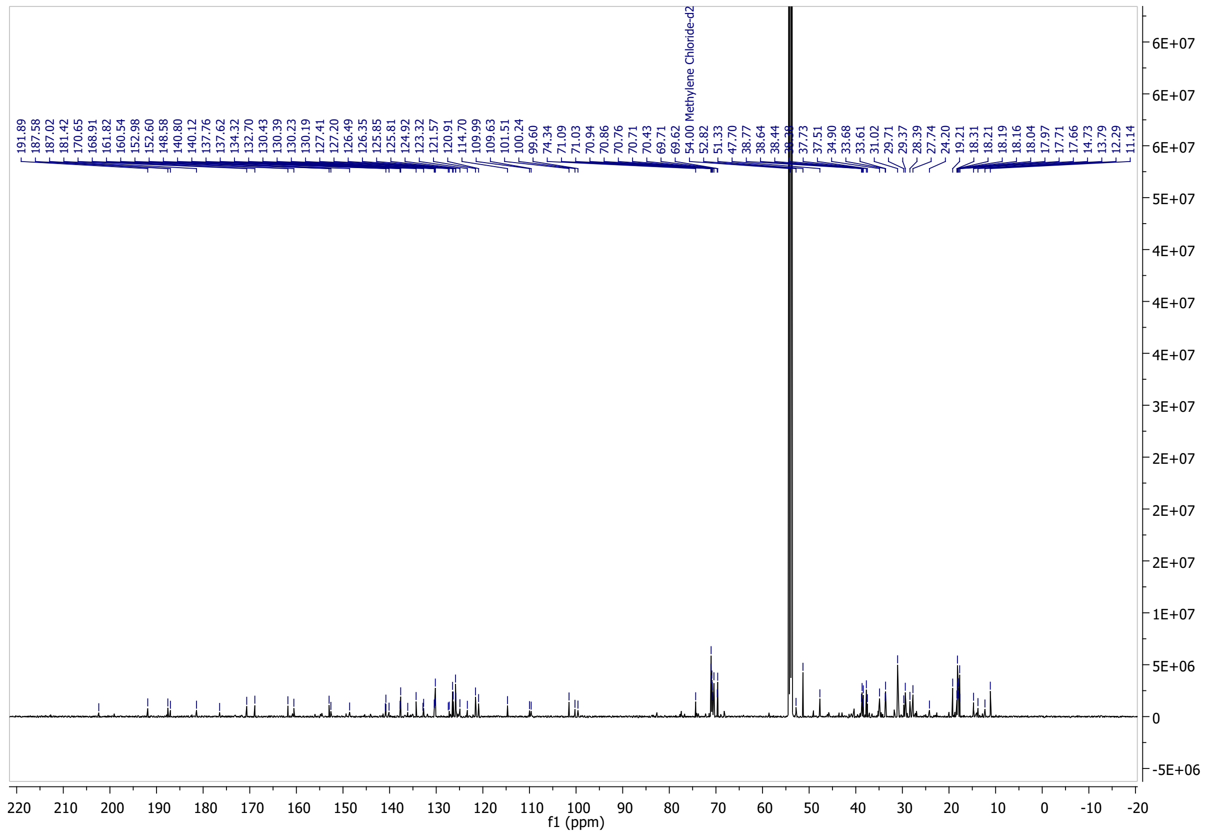

Compound **71**

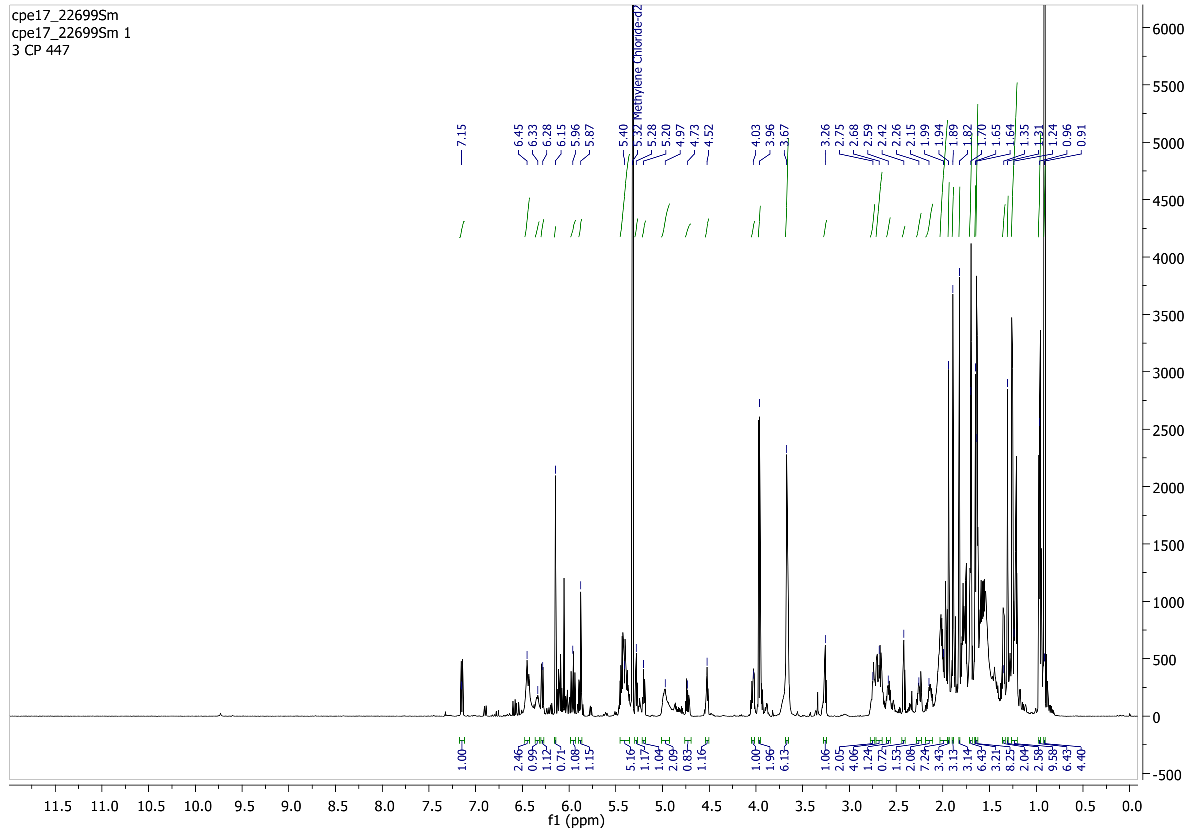
cpe17_22699Sm
cpe17_22699Sm 1
3 CP 447
Methylene Chloride-d2
7.15
6.45
6.33
6.28
6.15
5.96
5.87
5.40
5.32
5.28
5.20
4.97
4.73
4.52
4.03
3.96
3.67
3.26
2.75
2.68
2.59
2.42
2.26
2.15
1.99
1.94
1.89
1.82
1.70
1.65
1.64
1.35
1.31
1.24
0.96
0.91
f1 (ppm)

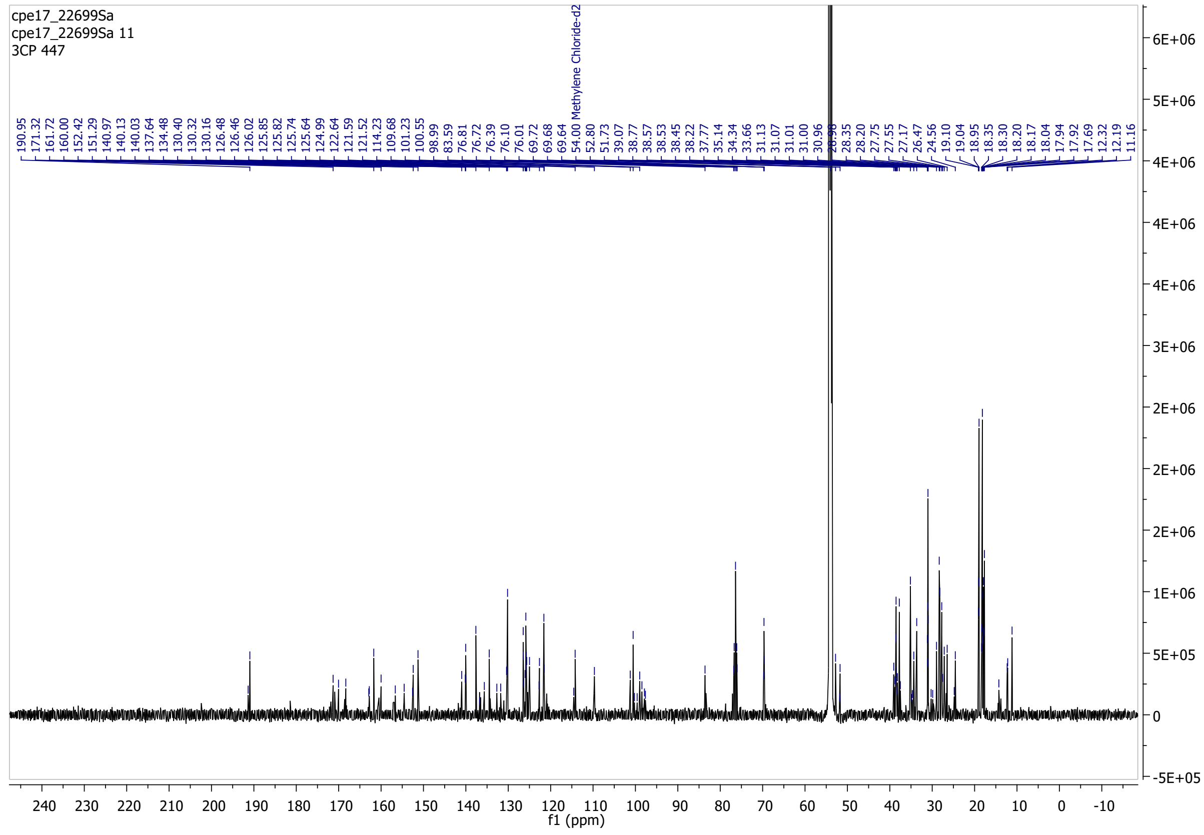
cpe17_22699Sa
cpe17_22699Sa 11
3CP 447
Methylene Chloride-d2
190.95
171.32
161.72
160.00
152.42
151.29
140.97
140.13
140.03
137.64
134.48
130.40
130.32
130.16
126.48
126.46
126.02
125.85
125.82
125.74
125.64
124.99
122.64
121.59
121.52
114.23
109.68
101.23
100.55
98.99
83.59
76.81
76.72
76.39
76.10
76.01
69.72
69.68
69.64
54.00
52.80
51.73
39.07
38.77
38.57
38.53
38.45
38.22
37.77
35.14
34.34
33.66
31.13
31.07
31.01
31.00
30.96
28.35
28.20
27.75
27.55
27.17
26.47
24.56
19.10
19.04
18.95
18.35
18.30
18.20
18.17
18.04
17.94
17.92
17.69
12.32
12.19
11.16
f1 (ppm)

Compound **72**

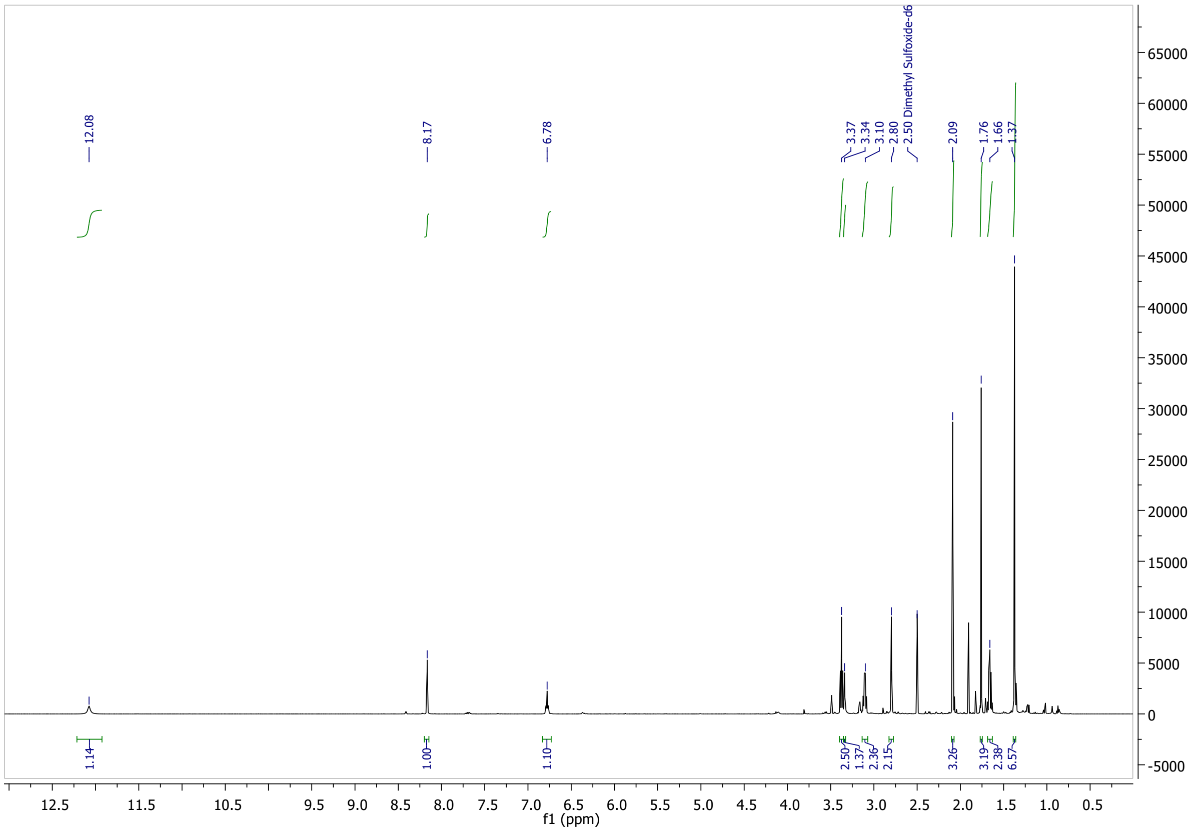
12.08
8.17
6.78
3.37
3.34
3.10
2.80
2.50 Dimethyl Sulfoxide-d6
2.09
1.76
1.66
1.37
1.14
1.00
1.10
2.50
1.37
2.36
2.15
3.26
3.19
2.38
6.57
f1 (ppm)

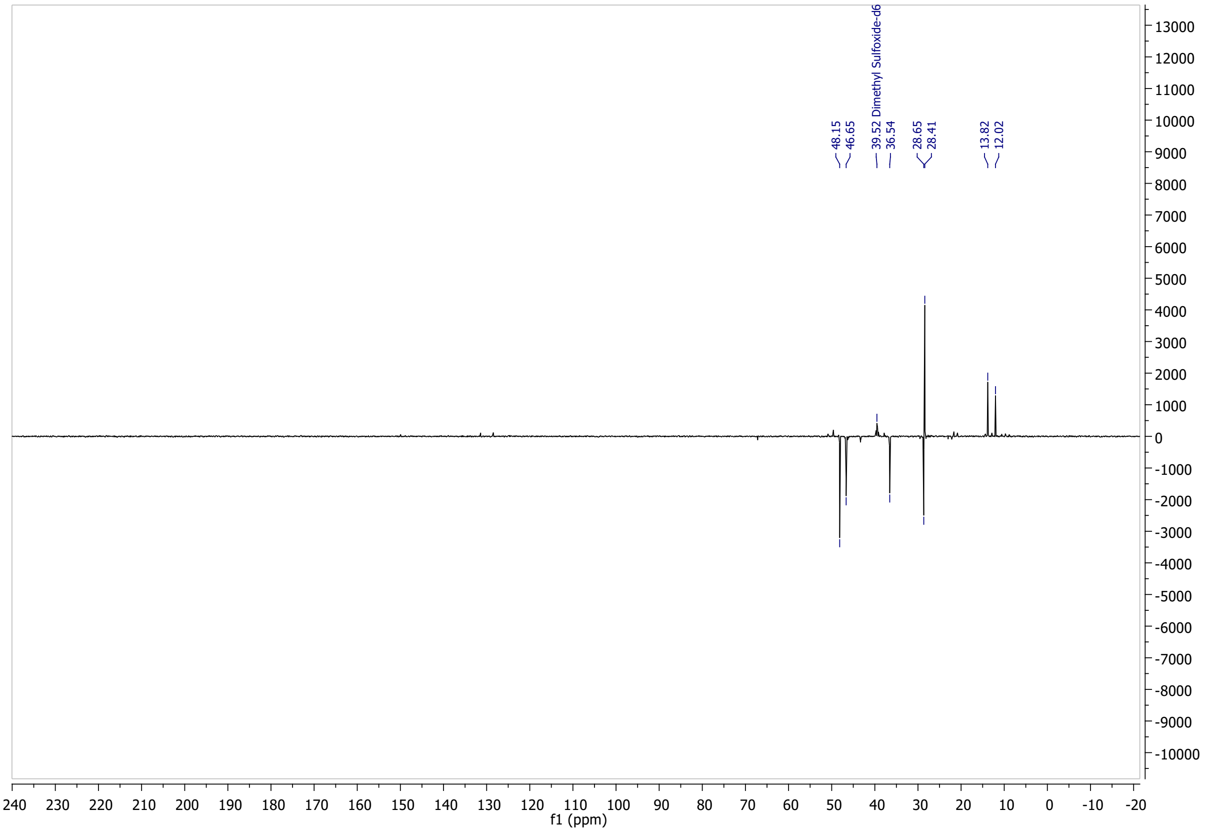
48.15
46.65
39.52 Dimethyl Sulfoxide-d6
36.54
28.65
28.41
13.82
12.02
f1 (ppm)

Compound **73**

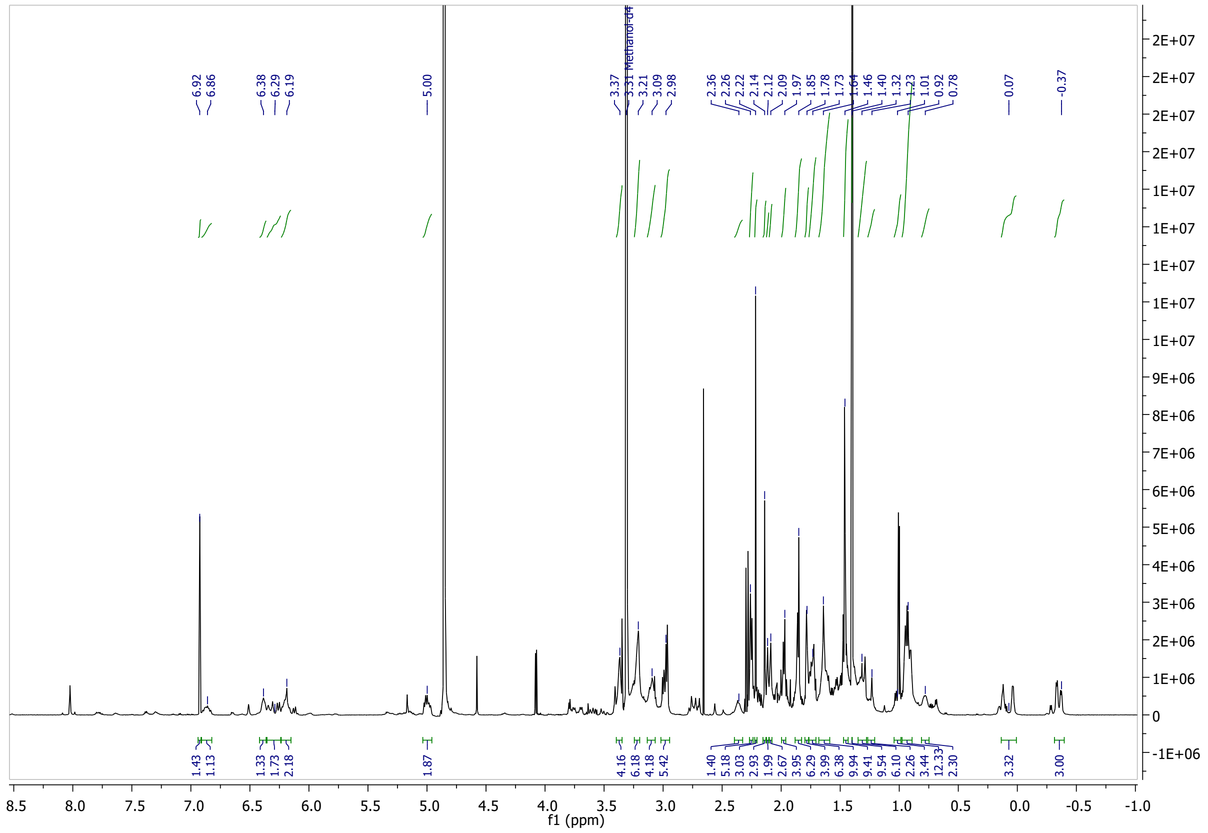

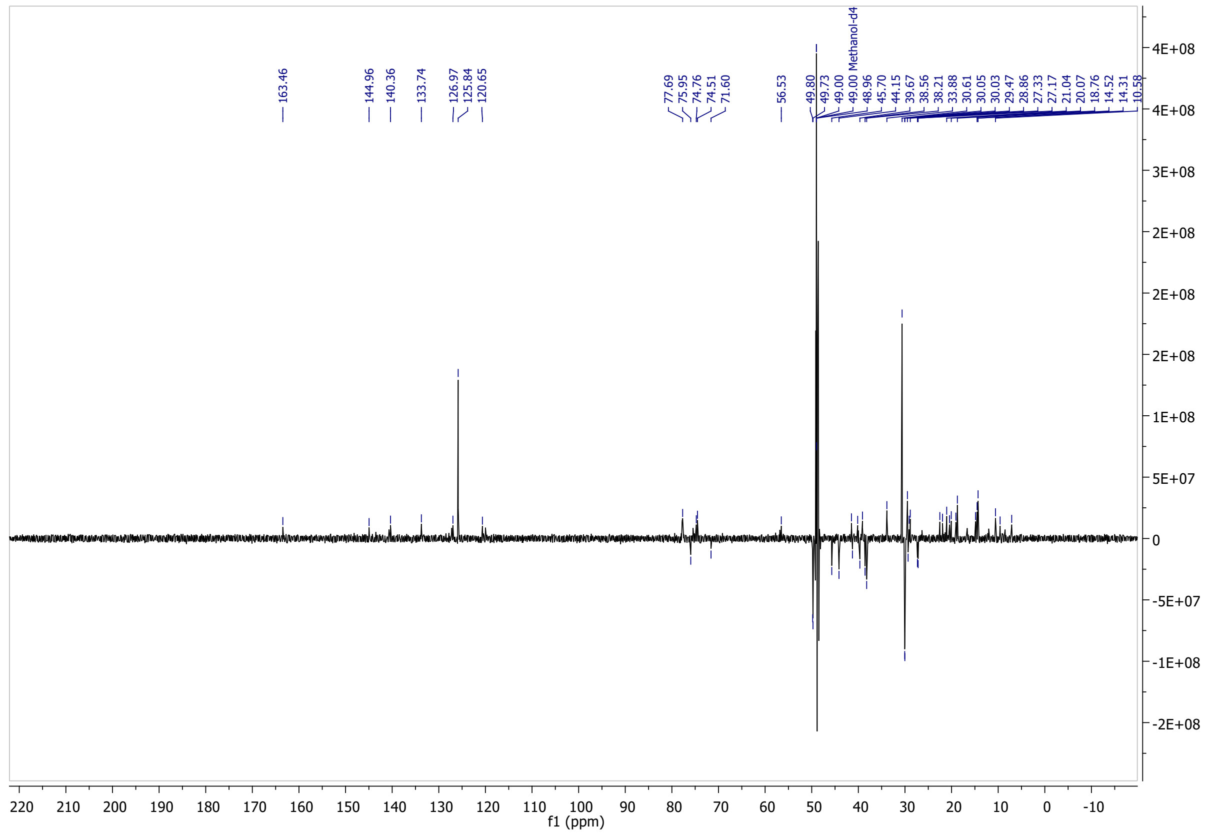

Compound **74**

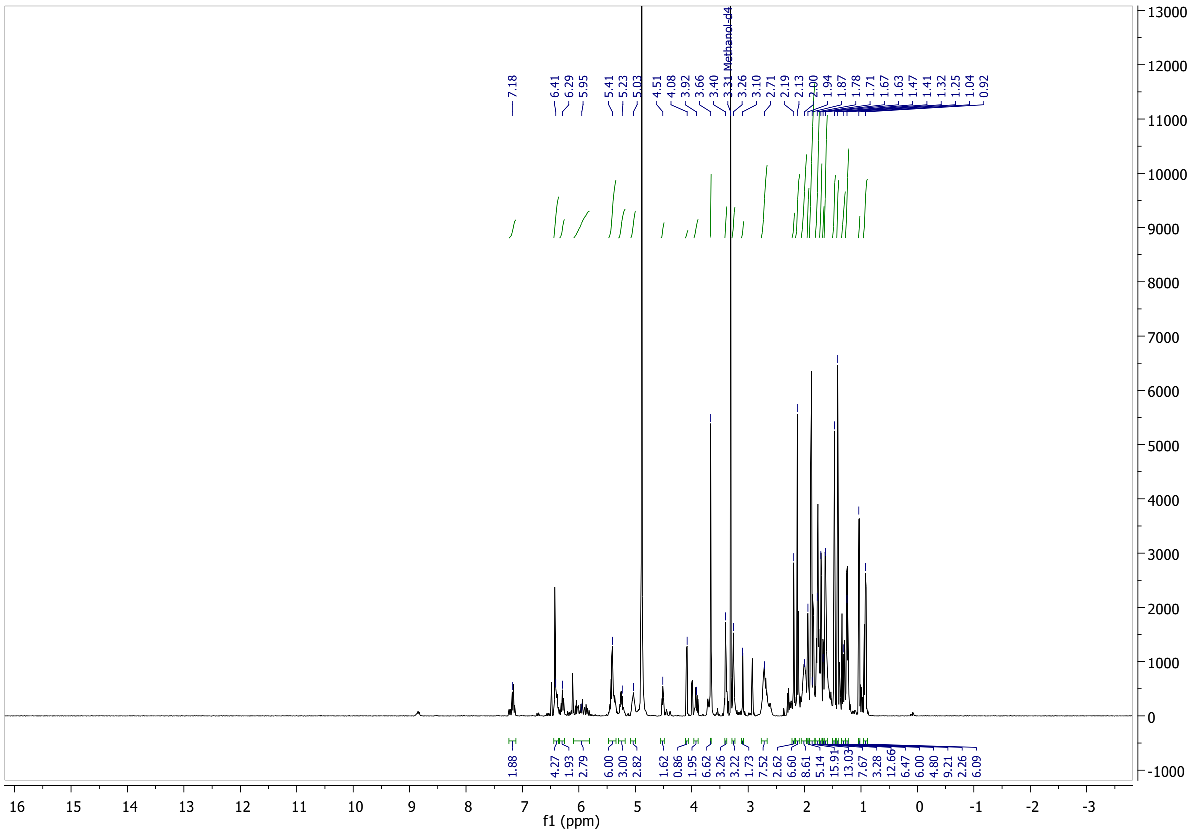

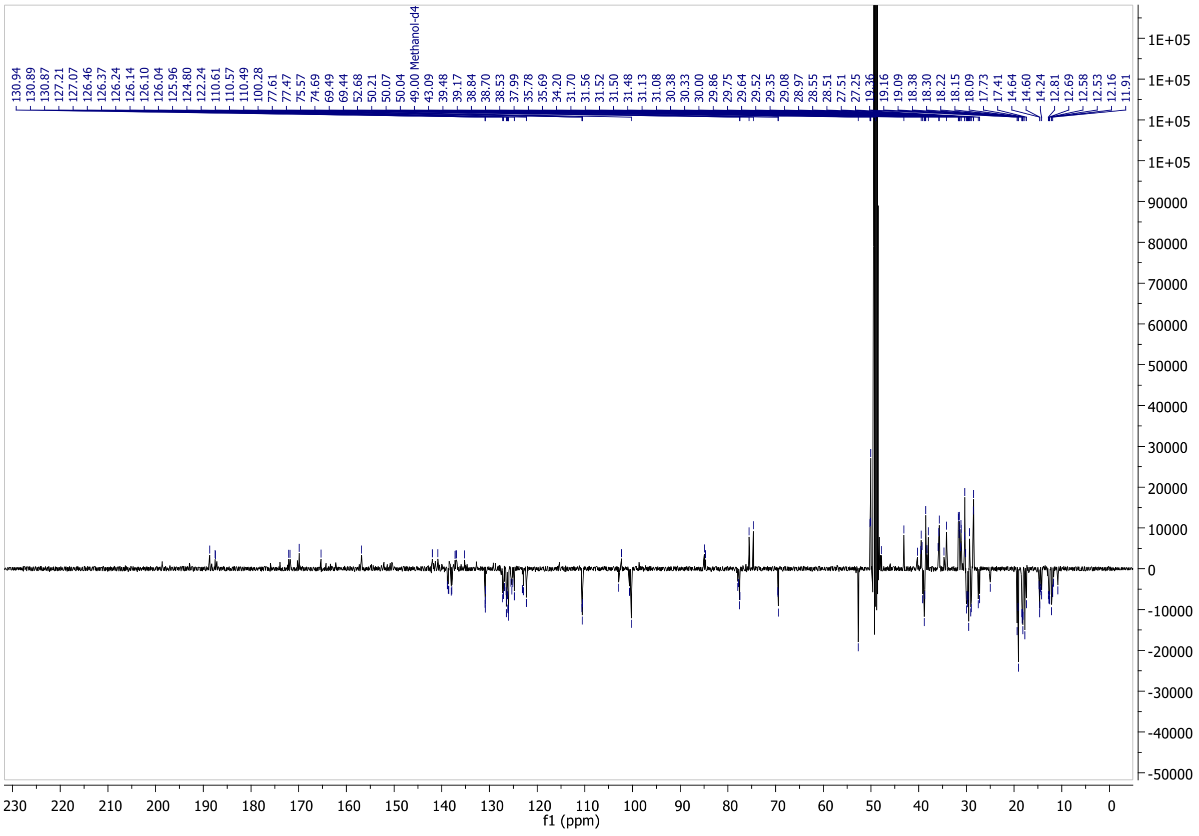

Compound **4**

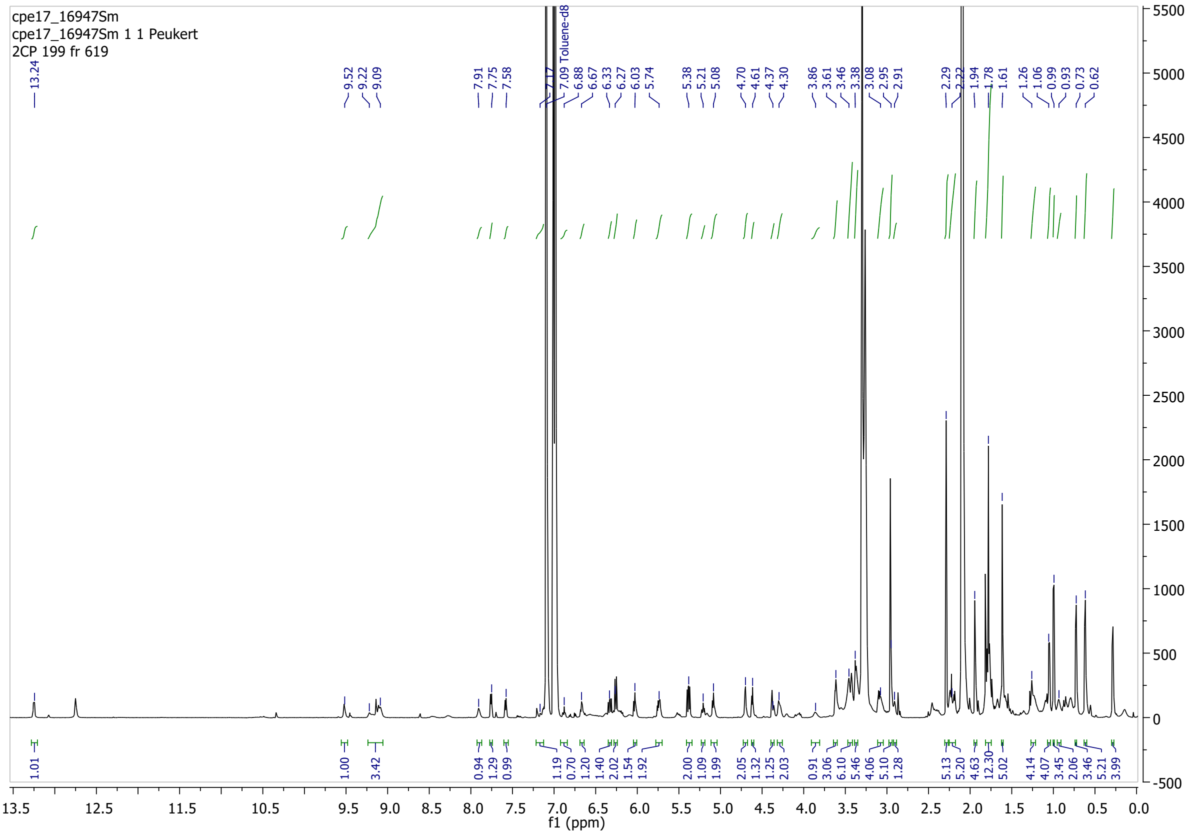
cpe17_16947Sm
cpe17_16947Sm 1 1 Peukert
2CP 199 fr 619
13.24
9.52
9.22
9.09
7.91
7.75
7.58
7.12
7.09 Toluene-d8
6.88
6.67
6.33
6.27
6.03
5.74
5.38
5.21
5.08
4.70
4.61
4.37
4.30
3.86
3.61
3.46
3.38
3.08
2.95
2.91
2.29
2.22
1.94
1.78
1.61
1.26
1.06
0.99
0.93
0.73
0.62
1.01
1.00
3.42
0.94
1.29
0.99
1.19
0.70
1.20
1.40
2.02
1.54
1.92
2.00
1.09
1.99
2.05
1.32
1.25
2.03
0.91
3.06
6.10
5.46
4.06
5.10
1.28
5.13
5.20
4.63
12.30
5.02
4.14
4.07
3.45
2.06
3.46
5.21
3.99
f1 (ppm)

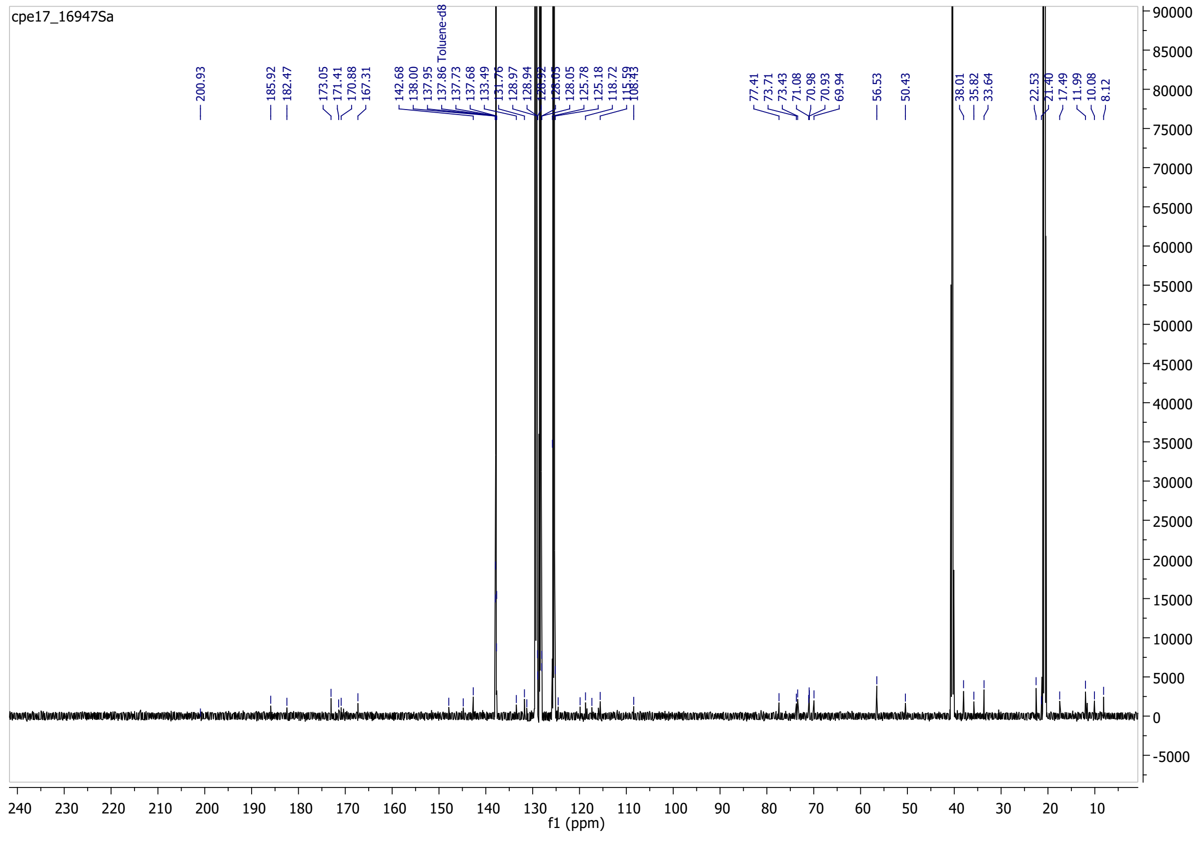
cpe17_16947Sa
200.93
185.92
182.47
173.05
171.41
170.88
167.31
142.68
138.00
137.95
137.86 Toluene-d8
137.73
137.68
133.49
131.76
128.97
128.94
128.92
128.05
128.05
125.78
125.18
118.72
115.59
108.43
77.41
73.71
73.43
71.08
70.98
70.93
69.94
56.53
50.43
38.01
35.82
33.64
22.53
21.40
17.49
11.99
10.08
8.12
f1 (ppm)

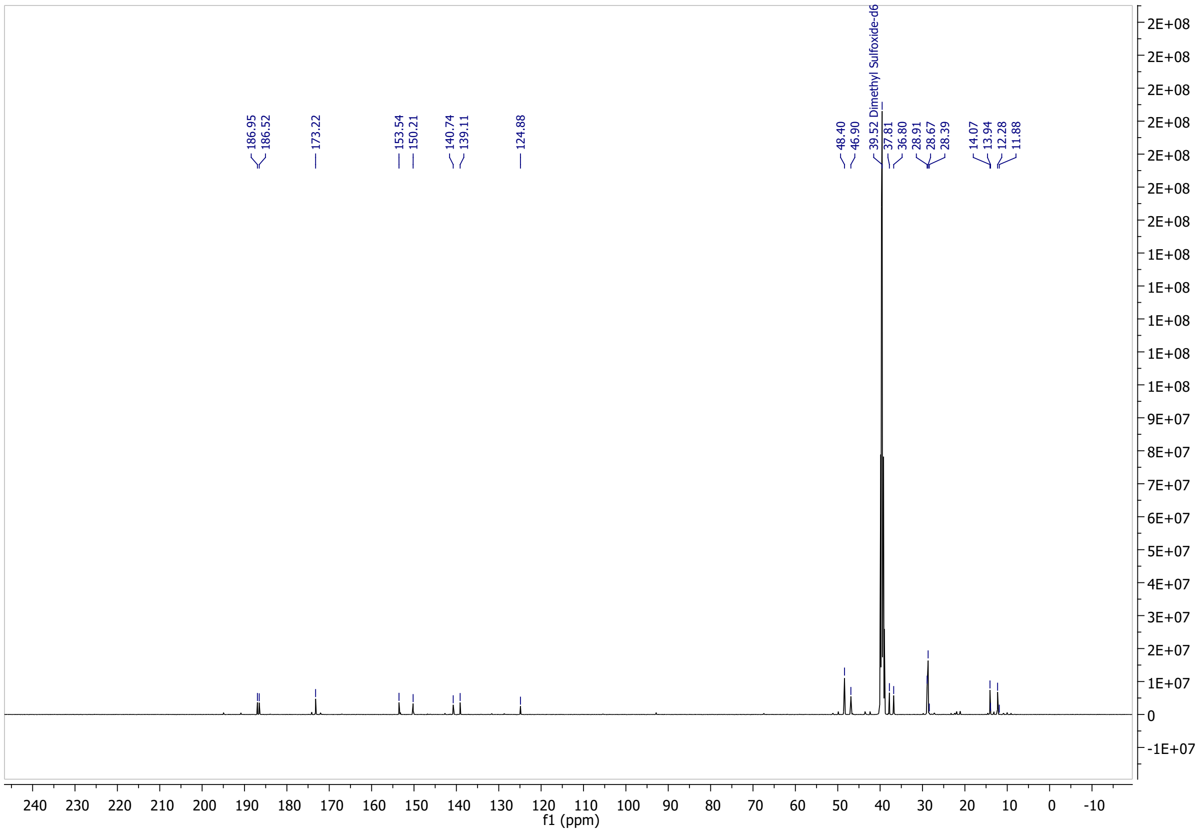
186.95
186.52
173.22
153.54
150.21
140.74
139.11
124.88
48.40
46.90
39.52 Dimethyl Sulfoxide-d6
37.81
36.80
28.91
28.67
28.39
14.07
13.94
12.28
11.88
f1 (ppm)

Compound **5a**

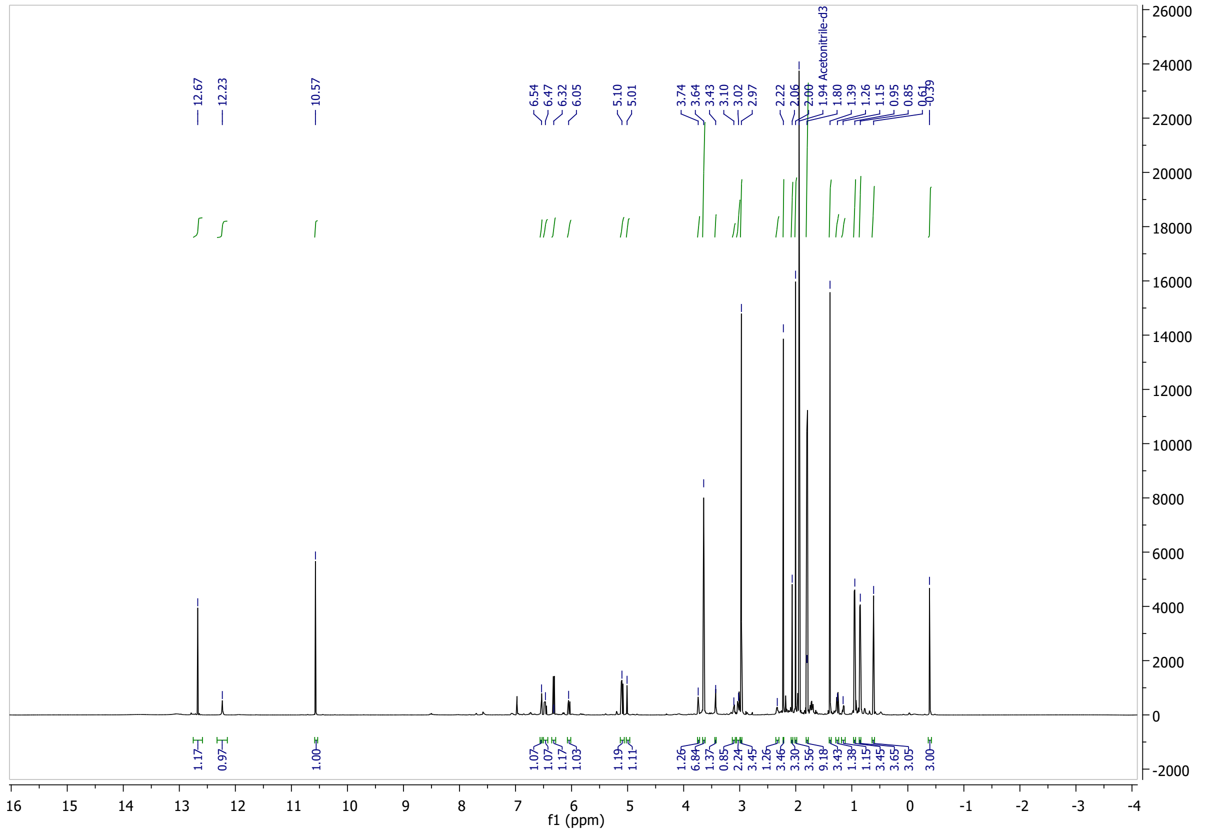

Compound **5**

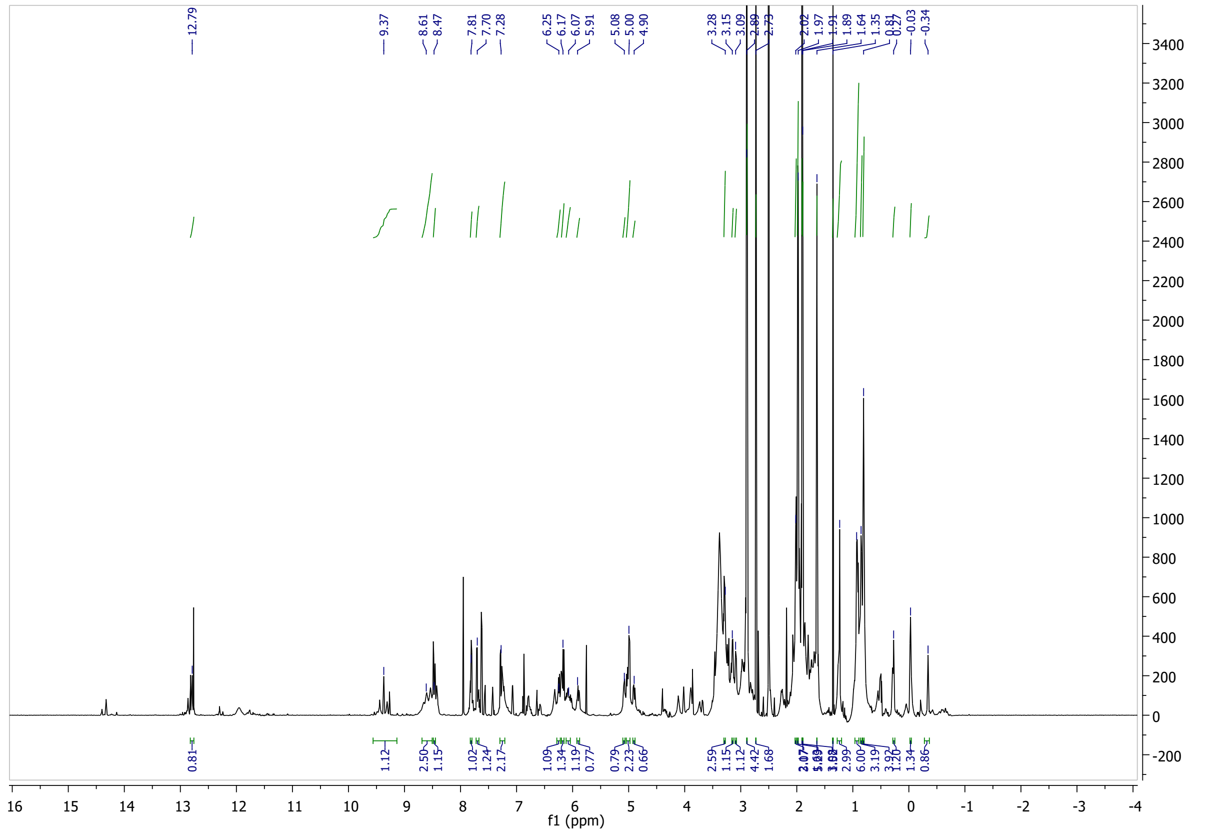

Compound **6**

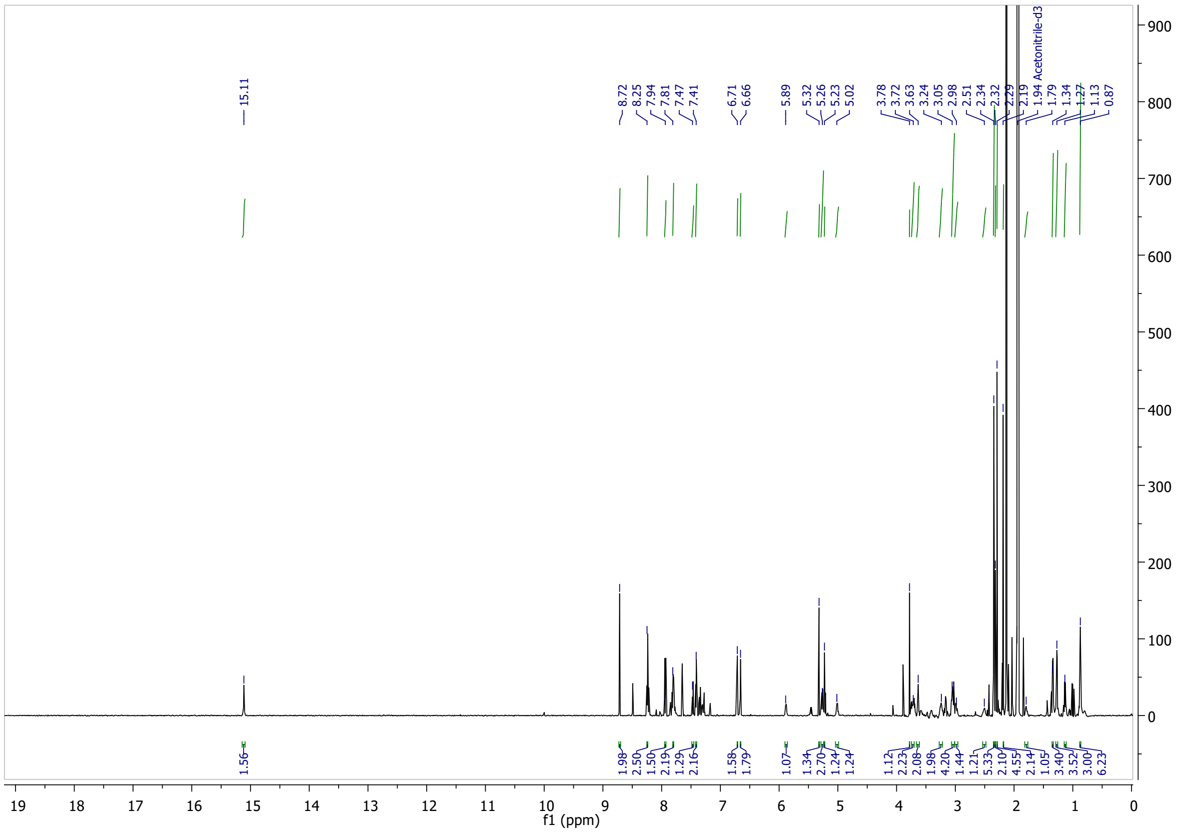
15.11
8.72
8.25
7.94
7.81
7.47
7.41
6.71
6.66
5.89
5.32
5.26
5.23
5.02
3.78
3.72
3.63
3.24
3.05
2.98
2.51
2.34
2.32
2.29
2.19
1.94 Acetonitrile-d3
1.79
1.34
1.27
1.13
0.87
f1 (ppm)

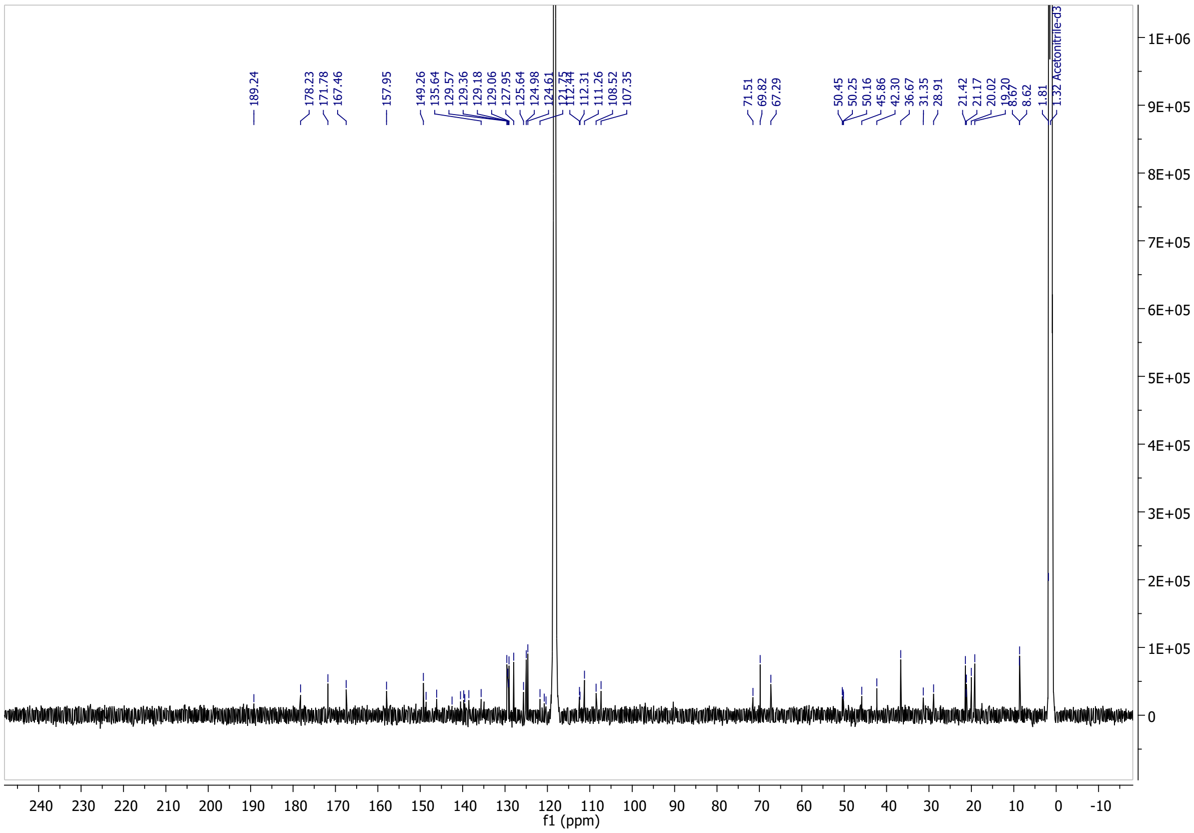
189.24
178.23
171.78
167.46
157.95
149.26
135.64
129.57
129.36
129.18
129.06
127.95
125.64
124.98
124.61
112.31
111.26
108.52
107.35
71.51
69.82
67.29
50.45
50.25
50.16
45.86
42.30
36.67
31.35
28.91
21.42
21.17
20.02
19.20
8.67
8.62
1.81
1.32 Acetonitrile-d3
f1 (ppm)

Compound **3**

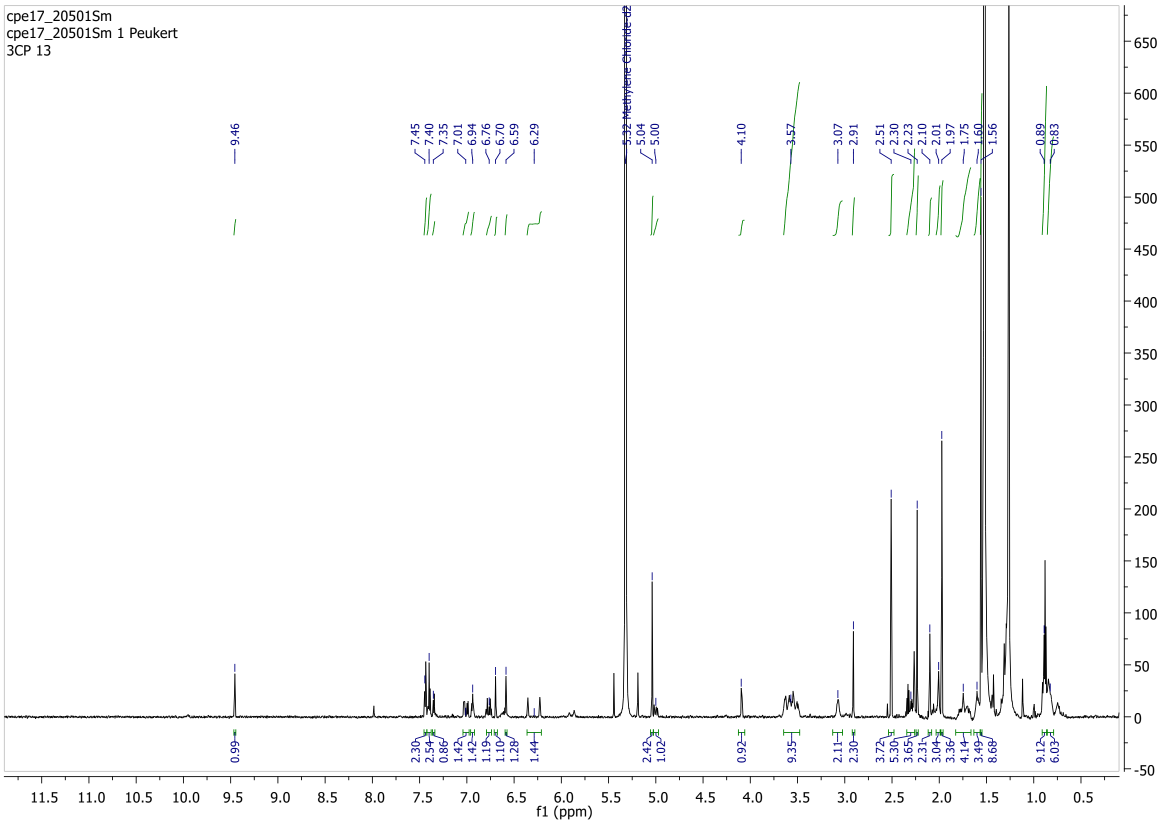
cpe17_20501Sm
cpe17_20501Sm 1 Peukert
3CP 13
5.32 Methylene Chloride-d2
f1 (ppm)

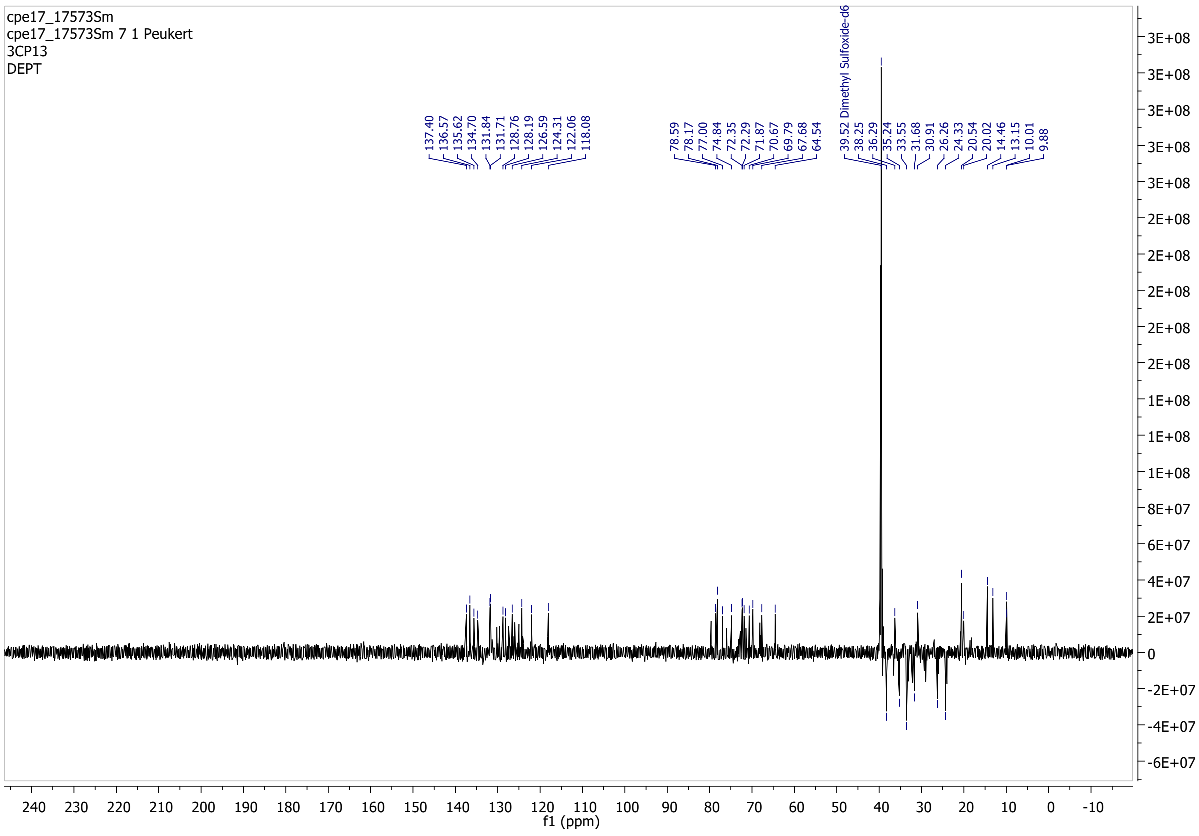
cpe17_17573Sm
cpe17_17573Sm 7 1 Peukert
3CP13
DEPT
39.52 Dimethyl Sulfoxide-d6
f1 (ppm)

Compound **18**

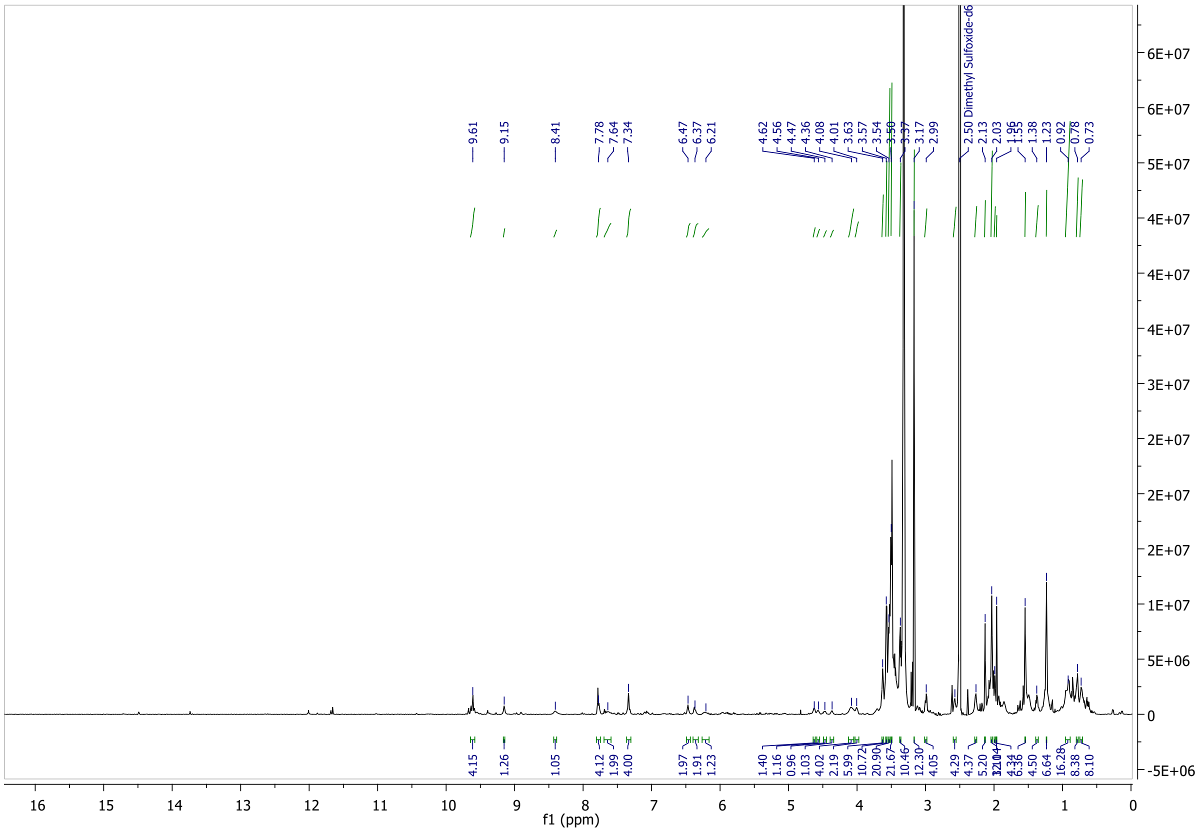
2.50 Dimethyl Sulfoxide-d6
f1 (ppm)

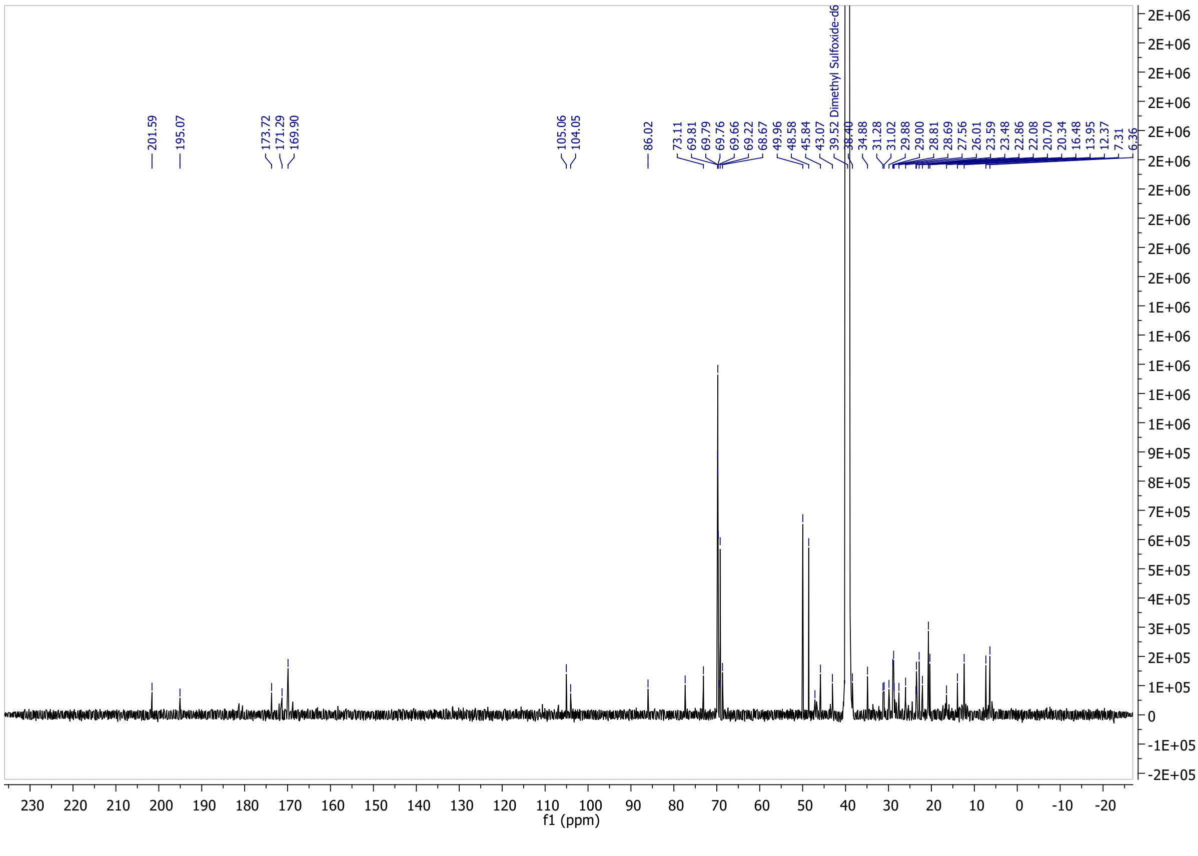
39.52 Dimethyl Sulfoxide-d6
f1 (ppm)

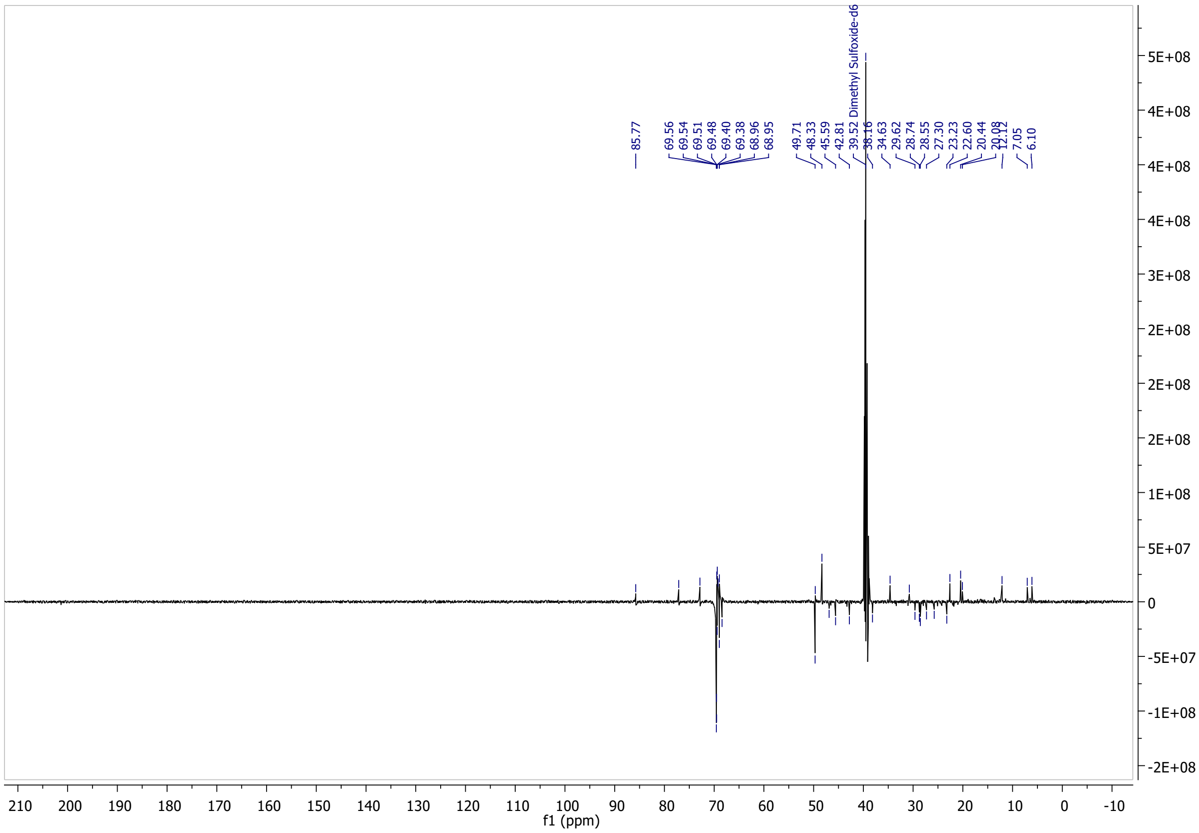

85.77
69.56
69.54
69.51
69.48
69.40
69.38
68.96
68.95
49.71
48.33
45.59
42.81
39.52 Dimethyl Sulfoxide-d6
38.16
34.63
29.62
28.74
28.55
27.30
23.23
22.60
20.44
20.08
12.12
7.05
6.10
f1 (ppm)

Compound **19**

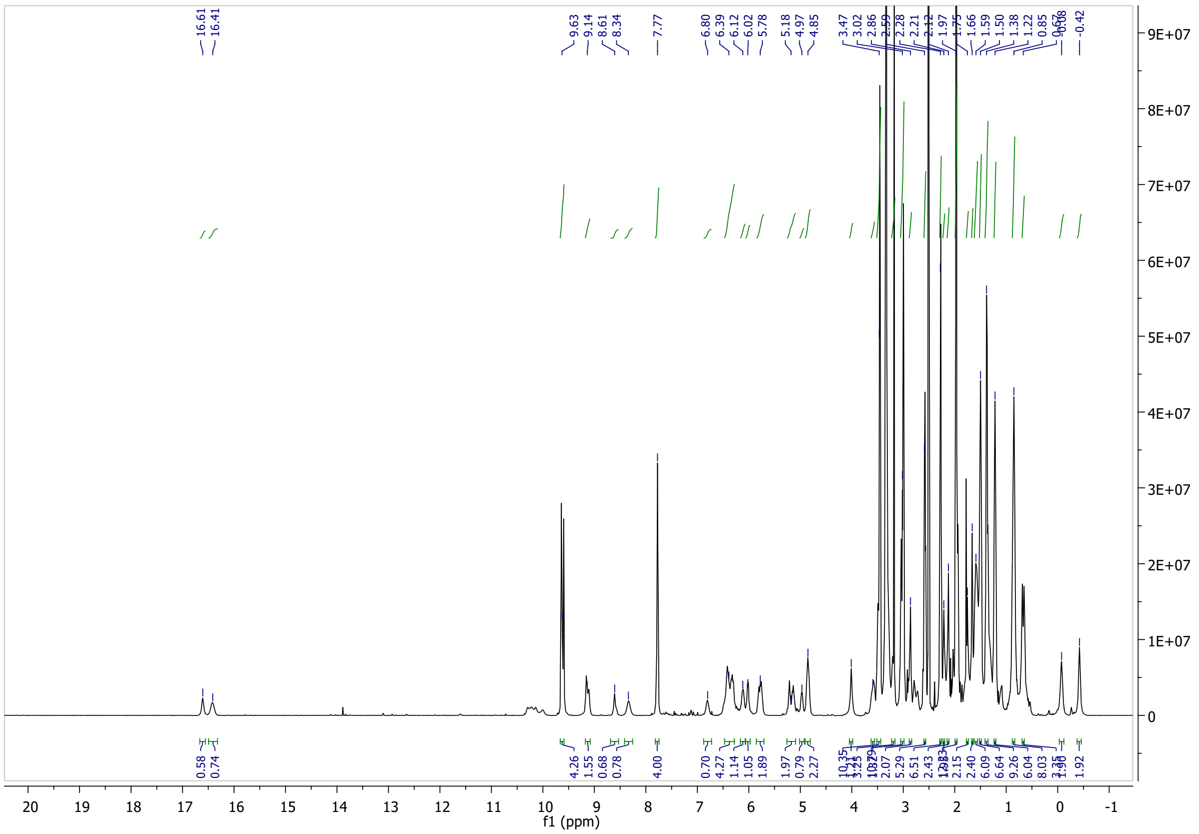

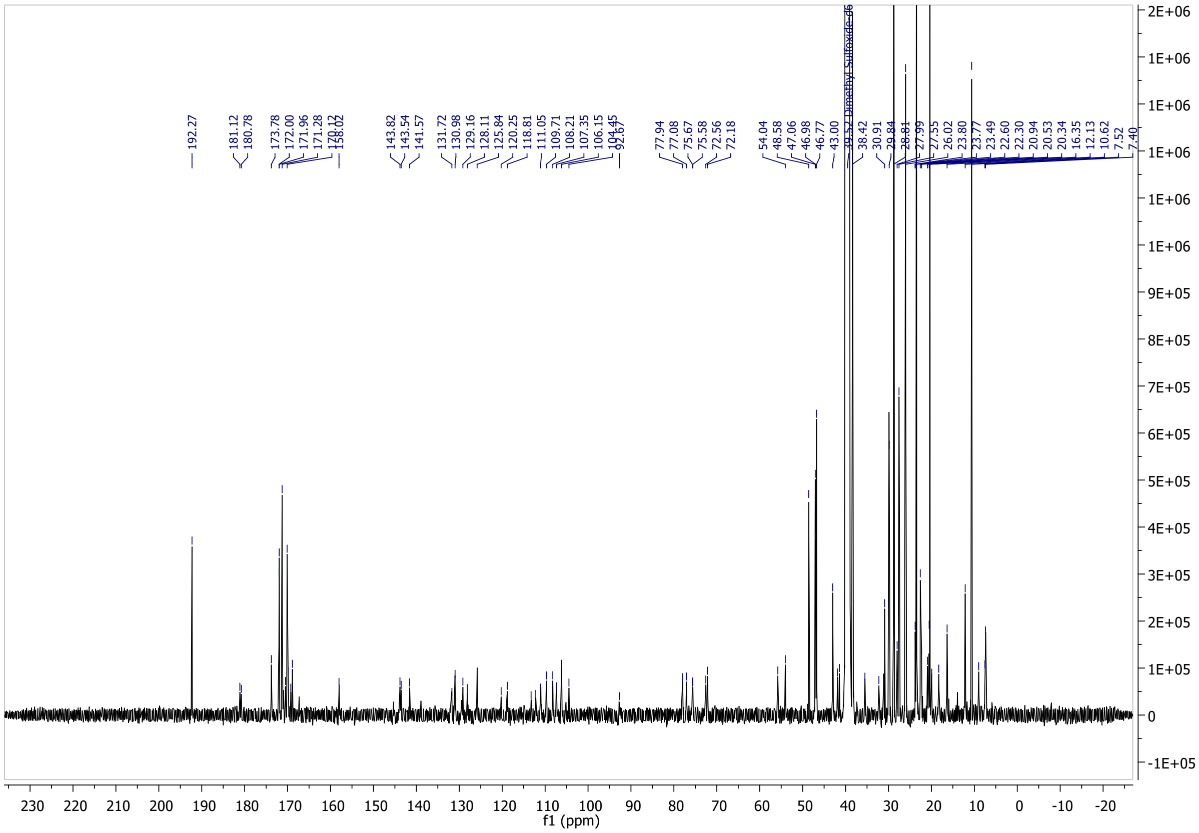

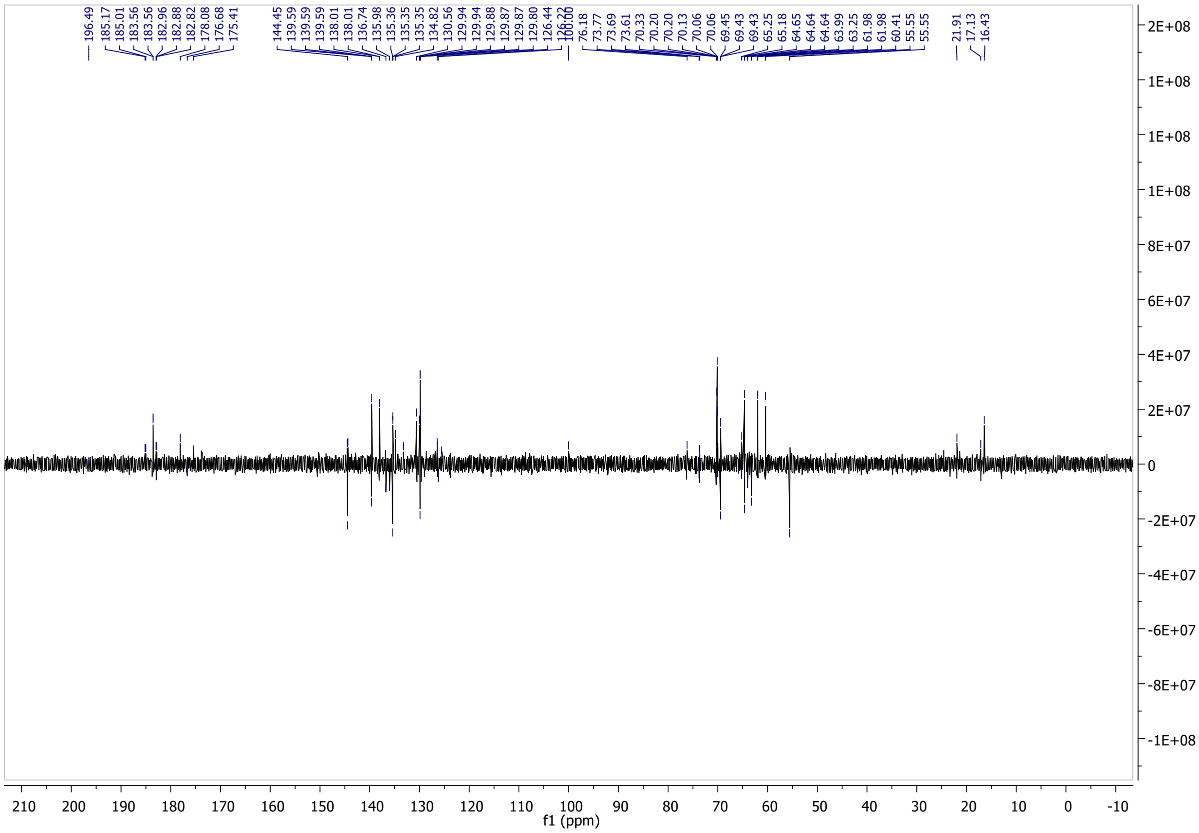
196.49
185.17
185.01
183.56
183.56
182.96
182.88
182.82
178.08
176.68
175.41
144.45
139.59
139.59
139.59
138.01
138.01
136.74
135.98
135.36
135.35
135.35
134.82
130.56
129.94
129.94
129.88
129.87
129.87
129.80
126.44
100.12
100.00
76.18
73.77
73.69
73.61
70.33
70.20
70.20
70.13
70.06
70.06
69.45
69.43
69.43
65.25
65.18
64.65
64.64
64.64
63.99
63.25
61.98
61.98
60.41
55.55
55.55
21.91
17.13
16.43
f1 (ppm)

Compound **20**

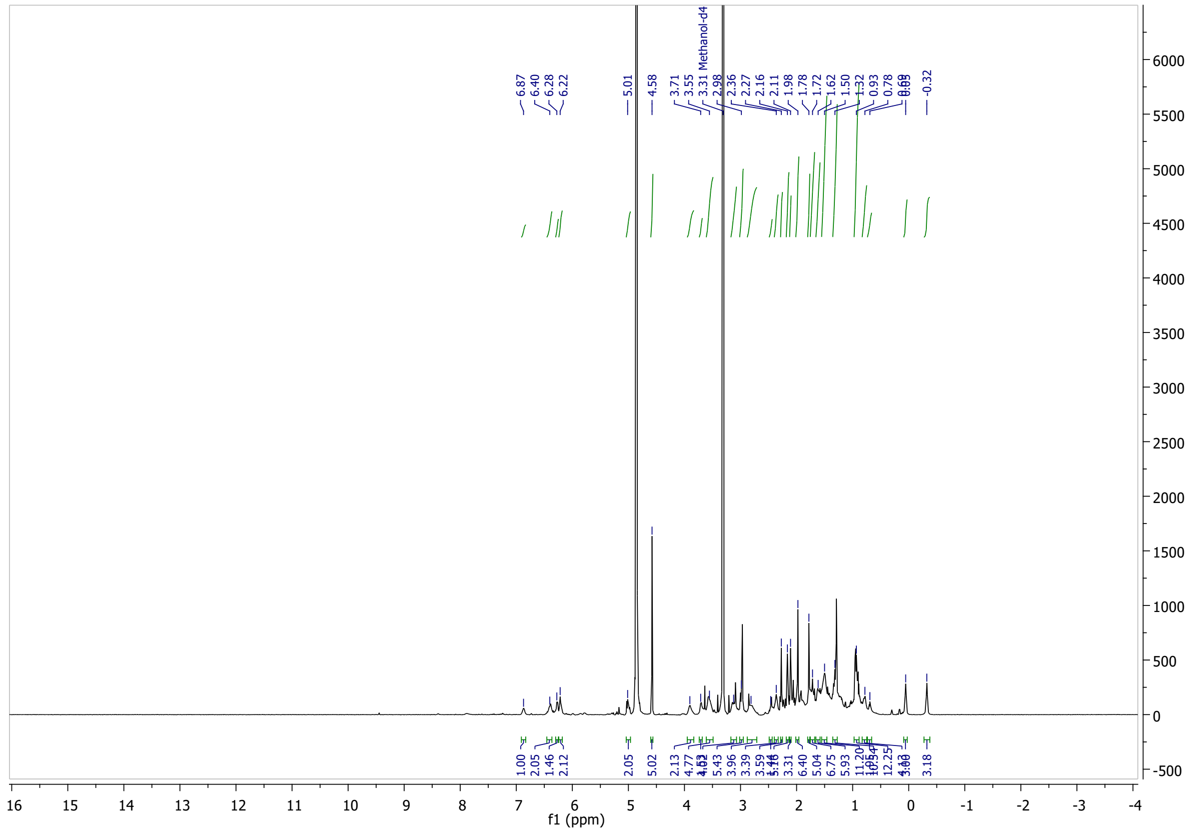

6.87
6.40
6.28
6.22
5.01
4.58
3.71
3.55
3.31 Methanol-d4
2.98
2.36
2.27
2.16
2.11
1.98
1.78
1.72
1.62
1.50
1.32
0.93
0.78
-0.32
1.00
2.05
1.46
2.12
2.05
5.02
2.13
4.77
5.43
3.96
3.39
3.59
3.31
6.40
5.04
6.75
5.93
12.25
3.18
f1 (ppm)

Compound **21**

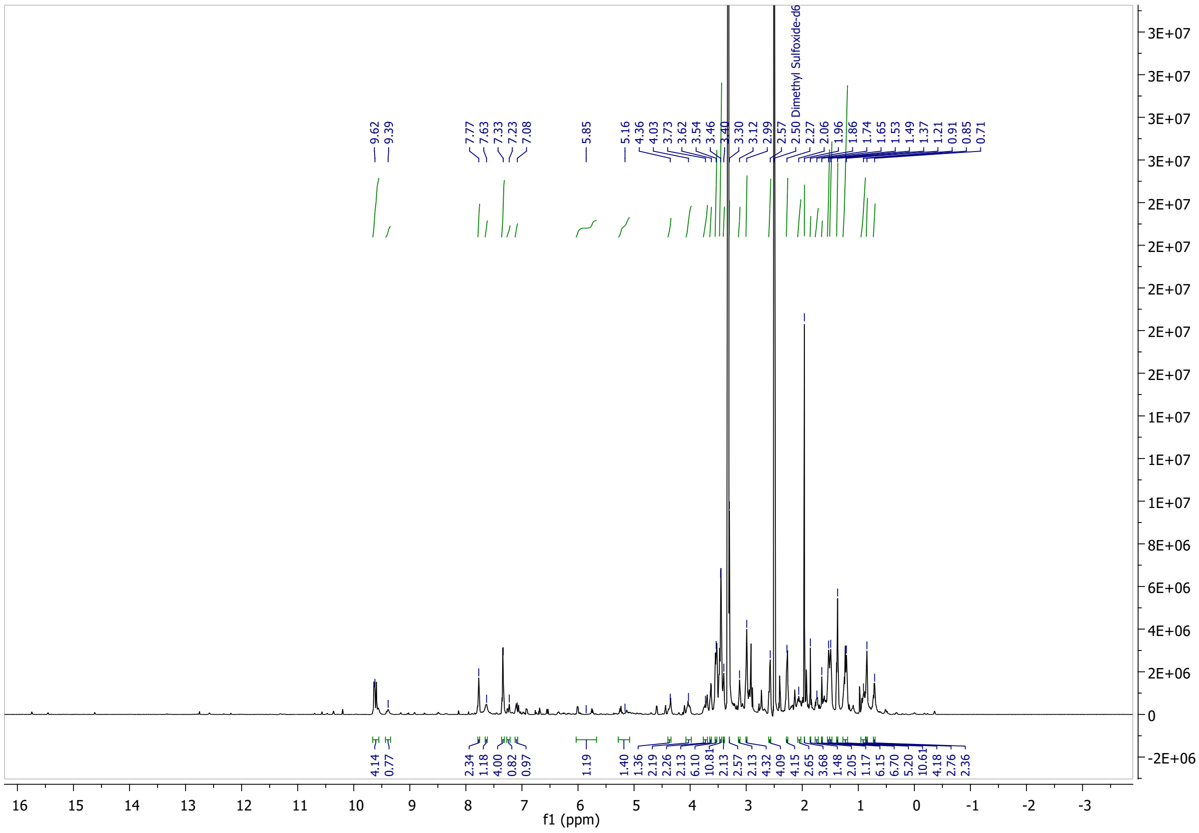

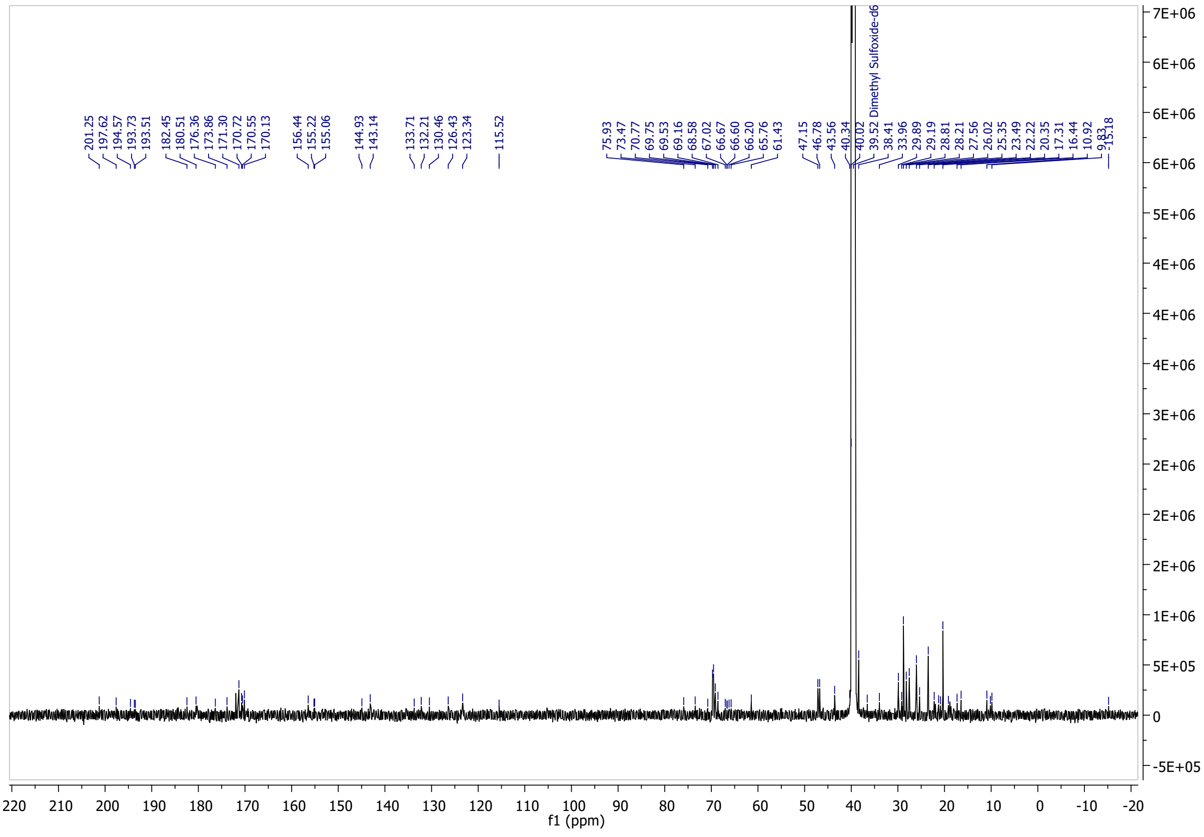

Compound **23**

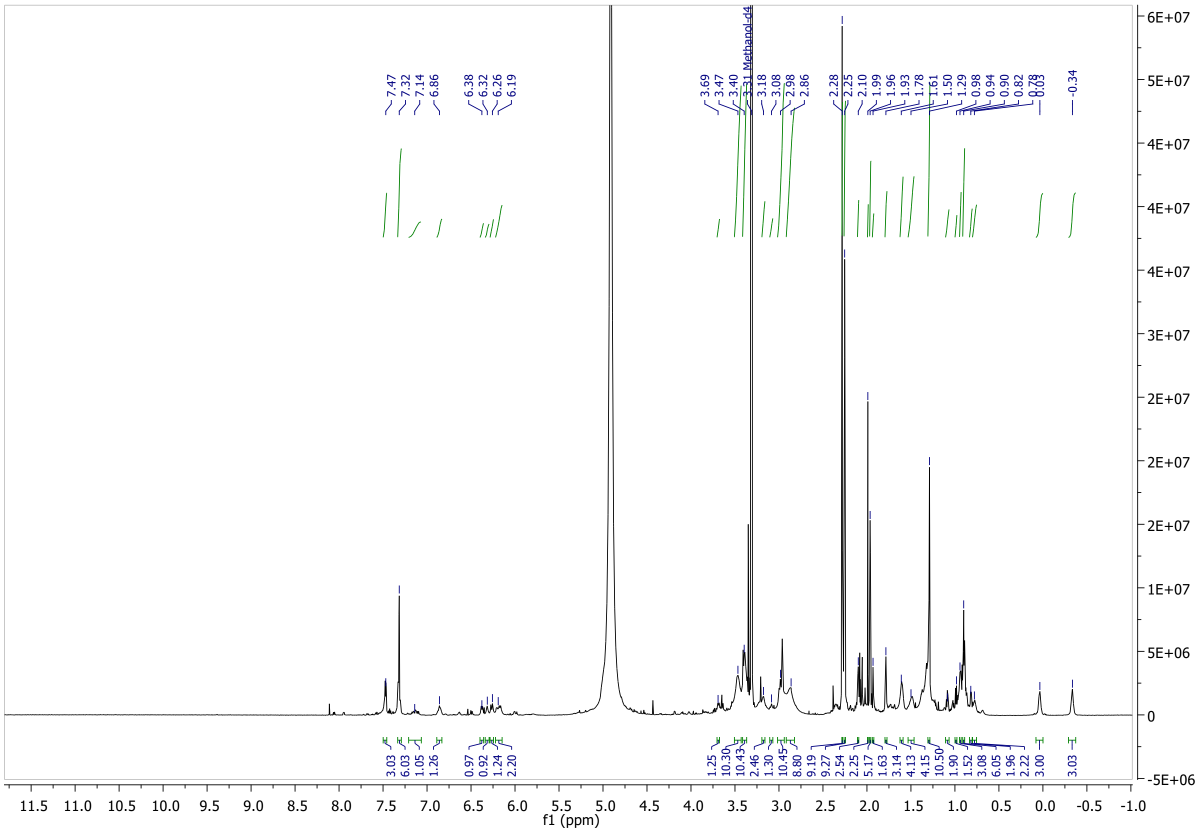

7.47
7.32
7.14
6.86
6.38
6.32
6.26
6.19
3.69
3.47
3.40
3.31 Methanol-d4
3.18
3.08
2.98
2.86
2.28
2.25
2.10
1.99
1.96
1.93
1.78
1.61
1.50
1.29
0.98
0.94
0.90
0.82
0.78
0.03
-0.34
f1 (ppm)

Compound **24**

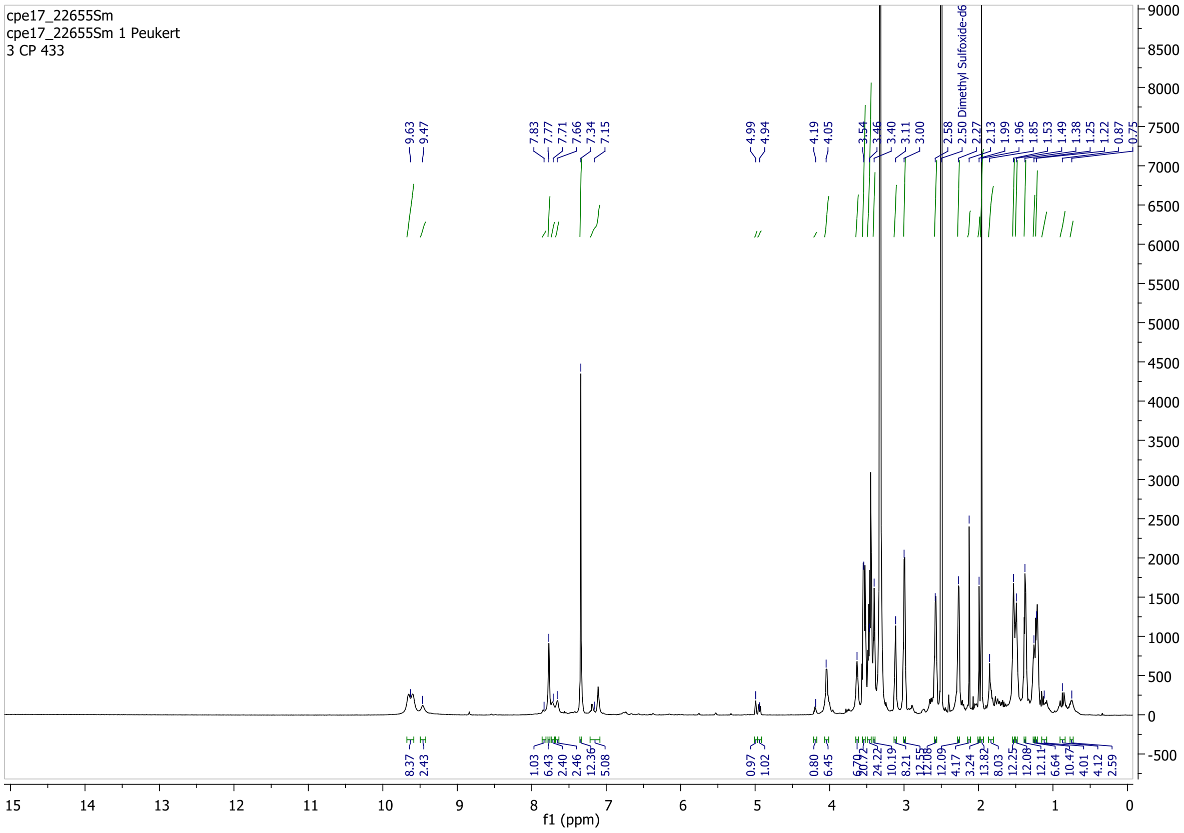
cpe17_22655Sm
cpe17_22655Sm 1 Peukert
3 CP 433
f1 (ppm)
Dimethyl Sulfoxide-d6

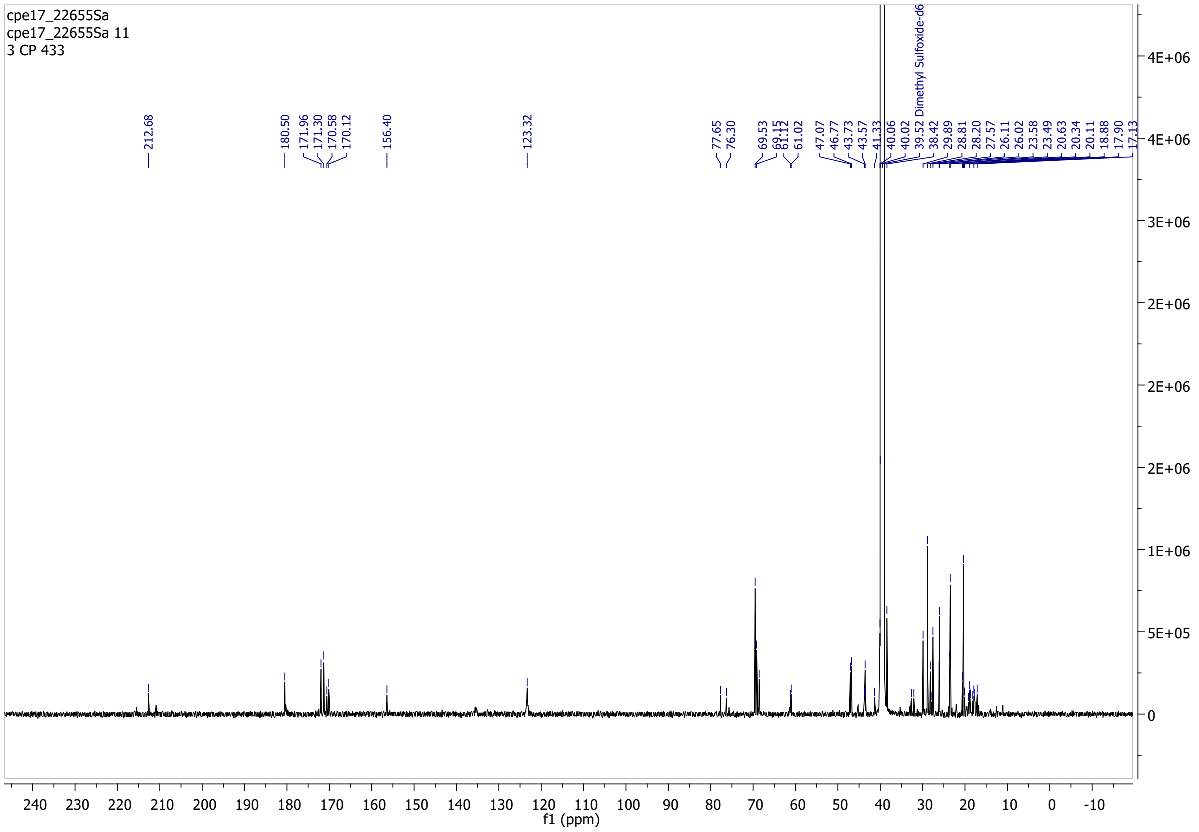
cpe17_22655Sa
cpe17_22655Sa 11
3 CP 433
f1 (ppm)
Dimethyl Sulfoxide-d6

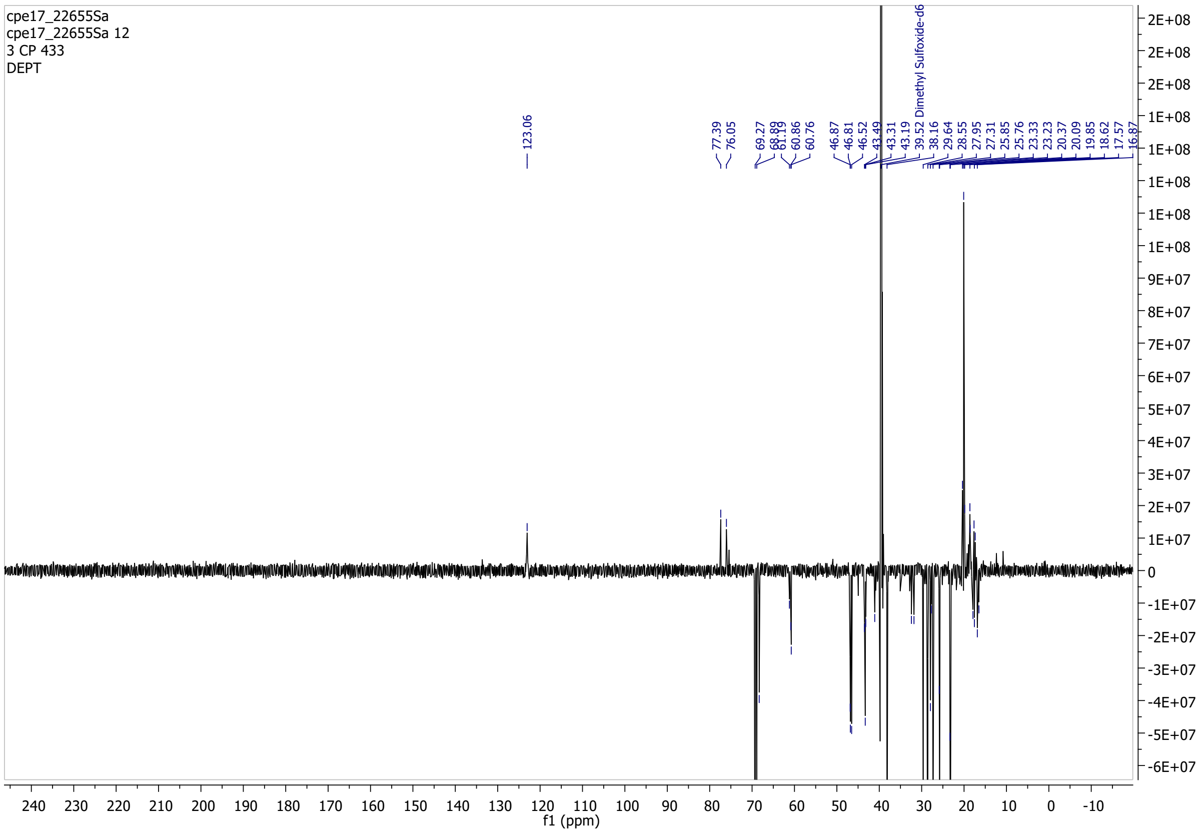
cpe17_22655Sa
cpe17_22655Sa 12
3 CP 433
DEPT
123.06
77.39
76.05
69.27
68.89
61.19
60.86
60.76
46.87
46.81
46.52
43.49
43.31
43.19
39.52 Dimethyl Sulfoxide-d6
38.16
29.64
28.55
27.95
27.31
25.85
25.76
23.33
23.23
20.37
20.09
19.85
18.62
17.57
16.87
f1 (ppm)

Compound **24SL**

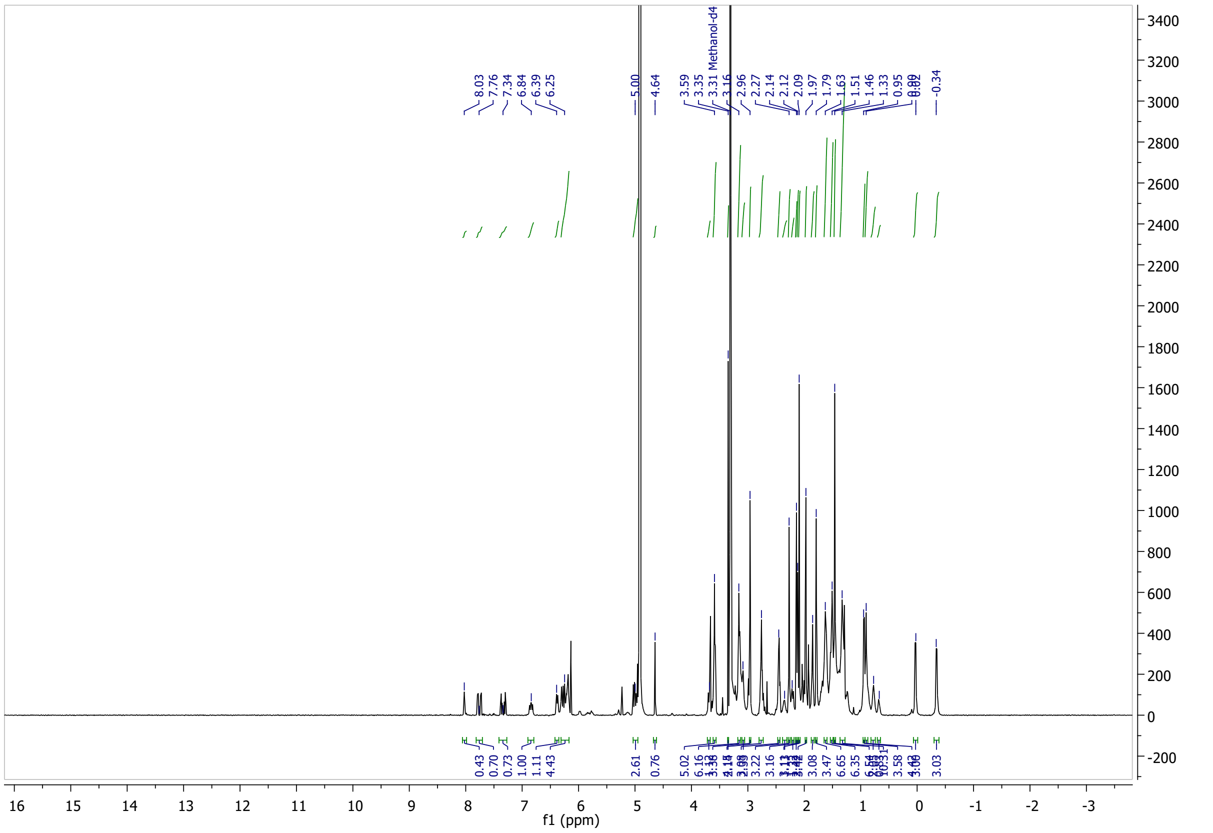

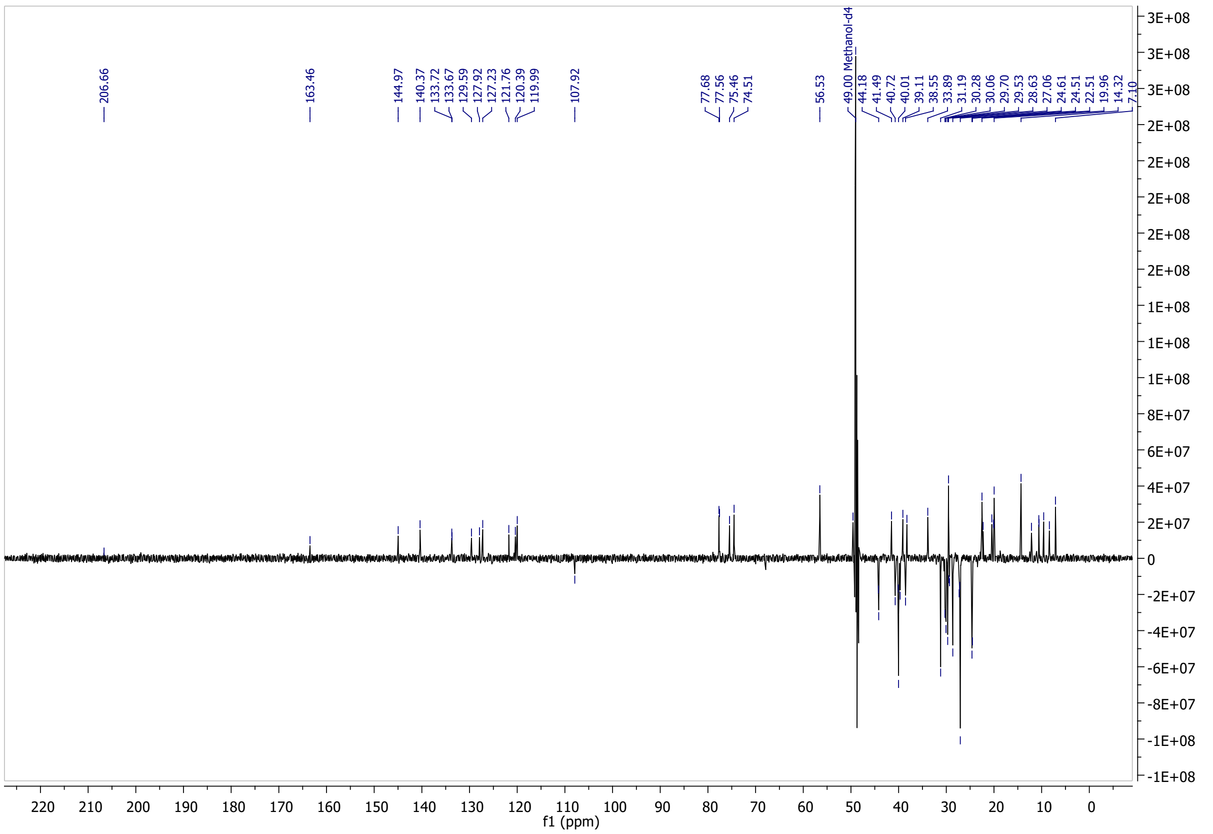

Compound **25**

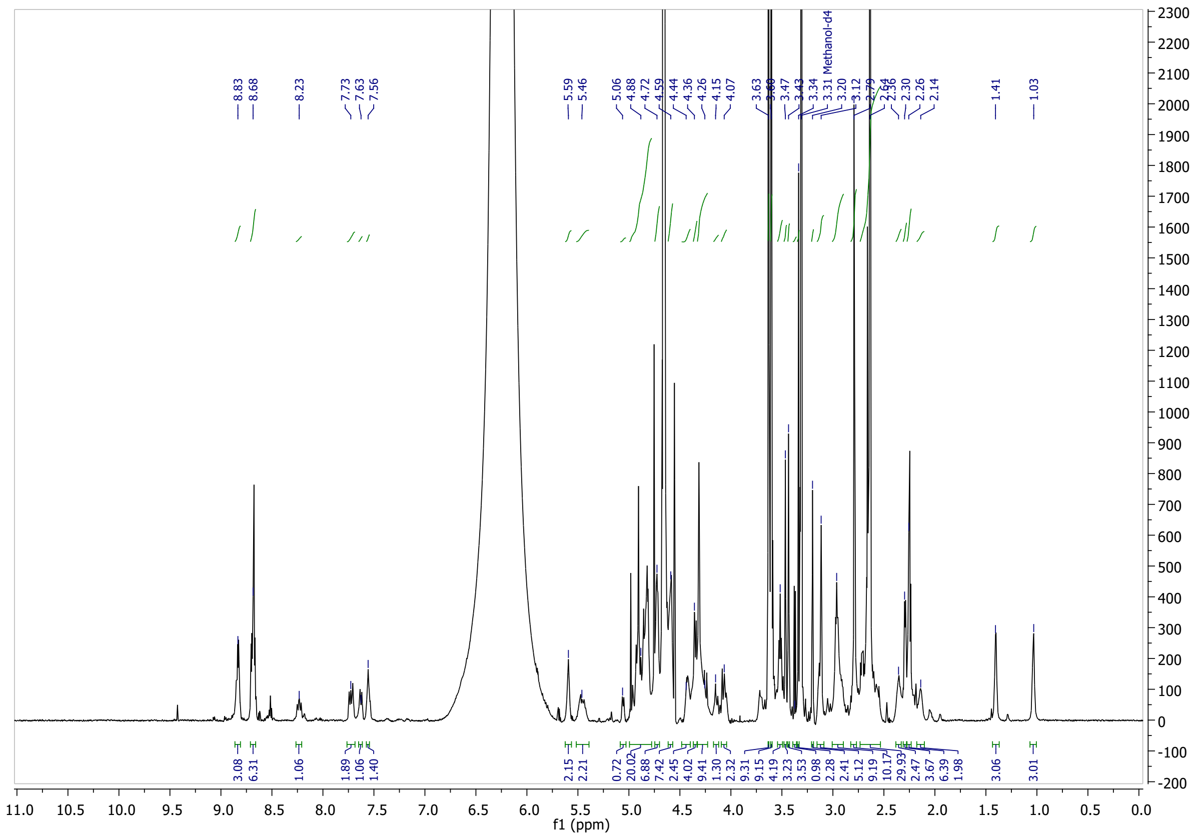

Compound **26**

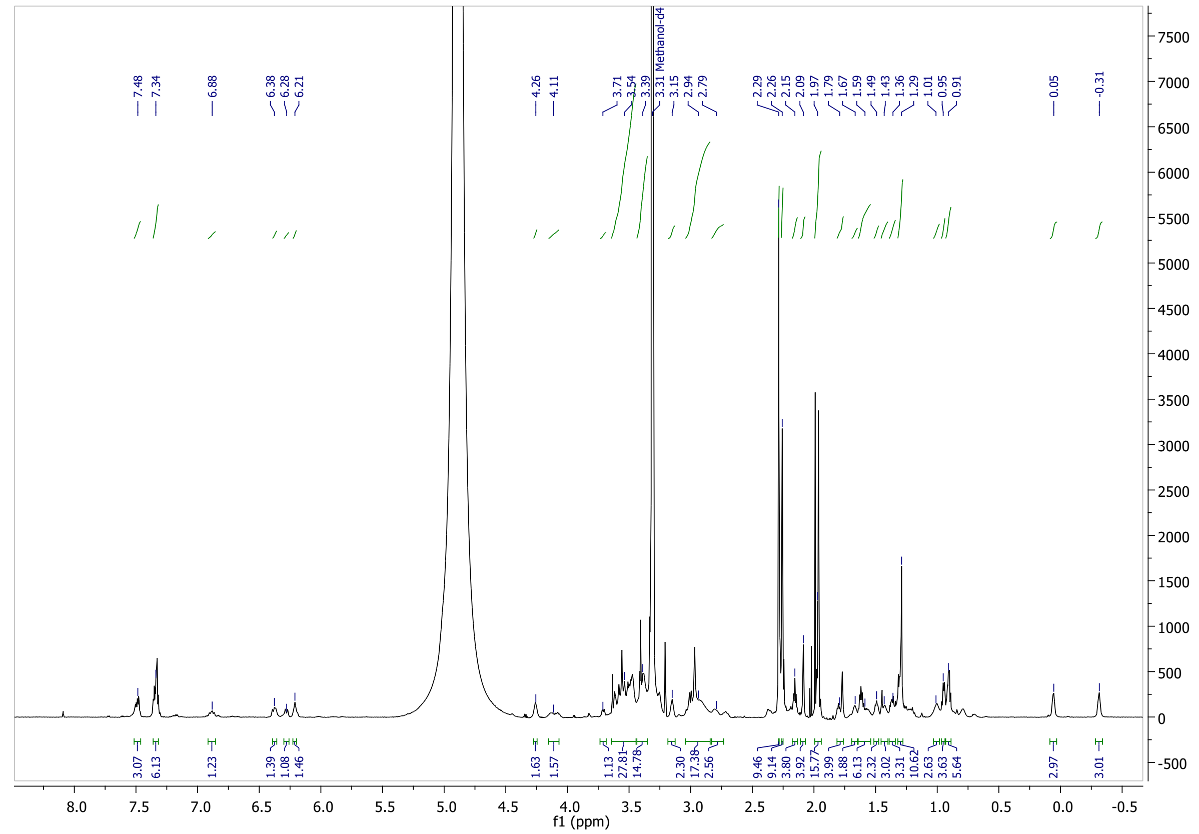

Compound **30**

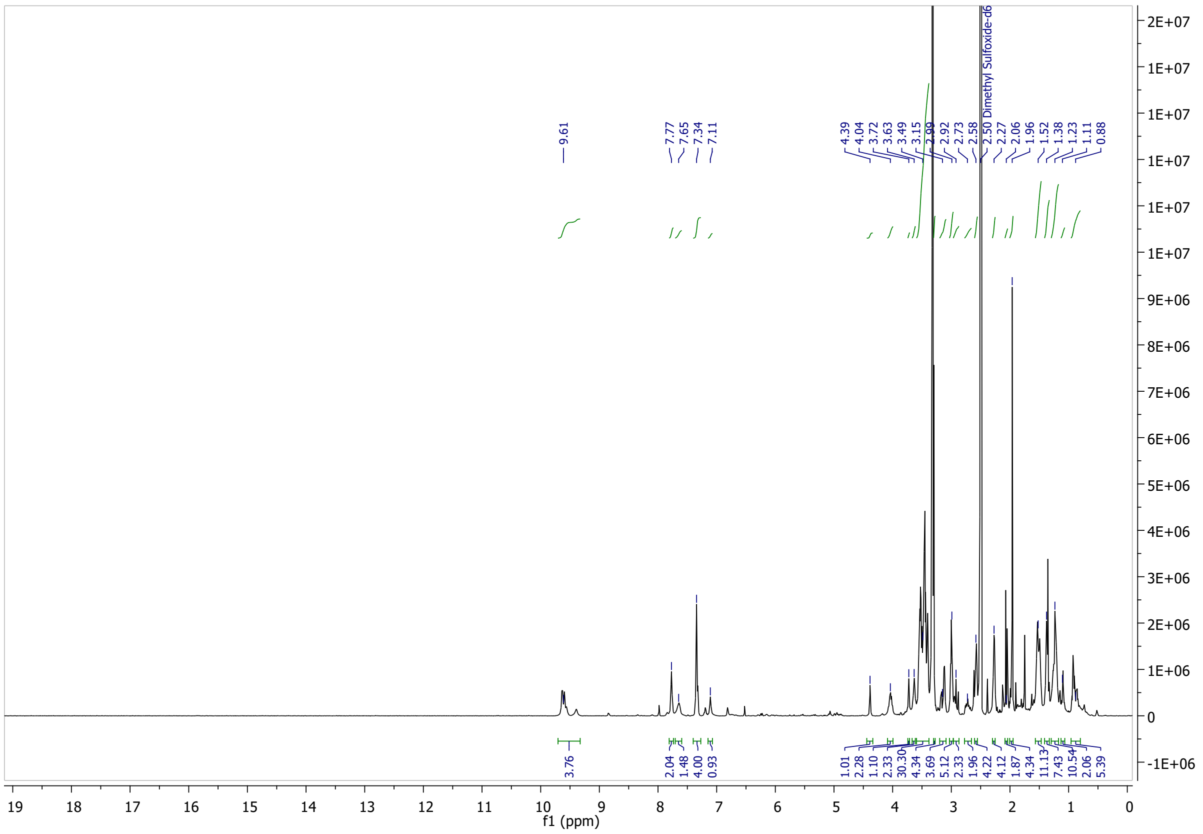

9.61
7.77
7.65
7.34
7.11
4.39
4.04
3.72
3.63
3.49
3.15
2.92
2.73
2.58
2.50 Dimethyl Sulfoxide-d6
2.27
2.06
1.96
1.52
1.38
1.23
1.11
0.88
3.76
2.04
1.48
4.00
0.93
1.01
2.28
1.10
2.33
30.30
4.34
3.69
5.12
2.33
1.96
4.22
4.12
1.87
4.34
11.13
7.43
10.54
2.06
5.39
f1 (ppm)

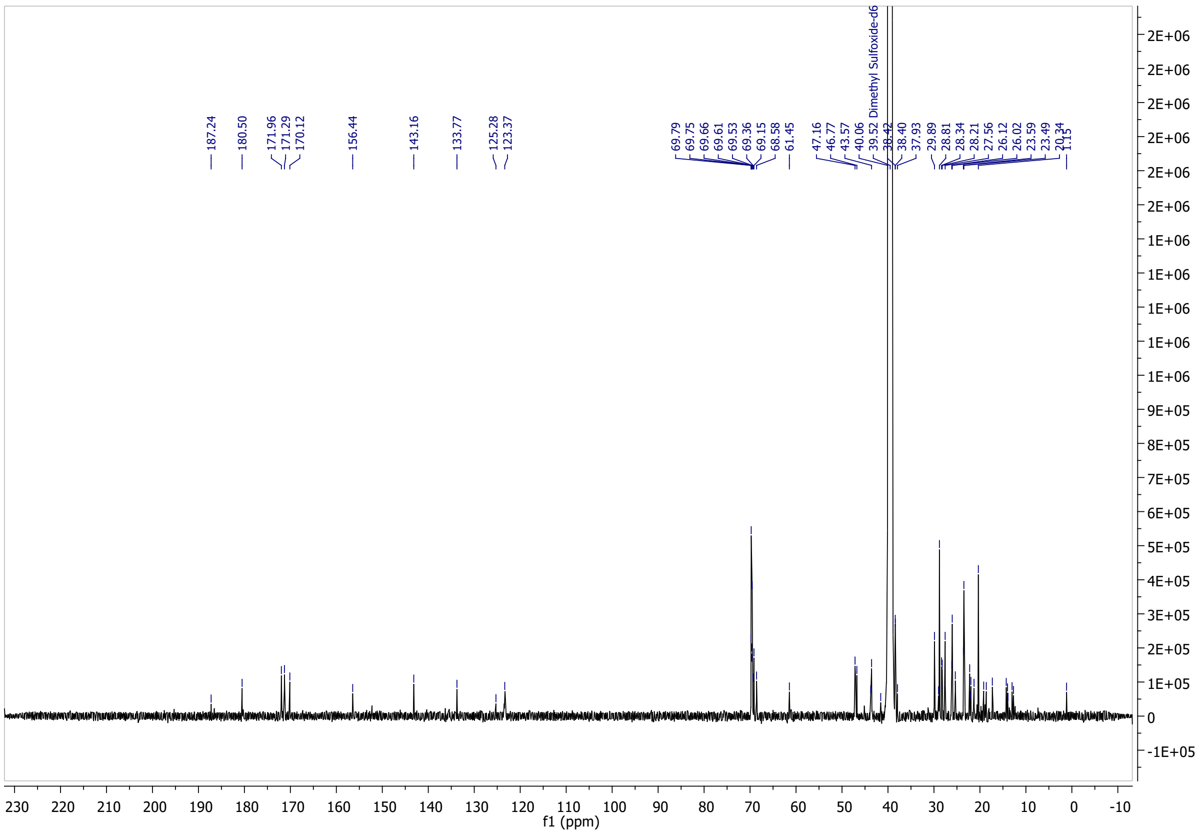

187.24
180.50
171.96
171.29
170.12
156.44
143.16
133.77
125.28
123.37
69.79
69.75
69.66
69.61
69.53
69.36
69.15
68.58
61.45
47.16
46.77
43.57
40.06
39.52 Dimethyl Sulfoxide-d6
38.42
38.40
37.93
29.89
28.81
28.34
28.21
27.56
26.12
26.02
23.59
23.49
20.34
1.15
f1 (ppm)

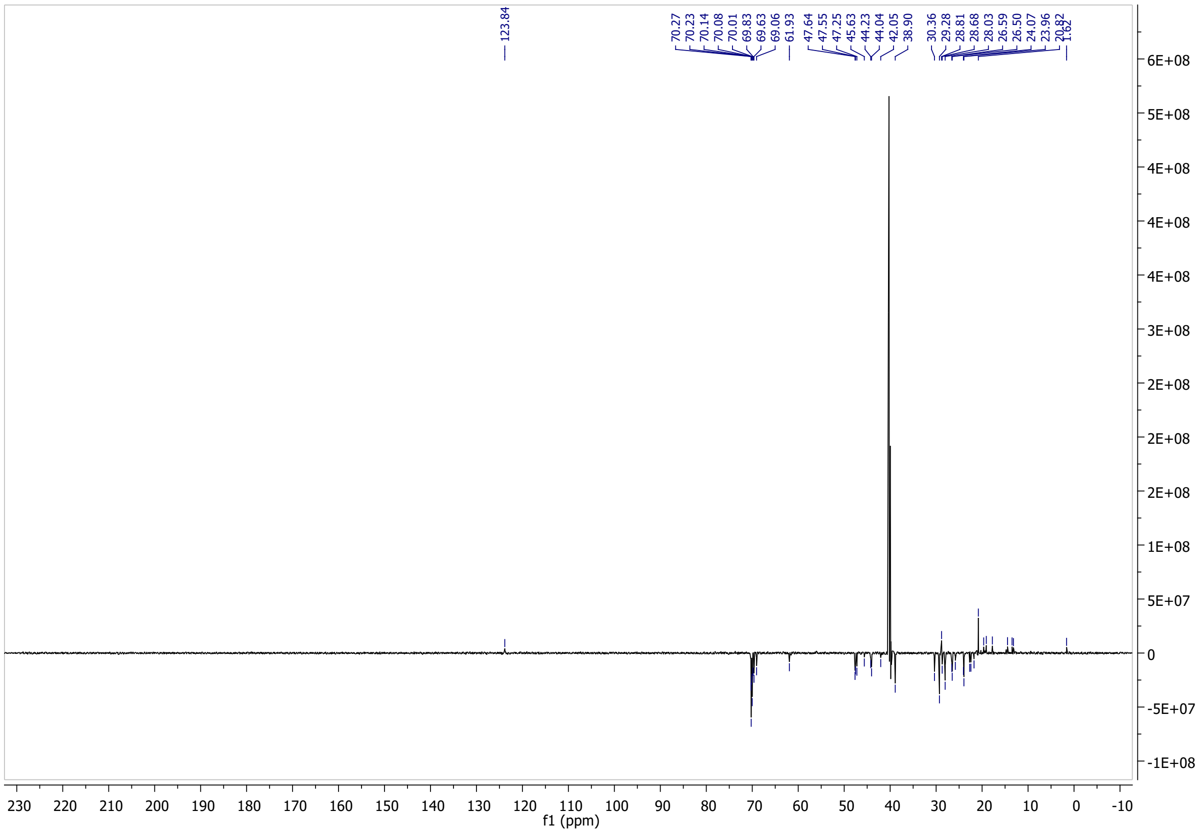

123.84
70.27
70.23
70.14
70.08
70.01
69.83
69.63
69.06
61.93
47.64
47.55
47.25
45.63
44.23
44.04
42.05
38.90
30.36
29.28
28.81
28.68
28.03
26.59
26.50
24.07
23.96
20.82
1.62
f1 (ppm)

Compound **30SL**

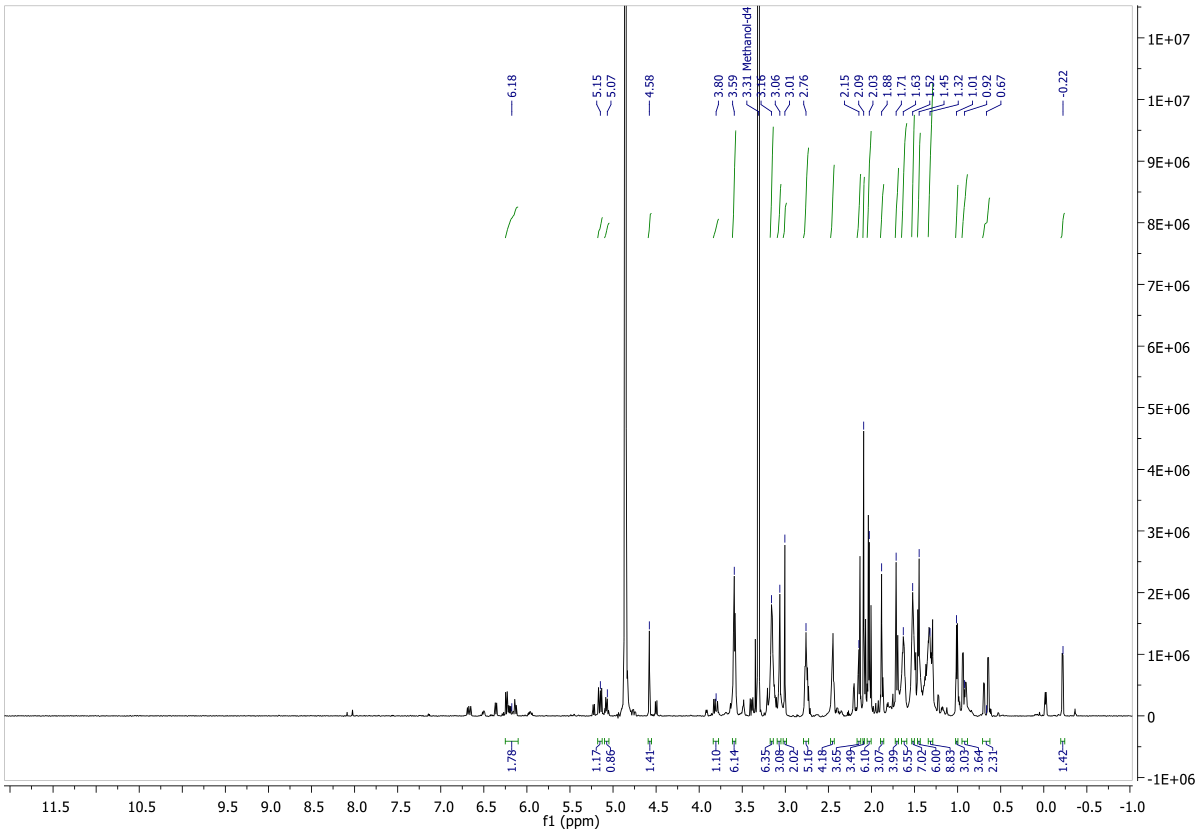

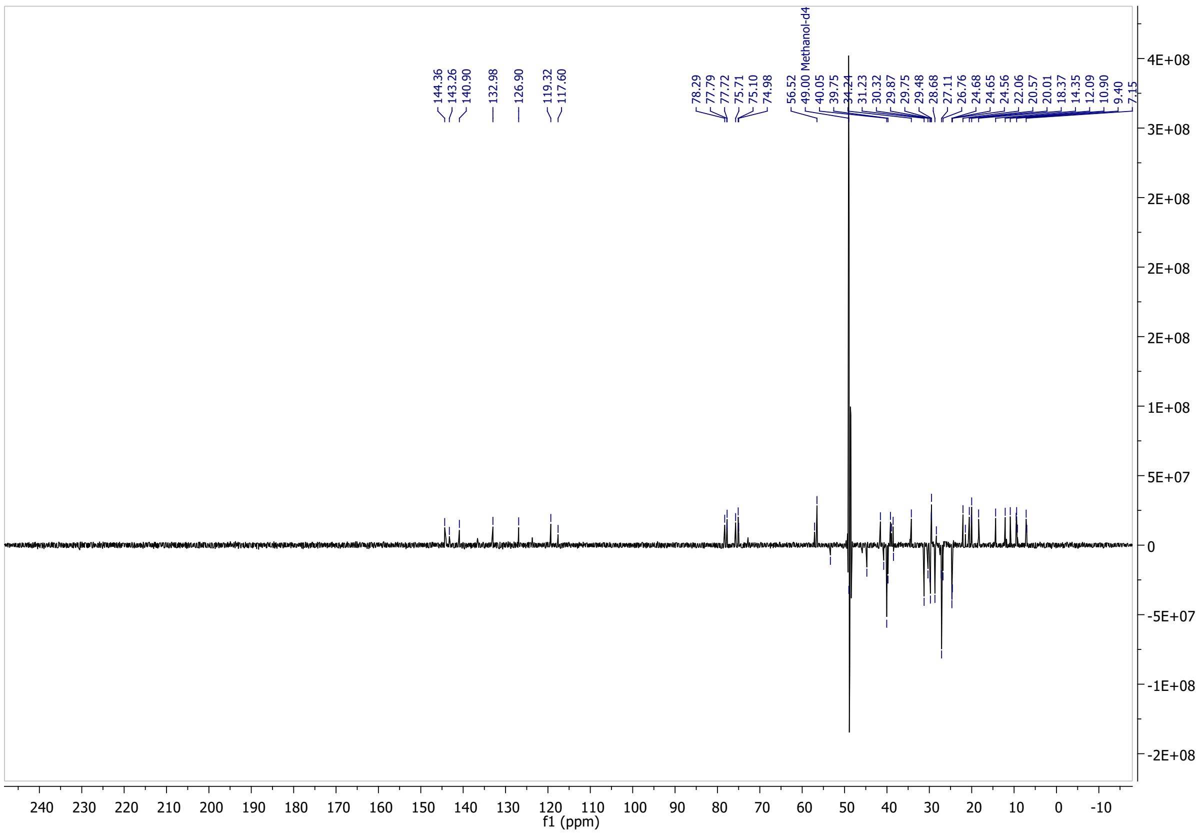

Compound **29**

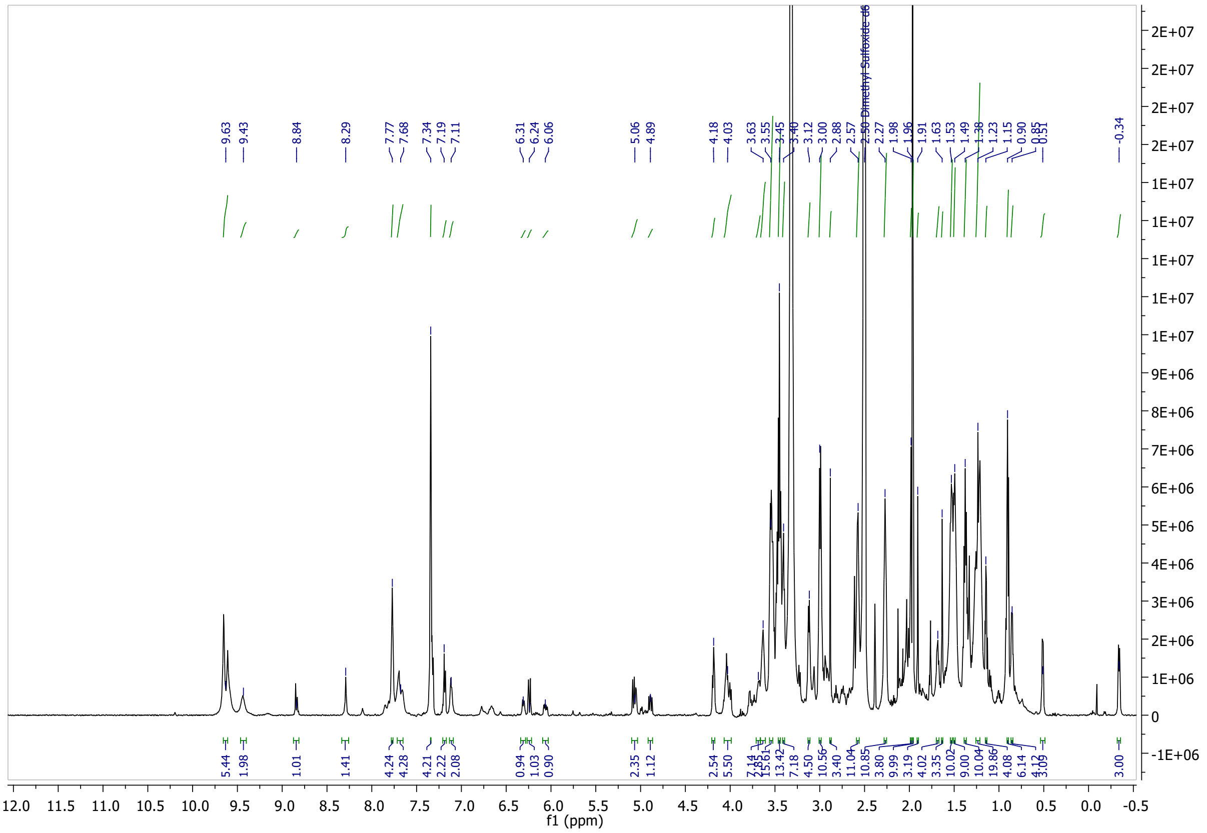

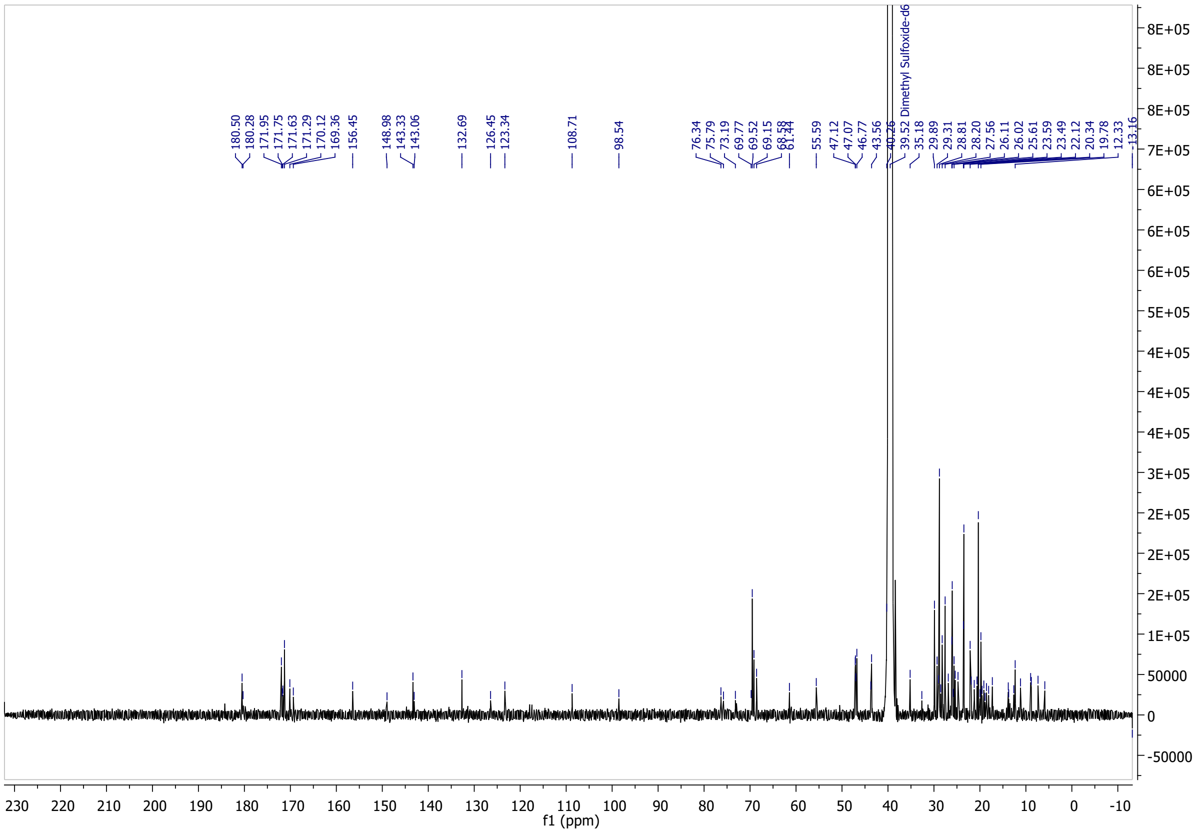

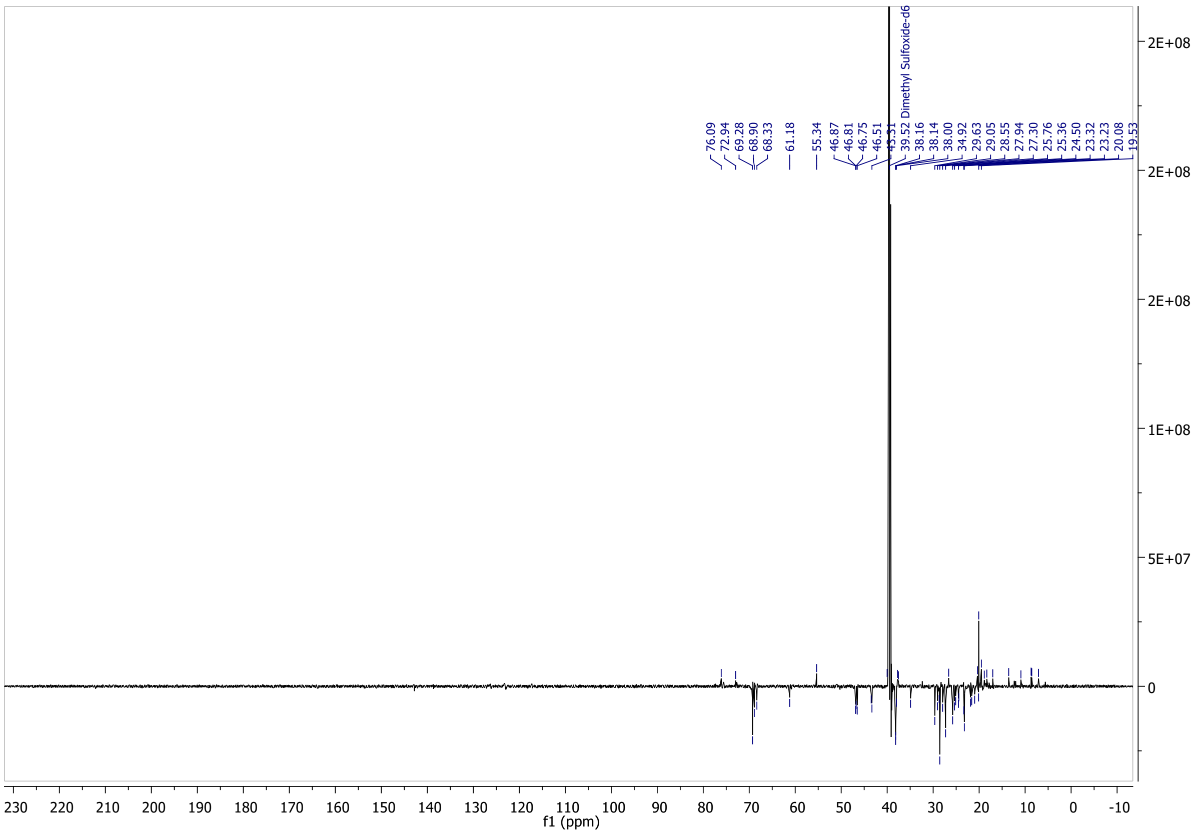
76.09
72.94
69.28
68.90
68.33
61.18
55.34
46.87
46.81
46.75
46.51
43.31
39.52 Dimethyl Sulfoxide-d6
38.16
38.14
38.00
34.92
29.63
29.05
28.55
27.94
27.30
25.76
25.36
24.50
23.32
23.23
20.08
19.53
2E+08
2E+08
2E+08
1E+08
5E+07
0
230 220 210 200 190 180 170 160 150 140 130 120 110 100 90 80 70 60 50 40 30 20 10 0 -10
f1 (ppm)

Compound **33**

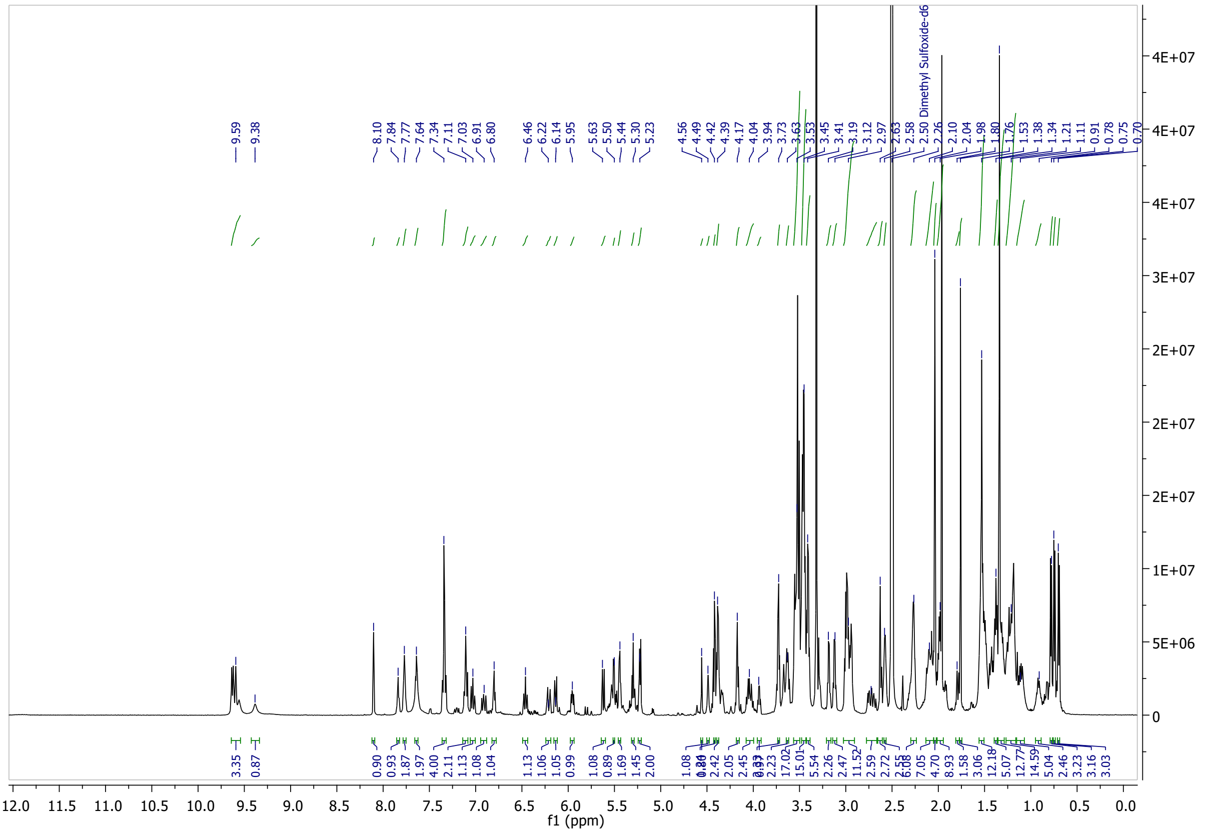

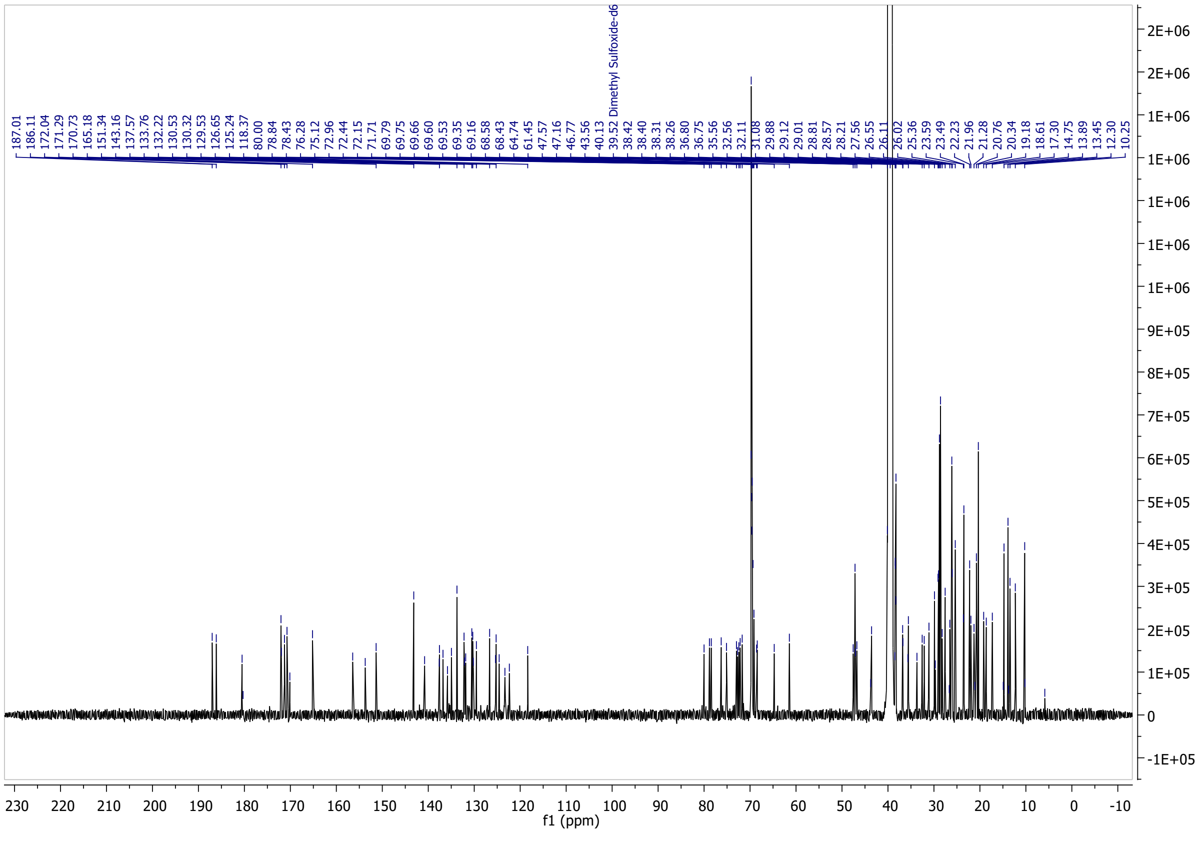

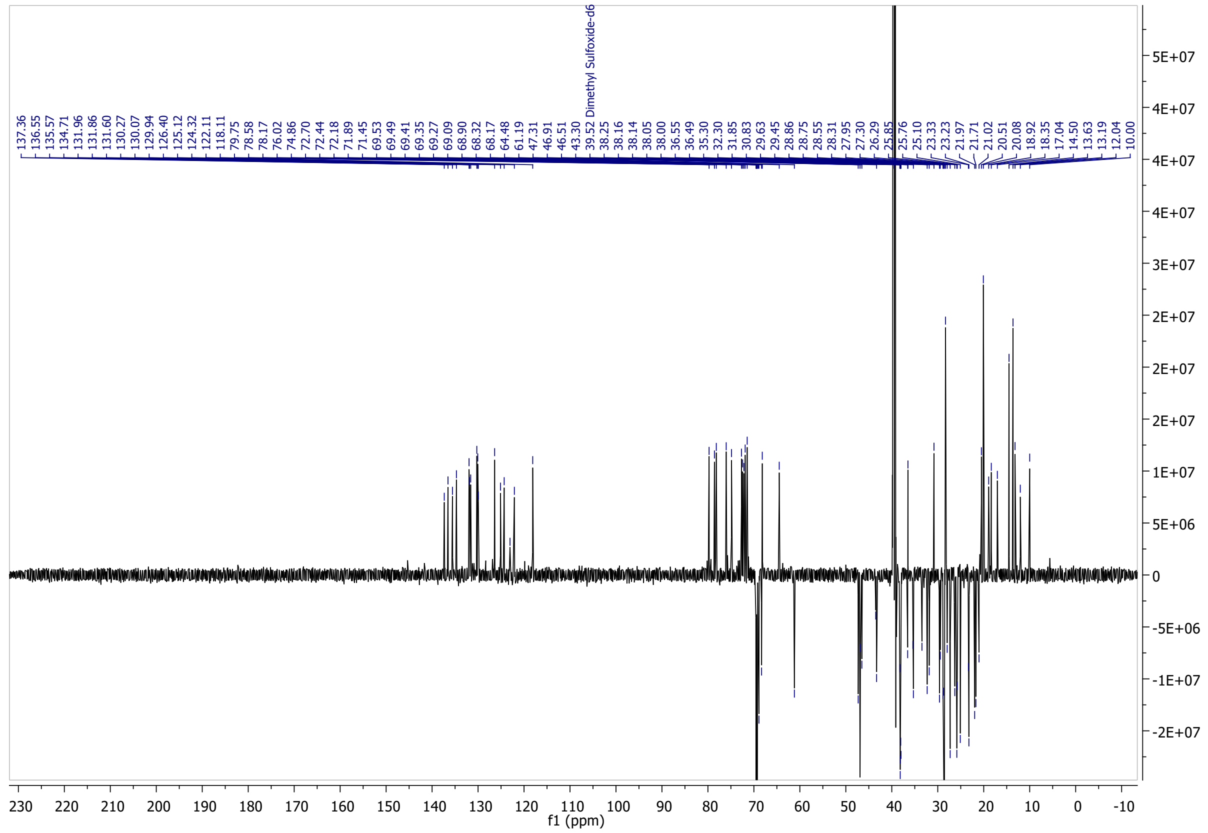
137.36
136.55
135.57
134.71
131.96
131.86
131.60
130.27
130.07
129.94
126.40
125.12
124.32
122.11
118.11
79.75
78.58
78.17
76.02
74.86
72.70
72.44
72.18
71.89
71.45
69.53
69.49
69.41
69.35
69.27
69.09
68.90
68.32
68.17
64.48
61.19
47.31
46.91
46.51
43.30
39.52 Dimethyl Sulfoxide-d6
38.25
38.16
38.14
38.05
38.00
36.55
36.49
35.30
32.30
31.85
30.83
29.63
29.45
28.86
28.75
28.55
28.31
27.95
27.30
26.29
25.85
25.76
25.10
23.33
23.23
21.97
21.71
21.02
20.51
20.08
18.92
18.35
17.04
14.50
13.63
13.19
12.04
10.00
f1 (ppm)

Compound **34**

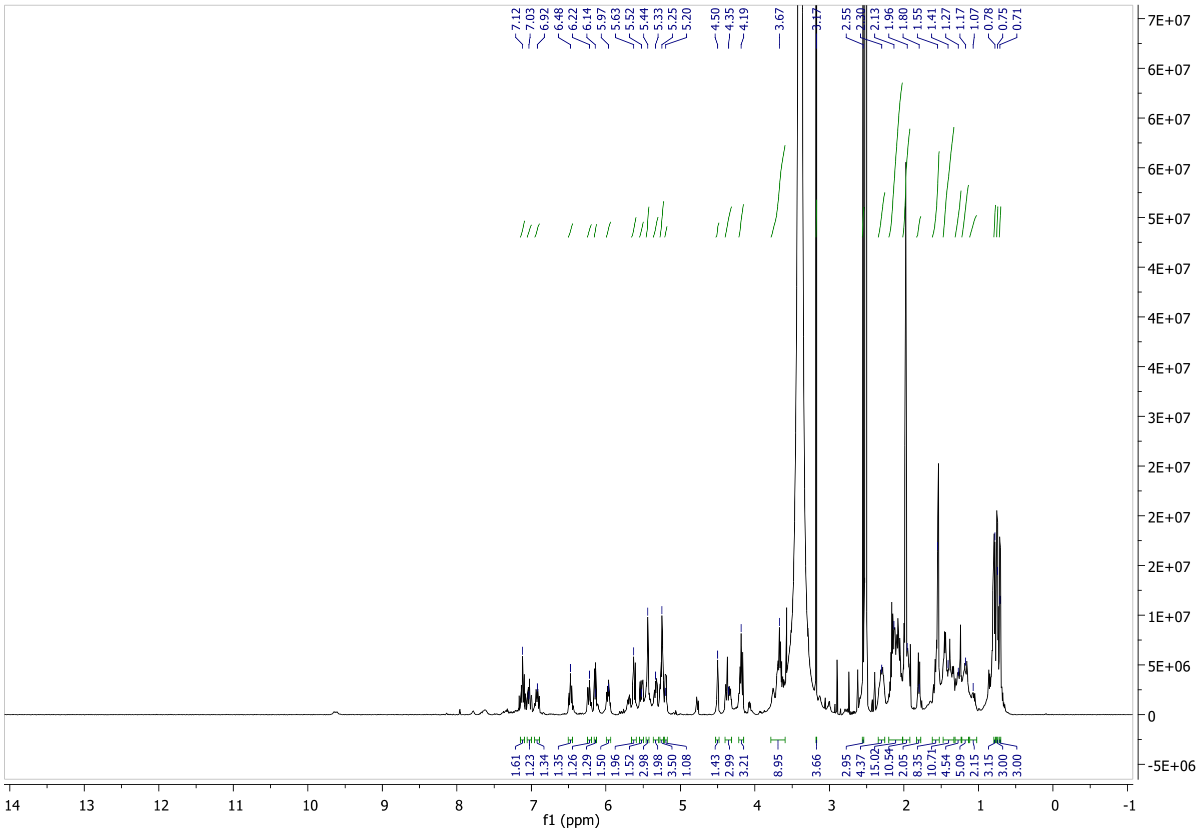

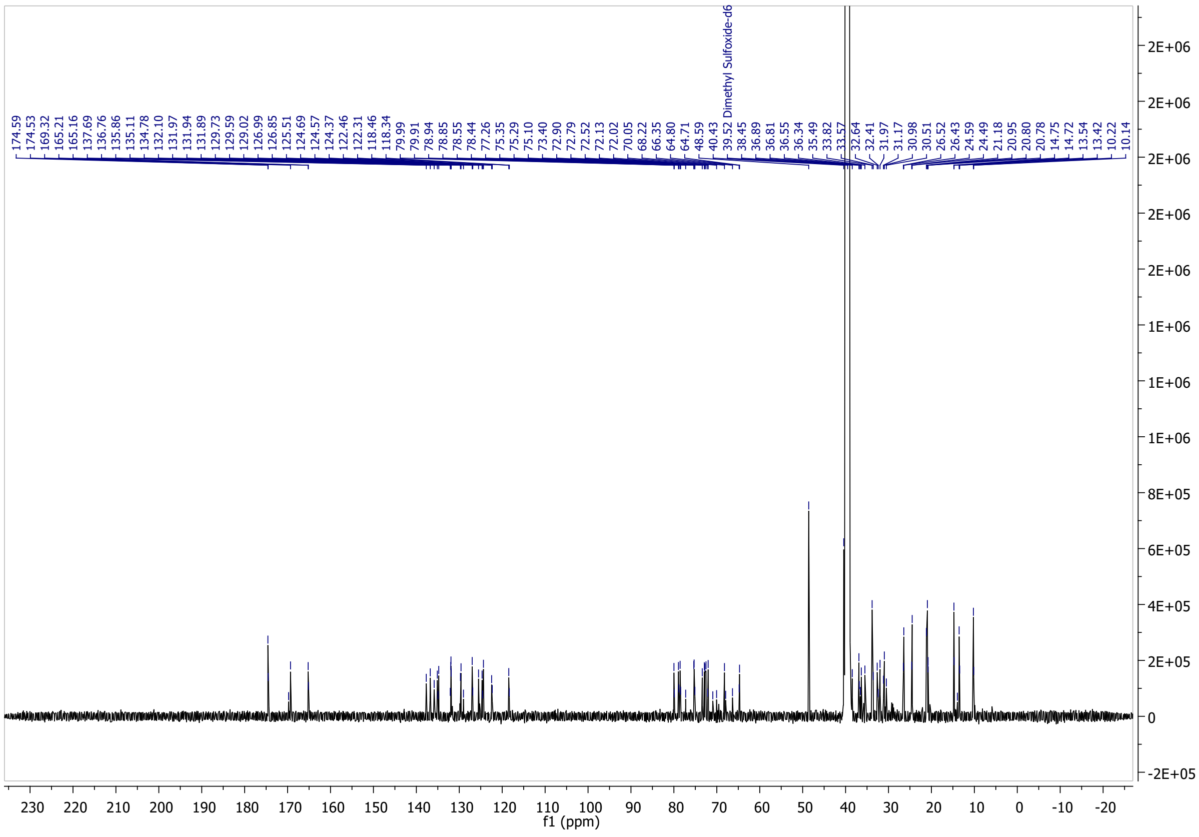

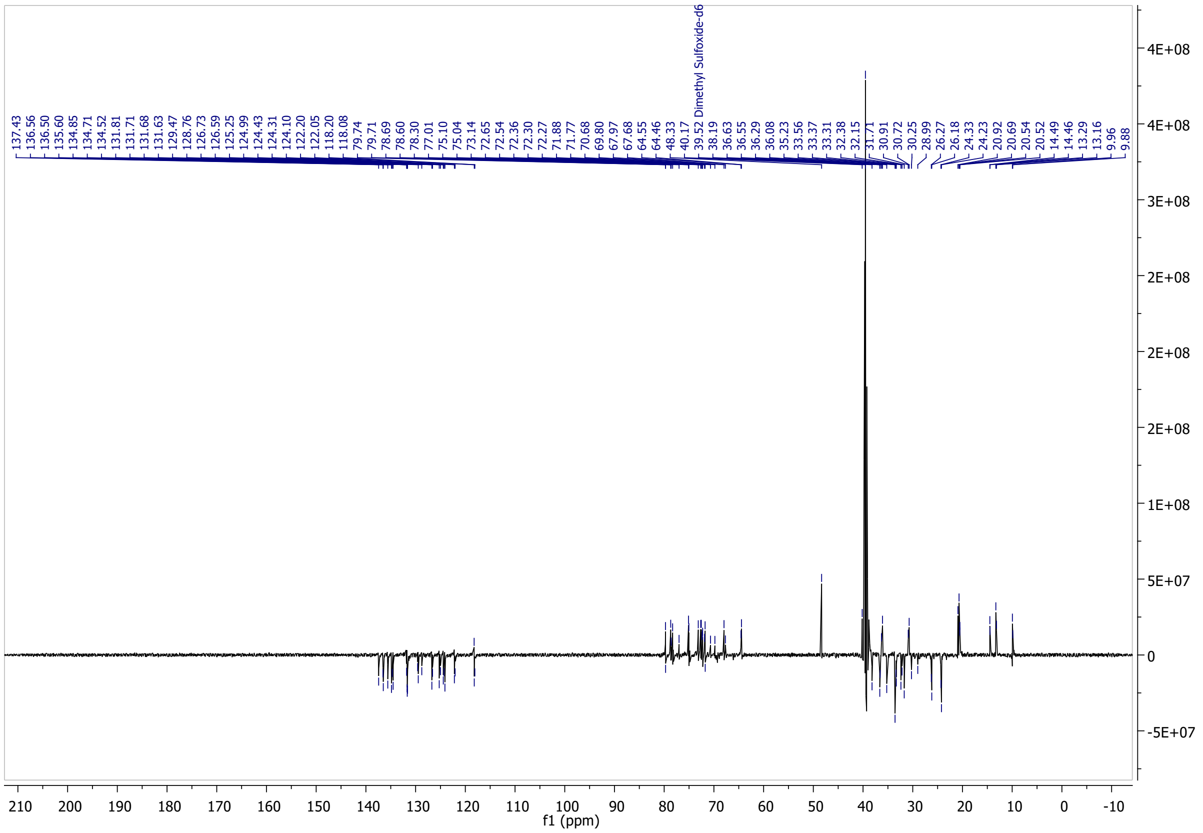
137.43
136.56
136.50
135.60
134.85
134.71
134.52
131.81
131.71
131.68
131.63
129.47
128.76
126.73
126.59
125.25
124.99
124.43
124.31
124.10
122.20
122.05
118.20
118.08
79.74
79.71
78.69
78.60
78.30
77.01
75.10
75.04
73.14
72.65
72.54
72.36
72.30
72.27
71.88
71.77
70.68
69.80
67.97
67.68
64.55
64.46
48.33
40.17
39.52 Dimethyl Sulfoxide-d6
38.19
36.63
36.55
36.29
36.08
35.23
33.56
33.37
33.31
32.38
32.15
31.71
30.91
30.72
30.25
28.99
26.27
26.18
24.33
24.23
20.92
20.69
20.54
20.52
14.49
14.46
13.29
13.16
9.96
9.88
4E+08
4E+08
3E+08
2E+08
2E+08
2E+08
1E+08
5E+07
0
-5E+07
210 200 190 180 170 160 150 140 130 120 110 100 90 80 70 60 50 40 30 20 10 0 -10
f1 (ppm)

Compound **36**

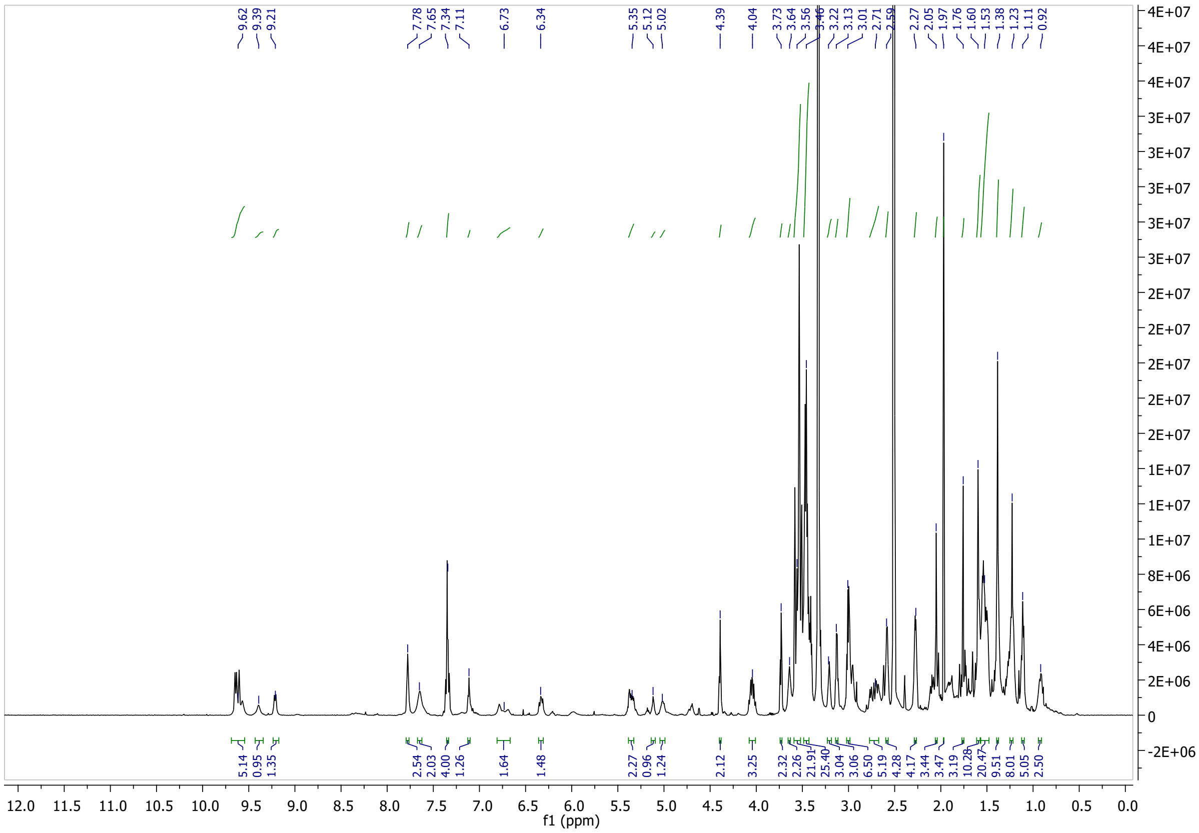

9.62
9.39
9.21
7.78
7.65
7.34
7.11
6.73
6.34
5.35
5.12
5.02
4.39
4.04
3.73
3.64
3.56
3.46
3.22
3.13
3.01
2.71
2.59
2.27
2.05
1.97
1.76
1.60
1.53
1.38
1.23
1.11
0.92
f1 (ppm)

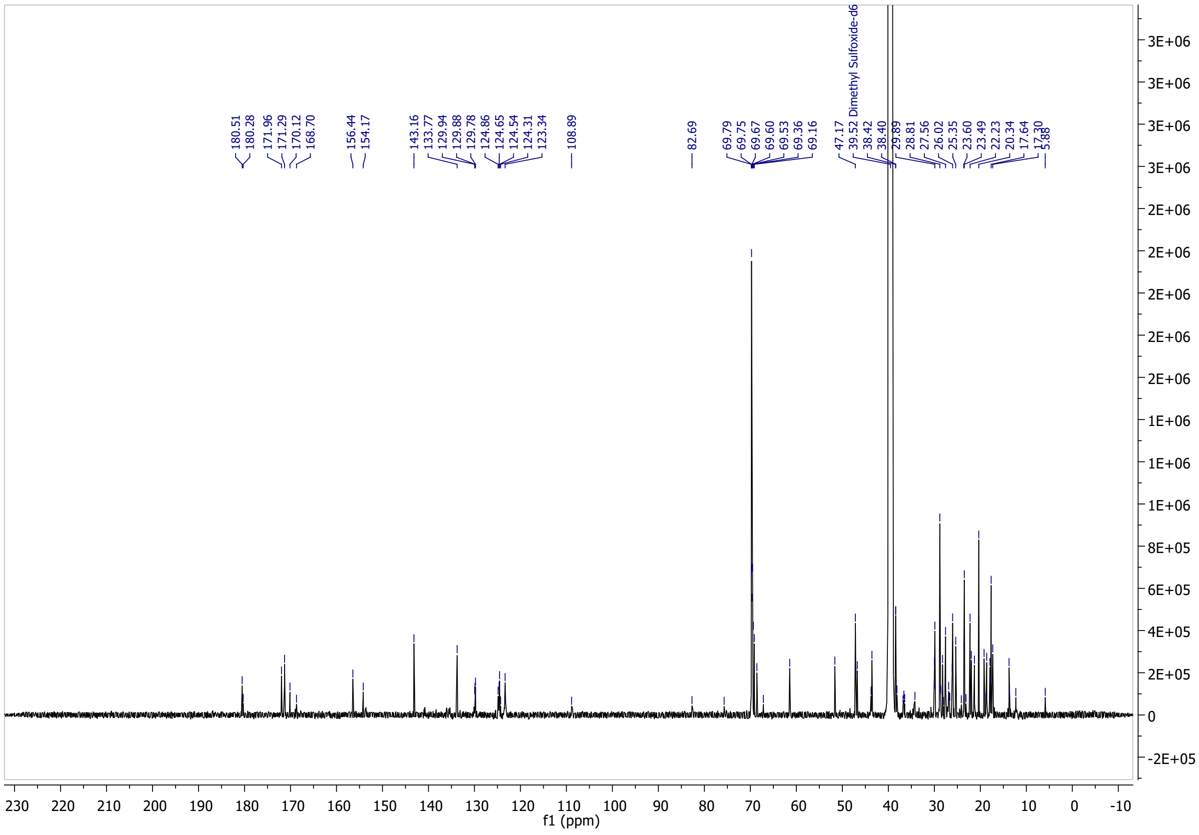

180.51
180.28
171.96
171.29
170.12
168.70
156.44
154.17
143.16
133.77
129.94
129.88
129.78
124.86
124.65
124.54
124.31
123.34
108.89
82.69
69.79
69.75
69.67
69.60
69.53
69.36
69.16
47.17
39.52 Dimethyl Sulfoxide-d6
38.42
38.40
f1 (ppm)

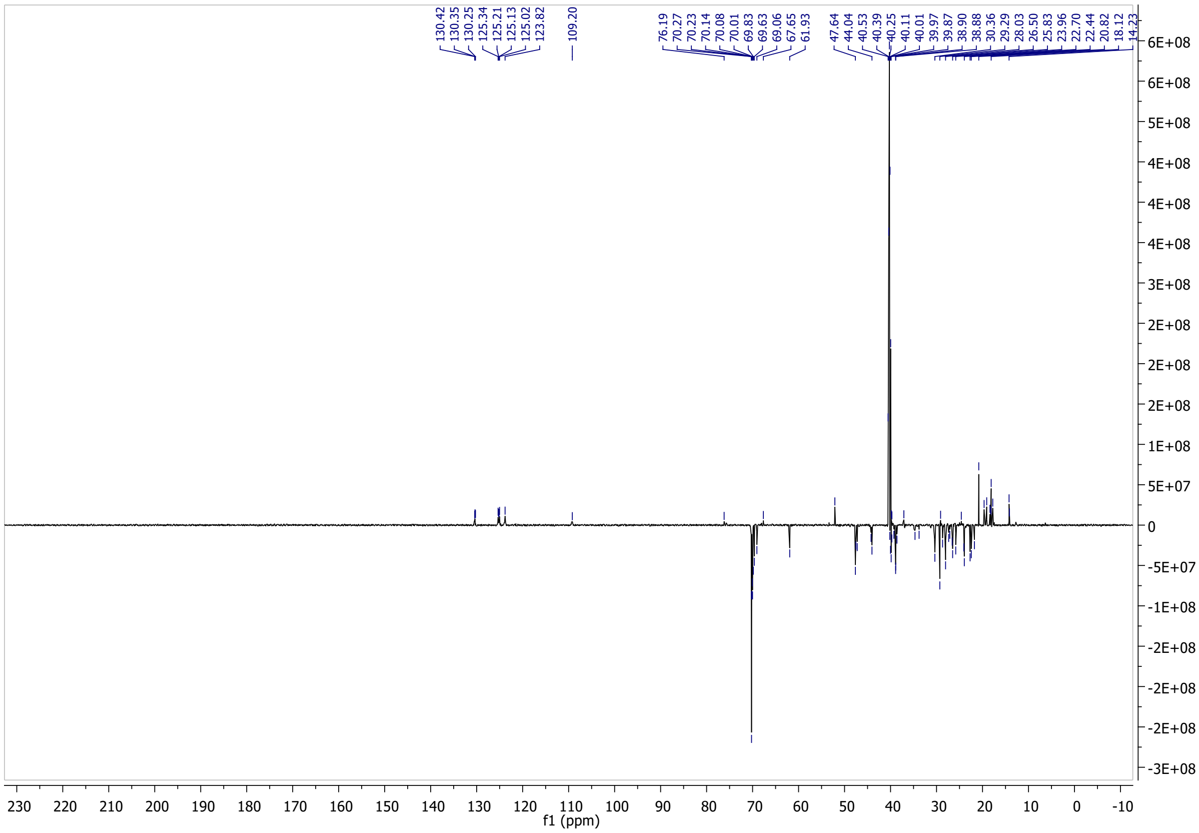
130.42
130.35
130.25
125.34
125.21
125.13
125.02
123.82
109.20
76.19
70.27
70.23
70.14
70.08
70.01
69.83
69.63
69.06
67.65
61.93
47.64
44.04
40.53
40.39
40.25
40.11
40.01
39.97
39.87
38.90
38.88
30.36
29.29
28.03
26.50
25.83
23.96
22.70
22.44
20.82
18.12
14.23
f1 (ppm)

Compound **37**

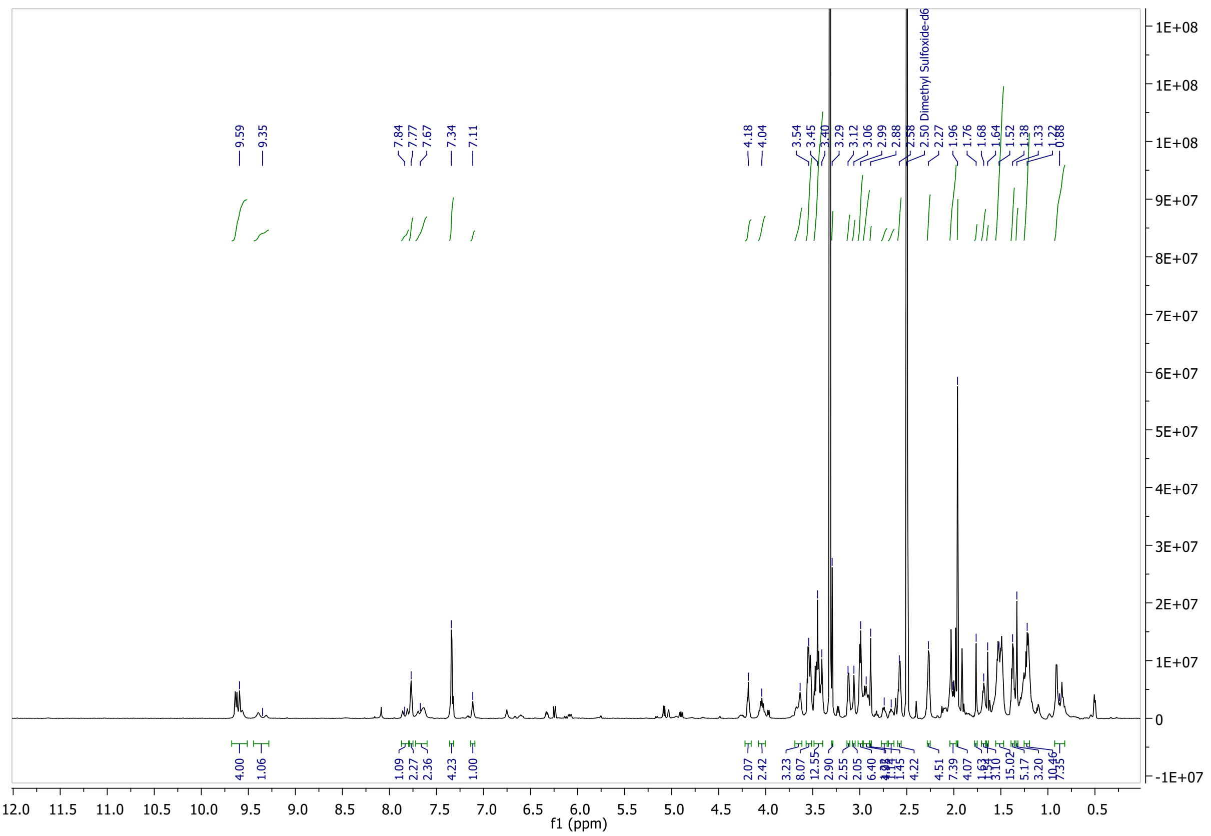

9.59
9.35
7.84
7.77
7.67
7.34
7.11
4.18
4.04
3.54
3.45
3.40
3.29
3.12
3.06
2.99
2.88
2.58
2.50 Dimethyl Sulfoxide-d6
2.27
1.96
1.76
1.68
1.64
1.52
1.38
1.33
1.22
0.88
f1 (ppm)

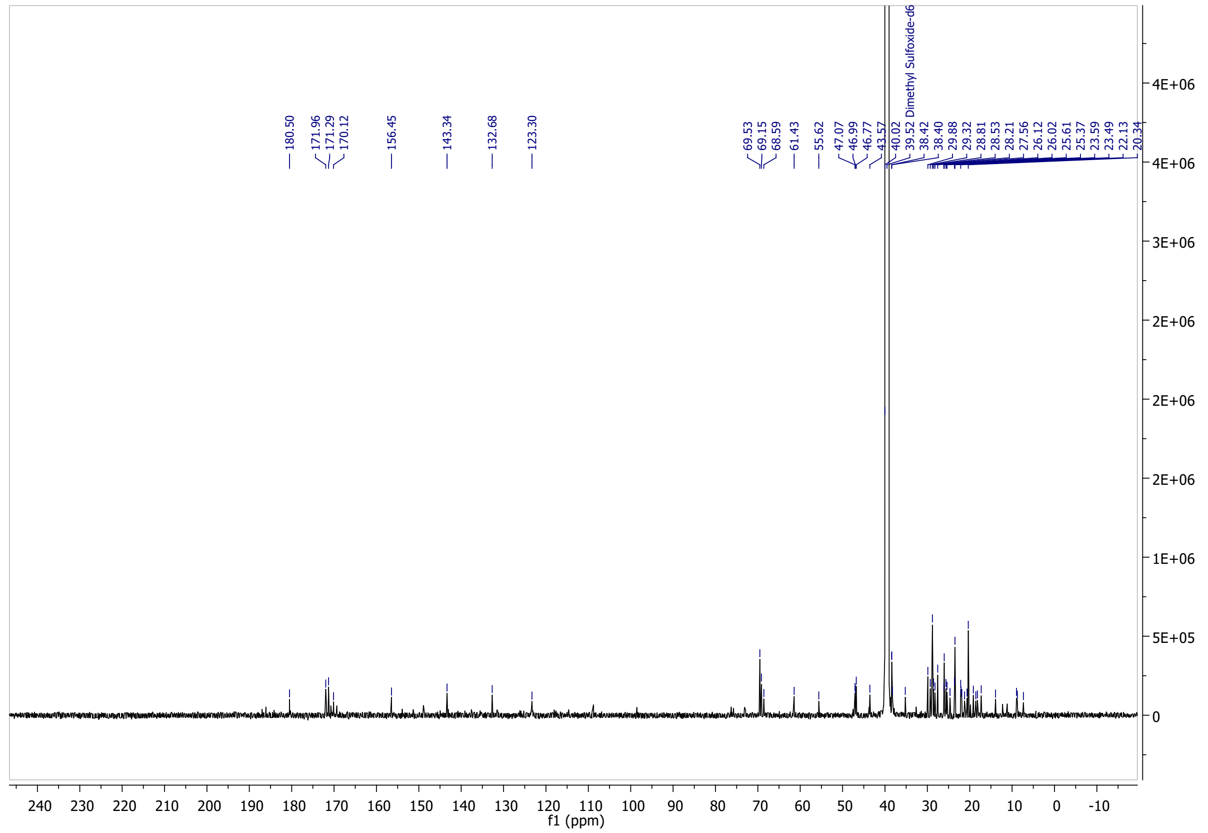

180.50
171.96
171.29
170.12
156.45
143.34
132.68
123.30
69.53
69.15
68.59
61.43
55.62
47.07
46.99
46.77
43.57
40.02
39.52 Dimethyl Sulfoxide-d6
38.42
38.40
29.88
29.32
28.81
28.53
28.21
27.56
26.12
26.02
25.61
25.37
23.59
23.49
22.13
20.34
f1 (ppm)

Compound 37SLa

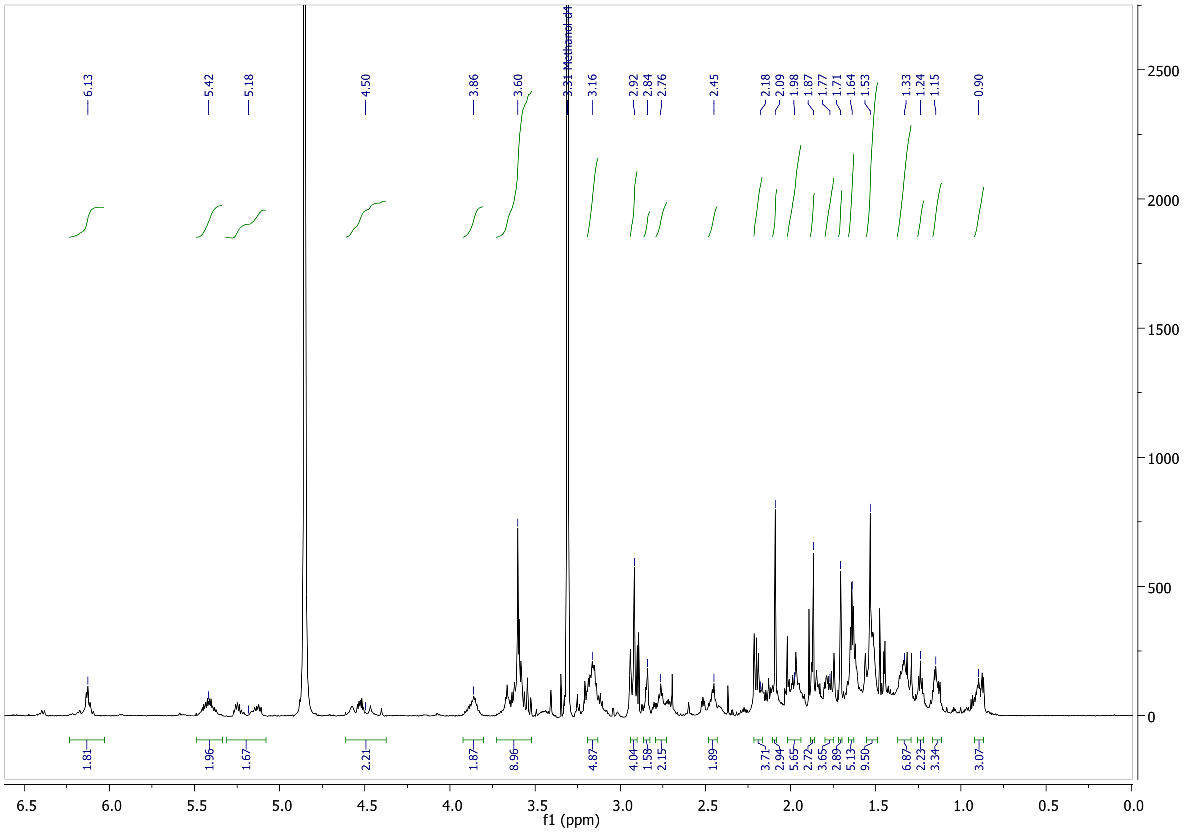

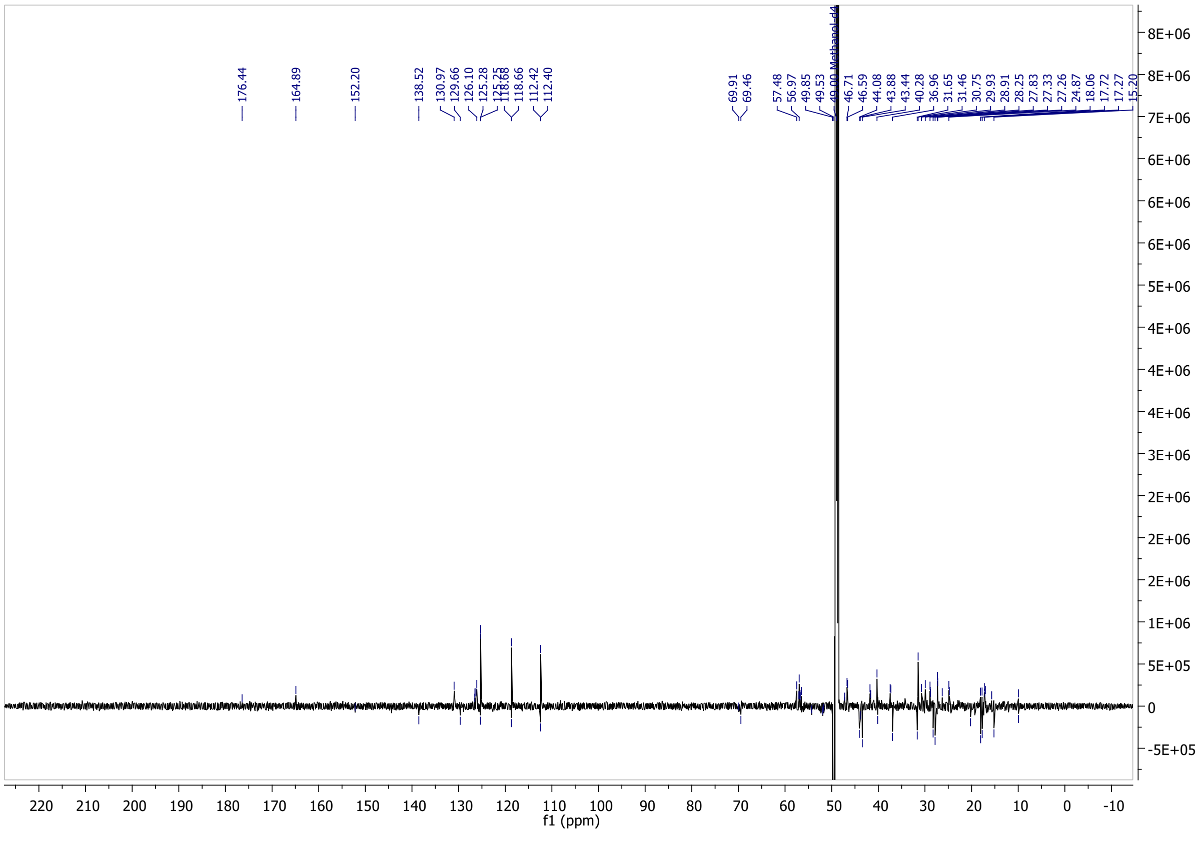

Compound **37SLb**

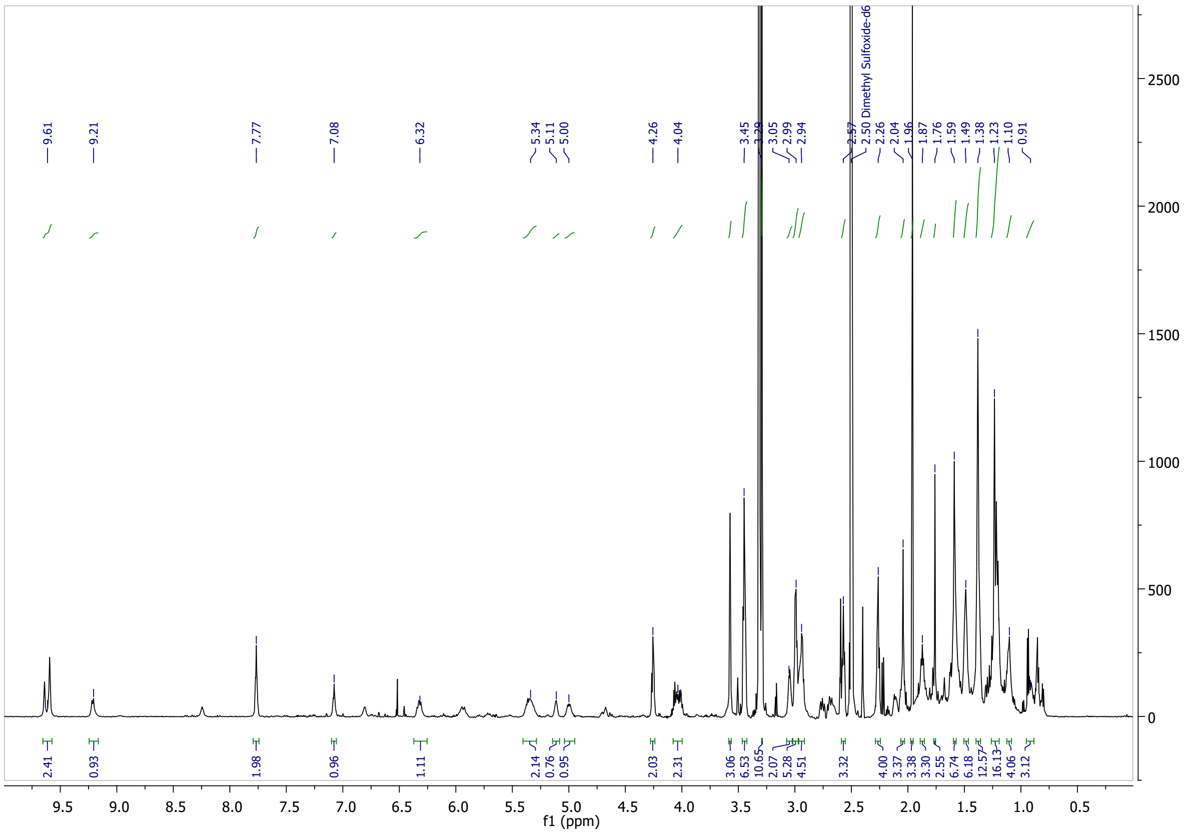

References

1. H. E. Gottlieb, V. Kotlyar and A. Nudelman, *J. Org. Chem.*, 1997, **62**, 7512-7515.
2. H. Irschik, R. Jansen, K. Gerth, G. Hofle and H. Reichenbach, *The Journal of antibiotics*, 1987, **40**, 7-13.
3. A. Schiefer, A. Schmitz, T. F. Schäberle, S. Specht, C. Lämmer, K. L. Johnston, D. G. Vassylyev, G. M. König, A. Hoerauf and K. Pfarr, *J. Infect. Dis.*, 2012, **206**, 249-257.
4. C. Peukert, L. N. B. Langer, S. M. Wegener, A. Tutov, J. P. Bankstahl, B. Karge, F. M. Bengel, T. L. Ross and M. Brönstrup, *J. Med. Chem.*, 2021, **64**, 12359-12378.
5. C. Ji and M. J. Miller, *BioMetals*, 2015, **28**, 541-551.
6. C. Peukert, L. N. B. Langer, S. M. Wegener, A. Tutov, J. P. Bankstahl, B. Karge, F. M. Bengel, T. L. Ross and M. Brönstrup, *J. Med. Chem.*, 2021, DOI: 10.1021/acs.jmedchem.1c01054.
7. *worldwide Pat.,* 2015.
8. *worldwide Pat.,* 2017.
9. R. A. Adams, G. Leon, N. M. Miller, S. P. Reyes, C. H. Thantrong, A. M. Thokkadam, A. S. Lemma, D. M. Sivaloganathan, X. Wan and M. P. Brynildsen, *J. Antibiot.*, 2021, **74**, 786-798.
10. N. Mori, Y. Ishii, K. Tateda, S. Kimura, Y. Kouyama, H. Inoko, S. Mitsunaga, K. Yamaguchi and E. Yoshihara, *J. Antimicrob. Chemother.*, 2012, **67**, 2173-2181.
11. B. Lee, J. Yan, A. Ulhaq, S. Miller, W. Seo, P. Lu, R. She, B. Spellberg, B. Luna and P. A. Bradford, *mSphere*, 2021, **6**, e00920-00921.
12. D. M. Rothstein, C. Shalish, C. K. Murphy, A. Sternlicht and L. A. Campbell, *Expert Opin. Investig. Drugs*, 2006, **15**, 603-623.
13. R. Jansen, D. Schummer, H. Irschik and G. Höfle, *Liebigs Ann.*, 1990, **1990**, 975-988.
14. K. A. Pardeshi, T. A. Kumar, G. Ravikumar, M. Shukla, G. Kaul, S. Chopra and H. Chakrapani, *Bioconj. Chem.*, 2019, **30**, 751-759.
15. C. Peukert, S. Popat Gholap, O. Green, L. Pinkert, J. van den Heuvel, M. van Ham, D. Shabat and M. Brönstrup, *Angew. Chem. Int. Ed.*, 2022, **n/a**, e202201423.
16. L. Pinkert, Y.-H. Lai, C. Peukert, S.-K. Hotop, B. Karge, L. M. Schulze, J. Grunenberg and M. Brönstrup, *J. Med. Chem.*, 2021, **64**, 15440-15460.
17. C. Peukert, S. Popat Gholap, O. Green, L. Pinkert, J. van den Heuvel, M. van Ham, D. Shabat and M. Brönstrup, *Angew. Chem. Int. Ed.*, 2022, **61**, e202201423.
18. J. M. Andrews, *J. Antimicrob. Chemother.*, 2001, **48**, 5-16.
19. K. Ferreira, H.-Y. Hu, V. Fetz, H. Prochnow, B. Rais, P. P. Müller and M. Brönstrup, *Angew. Chem. Int. Ed.*, 2017, **56**, 8272-8276.
20. L. Pinkert, Y.-H. Lai, C. Peukert, S.-K. Hotop, B. Karge, L. M. Schulze, J. Grunenberg and M. Brönstrup, *J. Med. Chem.*, 2021, **64**, 15440-15460.
21. BacDive, *E. coli W 10.13145/bacdive4428.20211221.6*, accessed 17.01.2022, DOI: 10.13145/bacdive4428.20211221.6.
22. BacDive, *Staphylococcus aureus DSM 11822, 10.13145/bacdive14461.20211221.6*, accessed 17.01.2022, DOI: 10.13145/bacdive14461.20211221.6.
23. K. p. s. p. C. BacDive, *Klebsiella pneumoniae subsp. pneumoniae C122, 10.13145/bacdive4955.20211221.6*, accessed 17.01.2022, DOI: 10.13145/bacdive4955.20211221.6.
24. BacDive, *Acinetobacter baumannii 2208 10.13145/bacdive8093.20211221.6*, accessed 17.01.2022, DOI: 10.13145/bacdive8093.20211221.6.
25. BacDive, *Acinetobacter baumannii 2197, 10.13145/bacdive8094.20220920.7*, accessed 17.01.2022, DOI: 10.13145/bacdive8093.20211221.6.
26. BacDive, *Pseudomonas aeruginosa PAO, 10.13145/bacdive12763.20211221.6*, accessed 17.01.2022, DOI: 10.13145/bacdive12763.20211221.6.
27. BacDive, *Enterococcus faecium DSM 20477 10.13145/bacdive5301.20211221.6*, accessed 17.01.2022, DOI: 10.13145/bacdive5301.20211221.6.
28. V. Gasser, E. Baco, O. Cunrath, P. S. August, Q. Perraud, N. Zill, C. Schleberger, A. Schmidt, A. Paulen, D. Bumann, G. L. A. Mislin and I. J. Schalk, *Environ. Microbiol.*, 2016, **18**, 819-832.

29. H. Prochnow, V. Fetz, S. K. Hotop, M. A. García-Rivera, A. Heumann and M. Brönstrup, *Anal. Chem.*, 2019, **91**, 1863-1872.
30. A. K. Krome, T. Becker, S. Kehraus, A. Schiefer, M. Gütschow, L. Chaverra-Muñoz, S. Hüttel, R. Jansen, M. Stadler, A. Ehrens, D. Pogorevc, R. Müller, M. P. Hübner, T. Hesterkamp, K. Pfarr, A. Hoerauf, K. G. Wagner and G. M. König, *Nat. Prod. Rep.*, 2022, **39**, 1705-1720.
31. R. Jansen, G. Höfle, H. Irschik and H. Reichenbach, *Liebigs Ann. Chem.*, 1985, **1985**, 822-836.
